# A Genome-wide Combinatorial Strategy Dissects Complex Genetic Architecture of Seed Coat Color in Chickpea

**DOI:** 10.3389/fpls.2015.00979

**Published:** 2015-11-17

**Authors:** Deepak Bajaj, Shouvik Das, Hari D. Upadhyaya, Rajeev Ranjan, Saurabh Badoni, Vinod Kumar, Shailesh Tripathi, C. L. Laxmipathi Gowda, Shivali Sharma, Sube Singh, Akhilesh K. Tyagi, Swarup K. Parida

**Affiliations:** ^1^National Institute of Plant Genome ResearchNew Delhi, India; ^2^International Crops Research Institute for the Semi-Arid TropicsTelangana, India; ^3^National Research Centre on Plant BiotechnologyNew Delhi, India; ^4^Division of Genetics, Indian Agricultural Research InstituteNew Delhi, India

**Keywords:** chickpea, GBS, GWAS, haplotype, QTL, seed coat color, SNP, wild accession

## Abstract

The study identified 9045 high-quality SNPs employing both genome-wide GBS- and candidate gene-based SNP genotyping assays in 172, including 93 cultivated (*desi* and *kabuli*) and 79 wild chickpea accessions. The GWAS in a structured population of 93 sequenced accessions detected 15 major genomic loci exhibiting significant association with seed coat color. Five seed color-associated major genomic loci underlying robust QTLs mapped on a high-density intra-specific genetic linkage map were validated by QTL mapping. The integration of association and QTL mapping with gene haplotype-specific LD mapping and transcript profiling identified novel allelic variants (non-synonymous SNPs) and haplotypes in a MATE secondary transporter gene regulating light/yellow brown and beige seed coat color differentiation in chickpea. The down-regulation and decreased transcript expression of beige seed coat color-associated MATE gene haplotype was correlated with reduced proanthocyanidins accumulation in the mature seed coats of beige than light/yellow brown seed colored *desi* and *kabuli* accessions for their coloration/pigmentation. This seed color-regulating MATE gene revealed strong purifying selection pressure primarily in LB/YB seed colored *desi* and wild *Cicer reticulatum* accessions compared with the BE seed colored *kabuli* accessions. The functionally relevant molecular tags identified have potential to decipher the complex transcriptional regulatory gene function of seed coat coloration and for understanding the selective sweep-based seed color trait evolutionary pattern in cultivated and wild accessions during chickpea domestication. The genome-wide integrated approach employed will expedite marker-assisted genetic enhancement for developing cultivars with desirable seed coat color types in chickpea.

## Introduction

Chickpea (*Cicer arietinum* L.) is the third largest produced food legume crop globally that serves as a vital human dietary source of protein enriched with essential amino acids (Kumar et al., 2011; Gaur et al., 2012; Varshney et al., 2013). The seeds are defining characteristics of chickpea aside from having high economic value. The physical appearance of seeds such as light colored and large sized seeds of chickpea has always been a trait of consumer preference and trade value, besides an important target quality component and adaptation traits (Cassells and Caddick, 2000; Graham et al., 2001; Hossain et al., 2010). The seed coat color is an important agronomic trait in chickpea that varies widely among core and mini-core germplasm collections, cultivated *desi* and *kabuli* types, landraces and wild species accessions (Upadhyaya and Ortiz, 2001; Upadhyaya et al., 2002, 2006). Considering the agronomic importance and wider level of existing phenotypic variation of seed coat color in chickpea, it is essential to identify the underlying heritable forces and potential genes/QTLs (quantitative trait loci) influencing this large trait variation in *desi* and *kabuli* chickpea. Based on defined agro-morphological descriptors, the large-scale phenotyping of core, mini-core and reference core chickpea accessions have classified and characterized these accessions into 24 diverse classes of seed coat color types (like brown, light brown, green, yellow-brown and creamy) (Upadhyaya and Ortiz, 2001; Upadhyaya et al., 2002, 2008). This revealed the predominance of yellow/light brown and beige seed coat color in *desi* and *kabuli* chickpea.

Tremendous efforts have been made for studying the genetic inheritance pattern as well as identification and molecular cloning of multiple genes harboring the major QTLs regulating seed coat color trait in diverse crop plants, including rice, soybean, sesame, *Brassica* and grape (Furukawa et al., 2006; Liu et al., 2006; Sweeney et al., 2006, 2007; Yang et al., 2010; Rahman et al., 2011; Huang et al., 2012; Zhang et al., 2013). The formation of different seed coat color is governed by organ/tissue-specific accumulation of plant secondary metabolites like flavonoid compounds, including anthocyanin, flavonols, isoflavones, flavonol glucosides, phlobaphenes, proanthocyanidin, leucoanthocyanidin, and proanthocyanidin in the seed coat (Furukawa et al., 2006). A complex biochemical pathway like phenylpropanoid (flavonoid and anthocyanin biosynthetic pathway) pathway is known to be involved in synthesis of these secondary metabolites in the specific tissues of crop plants (Dixon et al., 2002). The advances in structural and functional genomics have greatly catalyzed isolation of most of the transcriptionally regulated loci/genes that encode enzymes of this important biosynthetic pathway governing seed coat color in rice, maize, *Arabidopsis, Brassica, Medicago*, common bean, pea *Antirrhinum* and *Petunia* (Ludwig et al., 1989; Mol et al., 1998; Hu et al., 2000; Forkmann and Martens, 2001; Sakamoto et al., 2001; Winkel-Shirley, 2001; Saitoh et al., 2004; Xie and Dixon, 2005; Furukawa et al., 2006; Lepiniec et al., 2006; Quattrocchio et al., 2006; Zhao and Dixon, 2009; Golam Masum Akond et al., 2011; Li et al., 2012; Ferraro et al., 2014; Liu et al., 2014). However, the genetic analysis of seed color trait has mostly been restricted to estimation of heritability and molecular mapping of only a few seed coat color-associated QTLs because of its quantitative and polygenic nature of inheritance (epistatic and pleiotropic effects) both in *desi* and *kabuli* chickpea (Hossain et al., 2010). However, the markers associated with these QTLs controlling seed color are yet to be validated across diverse genetic backgrounds and environments for their deployment in marker-assisted breeding program to develop improved chickpea cultivars with desirable seed coat color.

A combinatorial approach of SNP (single nucleotide polymorphism) and SSR (simple sequence repeat) marker-based genome-wide association study (GWAS), candidate gene-based association analysis and traditional bi-parental QTL mapping in conjunction with differential gene expression profiling and gene-based molecular haplotyping/LD (linkage disequilibrium) mapping is a widely recognized strategy for dissecting the complex yield and quality component quantitative traits in different crop plants, including chickpea (Konishi et al., 2006; Sweeney et al., 2007; Tian et al., 2009; Mao et al., 2010; Li et al., 2011; Kharabian-Masouleh et al., 2012; Parida et al., 2012; Kujur et al., 2013, 2014; Negrão et al., 2013; Saxena et al., 2014a; Zuo and Li, 2014; Bajaj et al., 2015). Natural variation and artificial selection in many cloned genes (e.g., red pericarp color-regulating *Rc* gene in rice) underlying the QTLs regulating seed coat color during domestication have established seed color as a common target trait for both domestication and artificial breeding in crop plants (Sweeney et al., 2007; Hossain et al., 2011; Meyer et al., 2012). Henceforth, the above-said integrated strategy can be deployed for identification of functionally relevant novel molecular tags (markers, genes, QTLs, and alleles) controlling seed coat color and unraveling the molecular genetic basis of such natural trait variation in various cultivated and wild accessions of chickpea adapted to diverse agro-climatic regions. The resultant findings would assist us to genetically select *desi* and *kabuli* accessions for diverse desirable seed coat color, driving genomics-assisted breeding and ultimately development of tailored chickpea cultivars with preferred seed color.

In view of the aforementioned prospects, our study integrated GWAS and candidate gene-based association analysis with traditional QTL mapping, differential gene expression profiling, gene haplotype-specific LD mapping and proanthocyanidin (PA) quantitation assay to identify novel potential genomic loci (genes/gene-associated targets) and alleles/haplotypes regulating seed coat color and understand their haplotype-based evolutionary/domestication patterns in cultivated and wild chickpea.

## Materials and methods

### Discovery, genotyping and annotation of genome-wide SNPs

For large-scale discovery and high-throughput genotyping of SNPs at a genome-wide scale, 93 phenotypically and genotypically diverse *desi* (39 accessions) and *kabuli* (54) chickpea accessions (representing diverse eco-geographical regions of 21 countries of the world) were selected (Table S1) from the available chickpea germplasm collections (16991, including 211 minicore germplasm lines, Upadhyaya and Ortiz, 2001; Upadhyaya et al., 2001, 2008). In addition, 79 diverse *Cicer* accessions belonging to five annual wild species, namely *C. reticulatum* (14), *C. echinospermum* (8), *C. judaicum* (22), *C. bijugum* (19), and *C. pinnatifidum* (15) and one perennial species *C. microphyllum* were chosen for haplotype-based evolutionary study (Table S1). The genomic DNA from the young leaf samples of 172 cultivated and wild chickpea accessions were isolated using QIAGEN DNeasy 96 Plant Kit (QIAGEN, USA).

The genomic DNA of accessions was digested with *Ape*KI, ligated to adapters-carrying unique barcodes and 2 × 96-plex GBS libraries were constructed. Each of two 96-plex GBS libraries were pooled together and sequenced (100-bp single end) individually using Illumina two-lane flow cell of HiSeq2000 NGS (next-generation sequencing) platform following Elshire et al. (2011) and Spindel et al. (2013). The unique barcodes attached to each of 172 accessions were used as reference for de-multiplexing of their high-quality sequence reads. The decoded high-quality FASTQ sequences were mapped to reference *kabuli* draft chickpea genome (Varshney et al., 2013) employing Bowtie v2.1.0 (Langmead and Salzberg, 2012). The sequence alignment map files generated from *kabuli* genome were analyzed using the reference-based GBS pipeline/genotyping approach of STACKS v1.0 (http://creskolab.uoregon.edu/stacks) for identifying the high-quality SNPs (SNP base-quality, 20 supported by minimum sequence read-depth, 10) from 172 accessions (as per Kujur et al., 2015). The *kabuli* genome annotation (Varshney et al., 2013) was utilized as an anchor for structural and functional annotation of GBS-based SNPs in different coding (synonymous and non-synonymous SNPs) and non-coding sequence components of genes and genomes (chromosomes/pseudomolecules and scaffolds) of *kabuli* chickpea.

### Mining and genotyping of candidate gene-based SNPs

For large-scale mining and high-throughput genotyping of candidate gene-based SNPs in chickpea, a selected set of 28 cloned genes/QTLs known to be involved in regulation of seed pericarp color in related dicot species, *Arabidopsis thaliana, Medicago*, and soybean (Lepiniec et al., 2006; Zhao and Dixon, 2009; Yang et al., 2010) were acquired. This includes a tandem array of three seed color candidate co-orthologous *tt12*-MATE family transporter repeated genes located at the selective sweep target region of *kabuli* chromosome 4 (reported previously by Varshney et al., 2013). The coding sequences (CDS) of these known/candidate seed color-regulating genes were BLAST searched against the CDS of *kabuli* genes to identify the best possible gene orthologs in chickpea. The CDS (functional domains) and 1000-bp upstream regulatory regions (URRs) of identified true *kabuli* chickpea gene orthologs (*E*-value: 0 and bit score ≥500) were targeted to design (Batch Primer3, http://probes.pw.usda.gov/cgi-bin/batchprimer3/batchprimer3.cgi) the forward and reverse primers with expected amplification product size of 500–700 bp. For mining the gene-based SNPs, primarily 24 *desi, kabuli*, and wild chickpea accessions were selected randomly from 172 accessions (utilized for genome-wide GBS-based SNP genotyping) (Table S1). The gene-based primers were PCR amplified using the genomic DNA of all these 24 accessions and resolved in 1.5% agarose gel. The amplified PCR products were sequenced, the high-quality sequences aligned and SNPs were detected in diverse sequence components of orthologous chickpea genes among accessions following Saxena et al. (2014a). The gene-derived coding and URR-SNPs discovered were further genotyped in the genomic DNA of 172 cultivated and wild chickpea accessions employing Sequenom MALDI-TOF (matrix-assisted laser desorption ionization-time of flight) MassARRAY (http://www.sequenom.com) as per Saxena et al. (2014a). According to allele-specific mass differences between extension products, the genotyping information of different coding and URR-SNPs among accessions were recorded.

### Estimation of polymorphism and diversity statistics

To measure the PIC (polymorphism information content) and genetic diversity coefficient (Nei's genetic distance; Nei et al., 1983), and construct an unrooted neighbor-joining (NJ)-based phylogenetic tree (with 1000 bootstrap replicates) among chickpea accessions, the high-quality SNP genotyping data were analyzed with PowerMarker v3.51 (Liu and Muse, 2005), MEGA v5.0 (Tamura et al., 2011) and TASSEL v5.0 (http://www.maizegenetics.net). A 100-kb non-overlapping sliding window approach of TASSEL v5.0 was implemented to estimate the various nucleotide diversity measures (θπ, θω, and Tajima's D) following Xu et al. (2011) and Varshney et al. (2013).

### Determination of population genetic structure and LD patterns

A model-based program STRUCTURE v2.3.4 was utilized to determine the population genetic structure among chickpea accessions adopting the methods of Kujur et al. (2013, 2014) and Saxena et al. (2014a). The high quality genome-wide and candidate gene-based genotyping data of SNPs physically mapped on eight *kabuli* chromosomes were analyzed using a command line interface of PLINK and the full-matrix approach of TASSELv5.0 (Saxena et al., 2014a; Kumar et al., 2015) to evaluate the genome-wide LD patterns (r^2^, frequency correlation among pair of alleles across a pair of SNP loci) and LD decay (by plotting average r^2^ against 50 and 20 kb uniform physical intervals across chromosomes) in chromosomes and population of chickpea.

### Phenotyping for seed coat color

One hundred seventy-two chickpea accessions were grown in the field [following randomized complete block design (RCBD)] during crop growing season for two consecutive years (2011 and 2012) at two diverse geographical locations (New Delhi; latitude/longitude: 28.4°N/77.1°E and Hyderabad;17.1°N/78.9°E) of India. The seeds of all accessions were phenotyped and classified/characterized into different seed color types by visual estimation of seed coat/pericarp color of mature seeds (stored no longer than 5 months) representing each accession with at least three replications (Upadhyaya and Ortiz, 2001; Upadhyaya et al., 2002, 2006; Hossain et al., 2011). Based on these large-scale replicated multi-location/years field phenotyping, the 172 chickpea accessions were broadly categorized/characterized in accordance with 24 predominant seed coat color types (BL: Black, B: Brown, LB: Light brown, DB: Dark brown, RB: Reddish brown, GB: Grayish brown, SB: Salmon brown, OB: Orange brown, GR: Gray, BB: Brown beige, Y: Yellow, LY: Light yellow, YB: Yellow brown, OY: Orange yellow, O: Orange, YE: Yellow beige, I: Ivory, G: Green, LG: Light green, BR: Brown reddish, M: Variegated, BM: Black brown mosaic, LO: Light orange and BE: Beige) (Figure S1). The frequency distribution and broad-sense heritability (H^2^) of seed coat color were measured using SPSSv17.0 (http://www.spss.com/statistics) as per Kujur et al. (2013, 2014) and Saxena et al. (2014a).

### Trait association mapping

The genome-wide and candidate gene-based SNP genotyping information of 93 cultivated *desi* and *kabuli* accessions was integrated with their seed coat color phenotyping, ancestry coefficient (Q matrix obtained from population structure) and relative kinship matrix (K) data using GLM (Q model)- and MLM (Q+K model)-based approaches of TASSELv5.0 (Kujur et al., 2013, 2014; Kumar et al., 2015). Furthermore, mixed-model (P+K, K, and Q+K) association (EMMA) and P3D/compressed mixed linear model (CMLM) interfaces of GAPIT (Lipka et al., 2012) relying upon principal component analysis (PCA) were employed for GWAS. The relative distribution of observed and expected -log_10_(*P*)-value for each SNP marker-trait association were compared individually utilizing quantile-quantile plot of GAPIT. According to false discovery rate (FDR cut-off ≤ 0.05) (Benjamini and Hochberg, 1995), the adjusted *P*-value threshold of significance was corrected for multiple comparisons. By integrating all four model-based outputs of TASSEL and GAPIT, the potential SNP loci in the target genomic (gene) regions revealing significant association with seed coat color trait variation at highest R^2^ (degree of SNP marker-trait association) and lowest FDR adjusted *P*-values (threshold *P* <1 × 10^−6^) were identified.

### QTL mapping

For validation of seed color-associated genomic loci through QTL mapping, an intra-specific 190 F_7_ RIL mapping population (ICC 12299 × ICC 8261) was developed. The chickpea accessions ICC 12299 (originated from Nepal) and ICC 8261 (originated from Turkey) are LB/YB and BE seed coat colored *desi* and *kabuli* landraces, respectively. A selected set of 384 SNPs showing polymorphism between mapping parental accessions (ICC 12299 and ICC 8261) and 96 previously reported SSR markers (Winter et al., 1999, 2000; Hossain et al., 2011; Thudi et al., 2011) physically/genetically mapped on LGs (linkage groups)/chromosomes (considered as anchors) were genotyped using MALDI-TOF mass array SNP genotyping assay and fluorescent dye-labeled automated fragment analyser (Kujur et al., 2013, 2014; Saxena et al., 2014a,b; Bajaj et al., 2015). The genotyping data of parental polymorphic SNP and SSR markers mapped on LGs (chromosomes) of an intra-specific genetic map and replicated multi-location seed coat color field phenotyping information (following aforesaid phenotyping methods of 172 accessions) of 190 RILmapping individuals and parental accessions were analyzed using the composite interval mapping (CIM) function of MapQTL v6.0 (Van Ooijen, 2009, as per Saxena et al., 2014a). The phenotypic variation explained (PVE) by QTLs (R^2^ %) was measured at significant LOD (logarithm of odds threshold >5.0) to identify and map the major genomic loci underlying robust seed color QTLs in chickpea.

### Differential expression profiling

To infer differential gene regulatory function, the expression profiling of seed color-associated genes validated by both GWAS/candidate gene-based association analysis and QTL mapping was performed. For expression analysis, 11 contrasting chickpea accessions as well as parental accessions and two homozygous individuals of a RIL mapping population (ICC 12299 × ICC 8261) representing two predominant seed coat color types, LB/YB (*desi*: ICCX810800, *desi*: ICC 12299, *desi*: ICCV 10, *desi*: ICC 4958, *desi*: ICC 15061, *kabuli*: ICC 6204, and *kabuli*: Annigeri) and BE (*kabuli*: ICC 20268, *kabuli*: ICC 8261, *kabuli*: Phule G0515, and *desi*: ICC 4926) were selected (Table S1). The vegetative leaf tissues, including seeds without seed coats and seed coats scrapped from the immature (15–25 days after podding/DAP) and mature (26–36 DAP) seeds (30–40 seeds per accession in replicates) of all these accessions and mapping parents/individuals were used for RNA isolation. The isolated RNA was amplified with the gene-specific primers using the semi-quantitative and quantitative RT-PCR assays as per Bajaj et al. (2015). Three independent biological replicates of each sample and two technical replicates of each biological replicate with no template and primer as control were included in the quantitative RT-PCR assay. A house-keeping gene elongation factor 1-alpha (*EF1*α) was utilized as internal control in RT-PCR assay for normalization of expression values across various tissues and developmental stages of accessions and mapping parents/individuals. The significant difference of gene expression in immature and mature seed coats as compared to vegetative leaf tissues and seeds without seed coats (considered as control) of each accessions and mapping parents/individuals was determined and compared among each other to construct a heat map using TIGR MultiExperiment Viewer (MeV, http://www.tm4.org/mev).

### Molecular haplotyping

For gene-based marker haplotyping/LD mapping, the 2 kb URR, exons, introns and 1 kb DRR (downstream regulatory region) of one strong seed color-associated gene (validated by GWAS, QTL mapping, and differential expression profiling) amplified from 172, including 93 cultivated and 79 wild chickpea accessions (Table S1) were cloned and sequenced. For these, the gene amplified PCR products were purified (QIAquick PCR Purification Kit, QIAGEN, USA), ligated with pGEM-T Easy Vector (Promega, USA) and transformed into competent DH5α *E. coli* strain by electroporation (BIO-RAD, USA) to screen blue/white colonies. The plasmids isolated from 20 random positive white clones carrying the desired inserts were sequenced in both forward and reverse directions twice on a capillary-based Automated DNA Sequencer (Applied Biosystems, ABI 3730xl DNA Analyzer, USA) using the BigDye Terminator v3.1 sequencing kit and M13 forward and reverse primers. The trace files were base called separately for all the 10 clones of individual accessions and/primers, checked for quality using *phred* and then assembled into contigs using *phrap*. The high-quality sequences generated for the gene were aligned and compared among 172 chickpea accessions using the CLUSTALW multiple sequence alignment tool in MEGA 6.0 (Tamura et al., 2013) to discover the SNP and SSR allelic variants among these accessions. The SNP-SSR marker-based haplotypes were constituted in a gene to determine the haplotype-based LD patterns among accessions following Kujur et al. (2013, 2014) and Saxena et al. (2014a). To evaluate the trait-association potential and determine the haplotype-based evolutionary pattern of the gene, the marker-based gene haplotype-derived genotyping information was correlated with seed color field phenotyping data of accessions used. To assess the potential of seed color-regulatory haplotypes constituted in a gene, differential expression profiling in the seed coats dissected from mature seeds of aforesaid six contrasting chickpea accessions as well as mapping parents and two homozygous RIL individuals representing LB/YB (ICCX-810800, ICC 12299, and Annigeri)- and BE (ICC 20268, ICC 8261, and ICC 4926)-specific haplotype groups was performed using their respective gene haplotype-based primers (following aforementioned methods).

### Estimation of proanthocyanidin (PA)

The seed coats (~50 mg) (three independent biological replicates per accession) from matured seeds of aforementioned six contrasting chickpea accessions, mapping parents and two homozygous RIL individuals representing LB/YB and BE seed coat color haplotypes were scraped. These seed coat tissues were used for soluble (with 0.2% DMACA) and insoluble (with butanol-HCl) PA extraction and quantification following the methods of Pang et al. (2008) and Liu et al. (2014).

## Results and discussion

### Large-scale discovery, high-throughput genotyping and annotation of genome-wide GBS- and candidate gene-derived SNPs

The sequencing of 2 × 96-plex *Ape*KI GBS libraries generated on an average of 200.7 million high-quality sequence reads that are evenly distributed (mean: 2.1 million reads per accession) across 172, including 93 cultivated and 79 wild chickpea accessions (Table S1). Notably, on an average 87% of these sequence reads were mapped to unique physical positions on *kabuli* reference genome. The sequencing data generated from 93 cultivated (*desi* and *kabuli*) and 79 wild chickpea accessions in this study have been submitted to a freely accessible NCBI-short read archive (SRA) database (www.ncbi.nlm.nih.gov/sra) with accession numbers SRX845396 (http://www.ncbi.nlm.nih.gov/gquery/?term=SRX845396) and SRX971856, respectively. In total, 8673 high-quality SNPs (with read-depth 10, SNP base quality ≥20, <1% missing data and ~2% heterozygosity in each accession) differentiating 172 accessions were discovered using *kabuli* reference genome-based GBS assay (Table S2). This highlights the advantages of GBS assay in rapid large-scale mining and high-throughput genotyping of accurate and high-quality SNPs simultaneously at a genome-wide scale in chickpea. The successful applications of GBS assay particularly in construction of high-density intra- and inter-specific genetic linkage maps, high-resolution GWAS and fine mapping/positional cloning of genes/QTLs governing important agronomic traits have been demonstrated recently in diverse legume crops, including chickpea (Sonah et al., 2013, 2014; Deokar et al., 2014; Jaganathan et al., 2014; Liu et al., 2014). In this context, GBS-based genome-wide SNPs identified by us have potential to be utilized for genomics-assisted breeding applications in chickpea.

A number of diverse known cloned genes governing complex transcriptional regulatory anthocyanin and flavonoid biosynthetic pathways to accumulate specific secondary metabolites (majorly anthocyanin and proanthocyanidin) in the seed coat for its coloration/pigmentation have been identified and characterized in *Arabidopsis*, soybean and *Medicago* (Focks et al., 1999; Debeaujon et al., 2001; Sagasser et al., 2002; Saitoh et al., 2004; Baxter et al., 2005; Xie and Dixon, 2005; Furukawa et al., 2006; Lepiniec et al., 2006; Liu et al., 2006, 2014; Quattrocchio et al., 2006; Sweeney et al., 2006, 2007; Zhao and Dixon, 2009; Erdmann et al., 2010; Yang et al., 2010; Golam Masum Akond et al., 2011; Rahman et al., 2011; Bowerman et al., 2012; Chen et al., 2012, 2014; Huang et al., 2012; Li et al., 2012; Routaboul et al., 2012; Nguyen et al., 2013; Pourcel et al., 2013; Wang et al., 2013; Zhang et al., 2013; Ichino et al., 2014; Pesch et al., 2014; Wroblewski et al., 2014; Xu et al., 2014). Recently, a tandem array of three seed color-regulating candidate co-orthologous *tt12*-MATE family transporter repeated genes of *M. truncatula* primarily affecting proanthocyanidin biosynthesis under strong purifying selection pressure has been identified at the selective sweep target region of *kabuli* chromosome 4 (Varshney et al., 2013). Besides, the detailed genetic regulation of seed color in chickpea is poorly understood vis-a-vis other legumes. Therefore, the seed color-related known cloned and candidate gene orthologs of legumes (*Medicago* and soybean) and *Arabidopsis* were targeted in our study for genetic association mapping of seed color traits in chickpea. To access the potential of aforesaid known/candidate genes for seed color trait association in chickpea, genetic association mapping was performed by high-throughput genotyping of novel SNP allelic variants mined from diverse sequence components of these genes in 93 cultivated and 79 wild accessions. The PCR amplicon-based sequencing of 28 seed color-related known/candidate *kabuli* gene (CDS and 1 kb-URRs) orthologs in 24 selected chickpea accessions and further comparison of their high-quality gene amplicon sequences detected numerous coding and non-coding SNP allelic variants. The subsequent large-scale validation and high-throughput genotyping of these mined SNP alleles in 172 cultivated and wild accessions by MALDI-TOF mass array successfully identified 372 genic SNPs (233 coding non-synonymous/synonymous and 139 URR SNPs) with an average frequency of 2.82 SNPs/kb (varied from 1.58 to 3.65 SNPs/kb) (Table 1).

**Table 1:** **Twenty-eight seed color-related known/candidate gene orthologs of chickpea selected for genetic association mapping**.

**Characterized known cloned genes**				***Kabuli*** **chickpea gene homologs**				
**Crop plants**	**Gene identity**	**Gene accession IDs**	**References**	**Gene identity**	**Chromosomes**	**Sequenced gene amplicon size (bp)**	**Coding and upstream regulatory SNPs discovered**	**Average SNP frequency (SNP/kb)**
Soybean	*AR* (Anthocyanidin reductase)	*Glyma08g06630*	Yang et al., 2010	Ca05336	*Ca*_*Kabuli*_Chr06	3793	12	3.16
Soybean	*ANS* (Leucoanthocyanidin dioxygenase)	*Glyma01g42350*		Ca02395	*Ca*_*Kabuli*_Chr08	2179	6	2.75
Soybean	*DFR1* (Dihydroflavonol 4-reductase 1)	*Glyma14g07940*		Ca10786	*Ca*_*Kabuli*_Chr04	5497	15	2.73
Soybean	*DFR2* (Dihydroflavonol 4-reductase 2)	*Glyma17g37060*		Ca10787	*Ca*_*Kabuli*_Chr04	4719	13	2.75
Soybean	*Myb* TF (Myeloblastoma Transcription Factor)	*Glyma13g09980*		Ca11427	*Ca*_*Kabuli*_Chr05	1816	5	2.75
Soybean	*F3H1* (Flavanone 3-hydroxylase 1)	*Glyma16g23880*		Ca09972	*Ca*_*Kabuli*_Chr07	3822	13	3.40
Soybean	*F3H2* (Flavanone 3-hydroxylase 2)	*Glyma02g05450*		Ca09973	*Ca*_*Kabuli*_Chr07	3872	10	2.58
*Arabidopsis*	*tt3*-DFR (Dihydroflavonol 4-reductase)	*AT5G42800*	Wang et al., 2013	Ca10786	*Ca*_*Kabuli*_Chr04	5497	20	3.64
*Arabidopsis*	*tt4*-CHS (Chalcone Synthase)	*AT5G13930*	Wroblewski et al., 2014	Ca08294	*Ca*_*Kabuli*_Chr03	3010	11	3.65
*Arabidopsis*	*tt5*-CHI (Chalcone isomerase)	*AT3G55120*	Pourcel et al., 2013	Ca03250	*Ca*_*Kabuli*_Chr07	11604	37	3.19
*Arabidopsis*	*tt7*-F3‘H (Flavonoid 3′-hydroxylase)	*AT5G07990*	Routaboul et al., 2012	Ca13196	*Ca*_*Kabuli*_Chr07	1443	4	2.77
*Arabidopsis*	*tt10*-PPO (Polyphenol oxidase)	*AT5G48100*	Zhang et al., 2012	Ca23340	*Ca*_*Kabuli*_Chr03	3106	10	3.22
*Arabidopsis*	*tt12*-MATE secondary transporter (Multi antimicrobial extrusion)	*AT3G59030*	Debeaujon et al., 2001	Ca10158	*Ca*_*Kabuli*_Chr02	3676	9	2.45
*Arabidopsis*	*tt15*-GT (Glucosyl transferase)	*AT1G43620*	Focks et al., 1999	Ca04376	*Ca*_*Kabuli*_Chr04	6919	21	3.04
*Arabidopsis*	*tt18*-LDOX (Leucoanthocyanidin dioxygenase)	*AT4G22880*	Liu et al., 2014	Ca02395	*Ca*_*Kabuli*_Chr08	2179	4	1.84
*Arabidopsis*	*tt19*-GST (Glutathione S-transferase)	*AT5G17220*	Xu et al., 2014	Ca03442	*Ca*_*Kabuli*_Chr04	1903	3	1.58
*Arabidopsis*	*ban*-*ANR* (Anthocyanin reductase)	*AT1G61720*	Bowerman et al., 2012	Ca05336	*Ca*_*Kabuli*_Chr06	3793	9	2.37
*Arabidopsis*	*aha10* (Autoinhibited H^+^ ATPase isoform 10)	*AT1G17260*	Baxter et al., 2005	Ca01370	*Ca*_*Kabuli*_Chr03	10262	35	3.41
*Arabidopsis*	*tt1*-TF WIP (Wiskott-aldrich syndrome protein) type zinc finger	*AT1G34790*	Sagasser et al., 2002	Ca27078	*Ca*_*Kabuli*_Scaffold-2269	2324	5	2.15
*Arabidopsis*	*tt2*-TF AtMYB123 (Myeloblastoma transcription factor)	*AT5G35550*	Chen et al., 2012	Ca12762	*Ca*_*Kabuli*_Chr07	2226	5	2.25
*Arabidopsis*	*tt8*-TF AtbHLH042 (Basic helix-loop-helix)	*AT4G09820*	Chen et al., 2014	Ca14089	*Ca*_*Kabuli*_Chr01	2283	6	2.63
*Arabidopsis*	*tt16*-TF MADS AtAGL32 (Agamous like-32)	*AT5G23260*	Erdmann et al., 2010	Ca15760	*Ca*_*Kabuli*_Chr07	3027	8	2.64
*Arabidopsis*	*ttg1*-WD40	*AT5G24520*	Nguyen et al., 2013	Ca04058	*Ca*_*Kabuli*_Chr05	2028	4	1.97
*Arabidopsis*	*ttg2*-TF WRKY	*AT2G37260*	Pesch et al., 2014	Ca17273	*Ca*_*Kabuli*_Chr02	4380	16	3.65
*Arabidopsis*	*tt9*-Unknown	*AT3G28430*	Ichino et al., 2014	Ca22116	*Ca*_*Kabuli*_Chr01	16647	47	2.82
*Medicago*	MATE secondary transporter (TRANSPARENT TESTA)	*MTR1g108990*	Zhao and Dixon, 2009	Ca05556^a^	*Ca*_*Kabuli*_Chr04	3936	10	2.54
*Medicago*	MATE secondary transporter (TRANSPARENT TESTA)	*MTR1g108840*		Ca05557^a^	*Ca*_*Kabuli*_Chr04	5315	19	3.57
*Medicago*	MATE secondary transporter (TRANSPARENT TESTA)	*MTR1g108810*		Ca05558^a^	*Ca*_*Kabuli*_Chr04	4540	15	3.30

a *Tandem array of three seed color candidate co-orthologous tt12-MATE family transporter repeated genes located at the selective sweep target region of kabuli chromosome 4 reported by Varshney et al. (2013)*.

**Figure 1:**
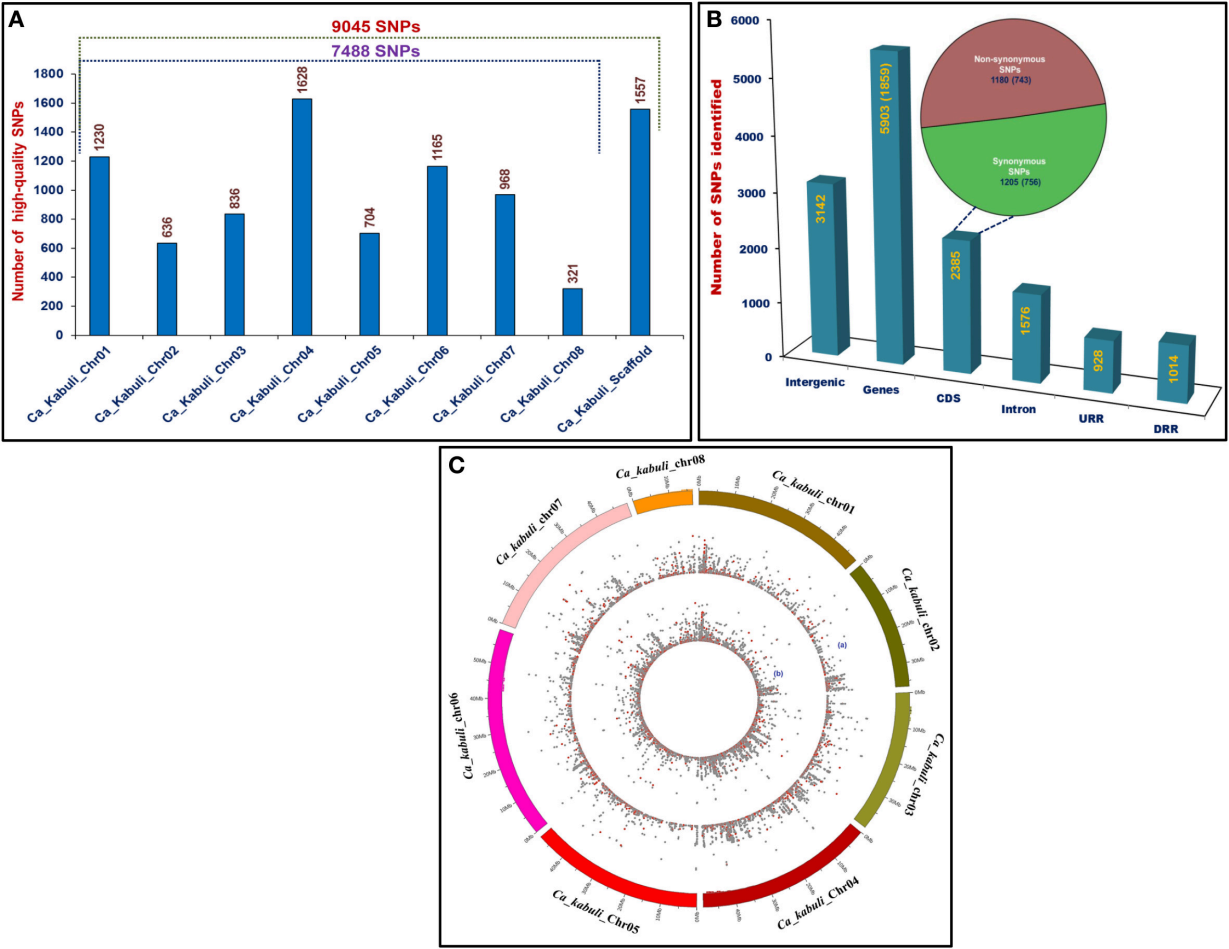
**(A)** Frequency distribution of 9045 SNPs discovered employing reference *kabuli* genome (eight chromosomes and scaffolds)-based GBS and candidate gene-derived SNP genotyping assays. **(B)** Structural annotation of SNPs in the diverse coding (synonymous and non-synonymous) and non-coding (intron, URR, and DRR) sequence components of genes and intergenic regions of *kabuli* genome. The gene annotation information of *kabuli* genome (Varshney et al., 2013) was considered as reference to infer CDS (coding sequences)/exons, URR (upstream regulatory region) and DRR (downstream regulatory region) sequence components of genes. **(C)** A Circos circular ideogram depicting the relative distribution of 9045 SNPs, including non-synonymous SNPs (marked with red dots) physically mapped on eight *kabuli* chromosomes. The outermost circles illustrate the eight *kabuli* chromosomes coded with different colors, while the two inner circles “a” and “b” represent the distribution of SNPs mined from 93 cultivated (*desi* and *kabuli*) and 79 wild chickpea accessions, respectively.

The genome-wide GBS- and candidate gene-based SNP genotyping overall identified 9045 SNPs showing polymorphism among 172 cultivated and wild chickpea accessions. The 7488 and 1557 SNPs of these, were physically mapped on eight chromosomes and scaffolds of *kabuli* genome, respectively (Figure 1A). A maximum number of 1628 SNPs (21.7%) were mapped on *kabuli* chromosome 4, whereas minimum of 321 SNPs (4.3%) were mapped on chromosome 8. The structural annotation of 9045 SNPs identified 5903 (65.3%) and 3142 (34.7%) SNPs in the 1859 genes and intergenic regions of *kabuli* genome (Figure 1B). The abundance of gene-based SNPs was observed in the CDS (2385 SNPs, 40.4%), followed by introns (1576, 26.7%) and least (928, 15.7%) in the URRs. In total, 1205 (50.5%) and 1180 (49.5%) coding SNPs in the 756 and 743 genes exhibited synonymous and non-synonymous substitutions, respectively (Figure 1B). The relative distribution of GBS-based SNPs, including non-synonymous SNPs physically mapped on eight *kabuli* chromosomes showing polymorphism among 93 cultivated (*desi* and *kabuli*) and 79 wild accessions are depicted individually in a Circos circular ideogram (Figure 1C). The functional annotation of 1859 SNP-carrying genes exhibited their maximum correspondence to growth, development and metabolism-related proteins (43%), followed by transcription factors (22%) and signal transduction proteins (11%). The structurally and functionally annotated genome-wide and gene-derived SNPs physically mapped on *kabuli* chromosomes and novel SNP allelic variants mined from diverse seed color-regulating known/cloned and candidate genes can be utilized for various large-scale genotyping applications particularly in complex seed color quantitative trait dissection of chickpea.

### Polymorphism and molecular diversity potential of SNPs

The identified 9045 genome-wide GBS- and candidate gene-based SNPs revealed polymorphism among 172 cultivated and wild chickpea accessions with a higher PIC (0.13–0.41 with a mean 0.32) and nucleotide diversity (θπ: 2.14, θω: 2.19, and Tajima's D: -1.59) potential (Table S3). The intra-specific polymorphic and nucleotide diversity potential detected by SNPs within wild and cultivated (*desi* and *kabuli*) chickpea species was lower as compared to that of inter-specific polymorphism and nucleotide diversity among species (Table S3).

The molecular diversity potential detected by 9045 SNPs among 172 *desi, kabuli* and wild chickpea accessions exhibited a broader range of genetic distances varying from 0.15 to 0.83 with a mean of 0.52. A lower genetic diversity level (mean genetic distance: 0.32) was observed among 93 *desi* and *kabuli* chickpea accessions than that of 79 wild chickpea accessions (0.47). The unrooted neighbor-joining phylogenetic tree construction using 9045 genome-wide and candidate gene-based SNPs depicted a distinct differentiation among 172 cultivated and wild chickpea accessions and further clustered these accessions into two major groups (cultivated and wild) as expected (Figure S2). A relatively higher intra- and inter-specific polymorphism and nucleotide diversity potential as well as broader allelic (functional) diversity detected by 9045 SNPs among 172 cultivated (*desi* and *kabuli*) and wild chickpea accessions is much higher compared to that of earlier similar documentation (Nayak et al., 2010; Gujaria et al., 2011; Roorkiwal et al., 2013; Varshney et al., 2013). This suggests the utility of our developed genome-wide and gene-based SNP markers for faster screening of preferable diverse accessions/inter-specific hybrids in cross (introgression)-breeding program for chickpea varietal improvement.

### Association analysis for seed coat color trait

**Figure 2:**
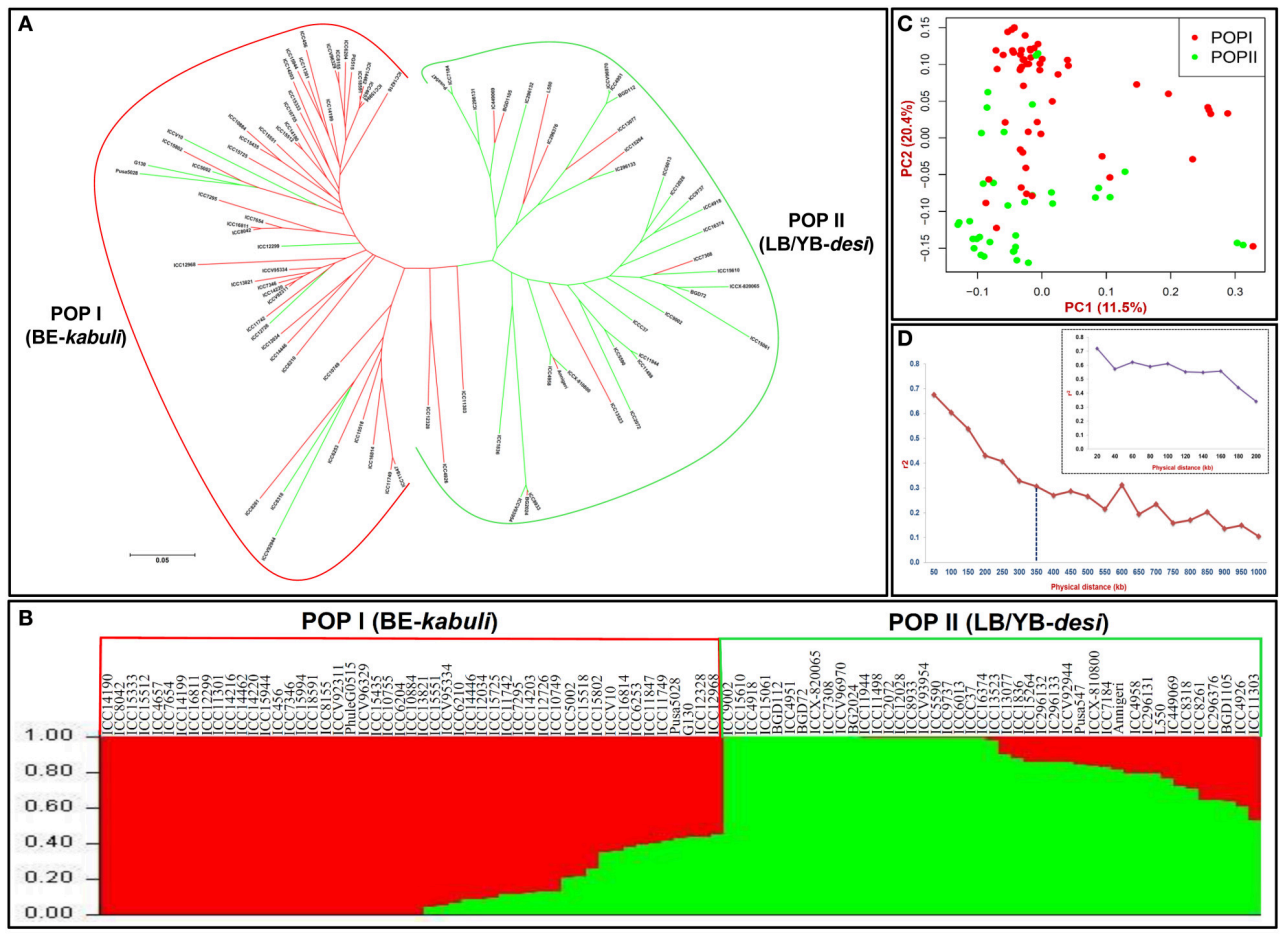
**(A)** Unrooted phylogenetic tree (Nei's genetic distance), **(B)** Population genetic structure (optimal population number *K* = 2 with two diverse color) and **(C)** Principal component analysis (PCA) using 8837 genome-wide GBS- and candidate gene-based SNPs assigned 93 BE (beige) and LB/YB (light/yellow brown) seed coat color representing *kabuli* and *desi* chickpea accessions mostly into two major populations-POP I and POP II, respectively. In population structure, the accessions represented by vertical bars along the horizontal axis were classified into K color segments based on their estimated membership fraction in each K cluster. In PCA, the PC1 and PC2 explained 7.1 and 17.4% of the total variance, respectively. **(D)** LD decay (mean r^2^) measured in a population of 93 cultivated *desi* and *kabuli* chickpea accessions using 7488 SNPs physically mapped on eight *kabuli* chromosomes. The plotted curved line denotes the mean *r*^2^-values among SNP loci spaced with uniform 50 kb physical intervals from 0 to 1000 kb across chromosomes. The plotted line in uppermost indicates the mean *r*^2^-values among SNPs spaced with uniform 20 kb physical intervals from 0 to 200 kb on chromosomes.

For GWAS and candidate gene-based association mapping, 8837 SNPs showing polymorphism among 93 *desi* and *kabuli* chickpea accessions based on genome-wide GBS- and candidate gene-based SNP genotyping were utilized. The neighbor-joining phylogenetic tree, high-resolution population genetic structure and PCA differentiated all 93 chickpea accessions from each other and clustered into two distinct populations; POP I and POP II representing mostly the *kabuli* and *desi* chickpea accessions with BE and LB/YB seed coat color, respectively (Figures 2A–C). The implication of chromosomal LD patterns, including LD decay in GWAS to identify potential genomic loci associated with diverse agronomic traits have been well demonstrated in many crop plants, including chickpea (Zhao et al., 2011; Thudi et al., 2014). The determination of LD patterns in a population of 93 accessions using 7488 SNPs (physically mapped across eight *kabuli* chromosomes) demonstrated a higher LD estimate (r^2^: 0.68) and extended LD decay (r^2^ decreased half of its maximum value) approximately at 350–400 kb physical distance in *kabuli* chromosomes (Figure 2D). This extensive LD estimates and extended LD decay in the domesticated self-pollinating crop plant like chickpea in contrast to other domesticated self-pollinated crops like rice and soybean (Hyten et al., 2007; Mather et al., 2007; McNally et al., 2009; Huang et al., 2010; Lam et al., 2010; Zhao et al., 2011) is quite expected. This could be due to extensive contribution of four sequential bottlenecks during the domestication of chickpea (Lev-Yadun et al., 2000; Abbo et al., 2003; Berger et al., 2005; Burger et al., 2008; Toker, 2009; Jain et al., 2013; Kujur et al., 2013; Varshney et al., 2013; Saxena et al., 2014b) leading to reduction of genetic diversity in cultivated chickpea than that of other domesticated selfing plant species. The longer LD in chickpea chromosomes requires fewer markers to saturate the genome resulting in low resolution and thus indicating low significant association between the genetic markers and genes controlling the phenotypes in chickpea. In the present context due to insufficient and non-uniform marker coverage on a larger genome of chickpea with low intra-specific polymorphism, the GWAS integrated with candidate gene-based association analysis will be of much relevance in efficient quantitative dissection of complex traits in chickpea (Neale and Savolainen, 2004). This strategy of trait association mapping has been successfully implemented in a domesticated as well as larger genome self-pollinated crop species like barley, where extended LD decay was observed at genome level (Haseneyer et al., 2010; Varshney et al., 2012). Henceforth, the combinatorial approach of GWAS and candidate gene-based association mapping would strengthen the possibility of identifying numerous high-resolution trait-associated valid genomic loci at a genome-wide scale in chickpea.

**Figure 3:**
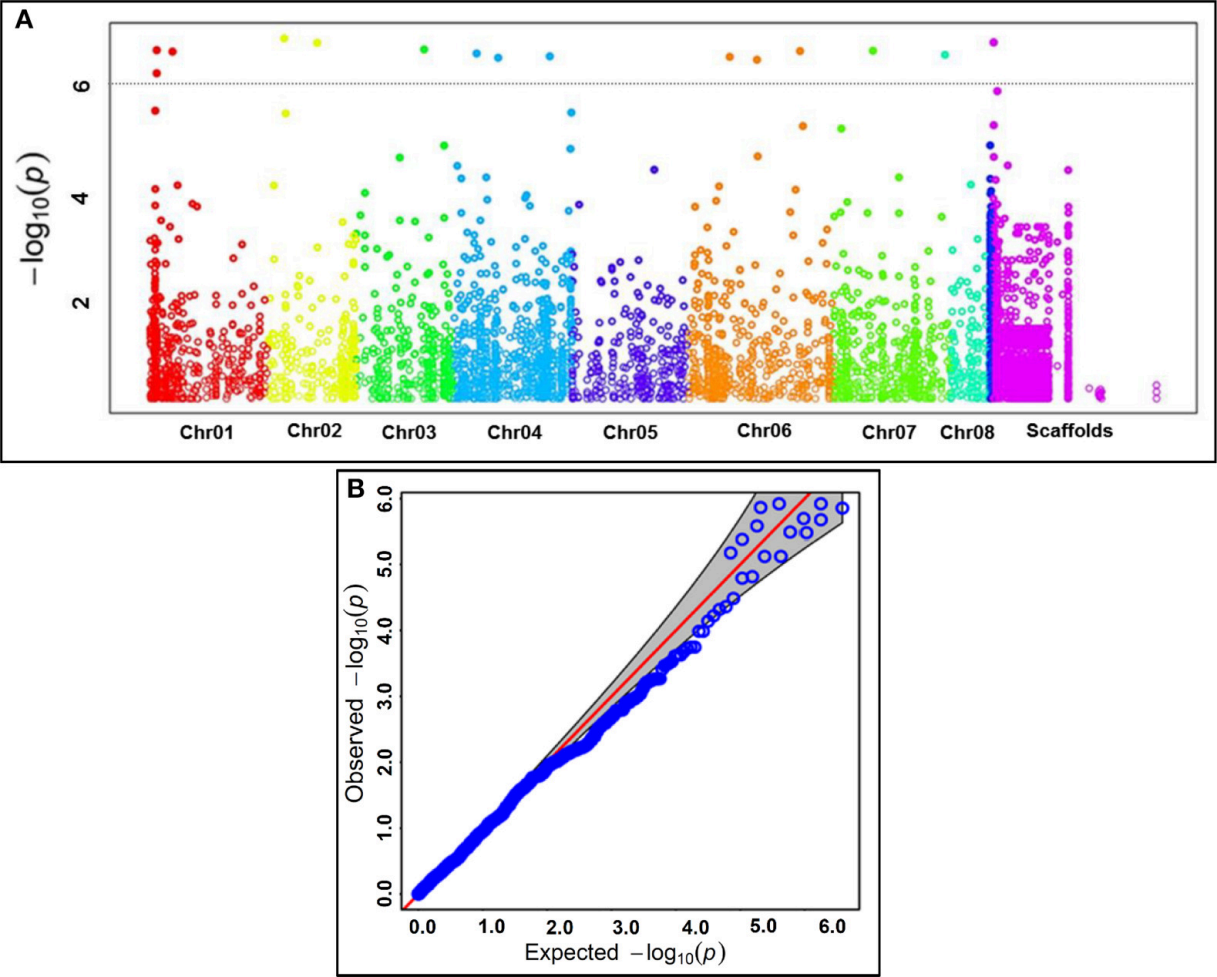
**(A)** GWAS-derived Manhattan plot showing significant *P*-values (measured integrating GLM, MLM, EMMA, and CMLM) associated with seed coat color trait using 9045 genome-wide GBS- and candidate gene-based SNPs. The x-axis represents the relative density of reference genome-based SNPs physically mapped on eight chromosomes and scaffolds of *kabuli* genome. The y-axis indicates the −log_10_(*P*)-value for significant association of SNPs with seed color trait. The SNPs showing significant association with seed color at cut-off *P* ≤ 1 × 10^−6^ are marked with dotted lines. **(B)** Quantile-quantile plots illustrating the comparison between expected and observed −log_10_(*P*)-values with FDR cut-off < 0.05 to detect significant genomic loci (genes) associated with seed color trait in chickpea.

Keeping that in view, the GWAS and candidate gene-based association analysis was performed by integrating 8837 SNP genotyping data of 93 accessions with their replicated multi-location/years seed coat color field phenotyping information. The normal frequency distribution along with broader seed color phenotypic variation (Figure S3A) and 75% broad-sense heritability (H^2^) among 93 accessions belonging to a population was evident. The accessions exclusively exhibiting consistent phenotypic expression of seed coat color across two geographical locations/years (supported by high H^2^) were utilized for subsequent SNP marker-trait association. The integration of outcomes from four model-based approaches [TASSEL (GLM and MLM) and GAPIT (EMMA and CMLM)] with a quantile-quantile plot of the expected and observed -log_10_ *P*-values at FDR cut-off ≤0.05 in GWAS and candidate gene-based association analysis identified 15 genomic loci showing significant association with seed coat color at a *P* ≤ 10^−6^ (Figures 3A,B, Table 2). This includes eight genome-wide GBS-based and seven candidate gene-derived SNPs. Fourteen seed color-associated genomic loci were physically mapped on seven *kabuli* chromosomes. The rest one SNP locus was represented from scaffold region of *kabuli* genome. One of 15 seed color-associated genomic SNP loci was derived from the intergenic region, whereas rest 14 SNP loci were represented from diverse coding (seven, including six non-synonymous SNPs) as well as non-coding intronic (two SNPs) and URR (five) sequence components of 13 *kabuli* genes (Table 2).

**Table 2:** **Fifteen seed coat color-regulating genomic loci (genes) identified by GWAS and candidate gene-based association mapping**.

SNP IDs^*^^a^	***Kabuli* chromosomes/scaffolds**	**SNPs physical positions (bp)**	**SNPs (Reference/alternate alleles)**	**Genes/gene-associated targets^b^**	**Structural annotation**	**Association analysis**	
						***P***	***R*^2^ (%)**
CWSNP151	1	1858509	(C/T)	Ca00233 (Inositol monophosphate)	CDS (Syn)	2.2 × 10^−7^	25
CWSNP156	1	1859914	(G/A)	Ca00233 (Inositol monophosphate)	Intron	2.0 × 10^−7^	27
CWSNP208	1	2053788	(A/G)	Ca00260 (Disease resistance protein)	CDS (NSyn) Threonine (ACG)-Alanine (GCG)	1.8 × 10^−7^	22
CWSNP1275	2	7701199	(T/G)	Ca18123 (*tt12*-MATE secondary transporter)	CDS (NSyn) Valine (GTG)-Glycine (GGG)	1.7 × 10^−9^	43
gSNP1	2	33782378	(C/A)	Ca10158 (*tt12*-MATE secondary transporter)	CDS (NSyn) Alanine (GCT)-Aspartic acid (GAT)	1.2 × 10^−7^	20
gSNP2	3	26031490	(A/G)	Ca08294 (*tt4*-Chalcone synthase)	URR	1.5 × 10^−7^	33
gSNP5	4	48021943	(G/T)	Ca10786 (*tt3*-Dihydroflavonol-4-reductase)	CDS (NSyn) Glycine (GGG)-Valine (GTG)	1.7 × 10^−7^	29
gSNP3	4	7931791	(T/C)	Ca03442 (*tt19*-Glutathione-S-reductase)	URR	1.4 × 10^−7^	30
gSNP4	4	15386163	(T/G)	Ca05557 (*tt12*-MATE secondary transporter)	CDS (NSyn) Serine (TCA)-Alanine (GCA)	1.3 × 10^−6^	24
gSNP6	6	15514534	(C/G)	Ca05336 (ban-ANR-Anthocyanin reductase)	URR	2.1 × 10^−8^	35
CWSNP5434	6	28325392	(C/T)	Ca25968 (Ribonuclease T2)	CDS (NSyn) Serine (TCA)-Leucine (TTA)	2.4 × 10^−7^	27
CWSNP5762	6	56793021	(G/T)	–	Intergenic	1.5 × 10^−7^	21
CWSNP6261	7	14281993	(G/T)	Ca23042 (domain of unknown function DUF231)	Intron	2.1 × 10^−7^	38
gSNP7	8	2062481	(G/A)	Ca02395 (*tt18*-Leucoanthocyanidin dioxygenase)	URR	2.1 × 10^−6^	26
CWSNP8591	Scaffold93	645442	(A/C)	Ca23085 (Integrase)	URR	1.9 × 10^−7^	23

* *CWSNP (cultivated wild SNP) and gSNP (gene-derived SNPs)*.

*CDS, coding sequence; NSyn, non-synonymous; Syn, synonymous; URR, upstream regulatory region*.

a *Details regarding SNPs are mentioned in the Table S2*.

*gSNP: gene-derived SNPs associated with seed coat color trait identified by candidate gene-based association mapping*.

The identified 15 significant SNPs explained (R^2^) an average of 28% (ranging 20–43%) seed coat color phenotypic variation in a population of 93 chickpea accessions. The total phenotypic variation for seed coat color explained by the 15 SNPs was 61%. The significant association of 15 multiple SNP loci in more than one genomic region (genes) with seed coat color gave clues for complex genetic inheritance pattern/regulation of this target quantitative trait in chickpea. This association was evident from high chromosomal and population-specific LD estimates with preferable LD decay of trait-associated linked/unlinked multiple SNP loci and further by the observed genetic heterogeneity of seed color traits across two populations. We observed no significant differences concerning the association potential of SNP loci with seed color in different populations despite diverse genetic architecture of seed coat color traits in two populations and along entire population. Therefore, seed coat color-associated genomic loci (genes) identified by us employing both GWAS and candidate gene-based association mapping could have potential for establishing rapid marker-trait linkages and identifying genes/QTLs regulating seed color in chickpea. Seven (three non-synonymous coding and four URR SNPs) SNP loci in the six different known cloned and candidate seed color genes (*tt12, tt4, tt3, tt19, ban-ANR*, and *tt18*) had significant association (P: 1.3 × 10^−6^ to 1.5 × 10^−8^ with R^2^: 20–35%) with seed coat color trait in chickpea (Table 2). Interestingly, significant association of non-synonymous and regulatory SNPs identified in the different dormancy and stress-related known/candidate genes (encoding inositol monophosphate and disease resistance proteins) with seed coat color was observed in chickpea. This is consistent with previous studies that reported strong correlation/interactions among genes/QTLs regulating all three important agronomic traits, including seed coat pigmentation, seed dormancy/germination ability and stress tolerance in legumes (Harborne and Williams, 2000; Furukawa et al., 2006; Lepiniec et al., 2006; Kovinich et al., 2012; Saxena et al., 2013; Smýkal et al., 2014). The transcriptional regulation of numerous genes involved in anthocyanin and flavonoid biosynthetic pathways lead to accumulation of diverse major secondary metabolites in specific plant organs, which are known to play most important role in collective alteration of seed coat pigmentation/coloration, dormancy and defense (stress) response in crop plants (Gore et al., 2002; Benitez et al., 2004; Senda et al., 2004; Isemura et al., 2007; Caldas and Blair, 2009; Diaz et al., 2010; Kongjaimun et al., 2012). Notably, one non-synonymous SNP locus (T/G) in the CDS of the MATE (multidrug and toxic compound extrusion) secondary transporter gene (Ca18123, mapped on *kabuli* chromosome 2) exhibiting strong association (P: 1.7 × 10^−9^ with R^2^: 43%) with seed color than that of other 14 seed color-associated SNPs was selected as a potential candidate for further analysis. The discovery of non-synonymous and regulatory SNP loci particularly in coding and URRs of known/candidate genes associated with seed color traits signifies their functional relevance in rapid seed color trait-regulatory gene identification and characterization in chickpea. The non-synonymous (amino acid substitutions) and regulatory SNPs in the genes have known to alter their transcriptional regulatory mechanism for controlling diverse traits of agronomic importance, including seed/grain size and weight in rice (Fan et al., 2006; Mao et al., 2010; Li et al., 2011; Zhang et al., 2012) and chickpea (Kujur et al., 2013, 2014; Bajaj et al., 2015; Saxena et al., 2014b).

Two candidate seed color-associated MATE secondary transporter genes [including one (Ca05557) reported earlier at the selective sweep region of *kabuli* chromosome 4, (Varshney et al., 2013)] identified by use of both candidate gene-based association mapping and GWAS were selected (Table S4) to compare and deduce the nucleotide diversity measures as well as the direction and magnitude of natural selection acting between these genes. These genes revealed a similar trend of nucleotide diversity (θπ and θω) and Tajima's D-based selection pressure occurring on LB/YB and BE seed colored *desi* and *kabuli* chickpea accessions, respectively (Table S4). Interestingly, the degree of nucleotide diversity was reduced by 61% in the LB/YB seed colored cultivated *desi* accessions than that of *kabuli* chickpea accessions with BE seed color. The extreme reduction of nucleotide diversity level (θπ: 0.73–0.79 and θω: 0.77–0.82) and strong purifying selection along with reduced Tajima's D (−2.89 to −2.63) specifically in LB/YB seed colored cultivated *desi* chickpea accessions compared with BE seed colored *kabuli* accessions (θπ: 1.93–1.96, θω: 1.95–1.98 and D: 1.82–1.90) was evident (Table S4). These findings provide definite evidence regarding signature of strong purifying selection in LB/YB seed colored *desi* accessions in contrast to BE seed colored *kabuli* chickpea accessions, which is in line with enduring purifying selection for seed coat/pericarp color traits in crop plants, including chickpea (Sweeney et al., 2007; Varshney et al., 2013). Therefore, seed color in chickpea vis-à-vis other crop plants can be considered as a target trait for both domestication and artificial breeding (Sweeney et al., 2007; Hossain et al., 2011; Meyer et al., 2012).

### Validation of seed color-associated genomic loci through QTL mapping

We constructed a high-density intra-specific genetic linkage map (ICC 12299 × ICC 8261) by integrating 415, including 382 SNP and 33 previously reported parental polymorphic SSR markers across eight chickpea LGs (LG1 to LG8). This genetic map covered a total map length of 1065.7 cM, with a mean inter-marker distance of 2.57 cM (Table S5). The most and least saturated genetic map was LG1 (average inter-marker distance: 1.92 cM) and LG5 (4.0 cM), respectively. The mean map density (average inter-marker distance: 2.57 cM) observed in our constructed intra-specific genetic linkage map was higher/comparable with earlier documentation (Cho et al., 2002, 2004; Cobos et al., 2005; Radhika et al., 2007; Kottapalli et al., 2009; Gaur et al., 2011; Kujur et al., 2013, 2014; Sabbavarapu et al., 2013; Stephens et al., 2014; Varshney et al., 2014). Therefore, this constructed high-density intra-specific genetic linkage map have potential to be utilized as a reference for rapid targeted mapping of potential genes/QTLs governing diverse agronomic traits, including seed coat color in chickpea. The bi-directional transgressive segregation-based normal frequency distribution (Figure S3B), including a significant variation in seed coat color trait along with 78% H^2^ in this developed mapping population (ICC 12299 × ICC 8261) was observed. Two principal seed coat color (BE and LB/YB) groupings; primary (PC1) and secondary (PC2) components accounted for 52 and 48% of the total seed coat color variance, respectively in 190 RIL mapping individuals and parental accessions were evident (Figure S3C). This continuous color distribution inferred the quantitative genetic inheritance pattern of seed coat color trait in the mapping population and thus the developed bi-parental mapping population could be utilized for complex seed color trait dissection through QTL mapping in chickpea.

The QTL mapping was performed by correlating the genotyping data of 415 mapped SNP and SSR markers with multilocation/years seed coat color field phenotyping data of the RIL mapping individuals and parental accessions. This analysis identified five major (LOD: 5.6–11.5) genomic regions harboring robust QTLs (validated across two locations/years) controlling seed coat color traits, which were mapped on five *kabuli* chromosomes (Table S6). The phenotypic variation explained (PVE) for seed color by individual QTL (R^2^) varied from 20.1 to 38.7%. The PVE estimated for all five major QTLs was 39.4%. All these QTLs revealed additive gene effects (ranging −3.9 to −2.5), inferring the effective contribution of ICC 8261 alleles at these loci for BE seed coat color trait. The five SNPs in the genes showing tight linkage with all five major seed color-associated robust QTLs (*CaqSC2.1, CaqSC4.1, CaqSC4.2, CaqSC6.1*, and *CaqSC7.1*) had high seed color trait association potential based on our GWAS and candidate gene-based association analysis (Table 2; Table S6). Remarkably, strong association potential of one non-synonymous SNP (T/G) identified in the CDS of the MATE secondary transporter gene as compared to other genomic loci associated with seed coat color trait was ascertained both by QTL mapping (R^2^: 35.6–38.7%) and genetic association analysis (R^2^: 43%) (Table 2). Therefore, we selected MATE secondary transporter gene as a target candidate for seed coat color trait regulation by its further validation through differential expression profiling and molecular haplotyping in chickpea.

To determine the potential and novelty of seed coat color-regulating 15 genomic loci and five major QTLs detected by association and QTL mapping, respectively, the markers linked/flanking the seed color-associated known QTLs/genes (reported earlier in QTL mapping studies, Hossain et al., 2011) were selected for their validation in seed color-specific 93 diverse *desi* and *kabuli* chickpea accessions and mapping population under study. These comparative analyses revealed correspondence of one known QTL (*CaqSC2.1*) regulating seed coat color between past (SSR marker interval: TA194-TR19) and our present studies based on congruent flanking [CWSNP 1273 (25.2 cM)-CWSNP1277 (30.2 cM)]/linked marker (CWSNP 1275) physical/genetic positions on chromosome (LG) 2. Henceforth, 14 genomic loci and 4 QTLs regulating seed color identified by us employing high-resolution GWAS/candidate gene-based association and QTL mapping are novel and exhibited population-specific genetic inheritance pattern for seed color trait regulation in chickpea. Further validation of these functionally relevant molecular tags is required in diverse genetic backgrounds and/or through fine mapping/map-based positional cloning prior to their utilization in marker-assisted genetic improvement of chickpea for desirable seed color.

### Validation of seed color-associated genes through differential expression profiling

To determine the regulatory pattern of genes for seed coat color, thirteen, including five seed color-associated genes validated both by association analysis and QTL mapping were utilized for differential expression profiling. The RNA isolated from vegetative leaf tissues, seeds without seed coats and seed coats scrapped from the immature and mature seeds of 11 contrasting chickpea accessions as well as mapping parents and two homozygous RIL individuals representing two predominant seed coat color types, LB/YB (*desi*: ICCX810800, *desi*: ICC 12299, *desi*: ICCV10, *desi*: ICC 4958, *desi*: ICC 15061, *kabuli*: ICC 6204, and *kabuli*: Annigeri) and BE (*kabuli*: ICC 20268, *kabuli*: ICC 8261, *kabuli*: Phule G0515, and *desi*: ICC 4926) was amplified with these gene-specific primers using semi-quantitative and quantitative RT-PCR assays (Table S7). Five genes, including four seed color known cloned genes (*tt4*-chalcone synthase, *tt3*-dihydroflavonol-4-reductase, *ban-ANR*-anthocyanin reductase, and *tt19*-glutathione-s-reductase) showed seed coat-specific differential (>2.5-fold up- and/down-regulation, *P* ≤ 0.01) expression [compared with leaves and seeds (without seed coats)] specifically in two seed developmental stages of LB/YB and BE seed colored *desi* and *kabuli* accessions (Figure S4). This suggests that differential regulation and accumulation of transcripts encoded by these four known genes might be essential in synthesis of proanthocyanidin and anthocyanin biosynthetic enzymes in chickpea seed coats for its coloration/pigmentation (Lepiniec et al., 2006; Zhao and Dixon, 2009; Zhao et al., 2010).

Remarkably, a seed coat-specific MATE secondary transporter gene (validated by both association and QTL mapping) showing strong association with seed coat color trait exhibited its higher differential down-regulation (~5–7-folds, *P* ≤ 0.001) in the mature/immature seed coats of BE seed colored chickpea accessions (ICC 20268, ICC 8261, Phule G0515, and ICC 4926) as compared to that of mature/immature seed coats of LB/YB seed colored accessions (ICCX810800, ICC 12299, ICCV10, ICC 4958, ICC 15061, ICC 6204, and Annigeri) (Figure S4). A pronounced down regulation (~13-folds, *P* ≤ 0.001) of this seed coat-specific gene in the mature seed coats of all four BE seed colored chickpea accessions in contrast to immature seed coats of all seven LB/YB seed colored accessions was observed. However, differential down-regulation (~3-folds, *P* ≤ 0.001) of MATE gene in the mature seed coats than the immature seed coats of the accessions within an individual LB/YB and BE seed coat colored group was evident (Figure S4). The reduction of chickpea MATE gene transcript level specifically in the mature seed coat parallels with differential expression pattern that observed previously using *TT12* and *MATE1* genes of *Arabidopsis* and *Medicago*, respectively (Marinova et al., 2007; Pang et al., 2008; Zhao and Dixon, 2009; Zhao et al., 2010). These outcomes over imply the functional significance of MATE gene in possible transcriptional regulation of seed color trait in chickpea.

### Molecular haplotyping of a strong seed color-regulating gene

**Figure 4:**
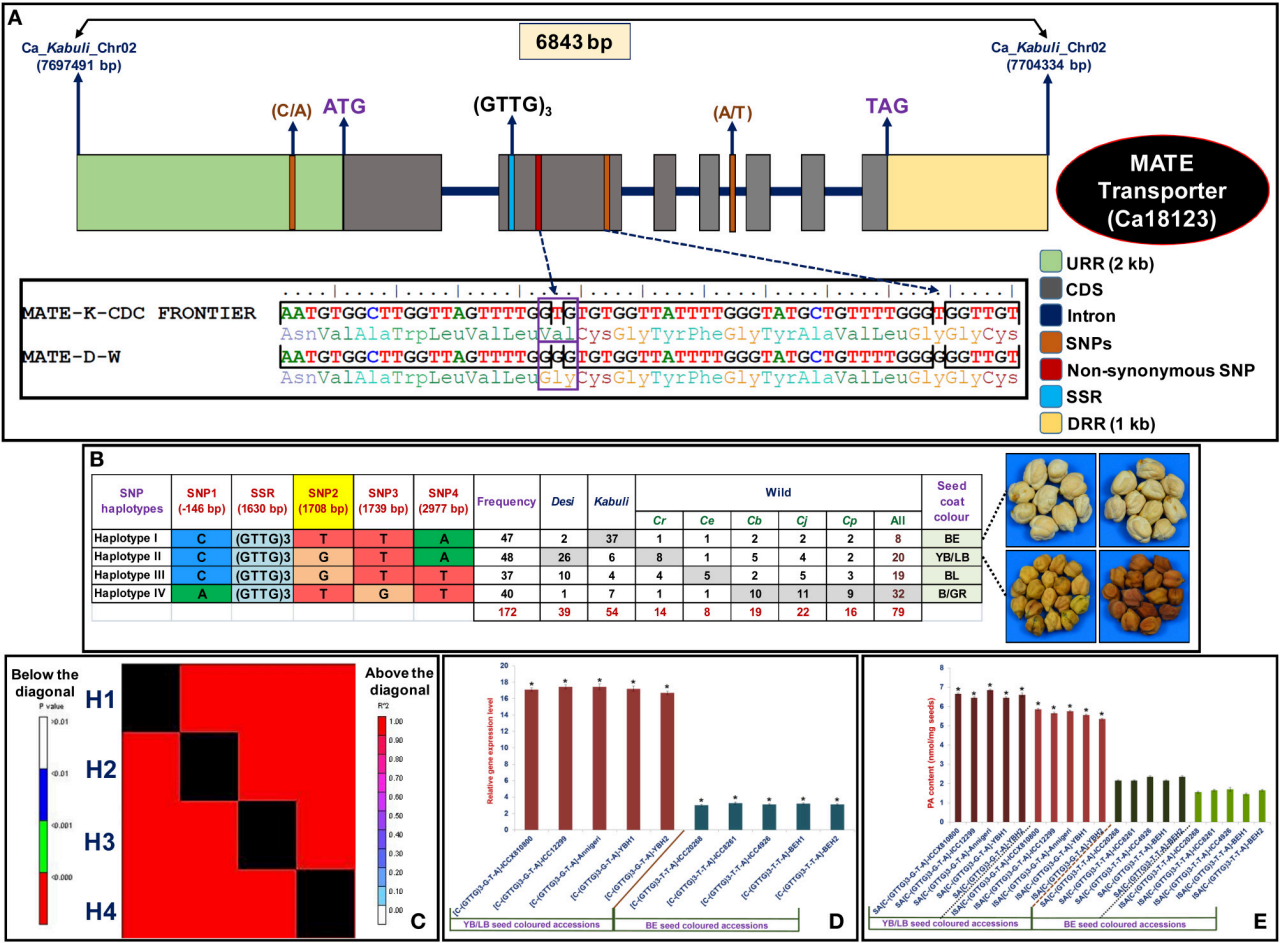
**The gene haplotype-based LD/association mapping, expression profiling and PA content estimation in a strong seed coat color-associated MATE secondary transporter ***kabuli*** gene (Ca18123), validating its potential for seed coat color regulation in chickpea**. The genotyping of four SNP (one non-synonymous SNPs) and one SSR markers in different coding and non-coding sequence components of gene **(A)** among 172 cultivated and wild chickpea accessions constituted four haplotypes **(B)**. The haplotypes I: [C-(GTTG)_3_-T-T-A] and II: [C-(GTTG)_3_-G-T-A] discriminated by one non-synonymous SNP (T/G at 1708 bp, yellow shaded) in the CDS of gene showed strong association potential for BE and LB/YB seed coat color differentiation, respectively. The four haplotype marker-based genotyping information imparted higher LD estimate covering the entire 6843 bp sequenced region of the gene **(C)**. The columns below the diagonal denote the correlation frequency (r^2^) among a pair of four different haplotypes constituted in a gene, whereas columns above the diagonal indicate the *P*-value significance (*P* < 0.01) of LD estimates (r^2^) for these haplotype combinations at 1000 permutation. **(D)** The differential expression of BE and YB/LB seed color-associated MATE gene haplotypes (I and II) in the mature seed coats of accessions, mapping parents and homozygous RIL individuals representing BE and LB/YB seed colored haplotype groups. The *elongation factor-1 alpha* gene was utilized as an internal control in the RT-PCR assay. **(E)** The soluble and insoluble PA contents (nmol/mg seeds) estimated in the mature seed coats of *desi* and *kabuli* chickpea accessions, mapping parents and homozygous RIL individuals representing BE and LB/YB seed colored haplotypes. Each bars represent the mean (± standard error) of three independent biological replicates along with two technical replicates for each sample used in RT-PCR and PA estimation assays. ^*^Significant differences in expression of gene haplotypes in mature seeds of LB/YB and BE seed colored accessions as compared to their leaf and seed (without seed coats) tissues at *p* < 0.001 (LSD-ANOVA significance). ^*^Significant variation in PA contents in the matured seeds of LB/YB seed colored accessions as compared to BE seed coat colored accessions at *p* < 0.001 (ANOVA significance). YBH (yellow brown homozygous) and BEH (beige homozygous) RIL mapping individuals.

For molecular haplotyping of a strong seed color-associated gene (validated by association analysis, QTL mapping and expression profiling), the 6843 bp cloned amplicon covering the entire 2 kb URR, 7 exons, 1 kb DRR and 6 intronic regions of MATE secondary transporter *kabuli* gene (Ca18123) were sequenced and compared among 93 cultivated and 79 wild chickpea accessions. These analyses identified four SNPs, including one URR and two coding SNPs as well as one coding SSR in the MATE gene (Figure 4A, Table S8). This includes one missense non-synonymous coding SNPs (T/G) showing valine (GTG) to glycine (GGG) amino acid substitution in the gene. The gene-based haplotype analysis by integrating the genotyping data of four SNPs and one SSR among 172 accessions constituted four haplotypes (Figure 4B). The haplotype-specific LD mapping and association analysis using four marker-based haplotypes identified in a *MATE* gene inferred strong association potential (P: 2.3 × 10^−11^ with R^2^: 45%) of the gene with seed coat color trait. Moreover, we observed a significant higher degree of LD (*r*^2^ > 0.85 with *P* < 1.1 × 10^−6^) resolution across the entire 6843 bp sequenced region of this strong seed color-associated gene (Figure 4C). Remarkably, two specific haplotypes, haplotype I [C-(GTTG)_3_-T-T-A] and haplotype II [C-(GTTG)_3_-G-T-A] affected by one functional non-synonymous coding SNPs (T/G) in the *MATE* gene had strong association potential for BE and YB/LB seed coat color differentiation, respectively (Figure 4B). The haplotypes I and II than other two haplotypes (III and IV) identified in the gene was represented most commonly by YB/LB and BE seed colored *desi* (66.7%) and *kabuli* (68.5%) accessions, respectively. A higher down-regulated expression (~5-fold) of BE seed color-specific gene haplotype I was observed in mature seed developmental stages of BE seed colored three contrasting chickpea accessions (ICC 20268, ICC 8261, and ICC 4926) as well as mapping parents and homozygous RIL individuals compared with three YB/LB seed colored accessions (ICCX 810800, ICC 12299, and Annigeri), mapping parents and homozygous mapping individuals (Figure 4D). To correlate the differential expression/regulatory pattern of MATE gene-encoding transcript with proanthocyanidins (PA) synthesis/accumulation (Lepiniec et al., 2006; Marinova et al., 2007; Zhao and Dixon, 2009; Zhao et al., 2010), we estimated the soluble and insoluble PA content (three replicates) in aforementioned mature seeds of six contrasting accessions as well as mapping parents and homozygous RIL individuals representing BE and YB/LB-specific haplotype groups I and II, respectively. This detected a significant reduction of both soluble and insoluble PA contents in the mature seed coats of BE seed colored accessions (soluble and insoluble PA: 2.1 and 1.6 nmol/mg seeds, respectively) than that of LB/YB (6.7 and 5.9 nmol/mg seeds) seed colored accessions (Figure 4E). Collectively, a strong association potential of MATE gene with seed color trait was ensured by integrating GWAS and candidate gene-based association analysis with QTL mapping, differential expression profiling, SNP-SSR marker-based high-resolution gene-specific LD mapping/haplotyping, haplotype-specific transcript profiling and PA estimation (soluble and insoluble) assay. These analyses identified novel natural allelic variant (T/G) showing non-synonymous amino acid substitution (valine to glycine) and potential haplotypes [C-(GTTG)_3_-T-T-A] and [C-(GTTG)_3_-G-T-A] in a MATE gene regulating BE and YB/LB seed coat color differentiation, respectively in *kabuli* and *desi* chickpea accessions. The implication of such combinatorial approach for rapid delineation of functionally relevant genes/QTLs controlling diverse agronomic traits, including starch biosynthesis, seed pericarp coloration/pigmentation, seed shattering and seed setting rate in rice as well as seed size/weight and pod number in chickpea have been well understood (Konishi et al., 2006; Sweeney et al., 2007; Kujur et al., 2013, 2014; Li et al., 2013; Saxena et al., 2014a; Bajaj et al., 2015). Therefore, natural allelic variants and haplotypes identified in a strong seed color-associated MATE gene by use of an integrated genomic approach can have potential to decipher its complex transcriptional regulatory function of seed coat coloration during seed development and subsequently for marker-assisted genetic enhancement of chickpea for preferred seed color.

We observed sharing of all four SNP-SSR marker-based haplotypes among 93 cultivated *desi* and *kabuli* as well as 79 wild accessions representing primary, secondary and tertiary gene pools (Figure 4B). The B/GR and BE seed color-associated haplotypes IV and I were represented most (40.5%) and least (10.1%) in the wild chickpea accessions, respectively. However, the B/GR-associated haplotype IV was primarily represented by the wild chickpea accessions (38%) of secondary gene pool (*C. bijugum, C. judaicum*, and *C. pinnatifidum*) (Figure 4B). The wild accessions of *C. reticulatum* from primary gene pool had maximum YB/LB (10.1%) seed color-associated haplotype II. A maximum haplotypes sharing of BE seed color-associated haplotype I in cultivated *kabuli* accessions with YB/LB seed color-associated haplotype II in *desi* accessions following YB/LB seed color-associated haolotype II between *desi* and wild *C. reticulatum* accessions was evident (Figure 4B). The B/GR seed color-associated haplotype IV gave evidence for its putative recombination event during chickpea domestication. These marker haplotypes sharing analyses reflected single origin of “G” functional SNP allele primarily from *C. reticulatum*, and subsequently it was introgressed into most of the LB/YB seed colored *desi* accessions as compared to *kabuli* accessions with BE seed coat color. The nucleotide diversity (θπ and θω) and Tajima's D estimation (Table S3) further inferred the occurrence of very strong purifying selection (with reduced D) in favor of retention of LB/YB seed coat color-associated “G” SNP locus and haplotype II in the MATE secondary transporter gene specifically in *desi* and wild *C. reticulatum* accessions toward assortment of more preferential LB/YB seed coat color in chickpea. These findings may be a result of domestication bottlenecks coupled with artificial selection/modern breeding efforts that are constantly practiced during the genetic improvement program of chickpea accessions for diverse seed coat color characteristics of high consumer preference and trade value. These findings infer that the natural allelic/haplotype variation discovered in the MATE gene are possibly associated with seed coat color trait evolution and therefore, the seed color is expected to represent an important component of domestication trait in chickpea.

The chickpea *MATE* gene is a homologs of *Arabidopsis TT12* (transparent testa 12) and *Medicago MATE1* genes encodes a membrane protein of multidrug and toxic compound extrusion (MATE) secondary transporter (Debeaujon et al., 2001). The detailed phenotypic characterization of *tt12* and *mate1* mutants and complementation analysis of their corresponding genes in *Arabidopsis* and *Medicago*, respectively inferred that these tonoplastic late biosynthetic genes mediate transportation of precursors for proanthocyanidin (PA) biosynthesis into the vacuoles of seed coat endothelial cells in developing immature seeds for their coloration/pigmentation (Lepiniec et al., 2006; Marinova et al., 2007; Zhao and Dixon, 2009; Zhao et al., 2010). The use of combinatorial approach of GWAS, QTL mapping, transcript profiling, gene-based marker haplotyping/LD mapping and PA estimation assay indicate that BE seed color-associated haplotype I [C-(GTTG)_3_-T-T-A] identified in a MATE gene might be involved in down-regulation/lower expression and accumulation of its encoding transcript, which led to reduced synthesis of both soluble and insoluble PAs in pericarps of BE seed colored mature seeds than that of YB/LB seed colored mature seeds with haplotype II [C-(GTTG)_3_-G-T-A]. These results are in agreement with earlier observations on lower accumulation of PAs (soluble/insoluble) in the vacuole endothelial cells of mature seed coat during later stages of seed development and their involvement in differential seed coat/pericarp coloration in crop plants (Abrahams et al., 2003; Kitamura et al., 2004; Liu et al., 2014). However, detailed molecular characterization of *MATE* secondary transporter gene is required to decipher its underlying regulatory mechanism, evolutionary cues and biochemical interactions governing diverse seed coat coloration in *desi, kabuli* and wild accessions during chickpea domestication. Therefore, the strong seed color-regulatory MATE gene once functionally well characterized, can be utilized in marker-assisted seed color breeding for developing varieties with improved seed coat color of consumer preference and trade value in chickpea.

### Conflict of interest statement

The reviewer Bharadwaj Chellapilla declares that, despite being affiliated with the same institute and having previously collaborated with the author Shailesh Tripathi, the review process was handled objectively. The authors declare that the research was conducted in the absence of any commercial or financial relationships that could be construed as a potential conflict of interest.
