# Supplementary material for: A Genome-wide Combinatorial Strategy Dissects Complex Genetic Architecture of Seed Coat Color in Chickpea
Source: Front Plant Sci. 2015 Nov 17;6:979. doi: 10.3389/fpls.2015.00979 (PMC4647070; doi:10.3389/fpls.2015.00979)
Supplement: Supplementary file 1 [file Table1.PDF]

Table S1: 172 cultivated (*desi* and *kabuli*) and wild chickpea accessions used for seed colour trait association mapping and molecular haplotyping

S.N.	*Cicer* species	Accession No.	Cultivar types	Geographical origin
**1**	**C. arietinum (annual cultivated)**	ICC5590	*Desi*	India
**2**		ICC6013	*Desi*	India
**3**		ICC5002	*Desi*	India
**4**		ICC7184	*Desi*	Turkey
**5**		*#$ICC4926	*Desi*	India
**6**		ICC4657	*Desi*	India
**7**		*#$ICC12299	*Desi*	Nepal
**8**		ICC456	*Desi*	India
**9**		ICC12726	*Desi*	Ethiopia
**10**		ICC11944	*Desi*	Nepal
**11**		ICC11498	*Desi*	India
**12**		ICC2072	*Desi*	India
**13**		ICC9737	*Desi*	Afghanistan
**14**		ICC9002	*Desi*	Iran
**15**		ICC16374	*Desi*	Malawi
**16**		ICC12028	*Desi*	Mexico
**17**		ICC8318	*Desi*	India
**18**		ICC15610	*Desi*	India
**19**		ICC4918	*Desi*	India
**20**		ICC1836	*Desi*	India
**21**		*#ICC15061	*Desi*	India
**22**		*#ICC4958	*Desi*	Central India
**23**		*#ICCV10	*Desi*	India
**24**		*#$ICCX-810800	*Desi*	Northern India
**25**		#IC296132	*Desi*	Northern India
**26**		#IC296131	*Desi*	Northern India
**27**		BGD112	*Desi*	Northern India
**28**		#ICC4951	*Desi*	Central India
**29**		ICC8933	*Desi*	Northern India
**30**		ICCV93954	*Desi*	Southern India
**31**		BGD72	*Desi*	Northern India
**32**		#IC296133	*Desi*	Northern India
**33**		ICCV92944	*Desi*	Central India
**34**		ICCC37	*Desi*	Southern India
**35**		ICCX-820065	*Desi*	Western India
**36**		Pusa5028	*Desi*	Northern India
**37**		Pusa547	*Desi*	Northern India
**38**		ICCV96970	*desi*	India
**39**		G130	*Desi*	India
**40**		ICC13523	*Kabuli*	Iran
**41**		ICC15802	*Kabuli*	Syria
**42**		ICC13077	*Kabuli*	India
**43**		ICC15264	*Kabuli*	Iran
**44**		ICC7308	*Kabuli*	Peru
**45**		ICC15435	*Kabuli*	Morocco

**46**		ICC7295	*Kabuli*	Tunisia
**47**		ICC12328	*Kabuli*	Cyprus
**48**		ICC14446	*Kabuli*	Italy
**49**		ICC10755	*Kabuli*	Turkey
**50**		ICC14190	*Kabuli*	India
**51**		ICC8042	*Kabuli*	Iran
**52**		*#$ICC8261	*Kabuli*	Turkey
**53**		ICC15333	*Kabuli*	Iran
**54**		ICC15512	*Kabuli*	Morocco
**55**		ICC10884	*Kabuli*	Ethiopia
**56**		ICC15551	*Kabuli*	Australia
**57**		ICC15725	*Kabuli*	Syrian Arab Republic
**58**		*#ICC6204	*Kabuli*	Spain
**59**		ICC6210	*Kabuli*	Spain
**60**		ICC7654	*Kabuli*	Turkey
**61**		ICC11847	*Kabuli*	Chile
**62**		ICC11749	*Kabuli*	Chile
**63**		ICC14199	*Kabuli*	Mexico
**64**		ICC15518	*Kabuli*	Morocco
**65**		ICC16814	*Kabuli*	Portugal
**66**		ICC16811	*Kabuli*	Portugal
**67**		ICC6253	*Kabuli*	Morocco
**68**		ICC11301	*Kabuli*	USA
**69**		ICC10749	*Kabuli*	Turkey
**70**	***C. arietinum* (annual cultivated)**	ICC14216	*Kabuli*	Mexico
**71**		ICC14203	*Kabuli*	Mexico
**72**		ICC11742	*Kabuli*	Chile
**73**		ICC13821	*Kabuli*	Ethiopia
**74**		ICC14462	*Kabuli*	India
**75**		ICC14220	*Kabuli*	Kenya
**76**		ICC15944	*Kabuli*	USA
**77**		ICC11303	*Kabuli*	Chile
**78**		ICC12034	*Kabuli*	Mexico
**79**		ICC7346	*Kabuli*	Mexico
**80**		ICC15994	*Kabuli*	Spain
**81**		ICC18591	*Kabuli*	Mexico
**82**		ICC8155	*Kabuli*	USA
**83**		#ICCV92311	*Kabuli*	India
**84**		*#$ICC20268	*Kabuli*	Central India
**85**		*#PhuleG0515	*Kabuli*	Central India
**86**		ICCV96329	*Kabuli*	Southern India
**87**		IC296376	*Kabuli*	Northern India
**88**		#ICCV2	*Kabuli*	Southern India
**89**		IC449069	*Kabuli*	Northern India
**90**		BGD1105	*Kabuli*	Northern India
**91**		*#$Annigeri	*Kabuli*	India
**92**		#L550	*Kabuli*	India
**93**		BG2024	*kabuli*	India

**94**	***C. reticulatum* (annual wild)**	#ICC17160	Wild	Turkey
**95**		ILWC233	Wild	Turkey
**96**		ILWC253	Wild	Turkey
**97**		ILWC254	Wild	Turkey
**98**		ILWC237	Wild	Turkey
**99**		ILWC257	Wild	Turkey
**100**		ILWC218	Wild	Turkey
**101**		ILWC247	Wild	Turkey
**102**		ILWC229	Wild	Turkey
**103**		ILWC219	Wild	Turkey
**104**		ILWC242	Wild	Turkey
**105**		ILWC21	Wild	Turkey
**106**		ILWC36	Wild	Turkey
**107**		ILWC216	Wild	Turkey
**108**	***C. echinospermum* (annual wild)**	#ICC17159	Wild	Turkey
**109**		ILWC35	Wild	Turkey
**110**		IG135418	Wild	Syrian Arab Republic
**111**		ILWC239	Wild	Turkey
**112**		ILWC245	Wild	Turkey
**113**		ILWC238	Wild	Turkey
**114**		ILWC246	Wild	Turkey
**115**		ILWC288	Wild	Turkey
**116**	***C. judaicum* (annual wild)**	ICC182	Wild	ICRISAT, India
**117**		#ICC17150	Wild	Lebanon
**118**		ILWC95	Wild	ICRISAT, India
**119**		ILWC31	Wild	Jordan
**120**		ILWC185	Wild	ICRISAT, India
**121**		ICC17148	Wild	Lebanon
**122**		ILWC280	Wild	Syrian Arab Republic
**123**		ILWC283	Wild	Syrian Arab Republic
**124**		ILWC20	Wild	Israel
**125**		ILWC211	Wild	Syrian Arab Republic
**126**		ILWC30	Wild	Israel
**127**		ILWC48	Wild	Syrian Arab Republic
**128**		ILWC50	Wild	Syrian Arab Republic
**129**		ILWC207	Wild	Syrian Arab Republic
**130**		ILWC45	Wild	Syrian Arab Republic
**131**		ILWC38	Wild	Lebanon
**132**		ILWC278	Wild	Syrian Arab Republic
**133**		ILWC275	Wild	Lebanon
**134**		ILWC223	Wild	Lebanon
**135**		ILWC256	Wild	Jordan
**136**		ILWC273	Wild	Lebanon
**137**		ILWC4	Wild	Lebanon
**138**	***C. bijugum* (annual wild)**	#ILWC42	Wild	Syrian Arab Republic
**139**		IG136792	Wild	Syrian Arab Republic
**140**		IG136786	Wild	Syrian Arab Republic
**141**		ILWC277	Wild	Syrian Arab Republic
**142**		ILWC8	Wild	Turkey

**143**	***C. bijugum* (annual wild)**	ILWC228	Wild	Turkey
**144**		ILWC240	Wild	Turkey
**145**		ILWC241	Wild	Turkey
**146**		ILWC260	Wild	Turkey
**147**		ILWC217	Wild	Turkey
**148**		ILWC209	Wild	Syrian Arab Republic
**149**		ILWC227	Wild	Turkey
**150**		ILWC284	Wild	Turkey
**151**		ILWC7	Wild	Turkey
**152**		ILWC220	Wild	Turkey
**153**		IG136796	Wild	Syrian Arab Republic
**154**		ILWC285	Wild	Turkey
**155**		ILWC286	Wild	Turkey
**156**		ILWC32	Wild	Turkey
**157**	***C. pinnatifidum* (annual wild)**	ILWC9	Wild	Turkey
**158**		ILWC22	Wild	Turkey
**159**		#IG136820	Wild	Syrian Arab Republic
**160**		ILWC251	Wild	Turkey
**161**		ILWC29	Wild	Turkey
**162**		ILWC33	Wild	Turkey
**163**		ILWC49	Wild	Syrian Arab Republic
**164**		ILWC226	Wild	Turkey
**165**		ILWC248	Wild	Turkey
**166**		ILWC249	Wild	Turkey
**167**		ILWC236	Wild	Turkey
**168**		ILWC225	Wild	Turkey
**169**		ILWC51	Wild	Turkey
**170**		ILWC250	Wild	Turkey
**171**		ILWC289	Wild	Turkey
**172**	***C. microphyllum* (perennial wild)**	*#microphyllum*	Wild	India

*#Accessions used for mining SNPs

*Accessions selected for differential expression profiling

$Accessions used for molecular haplotyping and PA estimation
