# Supplementary material for: A Genome-wide Combinatorial Strategy Dissects Complex Genetic Architecture of Seed Coat Color in Chickpea
Source: Front Plant Sci. 2015 Nov 17;6:979. doi: 10.3389/fpls.2015.00979 (PMC4647070; doi:10.3389/fpls.2015.00979)
Supplement: Supplementary file 2 [file Table2.PDF]

**Table S2:** Structural and functional annotation of 8673 GBS-based SNPs in *kabuli* chickpea genome

| SNP IDs | Chromosomes | Physical positions (bp) | SNPs | Gene accession IDs | Sequence components of genes | Putative functions |
|---|---|---|---|---|---|---|
| CWSNP1 | Ca-Kabuli-Chr1 | 462 | [G/C] | - | Intergenic | - |
| CWSNP2 | Ca-Kabuli-Chr1 | 523 | [G/C] | - | Intergenic | - |
| CWSNP3 | Ca-Kabuli-Chr1 | 548 | [G/C] | - | Intergenic | - |
| CWSNP4 | Ca-Kabuli-Chr1 | 578 | [G/A] | - | Intergenic | - |
| CWSNP5 | Ca-Kabuli-Chr1 | 579 | [T/C] | - | Intergenic | - |
| CWSNP6 | Ca-Kabuli-Chr1 | 589 | [A/C] | - | Intergenic | - |
| CWSNP7 | Ca-Kabuli-Chr1 | 627 | [C/A] | - | Intergenic | - |
| CWSNP8 | Ca-Kabuli-Chr1 | 839 | [A/T] | - | Intergenic | - |
| CWSNP9 | Ca-Kabuli-Chr1 | 903 | [G/T] | - | Intergenic | - |
| CWSNP10 | Ca-Kabuli-Chr1 | 927 | [C/A] | - | Intergenic | - |
| CWSNP11 | Ca-Kabuli-Chr1 | 98992 | [G/T] | Ca00007 | Intron | RNA recognition motif domain |
| CWSNP12 | Ca-Kabuli-Chr1 | 108984 | [G/A] | Ca00008 | Intron | - |
| CWSNP13 | Ca-Kabuli-Chr1 | 108979 | [T/G] | Ca00008 | Intron | - |
| CWSNP14 | Ca-Kabuli-Chr1 | 129401 | [A/G] | Ca00009 | Intron | Oligopeptide transporter |
| CWSNP15 | Ca-Kabuli-Chr1 | 215066 | [G/T] | Ca00017 | Intron | Protein kinase, catalytic domain |
| CWSNP16 | Ca-Kabuli-Chr1 | 215058 | [T/C] | Ca00017 | Intron | Protein kinase, catalytic domain |

| SNP IDs | Chromosomes | Physical positions (bp) | SNPs | Gene accession IDs | Sequence components of genes | Putative functions |
|---|---|---|---|---|---|---|
| CWSNP17 | Ca-Kabuli-Chr1 | 215053 | [G/T] | Ca00017 | Intron | Protein kinase, catalytic domain |
| CWSNP18 | Ca-Kabuli-Chr1 | 215048 | [C/T] | Ca00017 | Intron | Protein kinase, catalytic domain |
| CWSNP19 | Ca-Kabuli-Chr1 | 215029 | [C/A] | Ca00017 | Intron | Protein kinase, catalytic domain |
| CWSNP20 | Ca-Kabuli-Chr1 | 215027 | [T/C] | Ca00017 | Intron | Protein kinase, catalytic domain |
| CWSNP21 | Ca-Kabuli-Chr1 | 215006 | [C/T] | Ca00017 | Intron | Protein kinase, catalytic domain |
| CWSNP22 | Ca-Kabuli-Chr1 | 292962 | [T/C] | Ca00028 | Synonymous-CDS | Proteasome component (PCI) domain |
| CWSNP23 | Ca-Kabuli-Chr1 | 292972 | [G/A] | Ca00028 | Non-Synonymous-CDS | Proteasome component (PCI) domain |
| CWSNP24 | Ca-Kabuli-Chr1 | 292998 | [T/C] | Ca00028 | Synonymous-CDS | Proteasome component (PCI) domain |
| CWSNP25 | Ca-Kabuli-Chr1 | 347620 | [A/C] | - | Intergenic | - |
| CWSNP26 | Ca-Kabuli-Chr1 | 372530 | [A/G] | Ca00038 | Intron | Protein of unknown function DUF803 |
| CWSNP27 | Ca-Kabuli-Chr1 | 406570 | [A/G] | Ca00043 | Synonymous-CDS | Glycosyl-phosphatidylinositol-anchored |
| CWSNP28 | Ca-Kabuli-Chr1 | 425149 | [T/C] | - | Intergenic | - |
| CWSNP29 | Ca-Kabuli-Chr1 | 425076 | [A/G] | - | Intergenic | - |
| CWSNP30 | Ca-Kabuli-Chr1 | 435259 | [A/G] | - | URR | - |
| CWSNP31 | Ca-Kabuli-Chr1 | 435274 | [G/T] | - | URR | - |
| CWSNP32 | Ca-Kabuli-Chr1 | 435280 | [T/C] | - | URR | - |

| SNP IDs | Chromosomes | Physical positions (bp) | SNPs | Gene accession IDs | Sequence components of genes | Putative functions |
|---|---|---|---|---|---|---|
| CWSNP33 | Ca-Kabuli-Chr1 | 435297 | [G/A] | - | URR | - |
| CWSNP34 | Ca-Kabuli-Chr1 | 435304 | [G/T] | - | URR | - |
| CWSNP35 | Ca-Kabuli-Chr1 | 435343 | [C/A] | - | URR | - |
| CWSNP36 | Ca-Kabuli-Chr1 | 435338 | [T/C] | - | URR | - |
| CWSNP37 | Ca-Kabuli-Chr1 | 435264 | [G/T] | - | URR | - |
| CWSNP38 | Ca-Kabuli-Chr1 | 441286 | [C/T] | Ca00050 | Intron | Thiamine monophosphate synthase |
| CWSNP39 | Ca-Kabuli-Chr1 | 441288 | [T/A] | Ca00050 | Intron | Thiamine monophosphate synthase |
| CWSNP40 | Ca-Kabuli-Chr1 | 441343 | [T/A] | Ca00050 | Synonymous-CDS | Thiamine monophosphate synthase |
| CWSNP41 | Ca-Kabuli-Chr1 | 446409 | [T/C] | Ca00051 | Synonymous-CDS | Helicase,C-terminal |
| CWSNP42 | Ca-Kabuli-Chr1 | 446443 | [A/G] | Ca00051 | Non-Synonymous-CDS | Helicase,C-terminal |
| CWSNP43 | Ca-Kabuli-Chr1 | 446484 | [C/T] | Ca00051 | Synonymous-CDS | Helicase,C-terminal |
| CWSNP44 | Ca-Kabuli-Chr1 | 446558 | [G/T] | Ca00051 | Intron | Helicase,C-terminal |
| CWSNP45 | Ca-Kabuli-Chr1 | 459968 | [T/G] | Ca00053 | Intron | CMP/dCMPdeaminase,zinc-binding |
| CWSNP46 | Ca-Kabuli-Chr1 | 464039 | [A/C] | Ca00053 | Synonymous-CDS | CMP/dCMPdeaminase,zinc-binding |
| CWSNP47 | Ca-Kabuli-Chr1 | 464040 | [G/C] | Ca00053 | Synonymous-CDS | CMP/dCMPdeaminase,zinc-binding |
| CWSNP48 | Ca-Kabuli-Chr1 | 464043 | [C/G] | Ca00053 | Synonymous-CDS | CMP/dCMPdeaminase,zinc-binding |

| SNP IDs | Chromosomes | Physical positions (bp) | SNPs | Gene accession IDs | Sequence components of genes | Putative functions |
|---|---|---|---|---|---|---|
| CWSNP49 | Ca-Kabuli-Chr1 | 554677 | [G/A] | Ca00064 | Intron | Glycosyltransferase,family8 |
| CWSNP50 | Ca-Kabuli-Chr1 | 571020 | [T/G] | Ca00064 | Intron | Glycosyltransferase,family8 |
| CWSNP51 | Ca-Kabuli-Chr1 | 591609 | [T/C] | Ca00066 | Intron | PeptidaseS16,Lonprotease,C-terminal |
| CWSNP52 | Ca-Kabuli-Chr1 | 712110 | [T/C] | Ca00086 | Non-Synonymous-CDS | PeptidaseC50,separase |
| CWSNP53 | Ca-Kabuli-Chr1 | 741514 | [G/T] | - | DRR | - |
| CWSNP54 | Ca-Kabuli-Chr1 | 741535 | [C/T] | - | DRR | - |
| CWSNP55 | Ca-Kabuli-Chr1 | 742043 | [G/A] | - | DRR | - |
| CWSNP56 | Ca-Kabuli-Chr1 | 742026 | [T/G] | - | DRR | - |
| CWSNP57 | Ca-Kabuli-Chr1 | 750693 | [C/T] | - | URR | - |
| CWSNP58 | Ca-Kabuli-Chr1 | 777273 | [C/T] | - | DRR | - |
| CWSNP59 | Ca-Kabuli-Chr1 | 777240 | [G/A] | - | DRR | - |
| CWSNP60 | Ca-Kabuli-Chr1 | 790000 | [C/T] | - | URR | - |
| CWSNP61 | Ca-Kabuli-Chr1 | 790102 | [G/T] | - | URR | - |
| CWSNP62 | Ca-Kabuli-Chr1 | 864382 | [T/C] | - | Intergenic | - |
| CWSNP63 | Ca-Kabuli-Chr1 | 864381 | [G/A] | - | Intergenic | - |
| CWSNP64 | Ca-Kabuli-Chr1 | 864351 | [T/C] | - | Intergenic | - |

| SNP IDs | Chromosomes | Physical positions (bp) | SNPs | Gene accession IDs | Sequence components of genes | Putative functions |
|---|---|---|---|---|---|---|
| CWSNP65 | Ca-Kabuli-Chr1 | 866872 | [T/C] | - | URR | - |
| CWSNP66 | Ca-Kabuli-Chr1 | 876633 | [T/A] | - | DRR | - |
| CWSNP67 | Ca-Kabuli-Chr1 | 881543 | [C/A] | - | DRR | - |
| CWSNP68 | Ca-Kabuli-Chr1 | 881703 | [G/T] | - | DRR | - |
| CWSNP69 | Ca-Kabuli-Chr1 | 890948 | [C/T] | Ca00110 | Intron | Calcium-binding EF-hand |
| CWSNP70 | Ca-Kabuli-Chr1 | 910486 | [T/A] | - | URR | - |
| CWSNP71 | Ca-Kabuli-Chr1 | 996439 | [T/C] | - | Intergenic | - |
| CWSNP72 | Ca-Kabuli-Chr1 | 1008895 | [A/G] | - | Intergenic | - |
| CWSNP73 | Ca-Kabuli-Chr1 | 1009058 | [A/C] | - | DRR | - |
| CWSNP74 | Ca-Kabuli-Chr1 | 1018966 | [C/G] | - | Intergenic | - |
| CWSNP75 | Ca-Kabuli-Chr1 | 1023550 | [C/T] | - | Intergenic | - |
| CWSNP76 | Ca-Kabuli-Chr1 | 1032734 | [A/T] | Ca00128 | Non-Synonymous-CDS | K+ potassium transporter |
| CWSNP77 | Ca-Kabuli-Chr1 | 1059209 | [A/G] | Ca00129 | Non-Synonymous-CDS | IQ motif, EF-hand binding site |
| CWSNP78 | Ca-Kabuli-Chr1 | 1059311 | [A/G] | Ca00129 | Non-Synonymous-CDS | IQ motif, EF-hand binding site |
| CWSNP79 | Ca-Kabuli-Chr1 | 1083388 | [G/A] | - | URR | - |
| CWSNP80 | Ca-Kabuli-Chr1 | 1083403 | [C/T] | - | URR | - |

| SNP IDs | Chromosomes | Physical positions (bp) | SNPs | Gene accession IDs | Sequence components of genes | Putative functions |
|---|---|---|---|---|---|---|
| CWSNP81 | Ca-Kabuli-Chr1 | 1085228 | [A/C] | Ca00134 | Intron | Peptidase C14,caspase catalytic |
| CWSNP82 | Ca-Kabuli-Chr1 | 1085230 | [T/G] | Ca00134 | Intron | Peptidase C14,caspase catalytic |
| CWSNP83 | Ca-Kabuli-Chr1 | 1085238 | [A/G] | Ca00134 | Intron | Peptidase C14,caspase catalytic |
| CWSNP84 | Ca-Kabuli-Chr1 | 1085254 | [A/C] | Ca00134 | Intron | Peptidase C14,caspase catalytic |
| CWSNP85 | Ca-Kabuli-Chr1 | 1085263 | [A/C] | Ca00134 | Intron | Peptidase C14,caspase catalytic |
| CWSNP86 | Ca-Kabuli-Chr1 | 1085293 | [G/A] | Ca00134 | Intron | Peptidase C14,caspase catalytic |
| CWSNP87 | Ca-Kabuli-Chr1 | 1091548 | [T/A] | - | URR | - |
| CWSNP88 | Ca-Kabuli-Chr1 | 1091534 | [A/C] | - | URR | - |
| CWSNP89 | Ca-Kabuli-Chr1 | 1091504 | [C/T] | - | URR | - |
| CWSNP90 | Ca-Kabuli-Chr1 | 1098703 | [C/G] | - | Intergenic | - |
| CWSNP91 | Ca-Kabuli-Chr1 | 1098789 | [A/G] | - | Intergenic | - |
| CWSNP92 | Ca-Kabuli-Chr1 | 1115668 | [C/T] | - | URR | - |
| CWSNP93 | Ca-Kabuli-Chr1 | 1116043 | [C/T] | - | URR | - |
| CWSNP94 | Ca-Kabuli-Chr1 | 1140313 | [A/C] | - | DRR | - |
| CWSNP95 | Ca-Kabuli-Chr1 | 1140405 | [A/T] | - | DRR | - |
| CWSNP96 | Ca-Kabuli-Chr1 | 1150973 | [C/T] | - | DRR | - |

| SNP IDs | Chromosomes | Physical positions (bp) | SNPs | Gene accession IDs | Sequence components of genes | Putative functions |
|---|---|---|---|---|---|---|
| CWSNP97 | Ca-Kabuli-Chr1 | 1159383 | [C/A] | - | DRR | - |
| CWSNP98 | Ca-Kabuli-Chr1 | 1215024 | [T/C] | - | DRR | - |
| CWSNP99 | Ca-Kabuli-Chr1 | 1269180 | [C/A] | Ca00155 | Non-Synonymous-CDS | PeptidaseC48,SUMO/Sentrin/Ubl1 |
| CWSNP100 | Ca-Kabuli-Chr1 | 1278884 | [G/T] | - | Intergenic | - |
| CWSNP101 | Ca-Kabuli-Chr1 | 1278997 | [T/A] | - | Intergenic | - |
| CWSNP102 | Ca-Kabuli-Chr1 | 1295527 | [T/G] | - | URR | - |
| CWSNP103 | Ca-Kabuli-Chr1 | 1335518 | [A/C] | - | Intergenic | - |
| CWSNP104 | Ca-Kabuli-Chr1 | 1385553 | [C/A] | - | Intergenic | - |
| CWSNP105 | Ca-Kabuli-Chr1 | 1385554 | [G/T] | - | Intergenic | - |
| CWSNP106 | Ca-Kabuli-Chr1 | 1390073 | [A/G] | - | Intergenic | - |
| CWSNP107 | Ca-Kabuli-Chr1 | 1411501 | [C/T] | - | URR | - |
| CWSNP108 | Ca-Kabuli-Chr1 | 1411498 | [T/A] | - | URR | - |
| CWSNP109 | Ca-Kabuli-Chr1 | 1411495 | [A/G] | - | URR | - |
| CWSNP110 | Ca-Kabuli-Chr1 | 1428222 | [C/G] | - | DRR | - |
| CWSNP111 | Ca-Kabuli-Chr1 | 1428251 | [T/G] | - | DRR | - |
| CWSNP112 | Ca-Kabuli-Chr1 | 1428264 | [T/G] | - | DRR | - |

| SNP IDs | Chromosomes | Physical positions (bp) | SNPs | Gene accession IDs | Sequence components of genes | Putative functions |
|---|---|---|---|---|---|---|
| CWSNP113 | Ca-Kabuli-Chr1 | 1434121 | [A/G] | Ca00174 | Non-Synonymous-CDS | DNA polymeraseV |
| CWSNP114 | Ca-Kabuli-Chr1 | 1434883 | [A/G] | - | Intergenic | - |
| CWSNP115 | Ca-Kabuli-Chr1 | 1434875 | [A/G] | - | Intergenic | - |
| CWSNP116 | Ca-Kabuli-Chr1 | 1449848 | [G/A] | Ca00177 | Synonymous-CDS | Phox/Bem1p |
| CWSNP117 | Ca-Kabuli-Chr1 | 1449913 | [T/G] | Ca00177 | Non-Synonymous-CDS | Phox/Bem1p |
| CWSNP118 | Ca-Kabuli-Chr1 | 1449949 | [G/A] | Ca00177 | Non-Synonymous-CDS | Phox/Bem1p |
| CWSNP119 | Ca-Kabuli-Chr1 | 1449980 | [A/G] | Ca00177 | Synonymous-CDS | Phox/Bem1p |
| CWSNP120 | Ca-Kabuli-Chr1 | 1461574 | [T/A] | - | URR | - |
| CWSNP121 | Ca-Kabuli-Chr1 | 1461600 | [T/C] | - | URR | - |
| CWSNP122 | Ca-Kabuli-Chr1 | 1467056 | [A/G] | - | DRR | - |
| CWSNP123 | Ca-Kabuli-Chr1 | 1528325 | [G/A] | Ca00185 | Intron | Inorganic pyrophosphatase |
| CWSNP124 | Ca-Kabuli-Chr1 | 1546679 | [G/C] | Ca00187 | Synonymous-CDS | - |
| CWSNP125 | Ca-Kabuli-Chr1 | 1655018 | [G/A] | Ca00203 | Non-Synonymous-CDS | Kinesin , motor domain |
| CWSNP126 | Ca-Kabuli-Chr1 | 1655012 | [G/T] | Ca00203 | Non-Synonymous-CDS | Kinesin , motor domain |
| CWSNP127 | Ca-Kabuli-Chr1 | 1738179 | [G/C] | - | DRR | - |
| CWSNP128 | Ca-Kabuli-Chr1 | 1772813 | [T/C] | - | DRR | - |

| SNP IDs | Chromosomes | Physical positions (bp) | SNPs | Gene accession IDs | Sequence components of genes | Putative functions |
|---|---|---|---|---|---|---|
| CWSNP129 | Ca-Kabuli-Chr1 | 1782760 | [G/A] | Ca00224 | Intron | Zinc finger,RING-type |
| CWSNP130 | Ca-Kabuli-Chr1 | 1782793 | [G/T] | Ca00224 | Intron | Zinc finger,RING-type |
| CWSNP131 | Ca-Kabuli-Chr1 | 1782808 | [G/C] | Ca00224 | Intron | Zinc finger,RING-type |
| CWSNP132 | Ca-Kabuli-Chr1 | 1782953 | [G/A] | Ca00224 | Intron | Zinc finger,RING-type |
| CWSNP133 | Ca-Kabuli-Chr1 | 1782948 | [T/C] | Ca00224 | Intron | Zinc finger,RING-type |
| CWSNP134 | Ca-Kabuli-Chr1 | 1782930 | [G/A] | Ca00224 | Intron | Zinc finger,RING-type |
| CWSNP135 | Ca-Kabuli-Chr1 | 1782929 | [C/T] | Ca00224 | Intron | Zinc finger,RING-type |
| CWSNP136 | Ca-Kabuli-Chr1 | 1783207 | [G/A] | Ca00224 | Intron | Zinc finger,RING-type |
| CWSNP137 | Ca-Kabuli-Chr1 | 1783277 | [G/A] | Ca00224 | Intron | Zinc finger,RING-type |
| CWSNP138 | Ca-Kabuli-Chr1 | 1783280 | [A/C] | Ca00224 | Intron | Zinc finger,RING-type |
| CWSNP139 | Ca-Kabuli-Chr1 | 1783923 | [C/A] | Ca00224 | Non-Synonymous-CDS | Zinc finger,RING-type |
| CWSNP140 | Ca-Kabuli-Chr1 | 1784006 | [T/C] | Ca00224 | Synonymous-CDS | Zinc finger,RING-type |
| CWSNP141 | Ca-Kabuli-Chr1 | 1788958 | [T/A] | - | DRR | - |
| CWSNP142 | Ca-Kabuli-Chr1 | 1789087 | [T/C] | - | DRR | - |
| CWSNP143 | Ca-Kabuli-Chr1 | 1802992 | [C/G] | - | DRR | - |
| CWSNP144 | Ca-Kabuli-Chr1 | 1858073 | [C/T] | Ca00233 | Intron | Inositol monophosphatase |

| SNP IDs | Chromosomes | Physical positions (bp) | SNPs | Gene accession IDs | Sequence components of genes | Putative functions |
|---|---|---|---|---|---|---|
| CWSNP145 | Ca-Kabuli-Chr1 | 1858078 | [T/C] | Ca00233 | Intron | Inositol monophosphatase |
| CWSNP146 | Ca-Kabuli-Chr1 | 1858084 | [C/T] | Ca00233 | Intron | Inositol monophosphatase |
| CWSNP147 | Ca-Kabuli-Chr1 | 1858132 | [T/A] | Ca00233 | Intron | Inositol monophosphatase |
| CWSNP148 | Ca-Kabuli-Chr1 | 1858156 | [C/T] | Ca00233 | Intron | Inositol monophosphatase |
| CWSNP149 | Ca-Kabuli-Chr1 | 1858177 | [T/C] | Ca00233 | Synonymous-CDS | Inositol monophosphatase |
| CWSNP150 | Ca-Kabuli-Chr1 | 1858159 | [A/G] | Ca00233 | Intron | Inositol monophosphatase |
| CWSNP151 | Ca-Kabuli-Chr1 | 1858509 | [C/T] | Ca00233 | Synonymous-CDS | Inositol monophosphatase |
| CWSNP152 | Ca-Kabuli-Chr1 | 1858608 | [T/A] | Ca00233 | Intron | Inositol monophosphatase |
| CWSNP153 | Ca-Kabuli-Chr1 | 1858600 | [G/A] | Ca00233 | Intron | Inositol monophosphatase |
| CWSNP154 | Ca-Kabuli-Chr1 | 1859789 | [C/T] | Ca00233 | Intron | Inositol monophosphatase |
| CWSNP155 | Ca-Kabuli-Chr1 | 1859857 | [A/G] | Ca00233 | Intron | Inositol monophosphatase |
| CWSNP156 | Ca-Kabuli-Chr1 | 1859914 | [G/A] | Ca00233 | Intron | Inositol monophosphatase |
| CWSNP157 | Ca-Kabuli-Chr1 | 1859902 | [G/A] | Ca00233 | Intron | Inositol monophosphatase |
| CWSNP158 | Ca-Kabuli-Chr1 | 1861696 | [C/T] | Ca00233 | Synonymous-CDS | Inositol monophosphatase |
| CWSNP159 | Ca-Kabuli-Chr1 | 1861707 | [T/G] | Ca00233 | Intron | Inositol monophosphatase |
| CWSNP160 | Ca-Kabuli-Chr1 | 1861722 | [T/A] | Ca00233 | Intron | Inositol monophosphatase |

| SNP IDs | Chromosomes | Physical positions (bp) | SNPs | Gene accession IDs | Sequence components of genes | Putative functions |
|---|---|---|---|---|---|---|
| CWSNP161 | Ca-Kabuli-Chr1 | 1861729 | [T/C] | Ca00233 | Intron | Inositol monophosphatase |
| CWSNP162 | Ca-Kabuli-Chr1 | 1861837 | [T/G] | Ca00233 | Synonymous-CDS | Inositol monophosphatase |
| CWSNP163 | Ca-Kabuli-Chr1 | 1904233 | [T/C] | Ca00238 | Intron | Reverse transcriptase |
| CWSNP164 | Ca-Kabuli-Chr1 | 1904230 | [G/T] | Ca00238 | Intron | Reverse transcriptase |
| CWSNP165 | Ca-Kabuli-Chr1 | 1904365 | [G/A] | Ca00238 | Intron | Reverse transcriptase |
| CWSNP166 | Ca-Kabuli-Chr1 | 1904391 | [A/G] | Ca00238 | Intron | Reverse transcriptase |
| CWSNP167 | Ca-Kabuli-Chr1 | 1935729 | [A/G] | - | Intergenic | - |
| CWSNP168 | Ca-Kabuli-Chr1 | 1962876 | [A/G] | Ca00244 | Non-Synonymous-CDS | Transcriptional factor B3 |
| CWSNP169 | Ca-Kabuli-Chr1 | 1968828 | [G/A] | - | Intergenic | - |
| CWSNP170 | Ca-Kabuli-Chr1 | 2002844 | [G/A] | - | URR | - |
| CWSNP171 | Ca-Kabuli-Chr1 | 2002922 | [G/T] | - | URR | - |
| CWSNP172 | Ca-Kabuli-Chr1 | 2040943 | [C/T] | - | Intergenic | - |
| CWSNP173 | Ca-Kabuli-Chr1 | 2040946 | [G/T] | - | Intergenic | - |
| CWSNP174 | Ca-Kabuli-Chr1 | 2041027 | [C/G] | - | Intergenic | - |
| CWSNP175 | Ca-Kabuli-Chr1 | 2041190 | [A/C] | - | DRR | - |
| CWSNP176 | Ca-Kabuli-Chr1 | 2041180 | [T/C] | - | DRR | - |

| SNP IDs | Chromosomes | Physical positions (bp) | SNPs | Gene accession IDs | Sequence components of genes | Putative functions |
|---|---|---|---|---|---|---|
| CWSNP177 | Ca-Kabuli-Chr1 | 2041178 | [A/C] | - | DRR | - |
| CWSNP178 | Ca-Kabuli-Chr1 | 2041174 | [C/T] | - | DRR | - |
| CWSNP179 | Ca-Kabuli-Chr1 | 2041154 | [G/C] | - | DRR | - |
| CWSNP180 | Ca-Kabuli-Chr1 | 2041132 | [C/T] | - | Intergenic | - |
| CWSNP181 | Ca-Kabuli-Chr1 | 2042071 | [C/G] | - | DRR | - |
| CWSNP182 | Ca-Kabuli-Chr1 | 2042185 | [T/C] | - | DRR | - |
| CWSNP183 | Ca-Kabuli-Chr1 | 2042152 | [G/C] | - | DRR | - |
| CWSNP184 | Ca-Kabuli-Chr1 | 2042149 | [G/T] | - | DRR | - |
| CWSNP185 | Ca-Kabuli-Chr1 | 2044289 | [A/G] | - | Intergenic | - |
| CWSNP186 | Ca-Kabuli-Chr1 | 2044263 | [A/C] | - | Intergenic | - |
| CWSNP187 | Ca-Kabuli-Chr1 | 2044359 | [C/T] | - | Intergenic | - |
| CWSNP188 | Ca-Kabuli-Chr1 | 2044380 | [G/C] | - | Intergenic | - |
| CWSNP189 | Ca-Kabuli-Chr1 | 2044408 | [A/C] | - | Intergenic | - |
| CWSNP190 | Ca-Kabuli-Chr1 | 2044414 | [G/A] | - | Intergenic | - |
| CWSNP191 | Ca-Kabuli-Chr1 | 2044474 | [A/G] | - | Intergenic | - |
| CWSNP192 | Ca-Kabuli-Chr1 | 2050232 | [A/T] | - | Intergenic | - |

| SNP IDs | Chromosomes | Physical positions (bp) | SNPs | Gene accession IDs | Sequence components of genes | Putative functions |
|---|---|---|---|---|---|---|
| CWSNP193 | Ca-Kabuli-Chr1 | 2050187 | [C/G] | - | Intergenic | - |
| CWSNP194 | Ca-Kabuli-Chr1 | 2050479 | [A/C] | - | URR | - |
| CWSNP195 | Ca-Kabuli-Chr1 | 2050469 | [T/C] | - | URR | - |
| CWSNP196 | Ca-Kabuli-Chr1 | 2050467 | [A/C] | - | URR | - |
| CWSNP197 | Ca-Kabuli-Chr1 | 2050465 | [A/G] | - | URR | - |
| CWSNP198 | Ca-Kabuli-Chr1 | 2050463 | [C/T] | - | URR | - |
| CWSNP199 | Ca-Kabuli-Chr1 | 2050421 | [C/T] | - | URR | - |
| CWSNP200 | Ca-Kabuli-Chr1 | 2051360 | [A/G] | - | URR | - |
| CWSNP201 | Ca-Kabuli-Chr1 | 2051379 | [C/T] | - | URR | - |
| CWSNP202 | Ca-Kabuli-Chr1 | 2053732 | [C/T] | Ca00260 | Non-Synonymous-CDS | Disease resistance protein |
| CWSNP203 | Ca-Kabuli-Chr1 | 2053744 | [C/A] | Ca00260 | Non-Synonymous-CDS | Disease resistance protein |
| CWSNP204 | Ca-Kabuli-Chr1 | 2053749 | [T/A] | Ca00260 | Non-Synonymous-CDS | Disease resistance protein |
| CWSNP205 | Ca-Kabuli-Chr1 | 2053756 | [G/T] | Ca00260 | Non-Synonymous-CDS | Disease resistance protein |
| CWSNP206 | Ca-Kabuli-Chr1 | 2053758 | [A/G] | Ca00260 | Non-Synonymous-CDS | Disease resistance protein |
| CWSNP207 | Ca-Kabuli-Chr1 | 2053784 | [C/A] | Ca00260 | Non-Synonymous-CDS | Disease resistance protein |
| CWSNP208 | Ca-Kabuli-Chr1 | 2053788 | [A/G] | Ca00260 | Non-Synonymous-CDS | Disease resistance protein |

| SNP IDs | Chromosomes | Physical positions (bp) | SNPs | Gene accession IDs | Sequence components of genes | Putative functions |
|---|---|---|---|---|---|---|
| CWSNP209 | Ca-Kabuli-Chr1 | 2053790 | [G/A] | Ca00260 | Synonymous-CDS | Disease resistance protein |
| CWSNP210 | Ca-Kabuli-Chr1 | 2053808 | [G/T] | Ca00260 | Synonymous-CDS | Disease resistance protein |
| CWSNP211 | Ca-Kabuli-Chr1 | 2053856 | [T/G] | Ca00260 | Synonymous-CDS | Disease resistance protein |
| CWSNP212 | Ca-Kabuli-Chr1 | 2053833 | [T/G] | Ca00260 | Synonymous-CDS | Disease resistance protein |
| CWSNP213 | Ca-Kabuli-Chr1 | 2053830 | [A/G] | Ca00260 | Synonymous-CDS | Disease resistance protein |
| CWSNP214 | Ca-Kabuli-Chr1 | 2053819 | [C/A] | Ca00260 | Synonymous-CDS | Disease resistance protein |
| CWSNP215 | Ca-Kabuli-Chr1 | 2053818 | [G/C] | Ca00260 | Non-Synonymous-CDS | Disease resistance protein |
| CWSNP216 | Ca-Kabuli-Chr1 | 2071456 | [T/A] | - | Intergenic | - |
| CWSNP217 | Ca-Kabuli-Chr1 | 2071448 | [A/T] | - | URR | - |
| CWSNP218 | Ca-Kabuli-Chr1 | 2071424 | [T/A] | - | Intergenic | - |
| CWSNP219 | Ca-Kabuli-Chr1 | 2071708 | [C/G] | - | Intergenic | - |
| CWSNP220 | Ca-Kabuli-Chr1 | 2071703 | [A/C] | - | Intergenic | - |
| CWSNP221 | Ca-Kabuli-Chr1 | 2071699 | [T/C] | - | Intergenic | - |
| CWSNP222 | Ca-Kabuli-Chr1 | 2071693 | [T/C] | - | Intergenic | - |
| CWSNP223 | Ca-Kabuli-Chr1 | 2071691 | [A/C] | - | Intergenic | - |
| CWSNP224 | Ca-Kabuli-Chr1 | 2071689 | [A/G] | - | Intergenic | - |

| SNP IDs | Chromosomes | Physical positions (bp) | SNPs | Gene accession IDs | Sequence components of genes | Putative functions |
|---|---|---|---|---|---|---|
| CWSNP225 | Ca-Kabuli-Chr1 | 2071687 | [C/T] | - | Intergenic | - |
| CWSNP226 | Ca-Kabuli-Chr1 | 2071645 | [C/T] | - | Intergenic | - |
| CWSNP227 | Ca-Kabuli-Chr1 | 2072613 | [G/A] | - | Intergenic | - |
| CWSNP228 | Ca-Kabuli-Chr1 | 2072627 | [A/T] | - | Intergenic | - |
| CWSNP229 | Ca-Kabuli-Chr1 | 2075087 | [A/T] | - | URR | - |
| CWSNP230 | Ca-Kabuli-Chr1 | 2075057 | [G/T] | - | URR | - |
| CWSNP231 | Ca-Kabuli-Chr1 | 2075045 | [C/A] | - | URR | - |
| CWSNP232 | Ca-Kabuli-Chr1 | 2075044 | [A/G] | - | URR | - |
| CWSNP233 | Ca-Kabuli-Chr1 | 2075043 | [C/A] | - | URR | - |
| CWSNP234 | Ca-Kabuli-Chr1 | 2075015 | [G/A] | - | URR | - |
| CWSNP235 | Ca-Kabuli-Chr1 | 2075013 | [A/G] | - | URR | - |
| CWSNP236 | Ca-Kabuli-Chr1 | 2075009 | [C/A] | - | URR | - |
| CWSNP237 | Ca-Kabuli-Chr1 | 2082760 | [C/G] | Ca00265 | Intron | Protein of unknown function DUF707 |
| CWSNP238 | Ca-Kabuli-Chr1 | 2082801 | [G/A] | Ca00265 | Intron | Protein of unknown function DUF707 |
| CWSNP239 | Ca-Kabuli-Chr1 | 2083003 | [G/C] | Ca00265 | Intron | Protein of unknown function DUF707 |
| CWSNP240 | Ca-Kabuli-Chr1 | 2083059 | [A/T] | Ca00265 | Intron | Protein of unknown function DUF707 |

| SNP IDs | Chromosomes | Physical positions (bp) | SNPs | Gene accession IDs | Sequence components of genes | Putative functions |
|---|---|---|---|---|---|---|
| CWSNP241 | Ca-Kabuli-Chr1 | 2126267 | [G/A] | Ca00268 | Intron | Protein kinase, catalytic domain |
| CWSNP242 | Ca-Kabuli-Chr1 | 2148583 | [T/G] | - | Intergenic | - |
| CWSNP243 | Ca-Kabuli-Chr1 | 2170090 | [G/C] | - | Intergenic | - |
| CWSNP244 | Ca-Kabuli-Chr1 | 2170935 | [A/C] | - | Intergenic | - |
| CWSNP245 | Ca-Kabuli-Chr1 | 2172076 | [T/C] | - | Intergenic | - |
| CWSNP246 | Ca-Kabuli-Chr1 | 2172113 | [G/A] | - | Intergenic | - |
| CWSNP247 | Ca-Kabuli-Chr1 | 2172143 | [T/G] | - | Intergenic | - |
| CWSNP248 | Ca-Kabuli-Chr1 | 2183544 | [A/G] | Ca00274 | Intron | SNF2-related |
| CWSNP249 | Ca-Kabuli-Chr1 | 2232779 | [A/G] | Ca00279 | Intron | Amidohydrolase1 |
| CWSNP250 | Ca-Kabuli-Chr1 | 2252270 | [C/T] | Ca00281 | Intron | Zinc finger,RING-type |
| CWSNP251 | Ca-Kabuli-Chr1 | 2252343 | [G/T] | Ca00281 | Intron | Zinc finger,RING-type |
| CWSNP252 | Ca-Kabuli-Chr1 | 2252513 | [C/T] | Ca00281 | Intron | Zinc finger,RING-type |
| CWSNP253 | Ca-Kabuli-Chr1 | 2269319 | [T/G] | - | DRR | - |
| CWSNP254 | Ca-Kabuli-Chr1 | 2269489 | [A/C] | - | DRR | - |
| CWSNP255 | Ca-Kabuli-Chr1 | 2277459 | [G/A] | Ca00285 | Intron | RNA recognition motif domain |
| CWSNP256 | Ca-Kabuli-Chr1 | 2285879 | [G/A] | - | Intergenic | - |

| SNP IDs | Chromosomes | Physical positions (bp) | SNPs | Gene accession IDs | Sequence components of genes | Putative functions |
|---|---|---|---|---|---|---|
| CWSNP257 | Ca-Kabuli-Chr1 | 2287408 | [A/T] | Ca00287 | Non-Synonymous-CDS | - |
| CWSNP258 | Ca-Kabuli-Chr1 | 2366413 | [T/C] | - | URR | - |
| CWSNP259 | Ca-Kabuli-Chr1 | 2366409 | [T/A] | - | URR | - |
| CWSNP260 | Ca-Kabuli-Chr1 | 2373862 | [G/A] | Ca00301 | Intron | Ribosomal RNA methyltransferase RrmJ/FtsJ |
| CWSNP261 | Ca-Kabuli-Chr1 | 2373910 | [C/T] | Ca00301 | Intron | Ribosomal RNA methyltransferase RrmJ/FtsJ |
| CWSNP262 | Ca-Kabuli-Chr1 | 2373954 | [G/T] | Ca00301 | Intron | Ribosomal RNA methyltransferase RrmJ/FtsJ |
| CWSNP263 | Ca-Kabuli-Chr1 | 2400799 | [T/G] | - | Intergenic | - |
| CWSNP264 | Ca-Kabuli-Chr1 | 2428531 | [A/G] | - | Intergenic | - |
| CWSNP265 | Ca-Kabuli-Chr1 | 2439915 | [A/G] | - | Intergenic | - |
| CWSNP266 | Ca-Kabuli-Chr1 | 2439917 | [G/A] | - | Intergenic | - |
| CWSNP267 | Ca-Kabuli-Chr1 | 2473309 | [T/C] | - | DRR | - |
| CWSNP268 | Ca-Kabuli-Chr1 | 2513516 | [T/A] | Ca00314 | Non-Synonymous-CDS | Telomerase activating protein Est1 |
| CWSNP269 | Ca-Kabuli-Chr1 | 2513535 | [T/C] | Ca00314 | Non-Synonymous-CDS | Telomerase activating protein Est2 |
| CWSNP270 | Ca-Kabuli-Chr1 | 2513737 | [T/C] | Ca00314 | Synonymous-CDS | Telomerase activating protein Est3 |
| CWSNP271 | Ca-Kabuli-Chr1 | 2513860 | [G/A] | Ca00314 | Synonymous-CDS | Telomerase activating protein Est6 |
| CWSNP272 | Ca-Kabuli-Chr1 | 2513854 | [G/A] | Ca00314 | Synonymous-CDS | Telomerase activating protein Est5 |

| SNP IDs | Chromosomes | Physical positions (bp) | SNPs | Gene accession IDs | Sequence components of genes | Putative functions |
|---|---|---|---|---|---|---|
| CWSNP273 | Ca-Kabuli-Chr1 | 2513792 | [A/G] | Ca00314 | Non-Synonymous-CDS | Telomerase activating protein Est4 |
| CWSNP274 | Ca-Kabuli-Chr1 | 2542652 | [G/A] | - | DRR | - |
| CWSNP275 | Ca-Kabuli-Chr1 | 2649864 | [C/A] | Ca00326 | Synonymous-CDS | Pathogenesis-related transcriptional factor/ERF, DNA-binding |
| CWSNP276 | Ca-Kabuli-Chr1 | 2649974 | [A/C] | Ca00326 | Synonymous-CDS | Pathogenesis-related transcriptional factor/ERF, DNA-binding |
| CWSNP277 | Ca-Kabuli-Chr1 | 2671403 | [G/T] | Ca00327 | Synonymous-CDS | Pathogenesis-related transcriptional factor/ERF, DNA-binding |
| CWSNP278 | Ca-Kabuli-Chr1 | 2745499 | [C/A] | - | URR | - |
| CWSNP279 | Ca-Kabuli-Chr1 | 2745514 | [A/C] | - | Intergenic | - |
| CWSNP280 | Ca-Kabuli-Chr1 | 2745519 | [G/C] | - | Intergenic | - |
| CWSNP281 | Ca-Kabuli-Chr1 | 2745486 | [C/T] | - | URR | - |
| CWSNP282 | Ca-Kabuli-Chr1 | 2754998 | [C/T] | Ca00340 | Synonymous-CDS | Disease resistance protein |
| CWSNP283 | Ca-Kabuli-Chr1 | 2755012 | [C/T] | Ca00340 | Synonymous-CDS | Disease resistance protein |
| CWSNP284 | Ca-Kabuli-Chr1 | 2755015 | [T/C] | Ca00340 | Synonymous-CDS | Disease resistance protein |
| CWSNP285 | Ca-Kabuli-Chr1 | 2755443 | [T/A] | Ca00340 | Non-Synonymous-CDS | Disease resistance protein |
| CWSNP286 | Ca-Kabuli-Chr1 | 2755455 | [G/A] | Ca00340 | Synonymous-CDS | Disease resistance protein |
| CWSNP287 | Ca-Kabuli-Chr1 | 2755457 | [C/T] | Ca00340 | Non-Synonymous-CDS | Disease resistance protein |
| CWSNP288 | Ca-Kabuli-Chr1 | 2755459 | [T/G] | Ca00340 | Synonymous-CDS | Disease resistance protein |

| SNP IDs | Chromosomes | Physical positions (bp) | SNPs | Gene accession IDs | Sequence components of genes | Putative functions |
|---|---|---|---|---|---|---|
| CWSNP289 | Ca-Kabuli-Chr1 | 2755465 | [T/A] | Ca00340 | Synonymous-CDS | Disease resistance protein |
| CWSNP290 | Ca-Kabuli-Chr1 | 2755466 | [C/G] | Ca00340 | Synonymous-CDS | Disease resistance protein |
| CWSNP291 | Ca-Kabuli-Chr1 | 2755470 | [A/T] | Ca00340 | Synonymous-CDS | Disease resistance protein |
| CWSNP292 | Ca-Kabuli-Chr1 | 2755471 | [C/A] | Ca00340 | Synonymous-CDS | Disease resistance protein |
| CWSNP293 | Ca-Kabuli-Chr1 | 2755489 | [G/A] | Ca00340 | Non-Synonymous-CDS | Disease resistance protein |
| CWSNP294 | Ca-Kabuli-Chr1 | 2755494 | [T/C] | Ca00340 | Synonymous-CDS | Disease resistance protein |
| CWSNP295 | Ca-Kabuli-Chr1 | 2755496 | [A/G] | Ca00340 | Non-Synonymous-CDS | Disease resistance protein |
| CWSNP296 | Ca-Kabuli-Chr1 | 2755613 | [G/T] | Ca00340 | Non-Synonymous-CDS | Disease resistance protein |
| CWSNP297 | Ca-Kabuli-Chr1 | 2755604 | [A/C] | Ca00340 | Non-Synonymous-CDS | Disease resistance protein |
| CWSNP298 | Ca-Kabuli-Chr1 | 2755596 | [C/A] | Ca00340 | Non-Synonymous-CDS | Disease resistance protein |
| CWSNP299 | Ca-Kabuli-Chr1 | 2757711 | [T/G] | Ca00340 | Synonymous-CDS | Disease resistance protein |
| CWSNP300 | Ca-Kabuli-Chr1 | 2757696 | [A/G] | Ca00340 | Synonymous-CDS | Disease resistance protein |
| CWSNP301 | Ca-Kabuli-Chr1 | 2757693 | [C/T] | Ca00340 | Synonymous-CDS | Disease resistance protein |
| CWSNP302 | Ca-Kabuli-Chr1 | 2757678 | [A/G] | Ca00340 | Synonymous-CDS | Disease resistance protein |
| CWSNP303 | Ca-Kabuli-Chr1 | 2758781 | [G/C] | Ca00340 | Non-Synonymous-CDS | Disease resistance protein |
| CWSNP304 | Ca-Kabuli-Chr1 | 2758759 | [G/C] | Ca00340 | Non-Synonymous-CDS | Disease resistance protein |

| SNP IDs | Chromosomes | Physical positions (bp) | SNPs | Gene accession IDs | Sequence components of genes | Putative functions |
|---|---|---|---|---|---|---|
| CWSNP305 | Ca-Kabuli-Chr1 | 2758750 | [A/G] | Ca00340 | Non-Synonymous-CDS | Disease resistance protein |
| CWSNP306 | Ca-Kabuli-Chr1 | 2758746 | [G/A] | Ca00340 | Synonymous-CDS | Disease resistance protein |
| CWSNP307 | Ca-Kabuli-Chr1 | 2758739 | [A/T] | Ca00340 | Non-Synonymous-CDS | Disease resistance protein |
| CWSNP308 | Ca-Kabuli-Chr1 | 2758738 | [T/G] | Ca00340 | Non-Synonymous-CDS | Disease resistance protein |
| CWSNP309 | Ca-Kabuli-Chr1 | 2758859 | [A/T] | - | Intergenic | - |
| CWSNP310 | Ca-Kabuli-Chr1 | 2758835 | [T/A] | - | Intergenic | - |
| CWSNP311 | Ca-Kabuli-Chr1 | 2758827 | [A/T] | Ca00340 | Non-Synonymous-CDS | Disease resistance protein |
| CWSNP312 | Ca-Kabuli-Chr1 | 2786994 | [A/C] | - | DRR | - |
| CWSNP313 | Ca-Kabuli-Chr1 | 2786989 | [T/C] | - | DRR | - |
| CWSNP314 | Ca-Kabuli-Chr1 | 2786976 | [A/C] | - | DRR | - |
| CWSNP315 | Ca-Kabuli-Chr1 | 2786974 | [T/C] | - | DRR | - |
| CWSNP316 | Ca-Kabuli-Chr1 | 2786970 | [T/A] | - | DRR | - |
| CWSNP317 | Ca-Kabuli-Chr1 | 2807605 | [G/C] | Ca00345 | Non-Synonymous-CDS | - |
| CWSNP318 | Ca-Kabuli-Chr1 | 2807616 | [G/A] | Ca00345 | Non-Synonymous-CDS | - |
| CWSNP319 | Ca-Kabuli-Chr1 | 2832792 | [G/A] | - | Intergenic | - |
| CWSNP320 | Ca-Kabuli-Chr1 | 2832969 | [G/A] | Ca00347 | Synonymous-CDS | Transcriptional factor B3 |

| SNP IDs | Chromosomes | Physical positions (bp) | SNPs | Gene accession IDs | Sequence components of genes | Putative functions |
|---|---|---|---|---|---|---|
| CWSNP321 | Ca-Kabuli-Chr1 | 2832939 | [G/T] | Ca00347 | Non-Synonymous-CDS | Transcriptional factor B3 |
| CWSNP322 | Ca-Kabuli-Chr1 | 2833448 | [C/A] | Ca00347 | Non-Synonymous-CDS | Transcriptional factor B3 |
| CWSNP323 | Ca-Kabuli-Chr1 | 2855927 | [T/A] | - | Intergenic | - |
| CWSNP324 | Ca-Kabuli-Chr1 | 2856003 | [T/C] | - | Intergenic | - |
| CWSNP325 | Ca-Kabuli-Chr1 | 2856045 | [C/T] | - | Intergenic | - |
| CWSNP326 | Ca-Kabuli-Chr1 | 2856079 | [T/G] | - | Intergenic | - |
| CWSNP327 | Ca-Kabuli-Chr1 | 2856094 | [T/G] | - | Intergenic | - |
| CWSNP328 | Ca-Kabuli-Chr1 | 2856120 | [T/G] | - | Intergenic | - |
| CWSNP329 | Ca-Kabuli-Chr1 | 2856534 | [T/A] | - | Intergenic | - |
| CWSNP330 | Ca-Kabuli-Chr1 | 2858263 | [A/G] | - | Intergenic | - |
| CWSNP331 | Ca-Kabuli-Chr1 | 2858299 | [C/A] | - | Intergenic | - |
| CWSNP332 | Ca-Kabuli-Chr1 | 2868942 | [G/A] | - | DRR | - |
| CWSNP333 | Ca-Kabuli-Chr1 | 2881422 | [T/C] | Ca00351 | Synonymous-CDS | Disease resistance protein |
| CWSNP334 | Ca-Kabuli-Chr1 | 2881389 | [G/C] | Ca00351 | Non-Synonymous-CDS | Disease resistance protein |
| CWSNP335 | Ca-Kabuli-Chr1 | 2881386 | [G/T] | Ca00351 | Non-Synonymous-CDS | Disease resistance protein |
| CWSNP336 | Ca-Kabuli-Chr1 | 2883625 | [T/A] | Ca00351 | Non-Synonymous-CDS | Disease resistance protein |

| SNP IDs | Chromosomes | Physical positions (bp) | SNPs | Gene accession IDs | Sequence components of genes | Putative functions |
|---|---|---|---|---|---|---|
| CWSNP337 | Ca-Kabuli-Chr1 | 2883634 | [T/A] | - | Intergenic | - |
| CWSNP338 | Ca-Kabuli-Chr1 | 2883635 | [A/G] | - | Intergenic | - |
| CWSNP339 | Ca-Kabuli-Chr1 | 2883660 | [C/G] | - | Intergenic | - |
| CWSNP340 | Ca-Kabuli-Chr1 | 2883666 | [G/A] | - | Intergenic | - |
| CWSNP341 | Ca-Kabuli-Chr1 | 2883684 | [G/T] | - | Intergenic | - |
| CWSNP342 | Ca-Kabuli-Chr1 | 2883717 | [C/A] | - | Intergenic | - |
| CWSNP343 | Ca-Kabuli-Chr1 | 2883716 | [A/C] | - | Intergenic | - |
| CWSNP344 | Ca-Kabuli-Chr1 | 2885508 | [T/C] | - | Intergenic | - |
| CWSNP345 | Ca-Kabuli-Chr1 | 2885525 | [T/C] | - | Intergenic | - |
| CWSNP346 | Ca-Kabuli-Chr1 | 2885531 | [C/T] | - | Intergenic | - |
| CWSNP347 | Ca-Kabuli-Chr1 | 2885626 | [A/G] | - | Intergenic | - |
| CWSNP348 | Ca-Kabuli-Chr1 | 2885673 | [G/C] | - | DRR | - |
| CWSNP349 | Ca-Kabuli-Chr1 | 2885892 | [T/A] | - | DRR | - |
| CWSNP350 | Ca-Kabuli-Chr1 | 2885923 | [C/G] | - | DRR | - |
| CWSNP351 | Ca-Kabuli-Chr1 | 2886025 | [A/G] | - | DRR | - |
| CWSNP352 | Ca-Kabuli-Chr1 | 2886005 | [G/A] | - | DRR | - |

| SNP IDs | Chromosomes | Physical positions (bp) | SNPs | Gene accession IDs | Sequence components of genes | Putative functions |
|---|---|---|---|---|---|---|
| CWSNP353 | Ca-Kabuli-Chr1 | 2885997 | [G/A] | - | DRR | - |
| CWSNP354 | Ca-Kabuli-Chr1 | 2885988 | [C/G] | - | DRR | - |
| CWSNP355 | Ca-Kabuli-Chr1 | 2885987 | [T/A] | - | DRR | - |
| CWSNP356 | Ca-Kabuli-Chr1 | 2948211 | [C/T] | - | DRR | - |
| CWSNP357 | Ca-Kabuli-Chr1 | 3032061 | [C/A] | - | DRR | - |
| CWSNP358 | Ca-Kabuli-Chr1 | 3081851 | [T/G] | Ca00376 | Intron | Lipase,GDSL |
| CWSNP359 | Ca-Kabuli-Chr1 | 3081850 | [C/A] | Ca00376 | Intron | Lipase,GDSL |
| CWSNP360 | Ca-Kabuli-Chr1 | 3082280 | [G/A] | Ca00376 | Synonymous-CDS | Lipase,GDSL |
| CWSNP361 | Ca-Kabuli-Chr1 | 3151007 | [G/A] | - | Intergenic | - |
| CWSNP362 | Ca-Kabuli-Chr1 | 3150986 | [T/C] | - | Intergenic | - |
| CWSNP363 | Ca-Kabuli-Chr1 | 3150964 | [C/G] | - | Intergenic | - |
| CWSNP364 | Ca-Kabuli-Chr1 | 3298327 | [C/G] | - | URR | - |
| CWSNP365 | Ca-Kabuli-Chr1 | 3364952 | [A/C] | - | DRR | - |
| CWSNP366 | Ca-Kabuli-Chr1 | 3503059 | [C/A] | Ca00425 | Non-Synonymous-CDS | Armadillo |
| CWSNP367 | Ca-Kabuli-Chr1 | 3542263 | [A/C] | - | DRR | - |
| CWSNP368 | Ca-Kabuli-Chr1 | 3611231 | [C/A] | - | DRR | - |

| SNP IDs | Chromosomes | Physical positions (bp) | SNPs | Gene accession IDs | Sequence components of genes | Putative functions |
|---|---|---|---|---|---|---|
| CWSNP369 | Ca-Kabuli-Chr1 | 3711498 | [T/G] | - | DRR | - |
| CWSNP370 | Ca-Kabuli-Chr1 | 3711525 | [T/G] | - | DRR | - |
| CWSNP371 | Ca-Kabuli-Chr1 | 3852035 | [A/C] | Ca00467 | Intron | AUX/IAA protein |
| CWSNP372 | Ca-Kabuli-Chr1 | 3852476 | [A/C] | Ca00467 | Intron | AUX/IAA protein |
| CWSNP373 | Ca-Kabuli-Chr1 | 4073941 | [A/C] | - | Intergenic | - |
| CWSNP374 | Ca-Kabuli-Chr1 | 4189333 | [C/T] | - | DRR | - |
| CWSNP375 | Ca-Kabuli-Chr1 | 4245058 | [C/G] | - | Intergenic | - |
| CWSNP376 | Ca-Kabuli-Chr1 | 4245073 | [C/G] | - | Intergenic | - |
| CWSNP377 | Ca-Kabuli-Chr1 | 4245176 | [G/T] | - | Intergenic | - |
| CWSNP378 | Ca-Kabuli-Chr1 | 4292981 | [G/C] | - | Intergenic | - |
| CWSNP379 | Ca-Kabuli-Chr1 | 4302669 | [G/C] | - | URR | - |
| CWSNP380 | Ca-Kabuli-Chr1 | 4302770 | [A/C] | - | URR | - |
| CWSNP381 | Ca-Kabuli-Chr1 | 4302751 | [T/C] | - | URR | - |
| CWSNP382 | Ca-Kabuli-Chr1 | 4334299 | [A/T] | Ca00517 | Intron | Tetratricopeptide repeat-containing |
| CWSNP383 | Ca-Kabuli-Chr1 | 4334336 | [A/G] | Ca00517 | Intron | Tetratricopeptide repeat-containing |
| CWSNP384 | Ca-Kabuli-Chr1 | 4375710 | [T/C] | Ca00522 | Synonymous-CDS | Protein kinase, catalytic domain |

| SNP IDs | Chromosomes | Physical positions (bp) | SNPs | Gene accession IDs | Sequence components of genes | Putative functions |
|---|---|---|---|---|---|---|
| CWSNP385 | Ca-Kabuli-Chr1 | 4386114 | [T/G] | Ca00523 | Intron | SNF2-related |
| CWSNP386 | Ca-Kabuli-Chr1 | 4386111 | [T/C] | Ca00523 | Intron | SNF2-related |
| CWSNP387 | Ca-Kabuli-Chr1 | 4392680 | [A/G] | Ca00524 | Intron | Lipase,class3 |
| CWSNP388 | Ca-Kabuli-Chr1 | 4414410 | [A/T] | - | DRR | - |
| CWSNP389 | Ca-Kabuli-Chr1 | 4429044 | [C/T] | Ca00530 | Non-Synonymous-CDS | DNA-directed RNA polymerase, subunit 2,domain 6 |
| CWSNP390 | Ca-Kabuli-Chr1 | 4456662 | [G/A] | Ca00531 | Non-Synonymous-CDS | DNA-directed RNA polymerase, subunit 2,domain 6 |
| CWSNP391 | Ca-Kabuli-Chr1 | 4456654 | [A/C] | Ca00531 | Synonymous-CDS | DNA-directed RNA polymerase, subunit 2,domain 6 |
| CWSNP392 | Ca-Kabuli-Chr1 | 4456648 | [T/C] | Ca00531 | Non-Synonymous-CDS | DNA-directed RNA polymerase, subunit 2,domain 6 |
| CWSNP393 | Ca-Kabuli-Chr1 | 4457588 | [A/G] | Ca00531 | Intron | DNA-directed RNA polymerase, subunit 2,domain 6 |
| CWSNP394 | Ca-Kabuli-Chr1 | 4460112 | [A/T] | Ca00531 | Intron | DNA-directed RNA polymerase, subunit 2,domain 6 |
| CWSNP395 | Ca-Kabuli-Chr1 | 4494270 | [C/T] | Ca00534 | Synonymous-CDS | Protein kinase, catalytic domain |
| CWSNP396 | Ca-Kabuli-Chr1 | 4494467 | [A/G] | Ca00534 | Intron | Protein kinase, catalytic domain |
| CWSNP397 | Ca-Kabuli-Chr1 | 4512566 | [T/C] | - | DRR | - |
| CWSNP398 | Ca-Kabuli-Chr1 | 4528117 | [A/G] | Ca00537 | Intron | - |
| CWSNP399 | Ca-Kabuli-Chr1 | 4528184 | [C/T] | Ca00537 | Intron | - |
| CWSNP400 | Ca-Kabuli-Chr1 | 4530304 | [T/C] | Ca00537 | Synonymous-CDS | - |

| SNP IDs | Chromosomes | Physical positions (bp) | SNPs | Gene accession IDs | Sequence components of genes | Putative functions |
|---|---|---|---|---|---|---|
| CWSNP401 | Ca-Kabuli-Chr1 | 4560792 | [C/T] | Ca00541 | Synonymous-CDS | Protein kinase, catalytic domain |
| CWSNP402 | Ca-Kabuli-Chr1 | 4560920 | [G/A] | Ca00541 | Non-Synonymous-CDS | Protein kinase, catalytic domain |
| CWSNP403 | Ca-Kabuli-Chr1 | 4571540 | [C/G] | - | Intergenic | - |
| CWSNP404 | Ca-Kabuli-Chr1 | 4571539 | [G/A] | - | Intergenic | - |
| CWSNP405 | Ca-Kabuli-Chr1 | 4576888 | [A/G] | Ca00543 | Non-Synonymous-CDS | BRCT |
| CWSNP406 | Ca-Kabuli-Chr1 | 4578967 | [T/G] | Ca00543 | Intron | BRCT |
| CWSNP407 | Ca-Kabuli-Chr1 | 4578999 | [T/A] | Ca00543 | Intron | BRCT |
| CWSNP408 | Ca-Kabuli-Chr1 | 4582189 | [C/T] | - | DRR | - |
| CWSNP409 | Ca-Kabuli-Chr1 | 4626099 | [T/G] | - | URR | - |
| CWSNP410 | Ca-Kabuli-Chr1 | 4631467 | [G/A] | - | URR | - |
| CWSNP411 | Ca-Kabuli-Chr1 | 4632188 | [G/A] | - | URR | - |
| CWSNP412 | Ca-Kabuli-Chr1 | 4632965 | [G/A] | - | URR | - |
| CWSNP413 | Ca-Kabuli-Chr1 | 4634413 | [C/T] | Ca00548 | Synonymous-CDS | Dehydrogenase,E1component |
| CWSNP414 | Ca-Kabuli-Chr1 | 4662564 | [A/G] | Ca00550 | Intron | Transcription factor,SBP-box |
| CWSNP415 | Ca-Kabuli-Chr1 | 4672439 | [G/A] | Ca00551 | Non-Synonymous-CDS | Sugar/inositol transporter |
| CWSNP416 | Ca-Kabuli-Chr1 | 4715563 | [T/C] | Ca00555 | Non-Synonymous-CDS | Protein kinase, catalytic domain |

| SNP IDs | Chromosomes | Physical positions (bp) | SNPs | Gene accession IDs | Sequence components of genes | Putative functions |
|---|---|---|---|---|---|---|
| CWSNP417 | Ca-Kabuli-Chr1 | 4780345 | [G/T] | Ca00562 | Non-Synonymous-CDS | Zinc finger,RING-type |
| CWSNP418 | Ca-Kabuli-Chr1 | 4781362 | [C/T] | Ca00562 | Synonymous-CDS | Zinc finger,RING-type |
| CWSNP419 | Ca-Kabuli-Chr1 | 4781416 | [G/A] | Ca00562 | Synonymous-CDS | Zinc finger,RING-type |
| CWSNP420 | Ca-Kabuli-Chr1 | 4781704 | [G/A] | Ca00562 | Synonymous-CDS | Zinc finger,RING-type |
| CWSNP421 | Ca-Kabuli-Chr1 | 4808910 | [A/T] | Ca00566 | Intron | SAC3/GANP/Nin1/mts3/eIF-3p25 |
| CWSNP422 | Ca-Kabuli-Chr1 | 4878683 | [T/C] | - | Intergenic | - |
| CWSNP423 | Ca-Kabuli-Chr1 | 4878691 | [A/C] | - | Intergenic | - |
| CWSNP424 | Ca-Kabuli-Chr1 | 4878695 | [T/C] | - | Intergenic | - |
| CWSNP425 | Ca-Kabuli-Chr1 | 4878823 | [A/G] | - | Intergenic | - |
| CWSNP426 | Ca-Kabuli-Chr1 | 4903185 | [T/A] | - | DRR | - |
| CWSNP427 | Ca-Kabuli-Chr1 | 4906816 | [A/G] | - | Intergenic | - |
| CWSNP428 | Ca-Kabuli-Chr1 | 4906859 | [T/C] | - | Intergenic | - |
| CWSNP429 | Ca-Kabuli-Chr1 | 4906854 | [A/G] | - | Intergenic | - |
| CWSNP430 | Ca-Kabuli-Chr1 | 4909286 | [T/G] | Ca00577 | Synonymous-CDS | Disease resistance protein |
| CWSNP431 | Ca-Kabuli-Chr1 | 4909271 | [A/G] | Ca00577 | Synonymous-CDS | Disease resistance protein |
| CWSNP432 | Ca-Kabuli-Chr1 | 4909268 | [C/T] | Ca00577 | Synonymous-CDS | Disease resistance protein |

| SNP IDs | Chromosomes | Physical positions (bp) | SNPs | Gene accession IDs | Sequence components of genes | Putative functions |
|---|---|---|---|---|---|---|
| CWSNP433 | Ca-Kabuli-Chr1 | 4909253 | [A/G] | Ca00577 | Synonymous-CDS | Disease resistance protein |
| CWSNP434 | Ca-Kabuli-Chr1 | 4910497 | [A/G] | Ca00577 | Synonymous-CDS | Disease resistance protein |
| CWSNP435 | Ca-Kabuli-Chr1 | 4910494 | [C/A] | Ca00577 | Synonymous-CDS | Disease resistance protein |
| CWSNP436 | Ca-Kabuli-Chr1 | 4948740 | [A/T] | - | Intergenic | - |
| CWSNP437 | Ca-Kabuli-Chr1 | 4949578 | [A/T] | - | DRR | - |
| CWSNP438 | Ca-Kabuli-Chr1 | 4950726 | [G/T] | - | DRR | - |
| CWSNP439 | Ca-Kabuli-Chr1 | 4959562 | [A/C] | - | URR | - |
| CWSNP440 | Ca-Kabuli-Chr1 | 4996404 | [A/C] | Ca00587 | Synonymous-CDS | - |
| CWSNP441 | Ca-Kabuli-Chr1 | 4996459 | [T/C] | Ca00587 | Synonymous-CDS | - |
| CWSNP442 | Ca-Kabuli-Chr1 | 4996476 | [G/A] | Ca00587 | Synonymous-CDS | - |
| CWSNP443 | Ca-Kabuli-Chr1 | 4996536 | [C/T] | Ca00587 | Synonymous-CDS | - |
| CWSNP444 | Ca-Kabuli-Chr1 | 5071158 | [G/A] | Ca00593 | Intron | RNA recognition motif domain |
| CWSNP445 | Ca-Kabuli-Chr1 | 5072022 | [C/A] | Ca00593 | Synonymous-CDS | RNA recognition motif domain |
| CWSNP446 | Ca-Kabuli-Chr1 | 5080358 | [G/A] | - | URR | - |
| CWSNP447 | Ca-Kabuli-Chr1 | 5087428 | [T/C] | - | DRR | - |
| CWSNP448 | Ca-Kabuli-Chr1 | 5122663 | [A/G] | - | Intergenic | - |

| SNP IDs | Chromosomes | Physical positions (bp) | SNPs | Gene accession IDs | Sequence components of genes | Putative functions |
|---|---|---|---|---|---|---|
| CWSNP449 | Ca-Kabuli-Chr1 | 5140801 | [C/A] | - | Intergenic | - |
| CWSNP450 | Ca-Kabuli-Chr1 | 5141009 | [G/C] | - | Intergenic | - |
| CWSNP451 | Ca-Kabuli-Chr1 | 5146658 | [C/A] | - | Intergenic | - |
| CWSNP452 | Ca-Kabuli-Chr1 | 5146696 | [T/A] | - | Intergenic | - |
| CWSNP453 | Ca-Kabuli-Chr1 | 5164127 | [A/G] | - | Intergenic | - |
| CWSNP454 | Ca-Kabuli-Chr1 | 5164136 | [C/T] | - | Intergenic | - |
| CWSNP455 | Ca-Kabuli-Chr1 | 5164198 | [C/A] | - | Intergenic | - |
| CWSNP456 | Ca-Kabuli-Chr1 | 5197461 | [C/T] | Ca00608 | Intron | Uncharacterised protein family,carbohydratekinase-related |
| CWSNP457 | Ca-Kabuli-Chr1 | 5197475 | [T/A] | Ca00608 | Intron | Uncharacterised protein family,carbohydratekinase-related |
| CWSNP458 | Ca-Kabuli-Chr1 | 5200250 | [T/G] | Ca00608 | Intron | Uncharacterised protein family,carbohydratekinase-related |
| CWSNP459 | Ca-Kabuli-Chr1 | 5200289 | [G/T] | Ca00608 | Intron | Uncharacterised protein family,carbohydratekinase-related |
| CWSNP460 | Ca-Kabuli-Chr1 | 5249839 | [G/A] | Ca00613 | Non-Synonymous-CDS | Protein of unknown function DUF584 |
| CWSNP461 | Ca-Kabuli-Chr1 | 5249846 | [A/C] | Ca00613 | Synonymous-CDS | Protein of unknown function DUF585 |
| CWSNP462 | Ca-Kabuli-Chr1 | 5249868 | [G/A] | Ca00613 | Synonymous-CDS | Protein of unknown function DUF586 |
| CWSNP463 | Ca-Kabuli-Chr1 | 5288421 | [A/C] | Ca00616 | Synonymous-CDS | Heavy metal transport/detoxification protein |
| CWSNP464 | Ca-Kabuli-Chr1 | 5321398 | [A/G] | - | Intergenic | - |

| SNP IDs | Chromosomes | Physical positions (bp) | SNPs | Gene accession IDs | Sequence components of genes | Putative functions |
|---|---|---|---|---|---|---|
| CWSNP465 | Ca-Kabuli-Chr1 | 5430700 | [A/C] | Ca00628 | Synonymous-CDS | Protein of unknown function DUF584 |
| CWSNP466 | Ca-Kabuli-Chr1 | 5430703 | [T/G] | Ca00628 | Synonymous-CDS | Protein of unknown function DUF584 |
| CWSNP467 | Ca-Kabuli-Chr1 | 5430809 | [G/A] | Ca00628 | Non-Synonymous-CDS | Protein of unknown function DUF584 |
| CWSNP468 | Ca-Kabuli-Chr1 | 5444130 | [A/C] | - | Intergenic | - |
| CWSNP469 | Ca-Kabuli-Chr1 | 5444142 | [A/T] | - | Intergenic | - |
| CWSNP470 | Ca-Kabuli-Chr1 | 5487535 | [A/G] | Ca00634 | Non-Synonymous-CDS | - |
| CWSNP471 | Ca-Kabuli-Chr1 | 5489806 | [C/G] | Ca00634 | Intron | - |
| CWSNP472 | Ca-Kabuli-Chr1 | 5637388 | [T/A] | - | URR | - |
| CWSNP473 | Ca-Kabuli-Chr1 | 5637442 | [C/T] | - | URR | - |
| CWSNP474 | Ca-Kabuli-Chr1 | 5666178 | [A/C] | Ca00656 | Synonymous-CDS | DNA-binding WRKY |
| CWSNP475 | Ca-Kabuli-Chr1 | 5666217 | [C/A] | Ca00656 | Non-Synonymous-CDS | DNA-binding WRKY |
| CWSNP476 | Ca-Kabuli-Chr1 | 5666260 | [T/G] | Ca00656 | Synonymous-CDS | DNA-binding WRKY |
| CWSNP477 | Ca-Kabuli-Chr1 | 5666266 | [T/G] | Ca00656 | Non-Synonymous-CDS | DNA-binding WRKY |
| CWSNP478 | Ca-Kabuli-Chr1 | 5666263 | [T/G] | Ca00656 | Synonymous-CDS | DNA-binding WRKY |
| CWSNP479 | Ca-Kabuli-Chr1 | 5666355 | [T/G] | Ca00656 | Non-Synonymous-CDS | DNA-binding WRKY |
| CWSNP480 | Ca-Kabuli-Chr1 | 5667605 | [G/A] | Ca00656 | Synonymous-CDS | DNA-binding WRKY |

| SNP IDs | Chromosomes | Physical positions (bp) | SNPs | Gene accession IDs | Sequence components of genes | Putative functions |
|---|---|---|---|---|---|---|
| CWSNP481 | Ca-Kabuli-Chr1 | 5706858 | [A/T] | - | Intergenic | - |
| CWSNP482 | Ca-Kabuli-Chr1 | 5706961 | [G/T] | - | Intergenic | - |
| CWSNP483 | Ca-Kabuli-Chr1 | 5706948 | [A/T] | - | Intergenic | - |
| CWSNP484 | Ca-Kabuli-Chr1 | 5706944 | [T/G] | - | Intergenic | - |
| CWSNP485 | Ca-Kabuli-Chr1 | 5706920 | [G/A] | - | Intergenic | - |
| CWSNP486 | Ca-Kabuli-Chr1 | 5721616 | [A/T] | Ca00664 | Intron | Protein kinase, catalytic domain |
| CWSNP487 | Ca-Kabuli-Chr1 | 5749456 | [C/T] | - | Intergenic | - |
| CWSNP488 | Ca-Kabuli-Chr1 | 5791118 | [T/G] | - | DRR | - |
| CWSNP489 | Ca-Kabuli-Chr1 | 5796146 | [C/T] | - | DRR | - |
| CWSNP490 | Ca-Kabuli-Chr1 | 5796886 | [A/G] | Ca00670 | Intron | UBX |
| CWSNP491 | Ca-Kabuli-Chr1 | 5807492 | [T/C] | - | Intergenic | - |
| CWSNP492 | Ca-Kabuli-Chr1 | 5812283 | [G/T] | - | URR | - |
| CWSNP493 | Ca-Kabuli-Chr1 | 5862565 | [C/T] | - | Intergenic | - |
| CWSNP494 | Ca-Kabuli-Chr1 | 6011697 | [T/G] | - | Intergenic | - |
| CWSNP495 | Ca-Kabuli-Chr1 | 6011689 | [G/A] | - | Intergenic | - |
| CWSNP496 | Ca-Kabuli-Chr1 | 6011761 | [C/T] | - | Intergenic | - |

| SNP IDs | Chromosomes | Physical positions (bp) | SNPs | Gene accession IDs | Sequence components of genes | Putative functions |
|---|---|---|---|---|---|---|
| CWSNP497 | Ca-Kabuli-Chr1 | 6066972 | [C/T] | Ca00693 | Intron | RHO protein GDP dissociation inhibitor |
| CWSNP498 | Ca-Kabuli-Chr1 | 6066936 | [A/G] | Ca00693 | Intron | RHO protein GDP dissociation inhibitor |
| CWSNP499 | Ca-Kabuli-Chr1 | 6066933 | [C/T] | Ca00693 | Intron | RHO protein GDP dissociation inhibitor |
| CWSNP500 | Ca-Kabuli-Chr1 | 6067846 | [T/C] | Ca00693 | Intron | RHO protein GDP dissociation inhibitor |
| CWSNP501 | Ca-Kabuli-Chr1 | 6088283 | [C/T] | - | Intergenic | - |
| CWSNP502 | Ca-Kabuli-Chr1 | 6126772 | [G/A] | Ca00697 | Intron | Ribosomal proteinL14b/L23e |
| CWSNP503 | Ca-Kabuli-Chr1 | 6257653 | [C/G] | Ca07885 | Intron | Thymidinekinase |
| CWSNP504 | Ca-Kabuli-Chr1 | 6257712 | [A/T] | Ca07885 | Intron | Thymidinekinase |
| CWSNP505 | Ca-Kabuli-Chr1 | 6262461 | [A/G] | - | DRR | - |
| CWSNP506 | Ca-Kabuli-Chr1 | 6262529 | [T/G] | - | DRR | - |
| CWSNP507 | Ca-Kabuli-Chr1 | 6262577 | [A/G] | - | DRR | - |
| CWSNP508 | Ca-Kabuli-Chr1 | 6278572 | [T/C] | - | Intergenic | - |
| CWSNP509 | Ca-Kabuli-Chr1 | 6278625 | [T/C] | - | Intergenic | - |
| CWSNP510 | Ca-Kabuli-Chr1 | 6279644 | [G/T] | - | Intergenic | - |
| CWSNP511 | Ca-Kabuli-Chr1 | 6296858 | [G/A] | Ca07890 | Intron | Uncharacterised protein familyUPF0497,trans-membraneplant |
| CWSNP512 | Ca-Kabuli-Chr1 | 6296986 | [C/A] | Ca07890 | Intron | Uncharacterised protein familyUPF0497,trans-membraneplant |

| SNP IDs | Chromosomes | Physical positions (bp) | SNPs | Gene accession IDs | Sequence components of genes | Putative functions |
|---|---|---|---|---|---|---|
| CWSNP513 | Ca-Kabuli-Chr1 | 6315337 | [G/T] | Ca07892 | Intron | Zinc finger,ZZ-type |
| CWSNP514 | Ca-Kabuli-Chr1 | 6315402 | [C/T] | Ca07892 | Intron | Zinc finger,ZZ-type |
| CWSNP515 | Ca-Kabuli-Chr1 | 6315399 | [T/G] | Ca07892 | Intron | Zinc finger,ZZ-type |
| CWSNP516 | Ca-Kabuli-Chr1 | 6315397 | [C/G] | Ca07892 | Intron | Zinc finger,ZZ-type |
| CWSNP517 | Ca-Kabuli-Chr1 | 6328801 | [T/A] | - | Intergenic | - |
| CWSNP518 | Ca-Kabuli-Chr1 | 6328957 | [C/T] | - | Intergenic | - |
| CWSNP519 | Ca-Kabuli-Chr1 | 6328963 | [A/G] | - | Intergenic | - |
| CWSNP520 | Ca-Kabuli-Chr1 | 6329814 | [T/A] | Ca07893 | Synonymous-CDS | Bromo adjacent homology (BAH) domain |
| CWSNP521 | Ca-Kabuli-Chr1 | 6329847 | [A/G] | Ca07893 | Synonymous-CDS | Bromo adjacent homology (BAH) domain |
| CWSNP522 | Ca-Kabuli-Chr1 | 6381191 | [T/C] | - | Intergenic | - |
| CWSNP523 | Ca-Kabuli-Chr1 | 6390541 | [T/A] | - | Intergenic | - |
| CWSNP524 | Ca-Kabuli-Chr1 | 6390849 | [G/A] | - | URR | - |
| CWSNP525 | Ca-Kabuli-Chr1 | 6391017 | [T/C] | - | URR | - |
| CWSNP526 | Ca-Kabuli-Chr1 | 6433592 | [T/G] | - | Intergenic | - |
| CWSNP527 | Ca-Kabuli-Chr1 | 6433826 | [G/A] | Ca07907 | Synonymous-CDS | NUDIX hydrolase domain |
| CWSNP528 | Ca-Kabuli-Chr1 | 6433838 | [C/T] | Ca07907 | Synonymous-CDS | NUDIX hydrolase domain |

| SNP IDs | Chromosomes | Physical positions (bp) | SNPs | Gene accession IDs | Sequence components of genes | Putative functions |
|---|---|---|---|---|---|---|
| CWSNP529 | Ca-Kabuli-Chr1 | 6433841 | [C/T] | Ca07907 | Synonymous-CDS | NUDIX hydrolase domain |
| CWSNP530 | Ca-Kabuli-Chr1 | 6433883 | [A/G] | Ca07907 | Synonymous-CDS | NUDIX hydrolase domain |
| CWSNP531 | Ca-Kabuli-Chr1 | 6433934 | [T/A] | Ca07907 | Synonymous-CDS | NUDIX hydrolase domain |
| CWSNP532 | Ca-Kabuli-Chr1 | 6433925 | [C/T] | Ca07907 | Synonymous-CDS | NUDIX hydrolase domain |
| CWSNP533 | Ca-Kabuli-Chr1 | 6478736 | [A/G] | - | Intergenic | - |
| CWSNP534 | Ca-Kabuli-Chr1 | 6478758 | [A/G] | - | Intergenic | - |
| CWSNP535 | Ca-Kabuli-Chr1 | 6486446 | [G/C] | Ca07911 | Intron | - |
| CWSNP536 | Ca-Kabuli-Chr1 | 6490978 | [A/T] | Ca07911 | Intron | - |
| CWSNP537 | Ca-Kabuli-Chr1 | 6491008 | [A/T] | Ca07911 | Intron | - |
| CWSNP538 | Ca-Kabuli-Chr1 | 6491030 | [T/G] | Ca07911 | Intron | - |
| CWSNP539 | Ca-Kabuli-Chr1 | 6491017 | [T/C] | Ca07911 | Intron | - |
| CWSNP540 | Ca-Kabuli-Chr1 | 6521670 | [C/T] | - | DRR | - |
| CWSNP541 | Ca-Kabuli-Chr1 | 6521673 | [G/A] | - | DRR | - |
| CWSNP542 | Ca-Kabuli-Chr1 | 6521856 | [A/G] | - | DRR | - |
| CWSNP543 | Ca-Kabuli-Chr1 | 6521859 | [T/A] | - | DRR | - |
| CWSNP544 | Ca-Kabuli-Chr1 | 6521970 | [C/T] | - | Intergenic | - |

| SNP IDs | Chromosomes | Physical positions (bp) | SNPs | Gene accession IDs | Sequence components of genes | Putative functions |
|---|---|---|---|---|---|---|
| CWSNP545 | Ca-Kabuli-Chr1 | 6540056 | [C/T] | - | Intergenic | - |
| CWSNP546 | Ca-Kabuli-Chr1 | 6540170 | [G/A] | - | Intergenic | - |
| CWSNP547 | Ca-Kabuli-Chr1 | 6553107 | [A/C] | Ca07920 | Intron | - |
| CWSNP548 | Ca-Kabuli-Chr1 | 6564716 | [C/T] | Ca07921 | Synonymous-CDS | Polyprenylsynthetase |
| CWSNP549 | Ca-Kabuli-Chr1 | 6591506 | [G/C] | - | DRR | - |
| CWSNP550 | Ca-Kabuli-Chr1 | 6599917 | [C/A] | - | Intergenic | - |
| CWSNP551 | Ca-Kabuli-Chr1 | 6634482 | [G/C] | - | DRR | - |
| CWSNP552 | Ca-Kabuli-Chr1 | 6635366 | [T/C] | - | DRR | - |
| CWSNP553 | Ca-Kabuli-Chr1 | 6679253 | [A/G] | Ca07932 | Synonymous-CDS | WD40 repeat |
| CWSNP554 | Ca-Kabuli-Chr1 | 6679329 | [A/G] | Ca07932 | Non-Synonymous-CDS | WD40 repeat |
| CWSNP555 | Ca-Kabuli-Chr1 | 6803364 | [G/A] | - | URR | - |
| CWSNP556 | Ca-Kabuli-Chr1 | 6804110 | [A/T] | - | URR | - |
| CWSNP557 | Ca-Kabuli-Chr1 | 6849437 | [C/T] | Ca07951 | Intron | Vps53-like,N-terminal |
| CWSNP558 | Ca-Kabuli-Chr1 | 6849512 | [G/C] | Ca07951 | Intron | Vps53-like,N-terminal |
| CWSNP559 | Ca-Kabuli-Chr1 | 6880837 | [A/G] | - | URR | - |
| CWSNP560 | Ca-Kabuli-Chr1 | 6904278 | [A/C] | Ca07956 | Non-Synonymous-CDS | Protein kinase, catalytic domain |

| SNP IDs | Chromosomes | Physical positions (bp) | SNPs | Gene accession IDs | Sequence components of genes | Putative functions |
|---|---|---|---|---|---|---|
| CWSNP561 | Ca-Kabuli-Chr1 | 6904338 | [C/T] | Ca07956 | Intron | Protein kinase, catalytic domain |
| CWSNP562 | Ca-Kabuli-Chr1 | 7050825 | [G/A] | - | Intergenic | - |
| CWSNP563 | Ca-Kabuli-Chr1 | 7050958 | [C/T] | - | Intergenic | - |
| CWSNP564 | Ca-Kabuli-Chr1 | 7050953 | [C/T] | - | Intergenic | - |
| CWSNP565 | Ca-Kabuli-Chr1 | 7104985 | [G/T] | - | Intergenic | - |
| CWSNP566 | Ca-Kabuli-Chr1 | 7127843 | [A/G] | Ca07973 | Synonymous-CDS | Sec8 exocystcomplex component specific domain |
| CWSNP567 | Ca-Kabuli-Chr1 | 7136677 | [T/C] | - | Intergenic | - |
| CWSNP568 | Ca-Kabuli-Chr1 | 7147651 | [T/A] | Ca07974 | Intron | - |
| CWSNP569 | Ca-Kabuli-Chr1 | 7155344 | [A/G] | Ca07974 | Intron | - |
| CWSNP570 | Ca-Kabuli-Chr1 | 7157057 | [A/C] | Ca07974 | Intron | - |
| CWSNP571 | Ca-Kabuli-Chr1 | 7157112 | [T/C] | Ca07974 | Intron | - |
| CWSNP572 | Ca-Kabuli-Chr1 | 7160649 | [T/C] | Ca07974 | Intron | - |
| CWSNP573 | Ca-Kabuli-Chr1 | 7160695 | [G/A] | Ca07974 | Intron | - |
| CWSNP574 | Ca-Kabuli-Chr1 | 7160754 | [A/T] | Ca07974 | Intron | - |
| CWSNP575 | Ca-Kabuli-Chr1 | 7160781 | [A/C] | Ca07974 | Intron | - |
| CWSNP576 | Ca-Kabuli-Chr1 | 7160773 | [G/A] | Ca07974 | Intron | - |

| SNP IDs | Chromosomes | Physical positions (bp) | SNPs | Gene accession IDs | Sequence components of genes | Putative functions |
|---|---|---|---|---|---|---|
| CWSNP577 | Ca-Kabuli-Chr1 | 7184813 | [A/C] | Ca07975 | Synonymous-CDS | Root hair defective 3GTP-binding |
| CWSNP578 | Ca-Kabuli-Chr1 | 7187934 | [A/G] | - | Intergenic | - |
| CWSNP579 | Ca-Kabuli-Chr1 | 7196570 | [T/C] | - | URR | - |
| CWSNP580 | Ca-Kabuli-Chr1 | 7196601 | [T/C] | - | URR | - |
| CWSNP581 | Ca-Kabuli-Chr1 | 7231750 | [A/C] | - | DRR | - |
| CWSNP582 | Ca-Kabuli-Chr1 | 7325164 | [T/A] | - | DRR | - |
| CWSNP583 | Ca-Kabuli-Chr1 | 7417788 | [G/A] | Ca07994 | Intron | Homeobox |
| CWSNP584 | Ca-Kabuli-Chr1 | 7467280 | [A/G] | Ca07999 | Intron | AMP-dependent synthetase/ligase |
| CWSNP585 | Ca-Kabuli-Chr1 | 7478022 | [G/A] | Ca08000 | Synonymous-CDS | - |
| CWSNP586 | Ca-Kabuli-Chr1 | 7478046 | [T/G] | Ca08000 | Non-Synonymous-CDS | - |
| CWSNP587 | Ca-Kabuli-Chr1 | 7486102 | [C/T] | - | URR | - |
| CWSNP588 | Ca-Kabuli-Chr1 | 7486273 | [T/C] | - | URR | - |
| CWSNP589 | Ca-Kabuli-Chr1 | 7486282 | [G/A] | - | URR | - |
| CWSNP590 | Ca-Kabuli-Chr1 | 7497334 | [C/A] | - | Intergenic | - |
| CWSNP591 | Ca-Kabuli-Chr1 | 7497362 | [T/C] | - | Intergenic | - |
| CWSNP592 | Ca-Kabuli-Chr1 | 7500494 | [T/G] | Ca08003 | Synonymous-CDS | Protein kinase, catalytic domain |

| SNP IDs | Chromosomes | Physical positions (bp) | SNPs | Gene accession IDs | Sequence components of genes | Putative functions |
|---|---|---|---|---|---|---|
| CWSNP593 | Ca-Kabuli-Chr1 | 7500524 | [T/C] | Ca08003 | Synonymous-CDS | Protein kinase, catalytic domain |
| CWSNP594 | Ca-Kabuli-Chr1 | 7505779 | [A/T] | - | DRR | - |
| CWSNP595 | Ca-Kabuli-Chr1 | 7515002 | [T/C] | - | URR | - |
| CWSNP596 | Ca-Kabuli-Chr1 | 7515605 | [T/C] | - | URR | - |
| CWSNP597 | Ca-Kabuli-Chr1 | 7515578 | [A/C] | - | URR | - |
| CWSNP598 | Ca-Kabuli-Chr1 | 7557751 | [T/G] | Ca08009 | Intron | Histidinoldehydrogenase,conserved site |
| CWSNP599 | Ca-Kabuli-Chr1 | 7557811 | [A/T] | Ca08009 | Intron | Histidinoldehydrogenase,conserved site |
| CWSNP600 | Ca-Kabuli-Chr1 | 7690613 | [T/G] | Ca08021 | Non-Synonymous-CDS | - |
| CWSNP601 | Ca-Kabuli-Chr1 | 7690620 | [T/G] | Ca08021 | Non-Synonymous-CDS | - |
| CWSNP602 | Ca-Kabuli-Chr1 | 7690625 | [A/C] | Ca08021 | Synonymous-CDS | - |
| CWSNP603 | Ca-Kabuli-Chr1 | 7695844 | [T/C] | Ca08021 | Intron | - |
| CWSNP604 | Ca-Kabuli-Chr1 | 7811388 | [C/T] | - | DRR | - |
| CWSNP605 | Ca-Kabuli-Chr1 | 7925666 | [A/C] | Ca08042 | Synonymous-CDS | Biopterin transport-related protein BT1 |
| CWSNP606 | Ca-Kabuli-Chr1 | 7925774 | [C/T] | Ca08042 | Synonymous-CDS | Biopterin transport-related protein BT1 |
| CWSNP607 | Ca-Kabuli-Chr1 | 7969202 | [A/C] | - | DRR | - |
| CWSNP608 | Ca-Kabuli-Chr1 | 8036666 | [C/T] | Ca08051 | Intron | NAD-dependent epimerase/dehydratase |

| SNP IDs | Chromosomes | Physical positions (bp) | SNPs | Gene accession IDs | Sequence components of genes | Putative functions |
|---|---|---|---|---|---|---|
| CWSNP609 | Ca-Kabuli-Chr1 | 8036706 | [A/G] | Ca08051 | Intron | NAD-dependent epimerase/dehydratase |
| CWSNP610 | Ca-Kabuli-Chr1 | 8036917 | [T/G] | Ca08051 | Intron | NAD-dependent epimerase/dehydratase |
| CWSNP611 | Ca-Kabuli-Chr1 | 8036903 | [A/T] | Ca08051 | Intron | NAD-dependent epimerase/dehydratase |
| CWSNP612 | Ca-Kabuli-Chr1 | 8107967 | [A/C] | Ca08057 | Non-Synonymous-CDS | RNA helicase, ATP-dependent, DEAD-box,conserved site |
| CWSNP613 | Ca-Kabuli-Chr1 | 8108014 | [C/T] | Ca08057 | Non-Synonymous-CDS | RNA helicase, ATP-dependent, DEAD-box,conserved site |
| CWSNP614 | Ca-Kabuli-Chr1 | 8111588 | [G/A] | Ca08057 | Intron | RNA helicase, ATP-dependent, DEAD-box,conserved site |
| CWSNP615 | Ca-Kabuli-Chr1 | 8112343 | [C/T] | Ca08057 | Synonymous-CDS | RNA helicase, ATP-dependent, DEAD-box,conserved site |
| CWSNP616 | Ca-Kabuli-Chr1 | 8113235 | [G/T] | Ca08057 | Non-Synonymous-CDS | RNA helicase, ATP-dependent, DEAD-box,conserved site |
| CWSNP617 | Ca-Kabuli-Chr1 | 8113449 | [T/A] | Ca08057 | Synonymous-CDS | RNA helicase, ATP-dependent, DEAD-box,conserved site |
| CWSNP618 | Ca-Kabuli-Chr1 | 8113403 | [A/T] | Ca08057 | Non-Synonymous-CDS | RNA helicase, ATP-dependent, DEAD-box,conserved site |
| CWSNP619 | Ca-Kabuli-Chr1 | 8113398 | [C/G] | Ca08057 | Non-Synonymous-CDS | RNA helicase, ATP-dependent, DEAD-box,conserved site |
| CWSNP620 | Ca-Kabuli-Chr1 | 8123033 | [A/G] | - | URR | - |
| CWSNP621 | Ca-Kabuli-Chr1 | 8123257 | [A/G] | - | URR | - |
| CWSNP622 | Ca-Kabuli-Chr1 | 8123481 | [A/G] | - | URR | - |
| CWSNP623 | Ca-Kabuli-Chr1 | 8134368 | [T/C] | - | DRR | - |
| CWSNP624 | Ca-Kabuli-Chr1 | 8192580 | [T/C] | - | Intergenic | - |

| SNP IDs | Chromosomes | Physical positions (bp) | SNPs | Gene accession IDs | Sequence components of genes | Putative functions |
|---|---|---|---|---|---|---|
| CWSNP625 | Ca-Kabuli-Chr1 | 8464621 | [C/G] | Ca08082 | Intron | - |
| CWSNP626 | Ca-Kabuli-Chr1 | 8665705 | [T/G] | Ca02944 | Intron | ATPase,P-type,K/Mg/Cd/Cu/Zn/Na/Ca/Na/H-transporter |
| CWSNP627 | Ca-Kabuli-Chr1 | 8697177 | [G/T] | Ca02942 | Intron | Sodium/calcium exchanger membrane region |
| CWSNP628 | Ca-Kabuli-Chr1 | 8735740 | [G/T] | Ca02937 | Intron | Ubiquitin-associated/translation elongation factor EF1B, N-terminal |
| CWSNP629 | Ca-Kabuli-Chr1 | 8735727 | [G/C] | Ca02937 | Intron | Ubiquitin-associated/translation elongation factor EF1B, N-terminal |
| CWSNP630 | Ca-Kabuli-Chr1 | 8736733 | [G/A] | Ca02937 | Intron | Ubiquitin-associated/translation elongation factor EF1B, N-terminal |
| CWSNP631 | Ca-Kabuli-Chr1 | 8736711 | [G/T] | Ca02937 | Intron | Ubiquitin-associated/translation elongation factor EF1B, N-terminal |
| CWSNP632 | Ca-Kabuli-Chr1 | 8737380 | [A/G] | Ca02937 | Synonymous-CDS | Ubiquitin-associated/translation elongation factor EF1B, N-terminal |
| CWSNP633 | Ca-Kabuli-Chr1 | 8767122 | [T/A] | Ca02933 | Intron | Pentatrico peptide repeat |
| CWSNP634 | Ca-Kabuli-Chr1 | 8767121 | [A/T] | Ca02933 | Intron | Pentatrico peptide repeat |
| CWSNP635 | Ca-Kabuli-Chr1 | 8799685 | [T/G] | - | Intergenic | - |
| CWSNP636 | Ca-Kabuli-Chr1 | 8799701 | [T/C] | - | Intergenic | - |
| CWSNP637 | Ca-Kabuli-Chr1 | 8799708 | [T/C] | - | Intergenic | - |
| CWSNP638 | Ca-Kabuli-Chr1 | 8809188 | [T/A] | Ca02928 | Intron | ABC transporter, transmembrane domain |
| CWSNP639 | Ca-Kabuli-Chr1 | 8809191 | [C/T] | Ca02928 | Intron | ABC transporter, transmembrane domain |
| CWSNP640 | Ca-Kabuli-Chr1 | 8809295 | [A/T] | Ca02928 | Intron | ABC transporter, transmembrane domain |

| SNP IDs | Chromosomes | Physical positions (bp) | SNPs | Gene accession IDs | Sequence components of genes | Putative functions |
|---|---|---|---|---|---|---|
| CWSNP641 | Ca-Kabuli-Chr1 | 8809517 | [C/G] | Ca02928 | Intron | ABC transporter, transmembrane domain |
| CWSNP642 | Ca-Kabuli-Chr1 | 8822375 | [G/A] | - | DRR | - |
| CWSNP643 | Ca-Kabuli-Chr1 | 8822366 | [T/A] | - | DRR | - |
| CWSNP644 | Ca-Kabuli-Chr1 | 8852863 | [G/T] | - | Intergenic | - |
| CWSNP645 | Ca-Kabuli-Chr1 | 8852928 | [T/A] | - | Intergenic | - |
| CWSNP646 | Ca-Kabuli-Chr1 | 8852981 | [C/A] | - | Intergenic | - |
| CWSNP647 | Ca-Kabuli-Chr1 | 8852943 | [C/A] | - | Intergenic | - |
| CWSNP648 | Ca-Kabuli-Chr1 | 8853010 | [A/G] | - | Intergenic | - |
| CWSNP649 | Ca-Kabuli-Chr1 | 8854211 | [G/T] | Ca02923 | Intron | Natural resistance-associated macrophage protein |
| CWSNP650 | Ca-Kabuli-Chr1 | 8891438 | [T/G] | Ca02918 | Synonymous-CDS | ATPase ,P-type, H+transporting proton pump |
| CWSNP651 | Ca-Kabuli-Chr1 | 8891485 | [T/G] | Ca02918 | Intron | ATPase ,P-type, H+transporting proton pump |
| CWSNP652 | Ca-Kabuli-Chr1 | 8893985 | [G/A] | - | DRR | - |
| CWSNP653 | Ca-Kabuli-Chr1 | 8901769 | [C/A] | - | DRR | - |
| CWSNP654 | Ca-Kabuli-Chr1 | 8901842 | [T/A] | - | DRR | - |
| CWSNP655 | Ca-Kabuli-Chr1 | 8941004 | [A/C] | - | Intergenic | - |
| CWSNP656 | Ca-Kabuli-Chr1 | 8941029 | [G/A] | - | Intergenic | - |

| SNP IDs | Chromosomes | Physical positions (bp) | SNPs | Gene accession IDs | Sequence components of genes | Putative functions |
|---|---|---|---|---|---|---|
| CWSNP657 | Ca-Kabuli-Chr1 | 8941031 | [G/C] | - | Intergenic | - |
| CWSNP658 | Ca-Kabuli-Chr1 | 8942137 | [T/C] | Ca02909 | Non-Synonymous-CDS | Zinc finger,UBP-type |
| CWSNP659 | Ca-Kabuli-Chr1 | 8942131 | [T/C] | Ca02909 | Non-Synonymous-CDS | Zinc finger,UBP-type |
| CWSNP660 | Ca-Kabuli-Chr1 | 8942198 | [A/G] | Ca02909 | Synonymous-CDS | Zinc finger,UBP-type |
| CWSNP661 | Ca-Kabuli-Chr1 | 8942179 | [C/G] | Ca02909 | Non-Synonymous-CDS | Zinc finger,UBP-type |
| CWSNP662 | Ca-Kabuli-Chr1 | 8949723 | [G/T] | - | URR | - |
| CWSNP663 | Ca-Kabuli-Chr1 | 8960630 | [T/C] | - | DRR | - |
| CWSNP664 | Ca-Kabuli-Chr1 | 8969321 | [C/T] | Ca02904 | Intron | - |
| CWSNP665 | Ca-Kabuli-Chr1 | 8971718 | [C/G] | Ca02904 | Non-Synonymous-CDS | - |
| CWSNP666 | Ca-Kabuli-Chr1 | 8976169 | [A/T] | Ca02904 | Non-Synonymous-CDS | - |
| CWSNP667 | Ca-Kabuli-Chr1 | 9031825 | [G/T] | - | Intergenic | - |
| CWSNP668 | Ca-Kabuli-Chr1 | 9035744 | [G/A] | - | URR | - |
| CWSNP669 | Ca-Kabuli-Chr1 | 9040419 | [T/A] | - | DRR | - |
| CWSNP670 | Ca-Kabuli-Chr1 | 9048675 | [A/T] | - | DRR | - |
| CWSNP671 | Ca-Kabuli-Chr1 | 9065933 | [T/C] | - | Intergenic | - |
| CWSNP672 | Ca-Kabuli-Chr1 | 9093812 | [C/G] | - | URR | - |

| SNP IDs | Chromosomes | Physical positions (bp) | SNPs | Gene accession IDs | Sequence components of genes | Putative functions |
|---|---|---|---|---|---|---|
| CWSNP673 | Ca-Kabuli-Chr1 | 9093964 | [T/A] | - | URR | - |
| CWSNP674 | Ca-Kabuli-Chr1 | 9097885 | [A/G] | - | Intergenic | - |
| CWSNP675 | Ca-Kabuli-Chr1 | 9097881 | [C/G] | - | Intergenic | - |
| CWSNP676 | Ca-Kabuli-Chr1 | 9097879 | [C/A] | - | Intergenic | - |
| CWSNP677 | Ca-Kabuli-Chr1 | 9097878 | [A/G] | - | Intergenic | - |
| CWSNP678 | Ca-Kabuli-Chr1 | 9097877 | [C/A] | - | Intergenic | - |
| CWSNP679 | Ca-Kabuli-Chr1 | 9097869 | [G/T] | - | Intergenic | - |
| CWSNP680 | Ca-Kabuli-Chr1 | 9097949 | [A/C] | - | Intergenic | - |
| CWSNP681 | Ca-Kabuli-Chr1 | 9098003 | [C/T] | - | Intergenic | - |
| CWSNP682 | Ca-Kabuli-Chr1 | 9105432 | [C/T] | Ca02885 | Synonymous-CDS | Initiation factor2B-related |
| CWSNP683 | Ca-Kabuli-Chr1 | 9195312 | [G/A] | - | DRR | - |
| CWSNP684 | Ca-Kabuli-Chr1 | 9237766 | [T/G] | - | Intergenic | - |
| CWSNP685 | Ca-Kabuli-Chr1 | 9237813 | [G/T] | - | Intergenic | - |
| CWSNP686 | Ca-Kabuli-Chr1 | 9269888 | [T/A] | Ca02867 | Non-Synonymous-CDS | Nucleoporininte racting component Nup93/Nic96 |
| CWSNP687 | Ca-Kabuli-Chr1 | 9603896 | [T/A] | Ca02837 | Synonymous-CDS | WD40 repeat |
| CWSNP688 | Ca-Kabuli-Chr1 | 9634823 | [C/T] | Ca02835 | Synonymous-CDS | Peptidase C14,caspasenon-catalytic subunit p10 |

| SNP IDs | Chromosomes | Physical positions (bp) | SNPs | Gene accession IDs | Sequence components of genes | Putative functions |
|---|---|---|---|---|---|---|
| CWSNP689 | Ca-Kabuli-Chr1 | 9659316 | [C/T] | - | DRR | - |
| CWSNP690 | Ca-Kabuli-Chr1 | 9705162 | [A/C] | - | DRR | - |
| CWSNP691 | Ca-Kabuli-Chr1 | 10191578 | [A/C] | - | Intergenic | - |
| CWSNP692 | Ca-Kabuli-Chr1 | 10213111 | [T/C] | - | DRR | - |
| CWSNP693 | Ca-Kabuli-Chr1 | 10364986 | [C/G] | - | Intergenic | - |
| CWSNP694 | Ca-Kabuli-Chr1 | 10365024 | [G/A] | - | Intergenic | - |
| CWSNP695 | Ca-Kabuli-Chr1 | 10365029 | [T/A] | - | DRR | - |
| CWSNP696 | Ca-Kabuli-Chr1 | 10377700 | [G/C] | Ca02740 | Intron | Glycosidehydrolase,family35 |
| CWSNP697 | Ca-Kabuli-Chr1 | 10377698 | [T/C] | Ca02740 | Intron | Glycosidehydrolase,family35 |
| CWSNP698 | Ca-Kabuli-Chr1 | 10377691 | [T/C] | Ca02740 | Intron | Glycosidehydrolase,family35 |
| CWSNP699 | Ca-Kabuli-Chr1 | 10377860 | [T/A] | Ca02740 | Intron | Glycosidehydrolase,family35 |
| CWSNP700 | Ca-Kabuli-Chr1 | 10527733 | [A/G] | - | DRR | - |
| CWSNP701 | Ca-Kabuli-Chr1 | 10601318 | [T/G] | Ca02710 | Non-Synonymous-CDS | Pentatrico peptide repeat |
| CWSNP702 | Ca-Kabuli-Chr1 | 10623359 | [T/G] | - | Intergenic | - |
| CWSNP703 | Ca-Kabuli-Chr1 | 10655633 | [G/T] | Ca02700 | Intron | Protein of unknown function DUF3755 |
| CWSNP704 | Ca-Kabuli-Chr1 | 10710672 | [T/G] | - | DRR | - |

| SNP IDs | Chromosomes | Physical positions (bp) | SNPs | Gene accession IDs | Sequence components of genes | Putative functions |
|---|---|---|---|---|---|---|
| CWSNP705 | Ca-Kabuli-Chr1 | 10713001 | [T/A] | - | Intergenic | - |
| CWSNP706 | Ca-Kabuli-Chr1 | 10761609 | [G/T] | - | DRR | - |
| CWSNP707 | Ca-Kabuli-Chr1 | 10837203 | [C/T] | - | DRR | - |
| CWSNP708 | Ca-Kabuli-Chr1 | 10861332 | [C/G] | Ca02673 | Intron | Glycosidehydrolase,family1 |
| CWSNP709 | Ca-Kabuli-Chr1 | 10861326 | [T/G] | Ca02673 | Intron | Glycosidehydrolase,family1 |
| CWSNP710 | Ca-Kabuli-Chr1 | 10869336 | [G/A] | Ca02671 | Intron | Oxidoreductase,N-terminal |
| CWSNP711 | Ca-Kabuli-Chr1 | 11103176 | [T/C] | - | DRR | - |
| CWSNP712 | Ca-Kabuli-Chr1 | 11193334 | [G/C] | - | DRR | - |
| CWSNP713 | Ca-Kabuli-Chr1 | 11225828 | [T/C] | - | URR | - |
| CWSNP714 | Ca-Kabuli-Chr1 | 11227614 | [T/C] | - | URR | - |
| CWSNP715 | Ca-Kabuli-Chr1 | 11230109 | [T/C] | Ca02632 | Non-Synonymous-CDS | Pumilio RNA-binding repeat |
| CWSNP716 | Ca-Kabuli-Chr1 | 11230115 | [G/C] | Ca02632 | Non-Synonymous-CDS | Pumilio RNA-binding repeat |
| CWSNP717 | Ca-Kabuli-Chr1 | 11230116 | [T/C] | Ca02632 | Synonymous-CDS | Pumilio RNA-binding repeat |
| CWSNP718 | Ca-Kabuli-Chr1 | 11230118 | [A/T] | Ca02632 | Non-Synonymous-CDS | Pumilio RNA-binding repeat |
| CWSNP719 | Ca-Kabuli-Chr1 | 11230125 | [A/C] | Ca02632 | Synonymous-CDS | Pumilio RNA-binding repeat |
| CWSNP720 | Ca-Kabuli-Chr1 | 11230126 | [G/T] | Ca02632 | Non-Synonymous-CDS | Pumilio RNA-binding repeat |

| SNP IDs | Chromosomes | Physical positions (bp) | SNPs | Gene accession IDs | Sequence components of genes | Putative functions |
|---|---|---|---|---|---|---|
| CWSNP721 | Ca-Kabuli-Chr1 | 11230127 | [G/T] | Ca02632 | Non-Synonymous-CDS | Pumilio RNA-binding repeat |
| CWSNP722 | Ca-Kabuli-Chr1 | 11230128 | [T/G] | Ca02632 | Synonymous-CDS | Pumilio RNA-binding repeat |
| CWSNP723 | Ca-Kabuli-Chr1 | 11230133 | [G/A] | Ca02632 | Non-Synonymous-CDS | Pumilio RNA-binding repeat |
| CWSNP724 | Ca-Kabuli-Chr1 | 11230998 | [A/T] | Ca02632 | Synonymous-CDS | Pumilio RNA-binding repeat |
| CWSNP725 | Ca-Kabuli-Chr1 | 11231064 | [A/G] | Ca02632 | Synonymous-CDS | Pumilio RNA-binding repeat |
| CWSNP726 | Ca-Kabuli-Chr1 | 11231077 | [T/C] | Ca02632 | Non-Synonymous-CDS | Pumilio RNA-binding repeat |
| CWSNP727 | Ca-Kabuli-Chr1 | 11235211 | [C/T] | - | URR | - |
| CWSNP728 | Ca-Kabuli-Chr1 | 11265367 | [T/C] | - | Intergenic | - |
| CWSNP729 | Ca-Kabuli-Chr1 | 11277703 | [T/A] | - | DRR | - |
| CWSNP730 | Ca-Kabuli-Chr1 | 11277964 | [C/T] | - | DRR | - |
| CWSNP731 | Ca-Kabuli-Chr1 | 11284183 | [T/C] | - | Intergenic | - |
| CWSNP732 | Ca-Kabuli-Chr1 | 11285192 | [G/A] | Ca02625 | Synonymous-CDS | Transcription factor GRAS |
| CWSNP733 | Ca-Kabuli-Chr1 | 11285247 | [T/C] | Ca02625 | Synonymous-CDS | Transcription factor GRAS |
| CWSNP734 | Ca-Kabuli-Chr1 | 11291516 | [G/A] | - | URR | - |
| CWSNP735 | Ca-Kabuli-Chr1 | 11291741 | [A/C] | Ca02624 | Intron | SANT domain, DNA binding |
| CWSNP736 | Ca-Kabuli-Chr1 | 11291740 | [T/A] | Ca02624 | Intron | SANT domain, DNA binding |

| SNP IDs | Chromosomes | Physical positions (bp) | SNPs | Gene accession IDs | Sequence components of genes | Putative functions |
|---|---|---|---|---|---|---|
| CWSNP737 | Ca-Kabuli-Chr1 | 11291737 | [C/A] | Ca02624 | Intron | SANT domain, DNA binding |
| CWSNP738 | Ca-Kabuli-Chr1 | 11319264 | [A/C] | - | DRR | - |
| CWSNP739 | Ca-Kabuli-Chr1 | 11321670 | [G/C] | - | Intergenic | - |
| CWSNP740 | Ca-Kabuli-Chr1 | 11324018 | [C/A] | - | DRR | - |
| CWSNP741 | Ca-Kabuli-Chr1 | 11324023 | [C/T] | - | DRR | - |
| CWSNP742 | Ca-Kabuli-Chr1 | 11324035 | [C/T] | - | DRR | - |
| CWSNP743 | Ca-Kabuli-Chr1 | 11324050 | [G/T] | - | DRR | - |
| CWSNP744 | Ca-Kabuli-Chr1 | 11342388 | [T/C] | - | DRR | - |
| CWSNP745 | Ca-Kabuli-Chr1 | 11361588 | [T/G] | Ca02617 | Non-Synonymous-CDS | Protein kinase, catalytic domain |
| CWSNP746 | Ca-Kabuli-Chr1 | 11361583 | [T/C] | Ca02617 | Synonymous-CDS | Protein kinase, catalytic domain |
| CWSNP747 | Ca-Kabuli-Chr1 | 11405015 | [A/G] | Ca02611 | Non-Synonymous-CDS | Protein kinase, catalytic domain |
| CWSNP748 | Ca-Kabuli-Chr1 | 11502148 | [C/A] | - | DRR | - |
| CWSNP749 | Ca-Kabuli-Chr1 | 11514888 | [T/A] | Ca02599 | Intron | Histone H2A |
| CWSNP750 | Ca-Kabuli-Chr1 | 11522875 | [C/T] | Ca02598 | Synonymous-CDS | Histone H2A |
| CWSNP751 | Ca-Kabuli-Chr1 | 11601347 | [T/C] | Ca02586 | Intron | RNA recognition motif domain |
| CWSNP752 | Ca-Kabuli-Chr1 | 11624419 | [C/G] | - | Intergenic | - |

| SNP IDs | Chromosomes | Physical positions (bp) | SNPs | Gene accession IDs | Sequence components of genes | Putative functions |
|---|---|---|---|---|---|---|
| CWSNP753 | Ca-Kabuli-Chr1 | 11624464 | [C/T] | - | Intergenic | - |
| CWSNP754 | Ca-Kabuli-Chr1 | 11669794 | [T/G] | Ca02583 | Synonymous-CDS | Ankyrin repeat |
| CWSNP755 | Ca-Kabuli-Chr1 | 11673478 | [A/C] | - | Intergenic | - |
| CWSNP756 | Ca-Kabuli-Chr1 | 11677097 | [C/A] | - | Intergenic | - |
| CWSNP757 | Ca-Kabuli-Chr1 | 11685769 | [A/G] | Ca02582 | Synonymous-CDS | Protein kinase, catalytic domain |
| CWSNP758 | Ca-Kabuli-Chr1 | 11685790 | [T/C] | Ca02582 | Synonymous-CDS | Protein kinase, catalytic domain |
| CWSNP759 | Ca-Kabuli-Chr1 | 11793443 | [C/A] | Ca02573 | Non-Synonymous-CDS | Alcohol dehydrogenase superfamily, zinc-containing |
| CWSNP760 | Ca-Kabuli-Chr1 | 11822305 | [G/T] | - | Intergenic | - |
| CWSNP761 | Ca-Kabuli-Chr1 | 11822309 | [C/A] | - | Intergenic | - |
| CWSNP762 | Ca-Kabuli-Chr1 | 12189822 | [T/G] | Ca02538 | Synonymous-CDS | Double-stranded RNA-binding |
| CWSNP763 | Ca-Kabuli-Chr1 | 12326230 | [A/G] | - | URR | - |
| CWSNP764 | Ca-Kabuli-Chr1 | 12413012 | [G/T] | Ca02509 | Non-Synonymous-CDS | - |
| CWSNP765 | Ca-Kabuli-Chr1 | 12413016 | [T/A] | Ca02509 | Non-Synonymous-CDS | - |
| CWSNP766 | Ca-Kabuli-Chr1 | 12413025 | [G/C] | Ca02509 | Non-Synonymous-CDS | - |
| CWSNP767 | Ca-Kabuli-Chr1 | 12588525 | [A/G] | Ca02496 | Intron | - |
| CWSNP768 | Ca-Kabuli-Chr1 | 12688435 | [A/C] | - | DRR | - |

| SNP IDs | Chromosomes | Physical positions (bp) | SNPs | Gene accession IDs | Sequence components of genes | Putative functions |
|---|---|---|---|---|---|---|
| CWSNP769 | Ca-Kabuli-Chr1 | 12736353 | [A/C] | - | DRR | - |
| CWSNP770 | Ca-Kabuli-Chr1 | 12800610 | [A/C] | - | URR | - |
| CWSNP771 | Ca-Kabuli-Chr1 | 12800607 | [T/A] | - | URR | - |
| CWSNP772 | Ca-Kabuli-Chr1 | 12800601 | [T/A] | - | URR | - |
| CWSNP773 | Ca-Kabuli-Chr1 | 12800597 | [G/T] | - | URR | - |
| CWSNP774 | Ca-Kabuli-Chr1 | 12845445 | [A/C] | - | URR | - |
| CWSNP775 | Ca-Kabuli-Chr1 | 12880408 | [G/A] | - | Intergenic | - |
| CWSNP776 | Ca-Kabuli-Chr1 | 12992445 | [A/C] | - | Intergenic | - |
| CWSNP777 | Ca-Kabuli-Chr1 | 12992423 | [A/C] | - | Intergenic | - |
| CWSNP778 | Ca-Kabuli-Chr1 | 12992569 | [A/G] | - | Intergenic | - |
| CWSNP779 | Ca-Kabuli-Chr1 | 13034708 | [T/G] | - | URR | - |
| CWSNP780 | Ca-Kabuli-Chr1 | 13076695 | [G/A] | Ca14071 | Non-Synonymous-CDS | Zinc finger,RING-type |
| CWSNP781 | Ca-Kabuli-Chr1 | 13156473 | [A/C] | - | Intergenic | - |
| CWSNP782 | Ca-Kabuli-Chr1 | 13158244 | [C/T] | - | Intergenic | - |
| CWSNP783 | Ca-Kabuli-Chr1 | 13158201 | [C/T] | - | Intergenic | - |
| CWSNP784 | Ca-Kabuli-Chr1 | 13158192 | [T/C] | - | Intergenic | - |

| SNP IDs | Chromosomes | Physical positions (bp) | SNPs | Gene accession IDs | Sequence components of genes | Putative functions |
|---|---|---|---|---|---|---|
| CWSNP785 | Ca-Kabuli-Chr1 | 13497572 | [G/T] | Ca14116 | Intron | Photosystem IIPsp29, biogenesis |
| CWSNP786 | Ca-Kabuli-Chr1 | 13549635 | [T/C] | Ca14121 | Synonymous-CDS | Kinesin , motor domain |
| CWSNP787 | Ca-Kabuli-Chr1 | 13550600 | [G/A] | Ca14121 | Intron | Kinesin , motor domain |
| CWSNP788 | Ca-Kabuli-Chr1 | 13560364 | [T/G] | - | Intergenic | - |
| CWSNP789 | Ca-Kabuli-Chr1 | 13590903 | [G/T] | Ca14125 | Synonymous-CDS | Protein kinase, catalytic domain |
| CWSNP790 | Ca-Kabuli-Chr1 | 13591177 | [G/A] | Ca14125 | Non-Synonymous-CDS | Protein kinase, catalytic domain |
| CWSNP791 | Ca-Kabuli-Chr1 | 13591203 | [C/G] | Ca14125 | Non-Synonymous-CDS | Protein kinase, catalytic domain |
| CWSNP792 | Ca-Kabuli-Chr1 | 13596411 | [G/T] | - | DRR | - |
| CWSNP793 | Ca-Kabuli-Chr1 | 13596528 | [T/A] | - | DRR | - |
| CWSNP794 | Ca-Kabuli-Chr1 | 13596539 | [A/G] | - | DRR | - |
| CWSNP795 | Ca-Kabuli-Chr1 | 13596659 | [T/A] | - | DRR | - |
| CWSNP796 | Ca-Kabuli-Chr1 | 13596631 | [C/T] | - | DRR | - |
| CWSNP797 | Ca-Kabuli-Chr1 | 13596621 | [C/T] | - | DRR | - |
| CWSNP798 | Ca-Kabuli-Chr1 | 13607486 | [A/C] | Ca14127 | Synonymous-CDS | Protein kinase, catalytic domain |
| CWSNP799 | Ca-Kabuli-Chr1 | 13668759 | [T/A] | - | Intergenic | - |
| CWSNP800 | Ca-Kabuli-Chr1 | 13680008 | [G/C] | - | URR | - |

| SNP IDs | Chromosomes | Physical positions (bp) | SNPs | Gene accession IDs | Sequence components of genes | Putative functions |
|---|---|---|---|---|---|---|
| CWSNP801 | Ca-Kabuli-Chr1 | 13762410 | [C/A] | Ca14138 | Non-Synonymous-CDS | SET domain |
| CWSNP802 | Ca-Kabuli-Chr1 | 13769805 | [C/T] | - | URR | - |
| CWSNP803 | Ca-Kabuli-Chr1 | 13769771 | [T/C] | - | URR | - |
| CWSNP804 | Ca-Kabuli-Chr1 | 13782049 | [C/T] | Ca14141 | Synonymous-CDS | Glycosidehydrolase,family 14B, plant |
| CWSNP805 | Ca-Kabuli-Chr1 | 13828744 | [A/T] | Ca14148 | Intron | CCT domain |
| CWSNP806 | Ca-Kabuli-Chr1 | 14015757 | [T/C] | Ca07128 | Non-Synonymous-CDS | Ionotropic glutamate receptor |
| CWSNP807 | Ca-Kabuli-Chr1 | 14237273 | [A/T] | Ca07112 | Non-Synonymous-CDS | Cellulo sesynthase |
| CWSNP808 | Ca-Kabuli-Chr1 | 14623271 | [T/C] | - | Intergenic | - |
| CWSNP809 | Ca-Kabuli-Chr1 | 14916633 | [G/C] | - | Intergenic | - |
| CWSNP810 | Ca-Kabuli-Chr1 | 15253285 | [A/G] | - | Intergenic | - |
| CWSNP811 | Ca-Kabuli-Chr1 | 15319181 | [T/C] | Ca07002 | Synonymous-CDS | Zinc finger,PHD-type |
| CWSNP812 | Ca-Kabuli-Chr1 | 15613071 | [A/C] | Ca06983 | Intron | Ras GTPase |
| CWSNP813 | Ca-Kabuli-Chr1 | 15683101 | [A/C] | - | Intergenic | - |
| CWSNP814 | Ca-Kabuli-Chr1 | 15683117 | [G/T] | - | Intergenic | - |
| CWSNP815 | Ca-Kabuli-Chr1 | 15760638 | [C/A] | Ca06971 | Intron | Domain of unknown function DUF1692 |
| CWSNP816 | Ca-Kabuli-Chr1 | 15960425 | [A/C] | Ca06958 | Intron | Glycosyltransferase,family8 |

| SNP IDs | Chromosomes | Physical positions (bp) | SNPs | Gene accession IDs | Sequence components of genes | Putative functions |
|---|---|---|---|---|---|---|
| CWSNP817 | Ca-Kabuli-Chr1 | 15965278 | [G/A] | - | URR | - |
| CWSNP818 | Ca-Kabuli-Chr1 | 16061385 | [A/G] | - | Intergenic | - |
| CWSNP819 | Ca-Kabuli-Chr1 | 16099437 | [T/G] | - | Intergenic | - |
| CWSNP820 | Ca-Kabuli-Chr1 | 16099438 | [G/A] | - | Intergenic | - |
| CWSNP821 | Ca-Kabuli-Chr1 | 16099468 | [G/A] | - | Intergenic | - |
| CWSNP822 | Ca-Kabuli-Chr1 | 16099444 | [T/C] | - | Intergenic | - |
| CWSNP823 | Ca-Kabuli-Chr1 | 16099459 | [T/C] | - | Intergenic | - |
| CWSNP824 | Ca-Kabuli-Chr1 | 16099457 | [T/C] | - | Intergenic | - |
| CWSNP825 | Ca-Kabuli-Chr1 | 16104255 | [G/T] | - | Intergenic | - |
| CWSNP826 | Ca-Kabuli-Chr1 | 16104351 | [G/A] | - | Intergenic | - |
| CWSNP827 | Ca-Kabuli-Chr1 | 16104334 | [T/G] | - | Intergenic | - |
| CWSNP828 | Ca-Kabuli-Chr1 | 16104298 | [C/T] | - | Intergenic | - |
| CWSNP829 | Ca-Kabuli-Chr1 | 16114607 | [C/A] | Ca06940 | Non-Synonymous-CDS | General substrate transporter |
| CWSNP830 | Ca-Kabuli-Chr1 | 16196930 | [A/G] | - | Intergenic | - |
| CWSNP831 | Ca-Kabuli-Chr1 | 16228049 | [C/A] | Ca06935 | Intron | NADPH-dependentFMNreductase |
| CWSNP832 | Ca-Kabuli-Chr1 | 16228039 | [C/A] | Ca06935 | Intron | NADPH-dependentFMNreductase |

| SNP IDs | Chromosomes | Physical positions (bp) | SNPs | Gene accession IDs | Sequence components of genes | Putative functions |
|---|---|---|---|---|---|---|
| CWSNP833 | Ca-Kabuli-Chr1 | 16329161 | [G/A] | - | Intergenic | - |
| CWSNP834 | Ca-Kabuli-Chr1 | 16376254 | [G/T] | Ca06927 | Non-Synonymous-CDS | Ubiquitin-associated/translation elongation factor EF1B, N-terminal |
| CWSNP835 | Ca-Kabuli-Chr1 | 16379239 | [A/G] | Ca06927 | Intron | Ubiquitin-associated/translation elongation factor EF1B, N-terminal |
| CWSNP836 | Ca-Kabuli-Chr1 | 16379258 | [C/T] | Ca06927 | Intron | Ubiquitin-associated/translation elongation factor EF1B, N-terminal |
| CWSNP837 | Ca-Kabuli-Chr1 | 16454432 | [C/T] | - | DRR | - |
| CWSNP838 | Ca-Kabuli-Chr1 | 16493894 | [A/G] | Ca06914 | Synonymous-CDS | PeptidaseS54,rhomboid |
| CWSNP839 | Ca-Kabuli-Chr1 | 16575167 | [A/G] | Ca06906 | Intron | Protein phosphatase2C-like |
| CWSNP840 | Ca-Kabuli-Chr1 | 16933431 | [T/G] | - | URR | - |
| CWSNP841 | Ca-Kabuli-Chr1 | 17023111 | [G/C] | Ca06866 | Non-Synonymous-CDS | Protein kinase, catalytic domain |
| CWSNP842 | Ca-Kabuli-Chr1 | 17093640 | [T/C] | Ca06863 | Non-Synonymous-CDS | - |
| CWSNP843 | Ca-Kabuli-Chr1 | 17167019 | [G/A] | - | Intergenic | - |
| CWSNP844 | Ca-Kabuli-Chr1 | 17170727 | [T/C] | - | URR | - |
| CWSNP845 | Ca-Kabuli-Chr1 | 17194835 | [C/T] | - | URR | - |
| CWSNP846 | Ca-Kabuli-Chr1 | 17328721 | [G/A] | Ca22781 | Synonymous-CDS | Bromodomain |
| CWSNP847 | Ca-Kabuli-Chr1 | 17359688 | [A/C] | Ca22785 | Intron | Domain of unknown function DUF625 |
| CWSNP848 | Ca-Kabuli-Chr1 | 17359686 | [C/T] | Ca22785 | Intron | Domain of unknown function DUF625 |

| SNP IDs | Chromosomes | Physical positions (bp) | SNPs | Gene accession IDs | Sequence components of genes | Putative functions |
|---|---|---|---|---|---|---|
| CWSNP849 | Ca-Kabuli-Chr1 | 17395543 | [C/A] | Ca22785 | Non-Synonymous-CDS | Domain of unknown function DUF625 |
| CWSNP850 | Ca-Kabuli-Chr1 | 17437336 | [G/C] | Ca14789 | Synonymous-CDS | Glycosidehydrolase,family19,catalytic |
| CWSNP851 | Ca-Kabuli-Chr1 | 17983301 | [T/G] | Ca14744 | Synonymous-CDS | RAG1-activating protein-1-related |
| CWSNP852 | Ca-Kabuli-Chr1 | 17983310 | [T/G] | Ca14744 | Synonymous-CDS | RAG1-activating protein-1-related |
| CWSNP853 | Ca-Kabuli-Chr1 | 18064930 | [C/T] | Ca14736 | Non-Synonymous-CDS | - |
| CWSNP854 | Ca-Kabuli-Chr1 | 18065239 | [T/C] | Ca14736 | Non-Synonymous-CDS | - |
| CWSNP855 | Ca-Kabuli-Chr1 | 18092704 | [T/G] | - | Intergenic | - |
| CWSNP856 | Ca-Kabuli-Chr1 | 18131516 | [T/G] | - | URR | - |
| CWSNP857 | Ca-Kabuli-Chr1 | 18156634 | [A/G] | - | Intergenic | - |
| CWSNP858 | Ca-Kabuli-Chr1 | 18172762 | [G/T] | - | DRR | - |
| CWSNP859 | Ca-Kabuli-Chr1 | 18198444 | [C/T] | Ca14723 | Intron | - |
| CWSNP860 | Ca-Kabuli-Chr1 | 18488172 | [G/A] | - | URR | - |
| CWSNP861 | Ca-Kabuli-Chr1 | 18763916 | [G/A] | Ca17380 | Intron | SET domain |
| CWSNP862 | Ca-Kabuli-Chr1 | 18763911 | [C/A] | Ca17380 | Intron | SET domain |
| CWSNP863 | Ca-Kabuli-Chr1 | 18764225 | [C/T] | Ca17380 | Intron | SET domain |
| CWSNP864 | Ca-Kabuli-Chr1 | 18890616 | [G/A] | Ca17371 | Synonymous-CDS | Parallelbeta-helixrepeat |

| SNP IDs | Chromosomes | Physical positions (bp) | SNPs | Gene accession IDs | Sequence components of genes | Putative functions |
|---|---|---|---|---|---|---|
| CWSNP865 | Ca-Kabuli-Chr1 | 18910202 | [G/A] | - | DRR | - |
| CWSNP866 | Ca-Kabuli-Chr1 | 19000445 | [T/C] | - | Intergenic | - |
| CWSNP867 | Ca-Kabuli-Chr1 | 19130894 | [T/C] | - | Intergenic | - |
| CWSNP868 | Ca-Kabuli-Chr1 | 19130884 | [C/A] | - | Intergenic | - |
| CWSNP869 | Ca-Kabuli-Chr1 | 19130883 | [C/A] | - | Intergenic | - |
| CWSNP870 | Ca-Kabuli-Chr1 | 19130872 | [G/A] | - | Intergenic | - |
| CWSNP871 | Ca-Kabuli-Chr1 | 19218067 | [G/A] | - | Intergenic | - |
| CWSNP872 | Ca-Kabuli-Chr1 | 19218093 | [T/G] | - | Intergenic | - |
| CWSNP873 | Ca-Kabuli-Chr1 | 19254161 | [G/A] | Ca17349 | Intron | - |
| CWSNP874 | Ca-Kabuli-Chr1 | 19359812 | [A/C] | Ca17340 | Synonymous-CDS | Pentatrico peptide repeat |
| CWSNP875 | Ca-Kabuli-Chr1 | 19359844 | [C/A] | Ca17340 | Non-Synonymous-CDS | Pentatrico peptide repeat |
| CWSNP876 | Ca-Kabuli-Chr1 | 19526320 | [T/C] | Ca08868 | Non-Synonymous-CDS | Aldose1-epimerase |
| CWSNP877 | Ca-Kabuli-Chr1 | 19526652 | [G/T] | Ca08868 | Intron | Aldose1-epimerase |
| CWSNP878 | Ca-Kabuli-Chr1 | 19572408 | [C/T] | Ca08866 | Intron | Pumilio RNA-binding repeat |
| CWSNP879 | Ca-Kabuli-Chr1 | 19572494 | [G/C] | Ca08866 | Intron | Pumilio RNA-binding repeat |
| CWSNP880 | Ca-Kabuli-Chr1 | 19591671 | [A/G] | - | Intergenic | - |

| SNP IDs | Chromosomes | Physical positions (bp) | SNPs | Gene accession IDs | Sequence components of genes | Putative functions |
|---|---|---|---|---|---|---|
| CWSNP881 | Ca-Kabuli-Chr1 | 19903665 | [A/G] | Ca08847 | Intron | RNA polymerase I associated factor, A49-like |
| CWSNP882 | Ca-Kabuli-Chr1 | 19903683 | [G/A] | Ca08847 | Intron | RNA polymerase I associated factor, A49-like |
| CWSNP883 | Ca-Kabuli-Chr1 | 19903686 | [T/A] | Ca08847 | Intron | RNA polymerase I associated factor, A49-like |
| CWSNP884 | Ca-Kabuli-Chr1 | 19903690 | [A/G] | Ca08847 | Intron | RNA polymerase I associated factor, A49-like |
| CWSNP885 | Ca-Kabuli-Chr1 | 19903693 | [G/T] | Ca08847 | Intron | RNA polymerase I associated factor, A49-like |
| CWSNP886 | Ca-Kabuli-Chr1 | 19903701 | [G/A] | Ca08847 | Intron | RNA polymerase I associated factor, A49-like |
| CWSNP887 | Ca-Kabuli-Chr1 | 19903704 | [G/A] | Ca08847 | Intron | RNA polymerase I associated factor, A49-like |
| CWSNP888 | Ca-Kabuli-Chr1 | 20016525 | [C/T] | - | Intergenic | - |
| CWSNP889 | Ca-Kabuli-Chr1 | 20091291 | [T/G] | Ca08834 | Non-Synonymous-CDS | BRCT |
| CWSNP890 | Ca-Kabuli-Chr1 | 22465555 | [C/T] | - | Intergenic | - |
| CWSNP891 | Ca-Kabuli-Chr1 | 22719581 | [T/G] | - | Intergenic | - |
| CWSNP892 | Ca-Kabuli-Chr1 | 22743361 | [T/G] | Ca20613 | Non-Synonymous-CDS | Nucleosome assembly protein(NAP) |
| CWSNP893 | Ca-Kabuli-Chr1 | 23129094 | [C/T] | Ca22358 | Intron | Helicase,C-terminal |
| CWSNP894 | Ca-Kabuli-Chr1 | 23131178 | [A/C] | Ca22358 | Intron | Helicase,C-terminal |
| CWSNP895 | Ca-Kabuli-Chr1 | 23148736 | [A/C] | Ca22359 | Synonymous-CDS | Initiation factor2B-related |
| CWSNP896 | Ca-Kabuli-Chr1 | 23149053 | [A/G] | Ca22359 | Synonymous-CDS | Initiation factor2B-related |

| SNP IDs | Chromosomes | Physical positions (bp) | SNPs | Gene accession IDs | Sequence components of genes | Putative functions |
|---|---|---|---|---|---|---|
| CWSNP897 | Ca-Kabuli-Chr1 | 23149096 | [A/C] | Ca22359 | Non-Synonymous-CDS | Initiation factor2B-related |
| CWSNP898 | Ca-Kabuli-Chr1 | 23382483 | [T/A] | - | Intergenic | - |
| CWSNP899 | Ca-Kabuli-Chr1 | 23941438 | [G/A] | - | Intergenic | - |
| CWSNP900 | Ca-Kabuli-Chr1 | 24071014 | [T/G] | Ca19289 | Intron | AUX/IAA protein |
| CWSNP901 | Ca-Kabuli-Chr1 | 24173273 | [A/G] | - | Intergenic | - |
| CWSNP902 | Ca-Kabuli-Chr1 | 24450415 | [T/C] | Ca18343 | Intron | Ribulose-phosphate3-epimerase |
| CWSNP903 | Ca-Kabuli-Chr1 | 24450511 | [A/G] | Ca18343 | Intron | Ribulose-phosphate3-epimerase |
| CWSNP904 | Ca-Kabuli-Chr1 | 24450483 | [T/G] | Ca18343 | Intron | Ribulose-phosphate3-epimerase |
| CWSNP905 | Ca-Kabuli-Chr1 | 24693571 | [G/A] | - | DRR | - |
| CWSNP906 | Ca-Kabuli-Chr1 | 24693550 | [G/A] | - | DRR | - |
| CWSNP907 | Ca-Kabuli-Chr1 | 24693533 | [G/A] | - | DRR | - |
| CWSNP908 | Ca-Kabuli-Chr1 | 24693521 | [G/A] | - | DRR | - |
| CWSNP909 | Ca-Kabuli-Chr1 | 24828245 | [A/C] | - | DRR | - |
| CWSNP910 | Ca-Kabuli-Chr1 | 24845148 | [A/C] | Ca18325 | Synonymous-CDS | Zinc finger,RING-type |
| CWSNP911 | Ca-Kabuli-Chr1 | 25083482 | [A/C] | Ca18313 | Intron | Pectinesterase,catalytic |
| CWSNP912 | Ca-Kabuli-Chr1 | 25524382 | [C/T] | - | Intergenic | - |

| SNP IDs | Chromosomes | Physical positions (bp) | SNPs | Gene accession IDs | Sequence components of genes | Putative functions |
|---|---|---|---|---|---|---|
| CWSNP913 | Ca-Kabuli-Chr1 | 25524425 | [C/T] | - | Intergenic | - |
| CWSNP914 | Ca-Kabuli-Chr1 | 25524441 | [T/C] | - | Intergenic | - |
| CWSNP915 | Ca-Kabuli-Chr1 | 25524519 | [C/T] | - | Intergenic | - |
| CWSNP916 | Ca-Kabuli-Chr1 | 25524481 | [T/A] | - | Intergenic | - |
| CWSNP917 | Ca-Kabuli-Chr1 | 25524480 | [G/A] | - | Intergenic | - |
| CWSNP918 | Ca-Kabuli-Chr1 | 25524473 | [C/T] | - | Intergenic | - |
| CWSNP919 | Ca-Kabuli-Chr1 | 25524465 | [A/G] | - | Intergenic | - |
| CWSNP920 | Ca-Kabuli-Chr1 | 25524460 | [G/A] | - | Intergenic | - |
| CWSNP921 | Ca-Kabuli-Chr1 | 25524458 | [G/A] | - | Intergenic | - |
| CWSNP922 | Ca-Kabuli-Chr1 | 25524456 | [C/T] | - | Intergenic | - |
| CWSNP923 | Ca-Kabuli-Chr1 | 25524455 | [G/A] | - | Intergenic | - |
| CWSNP924 | Ca-Kabuli-Chr1 | 25524453 | [C/T] | - | Intergenic | - |
| CWSNP925 | Ca-Kabuli-Chr1 | 25524451 | [A/T] | - | Intergenic | - |
| CWSNP926 | Ca-Kabuli-Chr1 | 25524450 | [C/T] | - | Intergenic | - |
| CWSNP927 | Ca-Kabuli-Chr1 | 25524478 | [C/A] | - | Intergenic | - |
| CWSNP928 | Ca-Kabuli-Chr1 | 25524488 | [G/T] | - | Intergenic | - |

| SNP IDs | Chromosomes | Physical positions (bp) | SNPs | Gene accession IDs | Sequence components of genes | Putative functions |
|---|---|---|---|---|---|---|
| CWSNP929 | Ca-Kabuli-Chr1 | 25524505 | [A/G] | - | Intergenic | - |
| CWSNP930 | Ca-Kabuli-Chr1 | 25524520 | [C/T] | - | Intergenic | - |
| CWSNP931 | Ca-Kabuli-Chr1 | 25524524 | [C/T] | - | Intergenic | - |
| CWSNP932 | Ca-Kabuli-Chr1 | 25887539 | [G/A] | - | Intergenic | - |
| CWSNP933 | Ca-Kabuli-Chr1 | 26574146 | [C/T] | Ca18614 | Non-Synonymous-CDS | PeptidaseC48,SUMO/Sentrin/Ubl1 |
| CWSNP934 | Ca-Kabuli-Chr1 | 26579621 | [A/C] | - | Intergenic | - |
| CWSNP935 | Ca-Kabuli-Chr1 | 26807401 | [A/G] | - | URR | - |
| CWSNP936 | Ca-Kabuli-Chr1 | 26807395 | [T/G] | - | URR | - |
| CWSNP937 | Ca-Kabuli-Chr1 | 26856541 | [A/T] | Ca20289 | Non-Synonymous-CDS | - |
| CWSNP938 | Ca-Kabuli-Chr1 | 26856691 | [G/A] | Ca20289 | Synonymous-CDS | - |
| CWSNP939 | Ca-Kabuli-Chr1 | 26921471 | [C/T] | - | Intergenic | - |
| CWSNP940 | Ca-Kabuli-Chr1 | 26922011 | [T/G] | - | DRR | - |
| CWSNP941 | Ca-Kabuli-Chr1 | 26953535 | [C/T] | - | Intergenic | - |
| CWSNP942 | Ca-Kabuli-Chr1 | 27012495 | [C/T] | - | DRR | - |
| CWSNP943 | Ca-Kabuli-Chr1 | 27012660 | [G/C] | - | DRR | - |
| CWSNP944 | Ca-Kabuli-Chr1 | 27014895 | [T/G] | - | DRR | - |

| SNP IDs | Chromosomes | Physical positions (bp) | SNPs | Gene accession IDs | Sequence components of genes | Putative functions |
|---|---|---|---|---|---|---|
| CWSNP945 | Ca-Kabuli-Chr1 | 27014981 | [A/G] | - | DRR | - |
| CWSNP946 | Ca-Kabuli-Chr1 | 27014951 | [A/C] | - | DRR | - |
| CWSNP947 | Ca-Kabuli-Chr1 | 27016101 | [G/A] | - | DRR | - |
| CWSNP948 | Ca-Kabuli-Chr1 | 27016206 | [T/A] | - | DRR | - |
| CWSNP949 | Ca-Kabuli-Chr1 | 27017559 | [G/A] | Ca20299 | Intron | Cystathioninebeta-synthase,core |
| CWSNP950 | Ca-Kabuli-Chr1 | 27017560 | [G/A] | Ca20299 | Intron | Cystathioninebeta-synthase,core |
| CWSNP951 | Ca-Kabuli-Chr1 | 27017570 | [A/G] | Ca20299 | Intron | Cystathioninebeta-synthase,core |
| CWSNP952 | Ca-Kabuli-Chr1 | 27017604 | [T/G] | Ca20299 | Intron | Cystathioninebeta-synthase,core |
| CWSNP953 | Ca-Kabuli-Chr1 | 27017606 | [C/T] | Ca20299 | Intron | Cystathioninebeta-synthase,core |
| CWSNP954 | Ca-Kabuli-Chr1 | 27017617 | [G/A] | Ca20299 | Intron | Cystathioninebeta-synthase,core |
| CWSNP955 | Ca-Kabuli-Chr1 | 27017626 | [A/C] | Ca20299 | Intron | Cystathioninebeta-synthase,core |
| CWSNP956 | Ca-Kabuli-Chr1 | 27017628 | [C/A] | Ca20299 | Intron | Cystathioninebeta-synthase,core |
| CWSNP957 | Ca-Kabuli-Chr1 | 27017734 | [T/C] | Ca20299 | Intron | Cystathioninebeta-synthase,core |
| CWSNP958 | Ca-Kabuli-Chr1 | 27017765 | [A/T] | Ca20299 | Intron | Cystathioninebeta-synthase,core |
| CWSNP959 | Ca-Kabuli-Chr1 | 27017771 | [G/A] | Ca20299 | Intron | Cystathioninebeta-synthase,core |
| CWSNP960 | Ca-Kabuli-Chr1 | 27020193 | [T/C] | Ca20299 | Intron | Cystathioninebeta-synthase,core |

| SNP IDs | Chromosomes | Physical positions (bp) | SNPs | Gene accession IDs | Sequence components of genes | Putative functions |
|---|---|---|---|---|---|---|
| CWSNP961 | Ca-Kabuli-Chr1 | 27020354 | [C/A] | Ca20299 | Intron | Cystathioninebeta-synthase,core |
| CWSNP962 | Ca-Kabuli-Chr1 | 28088501 | [T/C] | Ca18523 | Synonymous-CDS | Vps54-like |
| CWSNP963 | Ca-Kabuli-Chr1 | 28106756 | [G/A] | - | Intergenic | - |
| CWSNP964 | Ca-Kabuli-Chr1 | 28133847 | [A/G] | Ca18521 | Intron | Leucine-rich repeat |
| CWSNP965 | Ca-Kabuli-Chr1 | 28133830 | [A/T] | Ca18521 | Intron | Leucine-rich repeat |
| CWSNP966 | Ca-Kabuli-Chr1 | 28133814 | [C/T] | Ca18521 | Intron | Leucine-rich repeat |
| CWSNP967 | Ca-Kabuli-Chr1 | 28143955 | [G/A] | Ca18521 | Intron | Leucine-rich repeat |
| CWSNP968 | Ca-Kabuli-Chr1 | 28152260 | [A/G] | Ca18521 | Intron | Leucine-rich repeat |
| CWSNP969 | Ca-Kabuli-Chr1 | 28656197 | [C/A] | - | Intergenic | - |
| CWSNP970 | Ca-Kabuli-Chr1 | 28907617 | [G/A] | Ca18486 | Non-Synonymous-CDS | Sugar/inositol transporter |
| CWSNP971 | Ca-Kabuli-Chr1 | 28909059 | [T/A] | Ca18486 | Non-Synonymous-CDS | Sugar/inositol transporter |
| CWSNP972 | Ca-Kabuli-Chr1 | 29020220 | [T/G] | - | Intergenic | - |
| CWSNP973 | Ca-Kabuli-Chr1 | 29460440 | [C/A] | Ca22559 | Non-Synonymous-CDS | - |
| CWSNP974 | Ca-Kabuli-Chr1 | 29637509 | [A/C] | Ca24382 | Intron | K Homology,type1 |
| CWSNP975 | Ca-Kabuli-Chr1 | 29637526 | [C/T] | Ca24382 | Intron | K Homology,type1 |
| CWSNP976 | Ca-Kabuli-Chr1 | 29637572 | [T/G] | Ca24382 | Intron | K Homology,type1 |

| SNP IDs | Chromosomes | Physical positions (bp) | SNPs | Gene accession IDs | Sequence components of genes | Putative functions |
|---|---|---|---|---|---|---|
| CWSNP977 | Ca-Kabuli-Chr1 | 29637571 | [T/G] | Ca24382 | Intron | K Homology,type1 |
| CWSNP978 | Ca-Kabuli-Chr1 | 30262124 | [A/G] | Ca22465 | Non-Synonymous-CDS | Kinesin , motor domain |
| CWSNP979 | Ca-Kabuli-Chr1 | 31080673 | [T/G] | Ca25827 | Intron | Enolase |
| CWSNP980 | Ca-Kabuli-Chr1 | 31080792 | [T/C] | Ca25827 | Intron | Enolase |
| CWSNP981 | Ca-Kabuli-Chr1 | 31240370 | [A/G] | - | Intergenic | - |
| CWSNP982 | Ca-Kabuli-Chr1 | 31369599 | [T/C] | Ca21855 | Intron | PeptidaseC13,legumain |
| CWSNP983 | Ca-Kabuli-Chr1 | 31590263 | [C/G] | Ca21868 | Intron | Orotidine5'-phosphate decarboxylase domain |
| CWSNP984 | Ca-Kabuli-Chr1 | 31590510 | [G/A] | Ca21868 | Synonymous-CDS | Orotidine5'-phosphate decarboxylase domain |
| CWSNP985 | Ca-Kabuli-Chr1 | 31590548 | [C/T] | Ca21868 | Non-Synonymous-CDS | Orotidine5'-phosphate decarboxylase domain |
| CWSNP986 | Ca-Kabuli-Chr1 | 32286538 | [C/T] | - | DRR | - |
| CWSNP987 | Ca-Kabuli-Chr1 | 32286534 | [G/C] | - | DRR | - |
| CWSNP988 | Ca-Kabuli-Chr1 | 32286518 | [C/A] | - | DRR | - |
| CWSNP989 | Ca-Kabuli-Chr1 | 32286517 | [G/A] | - | DRR | - |
| CWSNP990 | Ca-Kabuli-Chr1 | 32286469 | [G/A] | - | DRR | - |
| CWSNP991 | Ca-Kabuli-Chr1 | 32286466 | [A/G] | - | DRR | - |
| CWSNP992 | Ca-Kabuli-Chr1 | 32286568 | [A/C] | - | DRR | - |

| SNP IDs | Chromosomes | Physical positions (bp) | SNPs | Gene accession IDs | Sequence components of genes | Putative functions |
|---|---|---|---|---|---|---|
| CWSNP993 | Ca-Kabuli-Chr1 | 32286558 | [A/G] | - | DRR | - |
| CWSNP994 | Ca-Kabuli-Chr1 | 32286550 | [G/A] | - | DRR | - |
| CWSNP995 | Ca-Kabuli-Chr1 | 32286536 | [G/A] | - | DRR | - |
| CWSNP996 | Ca-Kabuli-Chr1 | 32286551 | [C/T] | - | DRR | - |
| CWSNP997 | Ca-Kabuli-Chr1 | 32286567 | [C/T] | - | DRR | - |
| CWSNP998 | Ca-Kabuli-Chr1 | 32468053 | [A/C] | - | Intergenic | - |
| CWSNP999 | Ca-Kabuli-Chr1 | 32468080 | [A/T] | - | Intergenic | - |
| CWSNP1000 | Ca-Kabuli-Chr1 | 32468105 | [C/G] | - | Intergenic | - |
| CWSNP1001 | Ca-Kabuli-Chr1 | 32468116 | [A/G] | - | Intergenic | - |
| CWSNP1002 | Ca-Kabuli-Chr1 | 32468119 | [T/C] | - | Intergenic | - |
| CWSNP1003 | Ca-Kabuli-Chr1 | 33058743 | [T/C] | Ca19437 | Intron | Argonaute/Dicerprotein,PAZ |
| CWSNP1004 | Ca-Kabuli-Chr1 | 33063207 | [C/T] | Ca19437 | Intron | Argonaute/Dicerprotein,PAZ |
| CWSNP1005 | Ca-Kabuli-Chr1 | 33194600 | [T/C] | Ca19440 | Intron | Helicase,C-terminal |
| CWSNP1006 | Ca-Kabuli-Chr1 | 33804152 | [C/T] | Ca19463 | Non-Synonymous-CDS | - |
| CWSNP1007 | Ca-Kabuli-Chr1 | 33804153 | [C/T] | Ca19463 | Synonymous-CDS | - |
| CWSNP1008 | Ca-Kabuli-Chr1 | 33804189 | [C/T] | Ca19463 | Non-Synonymous-CDS | - |

| SNP IDs | Chromosomes | Physical positions (bp) | SNPs | Gene accession IDs | Sequence components of genes | Putative functions |
|---|---|---|---|---|---|---|
| CWSNP1009 | Ca-Kabuli-Chr1 | 33804201 | [C/T] | Ca19463 | Non-Synonymous-CDS | - |
| CWSNP1010 | Ca-Kabuli-Chr1 | 33804232 | [T/G] | Ca19463 | Synonymous-CDS | - |
| CWSNP1011 | Ca-Kabuli-Chr1 | 33804247 | [A/G] | Ca19463 | Synonymous-CDS | - |
| CWSNP1012 | Ca-Kabuli-Chr1 | 33804262 | [C/G] | Ca19463 | Synonymous-CDS | - |
| CWSNP1013 | Ca-Kabuli-Chr1 | 34161921 | [G/A] | - | Intergenic | - |
| CWSNP1014 | Ca-Kabuli-Chr1 | 34261166 | [C/A] | Ca25475 | Intron | Glycosyltransferase,family48 |
| CWSNP1015 | Ca-Kabuli-Chr1 | 34261158 | [C/A] | Ca25475 | Intron | Glycosyltransferase,family48 |
| CWSNP1016 | Ca-Kabuli-Chr1 | 34311145 | [T/G] | Ca25478 | Synonymous-CDS | GTP-binding protein,HSR1-related |
| CWSNP1017 | Ca-Kabuli-Chr1 | 34727251 | [T/G] | Ca21419 | Synonymous-CDS | Zinc finger,CCCH-type |
| CWSNP1018 | Ca-Kabuli-Chr1 | 34772813 | [T/A] | Ca21416 | Non-Synonymous-CDS | - |
| CWSNP1019 | Ca-Kabuli-Chr1 | 34866709 | [C/A] | - | DRR | - |
| CWSNP1020 | Ca-Kabuli-Chr1 | 34948934 | [A/G] | Ca21408 | Intron | Proteasome,alpha-subunit,conserved site |
| CWSNP1021 | Ca-Kabuli-Chr1 | 35041241 | [T/G] | - | Intergenic | - |
| CWSNP1022 | Ca-Kabuli-Chr1 | 35041324 | [G/T] | - | Intergenic | - |
| CWSNP1023 | Ca-Kabuli-Chr1 | 35313277 | [A/G] | - | Intergenic | - |
| CWSNP1024 | Ca-Kabuli-Chr1 | 35315381 | [T/A] | - | Intergenic | - |

| SNP IDs | Chromosomes | Physical positions (bp) | SNPs | Gene accession IDs | Sequence components of genes | Putative functions |
|---|---|---|---|---|---|---|
| CWSNP1025 | Ca-Kabuli-Chr1 | 35315379 | [A/T] | - | Intergenic | - |
| CWSNP1026 | Ca-Kabuli-Chr1 | 35315347 | [C/T] | - | Intergenic | - |
| CWSNP1027 | Ca-Kabuli-Chr1 | 35315327 | [T/A] | - | Intergenic | - |
| CWSNP1028 | Ca-Kabuli-Chr1 | 35315436 | [G/A] | - | Intergenic | - |
| CWSNP1029 | Ca-Kabuli-Chr1 | 35708841 | [C/T] | Ca24110 | Synonymous-CDS | Mediator complex,subunit Med10 |
| CWSNP1030 | Ca-Kabuli-Chr1 | 36126090 | [C/T] | - | Intergenic | - |
| CWSNP1031 | Ca-Kabuli-Chr1 | 36794731 | [A/G] | Ca21700 | Non-Synonymous-CDS | PeptidaseC48,SUMO/Sentrin/Ubl1 |
| CWSNP1032 | Ca-Kabuli-Chr1 | 36794797 | [G/A] | Ca21700 | Non-Synonymous-CDS | PeptidaseC48,SUMO/Sentrin/Ubl1 |
| CWSNP1033 | Ca-Kabuli-Chr1 | 36794800 | [G/T] | Ca21700 | Non-Synonymous-CDS | PeptidaseC48,SUMO/Sentrin/Ubl1 |
| CWSNP1034 | Ca-Kabuli-Chr1 | 36794806 | [G/T] | Ca21700 | Non-Synonymous-CDS | PeptidaseC48,SUMO/Sentrin/Ubl1 |
| CWSNP1035 | Ca-Kabuli-Chr1 | 36794813 | [C/T] | Ca21700 | Synonymous-CDS | PeptidaseC48,SUMO/Sentrin/Ubl1 |
| CWSNP1036 | Ca-Kabuli-Chr1 | 36794887 | [G/A] | Ca21700 | Non-Synonymous-CDS | PeptidaseC48,SUMO/Sentrin/Ubl1 |
| CWSNP1037 | Ca-Kabuli-Chr1 | 36794870 | [C/T] | Ca21700 | Non-Synonymous-CDS | PeptidaseC48,SUMO/Sentrin/Ubl1 |
| CWSNP1038 | Ca-Kabuli-Chr1 | 36794906 | [A/C] | Ca21700 | Non-Synonymous-CDS | PeptidaseC48,SUMO/Sentrin/Ubl1 |
| CWSNP1039 | Ca-Kabuli-Chr1 | 36794912 | [G/T] | Ca21700 | Non-Synonymous-CDS | PeptidaseC48,SUMO/Sentrin/Ubl1 |
| CWSNP1040 | Ca-Kabuli-Chr1 | 37026302 | [C/T] | - | Intergenic | - |

| SNP IDs | Chromosomes | Physical positions (bp) | SNPs | Gene accession IDs | Sequence components of genes | Putative functions |
|---|---|---|---|---|---|---|
| CWSNP1041 | Ca-Kabuli-Chr1 | 37642546 | [G/A] | - | URR | - |
| CWSNP1042 | Ca-Kabuli-Chr1 | 37690416 | [G/A] | Ca23533 | Intron | Myb,DNA-binding |
| CWSNP1043 | Ca-Kabuli-Chr1 | 37690465 | [T/C] | Ca23533 | Intron | Myb,DNA-binding |
| CWSNP1044 | Ca-Kabuli-Chr1 | 37690498 | [C/A] | Ca23533 | Intron | Myb,DNA-binding |
| CWSNP1045 | Ca-Kabuli-Chr1 | 37690497 | [A/T] | Ca23533 | Intron | Myb,DNA-binding |
| CWSNP1046 | Ca-Kabuli-Chr1 | 37699039 | [T/G] | - | Intergenic | - |
| CWSNP1047 | Ca-Kabuli-Chr1 | 37699036 | [T/C] | - | Intergenic | - |
| CWSNP1048 | Ca-Kabuli-Chr1 | 37722095 | [G/A] | Ca23534 | Synonymous-CDS | Protein of unknown function DUF620 |
| CWSNP1049 | Ca-Kabuli-Chr1 | 37722143 | [G/C] | Ca23534 | Synonymous-CDS | Protein of unknown function DUF620 |
| CWSNP1050 | Ca-Kabuli-Chr1 | 37768179 | [T/A] | - | Intergenic | - |
| CWSNP1051 | Ca-Kabuli-Chr1 | 37768333 | [C/T] | - | Intergenic | - |
| CWSNP1052 | Ca-Kabuli-Chr1 | 37768299 | [A/G] | - | Intergenic | - |
| CWSNP1053 | Ca-Kabuli-Chr1 | 37880995 | [T/C] | Ca23539 | Intron | Zinc finger,PHD-type |
| CWSNP1054 | Ca-Kabuli-Chr1 | 37892088 | [A/C] | - | Intergenic | - |
| CWSNP1055 | Ca-Kabuli-Chr1 | 37892089 | [C/T] | - | Intergenic | - |
| CWSNP1056 | Ca-Kabuli-Chr1 | 37892139 | [A/T] | - | Intergenic | - |

| SNP IDs | Chromosomes | Physical positions (bp) | SNPs | Gene accession IDs | Sequence components of genes | Putative functions |
|---|---|---|---|---|---|---|
| CWSNP1057 | Ca-Kabuli-Chr1 | 38049721 | [C/T] | Ca27444 | Non-Synonymous-CDS | Cation efflux protein |
| CWSNP1058 | Ca-Kabuli-Chr1 | 38877670 | [T/C] | - | Intergenic | - |
| CWSNP1059 | Ca-Kabuli-Chr1 | 39436291 | [T/C] | Ca13931 | Intron | IQ motif, EF-hand binding site |
| CWSNP1060 | Ca-Kabuli-Chr1 | 39601982 | [T/G] | - | Intergenic | - |
| CWSNP1061 | Ca-Kabuli-Chr1 | 39602029 | [G/T] | - | Intergenic | - |
| CWSNP1062 | Ca-Kabuli-Chr1 | 39616139 | [A/C] | Ca13924 | Synonymous-CDS | CTPsynthase,N-terminal |
| CWSNP1063 | Ca-Kabuli-Chr1 | 39663432 | [C/G] | Ca13922 | Intron | ABC transporter-like |
| CWSNP1064 | Ca-Kabuli-Chr1 | 40224835 | [T/A] | - | Intergenic | - |
| CWSNP1065 | Ca-Kabuli-Chr1 | 40224834 | [C/G] | - | Intergenic | - |
| CWSNP1066 | Ca-Kabuli-Chr1 | 40224804 | [A/G] | - | Intergenic | - |
| CWSNP1067 | Ca-Kabuli-Chr1 | 40343216 | [G/T] | Ca13893 | Intron | Tetratricopeptide,MLP1/MLP2-like |
| CWSNP1068 | Ca-Kabuli-Chr1 | 40346280 | [A/C] | Ca13893 | Intron | Tetratricopeptide,MLP1/MLP2-like |
| CWSNP1069 | Ca-Kabuli-Chr1 | 40358693 | [C/T] | Ca13893 | Intron | Tetratricopeptide,MLP1/MLP2-like |
| CWSNP1070 | Ca-Kabuli-Chr1 | 40358747 | [T/G] | Ca13893 | Intron | Tetratricopeptide,MLP1/MLP2-like |
| CWSNP1071 | Ca-Kabuli-Chr1 | 40367940 | [G/A] | Ca13893 | Intron | Tetratricopeptide,MLP1/MLP2-like |
| CWSNP1072 | Ca-Kabuli-Chr1 | 41029732 | [C/T] | - | Intergenic | - |

| SNP IDs | Chromosomes | Physical positions (bp) | SNPs | Gene accession IDs | Sequence components of genes | Putative functions |
|---|---|---|---|---|---|---|
| CWSNP1073 | Ca-Kabuli-Chr1 | 41169218 | [G/T] | - | Intergenic | - |
| CWSNP1074 | Ca-Kabuli-Chr1 | 41185512 | [T/G] | - | Intergenic | - |
| CWSNP1075 | Ca-Kabuli-Chr1 | 41380061 | [G/T] | Ca23111 | Synonymous-CDS | GlutathioneS-transferase,N-terminal |
| CWSNP1076 | Ca-Kabuli-Chr1 | 41564809 | [T/C] | - | URR | - |
| CWSNP1077 | Ca-Kabuli-Chr1 | 41827388 | [T/C] | - | DRR | - |
| CWSNP1078 | Ca-Kabuli-Chr1 | 41867035 | [C/T] | - | URR | - |
| CWSNP1079 | Ca-Kabuli-Chr1 | 41897216 | [C/A] | Ca22528 | Intron | - |
| CWSNP1080 | Ca-Kabuli-Chr1 | 41946005 | [A/G] | - | Intergenic | - |
| CWSNP1081 | Ca-Kabuli-Chr1 | 42452939 | [G/T] | Ca13620 | Non-Synonymous-CDS | Transcription factorCBF/NF-Y/archaealhistone |
| CWSNP1082 | Ca-Kabuli-Chr1 | 42551585 | [C/A] | - | DRR | - |
| CWSNP1083 | Ca-Kabuli-Chr1 | 42552658 | [A/C] | - | Intergenic | - |
| CWSNP1084 | Ca-Kabuli-Chr1 | 42605239 | [T/G] | - | DRR | - |
| CWSNP1085 | Ca-Kabuli-Chr1 | 42613060 | [T/G] | - | DRR | - |
| CWSNP1086 | Ca-Kabuli-Chr1 | 43053424 | [G/C] | - | Intergenic | - |
| CWSNP1087 | Ca-Kabuli-Chr1 | 43273910 | [G/T] | - | Intergenic | - |
| CWSNP1088 | Ca-Kabuli-Chr1 | 43273922 | [T/C] | - | Intergenic | - |

| SNP IDs | Chromosomes | Physical positions (bp) | SNPs | Gene accession IDs | Sequence components of genes | Putative functions |
|---|---|---|---|---|---|---|
| CWSNP1089 | Ca-Kabuli-Chr1 | 43273925 | [A/G] | - | Intergenic | - |
| CWSNP1090 | Ca-Kabuli-Chr1 | 43273931 | [G/A] | - | Intergenic | - |
| CWSNP1091 | Ca-Kabuli-Chr1 | 43273941 | [C/G] | - | Intergenic | - |
| CWSNP1092 | Ca-Kabuli-Chr1 | 43273943 | [A/G] | - | Intergenic | - |
| CWSNP1093 | Ca-Kabuli-Chr1 | 43273961 | [C/T] | - | Intergenic | - |
| CWSNP1094 | Ca-Kabuli-Chr1 | 43273962 | [A/G] | - | Intergenic | - |
| CWSNP1095 | Ca-Kabuli-Chr1 | 43273968 | [T/G] | - | Intergenic | - |
| CWSNP1096 | Ca-Kabuli-Chr1 | 43273972 | [C/T] | - | Intergenic | - |
| CWSNP1097 | Ca-Kabuli-Chr1 | 43273981 | [G/T] | - | Intergenic | - |
| CWSNP1098 | Ca-Kabuli-Chr1 | 43273985 | [G/A] | - | Intergenic | - |
| CWSNP1099 | Ca-Kabuli-Chr1 | 43792565 | [G/A] | - | Intergenic | - |
| CWSNP1100 | Ca-Kabuli-Chr1 | 43930143 | [A/T] | - | Intergenic | - |
| CWSNP1101 | Ca-Kabuli-Chr1 | 43930138 | [T/C] | - | Intergenic | - |
| CWSNP1102 | Ca-Kabuli-Chr1 | 44085069 | [C/T] | Ca13530 | Intron | Endosulphine |
| CWSNP1103 | Ca-Kabuli-Chr1 | 44349004 | [T/C] | Ca23498 | Non-Synonymous-CDS | RibonucleaseII/R |
| CWSNP1104 | Ca-Kabuli-Chr1 | 44350953 | [G/T] | Ca23498 | Intron | RibonucleaseII/R |

| SNP IDs | Chromosomes | Physical positions (bp) | SNPs | Gene accession IDs | Sequence components of genes | Putative functions |
|---|---|---|---|---|---|---|
| CWSNP1105 | Ca-Kabuli-Chr1 | 44635939 | [T/C] | - | Intergenic | - |
| CWSNP1106 | Ca-Kabuli-Chr1 | 44635926 | [A/G] | - | Intergenic | - |
| CWSNP1107 | Ca-Kabuli-Chr1 | 44635893 | [A/G] | - | Intergenic | - |
| CWSNP1108 | Ca-Kabuli-Chr1 | 44976738 | [T/A] | Ca25713 | Intron | Protein kinase, catalytic domain |
| CWSNP1109 | Ca-Kabuli-Chr1 | 44976735 | [C/T] | Ca25713 | Intron | Protein kinase, catalytic domain |
| CWSNP1110 | Ca-Kabuli-Chr1 | 44988344 | [A/G] | - | Intergenic | - |
| CWSNP1111 | Ca-Kabuli-Chr1 | 45003190 | [G/A] | Ca25714 | Non-Synonymous-CDS | - |
| CWSNP1112 | Ca-Kabuli-Chr1 | 45003514 | [G/A] | Ca25714 | Non-Synonymous-CDS | - |
| CWSNP1113 | Ca-Kabuli-Chr1 | 45003509 | [C/A] | Ca25714 | Intron | - |
| CWSNP1114 | Ca-Kabuli-Chr1 | 45003505 | [A/C] | Ca25714 | Intron | - |
| CWSNP1115 | Ca-Kabuli-Chr1 | 45003938 | [A/G] | Ca25714 | Intron | - |
| CWSNP1116 | Ca-Kabuli-Chr1 | 45003939 | [T/A] | Ca25714 | Intron | - |
| CWSNP1117 | Ca-Kabuli-Chr1 | 45003940 | [T/G] | Ca25714 | Intron | - |
| CWSNP1118 | Ca-Kabuli-Chr1 | 45003944 | [C/T] | Ca25714 | Intron | - |
| CWSNP1119 | Ca-Kabuli-Chr1 | 45003945 | [T/A] | Ca25714 | Intron | - |
| CWSNP1120 | Ca-Kabuli-Chr1 | 45003946 | [T/G] | Ca25714 | Intron | - |

| SNP IDs | Chromosomes | Physical positions (bp) | SNPs | Gene accession IDs | Sequence components of genes | Putative functions |
|---|---|---|---|---|---|---|
| CWSNP1121 | Ca-Kabuli-Chr1 | 45003947 | [C/A] | Ca25714 | Intron | - |
| CWSNP1122 | Ca-Kabuli-Chr1 | 45003973 | [T/C] | Ca25714 | Intron | - |
| CWSNP1123 | Ca-Kabuli-Chr1 | 45003985 | [G/T] | Ca25714 | Intron | - |
| CWSNP1124 | Ca-Kabuli-Chr1 | 45003998 | [G/A] | Ca25714 | Intron | - |
| CWSNP1125 | Ca-Kabuli-Chr1 | 45004001 | [C/T] | Ca25714 | Intron | - |
| CWSNP1126 | Ca-Kabuli-Chr1 | 45007032 | [A/C] | Ca25714 | Intron | - |
| CWSNP1127 | Ca-Kabuli-Chr1 | 45007227 | [T/C] | Ca25714 | Intron | - |
| CWSNP1128 | Ca-Kabuli-Chr1 | 45007259 | [A/G] | Ca25714 | Intron | - |
| CWSNP1129 | Ca-Kabuli-Chr1 | 45007331 | [C/T] | Ca25714 | Intron | - |
| CWSNP1130 | Ca-Kabuli-Chr1 | 45008076 | [G/A] | Ca25714 | Intron | - |
| CWSNP1131 | Ca-Kabuli-Chr1 | 45008075 | [T/C] | Ca25714 | Intron | - |
| CWSNP1132 | Ca-Kabuli-Chr1 | 45008073 | [T/G] | Ca25714 | Intron | - |
| CWSNP1133 | Ca-Kabuli-Chr1 | 45008071 | [G/C] | Ca25714 | Intron | - |
| CWSNP1134 | Ca-Kabuli-Chr1 | 45008050 | [C/G] | Ca25714 | Intron | - |
| CWSNP1135 | Ca-Kabuli-Chr1 | 45008044 | [T/C] | Ca25714 | Intron | - |
| CWSNP1136 | Ca-Kabuli-Chr1 | 45008016 | [T/A] | Ca25714 | Intron | - |

| SNP IDs | Chromosomes | Physical positions (bp) | SNPs | Gene accession IDs | Sequence components of genes | Putative functions |
|---|---|---|---|---|---|---|
| CWSNP1137 | Ca-Kabuli-Chr1 | 45008015 | [G/A] | Ca25714 | Intron | - |
| CWSNP1138 | Ca-Kabuli-Chr1 | 45008009 | [C/T] | Ca25714 | Intron | - |
| CWSNP1139 | Ca-Kabuli-Chr1 | 45008006 | [C/A] | Ca25714 | Intron | - |
| CWSNP1140 | Ca-Kabuli-Chr1 | 45008004 | [C/T] | Ca25714 | Intron | - |
| CWSNP1141 | Ca-Kabuli-Chr1 | 45250874 | [G/A] | - | URR | - |
| CWSNP1142 | Ca-Kabuli-Chr1 | 45250872 | [G/T] | - | URR | - |
| CWSNP1143 | Ca-Kabuli-Chr1 | 45250869 | [G/A] | - | URR | - |
| CWSNP1144 | Ca-Kabuli-Chr1 | 45250863 | [C/T] | - | URR | - |
| CWSNP1145 | Ca-Kabuli-Chr1 | 45250862 | [A/T] | - | URR | - |
| CWSNP1146 | Ca-Kabuli-Chr1 | 45250834 | [A/G] | - | URR | - |
| CWSNP1147 | Ca-Kabuli-Chr1 | 45250828 | [G/C] | - | URR | - |
| CWSNP1148 | Ca-Kabuli-Chr1 | 45250807 | [C/G] | - | URR | - |
| CWSNP1149 | Ca-Kabuli-Chr1 | 45250805 | [A/C] | - | URR | - |
| CWSNP1150 | Ca-Kabuli-Chr1 | 45250803 | [A/G] | - | URR | - |
| CWSNP1151 | Ca-Kabuli-Chr1 | 45250802 | [C/T] | - | URR | - |
| CWSNP1152 | Ca-Kabuli-Chr1 | 46437023 | [T/G] | Ca21547 | Synonymous-CDS | Adenylatekinase |

| SNP IDs | Chromosomes | Physical positions (bp) | SNPs | Gene accession IDs | Sequence components of genes | Putative functions |
|---|---|---|---|---|---|---|
| CWSNP1153 | Ca-Kabuli-Chr1 | 46523221 | [C/T] | Ca21550 | Synonymous-CDS | - |
| CWSNP1154 | Ca-Kabuli-Chr1 | 46597131 | [T/G] | - | Intergenic | - |
| CWSNP1155 | Ca-Kabuli-Chr1 | 46745628 | [C/A] | Ca25111 | Intron | Protein of unknown function DUF3754 |
| CWSNP1156 | Ca-Kabuli-Chr1 | 46778814 | [A/C] | Ca25114 | Non-Synonymous-CDS | Protein kinase, catalytic domain |
| CWSNP1157 | Ca-Kabuli-Chr1 | 46791939 | [T/G] | - | Intergenic | - |
| CWSNP1158 | Ca-Kabuli-Chr1 | 46793357 | [T/C] | - | Intergenic | - |
| CWSNP1159 | Ca-Kabuli-Chr1 | 46816041 | [A/T] | - | Intergenic | - |
| CWSNP1160 | Ca-Kabuli-Chr1 | 46825211 | [G/A] | Ca25116 | Intron | - |
| CWSNP1161 | Ca-Kabuli-Chr1 | 46829662 | [C/T] | Ca25116 | Intron | - |
| CWSNP1162 | Ca-Kabuli-Chr1 | 46832306 | [T/G] | Ca25116 | Intron | - |
| CWSNP1163 | Ca-Kabuli-Chr1 | 46832395 | [A/G] | Ca25116 | Intron | - |
| CWSNP1164 | Ca-Kabuli-Chr1 | 46832383 | [T/C] | Ca25116 | Intron | - |
| CWSNP1165 | Ca-Kabuli-Chr1 | 46920524 | [C/T] | Ca12949 | Intron | - |
| CWSNP1166 | Ca-Kabuli-Chr1 | 46936201 | [C/T] | - | URR | - |
| CWSNP1167 | Ca-Kabuli-Chr1 | 46969221 | [T/A] | Ca12942 | Intron | Glycosyltransferase,family20 |
| CWSNP1168 | Ca-Kabuli-Chr1 | 47042798 | [T/A] | Ca12938 | Non-Synonymous-CDS | Protein of unknown function DUF92,TMEM19 |

| SNP IDs | Chromosomes | Physical positions (bp) | SNPs | Gene accession IDs | Sequence components of genes | Putative functions |
|---|---|---|---|---|---|---|
| CWSNP1169 | Ca-Kabuli-Chr1 | 47331082 | [T/C] | - | Intergenic | - |
| CWSNP1170 | Ca-Kabuli-Chr1 | 47331080 | [T/C] | - | Intergenic | - |
| CWSNP1171 | Ca-Kabuli-Chr1 | 47897914 | [C/T] | Ca12887 | Intron | GPImannosyltransferase |
| CWSNP1172 | Ca-Kabuli-Chr1 | 47938884 | [T/G] | Ca12883 | Intron | Ubiquitin-conjugating enzyme,E2 |
| CWSNP1173 | Ca-Kabuli-Chr1 | 48026664 | [G/T] | - | Intergenic | - |
| CWSNP1174 | Ca-Kabuli-Chr1 | 48026727 | [A/C] | - | Intergenic | - |
| CWSNP1175 | Ca-Kabuli-Chr1 | 48028094 | [G/A] | - | URR | - |
| CWSNP1176 | Ca-Kabuli-Chr1 | 48132521 | [A/C] | - | DRR | - |
| CWSNP1177 | Ca-Kabuli-Chr1 | 48265590 | [T/G] | Ca12856 | Synonymous-CDS | - |
| CWSNP1178 | Ca-Kabuli-Chr2 | 233589 | [C/T] | - | Intergenic | - |
| CWSNP1179 | Ca-Kabuli-Chr2 | 522943 | [A/G] | - | URR | - |
| CWSNP1180 | Ca-Kabuli-Chr2 | 737183 | [T/G] | Ca17000 | Synonymous-CDS | - |
| CWSNP1181 | Ca-Kabuli-Chr2 | 770164 | [A/C] | Ca17001 | Synonymous-CDS | PeptidaseC48,SUMO/Sentrin/Ubl1 |
| CWSNP1182 | Ca-Kabuli-Chr2 | 770183 | [C/T] | Ca17001 | Non-Synonymous-CDS | PeptidaseC48,SUMO/Sentrin/Ubl1 |
| CWSNP1183 | Ca-Kabuli-Chr2 | 770234 | [C/T] | Ca17001 | Non-Synonymous-CDS | PeptidaseC48,SUMO/Sentrin/Ubl1 |
| CWSNP1184 | Ca-Kabuli-Chr2 | 770237 | [C/T] | Ca17001 | Non-Synonymous-CDS | PeptidaseC48,SUMO/Sentrin/Ubl1 |

| **SNP IDs** | **Chromosomes** | **Physical positions (bp)** | **SNPs** | **Gene accession IDs** | **Sequence components of genes** | **Putative functions** |
|---|---|---|---|---|---|---|
| CWSNP1185 | Ca-Kabuli-Chr2 | 770225 | [C/T] | Ca17001 | Non-Synonymous-CDS | PeptidaseC48,SUMO/Sentrin/Ubl1 |
| CWSNP1186 | Ca-Kabuli-Chr2 | 770260 | [C/T] | Ca17001 | Non-Synonymous-CDS | PeptidaseC48,SUMO/Sentrin/Ubl1 |
| CWSNP1187 | Ca-Kabuli-Chr2 | 770266 | [C/A] | Ca17001 | Non-Synonymous-CDS | PeptidaseC48,SUMO/Sentrin/Ubl1 |
| CWSNP1188 | Ca-Kabuli-Chr2 | 1231500 | [A/G] | - | Intergenic | - |
| CWSNP1189 | Ca-Kabuli-Chr2 | 1231618 | [G/A] | - | Intergenic | - |
| CWSNP1190 | Ca-Kabuli-Chr2 | 1234932 | [C/T] | - | Intergenic | - |
| CWSNP1191 | Ca-Kabuli-Chr2 | 1234983 | [T/C] | - | Intergenic | - |
| CWSNP1192 | Ca-Kabuli-Chr2 | 1368911 | [A/G] | - | URR | - |
| CWSNP1193 | Ca-Kabuli-Chr2 | 1389661 | [A/G] | - | URR | - |
| CWSNP1194 | Ca-Kabuli-Chr2 | 1493971 | [A/G] | - | Intergenic | - |
| CWSNP1195 | Ca-Kabuli-Chr2 | 1610477 | [G/T] | Ca12629 | Non-Synonymous-CDS | Diacylglycerol kinase,catalytic domain |
| CWSNP1196 | Ca-Kabuli-Chr2 | 1668116 | [A/G] | - | Intergenic | - |
| CWSNP1197 | Ca-Kabuli-Chr2 | 1713343 | [T/C] | - | Intergenic | - |
| CWSNP1198 | Ca-Kabuli-Chr2 | 1828406 | [G/A] | Ca12602 | Non-Synonymous-CDS | Protein of unknown function DUF1421 |
| CWSNP1199 | Ca-Kabuli-Chr2 | 1867041 | [C/A] | Ca12598 | Intron | BacterialIg-like,group2 |
| CWSNP1200 | Ca-Kabuli-Chr2 | 2230056 | [T/C] | - | DRR | - |

| SNP IDs | Chromosomes | Physical positions (bp) | SNPs | Gene accession IDs | Sequence components of genes | Putative functions |
|---|---|---|---|---|---|---|
| CWSNP1201 | Ca-Kabuli-Chr2 | 2311915 | [C/A] | - | URR | - |
| CWSNP1202 | Ca-Kabuli-Chr2 | 2311917 | [A/C] | - | URR | - |
| CWSNP1203 | Ca-Kabuli-Chr2 | 2312104 | [C/T] | - | URR | - |
| CWSNP1204 | Ca-Kabuli-Chr2 | 2312037 | [T/G] | - | URR | - |
| CWSNP1205 | Ca-Kabuli-Chr2 | 2344777 | [G/A] | Ca12544 | Synonymous-CDS | Oligopeptide transporter |
| CWSNP1206 | Ca-Kabuli-Chr2 | 2344778 | [A/G] | Ca12544 | Non-Synonymous-CDS | Oligopeptide transporter |
| CWSNP1207 | Ca-Kabuli-Chr2 | 2344786 | [G/A] | Ca12544 | Synonymous-CDS | Oligopeptide transporter |
| CWSNP1208 | Ca-Kabuli-Chr2 | 2344835 | [G/T] | Ca12544 | Non-Synonymous-CDS | Oligopeptide transporter |
| CWSNP1209 | Ca-Kabuli-Chr2 | 2416422 | [C/T] | - | DRR | - |
| CWSNP1210 | Ca-Kabuli-Chr2 | 2650375 | [T/C] | - | URR | - |
| CWSNP1211 | Ca-Kabuli-Chr2 | 2650373 | [A/T] | - | URR | - |
| CWSNP1212 | Ca-Kabuli-Chr2 | 2697688 | [G/C] | Ca10574 | Non-Synonymous-CDS | RNA-binding,CRM domain |
| CWSNP1213 | Ca-Kabuli-Chr2 | 2708805 | [G/C] | - | URR | - |
| CWSNP1214 | Ca-Kabuli-Chr2 | 2783727 | [G/A] | - | DRR | - |
| CWSNP1215 | Ca-Kabuli-Chr2 | 2846667 | [T/C] | Ca10559 | Intron | Phospho glucose isomerase(PGI) |
| CWSNP1216 | Ca-Kabuli-Chr2 | 2846774 | [C/A] | Ca10559 | Non-Synonymous-CDS | Phospho glucose isomerase(PGI) |

| SNP IDs | Chromosomes | Physical positions (bp) | SNPs | Gene accession IDs | Sequence components of genes | Putative functions |
|---|---|---|---|---|---|---|
| CWSNP1217 | Ca-Kabuli-Chr2 | 2965867 | [G/A] | - | DRR | - |
| CWSNP1218 | Ca-Kabuli-Chr2 | 3100116 | [A/G] | - | DRR | - |
| CWSNP1219 | Ca-Kabuli-Chr2 | 3113560 | [G/A] | Ca10538 | Intron | Auxin efflux carrier |
| CWSNP1220 | Ca-Kabuli-Chr2 | 3141128 | [A/C] | - | Intergenic | - |
| CWSNP1221 | Ca-Kabuli-Chr2 | 3164404 | [A/G] | - | Intergenic | - |
| CWSNP1222 | Ca-Kabuli-Chr2 | 3164355 | [G/A] | - | Intergenic | - |
| CWSNP1223 | Ca-Kabuli-Chr2 | 3450904 | [C/A] | Ca10514 | Synonymous-CDS | ZF-HD homeobox protein,Cys/His-rich dimerisation domain |
| CWSNP1224 | Ca-Kabuli-Chr2 | 3503763 | [T/G] | - | DRR | - |
| CWSNP1225 | Ca-Kabuli-Chr2 | 3503759 | [T/A] | - | DRR | - |
| CWSNP1226 | Ca-Kabuli-Chr2 | 3503753 | [C/G] | - | DRR | - |
| CWSNP1227 | Ca-Kabuli-Chr2 | 3503750 | [C/G] | - | DRR | - |
| CWSNP1228 | Ca-Kabuli-Chr2 | 3789808 | [G/T] | - | DRR | - |
| CWSNP1229 | Ca-Kabuli-Chr2 | 3941397 | [A/T] | - | Intergenic | - |
| CWSNP1230 | Ca-Kabuli-Chr2 | 3994150 | [A/C] | - | Intergenic | - |
| CWSNP1231 | Ca-Kabuli-Chr2 | 4039854 | [G/A] | - | Intergenic | - |
| CWSNP1232 | Ca-Kabuli-Chr2 | 4404813 | [C/T] | Ca21106 | Intron | Glycosylhydrolase,family13,catalytic domain |

| SNP IDs | Chromosomes | Physical positions (bp) | SNPs | Gene accession IDs | Sequence components of genes | Putative functions |
|---|---|---|---|---|---|---|
| CWSNP1233 | Ca-Kabuli-Chr2 | 4966216 | [C/T] | Ca14714 | Non-Synonymous-CDS | DNAligase,ATP-dependent,N-terminal |
| CWSNP1234 | Ca-Kabuli-Chr2 | 4981626 | [A/G] | - | DRR | - |
| CWSNP1235 | Ca-Kabuli-Chr2 | 4981666 | [G/C] | - | DRR | - |
| CWSNP1236 | Ca-Kabuli-Chr2 | 5079639 | [C/T] | Ca14702 | Non-Synonymous-CDS | Zinc finger,RING-type |
| CWSNP1237 | Ca-Kabuli-Chr2 | 5247557 | [A/C] | - | Intergenic | - |
| CWSNP1238 | Ca-Kabuli-Chr2 | 5247558 | [G/T] | - | Intergenic | - |
| CWSNP1239 | Ca-Kabuli-Chr2 | 5259254 | [T/C] | Ca14682 | Non-Synonymous-CDS | - |
| CWSNP1240 | Ca-Kabuli-Chr2 | 5342864 | [C/A] | - | Intergenic | - |
| CWSNP1241 | Ca-Kabuli-Chr2 | 5342923 | [A/G] | - | Intergenic | - |
| CWSNP1242 | Ca-Kabuli-Chr2 | 5449978 | [T/C] | - | URR | - |
| CWSNP1243 | Ca-Kabuli-Chr2 | 5475344 | [A/G] | Ca14660 | Intron | Proteasome component (PCI) domain |
| CWSNP1244 | Ca-Kabuli-Chr2 | 5787862 | [G/A] | Ca19683 | Synonymous-CDS | Plant specific mitochondrial import receptor subunit TOM20 |
| CWSNP1245 | Ca-Kabuli-Chr2 | 5787887 | [A/C] | Ca19683 | Intron | Plant specific mitochondrial import receptor subunit TOM20 |
| CWSNP1246 | Ca-Kabuli-Chr2 | 6162378 | [T/G] | Ca19714 | Intron | Amino acid/polyamine transporter I |
| CWSNP1247 | Ca-Kabuli-Chr2 | 6213582 | [T/A] | Ca20925 | Non-Synonymous-CDS | - |
| CWSNP1248 | Ca-Kabuli-Chr2 | 6213566 | [C/T] | Ca20925 | Non-Synonymous-CDS | - |

| SNP IDs | Chromosomes | Physical positions (bp) | SNPs | Gene accession IDs | Sequence components of genes | Putative functions |
|---|---|---|---|---|---|---|
| CWSNP1249 | Ca-Kabuli-Chr2 | 6213530 | [C/A] | Ca20925 | Non-Synonymous-CDS | - |
| CWSNP1250 | Ca-Kabuli-Chr2 | 6213520 | [G/T] | Ca20925 | Non-Synonymous-CDS | - |
| CWSNP1251 | Ca-Kabuli-Chr2 | 6349208 | [T/G] | Ca20931 | Intron | Helicase/RNaseDC-terminal,HRDCdomain |
| CWSNP1252 | Ca-Kabuli-Chr2 | 6367704 | [A/G] | - | DRR | - |
| CWSNP1253 | Ca-Kabuli-Chr2 | 6408406 | [C/A] | Ca20937 | Intron | Double-stranded RNA-binding |
| CWSNP1254 | Ca-Kabuli-Chr2 | 6534776 | [C/A] | Ca20948 | Intron | TargetSNAREcoiled-coildomain |
| CWSNP1255 | Ca-Kabuli-Chr2 | 6728716 | [A/T] | Ca19158 | Intron | Glutamate/phenylalanine/leucine/valine dehydrogenase |
| CWSNP1256 | Ca-Kabuli-Chr2 | 6728719 | [C/T] | Ca19158 | Intron | Glutamate/phenylalanine/leucine/valine dehydrogenase |
| CWSNP1257 | Ca-Kabuli-Chr2 | 7027093 | [G/A] | Ca19136 | Intron | RNA recognition motif domain |
| CWSNP1258 | Ca-Kabuli-Chr2 | 7125992 | [T/G] | Ca19128 | Non-Synonymous-CDS | Oxidoreductase,N-terminal |
| CWSNP1259 | Ca-Kabuli-Chr2 | 7507259 | [C/A] | Ca18111 | Non-Synonymous-CDS | Peptidase C19,ubiquitin carboxyl-terminalhydrolase 2 |
| CWSNP1260 | Ca-Kabuli-Chr2 | 7633520 | [G/A] | - | Intergenic | - |
| CWSNP1261 | Ca-Kabuli-Chr2 | 7633547 | [C/T] | - | Intergenic | - |
| CWSNP1262 | Ca-Kabuli-Chr2 | 7633564 | [G/T] | - | Intergenic | - |
| CWSNP1263 | Ca-Kabuli-Chr2 | 7633572 | [T/C] | - | Intergenic | - |
| CWSNP1264 | Ca-Kabuli-Chr2 | 7633592 | [G/A] | - | Intergenic | - |

| SNP IDs | Chromosomes | Physical positions (bp) | SNPs | Gene accession IDs | Sequence components of genes | Putative functions |
|---|---|---|---|---|---|---|
| CWSNP1265 | Ca-Kabuli-Chr2 | 7633585 | [G/A] | - | Intergenic | - |
| CWSNP1266 | Ca-Kabuli-Chr2 | 7633869 | [G/A] | - | Intergenic | - |
| CWSNP1267 | Ca-Kabuli-Chr2 | 7633826 | [G/A] | - | Intergenic | - |
| CWSNP1268 | Ca-Kabuli-Chr2 | 7633818 | [G/A] | - | Intergenic | - |
| CWSNP1269 | Ca-Kabuli-Chr2 | 7633816 | [G/A] | - | Intergenic | - |
| CWSNP1270 | Ca-Kabuli-Chr2 | 7633813 | [G/T] | - | Intergenic | - |
| CWSNP1271 | Ca-Kabuli-Chr2 | 7633793 | [C/T] | - | Intergenic | - |
| CWSNP1272 | Ca-Kabuli-Chr2 | 7679627 | [A/T] | Ca18121 | Non-Synonymous-CDS | Multiantimicrobial extrusion protein |
| CWSNP1273 | Ca-Kabuli-Chr2 | 7679693 | [C/G] | Ca18121 | Non-Synonymous-CDS | Multiantimicrobial extrusion protein |
| CWSNP1274 | Ca-Kabuli-Chr2 | 7701189 | [T/G] | Ca18123 | Non-Synonymous-CDS | Multiantimicrobial extrusion protein |
| CWSNP1275 | Ca-Kabuli-Chr2 | 7701199 | [T/G] | Ca18123 | Non-Synonymous-CDS | Multiantimicrobial extrusion protein |
| CWSNP1276 | Ca-Kabuli-Chr2 | 7701230 | [T/G] | Ca18123 | Synonymous-CDS | Multiantimicrobial extrusion protein |
| CWSNP1277 | Ca-Kabuli-Chr2 | 7702468 | [A/T] | Ca18123 | Intron | Multiantimicrobial extrusion protein |
| CWSNP1278 | Ca-Kabuli-Chr2 | 7708663 | [T/C] | - | Intergenic | - |
| CWSNP1279 | Ca-Kabuli-Chr2 | 7708660 | [G/A] | - | Intergenic | - |
| CWSNP1280 | Ca-Kabuli-Chr2 | 7708630 | [C/A] | - | Intergenic | - |

| SNP IDs | Chromosomes | Physical positions (bp) | SNPs | Gene accession IDs | Sequence components of genes | Putative functions |
|---|---|---|---|---|---|---|
| CWSNP1281 | Ca-Kabuli-Chr2 | 7708596 | [T/C] | - | Intergenic | - |
| CWSNP1282 | Ca-Kabuli-Chr2 | 7708678 | [C/T] | - | Intergenic | - |
| CWSNP1283 | Ca-Kabuli-Chr2 | 7712181 | [C/T] | - | Intergenic | - |
| CWSNP1284 | Ca-Kabuli-Chr2 | 7714392 | [G/A] | Ca18124 | Non-Synonymous-CDS | - |
| CWSNP1285 | Ca-Kabuli-Chr2 | 7714454 | [A/G] | Ca18124 | Synonymous-CDS | - |
| CWSNP1286 | Ca-Kabuli-Chr2 | 7714463 | [T/C] | Ca18124 | Synonymous-CDS | - |
| CWSNP1287 | Ca-Kabuli-Chr2 | 7753419 | [G/C] | - | DRR | - |
| CWSNP1288 | Ca-Kabuli-Chr2 | 7753425 | [T/C] | - | DRR | - |
| CWSNP1289 | Ca-Kabuli-Chr2 | 7753440 | [C/T] | - | DRR | - |
| CWSNP1290 | Ca-Kabuli-Chr2 | 7819281 | [T/C] | - | DRR | - |
| CWSNP1291 | Ca-Kabuli-Chr2 | 7870919 | [G/T] | - | DRR | - |
| CWSNP1292 | Ca-Kabuli-Chr2 | 7870943 | [T/G] | - | DRR | - |
| CWSNP1293 | Ca-Kabuli-Chr2 | 7938890 | [T/C] | Ca18144 | Synonymous-CDS | Allinase,C-terminal |
| CWSNP1294 | Ca-Kabuli-Chr2 | 8337517 | [C/T] | Ca21468 | Intron | Phosphatidyl inositol 3-/4-kinase, catalytic |
| CWSNP1295 | Ca-Kabuli-Chr2 | 8484840 | [A/T] | - | URR | - |
| CWSNP1296 | Ca-Kabuli-Chr2 | 8487331 | [T/A] | - | Intergenic | - |

| SNP IDs | Chromosomes | Physical positions (bp) | SNPs | Gene accession IDs | Sequence components of genes | Putative functions |
|---|---|---|---|---|---|---|
| CWSNP1297 | Ca-Kabuli-Chr2 | 8487343 | [C/T] | - | Intergenic | - |
| CWSNP1298 | Ca-Kabuli-Chr2 | 8489241 | [C/A] | - | URR | - |
| CWSNP1299 | Ca-Kabuli-Chr2 | 8489237 | [G/A] | - | URR | - |
| CWSNP1300 | Ca-Kabuli-Chr2 | 8489172 | [C/A] | - | URR | - |
| CWSNP1301 | Ca-Kabuli-Chr2 | 9621953 | [A/C] | Ca25089 | Synonymous-CDS | Actin-binding FH2/DRF auto regulatory |
| CWSNP1302 | Ca-Kabuli-Chr2 | 9740010 | [A/G] | - | Intergenic | - |
| CWSNP1303 | Ca-Kabuli-Chr2 | 9740007 | [A/G] | - | Intergenic | - |
| CWSNP1304 | Ca-Kabuli-Chr2 | 9739980 | [G/C] | - | Intergenic | - |
| CWSNP1305 | Ca-Kabuli-Chr2 | 9739974 | [T/C] | - | Intergenic | - |
| CWSNP1306 | Ca-Kabuli-Chr2 | 9739959 | [T/C] | - | Intergenic | - |
| CWSNP1307 | Ca-Kabuli-Chr2 | 9739965 | [A/G] | - | Intergenic | - |
| CWSNP1308 | Ca-Kabuli-Chr2 | 9756955 | [G/A] | - | Intergenic | - |
| CWSNP1309 | Ca-Kabuli-Chr2 | 9756910 | [G/A] | - | Intergenic | - |
| CWSNP1310 | Ca-Kabuli-Chr2 | 9862794 | [A/G] | Ca20401 | Intron | Protein kinase, catalytic domain |
| CWSNP1311 | Ca-Kabuli-Chr2 | 10060196 | [G/T] | - | Intergenic | - |
| CWSNP1312 | Ca-Kabuli-Chr2 | 10060200 | [G/T] | - | Intergenic | - |

| SNP IDs | Chromosomes | Physical positions (bp) | SNPs | Gene accession IDs | Sequence components of genes | Putative functions |
|---|---|---|---|---|---|---|
| CWSNP1313 | Ca-Kabuli-Chr2 | 10060207 | [C/T] | - | Intergenic | - |
| CWSNP1314 | Ca-Kabuli-Chr2 | 10067195 | [T/A] | Ca20380 | Intron | SANT domain, DNA binding |
| CWSNP1315 | Ca-Kabuli-Chr2 | 10067451 | [A/T] | Ca20380 | Intron | SANT domain, DNA binding |
| CWSNP1316 | Ca-Kabuli-Chr2 | 10220396 | [T/C] | - | DRR | - |
| CWSNP1317 | Ca-Kabuli-Chr2 | 10294635 | [T/C] | Ca17594 | Intron | ATPase,P-type,K/Mg/Cd/Cu/Zn/Na/Ca/Na/H-transporter |
| CWSNP1318 | Ca-Kabuli-Chr2 | 10540735 | [C/T] | Ca17577 | Non-Synonymous-CDS | - |
| CWSNP1319 | Ca-Kabuli-Chr2 | 10705982 | [C/T] | - | Intergenic | - |
| CWSNP1320 | Ca-Kabuli-Chr2 | 10760939 | [G/A] | - | URR | - |
| CWSNP1321 | Ca-Kabuli-Chr2 | 10761036 | [C/G] | - | URR | - |
| CWSNP1322 | Ca-Kabuli-Chr2 | 11799472 | [G/A] | - | Intergenic | - |
| CWSNP1323 | Ca-Kabuli-Chr2 | 12252370 | [A/G] | - | Intergenic | - |
| CWSNP1324 | Ca-Kabuli-Chr2 | 12389166 | [G/T] | - | Intergenic | - |
| CWSNP1325 | Ca-Kabuli-Chr2 | 12389112 | [G/A] | - | Intergenic | - |
| CWSNP1326 | Ca-Kabuli-Chr2 | 12528825 | [C/G] | Ca18079 | Non-Synonymous-CDS | Zinc finger,Dof-type |
| CWSNP1327 | Ca-Kabuli-Chr2 | 12794438 | [G/A] | Ca18093 | Non-Synonymous-CDS | EF-HAND2 |
| CWSNP1328 | Ca-Kabuli-Chr2 | 13079445 | [T/G] | Ca11686 | Synonymous-CDS | Protein of unknown function DUF3110 |

| SNP IDs | Chromosomes | Physical positions (bp) | SNPs | Gene accession IDs | Sequence components of genes | Putative functions |
|---|---|---|---|---|---|---|
| CWSNP1329 | Ca-Kabuli-Chr2 | 13355018 | [T/G] | - | URR | - |
| CWSNP1330 | Ca-Kabuli-Chr2 | 13355022 | [G/T] | - | URR | - |
| CWSNP1331 | Ca-Kabuli-Chr2 | 13355033 | [A/T] | - | URR | - |
| CWSNP1332 | Ca-Kabuli-Chr2 | 13355615 | [T/G] | - | URR | - |
| CWSNP1333 | Ca-Kabuli-Chr2 | 13434995 | [A/C] | Ca11665 | Intron | Glutamyl-tRNA(Gln)amidotransferase,subunitB/E,N-terminal |
| CWSNP1334 | Ca-Kabuli-Chr2 | 13437317 | [A/G] | Ca11665 | Intron | Glutamyl-tRNA(Gln)amidotransferase,subunitB/E,N-terminal |
| CWSNP1335 | Ca-Kabuli-Chr2 | 13529057 | [G/A] | - | URR | - |
| CWSNP1336 | Ca-Kabuli-Chr2 | 13782906 | [C/A] | Ca11639 | Intron | Remorin,C-terminal |
| CWSNP1337 | Ca-Kabuli-Chr2 | 14136013 | [T/G] | - | Intergenic | - |
| CWSNP1338 | Ca-Kabuli-Chr2 | 14136223 | [A/C] | - | Intergenic | - |
| CWSNP1339 | Ca-Kabuli-Chr2 | 15234951 | [C/A] | Ca18564 | Synonymous-CDS | Protein of unknown function DUF1675 |
| CWSNP1340 | Ca-Kabuli-Chr2 | 15390673 | [A/G] | Ca18558 | Intron | Protein kinase, catalytic domain |
| CWSNP1341 | Ca-Kabuli-Chr2 | 16460174 | [G/A] | - | Intergenic | - |
| CWSNP1342 | Ca-Kabuli-Chr2 | 16460142 | [A/G] | - | Intergenic | - |
| CWSNP1343 | Ca-Kabuli-Chr2 | 16460097 | [C/A] | - | Intergenic | - |
| CWSNP1344 | Ca-Kabuli-Chr2 | 16460175 | [C/A] | - | Intergenic | - |

| SNP IDs | Chromosomes | Physical positions (bp) | SNPs | Gene accession IDs | Sequence components of genes | Putative functions |
|---|---|---|---|---|---|---|
| CWSNP1345 | Ca-Kabuli-Chr2 | 16462095 | [A/G] | - | Intergenic | - |
| CWSNP1346 | Ca-Kabuli-Chr2 | 16462089 | [C/T] | - | Intergenic | - |
| CWSNP1347 | Ca-Kabuli-Chr2 | 16555733 | [C/T] | Ca22041 | Intron | BSD |
| CWSNP1348 | Ca-Kabuli-Chr2 | 16555790 | [C/G] | Ca22041 | Intron | BSD |
| CWSNP1349 | Ca-Kabuli-Chr2 | 16563117 | [A/T] | Ca22041 | Intron | BSD |
| CWSNP1350 | Ca-Kabuli-Chr2 | 16563121 | [A/T] | Ca22041 | Intron | BSD |
| CWSNP1351 | Ca-Kabuli-Chr2 | 16563123 | [C/T] | Ca22041 | Intron | BSD |
| CWSNP1352 | Ca-Kabuli-Chr2 | 16563125 | [A/T] | Ca22041 | Intron | BSD |
| CWSNP1353 | Ca-Kabuli-Chr2 | 16563133 | [A/T] | Ca22041 | Intron | BSD |
| CWSNP1354 | Ca-Kabuli-Chr2 | 16705119 | [C/T] | - | Intergenic | - |
| CWSNP1355 | Ca-Kabuli-Chr2 | 17578623 | [G/A] | - | Intergenic | - |
| CWSNP1356 | Ca-Kabuli-Chr2 | 17578633 | [C/G] | - | Intergenic | - |
| CWSNP1357 | Ca-Kabuli-Chr2 | 17578689 | [G/A] | - | Intergenic | - |
| CWSNP1358 | Ca-Kabuli-Chr2 | 17578676 | [T/C] | - | Intergenic | - |
| CWSNP1359 | Ca-Kabuli-Chr2 | 17578701 | [A/C] | - | Intergenic | - |
| CWSNP1360 | Ca-Kabuli-Chr2 | 17578706 | [A/T] | - | Intergenic | - |

| SNP IDs | Chromosomes | Physical positions (bp) | SNPs | Gene accession IDs | Sequence components of genes | Putative functions |
|---|---|---|---|---|---|---|
| CWSNP1361 | Ca-Kabuli-Chr2 | 17578740 | [A/G] | - | Intergenic | - |
| CWSNP1362 | Ca-Kabuli-Chr2 | 17681915 | [C/T] | - | Intergenic | - |
| CWSNP1363 | Ca-Kabuli-Chr2 | 18312200 | [T/C] | - | Intergenic | - |
| CWSNP1364 | Ca-Kabuli-Chr2 | 18395410 | [T/C] | - | Intergenic | - |
| CWSNP1365 | Ca-Kabuli-Chr2 | 18631522 | [A/C] | - | Intergenic | - |
| CWSNP1366 | Ca-Kabuli-Chr2 | 18671692 | [C/A] | - | Intergenic | - |
| CWSNP1367 | Ca-Kabuli-Chr2 | 18671672 | [T/A] | - | Intergenic | - |
| CWSNP1368 | Ca-Kabuli-Chr2 | 18671666 | [C/T] | - | Intergenic | - |
| CWSNP1369 | Ca-Kabuli-Chr2 | 18671637 | [G/A] | - | Intergenic | - |
| CWSNP1370 | Ca-Kabuli-Chr2 | 18671618 | [T/G] | - | Intergenic | - |
| CWSNP1371 | Ca-Kabuli-Chr2 | 18671723 | [C/G] | - | Intergenic | - |
| CWSNP1372 | Ca-Kabuli-Chr2 | 18671746 | [C/T] | - | Intergenic | - |
| CWSNP1373 | Ca-Kabuli-Chr2 | 18671756 | [T/A] | - | Intergenic | - |
| CWSNP1374 | Ca-Kabuli-Chr2 | 18671765 | [C/A] | - | Intergenic | - |
| CWSNP1375 | Ca-Kabuli-Chr2 | 20392224 | [A/G] | Ca25924 | Synonymous-CDS | - |
| CWSNP1376 | Ca-Kabuli-Chr2 | 20762205 | [A/T] | Ca23762 | Synonymous-CDS | - |

| SNP IDs | Chromosomes | Physical positions (bp) | SNPs | Gene accession IDs | Sequence components of genes | Putative functions |
|---|---|---|---|---|---|---|
| CWSNP1377 | Ca-Kabuli-Chr2 | 21000509 | [C/T] | - | Intergenic | - |
| CWSNP1378 | Ca-Kabuli-Chr2 | 21000466 | [C/T] | - | Intergenic | - |
| CWSNP1379 | Ca-Kabuli-Chr2 | 21000463 | [C/T] | - | Intergenic | - |
| CWSNP1380 | Ca-Kabuli-Chr2 | 21849874 | [A/T] | Ca23059 | Intron | - |
| CWSNP1381 | Ca-Kabuli-Chr2 | 21965365 | [T/C] | - | Intergenic | - |
| CWSNP1382 | Ca-Kabuli-Chr2 | 22158602 | [C/T] | Ca15007 | Non-Synonymous-CDS | PeptidaseC48,SUMO/Sentrin/Ubl1 |
| CWSNP1383 | Ca-Kabuli-Chr2 | 22190743 | [C/T] | - | Intergenic | - |
| CWSNP1384 | Ca-Kabuli-Chr2 | 22190748 | [G/T] | - | Intergenic | - |
| CWSNP1385 | Ca-Kabuli-Chr2 | 22190751 | [C/T] | - | Intergenic | - |
| CWSNP1386 | Ca-Kabuli-Chr2 | 22190753 | [C/T] | - | Intergenic | - |
| CWSNP1387 | Ca-Kabuli-Chr2 | 22190815 | [C/T] | - | Intergenic | - |
| CWSNP1388 | Ca-Kabuli-Chr2 | 22347405 | [C/T] | Ca15004 | Intron | Cyclin,C-terminal |
| CWSNP1389 | Ca-Kabuli-Chr2 | 22457234 | [G/A] | - | URR | - |
| CWSNP1390 | Ca-Kabuli-Chr2 | 22457215 | [G/A] | - | URR | - |
| CWSNP1391 | Ca-Kabuli-Chr2 | 22457233 | [C/T] | - | URR | - |
| CWSNP1392 | Ca-Kabuli-Chr2 | 22457263 | [G/A] | - | URR | - |

| SNP IDs | Chromosomes | Physical positions (bp) | SNPs | Gene accession IDs | Sequence components of genes | Putative functions |
|---|---|---|---|---|---|---|
| CWSNP1393 | Ca-Kabuli-Chr2 | 22457257 | [G/A] | - | URR | - |
| CWSNP1394 | Ca-Kabuli-Chr2 | 22483072 | [C/T] | - | Intergenic | - |
| CWSNP1395 | Ca-Kabuli-Chr2 | 22483067 | [G/A] | - | Intergenic | - |
| CWSNP1396 | Ca-Kabuli-Chr2 | 22483052 | [C/T] | - | Intergenic | - |
| CWSNP1397 | Ca-Kabuli-Chr2 | 22483019 | [A/T] | - | Intergenic | - |
| CWSNP1398 | Ca-Kabuli-Chr2 | 22703091 | [A/C] | Ca14990 | Intron | RhoGTPase-activating protein domain |
| CWSNP1399 | Ca-Kabuli-Chr2 | 22820616 | [C/G] | Ca14985 | Intron | Peptidase C19,ubiquitin carboxyl-terminalhydrolase 2 |
| CWSNP1400 | Ca-Kabuli-Chr2 | 22820588 | [A/C] | Ca14985 | Intron | Peptidase C19,ubiquitin carboxyl-terminalhydrolase 2 |
| CWSNP1401 | Ca-Kabuli-Chr2 | 22897694 | [G/A] | - | Intergenic | - |
| CWSNP1402 | Ca-Kabuli-Chr2 | 22897647 | [T/C] | - | Intergenic | - |
| CWSNP1403 | Ca-Kabuli-Chr2 | 23247801 | [C/T] | - | Intergenic | - |
| CWSNP1404 | Ca-Kabuli-Chr2 | 23282374 | [T/C] | - | Intergenic | - |
| CWSNP1405 | Ca-Kabuli-Chr2 | 23329424 | [C/T] | - | Intergenic | - |
| CWSNP1406 | Ca-Kabuli-Chr2 | 23329440 | [G/A] | - | Intergenic | - |
| CWSNP1407 | Ca-Kabuli-Chr2 | 23439966 | [T/C] | Ca14960 | Non-Synonymous-CDS | Ubiquitin-associated/translation elongation factor EF1B, N-terminal |
| CWSNP1408 | Ca-Kabuli-Chr2 | 23910254 | [T/C] | - | DRR | - |

| SNP IDs | Chromosomes | Physical positions (bp) | SNPs | Gene accession IDs | Sequence components of genes | Putative functions |
|---|---|---|---|---|---|---|
| CWSNP1409 | Ca-Kabuli-Chr2 | 24048087 | [A/C] | Ca21673 | Intron | PUCCprotein |
| CWSNP1410 | Ca-Kabuli-Chr2 | 24511053 | [G/A] | - | Intergenic | - |
| CWSNP1411 | Ca-Kabuli-Chr2 | 24511114 | [G/A] | - | Intergenic | - |
| CWSNP1412 | Ca-Kabuli-Chr2 | 24511121 | [G/A] | - | Intergenic | - |
| CWSNP1413 | Ca-Kabuli-Chr2 | 24511119 | [T/G] | - | Intergenic | - |
| CWSNP1414 | Ca-Kabuli-Chr2 | 24511116 | [C/A] | - | Intergenic | - |
| CWSNP1415 | Ca-Kabuli-Chr2 | 24709295 | [G/A] | Ca22892 | Intron | DNA mismatch repair protein MutS, C-terminaldomain |
| CWSNP1416 | Ca-Kabuli-Chr2 | 24741195 | [C/T] | - | URR | - |
| CWSNP1417 | Ca-Kabuli-Chr2 | 24817957 | [G/A] | - | Intergenic | - |
| CWSNP1418 | Ca-Kabuli-Chr2 | 24817945 | [C/T] | - | Intergenic | - |
| CWSNP1419 | Ca-Kabuli-Chr2 | 24817940 | [G/A] | - | Intergenic | - |
| CWSNP1420 | Ca-Kabuli-Chr2 | 24817934 | [A/G] | - | Intergenic | - |
| CWSNP1421 | Ca-Kabuli-Chr2 | 24817921 | [G/A] | - | Intergenic | - |
| CWSNP1422 | Ca-Kabuli-Chr2 | 25022971 | [A/G] | Ca14236 | Non-Synonymous-CDS | Zinc finger,B-box |
| CWSNP1423 | Ca-Kabuli-Chr2 | 25163062 | [C/G] | Ca14248 | Intron | Glycosidehydrolase,family3,N-terminal |
| CWSNP1424 | Ca-Kabuli-Chr2 | 25317391 | [A/G] | Ca14265 | Synonymous-CDS | ABC transporter-like |

| SNP IDs | Chromosomes | Physical positions (bp) | SNPs | Gene accession IDs | Sequence components of genes | Putative functions |
|---|---|---|---|---|---|---|
| CWSNP1425 | Ca-Kabuli-Chr2 | 25493973 | [T/G] | Ca14279 | Synonymous-CDS | Chaperonin ClpA/B |
| CWSNP1426 | Ca-Kabuli-Chr2 | 25508679 | [A/G] | Ca14280 | Non-Synonymous-CDS | Zinc finger,CCCH-type |
| CWSNP1427 | Ca-Kabuli-Chr2 | 25930490 | [G/A] | Ca14304 | Non-Synonymous-CDS | Phospho glycerate/bisphosphoglycerate mutase, active site |
| CWSNP1428 | Ca-Kabuli-Chr2 | 26051667 | [T/G] | - | Intergenic | - |
| CWSNP1429 | Ca-Kabuli-Chr2 | 26092881 | [C/G] | - | Intergenic | - |
| CWSNP1430 | Ca-Kabuli-Chr2 | 26092913 | [C/A] | - | Intergenic | - |
| CWSNP1431 | Ca-Kabuli-Chr2 | 26092928 | [T/G] | - | Intergenic | - |
| CWSNP1432 | Ca-Kabuli-Chr2 | 26121495 | [A/C] | Ca14316 | Synonymous-CDS | - |
| CWSNP1433 | Ca-Kabuli-Chr2 | 26121491 | [T/G] | Ca14316 | Synonymous-CDS | - |
| CWSNP1434 | Ca-Kabuli-Chr2 | 26461867 | [C/G] | Ca17247 | Non-Synonymous-CDS | Metallo-dependent phosphatase |
| CWSNP1435 | Ca-Kabuli-Chr2 | 26502153 | [T/C] | - | Intergenic | - |
| CWSNP1436 | Ca-Kabuli-Chr2 | 26706849 | [A/G] | - | DRR | - |
| CWSNP1437 | Ca-Kabuli-Chr2 | 27057582 | [C/T] | Ca15636 | Intron | - |
| CWSNP1438 | Ca-Kabuli-Chr2 | 27063749 | [C/A] | Ca15636 | Non-Synonymous-CDS | - |
| CWSNP1439 | Ca-Kabuli-Chr2 | 27134574 | [A/G] | - | Intergenic | - |
| CWSNP1440 | Ca-Kabuli-Chr2 | 27134560 | [G/T] | - | Intergenic | - |

| SNP IDs | Chromosomes | Physical positions (bp) | SNPs | Gene accession IDs | Sequence components of genes | Putative functions |
|---|---|---|---|---|---|---|
| CWSNP1441 | Ca-Kabuli-Chr2 | 27182855 | [T/C] | - | Intergenic | - |
| CWSNP1442 | Ca-Kabuli-Chr2 | 27724425 | [G/T] | Ca15674 | Synonymous-CDS | BTB/POZ-like |
| CWSNP1443 | Ca-Kabuli-Chr2 | 27766561 | [T/A] | Ca15677 | Non-Synonymous-CDS | Cation efflux protein |
| CWSNP1444 | Ca-Kabuli-Chr2 | 27766605 | [A/C] | Ca15677 | Synonymous-CDS | Cation efflux protein |
| CWSNP1445 | Ca-Kabuli-Chr2 | 27766594 | [C/A] | Ca15677 | Non-Synonymous-CDS | Cation efflux protein |
| CWSNP1446 | Ca-Kabuli-Chr2 | 27835871 | [G/A] | - | DRR | - |
| CWSNP1447 | Ca-Kabuli-Chr2 | 27835850 | [G/A] | - | DRR | - |
| CWSNP1448 | Ca-Kabuli-Chr2 | 27835843 | [C/A] | - | DRR | - |
| CWSNP1449 | Ca-Kabuli-Chr2 | 27835837 | [C/T] | - | DRR | - |
| CWSNP1450 | Ca-Kabuli-Chr2 | 27835832 | [C/T] | - | DRR | - |
| CWSNP1451 | Ca-Kabuli-Chr2 | 28018158 | [T/C] | - | DRR | - |
| CWSNP1452 | Ca-Kabuli-Chr2 | 28061049 | [G/T] | Ca14397 | Non-Synonymous-CDS | Uncharacterised protein familyUPF0497,trans-membraneplant |
| CWSNP1453 | Ca-Kabuli-Chr2 | 28084953 | [C/T] | - | DRR | - |
| CWSNP1454 | Ca-Kabuli-Chr2 | 28407316 | [T/C] | - | Intergenic | - |
| CWSNP1455 | Ca-Kabuli-Chr2 | 28407324 | [C/T] | - | Intergenic | - |
| CWSNP1456 | Ca-Kabuli-Chr2 | 28534235 | [T/C] | - | Intergenic | - |

| SNP IDs | Chromosomes | Physical positions (bp) | SNPs | Gene accession IDs | Sequence components of genes | Putative functions |
|---|---|---|---|---|---|---|
| CWSNP1457 | Ca-Kabuli-Chr2 | 28741764 | [T/C] | - | Intergenic | - |
| CWSNP1458 | Ca-Kabuli-Chr2 | 28944712 | [T/C] | - | DRR | - |
| CWSNP1459 | Ca-Kabuli-Chr2 | 28944707 | [A/C] | - | DRR | - |
| CWSNP1460 | Ca-Kabuli-Chr2 | 28949911 | [A/C] | Ca14342 | Non-Synonymous-CDS | Glycosidehydrolase,family17 |
| CWSNP1461 | Ca-Kabuli-Chr2 | 28949919 | [A/T] | Ca14342 | Non-Synonymous-CDS | Glycosidehydrolase,family17 |
| CWSNP1462 | Ca-Kabuli-Chr2 | 29122589 | [T/G] | Ca14327 | Non-Synonymous-CDS | PeptidaseA1 |
| CWSNP1463 | Ca-Kabuli-Chr2 | 30335160 | [T/C] | - | Intergenic | - |
| CWSNP1464 | Ca-Kabuli-Chr2 | 30335212 | [T/A] | - | Intergenic | - |
| CWSNP1465 | Ca-Kabuli-Chr2 | 30335194 | [A/G] | - | Intergenic | - |
| CWSNP1466 | Ca-Kabuli-Chr2 | 30363957 | [C/A] | - | Intergenic | - |
| CWSNP1467 | Ca-Kabuli-Chr2 | 30364073 | [T/A] | - | Intergenic | - |
| CWSNP1468 | Ca-Kabuli-Chr2 | 30364042 | [A/G] | - | Intergenic | - |
| CWSNP1469 | Ca-Kabuli-Chr2 | 30364025 | [T/C] | - | Intergenic | - |
| CWSNP1470 | Ca-Kabuli-Chr2 | 30370411 | [T/C] | - | DRR | - |
| CWSNP1471 | Ca-Kabuli-Chr2 | 30467551 | [A/T] | Ca12503 | Intron | Mov34/MPN/PAD-1 |
| CWSNP1472 | Ca-Kabuli-Chr2 | 30508454 | [T/C] | Ca12501 | Intron | EXTL2, alpha-1,4-N-acetyl hexosaminyl transferase |

| SNP IDs | Chromosomes | Physical positions (bp) | SNPs | Gene accession IDs | Sequence components of genes | Putative functions |
|---|---|---|---|---|---|---|
| CWSNP1473 | Ca-Kabuli-Chr2 | 30530642 | [A/T] | Ca12498 | Synonymous-CDS | Ribosomal protein L10/acidic P0 |
| CWSNP1474 | Ca-Kabuli-Chr2 | 30537667 | [A/T] | - | URR | - |
| CWSNP1475 | Ca-Kabuli-Chr2 | 30537673 | [A/G] | - | URR | - |
| CWSNP1476 | Ca-Kabuli-Chr2 | 30537687 | [T/C] | - | URR | - |
| CWSNP1477 | Ca-Kabuli-Chr2 | 30537701 | [T/A] | - | URR | - |
| CWSNP1478 | Ca-Kabuli-Chr2 | 30537953 | [T/C] | - | URR | - |
| CWSNP1479 | Ca-Kabuli-Chr2 | 30686920 | [C/T] | Ca12488 | Intron | HAD-superfamily hydrolase,subfamily IG, 5'-nucleotidase |
| CWSNP1480 | Ca-Kabuli-Chr2 | 30867547 | [G/A] | - | DRR | - |
| CWSNP1481 | Ca-Kabuli-Chr2 | 30880148 | [A/G] | Ca12473 | Intron | Synaptobrevin |
| CWSNP1482 | Ca-Kabuli-Chr2 | 30880243 | [G/C] | Ca12473 | Intron | Synaptobrevin |
| CWSNP1483 | Ca-Kabuli-Chr2 | 30880246 | [G/A] | Ca12473 | Intron | Synaptobrevin |
| CWSNP1484 | Ca-Kabuli-Chr2 | 30880303 | [T/A] | Ca12473 | Intron | Synaptobrevin |
| CWSNP1485 | Ca-Kabuli-Chr2 | 30880302 | [T/C] | Ca12473 | Intron | Synaptobrevin |
| CWSNP1486 | Ca-Kabuli-Chr2 | 30880282 | [A/G] | Ca12473 | Intron | Synaptobrevin |
| CWSNP1487 | Ca-Kabuli-Chr2 | 30921092 | [C/A] | Ca12470 | Synonymous-CDS | - |
| CWSNP1488 | Ca-Kabuli-Chr2 | 30921758 | [C/T] | Ca12470 | Intron | - |

| SNP IDs | Chromosomes | Physical positions (bp) | SNPs | Gene accession IDs | Sequence components of genes | Putative functions |
|---|---|---|---|---|---|---|
| CWSNP1489 | Ca-Kabuli-Chr2 | 30921917 | [C/A] | Ca12470 | Intron | - |
| CWSNP1490 | Ca-Kabuli-Chr2 | 30921923 | [A/G] | Ca12470 | Intron | - |
| CWSNP1491 | Ca-Kabuli-Chr2 | 30954317 | [G/A] | Ca12469 | Intron | Clathrin,heavy chain/VPS,7-fold repeat |
| CWSNP1492 | Ca-Kabuli-Chr2 | 30954305 | [T/G] | Ca12469 | Intron | Clathrin,heavy chain/VPS,7-fold repeat |
| CWSNP1493 | Ca-Kabuli-Chr2 | 30977572 | [A/G] | - | Intergenic | - |
| CWSNP1494 | Ca-Kabuli-Chr2 | 30977528 | [C/T] | - | Intergenic | - |
| CWSNP1495 | Ca-Kabuli-Chr2 | 30977522 | [A/T] | - | Intergenic | - |
| CWSNP1496 | Ca-Kabuli-Chr2 | 30977508 | [A/T] | - | Intergenic | - |
| CWSNP1497 | Ca-Kabuli-Chr2 | 30997769 | [T/A] | Ca12465 | Synonymous-CDS | Pathogenesis-related transcriptional factor/ERF, DNA-binding |
| CWSNP1498 | Ca-Kabuli-Chr2 | 30997780 | [A/C] | Ca12465 | Synonymous-CDS | Pathogenesis-related transcriptional factor/ERF, DNA-binding |
| CWSNP1499 | Ca-Kabuli-Chr2 | 30997784 | [T/A] | Ca12465 | Synonymous-CDS | Pathogenesis-related transcriptional factor/ERF, DNA-binding |
| CWSNP1500 | Ca-Kabuli-Chr2 | 31005847 | [T/G] | Ca12464 | Synonymous-CDS | Lateral organ boundaries, LOB |
| CWSNP1501 | Ca-Kabuli-Chr2 | 31005845 | [C/T] | Ca12464 | Synonymous-CDS | Lateral organ boundaries, LOB |
| CWSNP1502 | Ca-Kabuli-Chr2 | 31055007 | [G/A] | Ca12459 | Intron | Rad21/Rec8-like protein,C-terminal,eukaryotic |
| CWSNP1503 | Ca-Kabuli-Chr2 | 31088681 | [G/T] | - | DRR | - |
| CWSNP1504 | Ca-Kabuli-Chr2 | 31088676 | [G/C] | - | DRR | - |

| SNP IDs | Chromosomes | Physical positions (bp) | SNPs | Gene accession IDs | Sequence components of genes | Putative functions |
|---|---|---|---|---|---|---|
| CWSNP1505 | Ca-Kabuli-Chr2 | 31088670 | [C/T] | - | DRR | - |
| CWSNP1506 | Ca-Kabuli-Chr2 | 31088648 | [A/G] | - | DRR | - |
| CWSNP1507 | Ca-Kabuli-Chr2 | 31088645 | [A/C] | - | DRR | - |
| CWSNP1508 | Ca-Kabuli-Chr2 | 31088778 | [A/T] | - | DRR | - |
| CWSNP1509 | Ca-Kabuli-Chr2 | 31088800 | [T/C] | - | DRR | - |
| CWSNP1510 | Ca-Kabuli-Chr2 | 31088882 | [A/G] | - | DRR | - |
| CWSNP1511 | Ca-Kabuli-Chr2 | 31109311 | [G/T] | - | URR | - |
| CWSNP1512 | Ca-Kabuli-Chr2 | 31109358 | [C/T] | - | URR | - |
| CWSNP1513 | Ca-Kabuli-Chr2 | 31109481 | [G/A] | - | URR | - |
| CWSNP1514 | Ca-Kabuli-Chr2 | 31109441 | [C/G] | - | URR | - |
| CWSNP1515 | Ca-Kabuli-Chr2 | 31109435 | [G/A] | - | URR | - |
| CWSNP1516 | Ca-Kabuli-Chr2 | 31109427 | [C/T] | - | URR | - |
| CWSNP1517 | Ca-Kabuli-Chr2 | 31141019 | [G/A] | - | DRR | - |
| CWSNP1518 | Ca-Kabuli-Chr2 | 31188355 | [A/G] | Ca12445 | Synonymous-CDS | Multi copper oxidase, type1 |
| CWSNP1519 | Ca-Kabuli-Chr2 | 31201872 | [T/C] | Ca12444 | Intron | Phox homologous domain |
| CWSNP1520 | Ca-Kabuli-Chr2 | 31201859 | [T/A] | Ca12444 | Intron | Phox homologous domain |

| SNP IDs | Chromosomes | Physical positions (bp) | SNPs | Gene accession IDs | Sequence components of genes | Putative functions |
|---|---|---|---|---|---|---|
| CWSNP1521 | Ca-Kabuli-Chr2 | 31203154 | [G/A] | Ca12444 | Synonymous-CDS | Phox homologous domain |
| CWSNP1522 | Ca-Kabuli-Chr2 | 31427861 | [A/G] | Ca12419 | Non-Synonymous-CDS | Protein of unknown function DUF827,plant |
| CWSNP1523 | Ca-Kabuli-Chr2 | 31427902 | [G/C] | Ca12419 | Non-Synonymous-CDS | Protein of unknown function DUF827,plant |
| CWSNP1524 | Ca-Kabuli-Chr2 | 31428024 | [T/G] | Ca12419 | Non-Synonymous-CDS | Protein of unknown function DUF827,plant |
| CWSNP1525 | Ca-Kabuli-Chr2 | 31428132 | [G/A] | Ca12419 | Synonymous-CDS | Protein of unknown function DUF827,plant |
| CWSNP1526 | Ca-Kabuli-Chr2 | 31428405 | [T/C] | Ca12419 | Synonymous-CDS | Protein of unknown function DUF827,plant |
| CWSNP1527 | Ca-Kabuli-Chr2 | 31428429 | [T/G] | Ca12419 | Non-Synonymous-CDS | Protein of unknown function DUF827,plant |
| CWSNP1528 | Ca-Kabuli-Chr2 | 31534265 | [A/C] | - | Intergenic | - |
| CWSNP1529 | Ca-Kabuli-Chr2 | 31534283 | [A/G] | - | Intergenic | - |
| CWSNP1530 | Ca-Kabuli-Chr2 | 31534455 | [T/C] | - | Intergenic | - |
| CWSNP1531 | Ca-Kabuli-Chr2 | 31686714 | [C/T] | - | Intergenic | - |
| CWSNP1532 | Ca-Kabuli-Chr2 | 31756777 | [G/T] | - | Intergenic | - |
| CWSNP1533 | Ca-Kabuli-Chr2 | 31847033 | [A/C] | - | Intergenic | - |
| CWSNP1534 | Ca-Kabuli-Chr2 | 31881918 | [G/T] | Ca17830 | Non-Synonymous-CDS | RZZcomplex,subunitZw10 |
| CWSNP1535 | Ca-Kabuli-Chr2 | 31885934 | [A/G] | - | DRR | - |
| CWSNP1536 | Ca-Kabuli-Chr2 | 31908694 | [G/A] | Ca17826 | Synonymous-CDS | Zinc finger,RING-type |

| SNP IDs | Chromosomes | Physical positions (bp) | SNPs | Gene accession IDs | Sequence components of genes | Putative functions |
|---|---|---|---|---|---|---|
| CWSNP1537 | Ca-Kabuli-Chr2 | 31908742 | [G/A] | Ca17826 | Synonymous-CDS | Zinc finger,RING-type |
| CWSNP1538 | Ca-Kabuli-Chr2 | 31908803 | [C/T] | Ca17826 | Intron | Zinc finger,RING-type |
| CWSNP1539 | Ca-Kabuli-Chr2 | 31908787 | [G/A] | Ca17826 | Intron | Zinc finger,RING-type |
| CWSNP1540 | Ca-Kabuli-Chr2 | 31908845 | [T/A] | Ca17826 | Intron | Zinc finger,RING-type |
| CWSNP1541 | Ca-Kabuli-Chr2 | 31908857 | [C/T] | Ca17826 | Intron | Zinc finger,RING-type |
| CWSNP1542 | Ca-Kabuli-Chr2 | 31941463 | [T/C] | Ca17823 | Synonymous-CDS | Zinc finger,CCCH-type |
| CWSNP1543 | Ca-Kabuli-Chr2 | 31941554 | [A/T] | Ca17823 | Intron | Zinc finger,CCCH-type |
| CWSNP1544 | Ca-Kabuli-Chr2 | 32003536 | [G/T] | - | Intergenic | - |
| CWSNP1545 | Ca-Kabuli-Chr2 | 32017361 | [G/A] | - | Intergenic | - |
| CWSNP1546 | Ca-Kabuli-Chr2 | 32049523 | [C/A] | Ca17817 | Non-Synonymous-CDS | - |
| CWSNP1547 | Ca-Kabuli-Chr2 | 32049525 | [A/C] | Ca17817 | Synonymous-CDS | - |
| CWSNP1548 | Ca-Kabuli-Chr2 | 32058591 | [T/C] | Ca17816 | Synonymous-CDS | Ankyrin repeat |
| CWSNP1549 | Ca-Kabuli-Chr2 | 32061053 | [C/T] | Ca17816 | Intron | Ankyrin repeat |
| CWSNP1550 | Ca-Kabuli-Chr2 | 32061054 | [T/C] | Ca17816 | Intron | Ankyrin repeat |
| CWSNP1551 | Ca-Kabuli-Chr2 | 32062010 | [A/C] | Ca17816 | Synonymous-CDS | Ankyrin repeat |
| CWSNP1552 | Ca-Kabuli-Chr2 | 32071107 | [C/G] | - | DRR | - |

| SNP IDs | Chromosomes | Physical positions (bp) | SNPs | Gene accession IDs | Sequence components of genes | Putative functions |
|---|---|---|---|---|---|---|
| CWSNP1553 | Ca-Kabuli-Chr2 | 32080002 | [T/C] | - | Intergenic | - |
| CWSNP1554 | Ca-Kabuli-Chr2 | 32080007 | [T/C] | - | Intergenic | - |
| CWSNP1555 | Ca-Kabuli-Chr2 | 32080010 | [G/C] | - | Intergenic | - |
| CWSNP1556 | Ca-Kabuli-Chr2 | 32080016 | [A/T] | - | Intergenic | - |
| CWSNP1557 | Ca-Kabuli-Chr2 | 32080022 | [C/A] | - | Intergenic | - |
| CWSNP1558 | Ca-Kabuli-Chr2 | 32080037 | [A/G] | - | Intergenic | - |
| CWSNP1559 | Ca-Kabuli-Chr2 | 32096192 | [A/C] | - | Intergenic | - |
| CWSNP1560 | Ca-Kabuli-Chr2 | 32096186 | [T/A] | - | Intergenic | - |
| CWSNP1561 | Ca-Kabuli-Chr2 | 32096180 | [C/G] | - | Intergenic | - |
| CWSNP1562 | Ca-Kabuli-Chr2 | 32096177 | [C/T] | - | Intergenic | - |
| CWSNP1563 | Ca-Kabuli-Chr2 | 32096172 | [C/T] | - | Intergenic | - |
| CWSNP1564 | Ca-Kabuli-Chr2 | 32099907 | [T/C] | - | Intergenic | - |
| CWSNP1565 | Ca-Kabuli-Chr2 | 32109805 | [G/A] | - | Intergenic | - |
| CWSNP1566 | Ca-Kabuli-Chr2 | 32109811 | [G/A] | - | Intergenic | - |
| CWSNP1567 | Ca-Kabuli-Chr2 | 32109813 | [C/T] | - | Intergenic | - |
| CWSNP1568 | Ca-Kabuli-Chr2 | 32109821 | [G/A] | - | Intergenic | - |

| SNP IDs | Chromosomes | Physical positions (bp) | SNPs | Gene accession IDs | Sequence components of genes | Putative functions |
|---|---|---|---|---|---|---|
| CWSNP1569 | Ca-Kabuli-Chr2 | 32109827 | [T/C] | - | Intergenic | - |
| CWSNP1570 | Ca-Kabuli-Chr2 | 32109859 | [A/C] | - | Intergenic | - |
| CWSNP1571 | Ca-Kabuli-Chr2 | 32109904 | [T/A] | - | Intergenic | - |
| CWSNP1572 | Ca-Kabuli-Chr2 | 32109895 | [A/G] | - | Intergenic | - |
| CWSNP1573 | Ca-Kabuli-Chr2 | 32109892 | [T/A] | - | Intergenic | - |
| CWSNP1574 | Ca-Kabuli-Chr2 | 32119094 | [A/T] | - | Intergenic | - |
| CWSNP1575 | Ca-Kabuli-Chr2 | 32119268 | [T/A] | - | Intergenic | - |
| CWSNP1576 | Ca-Kabuli-Chr2 | 32119274 | [T/C] | - | Intergenic | - |
| CWSNP1577 | Ca-Kabuli-Chr2 | 32119331 | [A/T] | - | Intergenic | - |
| CWSNP1578 | Ca-Kabuli-Chr2 | 32129272 | [T/C] | - | Intergenic | - |
| CWSNP1579 | Ca-Kabuli-Chr2 | 32129395 | [C/G] | - | Intergenic | - |
| CWSNP1580 | Ca-Kabuli-Chr2 | 32140939 | [A/T] | - | DRR | - |
| CWSNP1581 | Ca-Kabuli-Chr2 | 32140948 | [A/C] | - | DRR | - |
| CWSNP1582 | Ca-Kabuli-Chr2 | 32140987 | [A/G] | - | DRR | - |
| CWSNP1583 | Ca-Kabuli-Chr2 | 32141047 | [A/G] | - | DRR | - |
| CWSNP1584 | Ca-Kabuli-Chr2 | 32141038 | [T/C] | - | DRR | - |

| SNP IDs | Chromosomes | Physical positions (bp) | SNPs | Gene accession IDs | Sequence components of genes | Putative functions |
|---|---|---|---|---|---|---|
| CWSNP1585 | Ca-Kabuli-Chr2 | 32141019 | [G/A] | - | DRR | - |
| CWSNP1586 | Ca-Kabuli-Chr2 | 32141013 | [G/C] | - | DRR | - |
| CWSNP1587 | Ca-Kabuli-Chr2 | 32141002 | [A/T] | - | DRR | - |
| CWSNP1588 | Ca-Kabuli-Chr2 | 32140998 | [G/T] | - | DRR | - |
| CWSNP1589 | Ca-Kabuli-Chr2 | 32140990 | [G/A] | - | DRR | - |
| CWSNP1590 | Ca-Kabuli-Chr2 | 32203032 | [A/G] | Ca10295 | Synonymous-CDS | PeptidaseS8/S53,subtilisin/kexin/sedolisin |
| CWSNP1591 | Ca-Kabuli-Chr2 | 32203036 | [A/T] | Ca10295 | Non-Synonymous-CDS | PeptidaseS8/S53,subtilisin/kexin/sedolisin |
| CWSNP1592 | Ca-Kabuli-Chr2 | 32242818 | [T/G] | - | DRR | - |
| CWSNP1593 | Ca-Kabuli-Chr2 | 32348054 | [T/A] | Ca10283 | Intron | - |
| CWSNP1594 | Ca-Kabuli-Chr2 | 32348087 | [A/C] | Ca10283 | Intron | - |
| CWSNP1595 | Ca-Kabuli-Chr2 | 32348119 | [G/T] | Ca10283 | Intron | - |
| CWSNP1596 | Ca-Kabuli-Chr2 | 32348793 | [G/A] | Ca10283 | Intron | - |
| CWSNP1597 | Ca-Kabuli-Chr2 | 32348870 | [T/C] | Ca10283 | Intron | - |
| CWSNP1598 | Ca-Kabuli-Chr2 | 32411994 | [G/T] | - | URR | - |
| CWSNP1599 | Ca-Kabuli-Chr2 | 32412000 | [G/T] | - | URR | - |
| CWSNP1600 | Ca-Kabuli-Chr2 | 32481969 | [C/T] | - | Intergenic | - |

| SNP IDs | Chromosomes | Physical positions (bp) | SNPs | Gene accession IDs | Sequence components of genes | Putative functions |
|---|---|---|---|---|---|---|
| CWSNP1601 | Ca-Kabuli-Chr2 | 32481983 | [G/T] | - | Intergenic | - |
| CWSNP1602 | Ca-Kabuli-Chr2 | 32545320 | [A/G] | Ca10268 | Synonymous-CDS | Zinc finger,CCHC-type |
| CWSNP1603 | Ca-Kabuli-Chr2 | 32564427 | [T/C] | Ca10266 | Intron | RecF/RecN/SMC |
| CWSNP1604 | Ca-Kabuli-Chr2 | 32564480 | [G/A] | Ca10266 | Intron | RecF/RecN/SMC |
| CWSNP1605 | Ca-Kabuli-Chr2 | 32564770 | [G/T] | Ca10266 | Intron | RecF/RecN/SMC |
| CWSNP1606 | Ca-Kabuli-Chr2 | 32564796 | [A/T] | Ca10266 | Intron | RecF/RecN/SMC |
| CWSNP1607 | Ca-Kabuli-Chr2 | 32570394 | [G/A] | Ca10266 | Intron | RecF/RecN/SMC |
| CWSNP1608 | Ca-Kabuli-Chr2 | 32570429 | [G/A] | Ca10266 | Intron | RecF/RecN/SMC |
| CWSNP1609 | Ca-Kabuli-Chr2 | 32570504 | [G/C] | Ca10266 | Intron | RecF/RecN/SMC |
| CWSNP1610 | Ca-Kabuli-Chr2 | 32579849 | [T/C] | Ca10266 | Synonymous-CDS | RecF/RecN/SMC |
| CWSNP1611 | Ca-Kabuli-Chr2 | 32579900 | [C/G] | Ca10266 | Synonymous-CDS | RecF/RecN/SMC |
| CWSNP1612 | Ca-Kabuli-Chr2 | 32611624 | [C/T] | Ca10261 | Synonymous-CDS | Aldehyde oxidase/xanthine dehydrogenase,a/bhammer head |
| CWSNP1613 | Ca-Kabuli-Chr2 | 32611601 | [G/T] | Ca10261 | Non-Synonymous-CDS | Aldehyde oxidase/xanthine dehydrogenase,a/bhammer head |
| CWSNP1614 | Ca-Kabuli-Chr2 | 32670181 | [T/G] | Ca10254 | Non-Synonymous-CDS | BTB/POZ-like |
| CWSNP1615 | Ca-Kabuli-Chr2 | 32844754 | [T/G] | Ca10238 | Non-Synonymous-CDS | Late embryogenesis abundant protein, group2 |
| CWSNP1616 | Ca-Kabuli-Chr2 | 32936908 | [G/A] | Ca10230 | Non-Synonymous-CDS | Protein of unknown functionDM15 |

| SNP IDs | Chromosomes | Physical positions (bp) | SNPs | Gene accession IDs | Sequence components of genes | Putative functions |
|---|---|---|---|---|---|---|
| CWSNP1617 | Ca-Kabuli-Chr2 | 32958034 | [C/G] | - | Intergenic | - |
| CWSNP1618 | Ca-Kabuli-Chr2 | 32989205 | [G/T] | - | DRR | - |
| CWSNP1619 | Ca-Kabuli-Chr2 | 33103766 | [G/A] | - | URR | - |
| CWSNP1620 | Ca-Kabuli-Chr2 | 33184262 | [T/C] | Ca10203 | Non-Synonymous-CDS | - |
| CWSNP1621 | Ca-Kabuli-Chr2 | 33326510 | [C/G] | - | Intergenic | - |
| CWSNP1622 | Ca-Kabuli-Chr2 | 33398561 | [C/T] | - | Intergenic | - |
| CWSNP1623 | Ca-Kabuli-Chr2 | 33398550 | [G/A] | - | Intergenic | - |
| CWSNP1624 | Ca-Kabuli-Chr2 | 33398549 | [T/C] | - | Intergenic | - |
| CWSNP1625 | Ca-Kabuli-Chr2 | 33398527 | [T/G] | - | Intergenic | - |
| CWSNP1626 | Ca-Kabuli-Chr2 | 33399045 | [A/C] | Ca10186 | Synonymous-CDS | Disease resistance protein |
| CWSNP1627 | Ca-Kabuli-Chr2 | 33399098 | [G/A] | Ca10186 | Non-Synonymous-CDS | Disease resistance protein |
| CWSNP1628 | Ca-Kabuli-Chr2 | 33399063 | [C/T] | Ca10186 | Synonymous-CDS | Disease resistance protein |
| CWSNP1629 | Ca-Kabuli-Chr2 | 33399405 | [C/T] | Ca10186 | Synonymous-CDS | Disease resistance protein |
| CWSNP1630 | Ca-Kabuli-Chr2 | 33399404 | [C/A] | Ca10186 | Non-Synonymous-CDS | Disease resistance protein |
| CWSNP1631 | Ca-Kabuli-Chr2 | 33399397 | [C/T] | Ca10186 | Non-Synonymous-CDS | Disease resistance protein |
| CWSNP1632 | Ca-Kabuli-Chr2 | 33399393 | [C/A] | Ca10186 | Non-Synonymous-CDS | Disease resistance protein |

| SNP IDs | Chromosomes | Physical positions (bp) | SNPs | Gene accession IDs | Sequence components of genes | Putative functions |
|---|---|---|---|---|---|---|
| CWSNP1633 | Ca-Kabuli-Chr2 | 33399381 | [C/T] | Ca10186 | Synonymous-CDS | Disease resistance protein |
| CWSNP1634 | Ca-Kabuli-Chr2 | 33399342 | [A/C] | Ca10186 | Non-Synonymous-CDS | Disease resistance protein |
| CWSNP1635 | Ca-Kabuli-Chr2 | 33401608 | [A/G] | Ca10186 | Synonymous-CDS | Disease resistance protein |
| CWSNP1636 | Ca-Kabuli-Chr2 | 33490058 | [C/A] | Ca10183 | Non-Synonymous-CDS | Telomerase activating protein Est1 |
| CWSNP1637 | Ca-Kabuli-Chr2 | 33575286 | [A/C] | - | Intergenic | - |
| CWSNP1638 | Ca-Kabuli-Chr2 | 33582948 | [G/A] | Ca10177 | Non-Synonymous-CDS | PeptidaseC48,SUMO/Sentrin/Ubl1 |
| CWSNP1639 | Ca-Kabuli-Chr2 | 33582900 | [G/A] | Ca10177 | Non-Synonymous-CDS | PeptidaseC48,SUMO/Sentrin/Ubl1 |
| CWSNP1640 | Ca-Kabuli-Chr2 | 33650447 | [A/T] | - | Intergenic | - |
| CWSNP1641 | Ca-Kabuli-Chr2 | 33651426 | [G/A] | Ca10173 | Synonymous-CDS | Pentatrico peptide repeat |
| CWSNP1642 | Ca-Kabuli-Chr2 | 33651408 | [A/G] | - | Intergenic | - |
| CWSNP1643 | Ca-Kabuli-Chr2 | 33661177 | [C/T] | Ca10172 | Synonymous-CDS | - |
| CWSNP1644 | Ca-Kabuli-Chr2 | 33713018 | [G/A] | - | Intergenic | - |
| CWSNP1645 | Ca-Kabuli-Chr2 | 33786243 | [G/A] | - | Intergenic | - |
| CWSNP1646 | Ca-Kabuli-Chr2 | 33788913 | [A/C] | - | DRR | - |
| CWSNP1647 | Ca-Kabuli-Chr2 | 34152352 | [T/C] | Ca15238 | Intron | - |
| CWSNP1648 | Ca-Kabuli-Chr2 | 34155090 | [A/G] | Ca15238 | Intron | - |

| SNP IDs | Chromosomes | Physical positions (bp) | SNPs | Gene accession IDs | Sequence components of genes | Putative functions |
|---|---|---|---|---|---|---|
| CWSNP1649 | Ca-Kabuli-Chr2 | 34288116 | [G/C] | - | Intergenic | - |
| CWSNP1650 | Ca-Kabuli-Chr2 | 34299074 | [A/C] | - | Intergenic | - |
| CWSNP1651 | Ca-Kabuli-Chr2 | 34299079 | [A/C] | - | Intergenic | - |
| CWSNP1652 | Ca-Kabuli-Chr2 | 34343478 | [C/G] | - | DRR | - |
| CWSNP1653 | Ca-Kabuli-Chr2 | 34343705 | [G/A] | - | DRR | - |
| CWSNP1654 | Ca-Kabuli-Chr2 | 34346849 | [C/T] | - | DRR | - |
| CWSNP1655 | Ca-Kabuli-Chr2 | 34346842 | [T/C] | - | DRR | - |
| CWSNP1656 | Ca-Kabuli-Chr2 | 34372503 | [T/G] | - | DRR | - |
| CWSNP1657 | Ca-Kabuli-Chr2 | 34428978 | [C/A] | - | Intergenic | - |
| CWSNP1658 | Ca-Kabuli-Chr2 | 34428979 | [G/C] | - | Intergenic | - |
| CWSNP1659 | Ca-Kabuli-Chr2 | 34551849 | [A/C] | - | Intergenic | - |
| CWSNP1660 | Ca-Kabuli-Chr2 | 34554164 | [A/C] | - | Intergenic | - |
| CWSNP1661 | Ca-Kabuli-Chr2 | 34601444 | [C/A] | Ca15284 | Synonymous-CDS | Lipocalin |
| CWSNP1662 | Ca-Kabuli-Chr2 | 34638955 | [T/A] | - | URR | - |
| CWSNP1663 | Ca-Kabuli-Chr2 | 34734379 | [G/A] | Ca16897 | Synonymous-CDS | Heat shock protein Hsp70 |
| CWSNP1664 | Ca-Kabuli-Chr2 | 34820075 | [C/T] | - | URR | - |

| SNP IDs | Chromosomes | Physical positions (bp) | SNPs | Gene accession IDs | Sequence components of genes | Putative functions |
|---|---|---|---|---|---|---|
| CWSNP1665 | Ca-Kabuli-Chr2 | 34830995 | [A/G] | - | Intergenic | - |
| CWSNP1666 | Ca-Kabuli-Chr2 | 34847681 | [C/A] | Ca16883 | Synonymous-CDS | Bromodomain |
| CWSNP1667 | Ca-Kabuli-Chr2 | 34848053 | [G/A] | Ca16883 | Synonymous-CDS | Bromodomain |
| CWSNP1668 | Ca-Kabuli-Chr2 | 34848480 | [T/C] | Ca16883 | Non-Synonymous-CDS | Bromodomain |
| CWSNP1669 | Ca-Kabuli-Chr2 | 34862276 | [C/G] | Ca16882 | Non-Synonymous-CDS | Lipase,GDSL |
| CWSNP1670 | Ca-Kabuli-Chr2 | 34888414 | [T/C] | - | Intergenic | - |
| CWSNP1671 | Ca-Kabuli-Chr2 | 35013088 | [G/C] | - | Intergenic | - |
| CWSNP1672 | Ca-Kabuli-Chr2 | 35013084 | [T/C] | - | Intergenic | - |
| CWSNP1673 | Ca-Kabuli-Chr2 | 35052930 | [C/A] | Ca16868 | Intron | Aspartate carbamoyltransferase,eukaryotic |
| CWSNP1674 | Ca-Kabuli-Chr2 | 35054244 | [C/A] | Ca16868 | Intron | Aspartate carbamoyltransferase,eukaryotic |
| CWSNP1675 | Ca-Kabuli-Chr2 | 35059446 | [A/T] | - | DRR | - |
| CWSNP1676 | Ca-Kabuli-Chr2 | 35059434 | [T/C] | - | DRR | - |
| CWSNP1677 | Ca-Kabuli-Chr2 | 35060171 | [A/G] | - | DRR | - |
| CWSNP1678 | Ca-Kabuli-Chr2 | 35060178 | [A/T] | - | DRR | - |
| CWSNP1679 | Ca-Kabuli-Chr2 | 35060228 | [T/C] | - | DRR | - |
| CWSNP1680 | Ca-Kabuli-Chr2 | 35060273 | [G/A] | - | DRR | - |

| SNP IDs | Chromosomes | Physical positions (bp) | SNPs | Gene accession IDs | Sequence components of genes | Putative functions |
|---|---|---|---|---|---|---|
| CWSNP1681 | Ca-Kabuli-Chr2 | 35060264 | [A/T] | - | DRR | - |
| CWSNP1682 | Ca-Kabuli-Chr2 | 35066138 | [G/A] | - | URR | - |
| CWSNP1683 | Ca-Kabuli-Chr2 | 35120761 | [G/A] | Ca16857 | Synonymous-CDS | Metallo-dependent phosphatase |
| CWSNP1684 | Ca-Kabuli-Chr2 | 35122109 | [C/G] | Ca16857 | Intron | Metallo-dependent phosphatase |
| CWSNP1685 | Ca-Kabuli-Chr2 | 35154629 | [C/A] | - | Intergenic | - |
| CWSNP1686 | Ca-Kabuli-Chr2 | 35154603 | [G/A] | - | Intergenic | - |
| CWSNP1687 | Ca-Kabuli-Chr2 | 35171882 | [T/G] | Ca16849 | Synonymous-CDS | Glycerophosphoryldiester phosphodiesterase |
| CWSNP1688 | Ca-Kabuli-Chr2 | 35189471 | [C/T] | - | DRR | - |
| CWSNP1689 | Ca-Kabuli-Chr2 | 35209940 | [C/T] | Ca16844 | Non-Synonymous-CDS | C2 calcium-dependent membrane targeting |
| CWSNP1690 | Ca-Kabuli-Chr2 | 35362587 | [A/G] | - | Intergenic | - |
| CWSNP1691 | Ca-Kabuli-Chr2 | 35362642 | [A/G] | - | Intergenic | - |
| CWSNP1692 | Ca-Kabuli-Chr2 | 35362729 | [A/G] | Ca09693 | Synonymous-CDS | Frigida-like |
| CWSNP1693 | Ca-Kabuli-Chr2 | 35363501 | [T/C] | Ca09693 | Non-Synonymous-CDS | Frigida-like |
| CWSNP1694 | Ca-Kabuli-Chr2 | 35365029 | [C/T] | Ca09693 | Non-Synonymous-CDS | Frigida-like |
| CWSNP1695 | Ca-Kabuli-Chr2 | 35413400 | [T/A] | - | DRR | - |
| CWSNP1696 | Ca-Kabuli-Chr2 | 35441180 | [A/G] | - | Intergenic | - |

| SNP IDs | Chromosomes | Physical positions (bp) | SNPs | Gene accession IDs | Sequence components of genes | Putative functions |
|---|---|---|---|---|---|---|
| CWSNP1697 | Ca-Kabuli-Chr2 | 35441174 | [T/G] | - | Intergenic | - |
| CWSNP1698 | Ca-Kabuli-Chr2 | 35447976 | [A/G] | - | DRR | - |
| CWSNP1699 | Ca-Kabuli-Chr2 | 35449653 | [G/A] | Ca09705 | Intron | ABC transporter, transmembrane domain |
| CWSNP1700 | Ca-Kabuli-Chr2 | 35473250 | [C/T] | - | Intergenic | - |
| CWSNP1701 | Ca-Kabuli-Chr2 | 35505791 | [C/G] | - | DRR | - |
| CWSNP1702 | Ca-Kabuli-Chr2 | 35505874 | [T/G] | - | DRR | - |
| CWSNP1703 | Ca-Kabuli-Chr2 | 35593797 | [G/T] | Ca09728 | Synonymous-CDS | Bacterial Fmu(Sun)/eukaryotic nucleolar NOL1/Nop2p |
| CWSNP1704 | Ca-Kabuli-Chr2 | 35593824 | [A/C] | Ca09728 | Synonymous-CDS | Bacterial Fmu(Sun)/eukaryotic nucleolar NOL1/Nop2p |
| CWSNP1705 | Ca-Kabuli-Chr2 | 35593827 | [A/G] | Ca09728 | Synonymous-CDS | Bacterial Fmu(Sun)/eukaryotic nucleolar NOL1/Nop2p |
| CWSNP1706 | Ca-Kabuli-Chr2 | 35594018 | [A/T] | Ca09728 | Intron | Bacterial Fmu(Sun)/eukaryotic nucleolar NOL1/Nop2p |
| CWSNP1707 | Ca-Kabuli-Chr2 | 35594013 | [C/T] | Ca09728 | Intron | Bacterial Fmu(Sun)/eukaryotic nucleolar NOL1/Nop2p |
| CWSNP1708 | Ca-Kabuli-Chr2 | 35593985 | [A/G] | Ca09728 | Intron | Bacterial Fmu(Sun)/eukaryotic nucleolar NOL1/Nop2p |
| CWSNP1709 | Ca-Kabuli-Chr2 | 35593965 | [C/T] | Ca09728 | Intron | Bacterial Fmu(Sun)/eukaryotic nucleolar NOL1/Nop2p |
| CWSNP1710 | Ca-Kabuli-Chr2 | 35593956 | [A/G] | Ca09728 | Intron | Bacterial Fmu(Sun)/eukaryotic nucleolar NOL1/Nop2p |
| CWSNP1711 | Ca-Kabuli-Chr2 | 35601566 | [T/C] | - | Intergenic | - |
| CWSNP1712 | Ca-Kabuli-Chr2 | 35601571 | [A/C] | - | Intergenic | - |

| SNP IDs | Chromosomes | Physical positions (bp) | SNPs | Gene accession IDs | Sequence components of genes | Putative functions |
|---|---|---|---|---|---|---|
| CWSNP1713 | Ca-Kabuli-Chr2 | 35601578 | [C/T] | - | Intergenic | - |
| CWSNP1714 | Ca-Kabuli-Chr2 | 35601691 | [A/G] | - | Intergenic | - |
| CWSNP1715 | Ca-Kabuli-Chr2 | 35601657 | [T/C] | - | Intergenic | - |
| CWSNP1716 | Ca-Kabuli-Chr2 | 35601632 | [G/T] | - | Intergenic | - |
| CWSNP1717 | Ca-Kabuli-Chr2 | 35612587 | [C/A] | Ca09731 | Synonymous-CDS | Protein of unknown function DUF827,plant |
| CWSNP1718 | Ca-Kabuli-Chr2 | 35612644 | [G/A] | Ca09731 | Synonymous-CDS | Protein of unknown function DUF827,plant |
| CWSNP1719 | Ca-Kabuli-Chr2 | 35612895 | [T/C] | Ca09731 | Non-Synonymous-CDS | Protein of unknown function DUF827,plant |
| CWSNP1720 | Ca-Kabuli-Chr2 | 35612900 | [C/T] | Ca09731 | Non-Synonymous-CDS | Protein of unknown function DUF827,plant |
| CWSNP1721 | Ca-Kabuli-Chr2 | 35613135 | [T/C] | - | Intergenic | - |
| CWSNP1722 | Ca-Kabuli-Chr2 | 35658537 | [T/C] | Ca09735 | Synonymous-CDS | Oligopeptide transporterOPTsuperfamily |
| CWSNP1723 | Ca-Kabuli-Chr2 | 35661053 | [C/T] | Ca09735 | Non-Synonymous-CDS | Oligopeptide transporterOPTsuperfamily |
| CWSNP1724 | Ca-Kabuli-Chr2 | 35670478 | [A/G] | Ca09737 | Intron | Protein of unknown function DUF1296 |
| CWSNP1725 | Ca-Kabuli-Chr2 | 35704018 | [A/G] | - | URR | - |
| CWSNP1726 | Ca-Kabuli-Chr2 | 35704069 | [G/A] | - | URR | - |
| CWSNP1727 | Ca-Kabuli-Chr2 | 35704228 | [C/A] | - | URR | - |
| CWSNP1728 | Ca-Kabuli-Chr2 | 35704213 | [A/G] | - | URR | - |

| SNP IDs | Chromosomes | Physical positions (bp) | SNPs | Gene accession IDs | Sequence components of genes | Putative functions |
|---|---|---|---|---|---|---|
| CWSNP1729 | Ca-Kabuli-Chr2 | 35704204 | [T/C] | - | URR | - |
| CWSNP1730 | Ca-Kabuli-Chr2 | 35704200 | [T/C] | - | URR | - |
| CWSNP1731 | Ca-Kabuli-Chr2 | 35704180 | [A/G] | - | URR | - |
| CWSNP1732 | Ca-Kabuli-Chr2 | 35704178 | [G/A] | - | URR | - |
| CWSNP1733 | Ca-Kabuli-Chr2 | 35728285 | [T/A] | - | Intergenic | - |
| CWSNP1734 | Ca-Kabuli-Chr2 | 35754617 | [G/T] | - | Intergenic | - |
| CWSNP1735 | Ca-Kabuli-Chr2 | 35754557 | [C/T] | - | Intergenic | - |
| CWSNP1736 | Ca-Kabuli-Chr2 | 35754680 | [C/A] | - | Intergenic | - |
| CWSNP1737 | Ca-Kabuli-Chr2 | 35754682 | [A/G] | - | Intergenic | - |
| CWSNP1738 | Ca-Kabuli-Chr2 | 35754806 | [G/C] | - | Intergenic | - |
| CWSNP1739 | Ca-Kabuli-Chr2 | 35754757 | [G/C] | - | Intergenic | - |
| CWSNP1740 | Ca-Kabuli-Chr2 | 35759932 | [A/T] | Ca09749 | Intron | Regulatory protein RecX |
| CWSNP1741 | Ca-Kabuli-Chr2 | 35759940 | [T/C] | Ca09749 | Intron | Regulatory protein RecX |
| CWSNP1742 | Ca-Kabuli-Chr2 | 35759981 | [G/A] | Ca09749 | Intron | Regulatory protein RecX |
| CWSNP1743 | Ca-Kabuli-Chr2 | 35760062 | [G/C] | Ca09749 | Intron | Regulatory protein RecX |
| CWSNP1744 | Ca-Kabuli-Chr2 | 35760073 | [A/T] | Ca09749 | Intron | Regulatory protein RecX |

| SNP IDs | Chromosomes | Physical positions (bp) | SNPs | Gene accession IDs | Sequence components of genes | Putative functions |
|---|---|---|---|---|---|---|
| CWSNP1745 | Ca-Kabuli-Chr2 | 35760170 | [A/G] | Ca09749 | Intron | Regulatory protein RecX |
| CWSNP1746 | Ca-Kabuli-Chr2 | 35760155 | [G/A] | Ca09749 | Intron | Regulatory protein RecX |
| CWSNP1747 | Ca-Kabuli-Chr2 | 35795395 | [A/G] | Ca09754 | Synonymous-CDS | PeptidaseS8/S53,subtilisin/kexin/sedolisin |
| CWSNP1748 | Ca-Kabuli-Chr2 | 35795494 | [G/A] | Ca09754 | Synonymous-CDS | PeptidaseS8/S53,subtilisin/kexin/sedolisin |
| CWSNP1749 | Ca-Kabuli-Chr2 | 35795496 | [G/T] | Ca09754 | Non-Synonymous-CDS | PeptidaseS8/S53,subtilisin/kexin/sedolisin |
| CWSNP1750 | Ca-Kabuli-Chr2 | 35795521 | [T/C] | Ca09754 | Synonymous-CDS | PeptidaseS8/S53,subtilisin/kexin/sedolisin |
| CWSNP1751 | Ca-Kabuli-Chr2 | 35862878 | [T/G] | Ca09762 | Intron | Armadillo |
| CWSNP1752 | Ca-Kabuli-Chr2 | 35930699 | [T/G] | - | Intergenic | - |
| CWSNP1753 | Ca-Kabuli-Chr2 | 35993178 | [G/A] | Ca09773 | Synonymous-CDS | Disease resistance protein |
| CWSNP1754 | Ca-Kabuli-Chr2 | 36013809 | [A/G] | Ca09776 | Intron | Nucleoporin,Nup133/Nup155-like,C-terminal |
| CWSNP1755 | Ca-Kabuli-Chr2 | 36039428 | [C/T] | - | Intergenic | - |
| CWSNP1756 | Ca-Kabuli-Chr2 | 36039364 | [G/T] | - | Intergenic | - |
| CWSNP1757 | Ca-Kabuli-Chr2 | 36046593 | [G/T] | - | Intergenic | - |
| CWSNP1758 | Ca-Kabuli-Chr2 | 36046595 | [T/G] | - | Intergenic | - |
| CWSNP1759 | Ca-Kabuli-Chr2 | 36046741 | [G/A] | - | Intergenic | - |
| CWSNP1760 | Ca-Kabuli-Chr2 | 36046720 | [T/A] | - | Intergenic | - |

| SNP IDs | Chromosomes | Physical positions (bp) | SNPs | Gene accession IDs | Sequence components of genes | Putative functions |
|---|---|---|---|---|---|---|
| CWSNP1761 | Ca-Kabuli-Chr2 | 36046767 | [T/A] | - | Intergenic | - |
| CWSNP1762 | Ca-Kabuli-Chr2 | 36046820 | [T/A] | - | Intergenic | - |
| CWSNP1763 | Ca-Kabuli-Chr2 | 36069340 | [T/C] | Ca09784 | Synonymous-CDS | Signal transduction response regulator,receiver domain |
| CWSNP1764 | Ca-Kabuli-Chr2 | 36072247 | [C/A] | - | Intergenic | - |
| CWSNP1765 | Ca-Kabuli-Chr2 | 36074147 | [T/C] | - | DRR | - |
| CWSNP1766 | Ca-Kabuli-Chr2 | 36088577 | [A/T] | - | Intergenic | - |
| CWSNP1767 | Ca-Kabuli-Chr2 | 36088676 | [T/C] | - | Intergenic | - |
| CWSNP1768 | Ca-Kabuli-Chr2 | 36088853 | [G/A] | - | Intergenic | - |
| CWSNP1769 | Ca-Kabuli-Chr2 | 36101358 | [A/G] | - | URR | - |
| CWSNP1770 | Ca-Kabuli-Chr2 | 36101379 | [A/G] | - | URR | - |
| CWSNP1771 | Ca-Kabuli-Chr2 | 36117695 | [T/A] | - | Intergenic | - |
| CWSNP1772 | Ca-Kabuli-Chr2 | 36117647 | [G/A] | - | Intergenic | - |
| CWSNP1773 | Ca-Kabuli-Chr2 | 36276791 | [G/A] | - | DRR | - |
| CWSNP1774 | Ca-Kabuli-Chr2 | 36326361 | [A/C] | - | URR | - |
| CWSNP1775 | Ca-Kabuli-Chr2 | 36335252 | [A/C] | - | URR | - |
| CWSNP1776 | Ca-Kabuli-Chr2 | 36346336 | [A/G] | - | Intergenic | - |

| SNP IDs | Chromosomes | Physical positions (bp) | SNPs | Gene accession IDs | Sequence components of genes | Putative functions |
|---|---|---|---|---|---|---|
| CWSNP1777 | Ca-Kabuli-Chr2 | 36387245 | [T/C] | Ca09828 | Non-Synonymous-CDS | GDP-fucose proteinO-fucosyltransferase |
| CWSNP1778 | Ca-Kabuli-Chr2 | 36429512 | [T/C] | Ca09832 | Non-Synonymous-CDS | Phox/Bem1p |
| CWSNP1779 | Ca-Kabuli-Chr2 | 36467100 | [A/C] | - | URR | - |
| CWSNP1780 | Ca-Kabuli-Chr2 | 36469921 | [A/G] | Ca09835 | Intron | EPS15 homology (EH) |
| CWSNP1781 | Ca-Kabuli-Chr2 | 36505316 | [C/A] | - | DRR | - |
| CWSNP1782 | Ca-Kabuli-Chr2 | 36609730 | [T/A] | - | Intergenic | - |
| CWSNP1783 | Ca-Kabuli-Chr2 | 36609743 | [C/T] | - | Intergenic | - |
| CWSNP1784 | Ca-Kabuli-Chr2 | 36609744 | [G/C] | - | Intergenic | - |
| CWSNP1785 | Ca-Kabuli-Chr2 | 36609950 | [C/T] | - | Intergenic | - |
| CWSNP1786 | Ca-Kabuli-Chr2 | 36609968 | [C/T] | - | Intergenic | - |
| CWSNP1787 | Ca-Kabuli-Chr2 | 36634234 | [T/C] | - | Intergenic | - |
| CWSNP1788 | Ca-Kabuli-Chr2 | 36634297 | [C/G] | - | Intergenic | - |
| CWSNP1789 | Ca-Kabuli-Chr3 | 171675 | [T/A] | Ca22938 | Synonymous-CDS | NAD-dependent epimerase/dehydratase |
| CWSNP1790 | Ca-Kabuli-Chr3 | 220568 | [A/T] | Ca22935 | Intron | Glycosyltransferase,family48 |
| CWSNP1791 | Ca-Kabuli-Chr3 | 220572 | [T/C] | Ca22935 | Intron | Glycosyltransferase,family48 |
| CWSNP1792 | Ca-Kabuli-Chr3 | 220573 | [G/T] | Ca22935 | Intron | Glycosyltransferase,family48 |

| SNP IDs | Chromosomes | Physical positions (bp) | SNPs | Gene accession IDs | Sequence components of genes | Putative functions |
|---|---|---|---|---|---|---|
| CWSNP1793 | Ca-Kabuli-Chr3 | 419715 | [T/G] | - | DRR | - |
| CWSNP1794 | Ca-Kabuli-Chr3 | 988477 | [G/A] | - | Intergenic | - |
| CWSNP1795 | Ca-Kabuli-Chr3 | 1499038 | [G/A] | - | Intergenic | - |
| CWSNP1796 | Ca-Kabuli-Chr3 | 1499006 | [A/G] | - | Intergenic | - |
| CWSNP1797 | Ca-Kabuli-Chr3 | 1746388 | [C/A] | Ca19405 | Intron | Helicase,C-terminal |
| CWSNP1798 | Ca-Kabuli-Chr3 | 1746389 | [A/G] | Ca19405 | Intron | Helicase,C-terminal |
| CWSNP1799 | Ca-Kabuli-Chr3 | 2097376 | [T/G] | - | Intergenic | - |
| CWSNP1800 | Ca-Kabuli-Chr3 | 2097413 | [A/G] | - | Intergenic | - |
| CWSNP1801 | Ca-Kabuli-Chr3 | 2391515 | [T/G] | Ca26372 | Synonymous-CDS | NHLrepeat |
| CWSNP1802 | Ca-Kabuli-Chr3 | 2708703 | [C/T] | Ca22148 | Non-Synonymous-CDS | Zinc finger,B-box |
| CWSNP1803 | Ca-Kabuli-Chr3 | 2825546 | [G/T] | - | Intergenic | - |
| CWSNP1804 | Ca-Kabuli-Chr3 | 2927148 | [T/A] | - | DRR | - |
| CWSNP1805 | Ca-Kabuli-Chr3 | 2927150 | [A/T] | - | DRR | - |
| CWSNP1806 | Ca-Kabuli-Chr3 | 2975179 | [T/C] | Ca22161 | Synonymous-CDS | - |
| CWSNP1807 | Ca-Kabuli-Chr3 | 3280478 | [C/T] | Ca24555 | Non-Synonymous-CDS | RNA recognition motif domain |
| CWSNP1808 | Ca-Kabuli-Chr3 | 3617555 | [G/A] | Ca21346 | Non-Synonymous-CDS | ZF-HD homeobox protein,Cys/His-rich dimerisation domain |

| SNP IDs | Chromosomes | Physical positions (bp) | SNPs | Gene accession IDs | Sequence components of genes | Putative functions |
|---|---|---|---|---|---|---|
| CWSNP1809 | Ca-Kabuli-Chr3 | 3701394 | [A/T] | Ca21344 | Intron | Gibberellin regulated protein |
| CWSNP1810 | Ca-Kabuli-Chr3 | 3701396 | [C/T] | Ca21344 | Intron | Gibberellin regulated protein |
| CWSNP1811 | Ca-Kabuli-Chr3 | 3701398 | [A/T] | Ca21344 | Intron | Gibberellin regulated protein |
| CWSNP1812 | Ca-Kabuli-Chr3 | 3701401 | [A/T] | Ca21344 | Intron | Gibberellin regulated protein |
| CWSNP1813 | Ca-Kabuli-Chr3 | 3701405 | [G/T] | Ca21344 | Intron | Gibberellin regulated protein |
| CWSNP1814 | Ca-Kabuli-Chr3 | 3701415 | [C/T] | Ca21344 | Intron | Gibberellin regulated protein |
| CWSNP1815 | Ca-Kabuli-Chr3 | 3701422 | [C/T] | Ca21344 | Intron | Gibberellin regulated protein |
| CWSNP1816 | Ca-Kabuli-Chr3 | 3701426 | [T/C] | Ca21344 | Intron | Gibberellin regulated protein |
| CWSNP1817 | Ca-Kabuli-Chr3 | 3701435 | [A/T] | Ca21344 | Intron | Gibberellin regulated protein |
| CWSNP1818 | Ca-Kabuli-Chr3 | 3701437 | [A/T] | Ca21344 | Intron | Gibberellin regulated protein |
| CWSNP1819 | Ca-Kabuli-Chr3 | 3701440 | [G/T] | Ca21344 | Intron | Gibberellin regulated protein |
| CWSNP1820 | Ca-Kabuli-Chr3 | 3701370 | [G/A] | Ca21344 | Intron | Gibberellin regulated protein |
| CWSNP1821 | Ca-Kabuli-Chr3 | 4145743 | [G/A] | Ca22385 | Intron | Domain of unknown function DUF298 |
| CWSNP1822 | Ca-Kabuli-Chr3 | 4178154 | [C/T] | - | Intergenic | - |
| CWSNP1823 | Ca-Kabuli-Chr3 | 4629747 | [A/C] | Ca22084 | Intron | - |
| CWSNP1824 | Ca-Kabuli-Chr3 | 5224781 | [A/G] | Ca23868 | Non-Synonymous-CDS | - |

| SNP IDs | Chromosomes | Physical positions (bp) | SNPs | Gene accession IDs | Sequence components of genes | Putative functions |
|---|---|---|---|---|---|---|
| CWSNP1825 | Ca-Kabuli-Chr3 | 5225108 | [T/G] | Ca23868 | Non-Synonymous-CDS | - |
| CWSNP1826 | Ca-Kabuli-Chr3 | 5436267 | [A/G] | - | Intergenic | - |
| CWSNP1827 | Ca-Kabuli-Chr3 | 5436272 | [T/C] | - | Intergenic | - |
| CWSNP1828 | Ca-Kabuli-Chr3 | 5436277 | [C/T] | - | Intergenic | - |
| CWSNP1829 | Ca-Kabuli-Chr3 | 5436288 | [G/T] | - | Intergenic | - |
| CWSNP1830 | Ca-Kabuli-Chr3 | 5436295 | [G/A] | - | Intergenic | - |
| CWSNP1831 | Ca-Kabuli-Chr3 | 5436296 | [C/T] | - | Intergenic | - |
| CWSNP1832 | Ca-Kabuli-Chr3 | 5436299 | [A/C] | - | Intergenic | - |
| CWSNP1833 | Ca-Kabuli-Chr3 | 5436331 | [C/A] | - | Intergenic | - |
| CWSNP1834 | Ca-Kabuli-Chr3 | 5514176 | [A/G] | - | Intergenic | - |
| CWSNP1835 | Ca-Kabuli-Chr3 | 5514287 | [C/A] | - | Intergenic | - |
| CWSNP1836 | Ca-Kabuli-Chr3 | 6169510 | [A/C] | - | Intergenic | - |
| CWSNP1837 | Ca-Kabuli-Chr3 | 6467014 | [G/A] | - | Intergenic | - |
| CWSNP1838 | Ca-Kabuli-Chr3 | 6683646 | [A/G] | Ca25772 | Intron | - |
| CWSNP1839 | Ca-Kabuli-Chr3 | 6683587 | [T/A] | Ca25772 | Intron | - |
| CWSNP1840 | Ca-Kabuli-Chr3 | 6828181 | [A/T] | - | Intergenic | - |

| SNP IDs | Chromosomes | Physical positions (bp) | SNPs | Gene accession IDs | Sequence components of genes | Putative functions |
|---|---|---|---|---|---|---|
| CWSNP1841 | Ca-Kabuli-Chr3 | 6840644 | [C/T] | Ca16550 | Synonymous-CDS | WD40 repeat |
| CWSNP1842 | Ca-Kabuli-Chr3 | 7015213 | [T/G] | Ca16540 | Non-Synonymous-CDS | Zinc finger,ZZ-type |
| CWSNP1843 | Ca-Kabuli-Chr3 | 7204053 | [G/A] | - | Intergenic | - |
| CWSNP1844 | Ca-Kabuli-Chr3 | 7204071 | [T/C] | - | Intergenic | - |
| CWSNP1845 | Ca-Kabuli-Chr3 | 7204087 | [C/T] | - | Intergenic | - |
| CWSNP1846 | Ca-Kabuli-Chr3 | 7204088 | [G/C] | - | Intergenic | - |
| CWSNP1847 | Ca-Kabuli-Chr3 | 7204098 | [G/A] | - | Intergenic | - |
| CWSNP1848 | Ca-Kabuli-Chr3 | 7204099 | [C/T] | - | Intergenic | - |
| CWSNP1849 | Ca-Kabuli-Chr3 | 7204111 | [G/A] | - | Intergenic | - |
| CWSNP1850 | Ca-Kabuli-Chr3 | 7204112 | [G/A] | - | Intergenic | - |
| CWSNP1851 | Ca-Kabuli-Chr3 | 7204133 | [G/T] | - | Intergenic | - |
| CWSNP1852 | Ca-Kabuli-Chr3 | 7226689 | [T/C] | Ca16534 | Non-Synonymous-CDS | PeptidaseC48,SUMO/Sentrin/Ubl1 |
| CWSNP1853 | Ca-Kabuli-Chr3 | 7226661 | [G/T] | Ca16534 | Non-Synonymous-CDS | PeptidaseC48,SUMO/Sentrin/Ubl1 |
| CWSNP1854 | Ca-Kabuli-Chr3 | 7226632 | [G/T] | Ca16534 | Non-Synonymous-CDS | PeptidaseC48,SUMO/Sentrin/Ubl1 |
| CWSNP1855 | Ca-Kabuli-Chr3 | 7420705 | [G/A] | - | Intergenic | - |
| CWSNP1856 | Ca-Kabuli-Chr3 | 7482871 | [C/T] | - | URR | - |

| SNP IDs | Chromosomes | Physical positions (bp) | SNPs | Gene accession IDs | Sequence components of genes | Putative functions |
|---|---|---|---|---|---|---|
| CWSNP1857 | Ca-Kabuli-Chr3 | 7606901 | [T/C] | - | DRR | - |
| CWSNP1858 | Ca-Kabuli-Chr3 | 7665123 | [G/A] | - | URR | - |
| CWSNP1859 | Ca-Kabuli-Chr3 | 7665112 | [C/T] | - | URR | - |
| CWSNP1860 | Ca-Kabuli-Chr3 | 7665098 | [C/T] | - | URR | - |
| CWSNP1861 | Ca-Kabuli-Chr3 | 7665090 | [C/T] | - | URR | - |
| CWSNP1862 | Ca-Kabuli-Chr3 | 7665075 | [G/A] | - | URR | - |
| CWSNP1863 | Ca-Kabuli-Chr3 | 7665156 | [G/A] | - | URR | - |
| CWSNP1864 | Ca-Kabuli-Chr3 | 7665149 | [T/G] | - | URR | - |
| CWSNP1865 | Ca-Kabuli-Chr3 | 7665142 | [G/A] | - | URR | - |
| CWSNP1866 | Ca-Kabuli-Chr3 | 7665157 | [C/T] | - | URR | - |
| CWSNP1867 | Ca-Kabuli-Chr3 | 8536213 | [T/C] | - | Intergenic | - |
| CWSNP1868 | Ca-Kabuli-Chr3 | 8536275 | [C/T] | - | Intergenic | - |
| CWSNP1869 | Ca-Kabuli-Chr3 | 8536295 | [A/T] | - | Intergenic | - |
| CWSNP1870 | Ca-Kabuli-Chr3 | 8536301 | [G/T] | - | Intergenic | - |
| CWSNP1871 | Ca-Kabuli-Chr3 | 8536425 | [C/A] | - | Intergenic | - |
| CWSNP1872 | Ca-Kabuli-Chr3 | 8536498 | [T/A] | - | Intergenic | - |

| SNP IDs | Chromosomes | Physical positions (bp) | SNPs | Gene accession IDs | Sequence components of genes | Putative functions |
|---|---|---|---|---|---|---|
| CWSNP1873 | Ca-Kabuli-Chr3 | 8668628 | [A/C] | - | Intergenic | - |
| CWSNP1874 | Ca-Kabuli-Chr3 | 8669004 | [A/T] | Ca23713 | Non-Synonymous-CDS | Protein kinase, catalytic domain |
| CWSNP1875 | Ca-Kabuli-Chr3 | 8669132 | [T/A] | Ca23713 | Synonymous-CDS | Protein kinase, catalytic domain |
| CWSNP1876 | Ca-Kabuli-Chr3 | 8697186 | [A/G] | - | DRR | - |
| CWSNP1877 | Ca-Kabuli-Chr3 | 8933037 | [T/C] | Ca25280 | Intron | ABC transporter-like |
| CWSNP1878 | Ca-Kabuli-Chr3 | 8933084 | [G/A] | Ca25280 | Intron | ABC transporter-like |
| CWSNP1879 | Ca-Kabuli-Chr3 | 8933086 | [C/A] | Ca25280 | Intron | ABC transporter-like |
| CWSNP1880 | Ca-Kabuli-Chr3 | 8933221 | [T/A] | Ca25280 | Intron | ABC transporter-like |
| CWSNP1881 | Ca-Kabuli-Chr3 | 8933183 | [A/G] | Ca25280 | Intron | ABC transporter-like |
| CWSNP1882 | Ca-Kabuli-Chr3 | 8933255 | [A/T] | Ca25280 | Intron | ABC transporter-like |
| CWSNP1883 | Ca-Kabuli-Chr3 | 8933266 | [G/T] | Ca25280 | Intron | ABC transporter-like |
| CWSNP1884 | Ca-Kabuli-Chr3 | 8933409 | [A/C] | Ca25280 | Intron | ABC transporter-like |
| CWSNP1885 | Ca-Kabuli-Chr3 | 8933370 | [A/G] | Ca25280 | Intron | ABC transporter-like |
| CWSNP1886 | Ca-Kabuli-Chr3 | 8933355 | [A/T] | Ca25280 | Intron | ABC transporter-like |
| CWSNP1887 | Ca-Kabuli-Chr3 | 9223875 | [G/A] | - | Intergenic | - |
| CWSNP1888 | Ca-Kabuli-Chr3 | 9296469 | [T/G] | Ca20960 | Synonymous-CDS | Arf GTPase activating protein |

| SNP IDs | Chromosomes | Physical positions (bp) | SNPs | Gene accession IDs | Sequence components of genes | Putative functions |
|---|---|---|---|---|---|---|
| CWSNP1889 | Ca-Kabuli-Chr3 | 9577402 | [G/A] | Ca20977 | Non-Synonymous-CDS | - |
| CWSNP1890 | Ca-Kabuli-Chr3 | 9579805 | [A/T] | - | Intergenic | - |
| CWSNP1891 | Ca-Kabuli-Chr3 | 9579808 | [C/T] | - | Intergenic | - |
| CWSNP1892 | Ca-Kabuli-Chr3 | 9579811 | [G/C] | - | Intergenic | - |
| CWSNP1893 | Ca-Kabuli-Chr3 | 9579817 | [G/A] | - | Intergenic | - |
| CWSNP1894 | Ca-Kabuli-Chr3 | 9579818 | [G/T] | - | Intergenic | - |
| CWSNP1895 | Ca-Kabuli-Chr3 | 9579824 | [A/G] | - | Intergenic | - |
| CWSNP1896 | Ca-Kabuli-Chr3 | 9579830 | [T/A] | - | Intergenic | - |
| CWSNP1897 | Ca-Kabuli-Chr3 | 9579831 | [C/G] | - | Intergenic | - |
| CWSNP1898 | Ca-Kabuli-Chr3 | 9894316 | [C/T] | - | Intergenic | - |
| CWSNP1899 | Ca-Kabuli-Chr3 | 10142152 | [C/A] | Ca18811 | Intron | WD40 repeat |
| CWSNP1900 | Ca-Kabuli-Chr3 | 10159836 | [A/C] | - | Intergenic | - |
| CWSNP1901 | Ca-Kabuli-Chr3 | 10159859 | [G/A] | - | Intergenic | - |
| CWSNP1902 | Ca-Kabuli-Chr3 | 10159941 | [C/A] | - | Intergenic | - |
| CWSNP1903 | Ca-Kabuli-Chr3 | 10159943 | [G/A] | - | Intergenic | - |
| CWSNP1904 | Ca-Kabuli-Chr3 | 10159944 | [T/C] | - | Intergenic | - |

| SNP IDs | Chromosomes | Physical positions (bp) | SNPs | Gene accession IDs | Sequence components of genes | Putative functions |
|---|---|---|---|---|---|---|
| CWSNP1905 | Ca-Kabuli-Chr3 | 10186893 | [G/C] | Ca18812 | Intron | - |
| CWSNP1906 | Ca-Kabuli-Chr3 | 10186861 | [G/T] | Ca18812 | Intron | - |
| CWSNP1907 | Ca-Kabuli-Chr3 | 10186857 | [A/G] | Ca18812 | Intron | - |
| CWSNP1908 | Ca-Kabuli-Chr3 | 10186880 | [A/G] | Ca18812 | Intron | - |
| CWSNP1909 | Ca-Kabuli-Chr3 | 10186889 | [G/A] | Ca18812 | Intron | - |
| CWSNP1910 | Ca-Kabuli-Chr3 | 10186891 | [C/G] | Ca18812 | Intron | - |
| CWSNP1911 | Ca-Kabuli-Chr3 | 10201361 | [G/T] | Ca18813 | Synonymous-CDS | VRR-NUC |
| CWSNP1912 | Ca-Kabuli-Chr3 | 10722780 | [G/T] | Ca18831 | Intron | - |
| CWSNP1913 | Ca-Kabuli-Chr3 | 10722859 | [G/A] | Ca18831 | Intron | - |
| CWSNP1914 | Ca-Kabuli-Chr3 | 11143751 | [G/A] | Ca19368 | Non-Synonymous-CDS | - |
| CWSNP1915 | Ca-Kabuli-Chr3 | 11143818 | [G/A] | Ca19368 | Synonymous-CDS | - |
| CWSNP1916 | Ca-Kabuli-Chr3 | 11378425 | [G/T] | Ca19376 | Non-Synonymous-CDS | ABC transporter, transmembrane domain |
| CWSNP1917 | Ca-Kabuli-Chr3 | 11590793 | [C/T] | - | DRR | - |
| CWSNP1918 | Ca-Kabuli-Chr3 | 11590874 | [A/G] | - | DRR | - |
| CWSNP1919 | Ca-Kabuli-Chr3 | 11590860 | [G/A] | - | DRR | - |
| CWSNP1920 | Ca-Kabuli-Chr3 | 11590832 | [G/A] | - | DRR | - |

| SNP IDs | Chromosomes | Physical positions (bp) | SNPs | Gene accession IDs | Sequence components of genes | Putative functions |
|---|---|---|---|---|---|---|
| CWSNP1921 | Ca-Kabuli-Chr3 | 11590814 | [T/C] | - | DRR | - |
| CWSNP1922 | Ca-Kabuli-Chr3 | 11610500 | [A/G] | - | Intergenic | - |
| CWSNP1923 | Ca-Kabuli-Chr3 | 11610460 | [A/G] | - | Intergenic | - |
| CWSNP1924 | Ca-Kabuli-Chr3 | 11610481 | [C/T] | - | Intergenic | - |
| CWSNP1925 | Ca-Kabuli-Chr3 | 11811685 | [A/G] | Ca19392 | Intron | Putativezinc-fingerdomain |
| CWSNP1926 | Ca-Kabuli-Chr3 | 12051826 | [A/C] | - | URR | - |
| CWSNP1927 | Ca-Kabuli-Chr3 | 12058576 | [T/C] | Ca22246 | Intron | - |
| CWSNP1928 | Ca-Kabuli-Chr3 | 12067458 | [A/C] | Ca22246 | Intron | - |
| CWSNP1929 | Ca-Kabuli-Chr3 | 12069092 | [T/G] | Ca22246 | Synonymous-CDS | - |
| CWSNP1930 | Ca-Kabuli-Chr3 | 12095571 | [T/G] | Ca22245 | Synonymous-CDS | Crotonase,core |
| CWSNP1931 | Ca-Kabuli-Chr3 | 12124099 | [G/A] | - | Intergenic | - |
| CWSNP1932 | Ca-Kabuli-Chr3 | 12394742 | [G/A] | - | Intergenic | - |
| CWSNP1933 | Ca-Kabuli-Chr3 | 12599496 | [A/C] | Ca21021 | Synonymous-CDS | Zinc finger,Dof-type |
| CWSNP1934 | Ca-Kabuli-Chr3 | 12622503 | [A/G] | - | Intergenic | - |
| CWSNP1935 | Ca-Kabuli-Chr3 | 12622534 | [C/T] | - | Intergenic | - |
| CWSNP1936 | Ca-Kabuli-Chr3 | 12622578 | [G/A] | - | Intergenic | - |

| SNP IDs | Chromosomes | Physical positions (bp) | SNPs | Gene accession IDs | Sequence components of genes | Putative functions |
|---|---|---|---|---|---|---|
| CWSNP1937 | Ca-Kabuli-Chr3 | 12622567 | [A/C] | - | Intergenic | - |
| CWSNP1938 | Ca-Kabuli-Chr3 | 12622543 | [G/A] | - | Intergenic | - |
| CWSNP1939 | Ca-Kabuli-Chr3 | 12627144 | [G/A] | - | Intergenic | - |
| CWSNP1940 | Ca-Kabuli-Chr3 | 12627143 | [T/C] | - | Intergenic | - |
| CWSNP1941 | Ca-Kabuli-Chr3 | 12809794 | [T/C] | Ca21015 | Non-Synonymous-CDS | Kinesin , motor domain |
| CWSNP1942 | Ca-Kabuli-Chr3 | 12814631 | [C/T] | - | DRR | - |
| CWSNP1943 | Ca-Kabuli-Chr3 | 12814603 | [T/C] | - | DRR | - |
| CWSNP1944 | Ca-Kabuli-Chr3 | 12814600 | [A/G] | - | DRR | - |
| CWSNP1945 | Ca-Kabuli-Chr3 | 12814599 | [A/T] | - | DRR | - |
| CWSNP1946 | Ca-Kabuli-Chr3 | 12814597 | [T/A] | - | DRR | - |
| CWSNP1947 | Ca-Kabuli-Chr3 | 12814571 | [C/A] | - | DRR | - |
| CWSNP1948 | Ca-Kabuli-Chr3 | 12814558 | [A/G] | - | DRR | - |
| CWSNP1949 | Ca-Kabuli-Chr3 | 12897831 | [C/A] | - | Intergenic | - |
| CWSNP1950 | Ca-Kabuli-Chr3 | 12897839 | [G/A] | - | Intergenic | - |
| CWSNP1951 | Ca-Kabuli-Chr3 | 12897841 | [C/A] | - | Intergenic | - |
| CWSNP1952 | Ca-Kabuli-Chr3 | 12897881 | [C/T] | - | Intergenic | - |

| SNP IDs | Chromosomes | Physical positions (bp) | SNPs | Gene accession IDs | Sequence components of genes | Putative functions |
|---|---|---|---|---|---|---|
| CWSNP1953 | Ca-Kabuli-Chr3 | 12897882 | [T/G] | - | Intergenic | - |
| CWSNP1954 | Ca-Kabuli-Chr3 | 12897926 | [A/C] | - | Intergenic | - |
| CWSNP1955 | Ca-Kabuli-Chr3 | 12897906 | [C/A] | - | Intergenic | - |
| CWSNP1956 | Ca-Kabuli-Chr3 | 12897887 | [T/C] | - | Intergenic | - |
| CWSNP1957 | Ca-Kabuli-Chr3 | 12897884 | [C/T] | - | Intergenic | - |
| CWSNP1958 | Ca-Kabuli-Chr3 | 12897883 | [G/T] | - | Intergenic | - |
| CWSNP1959 | Ca-Kabuli-Chr3 | 12897886 | [G/A] | - | Intergenic | - |
| CWSNP1960 | Ca-Kabuli-Chr3 | 13185541 | [G/A] | Ca22687 | Non-Synonymous-CDS | - |
| CWSNP1961 | Ca-Kabuli-Chr3 | 13185530 | [C/T] | Ca22687 | Non-Synonymous-CDS | - |
| CWSNP1962 | Ca-Kabuli-Chr3 | 13185490 | [G/A] | Ca22687 | Non-Synonymous-CDS | - |
| CWSNP1963 | Ca-Kabuli-Chr3 | 13185493 | [G/A] | Ca22687 | Non-Synonymous-CDS | - |
| CWSNP1964 | Ca-Kabuli-Chr3 | 13185508 | [C/T] | Ca22687 | Non-Synonymous-CDS | - |
| CWSNP1965 | Ca-Kabuli-Chr3 | 13185516 | [C/T] | Ca22687 | Synonymous-CDS | - |
| CWSNP1966 | Ca-Kabuli-Chr3 | 13185574 | [G/A] | Ca22687 | Non-Synonymous-CDS | - |
| CWSNP1967 | Ca-Kabuli-Chr3 | 13185560 | [G/A] | Ca22687 | Non-Synonymous-CDS | - |
| CWSNP1968 | Ca-Kabuli-Chr3 | 13185575 | [C/T] | Ca22687 | Non-Synonymous-CDS | - |

| SNP IDs | Chromosomes | Physical positions (bp) | SNPs | Gene accession IDs | Sequence components of genes | Putative functions |
|---|---|---|---|---|---|---|
| CWSNP1969 | Ca-Kabuli-Chr3 | 13185591 | [C/T] | Ca22687 | Synonymous-CDS | - |
| CWSNP1970 | Ca-Kabuli-Chr3 | 13396925 | [A/T] | Ca22695 | Intron | Glycosyltransferase,family2 |
| CWSNP1971 | Ca-Kabuli-Chr3 | 13397207 | [A/C] | Ca22695 | Intron | Glycosyltransferase,family2 |
| CWSNP1972 | Ca-Kabuli-Chr3 | 13617948 | [A/C] | Ca25319 | Synonymous-CDS | - |
| CWSNP1973 | Ca-Kabuli-Chr3 | 14200381 | [A/C] | - | Intergenic | - |
| CWSNP1974 | Ca-Kabuli-Chr3 | 14497374 | [C/A] | - | URR | - |
| CWSNP1975 | Ca-Kabuli-Chr3 | 14653195 | [G/T] | Ca22505 | Intron | Zinc finger,RING-type |
| CWSNP1976 | Ca-Kabuli-Chr3 | 14653194 | [C/T] | Ca22505 | Intron | Zinc finger,RING-type |
| CWSNP1977 | Ca-Kabuli-Chr3 | 14653193 | [T/G] | Ca22505 | Intron | Zinc finger,RING-type |
| CWSNP1978 | Ca-Kabuli-Chr3 | 14653270 | [C/A] | Ca22505 | Intron | Zinc finger,RING-type |
| CWSNP1979 | Ca-Kabuli-Chr3 | 14653271 | [G/A] | Ca22505 | Intron | Zinc finger,RING-type |
| CWSNP1980 | Ca-Kabuli-Chr3 | 14658175 | [A/C] | Ca22505 | Intron | Zinc finger,RING-type |
| CWSNP1981 | Ca-Kabuli-Chr3 | 14812960 | [A/C] | Ca22500 | Non-Synonymous-CDS | UDP-glucuronosyl/UDP-glucosyltransferase |
| CWSNP1982 | Ca-Kabuli-Chr3 | 16396673 | [A/C] | Ca18261 | Synonymous-CDS | Glyoxalase/bleomycin resistance protein/dioxygenase |
| CWSNP1983 | Ca-Kabuli-Chr3 | 17136071 | [G/C] | - | Intergenic | - |
| CWSNP1984 | Ca-Kabuli-Chr3 | 17136097 | [T/C] | - | Intergenic | - |

| SNP IDs | Chromosomes | Physical positions (bp) | SNPs | Gene accession IDs | Sequence components of genes | Putative functions |
|---|---|---|---|---|---|---|
| CWSNP1985 | Ca-Kabuli-Chr3 | 17334924 | [G/T] | - | Intergenic | - |
| CWSNP1986 | Ca-Kabuli-Chr3 | 17460012 | [C/A] | - | Intergenic | - |
| CWSNP1987 | Ca-Kabuli-Chr3 | 17679409 | [C/T] | Ca21115 | Synonymous-CDS | - |
| CWSNP1988 | Ca-Kabuli-Chr3 | 17679404 | [C/T] | Ca21115 | Synonymous-CDS | - |
| CWSNP1989 | Ca-Kabuli-Chr3 | 17914677 | [T/G] | - | Intergenic | - |
| CWSNP1990 | Ca-Kabuli-Chr3 | 17936842 | [A/G] | - | URR | - |
| CWSNP1991 | Ca-Kabuli-Chr3 | 17984978 | [T/G] | Ca21132 | Synonymous-CDS | Protein kinase, catalytic domain |
| CWSNP1992 | Ca-Kabuli-Chr3 | 17986521 | [G/C] | Ca21132 | Intron | Protein kinase, catalytic domain |
| CWSNP1993 | Ca-Kabuli-Chr3 | 17986525 | [G/T] | Ca21132 | Intron | Protein kinase, catalytic domain |
| CWSNP1994 | Ca-Kabuli-Chr3 | 18215321 | [G/C] | Ca19612 | Synonymous-CDS | Oligopeptide transporter |
| CWSNP1995 | Ca-Kabuli-Chr3 | 18215332 | [T/C] | Ca19612 | Non-Synonymous-CDS | Oligopeptide transporter |
| CWSNP1996 | Ca-Kabuli-Chr3 | 18355427 | [T/C] | Ca19620 | Synonymous-CDS | Protein of unknown function DUF3411 |
| CWSNP1997 | Ca-Kabuli-Chr3 | 18355445 | [T/C] | Ca19620 | Synonymous-CDS | Protein of unknown function DUF3411 |
| CWSNP1998 | Ca-Kabuli-Chr3 | 18375547 | [T/C] | - | Intergenic | - |
| CWSNP1999 | Ca-Kabuli-Chr3 | 18382404 | [T/C] | - | Intergenic | - |
| CWSNP2000 | Ca-Kabuli-Chr3 | 18383884 | [T/C] | - | URR | - |

| SNP IDs | Chromosomes | Physical positions (bp) | SNPs | Gene accession IDs | Sequence components of genes | Putative functions |
|---|---|---|---|---|---|---|
| CWSNP2001 | Ca-Kabuli-Chr3 | 18476006 | [A/G] | - | Intergenic | - |
| CWSNP2002 | Ca-Kabuli-Chr3 | 18476065 | [C/A] | - | Intergenic | - |
| CWSNP2003 | Ca-Kabuli-Chr3 | 19372722 | [G/T] | - | Intergenic | - |
| CWSNP2004 | Ca-Kabuli-Chr3 | 19442839 | [G/A] | - | Intergenic | - |
| CWSNP2005 | Ca-Kabuli-Chr3 | 19442884 | [G/C] | - | Intergenic | - |
| CWSNP2006 | Ca-Kabuli-Chr3 | 19445925 | [A/G] | - | Intergenic | - |
| CWSNP2007 | Ca-Kabuli-Chr3 | 19445920 | [T/A] | - | Intergenic | - |
| CWSNP2008 | Ca-Kabuli-Chr3 | 19475095 | [G/T] | - | Intergenic | - |
| CWSNP2009 | Ca-Kabuli-Chr3 | 19742600 | [G/A] | - | Intergenic | - |
| CWSNP2010 | Ca-Kabuli-Chr3 | 19742530 | [C/T] | - | Intergenic | - |
| CWSNP2011 | Ca-Kabuli-Chr3 | 19788512 | [G/A] | - | Intergenic | - |
| CWSNP2012 | Ca-Kabuli-Chr3 | 19802763 | [C/T] | Ca09383 | Intron | Adenosine/AMPdeaminase |
| CWSNP2013 | Ca-Kabuli-Chr3 | 19804985 | [T/C] | Ca09383 | Synonymous-CDS | Adenosine/AMPdeaminase |
| CWSNP2014 | Ca-Kabuli-Chr3 | 19805063 | [A/C] | Ca09383 | Synonymous-CDS | Adenosine/AMPdeaminase |
| CWSNP2015 | Ca-Kabuli-Chr3 | 19829510 | [G/T] | - | Intergenic | - |
| CWSNP2016 | Ca-Kabuli-Chr3 | 19850532 | [T/C] | - | Intergenic | - |

| SNP IDs | Chromosomes | Physical positions (bp) | SNPs | Gene accession IDs | Sequence components of genes | Putative functions |
|---|---|---|---|---|---|---|
| CWSNP2017 | Ca-Kabuli-Chr3 | 19850589 | [C/T] | - | Intergenic | - |
| CWSNP2018 | Ca-Kabuli-Chr3 | 19857613 | [T/A] | Ca09388 | Intron | PeptidaseM24,structural domain |
| CWSNP2019 | Ca-Kabuli-Chr3 | 19883927 | [A/G] | Ca09392 | Non-Synonymous-CDS | Importin-beta,N-terminal |
| CWSNP2020 | Ca-Kabuli-Chr3 | 19896800 | [C/T] | Ca09393 | Synonymous-CDS | PeptidaseC48,SUMO/Sentrin/Ubl1 |
| CWSNP2021 | Ca-Kabuli-Chr3 | 19907774 | [T/C] | - | Intergenic | - |
| CWSNP2022 | Ca-Kabuli-Chr3 | 19956343 | [C/T] | - | DRR | - |
| CWSNP2023 | Ca-Kabuli-Chr3 | 19956391 | [C/T] | Ca09398 | Intron | Ubiquitin |
| CWSNP2024 | Ca-Kabuli-Chr3 | 19978343 | [A/C] | Ca09399 | Non-Synonymous-CDS | Ammonium transporter |
| CWSNP2025 | Ca-Kabuli-Chr3 | 19978334 | [A/C] | Ca09399 | Non-Synonymous-CDS | Ammonium transporter |
| CWSNP2026 | Ca-Kabuli-Chr3 | 19978407 | [C/T] | Ca09399 | Synonymous-CDS | Ammonium transporter |
| CWSNP2027 | Ca-Kabuli-Chr3 | 19979288 | [A/T] | Ca09399 | Synonymous-CDS | Ammonium transporter |
| CWSNP2028 | Ca-Kabuli-Chr3 | 19979318 | [C/A] | Ca09399 | Synonymous-CDS | Ammonium transporter |
| CWSNP2029 | Ca-Kabuli-Chr3 | 20000967 | [C/G] | - | Intergenic | - |
| CWSNP2030 | Ca-Kabuli-Chr3 | 20030298 | [G/A] | - | Intergenic | - |
| CWSNP2031 | Ca-Kabuli-Chr3 | 20240118 | [T/G] | Ca09424 | Intron | CCAAT-binding factor |
| CWSNP2032 | Ca-Kabuli-Chr3 | 20242663 | [C/T] | Ca09424 | Intron | CCAAT-binding factor |

| SNP IDs | Chromosomes | Physical positions (bp) | SNPs | Gene accession IDs | Sequence components of genes | Putative functions |
|---|---|---|---|---|---|---|
| CWSNP2033 | Ca-Kabuli-Chr3 | 20365442 | [A/C] | - | Intergenic | - |
| CWSNP2034 | Ca-Kabuli-Chr3 | 20415615 | [A/G] | - | Intergenic | - |
| CWSNP2035 | Ca-Kabuli-Chr3 | 20552836 | [T/G] | Ca09446 | Non-Synonymous-CDS | - |
| CWSNP2036 | Ca-Kabuli-Chr3 | 20795014 | [C/T] | Ca09455 | Intron | Integrase,catalyticcore |
| CWSNP2037 | Ca-Kabuli-Chr3 | 20794976 | [A/C] | Ca09455 | Intron | Integrase,catalyticcore |
| CWSNP2038 | Ca-Kabuli-Chr3 | 20794993 | [A/G] | Ca09455 | Intron | Integrase,catalyticcore |
| CWSNP2039 | Ca-Kabuli-Chr3 | 20799811 | [C/T] | - | Intergenic | - |
| CWSNP2040 | Ca-Kabuli-Chr3 | 20799841 | [C/T] | - | Intergenic | - |
| CWSNP2041 | Ca-Kabuli-Chr3 | 20799820 | [A/G] | - | Intergenic | - |
| CWSNP2042 | Ca-Kabuli-Chr3 | 20829399 | [A/G] | Ca09459 | Intron | HEAT |
| CWSNP2043 | Ca-Kabuli-Chr3 | 21069672 | [A/G] | Ca09474 | Synonymous-CDS | Aminoacyl-tRNA synthetase,classII(D/K/N) |
| CWSNP2044 | Ca-Kabuli-Chr3 | 21069714 | [G/A] | Ca09474 | Intron | Aminoacyl-tRNA synthetase,classII(D/K/N) |
| CWSNP2045 | Ca-Kabuli-Chr3 | 21069916 | [C/T] | Ca09474 | Synonymous-CDS | Aminoacyl-tRNA synthetase,classII(D/K/N) |
| CWSNP2046 | Ca-Kabuli-Chr3 | 21069917 | [G/A] | Ca09474 | Non-Synonymous-CDS | Aminoacyl-tRNA synthetase,classII(D/K/N) |
| CWSNP2047 | Ca-Kabuli-Chr3 | 21069959 | [G/A] | Ca09474 | Intron | Aminoacyl-tRNA synthetase,classII(D/K/N) |
| CWSNP2048 | Ca-Kabuli-Chr3 | 21069986 | [A/C] | Ca09474 | Intron | Aminoacyl-tRNA synthetase,classII(D/K/N) |

| SNP IDs | Chromosomes | Physical positions (bp) | SNPs | Gene accession IDs | Sequence components of genes | Putative functions |
|---|---|---|---|---|---|---|
| CWSNP2049 | Ca-Kabuli-Chr3 | 21421082 | [A/G] | - | Intergenic | - |
| CWSNP2050 | Ca-Kabuli-Chr3 | 21421146 | [C/A] | - | Intergenic | - |
| CWSNP2051 | Ca-Kabuli-Chr3 | 21421162 | [G/A] | - | Intergenic | - |
| CWSNP2052 | Ca-Kabuli-Chr3 | 21421358 | [G/A] | - | Intergenic | - |
| CWSNP2053 | Ca-Kabuli-Chr3 | 21421362 | [T/A] | - | Intergenic | - |
| CWSNP2054 | Ca-Kabuli-Chr3 | 21421369 | [G/A] | - | Intergenic | - |
| CWSNP2055 | Ca-Kabuli-Chr3 | 21421424 | [G/A] | - | Intergenic | - |
| CWSNP2056 | Ca-Kabuli-Chr3 | 21421440 | [C/T] | - | Intergenic | - |
| CWSNP2057 | Ca-Kabuli-Chr3 | 21500178 | [G/A] | - | Intergenic | - |
| CWSNP2058 | Ca-Kabuli-Chr3 | 21500179 | [A/T] | - | Intergenic | - |
| CWSNP2059 | Ca-Kabuli-Chr3 | 21524296 | [G/T] | - | Intergenic | - |
| CWSNP2060 | Ca-Kabuli-Chr3 | 21524379 | [A/G] | - | Intergenic | - |
| CWSNP2061 | Ca-Kabuli-Chr3 | 21558357 | [T/G] | - | DRR | - |
| CWSNP2062 | Ca-Kabuli-Chr3 | 21687966 | [C/A] | Ca09523 | Non-Synonymous-CDS | - |
| CWSNP2063 | Ca-Kabuli-Chr3 | 21687961 | [C/A] | Ca09523 | Non-Synonymous-CDS | - |
| CWSNP2064 | Ca-Kabuli-Chr3 | 21687960 | [G/A] | Ca09523 | Non-Synonymous-CDS | - |

| SNP IDs | Chromosomes | Physical positions (bp) | SNPs | Gene accession IDs | Sequence components of genes | Putative functions |
|---|---|---|---|---|---|---|
| CWSNP2065 | Ca-Kabuli-Chr3 | 21687959 | [T/C] | Ca09523 | Non-Synonymous-CDS | - |
| CWSNP2066 | Ca-Kabuli-Chr3 | 21687956 | [C/T] | Ca09523 | Synonymous-CDS | - |
| CWSNP2067 | Ca-Kabuli-Chr3 | 21687953 | [C/T] | Ca09523 | Non-Synonymous-CDS | - |
| CWSNP2068 | Ca-Kabuli-Chr3 | 21809546 | [T/A] | - | Intergenic | - |
| CWSNP2069 | Ca-Kabuli-Chr3 | 21809503 | [T/C] | - | Intergenic | - |
| CWSNP2070 | Ca-Kabuli-Chr3 | 21950053 | [C/G] | Ca20431 | Intron | Domain of unknown function DUF296 |
| CWSNP2071 | Ca-Kabuli-Chr3 | 22008087 | [G/A] | Ca20426 | Non-Synonymous-CDS | SEC7-like |
| CWSNP2072 | Ca-Kabuli-Chr3 | 22026111 | [A/G] | Ca20423 | Intron | Armadillo |
| CWSNP2073 | Ca-Kabuli-Chr3 | 22062965 | [C/T] | Ca20420 | Intron | Forkhead-associated (FHA) domain |
| CWSNP2074 | Ca-Kabuli-Chr3 | 22200927 | [T/C] | Ca20410 | Synonymous-CDS | - |
| CWSNP2075 | Ca-Kabuli-Chr3 | 22253842 | [T/C] | Ca20405 | Intron | Glycosyltransferase,family8 |
| CWSNP2076 | Ca-Kabuli-Chr3 | 22346294 | [T/C] | Ca06276 | Non-Synonymous-CDS | PeptidaseS8/S53,subtilisin/kexin/sedolisin |
| CWSNP2077 | Ca-Kabuli-Chr3 | 22404660 | [G/T] | - | URR | - |
| CWSNP2078 | Ca-Kabuli-Chr3 | 22727393 | [G/A] | Ca06238 | Non-Synonymous-CDS | Malicoxido reductase |
| CWSNP2079 | Ca-Kabuli-Chr3 | 22727439 | [A/C] | Ca06238 | Synonymous-CDS | Malicoxido reductase |
| CWSNP2080 | Ca-Kabuli-Chr3 | 22765862 | [T/C] | - | DRR | - |

| SNP IDs | Chromosomes | Physical positions (bp) | SNPs | Gene accession IDs | Sequence components of genes | Putative functions |
|---|---|---|---|---|---|---|
| CWSNP2081 | Ca-Kabuli-Chr3 | 22765987 | [G/T] | - | DRR | - |
| CWSNP2082 | Ca-Kabuli-Chr3 | 22846051 | [G/A] | Ca06225 | Intron | RNA helicase, ATP-dependent, DEAD-box,conserved site |
| CWSNP2083 | Ca-Kabuli-Chr3 | 22990523 | [C/T] | Ca06210 | Non-Synonymous-CDS | Glycosidehydrolase,family14 |
| CWSNP2084 | Ca-Kabuli-Chr3 | 23042249 | [A/C] | Ca06206 | Non-Synonymous-CDS | Mediator complex,subunit Med12 |
| CWSNP2085 | Ca-Kabuli-Chr3 | 23053066 | [A/C] | - | Intergenic | - |
| CWSNP2086 | Ca-Kabuli-Chr3 | 23400864 | [A/C] | - | Intergenic | - |
| CWSNP2087 | Ca-Kabuli-Chr3 | 23455008 | [A/G] | - | Intergenic | - |
| CWSNP2088 | Ca-Kabuli-Chr3 | 23455128 | [A/G] | - | Intergenic | - |
| CWSNP2089 | Ca-Kabuli-Chr3 | 23461370 | [C/A] | - | URR | - |
| CWSNP2090 | Ca-Kabuli-Chr3 | 23593288 | [G/A] | - | DRR | - |
| CWSNP2091 | Ca-Kabuli-Chr3 | 23593294 | [T/C] | - | DRR | - |
| CWSNP2092 | Ca-Kabuli-Chr3 | 23593309 | [T/C] | - | DRR | - |
| CWSNP2093 | Ca-Kabuli-Chr3 | 23918105 | [T/C] | - | URR | - |
| CWSNP2094 | Ca-Kabuli-Chr3 | 23966110 | [G/A] | Ca06124 | Synonymous-CDS | DNA-binding WRKY |
| CWSNP2095 | Ca-Kabuli-Chr3 | 24024513 | [A/C] | - | DRR | - |
| CWSNP2096 | Ca-Kabuli-Chr3 | 24194574 | [C/T] | - | URR | - |

| SNP IDs | Chromosomes | Physical positions (bp) | SNPs | Gene accession IDs | Sequence components of genes | Putative functions |
|---|---|---|---|---|---|---|
| CWSNP2097 | Ca-Kabuli-Chr3 | 24297894 | [C/A] | Ca06091 | Non-Synonymous-CDS | - |
| CWSNP2098 | Ca-Kabuli-Chr3 | 24417507 | [A/T] | Ca06076 | Synonymous-CDS | Actin-binding FH2/DRF auto regulatory |
| CWSNP2099 | Ca-Kabuli-Chr3 | 24538726 | [A/C] | - | DRR | - |
| CWSNP2100 | Ca-Kabuli-Chr3 | 24637834 | [G/T] | - | DRR | - |
| CWSNP2101 | Ca-Kabuli-Chr3 | 24637837 | [A/C] | - | DRR | - |
| CWSNP2102 | Ca-Kabuli-Chr3 | 24664582 | [G/T] | - | DRR | - |
| CWSNP2103 | Ca-Kabuli-Chr3 | 24664550 | [T/G] | - | DRR | - |
| CWSNP2104 | Ca-Kabuli-Chr3 | 25241115 | [G/A] | Ca06005 | Non-Synonymous-CDS | DNArepairmetallo-beta-lactamase |
| CWSNP2105 | Ca-Kabuli-Chr3 | 25244194 | [A/C] | Ca06004 | Synonymous-CDS | Methyltransferasesmall |
| CWSNP2106 | Ca-Kabuli-Chr3 | 25462227 | [A/C] | Ca05988 | Non-Synonymous-CDS | Glycerophosphoryldiester phosphodiesterase |
| CWSNP2107 | Ca-Kabuli-Chr3 | 25504708 | [A/G] | - | Intergenic | - |
| CWSNP2108 | Ca-Kabuli-Chr3 | 25580072 | [A/T] | Ca05977 | Non-Synonymous-CDS | Nodulin-like |
| CWSNP2109 | Ca-Kabuli-Chr3 | 25614515 | [T/G] | - | Intergenic | - |
| CWSNP2110 | Ca-Kabuli-Chr3 | 25688341 | [G/A] | - | DRR | - |
| CWSNP2111 | Ca-Kabuli-Chr3 | 25718829 | [C/A] | - | URR | - |
| CWSNP2112 | Ca-Kabuli-Chr3 | 25718827 | [T/A] | - | URR | - |

| SNP IDs | Chromosomes | Physical positions (bp) | SNPs | Gene accession IDs | Sequence components of genes | Putative functions |
|---|---|---|---|---|---|---|
| CWSNP2113 | Ca-Kabuli-Chr3 | 25757693 | [C/T] | Ca23882 | Synonymous-CDS | SET domain |
| CWSNP2114 | Ca-Kabuli-Chr3 | 26163449 | [G/A] | Ca08289 | Non-Synonymous-CDS | Calcium-binding EF-hand |
| CWSNP2115 | Ca-Kabuli-Chr3 | 26335889 | [T/A] | Ca08268 | Synonymous-CDS | Exportin-1/Importin-beta-like |
| CWSNP2116 | Ca-Kabuli-Chr3 | 26339286 | [T/C] | Ca08268 | Non-Synonymous-CDS | Exportin-1/Importin-beta-like |
| CWSNP2117 | Ca-Kabuli-Chr3 | 26573902 | [A/G] | Ca08252 | Non-Synonymous-CDS | NotCCR4-Not complex component, N-terminal |
| CWSNP2118 | Ca-Kabuli-Chr3 | 26574831 | [A/G] | Ca08252 | Synonymous-CDS | NotCCR4-Not complex component, N-terminal |
| CWSNP2119 | Ca-Kabuli-Chr3 | 26627150 | [T/G] | Ca08246 | Intron | YTH domain |
| CWSNP2120 | Ca-Kabuli-Chr3 | 26678433 | [T/G] | - | DRR | - |
| CWSNP2121 | Ca-Kabuli-Chr3 | 26726897 | [C/A] | - | Intergenic | - |
| CWSNP2122 | Ca-Kabuli-Chr3 | 26758452 | [C/A] | Ca08231 | Non-Synonymous-CDS | HEAT |
| CWSNP2123 | Ca-Kabuli-Chr3 | 26848870 | [G/C] | Ca08222 | Non-Synonymous-CDS | Regulator of chromosome condensation, RCC1 |
| CWSNP2124 | Ca-Kabuli-Chr3 | 26849288 | [T/A] | Ca08222 | Non-Synonymous-CDS | Regulator of chromosome condensation, RCC1 |
| CWSNP2125 | Ca-Kabuli-Chr3 | 26878028 | [A/T] | - | Intergenic | - |
| CWSNP2126 | Ca-Kabuli-Chr3 | 26900271 | [T/A] | - | URR | - |
| CWSNP2127 | Ca-Kabuli-Chr3 | 26911443 | [A/T] | Ca08214 | Intron | Methionine synthase,vitamin-B12 independent |
| CWSNP2128 | Ca-Kabuli-Chr3 | 26911514 | [A/G] | Ca08214 | Intron | Methionine synthase,vitamin-B12 independent |

| SNP IDs | Chromosomes | Physical positions (bp) | SNPs | Gene accession IDs | Sequence components of genes | Putative functions |
|---|---|---|---|---|---|---|
| CWSNP2129 | Ca-Kabuli-Chr3 | 26911723 | [T/A] | Ca08214 | Intron | Methionine synthase,vitamin-B12 independent |
| CWSNP2130 | Ca-Kabuli-Chr3 | 26911792 | [A/G] | Ca08214 | Synonymous-CDS | Methionine synthase,vitamin-B12 independent |
| CWSNP2131 | Ca-Kabuli-Chr3 | 27047847 | [A/C] | Ca08199 | Synonymous-CDS | BTB/POZ-like |
| CWSNP2132 | Ca-Kabuli-Chr3 | 27076716 | [T/C] | - | DRR | - |
| CWSNP2133 | Ca-Kabuli-Chr3 | 27076735 | [T/G] | - | DRR | - |
| CWSNP2134 | Ca-Kabuli-Chr3 | 27076744 | [C/A] | - | DRR | - |
| CWSNP2135 | Ca-Kabuli-Chr3 | 27338521 | [T/C] | - | URR | - |
| CWSNP2136 | Ca-Kabuli-Chr3 | 27632100 | [G/A] | Ca08144 | Non-Synonymous-CDS | Myb,DNA-binding |
| CWSNP2137 | Ca-Kabuli-Chr3 | 27654798 | [A/C] | Ca08142 | Synonymous-CDS | RNA recognition motif domain |
| CWSNP2138 | Ca-Kabuli-Chr3 | 27673727 | [C/A] | Ca08139 | Non-Synonymous-CDS | - |
| CWSNP2139 | Ca-Kabuli-Chr3 | 27716396 | [C/T] | - | DRR | - |
| CWSNP2140 | Ca-Kabuli-Chr3 | 27877479 | [C/T] | Ca08121 | Synonymous-CDS | Ubiquitin-associated/translation elongation factor EF1B, N-terminal |
| CWSNP2141 | Ca-Kabuli-Chr3 | 27889107 | [A/G] | Ca08120 | Non-Synonymous-CDS | Ribosomal proteinS1,RNA-binding domain |
| CWSNP2142 | Ca-Kabuli-Chr3 | 27890849 | [C/T] | Ca08120 | Synonymous-CDS | Ribosomal proteinS1,RNA-binding domain |
| CWSNP2143 | Ca-Kabuli-Chr3 | 27969207 | [C/T] | Ca08113 | Non-Synonymous-CDS | Zinc finger,C2H2-type |
| CWSNP2144 | Ca-Kabuli-Chr3 | 28176380 | [A/C] | - | Intergenic | - |

| SNP IDs | Chromosomes | Physical positions (bp) | SNPs | Gene accession IDs | Sequence components of genes | Putative functions |
|---|---|---|---|---|---|---|
| CWSNP2145 | Ca-Kabuli-Chr3 | 28176333 | [C/A] | - | Intergenic | - |
| CWSNP2146 | Ca-Kabuli-Chr3 | 28375082 | [G/A] | Ca07151 | Intron | Transcription elongation factor,TFIIS/CRSP70,N-terminal,sub-type |
| CWSNP2147 | Ca-Kabuli-Chr3 | 28420948 | [C/T] | - | URR | - |
| CWSNP2148 | Ca-Kabuli-Chr3 | 28680858 | [C/T] | Ca07175 | Intron | Glutamine amidotransferase,class-II |
| CWSNP2149 | Ca-Kabuli-Chr3 | 28845958 | [A/C] | - | URR | - |
| CWSNP2150 | Ca-Kabuli-Chr3 | 28943989 | [G/T] | - | DRR | - |
| CWSNP2151 | Ca-Kabuli-Chr3 | 29056768 | [T/C] | Ca07211 | Synonymous-CDS | ATPase,AAA+type,core |
| CWSNP2152 | Ca-Kabuli-Chr3 | 29107776 | [A/G] | Ca07215 | Intron | Heat shock protein DnaJ,N-terminal |
| CWSNP2153 | Ca-Kabuli-Chr3 | 29155741 | [C/G] | Ca07220 | Intron | Rhoterminationfactor,N-terminal |
| CWSNP2154 | Ca-Kabuli-Chr3 | 29177885 | [A/C] | - | Intergenic | - |
| CWSNP2155 | Ca-Kabuli-Chr3 | 29207338 | [G/A] | - | Intergenic | - |
| CWSNP2156 | Ca-Kabuli-Chr3 | 29302251 | [C/T] | Ca07237 | Intron | Pathogenesis-related transcriptional factor/ERF, DNA-binding |
| CWSNP2157 | Ca-Kabuli-Chr3 | 29303144 | [A/C] | Ca07237 | Intron | Pathogenesis-related transcriptional factor/ERF, DNA-binding |
| CWSNP2158 | Ca-Kabuli-Chr3 | 29304116 | [A/G] | - | Intergenic | - |
| CWSNP2159 | Ca-Kabuli-Chr3 | 29508831 | [C/T] | Ca07256 | Non-Synonymous-CDS | Protein kinase, catalytic domain |
| CWSNP2160 | Ca-Kabuli-Chr3 | 29509310 | [G/T] | Ca07256 | Synonymous-CDS | Protein kinase, catalytic domain |

| SNP IDs | Chromosomes | Physical positions (bp) | SNPs | Gene accession IDs | Sequence components of genes | Putative functions |
|---|---|---|---|---|---|---|
| CWSNP2161 | Ca-Kabuli-Chr3 | 29603720 | [T/C] | - | DRR | - |
| CWSNP2162 | Ca-Kabuli-Chr3 | 29666633 | [C/T] | - | Intergenic | - |
| CWSNP2163 | Ca-Kabuli-Chr3 | 29698166 | [G/T] | - | Intergenic | - |
| CWSNP2164 | Ca-Kabuli-Chr3 | 29698289 | [G/A] | - | Intergenic | - |
| CWSNP2165 | Ca-Kabuli-Chr3 | 29698273 | [A/C] | - | Intergenic | - |
| CWSNP2166 | Ca-Kabuli-Chr3 | 29730019 | [G/T] | - | Intergenic | - |
| CWSNP2167 | Ca-Kabuli-Chr3 | 29744878 | [T/G] | Ca07282 | Synonymous-CDS | - |
| CWSNP2168 | Ca-Kabuli-Chr3 | 29803866 | [C/T] | - | DRR | - |
| CWSNP2169 | Ca-Kabuli-Chr3 | 29803962 | [A/G] | - | DRR | - |
| CWSNP2170 | Ca-Kabuli-Chr3 | 29807091 | [C/G] | - | DRR | - |
| CWSNP2171 | Ca-Kabuli-Chr3 | 29807248 | [A/G] | - | DRR | - |
| CWSNP2172 | Ca-Kabuli-Chr3 | 29836337 | [C/A] | - | Intergenic | - |
| CWSNP2173 | Ca-Kabuli-Chr3 | 29840029 | [C/G] | - | URR | - |
| CWSNP2174 | Ca-Kabuli-Chr3 | 29840028 | [T/C] | - | URR | - |
| CWSNP2175 | Ca-Kabuli-Chr3 | 29840022 | [C/A] | - | URR | - |
| CWSNP2176 | Ca-Kabuli-Chr3 | 29840012 | [A/T] | - | URR | - |

| SNP IDs | Chromosomes | Physical positions (bp) | SNPs | Gene accession IDs | Sequence components of genes | Putative functions |
|---|---|---|---|---|---|---|
| CWSNP2177 | Ca-Kabuli-Chr3 | 29844509 | [T/A] | Ca07292 | Intron | Basic-leucine zipper (bZIP) Transcription factor |
| CWSNP2178 | Ca-Kabuli-Chr3 | 29869190 | [G/T] | Ca07295 | Intron | CARP motif |
| CWSNP2179 | Ca-Kabuli-Chr3 | 29869381 | [G/A] | Ca07295 | Non-Synonymous-CDS | CARP motif |
| CWSNP2180 | Ca-Kabuli-Chr3 | 29869380 | [G/A] | Ca07295 | Non-Synonymous-CDS | CARP motif |
| CWSNP2181 | Ca-Kabuli-Chr3 | 29872643 | [A/G] | Ca07295 | Intron | CARP motif |
| CWSNP2182 | Ca-Kabuli-Chr3 | 29872682 | [G/A] | Ca07295 | Intron | CARP motif |
| CWSNP2183 | Ca-Kabuli-Chr3 | 29912488 | [A/G] | - | URR | - |
| CWSNP2184 | Ca-Kabuli-Chr3 | 29915510 | [G/A] | - | Intergenic | - |
| CWSNP2185 | Ca-Kabuli-Chr3 | 29932378 | [C/G] | Ca07304 | Intron | Myb,DNA-binding |
| CWSNP2186 | Ca-Kabuli-Chr3 | 29932504 | [T/A] | Ca07304 | Intron | Myb,DNA-binding |
| CWSNP2187 | Ca-Kabuli-Chr3 | 29932502 | [G/C] | Ca07304 | Intron | Myb,DNA-binding |
| CWSNP2188 | Ca-Kabuli-Chr3 | 29954498 | [G/A] | - | Intergenic | - |
| CWSNP2189 | Ca-Kabuli-Chr3 | 29954533 | [A/G] | - | Intergenic | - |
| CWSNP2190 | Ca-Kabuli-Chr3 | 29954648 | [A/T] | - | Intergenic | - |
| CWSNP2191 | Ca-Kabuli-Chr3 | 29954612 | [T/G] | - | Intergenic | - |
| CWSNP2192 | Ca-Kabuli-Chr3 | 29974532 | [A/G] | Ca07307 | Synonymous-CDS | Zinc finger,CCCH-type |

| SNP IDs | Chromosomes | Physical positions (bp) | SNPs | Gene accession IDs | Sequence components of genes | Putative functions |
|---|---|---|---|---|---|---|
| CWSNP2193 | Ca-Kabuli-Chr3 | 29974595 | [C/T] | Ca07307 | Synonymous-CDS | Zinc finger,CCCH-type |
| CWSNP2194 | Ca-Kabuli-Chr3 | 29982638 | [A/G] | - | URR | - |
| CWSNP2195 | Ca-Kabuli-Chr3 | 29982644 | [T/C] | - | URR | - |
| CWSNP2196 | Ca-Kabuli-Chr3 | 30004311 | [T/G] | - | DRR | - |
| CWSNP2197 | Ca-Kabuli-Chr3 | 30004295 | [A/G] | - | DRR | - |
| CWSNP2198 | Ca-Kabuli-Chr3 | 30009751 | [A/C] | - | URR | - |
| CWSNP2199 | Ca-Kabuli-Chr3 | 30011584 | [T/C] | - | URR | - |
| CWSNP2200 | Ca-Kabuli-Chr3 | 30011669 | [A/G] | - | URR | - |
| CWSNP2201 | Ca-Kabuli-Chr3 | 30011642 | [T/A] | - | URR | - |
| CWSNP2202 | Ca-Kabuli-Chr3 | 30011621 | [C/T] | - | URR | - |
| CWSNP2203 | Ca-Kabuli-Chr3 | 30029858 | [T/A] | Ca07318 | Non-Synonymous-CDS | NUDIX hydrolase domain |
| CWSNP2204 | Ca-Kabuli-Chr3 | 30029842 | [G/C] | Ca07318 | Intron | NUDIX hydrolase domain |
| CWSNP2205 | Ca-Kabuli-Chr3 | 30029817 | [T/G] | Ca07318 | Intron | NUDIX hydrolase domain |
| CWSNP2206 | Ca-Kabuli-Chr3 | 30084154 | [T/C] | - | Intergenic | - |
| CWSNP2207 | Ca-Kabuli-Chr3 | 30101476 | [A/C] | Ca07324 | Intron | Methyltransferase-related |
| CWSNP2208 | Ca-Kabuli-Chr3 | 30321177 | [C/G] | - | Intergenic | - |

| SNP IDs | Chromosomes | Physical positions (bp) | SNPs | Gene accession IDs | Sequence components of genes | Putative functions |
|---|---|---|---|---|---|---|
| CWSNP2209 | Ca-Kabuli-Chr3 | 30376319 | [G/A] | Ca07358 | Synonymous-CDS | Short-chain dehydrogenase/reductaseSDR |
| CWSNP2210 | Ca-Kabuli-Chr3 | 30385153 | [T/A] | Ca07359 | Intron | - |
| CWSNP2211 | Ca-Kabuli-Chr3 | 30397262 | [A/T] | Ca07359 | Intron | - |
| CWSNP2212 | Ca-Kabuli-Chr3 | 30510333 | [C/T] | - | Intergenic | - |
| CWSNP2213 | Ca-Kabuli-Chr3 | 30675468 | [T/G] | - | Intergenic | - |
| CWSNP2214 | Ca-Kabuli-Chr3 | 30837284 | [C/T] | - | DRR | - |
| CWSNP2215 | Ca-Kabuli-Chr3 | 30837265 | [T/C] | - | DRR | - |
| CWSNP2216 | Ca-Kabuli-Chr3 | 31267639 | [T/A] | - | DRR | - |
| CWSNP2217 | Ca-Kabuli-Chr3 | 31267675 | [A/G] | - | DRR | - |
| CWSNP2218 | Ca-Kabuli-Chr3 | 31267681 | [G/A] | - | DRR | - |
| CWSNP2219 | Ca-Kabuli-Chr3 | 31267828 | [A/G] | - | DRR | - |
| CWSNP2220 | Ca-Kabuli-Chr3 | 31267927 | [T/G] | - | DRR | - |
| CWSNP2221 | Ca-Kabuli-Chr3 | 31268143 | [A/G] | - | DRR | - |
| CWSNP2222 | Ca-Kabuli-Chr3 | 31279616 | [T/A] | Ca12224 | Intron | Protein of unknown function DUF647 |
| CWSNP2223 | Ca-Kabuli-Chr3 | 31279606 | [G/A] | Ca12224 | Intron | Protein of unknown function DUF647 |
| CWSNP2224 | Ca-Kabuli-Chr3 | 31279605 | [G/A] | Ca12224 | Intron | Protein of unknown function DUF647 |

| SNP IDs | Chromosomes | Physical positions (bp) | SNPs | Gene accession IDs | Sequence components of genes | Putative functions |
|---|---|---|---|---|---|---|
| CWSNP2225 | Ca-Kabuli-Chr3 | 31287909 | [A/G] | Ca12225 | Synonymous-CDS | General substrate transporter |
| CWSNP2226 | Ca-Kabuli-Chr3 | 31288143 | [T/C] | Ca12225 | Synonymous-CDS | General substrate transporter |
| CWSNP2227 | Ca-Kabuli-Chr3 | 31301783 | [A/G] | - | DRR | - |
| CWSNP2228 | Ca-Kabuli-Chr3 | 31302060 | [T/C] | - | DRR | - |
| CWSNP2229 | Ca-Kabuli-Chr3 | 31313021 | [G/T] | - | URR | - |
| CWSNP2230 | Ca-Kabuli-Chr3 | 31317521 | [T/G] | - | URR | - |
| CWSNP2231 | Ca-Kabuli-Chr3 | 31321725 | [A/G] | - | URR | - |
| CWSNP2232 | Ca-Kabuli-Chr3 | 31321778 | [G/A] | - | URR | - |
| CWSNP2233 | Ca-Kabuli-Chr3 | 31321850 | [A/G] | - | URR | - |
| CWSNP2234 | Ca-Kabuli-Chr3 | 31341335 | [C/T] | Ca12234 | Synonymous-CDS | Domain of unknown function DUF640 |
| CWSNP2235 | Ca-Kabuli-Chr3 | 31342119 | [A/T] | - | Intergenic | - |
| CWSNP2236 | Ca-Kabuli-Chr3 | 31354988 | [G/C] | - | DRR | - |
| CWSNP2237 | Ca-Kabuli-Chr3 | 31355001 | [C/T] | - | DRR | - |
| CWSNP2238 | Ca-Kabuli-Chr3 | 31355026 | [T/C] | - | DRR | - |
| CWSNP2239 | Ca-Kabuli-Chr3 | 31431216 | [C/T] | Ca12240 | Intron | Protein kinase, catalytic domain |
| CWSNP2240 | Ca-Kabuli-Chr3 | 31478804 | [T/C] | Ca12246 | Synonymous-CDS | Glucose-methanol-cholineoxidoreductase,N-terminal |

| SNP IDs | Chromosomes | Physical positions (bp) | SNPs | Gene accession IDs | Sequence components of genes | Putative functions |
|---|---|---|---|---|---|---|
| CWSNP2241 | Ca-Kabuli-Chr3 | 31478882 | [C/T] | Ca12246 | Synonymous-CDS | Glucose-methanol-cholineoxidoreductase,N-terminal |
| CWSNP2242 | Ca-Kabuli-Chr3 | 31480603 | [T/C] | - | URR | - |
| CWSNP2243 | Ca-Kabuli-Chr3 | 31516662 | [G/T] | - | URR | - |
| CWSNP2244 | Ca-Kabuli-Chr3 | 31516766 | [T/C] | - | URR | - |
| CWSNP2245 | Ca-Kabuli-Chr3 | 31520119 | [C/T] | - | Intergenic | - |
| CWSNP2246 | Ca-Kabuli-Chr3 | 31520225 | [A/T] | - | URR | - |
| CWSNP2247 | Ca-Kabuli-Chr3 | 31520198 | [G/A] | - | URR | - |
| CWSNP2248 | Ca-Kabuli-Chr3 | 31520190 | [T/C] | - | Intergenic | - |
| CWSNP2249 | Ca-Kabuli-Chr3 | 31521142 | [T/C] | - | URR | - |
| CWSNP2250 | Ca-Kabuli-Chr3 | 31521155 | [T/A] | - | URR | - |
| CWSNP2251 | Ca-Kabuli-Chr3 | 31589355 | [A/G] | Ca12258 | Intron | SET domain |
| CWSNP2252 | Ca-Kabuli-Chr3 | 31589329 | [C/T] | Ca12258 | Intron | SET domain |
| CWSNP2253 | Ca-Kabuli-Chr3 | 31630195 | [T/C] | Ca12262 | Intron | RNA recognition motif domain |
| CWSNP2254 | Ca-Kabuli-Chr3 | 31631451 | [A/C] | Ca12262 | Non-Synonymous-CDS | RNA recognition motif domain |
| CWSNP2255 | Ca-Kabuli-Chr3 | 31631561 | [G/A] | Ca12262 | Synonymous-CDS | RNA recognition motif domain |
| CWSNP2256 | Ca-Kabuli-Chr3 | 31631534 | [A/G] | Ca12262 | Synonymous-CDS | RNA recognition motif domain |

| SNP IDs | Chromosomes | Physical positions (bp) | SNPs | Gene accession IDs | Sequence components of genes | Putative functions |
|---|---|---|---|---|---|---|
| CWSNP2257 | Ca-Kabuli-Chr3 | 31631622 | [G/A] | Ca12262 | Intron | RNA recognition motif domain |
| CWSNP2258 | Ca-Kabuli-Chr3 | 31631644 | [A/C] | Ca12262 | Intron | RNA recognition motif domain |
| CWSNP2259 | Ca-Kabuli-Chr3 | 31646802 | [G/A] | - | Intergenic | - |
| CWSNP2260 | Ca-Kabuli-Chr3 | 31683906 | [A/G] | Ca12270 | Non-Synonymous-CDS | PeptidaseS8/S53,subtilisin/kexin/sedolisin |
| CWSNP2261 | Ca-Kabuli-Chr3 | 31689128 | [C/T] | - | URR | - |
| CWSNP2262 | Ca-Kabuli-Chr3 | 31698493 | [A/C] | Ca12272 | Intron | ABC transporter, transmembrane domain |
| CWSNP2263 | Ca-Kabuli-Chr3 | 31771314 | [C/A] | - | URR | - |
| CWSNP2264 | Ca-Kabuli-Chr3 | 31931014 | [A/C] | Ca12294 | Synonymous-CDS | - |
| CWSNP2265 | Ca-Kabuli-Chr3 | 31951994 | [G/A] | Ca12295 | Synonymous-CDS | HEAT |
| CWSNP2266 | Ca-Kabuli-Chr3 | 31953466 | [G/A] | Ca12295 | Intron | HEAT |
| CWSNP2267 | Ca-Kabuli-Chr3 | 32351052 | [C/T] | Ca11958 | Synonymous-CDS | Heat shock protein Hsp70 |
| CWSNP2268 | Ca-Kabuli-Chr3 | 32351031 | [G/C] | Ca11958 | Synonymous-CDS | Heat shock protein Hsp70 |
| CWSNP2269 | Ca-Kabuli-Chr3 | 32357599 | [C/T] | Ca11959 | Synonymous-CDS | Heat shock protein Hsp70 |
| CWSNP2270 | Ca-Kabuli-Chr3 | 32357578 | [G/C] | Ca11959 | Synonymous-CDS | Heat shock protein Hsp70 |
| CWSNP2271 | Ca-Kabuli-Chr3 | 32510033 | [T/G] | - | Intergenic | - |
| CWSNP2272 | Ca-Kabuli-Chr3 | 32510090 | [A/G] | - | Intergenic | - |

| SNP IDs | Chromosomes | Physical positions (bp) | SNPs | Gene accession IDs | Sequence components of genes | Putative functions |
|---|---|---|---|---|---|---|
| CWSNP2273 | Ca-Kabuli-Chr3 | 32510124 | [C/T] | - | Intergenic | - |
| CWSNP2274 | Ca-Kabuli-Chr3 | 32510111 | [C/T] | - | Intergenic | - |
| CWSNP2275 | Ca-Kabuli-Chr3 | 32510071 | [G/T] | - | Intergenic | - |
| CWSNP2276 | Ca-Kabuli-Chr3 | 32510081 | [C/T] | - | Intergenic | - |
| CWSNP2277 | Ca-Kabuli-Chr3 | 32514908 | [A/G] | - | Intergenic | - |
| CWSNP2278 | Ca-Kabuli-Chr3 | 32514889 | [C/T] | - | Intergenic | - |
| CWSNP2279 | Ca-Kabuli-Chr3 | 32514868 | [A/G] | - | Intergenic | - |
| CWSNP2280 | Ca-Kabuli-Chr3 | 32514867 | [C/T] | - | Intergenic | - |
| CWSNP2281 | Ca-Kabuli-Chr3 | 32514859 | [C/T] | - | Intergenic | - |
| CWSNP2282 | Ca-Kabuli-Chr3 | 32525128 | [G/T] | Ca11978 | Non-Synonymous-CDS | Ras GTPase |
| CWSNP2283 | Ca-Kabuli-Chr3 | 32663059 | [A/C] | Ca11994 | Non-Synonymous-CDS | RNA recognition motif domain |
| CWSNP2284 | Ca-Kabuli-Chr3 | 32827067 | [C/G] | - | URR | - |
| CWSNP2285 | Ca-Kabuli-Chr3 | 32827065 | [T/G] | - | URR | - |
| CWSNP2286 | Ca-Kabuli-Chr3 | 32845395 | [C/A] | - | DRR | - |
| CWSNP2287 | Ca-Kabuli-Chr3 | 32865530 | [C/G] | Ca12023 | Intron | Helicase,C-terminal |
| CWSNP2288 | Ca-Kabuli-Chr3 | 33091737 | [A/C] | Ca12046 | Intron | SNF2-related |

| SNP IDs | Chromosomes | Physical positions (bp) | SNPs | Gene accession IDs | Sequence components of genes | Putative functions |
|---|---|---|---|---|---|---|
| CWSNP2289 | Ca-Kabuli-Chr3 | 33120825 | [G/A] | - | DRR | - |
| CWSNP2290 | Ca-Kabuli-Chr3 | 33520615 | [A/C] | Ca23669 | Non-Synonymous-CDS | - |
| CWSNP2291 | Ca-Kabuli-Chr3 | 33522974 | [G/A] | Ca23669 | Intron | - |
| CWSNP2292 | Ca-Kabuli-Chr3 | 33627826 | [A/C] | - | Intergenic | - |
| CWSNP2293 | Ca-Kabuli-Chr3 | 33715215 | [G/T] | Ca20869 | Intron | Alpha/beta hydrolase fold-1 |
| CWSNP2294 | Ca-Kabuli-Chr3 | 33900522 | [A/G] | Ca20884 | Intron | ABC transporter, transmembrane domain |
| CWSNP2295 | Ca-Kabuli-Chr3 | 33904394 | [T/A] | - | Intergenic | - |
| CWSNP2296 | Ca-Kabuli-Chr3 | 34247736 | [T/G] | Ca00702 | Non-Synonymous-CDS | ABC transporter, transmembrane domain |
| CWSNP2297 | Ca-Kabuli-Chr3 | 34352385 | [A/C] | - | URR | - |
| CWSNP2298 | Ca-Kabuli-Chr3 | 34496982 | [G/C] | Ca00725 | Synonymous-CDS | WD40 repeat |
| CWSNP2299 | Ca-Kabuli-Chr3 | 34524656 | [C/A] | Ca00726 | Non-Synonymous-CDS | - |
| CWSNP2300 | Ca-Kabuli-Chr3 | 34543039 | [C/T] | Ca00726 | Non-Synonymous-CDS | - |
| CWSNP2301 | Ca-Kabuli-Chr3 | 34543111 | [A/G] | Ca00726 | Non-Synonymous-CDS | - |
| CWSNP2302 | Ca-Kabuli-Chr3 | 34547052 | [A/T] | Ca00726 | Non-Synonymous-CDS | - |
| CWSNP2303 | Ca-Kabuli-Chr3 | 34547069 | [G/T] | Ca00726 | Non-Synonymous-CDS | - |
| CWSNP2304 | Ca-Kabuli-Chr3 | 34603669 | [T/A] | Ca00732 | Non-Synonymous-CDS | Zinc finger,PHD-type |

| SNP IDs | Chromosomes | Physical positions (bp) | SNPs | Gene accession IDs | Sequence components of genes | Putative functions |
|---|---|---|---|---|---|---|
| CWSNP2305 | Ca-Kabuli-Chr3 | 34662736 | [T/G] | Ca00740 | Intron | RNA recognition motif domain |
| CWSNP2306 | Ca-Kabuli-Chr3 | 34699114 | [C/A] | Ca00745 | Non-Synonymous-CDS | HECT |
| CWSNP2307 | Ca-Kabuli-Chr3 | 34699076 | [T/C] | Ca00745 | Non-Synonymous-CDS | HECT |
| CWSNP2308 | Ca-Kabuli-Chr3 | 34722508 | [A/T] | - | Intergenic | - |
| CWSNP2309 | Ca-Kabuli-Chr3 | 34722931 | [A/G] | - | URR | - |
| CWSNP2310 | Ca-Kabuli-Chr3 | 34722979 | [T/C] | - | URR | - |
| CWSNP2311 | Ca-Kabuli-Chr3 | 34730463 | [T/C] | Ca00748 | Intron | Protein of unknown function DUF81 |
| CWSNP2312 | Ca-Kabuli-Chr3 | 34730954 | [G/A] | - | Intergenic | - |
| CWSNP2313 | Ca-Kabuli-Chr3 | 34852030 | [G/A] | - | DRR | - |
| CWSNP2314 | Ca-Kabuli-Chr3 | 34852070 | [T/G] | - | DRR | - |
| CWSNP2315 | Ca-Kabuli-Chr3 | 34889316 | [C/G] | Ca00767 | Synonymous-CDS | Fructose-bisphosphatealdolase,class-I |
| CWSNP2316 | Ca-Kabuli-Chr3 | 34911621 | [G/A] | Ca00768 | Synonymous-CDS | Pathogenesis-related transcriptional factor/ERF, DNA-binding |
| CWSNP2317 | Ca-Kabuli-Chr3 | 35071821 | [A/G] | Ca00779 | Intron | Protein kinase, catalytic domain |
| CWSNP2318 | Ca-Kabuli-Chr3 | 35071852 | [T/G] | Ca00779 | Intron | Protein kinase, catalytic domain |
| CWSNP2319 | Ca-Kabuli-Chr3 | 35085559 | [T/A] | Ca00781 | Synonymous-CDS | PeptidaseS8/S53,subtilisin/kexin/sedolisin |
| CWSNP2320 | Ca-Kabuli-Chr3 | 35085658 | [C/A] | Ca00781 | Synonymous-CDS | PeptidaseS8/S53,subtilisin/kexin/sedolisin |

| SNP IDs | Chromosomes | Physical positions (bp) | SNPs | Gene accession IDs | Sequence components of genes | Putative functions |
|---|---|---|---|---|---|---|
| CWSNP2321 | Ca-Kabuli-Chr3 | 35126649 | [C/G] | Ca00786 | Non-Synonymous-CDS | - |
| CWSNP2322 | Ca-Kabuli-Chr3 | 35126760 | [G/A] | Ca00786 | Non-Synonymous-CDS | - |
| CWSNP2323 | Ca-Kabuli-Chr3 | 35332088 | [T/A] | Ca00812 | Non-Synonymous-CDS | Kinesin , motor domain |
| CWSNP2324 | Ca-Kabuli-Chr3 | 35383318 | [C/T] | Ca00819 | Intron | Peptidyl-prolylcis-transisomerase,cyclophilin-type |
| CWSNP2325 | Ca-Kabuli-Chr3 | 35403074 | [A/G] | - | DRR | - |
| CWSNP2326 | Ca-Kabuli-Chr3 | 35420108 | [T/A] | Ca00824 | Intron | LETM1-like |
| CWSNP2327 | Ca-Kabuli-Chr3 | 35424994 | [T/C] | - | DRR | - |
| CWSNP2328 | Ca-Kabuli-Chr3 | 35483932 | [T/C] | - | DRR | - |
| CWSNP2329 | Ca-Kabuli-Chr3 | 35483960 | [T/C] | - | DRR | - |
| CWSNP2330 | Ca-Kabuli-Chr3 | 35484034 | [G/C] | - | DRR | - |
| CWSNP2331 | Ca-Kabuli-Chr3 | 35487736 | [T/C] | - | Intergenic | - |
| CWSNP2332 | Ca-Kabuli-Chr3 | 35488597 | [T/C] | - | URR | - |
| CWSNP2333 | Ca-Kabuli-Chr3 | 35490991 | [A/G] | - | URR | - |
| CWSNP2334 | Ca-Kabuli-Chr3 | 35490951 | [C/T] | - | URR | - |
| CWSNP2335 | Ca-Kabuli-Chr3 | 35543614 | [C/T] | - | DRR | - |
| CWSNP2336 | Ca-Kabuli-Chr3 | 35631679 | [C/G] | - | URR | - |

| SNP IDs | Chromosomes | Physical positions (bp) | SNPs | Gene accession IDs | Sequence components of genes | Putative functions |
|---|---|---|---|---|---|---|
| CWSNP2337 | Ca-Kabuli-Chr3 | 35631689 | [C/A] | - | URR | - |
| CWSNP2338 | Ca-Kabuli-Chr3 | 35631723 | [A/G] | - | URR | - |
| CWSNP2339 | Ca-Kabuli-Chr3 | 35747394 | [C/G] | - | Intergenic | - |
| CWSNP2340 | Ca-Kabuli-Chr3 | 35807265 | [T/C] | - | DRR | - |
| CWSNP2341 | Ca-Kabuli-Chr3 | 35807407 | [A/T] | - | DRR | - |
| CWSNP2342 | Ca-Kabuli-Chr3 | 35829834 | [A/G] | - | URR | - |
| CWSNP2343 | Ca-Kabuli-Chr3 | 35920684 | [A/C] | - | URR | - |
| CWSNP2344 | Ca-Kabuli-Chr3 | 35948487 | [G/A] | - | Intergenic | - |
| CWSNP2345 | Ca-Kabuli-Chr3 | 35979412 | [G/T] | Ca00892 | Synonymous-CDS | FAD-linkedoxidase,C-terminal |
| CWSNP2346 | Ca-Kabuli-Chr3 | 36003699 | [G/A] | Ca00896 | Intron | Protease-associated domain,PA |
| CWSNP2347 | Ca-Kabuli-Chr3 | 36003682 | [G/T] | Ca00896 | Intron | Protease-associated domain,PA |
| CWSNP2348 | Ca-Kabuli-Chr3 | 36004668 | [A/G] | Ca00896 | Intron | Protease-associated domain,PA |
| CWSNP2349 | Ca-Kabuli-Chr3 | 36046938 | [C/T] | Ca00903 | Synonymous-CDS | SWAP/Surp |
| CWSNP2350 | Ca-Kabuli-Chr3 | 36046956 | [A/C] | Ca00903 | Synonymous-CDS | SWAP/Surp |
| CWSNP2351 | Ca-Kabuli-Chr3 | 36050259 | [T/G] | Ca00903 | Synonymous-CDS | SWAP/Surp |
| CWSNP2352 | Ca-Kabuli-Chr3 | 36050319 | [T/C] | Ca00903 | Synonymous-CDS | SWAP/Surp |

| SNP IDs | Chromosomes | Physical positions (bp) | SNPs | Gene accession IDs | Sequence components of genes | Putative functions |
|---|---|---|---|---|---|---|
| CWSNP2353 | Ca-Kabuli-Chr3 | 36071033 | [A/G] | - | Intergenic | - |
| CWSNP2354 | Ca-Kabuli-Chr3 | 36085966 | [G/A] | - | DRR | - |
| CWSNP2355 | Ca-Kabuli-Chr3 | 36117521 | [A/G] | Ca00911 | Synonymous-CDS | - |
| CWSNP2356 | Ca-Kabuli-Chr3 | 36118120 | [T/C] | Ca00911 | Intron | - |
| CWSNP2357 | Ca-Kabuli-Chr3 | 36118156 | [C/T] | Ca00911 | Intron | - |
| CWSNP2358 | Ca-Kabuli-Chr3 | 36119592 | [T/C] | Ca00911 | Non-Synonymous-CDS | - |
| CWSNP2359 | Ca-Kabuli-Chr3 | 36119602 | [C/A] | Ca00911 | Non-Synonymous-CDS | - |
| CWSNP2360 | Ca-Kabuli-Chr3 | 36130561 | [T/A] | - | Intergenic | - |
| CWSNP2361 | Ca-Kabuli-Chr3 | 36130610 | [C/A] | - | Intergenic | - |
| CWSNP2362 | Ca-Kabuli-Chr3 | 36132440 | [T/C] | Ca00912 | Intron | NPL4 |
| CWSNP2363 | Ca-Kabuli-Chr3 | 36132437 | [C/T] | Ca00912 | Intron | NPL4 |
| CWSNP2364 | Ca-Kabuli-Chr3 | 36132401 | [C/T] | Ca00912 | Intron | NPL4 |
| CWSNP2365 | Ca-Kabuli-Chr3 | 36132480 | [C/T] | Ca00912 | Intron | NPL4 |
| CWSNP2366 | Ca-Kabuli-Chr3 | 36132539 | [C/T] | Ca00912 | Intron | NPL4 |
| CWSNP2367 | Ca-Kabuli-Chr3 | 36132586 | [T/G] | Ca00912 | Intron | NPL4 |
| CWSNP2368 | Ca-Kabuli-Chr3 | 36132570 | [G/A] | Ca00912 | Intron | NPL4 |

| SNP IDs | Chromosomes | Physical positions (bp) | SNPs | Gene accession IDs | Sequence components of genes | Putative functions |
|---|---|---|---|---|---|---|
| CWSNP2369 | Ca-Kabuli-Chr3 | 36132554 | [A/T] | Ca00912 | Intron | NPL4 |
| CWSNP2370 | Ca-Kabuli-Chr3 | 36150722 | [G/A] | - | URR | - |
| CWSNP2371 | Ca-Kabuli-Chr3 | 36150717 | [T/C] | - | URR | - |
| CWSNP2372 | Ca-Kabuli-Chr3 | 36167136 | [G/T] | - | Intergenic | - |
| CWSNP2373 | Ca-Kabuli-Chr3 | 36177201 | [T/C] | - | Intergenic | - |
| CWSNP2374 | Ca-Kabuli-Chr3 | 36205806 | [T/G] | - | Intergenic | - |
| CWSNP2375 | Ca-Kabuli-Chr3 | 36205819 | [A/G] | - | Intergenic | - |
| CWSNP2376 | Ca-Kabuli-Chr3 | 36205845 | [A/G] | - | Intergenic | - |
| CWSNP2377 | Ca-Kabuli-Chr3 | 36206615 | [A/C] | Ca00925 | Intron | rRNA processing |
| CWSNP2378 | Ca-Kabuli-Chr3 | 36212809 | [G/A] | - | URR | - |
| CWSNP2379 | Ca-Kabuli-Chr3 | 36212843 | [T/A] | - | URR | - |
| CWSNP2380 | Ca-Kabuli-Chr3 | 36217686 | [G/A] | Ca00927 | Intron | Zinc finger,B-box |
| CWSNP2381 | Ca-Kabuli-Chr3 | 36227565 | [A/T] | - | Intergenic | - |
| CWSNP2382 | Ca-Kabuli-Chr3 | 36227566 | [A/T] | - | Intergenic | - |
| CWSNP2383 | Ca-Kabuli-Chr3 | 36227948 | [A/G] | - | Intergenic | - |
| CWSNP2384 | Ca-Kabuli-Chr3 | 36228032 | [G/T] | - | Intergenic | - |

| SNP IDs | Chromosomes | Physical positions (bp) | SNPs | Gene accession IDs | Sequence components of genes | Putative functions |
|---|---|---|---|---|---|---|
| CWSNP2385 | Ca-Kabuli-Chr3 | 36228121 | [T/A] | Ca00929 | Intron | Ribosomal proteinS19/S15 |
| CWSNP2386 | Ca-Kabuli-Chr3 | 36242504 | [C/T] | - | URR | - |
| CWSNP2387 | Ca-Kabuli-Chr3 | 36242490 | [G/T] | - | URR | - |
| CWSNP2388 | Ca-Kabuli-Chr3 | 36242480 | [G/C] | - | URR | - |
| CWSNP2389 | Ca-Kabuli-Chr3 | 36247792 | [T/C] | - | Intergenic | - |
| CWSNP2390 | Ca-Kabuli-Chr3 | 36260865 | [C/A] | - | URR | - |
| CWSNP2391 | Ca-Kabuli-Chr3 | 36282091 | [T/G] | Ca00938 | Non-Synonymous-CDS | Domain of unknown function DUF255 |
| CWSNP2392 | Ca-Kabuli-Chr3 | 36293589 | [A/G] | Ca00939 | Synonymous-CDS | Zinc finger,CW-type |
| CWSNP2393 | Ca-Kabuli-Chr3 | 36293625 | [C/T] | Ca00939 | Synonymous-CDS | Zinc finger,CW-type |
| CWSNP2394 | Ca-Kabuli-Chr3 | 36293637 | [C/T] | Ca00939 | Synonymous-CDS | Zinc finger,CW-type |
| CWSNP2395 | Ca-Kabuli-Chr3 | 36330750 | [A/G] | Ca00942 | Non-Synonymous-CDS | Homeobox |
| CWSNP2396 | Ca-Kabuli-Chr3 | 36330815 | [T/C] | Ca00942 | Synonymous-CDS | Homeobox |
| CWSNP2397 | Ca-Kabuli-Chr3 | 36345158 | [T/C] | Ca00943 | Non-Synonymous-CDS | Zinc finger,C2H2-type |
| CWSNP2398 | Ca-Kabuli-Chr3 | 36361339 | [C/T] | - | Intergenic | - |
| CWSNP2399 | Ca-Kabuli-Chr3 | 36378294 | [G/T] | Ca00946 | Synonymous-CDS | Protein kinase, catalytic domain |
| CWSNP2400 | Ca-Kabuli-Chr3 | 36378299 | [C/G] | Ca00946 | Intron | Protein kinase, catalytic domain |

| SNP IDs | Chromosomes | Physical positions (bp) | SNPs | Gene accession IDs | Sequence components of genes | Putative functions |
|---|---|---|---|---|---|---|
| CWSNP2401 | Ca-Kabuli-Chr3 | 36378358 | [G/A] | Ca00946 | Intron | Protein kinase, catalytic domain |
| CWSNP2402 | Ca-Kabuli-Chr3 | 36387615 | [A/T] | - | DRR | - |
| CWSNP2403 | Ca-Kabuli-Chr3 | 36387732 | [G/A] | - | DRR | - |
| CWSNP2404 | Ca-Kabuli-Chr3 | 36387718 | [C/T] | - | DRR | - |
| CWSNP2405 | Ca-Kabuli-Chr3 | 36396559 | [C/A] | - | URR | - |
| CWSNP2406 | Ca-Kabuli-Chr3 | 36432389 | [A/G] | Ca00957 | Synonymous-CDS | Thiamin pyrophosphokinase, catalytic domain |
| CWSNP2407 | Ca-Kabuli-Chr3 | 36447274 | [G/A] | - | DRR | - |
| CWSNP2408 | Ca-Kabuli-Chr3 | 36447364 | [T/A] | - | DRR | - |
| CWSNP2409 | Ca-Kabuli-Chr3 | 36447520 | [T/A] | - | DRR | - |
| CWSNP2410 | Ca-Kabuli-Chr3 | 36447525 | [C/T] | - | DRR | - |
| CWSNP2411 | Ca-Kabuli-Chr3 | 36488722 | [T/A] | - | Intergenic | - |
| CWSNP2412 | Ca-Kabuli-Chr3 | 36488736 | [T/C] | - | Intergenic | - |
| CWSNP2413 | Ca-Kabuli-Chr3 | 36488853 | [A/G] | - | Intergenic | - |
| CWSNP2414 | Ca-Kabuli-Chr3 | 36503906 | [C/T] | Ca00967 | Non-Synonymous-CDS | Small-subunit processome,Utp14 |
| CWSNP2415 | Ca-Kabuli-Chr3 | 36503912 | [T/C] | Ca00967 | Non-Synonymous-CDS | Small-subunit processome,Utp14 |
| CWSNP2416 | Ca-Kabuli-Chr3 | 36524598 | [G/C] | - | DRR | - |

| SNP IDs | Chromosomes | Physical positions (bp) | SNPs | Gene accession IDs | Sequence components of genes | Putative functions |
|---|---|---|---|---|---|---|
| CWSNP2417 | Ca-Kabuli-Chr3 | 36524635 | [A/G] | - | DRR | - |
| CWSNP2418 | Ca-Kabuli-Chr3 | 36524659 | [T/C] | - | DRR | - |
| CWSNP2419 | Ca-Kabuli-Chr3 | 36531151 | [C/A] | - | URR | - |
| CWSNP2420 | Ca-Kabuli-Chr3 | 36588856 | [C/T] | Ca00978 | Synonymous-CDS | Pectinesterase,catalytic |
| CWSNP2421 | Ca-Kabuli-Chr3 | 36588896 | [C/G] | Ca00978 | Non-Synonymous-CDS | Pectinesterase,catalytic |
| CWSNP2422 | Ca-Kabuli-Chr3 | 36609043 | [G/A] | Ca00980 | Non-Synonymous-CDS | Metal-dependentphosphohydrolase,HDdomain |
| CWSNP2423 | Ca-Kabuli-Chr3 | 36616855 | [T/C] | - | DRR | - |
| CWSNP2424 | Ca-Kabuli-Chr3 | 36644117 | [A/T] | Ca00985 | Synonymous-CDS | TargetSNAREcoiled-coildomain |
| CWSNP2425 | Ca-Kabuli-Chr3 | 36733211 | [A/G] | Ca00997 | Synonymous-CDS | Heat shock protein DnaJ,N-terminal |
| CWSNP2426 | Ca-Kabuli-Chr3 | 36749775 | [T/A] | Ca00998 | Synonymous-CDS | Glycosidehydrolase,family3,N-terminal |
| CWSNP2427 | Ca-Kabuli-Chr3 | 36749826 | [G/A] | Ca00998 | Synonymous-CDS | Glycosidehydrolase,family3,N-terminal |
| CWSNP2428 | Ca-Kabuli-Chr3 | 36749829 | [T/C] | Ca00998 | Non-Synonymous-CDS | Glycosidehydrolase,family3,N-terminal |
| CWSNP2429 | Ca-Kabuli-Chr3 | 36749831 | [T/C] | Ca00998 | Non-Synonymous-CDS | Glycosidehydrolase,family3,N-terminal |
| CWSNP2430 | Ca-Kabuli-Chr3 | 36749871 | [T/C] | Ca00998 | Synonymous-CDS | Glycosidehydrolase,family3,N-terminal |
| CWSNP2431 | Ca-Kabuli-Chr3 | 36749916 | [C/A] | Ca00998 | Non-Synonymous-CDS | Glycosidehydrolase,family3,N-terminal |
| CWSNP2432 | Ca-Kabuli-Chr3 | 36783494 | [T/A] | - | DRR | - |

| SNP IDs | Chromosomes | Physical positions (bp) | SNPs | Gene accession IDs | Sequence components of genes | Putative functions |
|---|---|---|---|---|---|---|
| CWSNP2433 | Ca-Kabuli-Chr3 | 36860134 | [A/G] | - | Intergenic | - |
| CWSNP2434 | Ca-Kabuli-Chr3 | 36883268 | [T/C] | - | URR | - |
| CWSNP2435 | Ca-Kabuli-Chr3 | 36883333 | [A/T] | - | URR | - |
| CWSNP2436 | Ca-Kabuli-Chr3 | 36895362 | [T/C] | Ca01015 | Intron | - |
| CWSNP2437 | Ca-Kabuli-Chr3 | 36938148 | [A/G] | Ca01019 | Intron | RNA recognition motif domain |
| CWSNP2438 | Ca-Kabuli-Chr3 | 36938271 | [G/C] | Ca01019 | Intron | RNA recognition motif domain |
| CWSNP2439 | Ca-Kabuli-Chr3 | 36943522 | [A/G] | - | Intergenic | - |
| CWSNP2440 | Ca-Kabuli-Chr3 | 36991718 | [C/A] | Ca01027 | Intron | Ras small GTPase, Rab type |
| CWSNP2441 | Ca-Kabuli-Chr3 | 37003976 | [C/T] | - | URR | - |
| CWSNP2442 | Ca-Kabuli-Chr3 | 37004026 | [A/G] | - | URR | - |
| CWSNP2443 | Ca-Kabuli-Chr3 | 37067095 | [T/G] | Ca01033 | Synonymous-CDS | Zinc finger,RING-type |
| CWSNP2444 | Ca-Kabuli-Chr3 | 37072312 | [T/C] | Ca01034 | Intron | WD40 repeat |
| CWSNP2445 | Ca-Kabuli-Chr3 | 37090968 | [T/C] | - | DRR | - |
| CWSNP2446 | Ca-Kabuli-Chr3 | 37100897 | [G/T] | Ca01036 | Intron | Transcription factor,SBP-box |
| CWSNP2447 | Ca-Kabuli-Chr3 | 37101339 | [A/T] | Ca01036 | Non-Synonymous-CDS | Transcription factor,SBP-box |
| CWSNP2448 | Ca-Kabuli-Chr3 | 37101433 | [T/G] | Ca01036 | Non-Synonymous-CDS | Transcription factor,SBP-box |

| **SNP IDs** | **Chromosomes** | **Physical positions (bp)** | **SNPs** | **Gene accession IDs** | **Sequence components of genes** | **Putative functions** |
|---|---|---|---|---|---|---|
| CWSNP2449 | Ca-Kabuli-Chr3 | 37101409 | [A/G] | Ca01036 | Synonymous-CDS | Transcription factor,SBP-box |
| CWSNP2450 | Ca-Kabuli-Chr3 | 37108908 | [A/G] | Ca01037 | Intron | Protein kinase, catalytic domain |
| CWSNP2451 | Ca-Kabuli-Chr3 | 37108904 | [A/T] | Ca01037 | Intron | Protein kinase, catalytic domain |
| CWSNP2452 | Ca-Kabuli-Chr3 | 37110070 | [A/G] | Ca01037 | Non-Synonymous-CDS | Protein kinase, catalytic domain |
| CWSNP2453 | Ca-Kabuli-Chr3 | 37110124 | [A/T] | Ca01037 | Intron | Protein kinase, catalytic domain |
| CWSNP2454 | Ca-Kabuli-Chr3 | 37146766 | [C/G] | Ca01044 | Non-Synonymous-CDS | Potassium channel, two pore-domain |
| CWSNP2455 | Ca-Kabuli-Chr3 | 37146840 | [A/G] | Ca01044 | Synonymous-CDS | Potassium channel, two pore-domain |
| CWSNP2456 | Ca-Kabuli-Chr3 | 37154761 | [T/G] | - | Intergenic | - |
| CWSNP2457 | Ca-Kabuli-Chr3 | 37154857 | [T/C] | - | Intergenic | - |
| CWSNP2458 | Ca-Kabuli-Chr3 | 37154938 | [A/C] | - | Intergenic | - |
| CWSNP2459 | Ca-Kabuli-Chr3 | 37155925 | [G/A] | - | Intergenic | - |
| CWSNP2460 | Ca-Kabuli-Chr3 | 37156951 | [A/G] | - | DRR | - |
| CWSNP2461 | Ca-Kabuli-Chr3 | 37160822 | [G/T] | Ca01047 | Synonymous-CDS | Zinc finger,Sec23/Sec24-type |
| CWSNP2462 | Ca-Kabuli-Chr3 | 37160939 | [A/G] | Ca01047 | Synonymous-CDS | Zinc finger,Sec23/Sec24-type |
| CWSNP2463 | Ca-Kabuli-Chr3 | 37303478 | [G/A] | Ca01064 | Synonymous-CDS | WD40 repeat |
| CWSNP2464 | Ca-Kabuli-Chr3 | 37309420 | [A/C] | Ca01064 | Intron | WD40 repeat |

| SNP IDs | Chromosomes | Physical positions (bp) | SNPs | Gene accession IDs | Sequence components of genes | Putative functions |
|---|---|---|---|---|---|---|
| CWSNP2465 | Ca-Kabuli-Chr3 | 37355013 | [A/G] | Ca01069 | Non-Synonymous-CDS | PAS |
| CWSNP2466 | Ca-Kabuli-Chr3 | 37444451 | [C/A] | - | DRR | - |
| CWSNP2467 | Ca-Kabuli-Chr3 | 37599142 | [T/C] | - | DRR | - |
| CWSNP2468 | Ca-Kabuli-Chr3 | 37630502 | [T/C] | - | URR | - |
| CWSNP2469 | Ca-Kabuli-Chr3 | 37635909 | [G/A] | - | DRR | - |
| CWSNP2470 | Ca-Kabuli-Chr3 | 37759329 | [G/A] | Ca01114 | Intron | Armadillo |
| CWSNP2471 | Ca-Kabuli-Chr3 | 37765767 | [A/C] | - | Intergenic | - |
| CWSNP2472 | Ca-Kabuli-Chr3 | 37908317 | [T/C] | - | URR | - |
| CWSNP2473 | Ca-Kabuli-Chr3 | 37908492 | [A/C] | - | URR | - |
| CWSNP2474 | Ca-Kabuli-Chr3 | 37910881 | [A/G] | - | URR | - |
| CWSNP2475 | Ca-Kabuli-Chr3 | 37942807 | [A/G] | - | Intergenic | - |
| CWSNP2476 | Ca-Kabuli-Chr3 | 37942962 | [T/C] | - | Intergenic | - |
| CWSNP2477 | Ca-Kabuli-Chr3 | 37985776 | [T/A] | - | URR | - |
| CWSNP2478 | Ca-Kabuli-Chr3 | 37985977 | [A/G] | - | URR | - |
| CWSNP2479 | Ca-Kabuli-Chr3 | 37985947 | [A/T] | - | URR | - |
| CWSNP2480 | Ca-Kabuli-Chr3 | 38026539 | [A/G] | - | URR | - |

| SNP IDs | Chromosomes | Physical positions (bp) | SNPs | Gene accession IDs | Sequence components of genes | Putative functions |
|---|---|---|---|---|---|---|
| CWSNP2481 | Ca-Kabuli-Chr3 | 38026725 | [G/A] | - | URR | - |
| CWSNP2482 | Ca-Kabuli-Chr3 | 38029095 | [G/C] | Ca01143 | Non-Synonymous-CDS | - |
| CWSNP2483 | Ca-Kabuli-Chr3 | 38033969 | [A/G] | - | URR | - |
| CWSNP2484 | Ca-Kabuli-Chr3 | 38046510 | [T/C] | - | Intergenic | - |
| CWSNP2485 | Ca-Kabuli-Chr3 | 38049996 | [A/G] | - | URR | - |
| CWSNP2486 | Ca-Kabuli-Chr3 | 38059303 | [T/C] | - | URR | - |
| CWSNP2487 | Ca-Kabuli-Chr3 | 38059311 | [T/A] | - | URR | - |
| CWSNP2488 | Ca-Kabuli-Chr3 | 38060814 | [A/G] | - | URR | - |
| CWSNP2489 | Ca-Kabuli-Chr3 | 38060755 | [T/C] | - | URR | - |
| CWSNP2490 | Ca-Kabuli-Chr3 | 38060742 | [C/T] | - | URR | - |
| CWSNP2491 | Ca-Kabuli-Chr3 | 38074259 | [C/A] | - | URR | - |
| CWSNP2492 | Ca-Kabuli-Chr3 | 38103375 | [T/C] | Ca01154 | Intron | PeptidaseS1/S6,chymotrypsin/Hap |
| CWSNP2493 | Ca-Kabuli-Chr3 | 38103378 | [C/T] | Ca01154 | Intron | PeptidaseS1/S6,chymotrypsin/Hap |
| CWSNP2494 | Ca-Kabuli-Chr3 | 38103385 | [C/T] | Ca01154 | Intron | PeptidaseS1/S6,chymotrypsin/Hap |
| CWSNP2495 | Ca-Kabuli-Chr3 | 38103524 | [C/T] | Ca01154 | Synonymous-CDS | PeptidaseS1/S6,chymotrypsin/Hap |
| CWSNP2496 | Ca-Kabuli-Chr3 | 38112801 | [C/T] | - | DRR | - |

| SNP IDs | Chromosomes | Physical positions (bp) | SNPs | Gene accession IDs | Sequence components of genes | Putative functions |
|---|---|---|---|---|---|---|
| CWSNP2497 | Ca-Kabuli-Chr3 | 38112802 | [T/C] | - | DRR | - |
| CWSNP2498 | Ca-Kabuli-Chr3 | 38112971 | [A/G] | - | DRR | - |
| CWSNP2499 | Ca-Kabuli-Chr3 | 38117169 | [T/C] | Ca01156 | Intron | Acyl-CoA-binding protein,ACBP |
| CWSNP2500 | Ca-Kabuli-Chr3 | 38130410 | [A/G] | - | Intergenic | - |
| CWSNP2501 | Ca-Kabuli-Chr3 | 38145115 | [C/T] | Ca01158 | Synonymous-CDS | Cystathioninebeta-synthase,core |
| CWSNP2502 | Ca-Kabuli-Chr3 | 38202223 | [C/T] | Ca01163 | Intron | Creatinase |
| CWSNP2503 | Ca-Kabuli-Chr3 | 38220910 | [T/C] | - | URR | - |
| CWSNP2504 | Ca-Kabuli-Chr3 | 38220958 | [A/G] | - | URR | - |
| CWSNP2505 | Ca-Kabuli-Chr3 | 38222750 | [A/C] | - | DRR | - |
| CWSNP2506 | Ca-Kabuli-Chr3 | 38222747 | [G/A] | - | DRR | - |
| CWSNP2507 | Ca-Kabuli-Chr3 | 38222733 | [C/G] | - | DRR | - |
| CWSNP2508 | Ca-Kabuli-Chr3 | 38228118 | [G/T] | - | URR | - |
| CWSNP2509 | Ca-Kabuli-Chr3 | 38386392 | [T/C] | - | DRR | - |
| CWSNP2510 | Ca-Kabuli-Chr3 | 38452060 | [A/C] | Ca01198 | Non-Synonymous-CDS | Heat shock protein DnaJ,N-terminal |
| CWSNP2511 | Ca-Kabuli-Chr3 | 38539721 | [T/G] | Ca01207 | Non-Synonymous-CDS | Alpha/beta hydrolase fold-1 |
| CWSNP2512 | Ca-Kabuli-Chr3 | 38551142 | [C/T] | Ca01208 | Intron | WD40 repeat |

| SNP IDs | Chromosomes | Physical positions (bp) | SNPs | Gene accession IDs | Sequence components of genes | Putative functions |
|---|---|---|---|---|---|---|
| CWSNP2513 | Ca-Kabuli-Chr3 | 38672863 | [C/A] | - | Intergenic | - |
| CWSNP2514 | Ca-Kabuli-Chr3 | 38731466 | [T/G] | Ca01231 | Non-Synonymous-CDS | Ammonium transporter |
| CWSNP2515 | Ca-Kabuli-Chr3 | 38764390 | [A/C] | - | Intergenic | - |
| CWSNP2516 | Ca-Kabuli-Chr3 | 38766174 | [A/G] | - | DRR | - |
| CWSNP2517 | Ca-Kabuli-Chr3 | 38766311 | [A/C] | - | DRR | - |
| CWSNP2518 | Ca-Kabuli-Chr3 | 38805831 | [A/C] | Ca01240 | Intron | Ankyrin repeat |
| CWSNP2519 | Ca-Kabuli-Chr3 | 38821990 | [A/G] | - | DRR | - |
| CWSNP2520 | Ca-Kabuli-Chr3 | 38831533 | [T/C] | - | Intergenic | - |
| CWSNP2521 | Ca-Kabuli-Chr3 | 38848412 | [C/G] | Ca01245 | Synonymous-CDS | K Homology |
| CWSNP2522 | Ca-Kabuli-Chr3 | 38913449 | [T/C] | - | DRR | - |
| CWSNP2523 | Ca-Kabuli-Chr3 | 38923321 | [C/T] | Ca01252 | Synonymous-CDS | Glycosyltransferase,family8 |
| CWSNP2524 | Ca-Kabuli-Chr3 | 38965610 | [T/A] | Ca01258 | Non-Synonymous-CDS | SANT domain, DNA binding |
| CWSNP2525 | Ca-Kabuli-Chr3 | 38982481 | [A/G] | Ca01260 | Intron | - |
| CWSNP2526 | Ca-Kabuli-Chr3 | 38987916 | [A/C] | - | DRR | - |
| CWSNP2527 | Ca-Kabuli-Chr3 | 39034246 | [C/T] | Ca01265 | Non-Synonymous-CDS | - |
| CWSNP2528 | Ca-Kabuli-Chr3 | 39036130 | [T/C] | Ca01265 | Intron | - |

| SNP IDs | Chromosomes | Physical positions (bp) | SNPs | Gene accession IDs | Sequence components of genes | Putative functions |
|---|---|---|---|---|---|---|
| CWSNP2529 | Ca-Kabuli-Chr3 | 39055529 | [C/T] | Ca01268 | Intron | EF-Hand1,calcium-binding site |
| CWSNP2530 | Ca-Kabuli-Chr3 | 39081040 | [A/G] | - | Intergenic | - |
| CWSNP2531 | Ca-Kabuli-Chr3 | 39084916 | [T/A] | Ca01271 | Non-Synonymous-CDS | Domain of unknown function DUF296 |
| CWSNP2532 | Ca-Kabuli-Chr3 | 39084979 | [A/C] | Ca01271 | Synonymous-CDS | Domain of unknown function DUF296 |
| CWSNP2533 | Ca-Kabuli-Chr3 | 39093242 | [G/A] | - | Intergenic | - |
| CWSNP2534 | Ca-Kabuli-Chr3 | 39093243 | [C/A] | - | Intergenic | - |
| CWSNP2535 | Ca-Kabuli-Chr3 | 39093245 | [T/A] | - | Intergenic | - |
| CWSNP2536 | Ca-Kabuli-Chr3 | 39093215 | [G/A] | - | Intergenic | - |
| CWSNP2537 | Ca-Kabuli-Chr3 | 39115274 | [A/C] | Ca01274 | Intron | HEAT |
| CWSNP2538 | Ca-Kabuli-Chr3 | 39115986 | [C/G] | Ca01274 | Intron | HEAT |
| CWSNP2539 | Ca-Kabuli-Chr3 | 39115979 | [A/G] | Ca01274 | Intron | HEAT |
| CWSNP2540 | Ca-Kabuli-Chr3 | 39115972 | [T/A] | Ca01274 | Intron | HEAT |
| CWSNP2541 | Ca-Kabuli-Chr3 | 39115970 | [T/G] | Ca01274 | Intron | HEAT |
| CWSNP2542 | Ca-Kabuli-Chr3 | 39118510 | [C/T] | Ca01274 | Intron | HEAT |
| CWSNP2543 | Ca-Kabuli-Chr3 | 39207112 | [A/C] | Ca01277 | Synonymous-CDS | CytoplasmicFMR1-interacting |
| CWSNP2544 | Ca-Kabuli-Chr3 | 39215327 | [C/T] | Ca01278 | Non-Synonymous-CDS | Armadillo |

| SNP IDs | Chromosomes | Physical positions (bp) | SNPs | Gene accession IDs | Sequence components of genes | Putative functions |
|---|---|---|---|---|---|---|
| CWSNP2545 | Ca-Kabuli-Chr3 | 39215476 | [G/A] | Ca01278 | Synonymous-CDS | Armadillo |
| CWSNP2546 | Ca-Kabuli-Chr3 | 39254527 | [T/G] | - | Intergenic | - |
| CWSNP2547 | Ca-Kabuli-Chr3 | 39255721 | [A/C] | - | Intergenic | - |
| CWSNP2548 | Ca-Kabuli-Chr3 | 39255715 | [G/A] | - | Intergenic | - |
| CWSNP2549 | Ca-Kabuli-Chr3 | 39255713 | [C/A] | - | Intergenic | - |
| CWSNP2550 | Ca-Kabuli-Chr3 | 39255709 | [A/T] | - | Intergenic | - |
| CWSNP2551 | Ca-Kabuli-Chr3 | 39257226 | [A/G] | Ca01285 | Intron | - |
| CWSNP2552 | Ca-Kabuli-Chr3 | 39405252 | [C/A] | Ca01306 | Non-Synonymous-CDS | Lipase,class3 |
| CWSNP2553 | Ca-Kabuli-Chr3 | 39504242 | [A/G] | - | Intergenic | - |
| CWSNP2554 | Ca-Kabuli-Chr3 | 39592340 | [C/T] | - | URR | - |
| CWSNP2555 | Ca-Kabuli-Chr3 | 39612466 | [T/C] | Ca01338 | Intron | Profilin/allergen |
| CWSNP2556 | Ca-Kabuli-Chr3 | 39612458 | [C/A] | Ca01338 | Intron | Profilin/allergen |
| CWSNP2557 | Ca-Kabuli-Chr3 | 39612447 | [C/A] | Ca01338 | Intron | Profilin/allergen |
| CWSNP2558 | Ca-Kabuli-Chr3 | 39612568 | [A/T] | Ca01338 | Non-Synonymous-CDS | Profilin/allergen |
| CWSNP2559 | Ca-Kabuli-Chr3 | 39628916 | [G/A] | - | DRR | - |
| CWSNP2560 | Ca-Kabuli-Chr3 | 39722314 | [C/T] | Ca01355 | Non-Synonymous-CDS | Dehydrogenase,E1component |

| SNP IDs | Chromosomes | Physical positions (bp) | SNPs | Gene accession IDs | Sequence components of genes | Putative functions |
|---|---|---|---|---|---|---|
| CWSNP2561 | Ca-Kabuli-Chr3 | 39787513 | [A/C] | Ca01365 | Synonymous-CDS | - |
| CWSNP2562 | Ca-Kabuli-Chr3 | 39801213 | [A/G] | Ca01367 | Intron | RibonucleaseIII |
| CWSNP2563 | Ca-Kabuli-Chr3 | 39817122 | [G/A] | Ca01368 | Synonymous-CDS | Transcriptional factor B3 |
| CWSNP2564 | Ca-Kabuli-Chr3 | 39839763 | [T/G] | Ca01370 | Intron | ATPase ,P-type, H+transporting proton pump |
| CWSNP2565 | Ca-Kabuli-Chr3 | 39890991 | [A/T] | Ca01375 | Intron | Exportin-1/Importin-beta-like |
| CWSNP2566 | Ca-Kabuli-Chr3 | 39908983 | [C/G] | - | Intergenic | - |
| CWSNP2567 | Ca-Kabuli-Chr3 | 39909824 | [C/G] | - | Intergenic | - |
| CWSNP2568 | Ca-Kabuli-Chr3 | 39914344 | [T/G] | Ca01376 | Intron | RNA recognition motif domain |
| CWSNP2569 | Ca-Kabuli-Chr4 | 20297 | [A/G] | Ca27693 | Synonymous-CDS | Basic-leucine zipper (bZIP) Transcription factor |
| CWSNP2570 | Ca-Kabuli-Chr4 | 20495 | [G/C] | Ca27693 | Intron | Basic-leucine zipper (bZIP) Transcription factor |
| CWSNP2571 | Ca-Kabuli-Chr4 | 167229 | [T/G] | Ca07652 | Synonymous-CDS | LNS2,Lipin/Ned1/Smp2 |
| CWSNP2572 | Ca-Kabuli-Chr4 | 338721 | [C/T] | - | Intergenic | - |
| CWSNP2573 | Ca-Kabuli-Chr4 | 349309 | [C/T] | Ca07667 | Non-Synonymous-CDS | Multiantimicrobial extrusion protein |
| CWSNP2574 | Ca-Kabuli-Chr4 | 369258 | [C/T] | Ca07669 | Non-Synonymous-CDS | Multiantimicrobial extrusion protein |
| CWSNP2575 | Ca-Kabuli-Chr4 | 394661 | [C/G] | Ca07671 | Non-Synonymous-CDS | Inorganic pyrophosphatase |
| CWSNP2576 | Ca-Kabuli-Chr4 | 412017 | [C/A] | Ca07673 | Non-Synonymous-CDS | Arf GTPase activating protein |

| SNP IDs | Chromosomes | Physical positions (bp) | SNPs | Gene accession IDs | Sequence components of genes | Putative functions |
|---|---|---|---|---|---|---|
| CWSNP2577 | Ca-Kabuli-Chr4 | 455361 | [A/G] | Ca07679 | Intron | ArgininebiosynthesisproteinArgJ |
| CWSNP2578 | Ca-Kabuli-Chr4 | 480138 | [C/A] | Ca07682 | Non-Synonymous-CDS | - |
| CWSNP2579 | Ca-Kabuli-Chr4 | 586703 | [T/C] | - | DRR | - |
| CWSNP2580 | Ca-Kabuli-Chr4 | 589191 | [A/G] | - | Intergenic | - |
| CWSNP2581 | Ca-Kabuli-Chr4 | 589204 | [G/A] | - | Intergenic | - |
| CWSNP2582 | Ca-Kabuli-Chr4 | 589220 | [A/C] | - | Intergenic | - |
| CWSNP2583 | Ca-Kabuli-Chr4 | 595982 | [C/G] | Ca07691 | Synonymous-CDS | Helix-loop-helix DNA-binding domain |
| CWSNP2584 | Ca-Kabuli-Chr4 | 646567 | [C/T] | - | Intergenic | - |
| CWSNP2585 | Ca-Kabuli-Chr4 | 660034 | [A/C] | - | URR | - |
| CWSNP2586 | Ca-Kabuli-Chr4 | 660029 | [T/G] | - | URR | - |
| CWSNP2587 | Ca-Kabuli-Chr4 | 1190184 | [A/C] | Ca07749 | Intron | Ribosomal proteinL21e |
| CWSNP2588 | Ca-Kabuli-Chr4 | 1526270 | [T/C] | - | URR | - |
| CWSNP2589 | Ca-Kabuli-Chr4 | 1687346 | [A/G] | - | Intergenic | - |
| CWSNP2590 | Ca-Kabuli-Chr4 | 1691555 | [T/G] | - | Intergenic | - |
| CWSNP2591 | Ca-Kabuli-Chr4 | 1708370 | [C/T] | Ca07797 | Synonymous-CDS | BEACH domain |
| CWSNP2592 | Ca-Kabuli-Chr4 | 1711864 | [A/C] | Ca07797 | Synonymous-CDS | BEACH domain |

| SNP IDs | Chromosomes | Physical positions (bp) | SNPs | Gene accession IDs | Sequence components of genes | Putative functions |
|---|---|---|---|---|---|---|
| CWSNP2593 | Ca-Kabuli-Chr4 | 1711865 | [G/A] | Ca07797 | Synonymous-CDS | BEACH domain |
| CWSNP2594 | Ca-Kabuli-Chr4 | 1720642 | [A/G] | Ca07797 | Intron | BEACH domain |
| CWSNP2595 | Ca-Kabuli-Chr4 | 1755066 | [G/C] | - | Intergenic | - |
| CWSNP2596 | Ca-Kabuli-Chr4 | 1773886 | [C/G] | - | Intergenic | - |
| CWSNP2597 | Ca-Kabuli-Chr4 | 1773939 | [C/T] | - | Intergenic | - |
| CWSNP2598 | Ca-Kabuli-Chr4 | 1773951 | [C/G] | - | Intergenic | - |
| CWSNP2599 | Ca-Kabuli-Chr4 | 1785705 | [G/C] | Ca07801 | Intron | Protein kinase, catalytic domain |
| CWSNP2600 | Ca-Kabuli-Chr4 | 1833725 | [C/T] | - | DRR | - |
| CWSNP2601 | Ca-Kabuli-Chr4 | 1867921 | [G/A] | Ca07807 | Intron | Sulphate transporter/antisigma-facto rantagonist STAS |
| CWSNP2602 | Ca-Kabuli-Chr4 | 1869577 | [C/T] | - | Intergenic | - |
| CWSNP2603 | Ca-Kabuli-Chr4 | 1870333 | [A/G] | - | DRR | - |
| CWSNP2604 | Ca-Kabuli-Chr4 | 1870317 | [T/C] | - | DRR | - |
| CWSNP2605 | Ca-Kabuli-Chr4 | 1870666 | [G/C] | - | DRR | - |
| CWSNP2606 | Ca-Kabuli-Chr4 | 1870806 | [C/A] | - | DRR | - |
| CWSNP2607 | Ca-Kabuli-Chr4 | 1870802 | [C/T] | - | DRR | - |
| CWSNP2608 | Ca-Kabuli-Chr4 | 1873733 | [T/C] | Ca07808 | Non-Synonymous-CDS | KIP1-like |

| SNP IDs | Chromosomes | Physical positions (bp) | SNPs | Gene accession IDs | Sequence components of genes | Putative functions |
|---|---|---|---|---|---|---|
| CWSNP2609 | Ca-Kabuli-Chr4 | 1941089 | [C/T] | - | URR | - |
| CWSNP2610 | Ca-Kabuli-Chr4 | 2034429 | [C/A] | Ca07822 | Intron | RNA-processing protein,HAT helix |
| CWSNP2611 | Ca-Kabuli-Chr4 | 2034398 | [C/T] | Ca07822 | Intron | RNA-processing protein,HAT helix |
| CWSNP2612 | Ca-Kabuli-Chr4 | 2034396 | [A/G] | Ca07822 | Intron | RNA-processing protein,HAT helix |
| CWSNP2613 | Ca-Kabuli-Chr4 | 2034739 | [A/C] | Ca07822 | Intron | RNA-processing protein,HAT helix |
| CWSNP2614 | Ca-Kabuli-Chr4 | 2035797 | [G/A] | Ca07822 | Synonymous-CDS | RNA-processing protein,HAT helix |
| CWSNP2615 | Ca-Kabuli-Chr4 | 2035801 | [G/A] | Ca07822 | Synonymous-CDS | RNA-processing protein,HAT helix |
| CWSNP2616 | Ca-Kabuli-Chr4 | 2035841 | [C/T] | - | Intergenic | - |
| CWSNP2617 | Ca-Kabuli-Chr4 | 2035894 | [C/A] | - | Intergenic | - |
| CWSNP2618 | Ca-Kabuli-Chr4 | 2125774 | [G/A] | Ca07833 | Non-Synonymous-CDS | Ras GTPase |
| CWSNP2619 | Ca-Kabuli-Chr4 | 2213710 | [G/A] | - | DRR | - |
| CWSNP2620 | Ca-Kabuli-Chr4 | 2361088 | [T/A] | Ca07858 | Intron | ABC transporter-like |
| CWSNP2621 | Ca-Kabuli-Chr4 | 2380206 | [A/G] | - | Intergenic | - |
| CWSNP2622 | Ca-Kabuli-Chr4 | 2393033 | [T/G] | Ca07861 | Intron | DNA helicase,UvrD/REP type |
| CWSNP2623 | Ca-Kabuli-Chr4 | 2411925 | [G/T] | Ca07861 | Intron | DNA helicase,UvrD/REP type |
| CWSNP2624 | Ca-Kabuli-Chr4 | 2609945 | [C/T] | - | Intergenic | - |

| SNP IDs | Chromosomes | Physical positions (bp) | SNPs | Gene accession IDs | Sequence components of genes | Putative functions |
|---|---|---|---|---|---|---|
| CWSNP2625 | Ca-Kabuli-Chr4 | 2676174 | [C/T] | Ca12071 | Synonymous-CDS | Signal transduction response regulator,receiver domain |
| CWSNP2626 | Ca-Kabuli-Chr4 | 2886775 | [T/G] | Ca12085 | Synonymous-CDS | General substrate transporter |
| CWSNP2627 | Ca-Kabuli-Chr4 | 3037899 | [A/C] | - | URR | - |
| CWSNP2628 | Ca-Kabuli-Chr4 | 3037986 | [T/A] | - | URR | - |
| CWSNP2629 | Ca-Kabuli-Chr4 | 3037929 | [T/G] | - | URR | - |
| CWSNP2630 | Ca-Kabuli-Chr4 | 3038022 | [C/T] | - | URR | - |
| CWSNP2631 | Ca-Kabuli-Chr4 | 3046920 | [A/G] | Ca12103 | Intron | TetratricopeptideTPR-1 |
| CWSNP2632 | Ca-Kabuli-Chr4 | 3158321 | [A/G] | - | URR | - |
| CWSNP2633 | Ca-Kabuli-Chr4 | 3158323 | [T/G] | - | URR | - |
| CWSNP2634 | Ca-Kabuli-Chr4 | 3158328 | [G/T] | - | URR | - |
| CWSNP2635 | Ca-Kabuli-Chr4 | 3158331 | [A/T] | - | URR | - |
| CWSNP2636 | Ca-Kabuli-Chr4 | 3158426 | [G/A] | - | URR | - |
| CWSNP2637 | Ca-Kabuli-Chr4 | 3158504 | [A/T] | - | URR | - |
| CWSNP2638 | Ca-Kabuli-Chr4 | 3158549 | [A/G] | - | URR | - |
| CWSNP2639 | Ca-Kabuli-Chr4 | 3164083 | [G/A] | - | Intergenic | - |
| CWSNP2640 | Ca-Kabuli-Chr4 | 3166657 | [C/T] | Ca12117 | Intron | - |

| SNP IDs | Chromosomes | Physical positions (bp) | SNPs | Gene accession IDs | Sequence components of genes | Putative functions |
|---|---|---|---|---|---|---|
| CWSNP2641 | Ca-Kabuli-Chr4 | 3166663 | [C/T] | Ca12117 | Intron | - |
| CWSNP2642 | Ca-Kabuli-Chr4 | 3166674 | [G/T] | Ca12117 | Intron | - |
| CWSNP2643 | Ca-Kabuli-Chr4 | 3171162 | [G/A] | Ca12117 | Synonymous-CDS | - |
| CWSNP2644 | Ca-Kabuli-Chr4 | 3171385 | [A/G] | Ca12117 | Intron | - |
| CWSNP2645 | Ca-Kabuli-Chr4 | 3171428 | [T/C] | Ca12117 | Intron | - |
| CWSNP2646 | Ca-Kabuli-Chr4 | 3171551 | [C/T] | Ca12117 | Intron | - |
| CWSNP2647 | Ca-Kabuli-Chr4 | 3264351 | [T/C] | Ca12126 | Synonymous-CDS | Domain of unknown function DUF640 |
| CWSNP2648 | Ca-Kabuli-Chr4 | 3280217 | [A/G] | - | URR | - |
| CWSNP2649 | Ca-Kabuli-Chr4 | 3281050 | [T/G] | - | URR | - |
| CWSNP2650 | Ca-Kabuli-Chr4 | 3290298 | [T/G] | - | DRR | - |
| CWSNP2651 | Ca-Kabuli-Chr4 | 3356252 | [C/G] | - | Intergenic | - |
| CWSNP2652 | Ca-Kabuli-Chr4 | 3356242 | [C/G] | - | Intergenic | - |
| CWSNP2653 | Ca-Kabuli-Chr4 | 3400828 | [G/C] | Ca12141 | Intron | Glycosidehydrolase,family47 |
| CWSNP2654 | Ca-Kabuli-Chr4 | 3427396 | [A/G] | Ca12144 | Intron | Castor/Polluxionchannel |
| CWSNP2655 | Ca-Kabuli-Chr4 | 3429035 | [G/A] | Ca12144 | Intron | Castor/Polluxionchannel |
| CWSNP2656 | Ca-Kabuli-Chr4 | 3429059 | [C/A] | Ca12144 | Intron | Castor/Polluxionchannel |

| SNP IDs | Chromosomes | Physical positions (bp) | SNPs | Gene accession IDs | Sequence components of genes | Putative functions |
|---|---|---|---|---|---|---|
| CWSNP2657 | Ca-Kabuli-Chr4 | 3455047 | [G/A] | Ca12146 | Non-Synonymous-CDS | Armadillo |
| CWSNP2658 | Ca-Kabuli-Chr4 | 3689840 | [T/C] | Ca12165 | Intron | ATPase,AAA+type,core |
| CWSNP2659 | Ca-Kabuli-Chr4 | 3741522 | [T/G] | - | URR | - |
| CWSNP2660 | Ca-Kabuli-Chr4 | 3741539 | [T/G] | - | URR | - |
| CWSNP2661 | Ca-Kabuli-Chr4 | 3762559 | [T/C] | - | Intergenic | - |
| CWSNP2662 | Ca-Kabuli-Chr4 | 3762561 | [C/A] | - | Intergenic | - |
| CWSNP2663 | Ca-Kabuli-Chr4 | 3762585 | [G/T] | - | Intergenic | - |
| CWSNP2664 | Ca-Kabuli-Chr4 | 3794764 | [G/A] | - | Intergenic | - |
| CWSNP2665 | Ca-Kabuli-Chr4 | 3794885 | [C/A] | - | Intergenic | - |
| CWSNP2666 | Ca-Kabuli-Chr4 | 3888185 | [A/G] | - | Intergenic | - |
| CWSNP2667 | Ca-Kabuli-Chr4 | 3888328 | [C/T] | - | Intergenic | - |
| CWSNP2668 | Ca-Kabuli-Chr4 | 3888529 | [T/G] | - | Intergenic | - |
| CWSNP2669 | Ca-Kabuli-Chr4 | 4027829 | [A/T] | - | Intergenic | - |
| CWSNP2670 | Ca-Kabuli-Chr4 | 4027872 | [A/C] | - | Intergenic | - |
| CWSNP2671 | Ca-Kabuli-Chr4 | 4097989 | [G/A] | Ca03846 | Synonymous-CDS | UBX |
| CWSNP2672 | Ca-Kabuli-Chr4 | 4097935 | [T/C] | Ca03846 | Intron | UBX |

| SNP IDs | Chromosomes | Physical positions (bp) | SNPs | Gene accession IDs | Sequence components of genes | Putative functions |
|---|---|---|---|---|---|---|
| CWSNP2673 | Ca-Kabuli-Chr4 | 4148061 | [T/C] | Ca03841 | Synonymous-CDS | Plant specific mitochondrial import receptor subunit TOM20 |
| CWSNP2674 | Ca-Kabuli-Chr4 | 4148250 | [A/G] | Ca03841 | Intron | Plant specific mitochondrial import receptor subunit TOM20 |
| CWSNP2675 | Ca-Kabuli-Chr4 | 4154507 | [G/T] | Ca03840 | Intron | - |
| CWSNP2676 | Ca-Kabuli-Chr4 | 4305891 | [A/C] | Ca03829 | Synonymous-CDS | Inositol polyphosphate-related phosphatase |
| CWSNP2677 | Ca-Kabuli-Chr4 | 4410351 | [T/C] | Ca03820 | Synonymous-CDS | Single-strandednucleicacidbindingR3H |
| CWSNP2678 | Ca-Kabuli-Chr4 | 4426659 | [C/T] | - | DRR | - |
| CWSNP2679 | Ca-Kabuli-Chr4 | 4452018 | [T/A] | - | Intergenic | - |
| CWSNP2680 | Ca-Kabuli-Chr4 | 4452380 | [C/G] | - | Intergenic | - |
| CWSNP2681 | Ca-Kabuli-Chr4 | 4454305 | [A/C] | - | Intergenic | - |
| CWSNP2682 | Ca-Kabuli-Chr4 | 4454464 | [A/G] | - | DRR | - |
| CWSNP2683 | Ca-Kabuli-Chr4 | 4461562 | [G/C] | Ca03811 | Intron | TRM13/UPF0224family,U11-48K-likeCHHCZinc fingerdomain |
| CWSNP2684 | Ca-Kabuli-Chr4 | 4468394 | [C/T] | Ca03810 | Intron | TetratricopeptideTPR-1 |
| CWSNP2685 | Ca-Kabuli-Chr4 | 4536310 | [A/G] | - | DRR | - |
| CWSNP2686 | Ca-Kabuli-Chr4 | 4536286 | [G/C] | - | DRR | - |
| CWSNP2687 | Ca-Kabuli-Chr4 | 4578836 | [C/T] | - | DRR | - |
| CWSNP2688 | Ca-Kabuli-Chr4 | 4605340 | [A/C] | - | DRR | - |

| SNP IDs | Chromosomes | Physical positions (bp) | SNPs | Gene accession IDs | Sequence components of genes | Putative functions |
|---|---|---|---|---|---|---|
| CWSNP2689 | Ca-Kabuli-Chr4 | 4628243 | [G/A] | - | Intergenic | - |
| CWSNP2690 | Ca-Kabuli-Chr4 | 4630407 | [G/A] | - | URR | - |
| CWSNP2691 | Ca-Kabuli-Chr4 | 4633913 | [T/G] | - | Intergenic | - |
| CWSNP2692 | Ca-Kabuli-Chr4 | 4634542 | [A/T] | - | URR | - |
| CWSNP2693 | Ca-Kabuli-Chr4 | 4670913 | [A/G] | Ca03789 | Intron | EF-Hand1,calcium-binding site |
| CWSNP2694 | Ca-Kabuli-Chr4 | 4675182 | [T/G] | - | DRR | - |
| CWSNP2695 | Ca-Kabuli-Chr4 | 4675177 | [T/A] | - | DRR | - |
| CWSNP2696 | Ca-Kabuli-Chr4 | 4675176 | [T/G] | - | DRR | - |
| CWSNP2697 | Ca-Kabuli-Chr4 | 4675172 | [T/A] | - | DRR | - |
| CWSNP2698 | Ca-Kabuli-Chr4 | 4675171 | [T/A] | - | DRR | - |
| CWSNP2699 | Ca-Kabuli-Chr4 | 4675166 | [A/T] | - | DRR | - |
| CWSNP2700 | Ca-Kabuli-Chr4 | 4679236 | [A/G] | Ca03788 | Synonymous-CDS | Pentatrico peptide repeat |
| CWSNP2701 | Ca-Kabuli-Chr4 | 4799122 | [C/T] | Ca03778 | Non-Synonymous-CDS | SNF2-related |
| CWSNP2702 | Ca-Kabuli-Chr4 | 4799116 | [C/T] | Ca03778 | Non-Synonymous-CDS | SNF2-related |
| CWSNP2703 | Ca-Kabuli-Chr4 | 4907987 | [G/A] | - | Intergenic | - |
| CWSNP2704 | Ca-Kabuli-Chr4 | 4911083 | [T/C] | Ca03767 | Intron | Phospho ribosylaminoimidazole carboxylase,core |

| SNP IDs | Chromosomes | Physical positions (bp) | SNPs | Gene accession IDs | Sequence components of genes | Putative functions |
|---|---|---|---|---|---|---|
| CWSNP2705 | Ca-Kabuli-Chr4 | 4911130 | [T/C] | Ca03767 | Intron | Phospho ribosylaminoimidazole carboxylase,core |
| CWSNP2706 | Ca-Kabuli-Chr4 | 4911209 | [A/G] | Ca03767 | Synonymous-CDS | Phospho ribosylaminoimidazole carboxylase,core |
| CWSNP2707 | Ca-Kabuli-Chr4 | 4911217 | [A/C] | Ca03767 | Synonymous-CDS | Phospho ribosylaminoimidazole carboxylase,core |
| CWSNP2708 | Ca-Kabuli-Chr4 | 4920706 | [T/C] | Ca03766 | Non-Synonymous-CDS | Zinc finger,PMZ-type |
| CWSNP2709 | Ca-Kabuli-Chr4 | 4920712 | [A/T] | Ca03766 | Non-Synonymous-CDS | Zinc finger,PMZ-type |
| CWSNP2710 | Ca-Kabuli-Chr4 | 4920719 | [A/G] | Ca03766 | Non-Synonymous-CDS | Zinc finger,PMZ-type |
| CWSNP2711 | Ca-Kabuli-Chr4 | 4928764 | [T/C] | - | Intergenic | - |
| CWSNP2712 | Ca-Kabuli-Chr4 | 4928890 | [A/G] | - | Intergenic | - |
| CWSNP2713 | Ca-Kabuli-Chr4 | 4953629 | [C/A] | - | DRR | - |
| CWSNP2714 | Ca-Kabuli-Chr4 | 5038300 | [G/C] | - | DRR | - |
| CWSNP2715 | Ca-Kabuli-Chr4 | 5186401 | [A/G] | Ca03734 | Non-Synonymous-CDS | Pyridoxalphosphate-dependentenzyme,betasubunit |
| CWSNP2716 | Ca-Kabuli-Chr4 | 5222851 | [C/T] | Ca03733 | Intron | Domain of unknown function DUF1981,SEC7associated |
| CWSNP2717 | Ca-Kabuli-Chr4 | 5223100 | [G/A] | Ca03733 | Intron | Domain of unknown function DUF1981,SEC7associated |
| CWSNP2718 | Ca-Kabuli-Chr4 | 5232364 | [T/C] | - | DRR | - |
| CWSNP2719 | Ca-Kabuli-Chr4 | 5263199 | [A/G] | - | DRR | - |
| CWSNP2720 | Ca-Kabuli-Chr4 | 5268995 | [A/C] | - | URR | - |

| SNP IDs | Chromosomes | Physical positions (bp) | SNPs | Gene accession IDs | Sequence components of genes | Putative functions |
|---|---|---|---|---|---|---|
| CWSNP2721 | Ca-Kabuli-Chr4 | 5282131 | [T/C] | - | Intergenic | - |
| CWSNP2722 | Ca-Kabuli-Chr4 | 5390317 | [G/A] | - | DRR | - |
| CWSNP2723 | Ca-Kabuli-Chr4 | 5473174 | [C/T] | - | DRR | - |
| CWSNP2724 | Ca-Kabuli-Chr4 | 5484259 | [G/T] | - | Intergenic | - |
| CWSNP2725 | Ca-Kabuli-Chr4 | 5484257 | [G/C] | - | Intergenic | - |
| CWSNP2726 | Ca-Kabuli-Chr4 | 5484238 | [G/A] | - | Intergenic | - |
| CWSNP2727 | Ca-Kabuli-Chr4 | 5484236 | [C/T] | - | Intergenic | - |
| CWSNP2728 | Ca-Kabuli-Chr4 | 5484220 | [A/G] | - | Intergenic | - |
| CWSNP2729 | Ca-Kabuli-Chr4 | 5485052 | [G/T] | - | Intergenic | - |
| CWSNP2730 | Ca-Kabuli-Chr4 | 5485050 | [G/C] | - | Intergenic | - |
| CWSNP2731 | Ca-Kabuli-Chr4 | 5485031 | [G/A] | - | Intergenic | - |
| CWSNP2732 | Ca-Kabuli-Chr4 | 5485029 | [C/T] | - | Intergenic | - |
| CWSNP2733 | Ca-Kabuli-Chr4 | 5485013 | [A/G] | - | Intergenic | - |
| CWSNP2734 | Ca-Kabuli-Chr4 | 5528421 | [T/G] | Ca03702 | Synonymous-CDS | Zinc finger,RING-type |
| CWSNP2735 | Ca-Kabuli-Chr4 | 5701873 | [A/C] | - | Intergenic | - |
| CWSNP2736 | Ca-Kabuli-Chr4 | 5701900 | [G/T] | - | Intergenic | - |

| SNP IDs | Chromosomes | Physical positions (bp) | SNPs | Gene accession IDs | Sequence components of genes | Putative functions |
|---|---|---|---|---|---|---|
| CWSNP2737 | Ca-Kabuli-Chr4 | 5701902 | [C/T] | - | Intergenic | - |
| CWSNP2738 | Ca-Kabuli-Chr4 | 5701907 | [G/T] | - | Intergenic | - |
| CWSNP2739 | Ca-Kabuli-Chr4 | 5779698 | [A/G] | - | Intergenic | - |
| CWSNP2740 | Ca-Kabuli-Chr4 | 5787727 | [G/A] | - | URR | - |
| CWSNP2741 | Ca-Kabuli-Chr4 | 5787722 | [G/A] | - | URR | - |
| CWSNP2742 | Ca-Kabuli-Chr4 | 5853798 | [T/G] | Ca03664 | Intron | Zinc finger,RING-type |
| CWSNP2743 | Ca-Kabuli-Chr4 | 5900290 | [T/C] | - | Intergenic | - |
| CWSNP2744 | Ca-Kabuli-Chr4 | 5905385 | [G/A] | - | URR | - |
| CWSNP2745 | Ca-Kabuli-Chr4 | 5907409 | [T/G] | - | Intergenic | - |
| CWSNP2746 | Ca-Kabuli-Chr4 | 5907421 | [T/G] | - | Intergenic | - |
| CWSNP2747 | Ca-Kabuli-Chr4 | 5907465 | [C/A] | - | Intergenic | - |
| CWSNP2748 | Ca-Kabuli-Chr4 | 5999964 | [T/C] | Ca03648 | Intron | WD40 repeat |
| CWSNP2749 | Ca-Kabuli-Chr4 | 6000010 | [T/A] | Ca03648 | Intron | WD40 repeat |
| CWSNP2750 | Ca-Kabuli-Chr4 | 6000013 | [A/C] | Ca03648 | Intron | WD40 repeat |
| CWSNP2751 | Ca-Kabuli-Chr4 | 6000037 | [T/G] | Ca03648 | Intron | WD40 repeat |
| CWSNP2752 | Ca-Kabuli-Chr4 | 6041134 | [A/C] | - | URR | - |

| SNP IDs | Chromosomes | Physical positions (bp) | SNPs | Gene accession IDs | Sequence components of genes | Putative functions |
|---|---|---|---|---|---|---|
| CWSNP2753 | Ca-Kabuli-Chr4 | 6041130 | [C/A] | - | URR | - |
| CWSNP2754 | Ca-Kabuli-Chr4 | 6041126 | [A/G] | - | URR | - |
| CWSNP2755 | Ca-Kabuli-Chr4 | 6041120 | [G/T] | - | URR | - |
| CWSNP2756 | Ca-Kabuli-Chr4 | 6041117 | [C/T] | - | URR | - |
| CWSNP2757 | Ca-Kabuli-Chr4 | 6157625 | [T/C] | Ca03628 | Non-Synonymous-CDS | PeptidaseS16,Lonprotease,C-terminal |
| CWSNP2758 | Ca-Kabuli-Chr4 | 6201137 | [T/C] | - | Intergenic | - |
| CWSNP2759 | Ca-Kabuli-Chr4 | 6281228 | [A/C] | - | DRR | - |
| CWSNP2760 | Ca-Kabuli-Chr4 | 6387938 | [T/G] | - | DRR | - |
| CWSNP2761 | Ca-Kabuli-Chr4 | 6403910 | [A/C] | - | Intergenic | - |
| CWSNP2762 | Ca-Kabuli-Chr4 | 6408543 | [T/G] | - | Intergenic | - |
| CWSNP2763 | Ca-Kabuli-Chr4 | 6544342 | [A/C] | - | Intergenic | - |
| CWSNP2764 | Ca-Kabuli-Chr4 | 6544337 | [A/C] | - | Intergenic | - |
| CWSNP2765 | Ca-Kabuli-Chr4 | 6688218 | [G/A] | - | Intergenic | - |
| CWSNP2766 | Ca-Kabuli-Chr4 | 6749550 | [G/A] | - | URR | - |
| CWSNP2767 | Ca-Kabuli-Chr4 | 6761778 | [C/A] | Ca03565 | Non-Synonymous-CDS | Pectinacetylesterase |
| CWSNP2768 | Ca-Kabuli-Chr4 | 6765884 | [T/C] | Ca03564 | Non-Synonymous-CDS | SANT domain, DNA binding |

| SNP IDs | Chromosomes | Physical positions (bp) | SNPs | Gene accession IDs | Sequence components of genes | Putative functions |
|---|---|---|---|---|---|---|
| CWSNP2769 | Ca-Kabuli-Chr4 | 6801632 | [C/A] | - | URR | - |
| CWSNP2770 | Ca-Kabuli-Chr4 | 6841854 | [G/A] | - | URR | - |
| CWSNP2771 | Ca-Kabuli-Chr4 | 6841856 | [G/A] | - | URR | - |
| CWSNP2772 | Ca-Kabuli-Chr4 | 6841858 | [G/A] | - | URR | - |
| CWSNP2773 | Ca-Kabuli-Chr4 | 6841859 | [A/C] | - | URR | - |
| CWSNP2774 | Ca-Kabuli-Chr4 | 6841860 | [G/A] | - | URR | - |
| CWSNP2775 | Ca-Kabuli-Chr4 | 6889964 | [T/G] | - | URR | - |
| CWSNP2776 | Ca-Kabuli-Chr4 | 6889974 | [T/G] | - | URR | - |
| CWSNP2777 | Ca-Kabuli-Chr4 | 6890000 | [A/G] | Ca03550 | Synonymous-CDS | Pectinesterase,catalytic |
| CWSNP2778 | Ca-Kabuli-Chr4 | 6890005 | [C/T] | Ca03550 | Non-Synonymous-CDS | Pectinesterase,catalytic |
| CWSNP2779 | Ca-Kabuli-Chr4 | 6890014 | [G/T] | Ca03550 | Non-Synonymous-CDS | Pectinesterase,catalytic |
| CWSNP2780 | Ca-Kabuli-Chr4 | 6889994 | [T/G] | Ca03550 | Non-Synonymous-CDS | Pectinesterase,catalytic |
| CWSNP2781 | Ca-Kabuli-Chr4 | 6895804 | [G/A] | Ca03549 | Synonymous-CDS | Protein kinase, catalytic domain |
| CWSNP2782 | Ca-Kabuli-Chr4 | 6919896 | [C/T] | Ca03548 | Non-Synonymous-CDS | Transcription factor,SBP-box |
| CWSNP2783 | Ca-Kabuli-Chr4 | 6969799 | [T/C] | - | Intergenic | - |
| CWSNP2784 | Ca-Kabuli-Chr4 | 7142498 | [G/A] | - | Intergenic | - |

| SNP IDs | Chromosomes | Physical positions (bp) | SNPs | Gene accession IDs | Sequence components of genes | Putative functions |
|---|---|---|---|---|---|---|
| CWSNP2785 | Ca-Kabuli-Chr4 | 7143238 | [C/T] | - | DRR | - |
| CWSNP2786 | Ca-Kabuli-Chr4 | 7155826 | [G/A] | - | Intergenic | - |
| CWSNP2787 | Ca-Kabuli-Chr4 | 7335587 | [C/T] | Ca03503 | Non-Synonymous-CDS | BRCT |
| CWSNP2788 | Ca-Kabuli-Chr4 | 7349958 | [T/C] | Ca03501 | Non-Synonymous-CDS | Lipase,class3 |
| CWSNP2789 | Ca-Kabuli-Chr4 | 7349948 | [T/C] | Ca03501 | Synonymous-CDS | Lipase,class3 |
| CWSNP2790 | Ca-Kabuli-Chr4 | 7350192 | [G/T] | Ca03501 | Non-Synonymous-CDS | Lipase,class3 |
| CWSNP2791 | Ca-Kabuli-Chr4 | 7371708 | [T/C] | - | Intergenic | - |
| CWSNP2792 | Ca-Kabuli-Chr4 | 7819865 | [T/C] | Ca03457 | Intron | Glycosidehydrolase,family1 |
| CWSNP2793 | Ca-Kabuli-Chr4 | 7868480 | [A/T] | - | DRR | - |
| CWSNP2794 | Ca-Kabuli-Chr4 | 8050770 | [T/C] | Ca03429 | Non-Synonymous-CDS | Tubby,C-terminal |
| CWSNP2795 | Ca-Kabuli-Chr4 | 8051306 | [T/C] | Ca03429 | Intron | Tubby,C-terminal |
| CWSNP2796 | Ca-Kabuli-Chr4 | 8198702 | [T/G] | Ca08309 | Non-Synonymous-CDS | WD40 repeat |
| CWSNP2797 | Ca-Kabuli-Chr4 | 8269099 | [T/G] | Ca08315 | Intron | Protein of unknown function DUF3595 |
| CWSNP2798 | Ca-Kabuli-Chr4 | 8269347 | [C/G] | Ca08315 | Intron | Protein of unknown function DUF3595 |
| CWSNP2799 | Ca-Kabuli-Chr4 | 8383890 | [G/A] | Ca08327 | Intron | BSD |
| CWSNP2800 | Ca-Kabuli-Chr4 | 8396861 | [T/A] | - | Intergenic | - |

| SNP IDs | Chromosomes | Physical positions (bp) | SNPs | Gene accession IDs | Sequence components of genes | Putative functions |
|---|---|---|---|---|---|---|
| CWSNP2801 | Ca-Kabuli-Chr4 | 8396837 | [T/G] | - | Intergenic | - |
| CWSNP2802 | Ca-Kabuli-Chr4 | 8401109 | [A/G] | Ca08329 | Intron | ChaperoninCpn60 |
| CWSNP2803 | Ca-Kabuli-Chr4 | 8401126 | [G/T] | Ca08329 | Intron | ChaperoninCpn60 |
| CWSNP2804 | Ca-Kabuli-Chr4 | 8401143 | [A/T] | Ca08329 | Synonymous-CDS | ChaperoninCpn60 |
| CWSNP2805 | Ca-Kabuli-Chr4 | 8401248 | [T/A] | - | Intergenic | - |
| CWSNP2806 | Ca-Kabuli-Chr4 | 8401230 | [A/G] | - | Intergenic | - |
| CWSNP2807 | Ca-Kabuli-Chr4 | 8404165 | [C/A] | - | URR | - |
| CWSNP2808 | Ca-Kabuli-Chr4 | 8471029 | [G/A] | - | Intergenic | - |
| CWSNP2809 | Ca-Kabuli-Chr4 | 8473120 | [G/A] | Ca08339 | Intron | Phosphofructokinase domain |
| CWSNP2810 | Ca-Kabuli-Chr4 | 8473127 | [G/A] | Ca08339 | Intron | Phosphofructokinase domain |
| CWSNP2811 | Ca-Kabuli-Chr4 | 8473294 | [G/A] | Ca08339 | Intron | Phosphofructokinase domain |
| CWSNP2812 | Ca-Kabuli-Chr4 | 8473288 | [A/G] | Ca08339 | Intron | Phosphofructokinase domain |
| CWSNP2813 | Ca-Kabuli-Chr4 | 8473246 | [T/C] | Ca08339 | Intron | Phosphofructokinase domain |
| CWSNP2814 | Ca-Kabuli-Chr4 | 8475634 | [A/C] | Ca08339 | Synonymous-CDS | Phosphofructokinase domain |
| CWSNP2815 | Ca-Kabuli-Chr4 | 8483482 | [T/C] | Ca08340 | Synonymous-CDS | WD40 repeat |
| CWSNP2816 | Ca-Kabuli-Chr4 | 8483494 | [T/C] | Ca08340 | Synonymous-CDS | WD40 repeat |

| SNP IDs | Chromosomes | Physical positions (bp) | SNPs | Gene accession IDs | Sequence components of genes | Putative functions |
|---|---|---|---|---|---|---|
| CWSNP2817 | Ca-Kabuli-Chr4 | 8496641 | [C/A] | Ca08341 | Synonymous-CDS | WD40 repeat |
| CWSNP2818 | Ca-Kabuli-Chr4 | 8510011 | [A/C] | Ca08343 | Synonymous-CDS | - |
| CWSNP2819 | Ca-Kabuli-Chr4 | 8512095 | [G/A] | Ca08343 | Synonymous-CDS | - |
| CWSNP2820 | Ca-Kabuli-Chr4 | 8518979 | [G/A] | - | Intergenic | - |
| CWSNP2821 | Ca-Kabuli-Chr4 | 8601364 | [T/G] | Ca08354 | Intron | Exo70exocystcomplexsubunit |
| CWSNP2822 | Ca-Kabuli-Chr4 | 8659774 | [G/A] | - | URR | - |
| CWSNP2823 | Ca-Kabuli-Chr4 | 8660115 | [A/T] | - | URR | - |
| CWSNP2824 | Ca-Kabuli-Chr4 | 8668648 | [G/A] | - | URR | - |
| CWSNP2825 | Ca-Kabuli-Chr4 | 8668753 | [A/G] | - | URR | - |
| CWSNP2826 | Ca-Kabuli-Chr4 | 8668945 | [T/C] | - | URR | - |
| CWSNP2827 | Ca-Kabuli-Chr4 | 8669515 | [C/T] | - | URR | - |
| CWSNP2828 | Ca-Kabuli-Chr4 | 8669513 | [G/A] | - | URR | - |
| CWSNP2829 | Ca-Kabuli-Chr4 | 8669505 | [G/T] | - | URR | - |
| CWSNP2830 | Ca-Kabuli-Chr4 | 8681949 | [G/A] | - | URR | - |
| CWSNP2831 | Ca-Kabuli-Chr4 | 8695562 | [C/T] | Ca08365 | Intron | Glycosidehydrolase,family13,N-terminal |
| CWSNP2832 | Ca-Kabuli-Chr4 | 8751638 | [A/C] | Ca08371 | Synonymous-CDS | No apical meristem (NAM) protein |

| SNP IDs | Chromosomes | Physical positions (bp) | SNPs | Gene accession IDs | Sequence components of genes | Putative functions |
|---|---|---|---|---|---|---|
| CWSNP2833 | Ca-Kabuli-Chr4 | 8751629 | [T/C] | Ca08371 | Synonymous-CDS | No apical meristem (NAM) protein |
| CWSNP2834 | Ca-Kabuli-Chr4 | 8751618 | [A/C] | Ca08371 | Non-Synonymous-CDS | No apical meristem (NAM) protein |
| CWSNP2835 | Ca-Kabuli-Chr4 | 8751591 | [A/C] | - | Intergenic | - |
| CWSNP2836 | Ca-Kabuli-Chr4 | 8782879 | [G/C] | - | URR | - |
| CWSNP2837 | Ca-Kabuli-Chr4 | 8791654 | [A/G] | Ca08375 | Synonymous-CDS | Kelchrepeattype2 |
| CWSNP2838 | Ca-Kabuli-Chr4 | 8805504 | [A/G] | - | DRR | - |
| CWSNP2839 | Ca-Kabuli-Chr4 | 8805578 | [A/C] | - | DRR | - |
| CWSNP2840 | Ca-Kabuli-Chr4 | 8805667 | [C/T] | - | DRR | - |
| CWSNP2841 | Ca-Kabuli-Chr4 | 8805867 | [A/C] | - | DRR | - |
| CWSNP2842 | Ca-Kabuli-Chr4 | 8805839 | [G/A] | - | DRR | - |
| CWSNP2843 | Ca-Kabuli-Chr4 | 8809032 | [C/T] | Ca08378 | Intron | K Homology |
| CWSNP2844 | Ca-Kabuli-Chr4 | 8809064 | [T/C] | Ca08378 | Synonymous-CDS | K Homology |
| CWSNP2845 | Ca-Kabuli-Chr4 | 8809456 | [A/C] | - | URR | - |
| CWSNP2846 | Ca-Kabuli-Chr4 | 8844846 | [A/G] | Ca08381 | Synonymous-CDS | Zinc finger,RING-type |
| CWSNP2847 | Ca-Kabuli-Chr4 | 8848654 | [C/T] | Ca08381 | Synonymous-CDS | Zinc finger,RING-type |
| CWSNP2848 | Ca-Kabuli-Chr4 | 8848653 | [T/C] | Ca08381 | Non-Synonymous-CDS | Zinc finger,RING-type |

| SNP IDs | Chromosomes | Physical positions (bp) | SNPs | Gene accession IDs | Sequence components of genes | Putative functions |
|---|---|---|---|---|---|---|
| CWSNP2849 | Ca-Kabuli-Chr4 | 8852420 | [T/C] | Ca08382 | Synonymous-CDS | - |
| CWSNP2850 | Ca-Kabuli-Chr4 | 8919395 | [A/G] | - | URR | - |
| CWSNP2851 | Ca-Kabuli-Chr4 | 8986031 | [T/A] | - | DRR | - |
| CWSNP2852 | Ca-Kabuli-Chr4 | 8986026 | [T/A] | - | DRR | - |
| CWSNP2853 | Ca-Kabuli-Chr4 | 8986024 | [T/A] | - | DRR | - |
| CWSNP2854 | Ca-Kabuli-Chr4 | 8986018 | [G/A] | - | DRR | - |
| CWSNP2855 | Ca-Kabuli-Chr4 | 8986008 | [T/A] | - | DRR | - |
| CWSNP2856 | Ca-Kabuli-Chr4 | 8986002 | [T/A] | - | DRR | - |
| CWSNP2857 | Ca-Kabuli-Chr4 | 8985998 | [T/A] | - | DRR | - |
| CWSNP2858 | Ca-Kabuli-Chr4 | 9075398 | [G/A] | - | Intergenic | - |
| CWSNP2859 | Ca-Kabuli-Chr4 | 9075395 | [A/G] | - | Intergenic | - |
| CWSNP2860 | Ca-Kabuli-Chr4 | 9075459 | [C/T] | - | Intergenic | - |
| CWSNP2861 | Ca-Kabuli-Chr4 | 9075520 | [C/T] | - | Intergenic | - |
| CWSNP2862 | Ca-Kabuli-Chr4 | 9075565 | [A/G] | - | Intergenic | - |
| CWSNP2863 | Ca-Kabuli-Chr4 | 9075543 | [T/C] | - | Intergenic | - |
| CWSNP2864 | Ca-Kabuli-Chr4 | 9075671 | [A/G] | - | Intergenic | - |

| SNP IDs | Chromosomes | Physical positions (bp) | SNPs | Gene accession IDs | Sequence components of genes | Putative functions |
|---|---|---|---|---|---|---|
| CWSNP2865 | Ca-Kabuli-Chr4 | 9075677 | [T/C] | - | Intergenic | - |
| CWSNP2866 | Ca-Kabuli-Chr4 | 9075682 | [G/A] | - | Intergenic | - |
| CWSNP2867 | Ca-Kabuli-Chr4 | 9075737 | [G/A] | - | Intergenic | - |
| CWSNP2868 | Ca-Kabuli-Chr4 | 9075744 | [T/G] | - | Intergenic | - |
| CWSNP2869 | Ca-Kabuli-Chr4 | 9187887 | [G/C] | Ca08410 | Non-Synonymous-CDS | Vps51/Vps67 |
| CWSNP2870 | Ca-Kabuli-Chr4 | 9187973 | [T/G] | Ca08410 | Synonymous-CDS | Vps51/Vps67 |
| CWSNP2871 | Ca-Kabuli-Chr4 | 9189976 | [T/A] | Ca08410 | Non-Synonymous-CDS | Vps51/Vps67 |
| CWSNP2872 | Ca-Kabuli-Chr4 | 9331949 | [C/T] | - | DRR | - |
| CWSNP2873 | Ca-Kabuli-Chr4 | 9451895 | [T/A] | - | Intergenic | - |
| CWSNP2874 | Ca-Kabuli-Chr4 | 9451850 | [T/G] | - | Intergenic | - |
| CWSNP2875 | Ca-Kabuli-Chr4 | 9579519 | [A/T] | - | Intergenic | - |
| CWSNP2876 | Ca-Kabuli-Chr4 | 9579517 | [T/C] | - | Intergenic | - |
| CWSNP2877 | Ca-Kabuli-Chr4 | 9614905 | [C/A] | Ca08443 | Non-Synonymous-CDS | - |
| CWSNP2878 | Ca-Kabuli-Chr4 | 9706453 | [T/G] | - | DRR | - |
| CWSNP2879 | Ca-Kabuli-Chr4 | 9836559 | [G/T] | Ca08462 | Intron | VHS |
| CWSNP2880 | Ca-Kabuli-Chr4 | 10007069 | [A/G] | - | Intergenic | - |

| SNP IDs | Chromosomes | Physical positions (bp) | SNPs | Gene accession IDs | Sequence components of genes | Putative functions |
|---|---|---|---|---|---|---|
| CWSNP2881 | Ca-Kabuli-Chr4 | 10116516 | [T/G] | Ca08486 | Synonymous-CDS | Pleckstrin homology domain |
| CWSNP2882 | Ca-Kabuli-Chr4 | 10151943 | [C/T] | Ca08487 | Non-Synonymous-CDS | Zinc finger,GATA-type |
| CWSNP2883 | Ca-Kabuli-Chr4 | 10184428 | [G/T] | Ca08489 | Non-Synonymous-CDS | GCN5-relatedN-acetyltransferase (GNAT) domain |
| CWSNP2884 | Ca-Kabuli-Chr4 | 10184427 | [C/A] | Ca08489 | Synonymous-CDS | GCN5-relatedN-acetyltransferase (GNAT) domain |
| CWSNP2885 | Ca-Kabuli-Chr4 | 10294380 | [A/G] | Ca08496 | Intron | Carbamoyl-phosphatesynthase,GATase domain |
| CWSNP2886 | Ca-Kabuli-Chr4 | 10305285 | [C/A] | Ca08496 | Intron | Carbamoyl-phosphatesynthase,GATase domain |
| CWSNP2887 | Ca-Kabuli-Chr4 | 10411809 | [G/A] | - | Intergenic | - |
| CWSNP2888 | Ca-Kabuli-Chr4 | 10422142 | [A/T] | - | Intergenic | - |
| CWSNP2889 | Ca-Kabuli-Chr4 | 10422469 | [C/A] | - | DRR | - |
| CWSNP2890 | Ca-Kabuli-Chr4 | 10430622 | [A/C] | - | URR | - |
| CWSNP2891 | Ca-Kabuli-Chr4 | 10430617 | [A/C] | - | URR | - |
| CWSNP2892 | Ca-Kabuli-Chr4 | 10430613 | [C/A] | - | URR | - |
| CWSNP2893 | Ca-Kabuli-Chr4 | 10430603 | [G/A] | - | URR | - |
| CWSNP2894 | Ca-Kabuli-Chr4 | 10430595 | [A/C] | - | URR | - |
| CWSNP2895 | Ca-Kabuli-Chr4 | 10430588 | [T/C] | - | URR | - |
| CWSNP2896 | Ca-Kabuli-Chr4 | 10430574 | [A/T] | - | URR | - |

| SNP IDs | Chromosomes | Physical positions (bp) | SNPs | Gene accession IDs | Sequence components of genes | Putative functions |
|---|---|---|---|---|---|---|
| CWSNP2897 | Ca-Kabuli-Chr4 | 10464927 | [T/G] | - | DRR | - |
| CWSNP2898 | Ca-Kabuli-Chr4 | 10465328 | [T/C] | - | DRR | - |
| CWSNP2899 | Ca-Kabuli-Chr4 | 10901282 | [T/C] | - | DRR | - |
| CWSNP2900 | Ca-Kabuli-Chr4 | 10917717 | [C/G] | - | Intergenic | - |
| CWSNP2901 | Ca-Kabuli-Chr4 | 10925490 | [G/A] | Ca04326 | Synonymous-CDS | Filamin/ABP280repeat-like |
| CWSNP2902 | Ca-Kabuli-Chr4 | 10971039 | [C/A] | Ca04330 | Intron | WD40 repeat |
| CWSNP2903 | Ca-Kabuli-Chr4 | 10990000 | [T/C] | Ca04331 | Intron | Protein kinase, catalytic domain |
| CWSNP2904 | Ca-Kabuli-Chr4 | 10990049 | [G/T] | Ca04331 | Intron | Protein kinase, catalytic domain |
| CWSNP2905 | Ca-Kabuli-Chr4 | 10990058 | [T/C] | Ca04331 | Intron | Protein kinase, catalytic domain |
| CWSNP2906 | Ca-Kabuli-Chr4 | 10990265 | [T/A] | Ca04331 | Intron | Protein kinase, catalytic domain |
| CWSNP2907 | Ca-Kabuli-Chr4 | 10991842 | [G/A] | Ca04331 | Intron | Protein kinase, catalytic domain |
| CWSNP2908 | Ca-Kabuli-Chr4 | 10991910 | [C/T] | Ca04331 | Intron | Protein kinase, catalytic domain |
| CWSNP2909 | Ca-Kabuli-Chr4 | 11040108 | [C/T] | - | Intergenic | - |
| CWSNP2910 | Ca-Kabuli-Chr4 | 11051335 | [C/T] | Ca04337 | Non-Synonymous-CDS | No apical meristem (NAM) protein |
| CWSNP2911 | Ca-Kabuli-Chr4 | 11059403 | [T/G] | - | DRR | - |
| CWSNP2912 | Ca-Kabuli-Chr4 | 11059404 | [T/G] | - | DRR | - |

| SNP IDs | Chromosomes | Physical positions (bp) | SNPs | Gene accession IDs | Sequence components of genes | Putative functions |
|---|---|---|---|---|---|---|
| CWSNP2913 | Ca-Kabuli-Chr4 | 11059414 | [T/G] | - | DRR | - |
| CWSNP2914 | Ca-Kabuli-Chr4 | 11096225 | [A/G] | - | Intergenic | - |
| CWSNP2915 | Ca-Kabuli-Chr4 | 11096290 | [G/A] | - | Intergenic | - |
| CWSNP2916 | Ca-Kabuli-Chr4 | 11113277 | [A/C] | Ca04344 | Synonymous-CDS | RNA recognition motif domain |
| CWSNP2917 | Ca-Kabuli-Chr4 | 11142202 | [A/G] | - | Intergenic | - |
| CWSNP2918 | Ca-Kabuli-Chr4 | 11142227 | [C/T] | - | Intergenic | - |
| CWSNP2919 | Ca-Kabuli-Chr4 | 11230403 | [G/T] | - | Intergenic | - |
| CWSNP2920 | Ca-Kabuli-Chr4 | 11230400 | [T/A] | - | Intergenic | - |
| CWSNP2921 | Ca-Kabuli-Chr4 | 11230397 | [G/C] | - | Intergenic | - |
| CWSNP2922 | Ca-Kabuli-Chr4 | 11231142 | [A/G] | - | Intergenic | - |
| CWSNP2923 | Ca-Kabuli-Chr4 | 11231137 | [A/G] | - | Intergenic | - |
| CWSNP2924 | Ca-Kabuli-Chr4 | 11231135 | [C/T] | - | Intergenic | - |
| CWSNP2925 | Ca-Kabuli-Chr4 | 11244334 | [T/G] | - | Intergenic | - |
| CWSNP2926 | Ca-Kabuli-Chr4 | 11244395 | [G/T] | - | Intergenic | - |
| CWSNP2927 | Ca-Kabuli-Chr4 | 11246093 | [G/A] | - | Intergenic | - |
| CWSNP2928 | Ca-Kabuli-Chr4 | 11246173 | [C/T] | - | Intergenic | - |

| SNP IDs | Chromosomes | Physical positions (bp) | SNPs | Gene accession IDs | Sequence components of genes | Putative functions |
|---|---|---|---|---|---|---|
| CWSNP2929 | Ca-Kabuli-Chr4 | 11246164 | [G/A] | - | Intergenic | - |
| CWSNP2930 | Ca-Kabuli-Chr4 | 11273328 | [T/C] | Ca04359 | Intron | - |
| CWSNP2931 | Ca-Kabuli-Chr4 | 11273405 | [A/T] | Ca04359 | Intron | - |
| CWSNP2932 | Ca-Kabuli-Chr4 | 11274281 | [T/C] | Ca04359 | Synonymous-CDS | - |
| CWSNP2933 | Ca-Kabuli-Chr4 | 11275171 | [T/C] | Ca04359 | Synonymous-CDS | - |
| CWSNP2934 | Ca-Kabuli-Chr4 | 11276413 | [C/G] | Ca04359 | Intron | - |
| CWSNP2935 | Ca-Kabuli-Chr4 | 11276484 | [T/C] | Ca04359 | Synonymous-CDS | - |
| CWSNP2936 | Ca-Kabuli-Chr4 | 11277138 | [A/G] | Ca04359 | Synonymous-CDS | - |
| CWSNP2937 | Ca-Kabuli-Chr4 | 11277297 | [G/A] | Ca04359 | Synonymous-CDS | - |
| CWSNP2938 | Ca-Kabuli-Chr4 | 11277574 | [G/C] | Ca04359 | Non-Synonymous-CDS | - |
| CWSNP2939 | Ca-Kabuli-Chr4 | 11334350 | [A/G] | - | URR | - |
| CWSNP2940 | Ca-Kabuli-Chr4 | 11334343 | [A/T] | - | URR | - |
| CWSNP2941 | Ca-Kabuli-Chr4 | 11343257 | [T/C] | Ca04368 | Intron | Zinc finger,DHHC-type,palmitoyltransferase |
| CWSNP2942 | Ca-Kabuli-Chr4 | 11343230 | [G/C] | Ca04368 | Intron | Zinc finger,DHHC-type,palmitoyltransferase |
| CWSNP2943 | Ca-Kabuli-Chr4 | 11379077 | [T/C] | - | Intergenic | - |
| CWSNP2944 | Ca-Kabuli-Chr4 | 11398699 | [A/G] | - | Intergenic | - |

| SNP IDs | Chromosomes | Physical positions (bp) | SNPs | Gene accession IDs | Sequence components of genes | Putative functions |
|---|---|---|---|---|---|---|
| CWSNP2945 | Ca-Kabuli-Chr4 | 11398682 | [G/A] | - | Intergenic | - |
| CWSNP2946 | Ca-Kabuli-Chr4 | 11398889 | [A/G] | - | Intergenic | - |
| CWSNP2947 | Ca-Kabuli-Chr4 | 11435651 | [A/G] | - | Intergenic | - |
| CWSNP2948 | Ca-Kabuli-Chr4 | 11441604 | [T/A] | - | DRR | - |
| CWSNP2949 | Ca-Kabuli-Chr4 | 11465057 | [C/G] | - | DRR | - |
| CWSNP2950 | Ca-Kabuli-Chr4 | 11465075 | [T/C] | - | DRR | - |
| CWSNP2951 | Ca-Kabuli-Chr4 | 11465113 | [C/T] | - | DRR | - |
| CWSNP2952 | Ca-Kabuli-Chr4 | 11490100 | [C/T] | Ca04384 | Synonymous-CDS | Tubby,C-terminal |
| CWSNP2953 | Ca-Kabuli-Chr4 | 11490154 | [A/T] | Ca04384 | Synonymous-CDS | Tubby,C-terminal |
| CWSNP2954 | Ca-Kabuli-Chr4 | 11490125 | [G/T] | Ca04384 | Synonymous-CDS | Tubby,C-terminal |
| CWSNP2955 | Ca-Kabuli-Chr4 | 11490496 | [G/A] | Ca04384 | Synonymous-CDS | Tubby,C-terminal |
| CWSNP2956 | Ca-Kabuli-Chr4 | 11517582 | [C/T] | - | Intergenic | - |
| CWSNP2957 | Ca-Kabuli-Chr4 | 11517517 | [C/A] | - | Intergenic | - |
| CWSNP2958 | Ca-Kabuli-Chr4 | 11572051 | [C/T] | - | Intergenic | - |
| CWSNP2959 | Ca-Kabuli-Chr4 | 11646453 | [G/A] | Ca04392 | Non-Synonymous-CDS | Protein synthesis factor, GTP-binding |
| CWSNP2960 | Ca-Kabuli-Chr4 | 11658314 | [A/G] | - | DRR | - |

| SNP IDs | Chromosomes | Physical positions (bp) | SNPs | Gene accession IDs | Sequence components of genes | Putative functions |
|---|---|---|---|---|---|---|
| CWSNP2961 | Ca-Kabuli-Chr4 | 11689611 | [C/T] | Ca04395 | Non-Synonymous-CDS | Basic-leucine zipper (bZIP) Transcription factor |
| CWSNP2962 | Ca-Kabuli-Chr4 | 11752862 | [A/G] | Ca04400 | Synonymous-CDS | - |
| CWSNP2963 | Ca-Kabuli-Chr4 | 11752855 | [A/G] | Ca04400 | Non-Synonymous-CDS | - |
| CWSNP2964 | Ca-Kabuli-Chr4 | 11772023 | [G/T] | Ca04401 | Non-Synonymous-CDS | Zinc finger,FYVE-type |
| CWSNP2965 | Ca-Kabuli-Chr4 | 11926352 | [G/C] | Ca04414 | Intron | ATPase,AAA+type,core |
| CWSNP2966 | Ca-Kabuli-Chr4 | 12004082 | [A/T] | - | DRR | - |
| CWSNP2967 | Ca-Kabuli-Chr4 | 12004209 | [C/T] | - | DRR | - |
| CWSNP2968 | Ca-Kabuli-Chr4 | 12004205 | [A/C] | - | DRR | - |
| CWSNP2969 | Ca-Kabuli-Chr4 | 12167328 | [A/G] | - | DRR | - |
| CWSNP2970 | Ca-Kabuli-Chr4 | 12167568 | [G/A] | - | DRR | - |
| CWSNP2971 | Ca-Kabuli-Chr4 | 12249494 | [T/C] | Ca04448 | Intron | Protein kinase, catalytic domain |
| CWSNP2972 | Ca-Kabuli-Chr4 | 12484911 | [A/T] | - | Intergenic | - |
| CWSNP2973 | Ca-Kabuli-Chr4 | 12506440 | [A/G] | - | Intergenic | - |
| CWSNP2974 | Ca-Kabuli-Chr4 | 12506450 | [T/A] | - | Intergenic | - |
| CWSNP2975 | Ca-Kabuli-Chr4 | 12514728 | [G/A] | - | Intergenic | - |
| CWSNP2976 | Ca-Kabuli-Chr4 | 12514726 | [C/T] | - | Intergenic | - |

| SNP IDs | Chromosomes | Physical positions (bp) | SNPs | Gene accession IDs | Sequence components of genes | Putative functions |
|---|---|---|---|---|---|---|
| CWSNP2977 | Ca-Kabuli-Chr4 | 12514724 | [T/C] | - | Intergenic | - |
| CWSNP2978 | Ca-Kabuli-Chr4 | 12514706 | [G/A] | - | Intergenic | - |
| CWSNP2979 | Ca-Kabuli-Chr4 | 12525328 | [A/G] | Ca04475 | Synonymous-CDS | Cytochromeb561,eukaryote |
| CWSNP2980 | Ca-Kabuli-Chr4 | 12525639 | [T/A] | Ca04475 | Intron | Cytochromeb561,eukaryote |
| CWSNP2981 | Ca-Kabuli-Chr4 | 12534847 | [T/C] | - | Intergenic | - |
| CWSNP2982 | Ca-Kabuli-Chr4 | 12558541 | [T/C] | Ca04478 | Intron | NUDIX hydrolase domain |
| CWSNP2983 | Ca-Kabuli-Chr4 | 12558680 | [C/T] | Ca04478 | Intron | NUDIX hydrolase domain |
| CWSNP2984 | Ca-Kabuli-Chr4 | 12568707 | [G/A] | Ca04479 | Synonymous-CDS | Homoserinekinase |
| CWSNP2985 | Ca-Kabuli-Chr4 | 12568845 | [G/T] | Ca04479 | Synonymous-CDS | Homoserinekinase |
| CWSNP2986 | Ca-Kabuli-Chr4 | 12568905 | [G/A] | Ca04479 | Synonymous-CDS | Homoserinekinase |
| CWSNP2987 | Ca-Kabuli-Chr4 | 12568944 | [A/G] | Ca04479 | Synonymous-CDS | Homoserinekinase |
| CWSNP2988 | Ca-Kabuli-Chr4 | 12574650 | [G/A] | Ca04480 | Non-Synonymous-CDS | DNA mismatch repair protein MutS, C-terminaldomain |
| CWSNP2989 | Ca-Kabuli-Chr4 | 12578259 | [A/G] | Ca04480 | Synonymous-CDS | DNA mismatch repair protein MutS, C-terminaldomain |
| CWSNP2990 | Ca-Kabuli-Chr4 | 12578350 | [A/G] | Ca04480 | Intron | DNA mismatch repair protein MutS, C-terminaldomain |
| CWSNP2991 | Ca-Kabuli-Chr4 | 12581371 | [G/T] | Ca04480 | Synonymous-CDS | DNA mismatch repair protein MutS, C-terminaldomain |
| CWSNP2992 | Ca-Kabuli-Chr4 | 12740351 | [T/G] | Ca04493 | Synonymous-CDS | IQ motif, EF-hand binding site |

| SNP IDs | Chromosomes | Physical positions (bp) | SNPs | Gene accession IDs | Sequence components of genes | Putative functions |
|---|---|---|---|---|---|---|
| CWSNP2993 | Ca-Kabuli-Chr4 | 12869023 | [C/T] | - | URR | - |
| CWSNP2994 | Ca-Kabuli-Chr4 | 12973657 | [C/A] | Ca04519 | Non-Synonymous-CDS | GDP-fucose proteinO-fucosyltransferase |
| CWSNP2995 | Ca-Kabuli-Chr4 | 12982420 | [G/A] | Ca04520 | Intron | Alpha-D-phosphohexomutase |
| CWSNP2996 | Ca-Kabuli-Chr4 | 13179829 | [T/C] | - | DRR | - |
| CWSNP2997 | Ca-Kabuli-Chr4 | 13203008 | [A/T] | - | Intergenic | - |
| CWSNP2998 | Ca-Kabuli-Chr4 | 13229035 | [C/T] | - | Intergenic | - |
| CWSNP2999 | Ca-Kabuli-Chr4 | 13252015 | [A/T] | - | Intergenic | - |
| CWSNP3000 | Ca-Kabuli-Chr4 | 13252077 | [C/G] | - | Intergenic | - |
| CWSNP3001 | Ca-Kabuli-Chr4 | 13252212 | [G/A] | - | Intergenic | - |
| CWSNP3002 | Ca-Kabuli-Chr4 | 13282984 | [A/C] | - | URR | - |
| CWSNP3003 | Ca-Kabuli-Chr4 | 13588956 | [C/T] | Ca04577 | Synonymous-CDS | Biopterin transport-related protein BT1 |
| CWSNP3004 | Ca-Kabuli-Chr4 | 13588928 | [T/C] | Ca04577 | Intron | Biopterin transport-related protein BT1 |
| CWSNP3005 | Ca-Kabuli-Chr4 | 13687456 | [G/A] | Ca04589 | Intron | Frigida-like |
| CWSNP3006 | Ca-Kabuli-Chr4 | 13713380 | [G/T] | - | Intergenic | - |
| CWSNP3007 | Ca-Kabuli-Chr4 | 13713367 | [G/C] | - | Intergenic | - |
| CWSNP3008 | Ca-Kabuli-Chr4 | 13724666 | [G/C] | Ca04596 | Non-Synonymous-CDS | Zinc finger,PHD-type |

| SNP IDs | Chromosomes | Physical positions (bp) | SNPs | Gene accession IDs | Sequence components of genes | Putative functions |
|---|---|---|---|---|---|---|
| CWSNP3009 | Ca-Kabuli-Chr4 | 13787448 | [A/C] | - | DRR | - |
| CWSNP3010 | Ca-Kabuli-Chr4 | 13787649 | [T/C] | - | Intergenic | - |
| CWSNP3011 | Ca-Kabuli-Chr4 | 13787720 | [G/C] | - | Intergenic | - |
| CWSNP3012 | Ca-Kabuli-Chr4 | 13838796 | [A/G] | Ca04608 | Synonymous-CDS | - |
| CWSNP3013 | Ca-Kabuli-Chr4 | 13839294 | [A/G] | Ca04608 | Synonymous-CDS | - |
| CWSNP3014 | Ca-Kabuli-Chr4 | 13839288 | [G/A] | Ca04608 | Synonymous-CDS | - |
| CWSNP3015 | Ca-Kabuli-Chr4 | 13840191 | [T/A] | Ca04608 | Synonymous-CDS | - |
| CWSNP3016 | Ca-Kabuli-Chr4 | 13840227 | [C/A] | Ca04608 | Synonymous-CDS | - |
| CWSNP3017 | Ca-Kabuli-Chr4 | 13840251 | [A/G] | Ca04608 | Synonymous-CDS | - |
| CWSNP3018 | Ca-Kabuli-Chr4 | 13840484 | [T/C] | Ca04608 | Synonymous-CDS | - |
| CWSNP3019 | Ca-Kabuli-Chr4 | 13841340 | [G/T] | Ca04608 | Non-Synonymous-CDS | - |
| CWSNP3020 | Ca-Kabuli-Chr4 | 13845581 | [A/T] | - | Intergenic | - |
| CWSNP3021 | Ca-Kabuli-Chr4 | 13845639 | [A/C] | - | Intergenic | - |
| CWSNP3022 | Ca-Kabuli-Chr4 | 13845675 | [T/A] | - | URR | - |
| CWSNP3023 | Ca-Kabuli-Chr4 | 13900013 | [A/G] | - | Intergenic | - |
| CWSNP3024 | Ca-Kabuli-Chr4 | 13949513 | [A/C] | - | Intergenic | - |

| SNP IDs | Chromosomes | Physical positions (bp) | SNPs | Gene accession IDs | Sequence components of genes | Putative functions |
|---|---|---|---|---|---|---|
| CWSNP3025 | Ca-Kabuli-Chr4 | 13949580 | [C/G] | - | Intergenic | - |
| CWSNP3026 | Ca-Kabuli-Chr4 | 13954832 | [G/A] | - | Intergenic | - |
| CWSNP3027 | Ca-Kabuli-Chr4 | 13968291 | [C/T] | - | Intergenic | - |
| CWSNP3028 | Ca-Kabuli-Chr4 | 13968397 | [A/G] | - | Intergenic | - |
| CWSNP3029 | Ca-Kabuli-Chr4 | 13968354 | [A/G] | - | Intergenic | - |
| CWSNP3030 | Ca-Kabuli-Chr4 | 13982970 | [G/A] | - | URR | - |
| CWSNP3031 | Ca-Kabuli-Chr4 | 14031548 | [A/G] | - | Intergenic | - |
| CWSNP3032 | Ca-Kabuli-Chr4 | 14056908 | [A/G] | - | DRR | - |
| CWSNP3033 | Ca-Kabuli-Chr4 | 14073059 | [C/T] | - | URR | - |
| CWSNP3034 | Ca-Kabuli-Chr4 | 14073056 | [C/T] | - | URR | - |
| CWSNP3035 | Ca-Kabuli-Chr4 | 14073045 | [G/A] | - | URR | - |
| CWSNP3036 | Ca-Kabuli-Chr4 | 14073044 | [C/T] | - | URR | - |
| CWSNP3037 | Ca-Kabuli-Chr4 | 14073004 | [C/A] | - | URR | - |
| CWSNP3038 | Ca-Kabuli-Chr4 | 14082157 | [C/A] | Ca04635 | Intron | PDZ-binding protein,CRIPT |
| CWSNP3039 | Ca-Kabuli-Chr4 | 14082158 | [T/G] | Ca04635 | Intron | PDZ-binding protein,CRIPT |
| CWSNP3040 | Ca-Kabuli-Chr4 | 14082186 | [G/A] | Ca04635 | Intron | PDZ-binding protein,CRIPT |

| SNP IDs | Chromosomes | Physical positions (bp) | SNPs | Gene accession IDs | Sequence components of genes | Putative functions |
|---|---|---|---|---|---|---|
| CWSNP3041 | Ca-Kabuli-Chr4 | 14082210 | [T/C] | Ca04635 | Intron | PDZ-binding protein,CRIPT |
| CWSNP3042 | Ca-Kabuli-Chr4 | 14082285 | [C/G] | - | Intergenic | - |
| CWSNP3043 | Ca-Kabuli-Chr4 | 14093027 | [A/G] | Ca04636 | Synonymous-CDS | Ubiquitin |
| CWSNP3044 | Ca-Kabuli-Chr4 | 14104096 | [A/C] | - | URR | - |
| CWSNP3045 | Ca-Kabuli-Chr4 | 14112911 | [C/A] | - | Intergenic | - |
| CWSNP3046 | Ca-Kabuli-Chr4 | 14132659 | [A/C] | - | Intergenic | - |
| CWSNP3047 | Ca-Kabuli-Chr4 | 14149589 | [G/A] | Ca04645 | Non-Synonymous-CDS | Protein kinase, catalytic domain |
| CWSNP3048 | Ca-Kabuli-Chr4 | 14149588 | [A/G] | Ca04645 | Non-Synonymous-CDS | Protein kinase, catalytic domain |
| CWSNP3049 | Ca-Kabuli-Chr4 | 14155978 | [A/G] | Ca04646 | Synonymous-CDS | Protein kinase, catalytic domain |
| CWSNP3050 | Ca-Kabuli-Chr4 | 14156014 | [C/T] | Ca04646 | Synonymous-CDS | Protein kinase, catalytic domain |
| CWSNP3051 | Ca-Kabuli-Chr4 | 14189356 | [A/C] | Ca04652 | Non-Synonymous-CDS | Protein kinase, catalytic domain |
| CWSNP3052 | Ca-Kabuli-Chr4 | 14270763 | [G/A] | - | DRR | - |
| CWSNP3053 | Ca-Kabuli-Chr4 | 14515543 | [T/A] | - | URR | - |
| CWSNP3054 | Ca-Kabuli-Chr4 | 14657743 | [C/T] | Ca05639 | Intron | Protein of unknown function DUF566 |
| CWSNP3055 | Ca-Kabuli-Chr4 | 14748051 | [A/C] | Ca05628 | Synonymous-CDS | Protein of unknown function DUF620 |
| CWSNP3056 | Ca-Kabuli-Chr4 | 14785557 | [A/C] | - | Intergenic | - |

| SNP IDs | Chromosomes | Physical positions (bp) | SNPs | Gene accession IDs | Sequence components of genes | Putative functions |
|---|---|---|---|---|---|---|
| CWSNP3057 | Ca-Kabuli-Chr4 | 14831204 | [A/T] | - | Intergenic | - |
| CWSNP3058 | Ca-Kabuli-Chr4 | 14870658 | [T/G] | Ca05614 | Non-Synonymous-CDS | Amino glycosidephospho transferase |
| CWSNP3059 | Ca-Kabuli-Chr4 | 14870641 | [G/A] | Ca05614 | Synonymous-CDS | Amino glycosidephospho transferase |
| CWSNP3060 | Ca-Kabuli-Chr4 | 15240678 | [T/C] | - | DRR | - |
| CWSNP3061 | Ca-Kabuli-Chr4 | 15357695 | [A/G] | - | URR | - |
| CWSNP3062 | Ca-Kabuli-Chr4 | 15394470 | [T/C] | - | DRR | - |
| CWSNP3063 | Ca-Kabuli-Chr4 | 15542597 | [A/G] | Ca05547 | Synonymous-CDS | Zinc finger,CCCH-type |
| CWSNP3064 | Ca-Kabuli-Chr4 | 15552690 | [C/T] | - | Intergenic | - |
| CWSNP3065 | Ca-Kabuli-Chr4 | 15697128 | [A/G] | Ca05536 | Non-Synonymous-CDS | Phox/Bem1p |
| CWSNP3066 | Ca-Kabuli-Chr4 | 15697303 | [G/T] | Ca05536 | Synonymous-CDS | Phox/Bem1p |
| CWSNP3067 | Ca-Kabuli-Chr4 | 15698212 | [G/T] | - | DRR | - |
| CWSNP3068 | Ca-Kabuli-Chr4 | 15698179 | [G/A] | - | DRR | - |
| CWSNP3069 | Ca-Kabuli-Chr4 | 15698233 | [A/C] | - | DRR | - |
| CWSNP3070 | Ca-Kabuli-Chr4 | 15698305 | [T/C] | - | DRR | - |
| CWSNP3071 | Ca-Kabuli-Chr4 | 15698486 | [T/C] | - | DRR | - |
| CWSNP3072 | Ca-Kabuli-Chr4 | 15698456 | [T/C] | - | DRR | - |

| SNP IDs | Chromosomes | Physical positions (bp) | SNPs | Gene accession IDs | Sequence components of genes | Putative functions |
|---|---|---|---|---|---|---|
| CWSNP3073 | Ca-Kabuli-Chr4 | 15698444 | [C/T] | - | DRR | - |
| CWSNP3074 | Ca-Kabuli-Chr4 | 15698420 | [G/C] | - | DRR | - |
| CWSNP3075 | Ca-Kabuli-Chr4 | 15700603 | [A/T] | - | Intergenic | - |
| CWSNP3076 | Ca-Kabuli-Chr4 | 15736619 | [T/G] | Ca05530 | Intron | Cytidylyltransferase |
| CWSNP3077 | Ca-Kabuli-Chr4 | 15752567 | [G/C] | Ca05529 | Non-Synonymous-CDS | Glycosyltransferase,family20 |
| CWSNP3078 | Ca-Kabuli-Chr4 | 15772275 | [A/G] | Ca05528 | Synonymous-CDS | Reticulon |
| CWSNP3079 | Ca-Kabuli-Chr4 | 15825621 | [A/T] | Ca05524 | Intron | Protein of unknown function DUF789 |
| CWSNP3080 | Ca-Kabuli-Chr4 | 15860388 | [A/G] | - | DRR | - |
| CWSNP3081 | Ca-Kabuli-Chr4 | 15860359 | [C/A] | - | DRR | - |
| CWSNP3082 | Ca-Kabuli-Chr4 | 15900602 | [G/A] | - | URR | - |
| CWSNP3083 | Ca-Kabuli-Chr4 | 15925936 | [T/G] | - | DRR | - |
| CWSNP3084 | Ca-Kabuli-Chr4 | 15926160 | [A/G] | - | DRR | - |
| CWSNP3085 | Ca-Kabuli-Chr4 | 15934607 | [A/T] | Ca05513 | Intron | Beta-lactamase-like |
| CWSNP3086 | Ca-Kabuli-Chr4 | 15934901 | [T/G] | - | URR | - |
| CWSNP3087 | Ca-Kabuli-Chr4 | 15957070 | [T/A] | - | Intergenic | - |
| CWSNP3088 | Ca-Kabuli-Chr4 | 16045870 | [G/A] | Ca05504 | Intron | Glutamyl/glutaminyl-tRNA synthetase,classIc |

| SNP IDs | Chromosomes | Physical positions (bp) | SNPs | Gene accession IDs | Sequence components of genes | Putative functions |
|---|---|---|---|---|---|---|
| CWSNP3089 | Ca-Kabuli-Chr4 | 16046928 | [T/C] | Ca05504 | Synonymous-CDS | Glutamyl/glutaminyl-tRNA synthetase,classIc |
| CWSNP3090 | Ca-Kabuli-Chr4 | 16278600 | [C/T] | Ca05487 | Non-Synonymous-CDS | Zinc finger,B-box |
| CWSNP3091 | Ca-Kabuli-Chr4 | 16278671 | [T/A] | Ca05487 | Synonymous-CDS | Zinc finger,B-box |
| CWSNP3092 | Ca-Kabuli-Chr4 | 16571288 | [T/C] | Ca05467 | Intron | ATPase,AAA+type,core |
| CWSNP3093 | Ca-Kabuli-Chr4 | 16622514 | [C/A] | Ca05461 | Non-Synonymous-CDS | KOW |
| CWSNP3094 | Ca-Kabuli-Chr4 | 16628559 | [T/G] | - | DRR | - |
| CWSNP3095 | Ca-Kabuli-Chr4 | 16628591 | [T/G] | - | DRR | - |
| CWSNP3096 | Ca-Kabuli-Chr4 | 16628594 | [T/G] | - | DRR | - |
| CWSNP3097 | Ca-Kabuli-Chr4 | 16628540 | [G/A] | - | DRR | - |
| CWSNP3098 | Ca-Kabuli-Chr4 | 16628529 | [C/A] | - | DRR | - |
| CWSNP3099 | Ca-Kabuli-Chr4 | 16629560 | [G/T] | - | DRR | - |
| CWSNP3100 | Ca-Kabuli-Chr4 | 16629559 | [T/G] | - | DRR | - |
| CWSNP3101 | Ca-Kabuli-Chr4 | 16629638 | [C/A] | - | DRR | - |
| CWSNP3102 | Ca-Kabuli-Chr4 | 16749740 | [A/G] | - | Intergenic | - |
| CWSNP3103 | Ca-Kabuli-Chr4 | 16750416 | [A/G] | - | Intergenic | - |
| CWSNP3104 | Ca-Kabuli-Chr4 | 16789476 | [G/A] | Ca05446 | Synonymous-CDS | Haemerythrin/HHEcation-binding motif |

| SNP IDs | Chromosomes | Physical positions (bp) | SNPs | Gene accession IDs | Sequence components of genes | Putative functions |
|---|---|---|---|---|---|---|
| CWSNP3105 | Ca-Kabuli-Chr4 | 16926614 | [A/T] | Ca05430 | Non-Synonymous-CDS | Zinc finger,C2H2-type |
| CWSNP3106 | Ca-Kabuli-Chr4 | 17028368 | [G/A] | - | Intergenic | - |
| CWSNP3107 | Ca-Kabuli-Chr4 | 17032676 | [T/C] | - | URR | - |
| CWSNP3108 | Ca-Kabuli-Chr4 | 17032673 | [C/T] | - | URR | - |
| CWSNP3109 | Ca-Kabuli-Chr4 | 17147875 | [C/T] | - | Intergenic | - |
| CWSNP3110 | Ca-Kabuli-Chr4 | 17147926 | [T/C] | - | Intergenic | - |
| CWSNP3111 | Ca-Kabuli-Chr4 | 17187560 | [C/G] | - | Intergenic | - |
| CWSNP3112 | Ca-Kabuli-Chr4 | 17191562 | [T/A] | Ca05405 | Synonymous-CDS | Protein phosphatase2C,manganese/magnesiumaspartate binding site |
| CWSNP3113 | Ca-Kabuli-Chr4 | 17199316 | [T/C] | Ca05404 | Synonymous-CDS | Domain of unknown function DUF632 |
| CWSNP3114 | Ca-Kabuli-Chr4 | 17199477 | [G/A] | Ca05404 | Synonymous-CDS | Domain of unknown function DUF632 |
| CWSNP3115 | Ca-Kabuli-Chr4 | 17202770 | [A/G] | - | DRR | - |
| CWSNP3116 | Ca-Kabuli-Chr4 | 17225334 | [G/A] | Ca05400 | Synonymous-CDS | HEAT |
| CWSNP3117 | Ca-Kabuli-Chr4 | 17264248 | [C/A] | Ca05398 | Synonymous-CDS | Major facilitator superfamily MFS-1 |
| CWSNP3118 | Ca-Kabuli-Chr4 | 17264592 | [G/A] | Ca05398 | Intron | Major facilitator superfamily MFS-1 |
| CWSNP3119 | Ca-Kabuli-Chr4 | 17264621 | [C/G] | Ca05398 | Intron | Major facilitator superfamily MFS-1 |
| CWSNP3120 | Ca-Kabuli-Chr4 | 17264637 | [C/T] | Ca05398 | Intron | Major facilitator superfamily MFS-1 |

| SNP IDs | Chromosomes | Physical positions (bp) | SNPs | Gene accession IDs | Sequence components of genes | Putative functions |
|---|---|---|---|---|---|---|
| CWSNP3121 | Ca-Kabuli-Chr4 | 17282119 | [T/C] | - | URR | - |
| CWSNP3122 | Ca-Kabuli-Chr4 | 17282148 | [G/C] | - | URR | - |
| CWSNP3123 | Ca-Kabuli-Chr4 | 17282336 | [G/T] | - | URR | - |
| CWSNP3124 | Ca-Kabuli-Chr4 | 17282345 | [G/C] | - | URR | - |
| CWSNP3125 | Ca-Kabuli-Chr4 | 17289594 | [G/C] | - | Intergenic | - |
| CWSNP3126 | Ca-Kabuli-Chr4 | 17297498 | [G/T] | Ca05392 | Intron | Ankyrin repeat-containing domain |
| CWSNP3127 | Ca-Kabuli-Chr4 | 17297567 | [G/A] | Ca05392 | Intron | Ankyrin repeat-containing domain |
| CWSNP3128 | Ca-Kabuli-Chr4 | 17297563 | [A/G] | Ca05392 | Intron | Ankyrin repeat-containing domain |
| CWSNP3129 | Ca-Kabuli-Chr4 | 17309656 | [A/C] | Ca05391 | Synonymous-CDS | Phosphatidyl inositol 3-/4-kinase, catalytic |
| CWSNP3130 | Ca-Kabuli-Chr4 | 17345436 | [G/T] | Ca05389 | Intron | Fibrillarin |
| CWSNP3131 | Ca-Kabuli-Chr4 | 17345500 | [G/A] | Ca05389 | Synonymous-CDS | Fibrillarin |
| CWSNP3132 | Ca-Kabuli-Chr4 | 17345483 | [C/A] | Ca05389 | Intron | Fibrillarin |
| CWSNP3133 | Ca-Kabuli-Chr4 | 17358736 | [C/T] | Ca05388 | Intron | Protein kinase, catalytic domain |
| CWSNP3134 | Ca-Kabuli-Chr4 | 17361143 | [T/A] | Ca05388 | Intron | Protein kinase, catalytic domain |
| CWSNP3135 | Ca-Kabuli-Chr4 | 17396325 | [A/G] | Ca05386 | Intron | Zinc finger,RING-type |
| CWSNP3136 | Ca-Kabuli-Chr4 | 17396349 | [T/G] | Ca05386 | Intron | Zinc finger,RING-type |

| SNP IDs | Chromosomes | Physical positions (bp) | SNPs | Gene accession IDs | Sequence components of genes | Putative functions |
|---|---|---|---|---|---|---|
| CWSNP3137 | Ca-Kabuli-Chr4 | 17396352 | [T/G] | Ca05386 | Intron | Zinc finger,RING-type |
| CWSNP3138 | Ca-Kabuli-Chr4 | 17429456 | [A/T] | Ca05384 | Synonymous-CDS | Transcription factor jumonji/aspartyl beta-hydroxylase |
| CWSNP3139 | Ca-Kabuli-Chr4 | 17429457 | [A/T] | Ca05384 | Synonymous-CDS | Transcription factor jumonji/aspartyl beta-hydroxylase |
| CWSNP3140 | Ca-Kabuli-Chr4 | 17429468 | [T/C] | Ca05384 | Synonymous-CDS | Transcription factor jumonji/aspartyl beta-hydroxylase |
| CWSNP3141 | Ca-Kabuli-Chr4 | 17429747 | [G/A] | Ca05384 | Synonymous-CDS | Transcription factor jumonji/aspartyl beta-hydroxylase |
| CWSNP3142 | Ca-Kabuli-Chr4 | 17429742 | [A/G] | Ca05384 | Synonymous-CDS | Transcription factor jumonji/aspartyl beta-hydroxylase |
| CWSNP3143 | Ca-Kabuli-Chr4 | 17429735 | [A/G] | Ca05384 | Synonymous-CDS | Transcription factor jumonji/aspartyl beta-hydroxylase |
| CWSNP3144 | Ca-Kabuli-Chr4 | 17429699 | [G/C] | Ca05384 | Synonymous-CDS | Transcription factor jumonji/aspartyl beta-hydroxylase |
| CWSNP3145 | Ca-Kabuli-Chr4 | 17433782 | [G/A] | Ca05384 | Intron | Transcription factor jumonji/aspartyl beta-hydroxylase |
| CWSNP3146 | Ca-Kabuli-Chr4 | 17433914 | [G/A] | Ca05384 | Intron | Transcription factor jumonji/aspartyl beta-hydroxylase |
| CWSNP3147 | Ca-Kabuli-Chr4 | 17444578 | [A/C] | - | Intergenic | - |
| CWSNP3148 | Ca-Kabuli-Chr4 | 17472992 | [T/G] | - | DRR | - |
| CWSNP3149 | Ca-Kabuli-Chr4 | 17473234 | [C/A] | - | DRR | - |
| CWSNP3150 | Ca-Kabuli-Chr4 | 17603613 | [T/C] | - | URR | - |
| CWSNP3151 | Ca-Kabuli-Chr4 | 17621035 | [G/T] | Ca05368 | Intron | Dihydroorotatede hydrogenase,conserved site |
| CWSNP3152 | Ca-Kabuli-Chr4 | 17621609 | [G/A] | Ca05368 | Non-Synonymous-CDS | Dihydroorotatede hydrogenase,conserved site |

| SNP IDs | Chromosomes | Physical positions (bp) | SNPs | Gene accession IDs | Sequence components of genes | Putative functions |
|---|---|---|---|---|---|---|
| CWSNP3153 | Ca-Kabuli-Chr4 | 17647558 | [G/A] | - | DRR | - |
| CWSNP3154 | Ca-Kabuli-Chr4 | 17685058 | [T/G] | - | Intergenic | - |
| CWSNP3155 | Ca-Kabuli-Chr4 | 17685113 | [C/A] | - | Intergenic | - |
| CWSNP3156 | Ca-Kabuli-Chr4 | 17718540 | [C/T] | - | DRR | - |
| CWSNP3157 | Ca-Kabuli-Chr4 | 17718536 | [C/A] | - | DRR | - |
| CWSNP3158 | Ca-Kabuli-Chr4 | 17718922 | [T/G] | - | DRR | - |
| CWSNP3159 | Ca-Kabuli-Chr4 | 17718997 | [C/A] | - | DRR | - |
| CWSNP3160 | Ca-Kabuli-Chr4 | 17729988 | [A/C] | Ca05352 | Intron | SNF2-related |
| CWSNP3161 | Ca-Kabuli-Chr4 | 17748171 | [A/T] | - | Intergenic | - |
| CWSNP3162 | Ca-Kabuli-Chr4 | 17752013 | [C/T] | - | DRR | - |
| CWSNP3163 | Ca-Kabuli-Chr4 | 17760841 | [A/G] | Ca05349 | Intron | PeptidaseS9,prolyloligopeptidase,catalytic domain |
| CWSNP3164 | Ca-Kabuli-Chr4 | 17760853 | [T/G] | Ca05349 | Intron | PeptidaseS9,prolyloligopeptidase,catalytic domain |
| CWSNP3165 | Ca-Kabuli-Chr4 | 17771708 | [C/G] | - | Intergenic | - |
| CWSNP3166 | Ca-Kabuli-Chr4 | 17784792 | [T/A] | Ca05346 | Intron | Protein of unknown function DUF639 |
| CWSNP3167 | Ca-Kabuli-Chr4 | 17826472 | [G/A] | - | Intergenic | - |
| CWSNP3168 | Ca-Kabuli-Chr4 | 17826533 | [A/C] | - | Intergenic | - |

| SNP IDs | Chromosomes | Physical positions (bp) | SNPs | Gene accession IDs | Sequence components of genes | Putative functions |
|---|---|---|---|---|---|---|
| CWSNP3169 | Ca-Kabuli-Chr4 | 17826601 | [T/C] | - | Intergenic | - |
| CWSNP3170 | Ca-Kabuli-Chr4 | 17826580 | [C/G] | - | Intergenic | - |
| CWSNP3171 | Ca-Kabuli-Chr4 | 17826543 | [T/C] | - | Intergenic | - |
| CWSNP3172 | Ca-Kabuli-Chr4 | 17847854 | [T/C] | Ca18621 | Intron | Methionyl-tRNA synthetase |
| CWSNP3173 | Ca-Kabuli-Chr4 | 17856307 | [T/C] | Ca18622 | Synonymous-CDS | IKI3 |
| CWSNP3174 | Ca-Kabuli-Chr4 | 17856311 | [T/G] | Ca18622 | Synonymous-CDS | IKI3 |
| CWSNP3175 | Ca-Kabuli-Chr4 | 17856334 | [A/G] | Ca18622 | Synonymous-CDS | IKI3 |
| CWSNP3176 | Ca-Kabuli-Chr4 | 17857753 | [C/T] | Ca18622 | Synonymous-CDS | IKI3 |
| CWSNP3177 | Ca-Kabuli-Chr4 | 17858122 | [T/G] | Ca18622 | Intron | IKI3 |
| CWSNP3178 | Ca-Kabuli-Chr4 | 17858133 | [C/T] | Ca18622 | Intron | IKI3 |
| CWSNP3179 | Ca-Kabuli-Chr4 | 17858135 | [G/A] | Ca18622 | Intron | IKI3 |
| CWSNP3180 | Ca-Kabuli-Chr4 | 17858140 | [A/C] | Ca18622 | Intron | IKI3 |
| CWSNP3181 | Ca-Kabuli-Chr4 | 17858143 | [A/T] | Ca18622 | Intron | IKI3 |
| CWSNP3182 | Ca-Kabuli-Chr4 | 17858144 | [G/C] | Ca18622 | Intron | IKI3 |
| CWSNP3183 | Ca-Kabuli-Chr4 | 17858152 | [A/T] | Ca18622 | Intron | IKI3 |
| CWSNP3184 | Ca-Kabuli-Chr4 | 17858159 | [C/A] | Ca18622 | Intron | IKI3 |

| SNP IDs | Chromosomes | Physical positions (bp) | SNPs | Gene accession IDs | Sequence components of genes | Putative functions |
|---|---|---|---|---|---|---|
| CWSNP3185 | Ca-Kabuli-Chr4 | 17858226 | [C/T] | Ca18622 | Intron | IKI3 |
| CWSNP3186 | Ca-Kabuli-Chr4 | 17871763 | [T/C] | - | URR | - |
| CWSNP3187 | Ca-Kabuli-Chr4 | 17871804 | [T/C] | - | URR | - |
| CWSNP3188 | Ca-Kabuli-Chr4 | 17871828 | [A/C] | - | URR | - |
| CWSNP3189 | Ca-Kabuli-Chr4 | 17871919 | [A/G] | - | URR | - |
| CWSNP3190 | Ca-Kabuli-Chr4 | 17871915 | [T/A] | - | URR | - |
| CWSNP3191 | Ca-Kabuli-Chr4 | 17913731 | [A/C] | - | DRR | - |
| CWSNP3192 | Ca-Kabuli-Chr4 | 17913726 | [G/A] | - | DRR | - |
| CWSNP3193 | Ca-Kabuli-Chr4 | 17913719 | [G/C] | - | DRR | - |
| CWSNP3194 | Ca-Kabuli-Chr4 | 17913698 | [C/T] | - | DRR | - |
| CWSNP3195 | Ca-Kabuli-Chr4 | 17913682 | [C/A] | - | DRR | - |
| CWSNP3196 | Ca-Kabuli-Chr4 | 17920616 | [C/G] | Ca18632 | Synonymous-CDS | ABC transporter, transmembrane domain |
| CWSNP3197 | Ca-Kabuli-Chr4 | 18070293 | [G/T] | Ca18648 | Non-Synonymous-CDS | Ankyrin repeat |
| CWSNP3198 | Ca-Kabuli-Chr4 | 18071114 | [C/A] | Ca18648 | Non-Synonymous-CDS | Ankyrin repeat |
| CWSNP3199 | Ca-Kabuli-Chr4 | 18071324 | [G/A] | Ca18648 | Non-Synonymous-CDS | Ankyrin repeat |
| CWSNP3200 | Ca-Kabuli-Chr4 | 18089423 | [G/C] | Ca18650 | Synonymous-CDS | - |

| SNP IDs | Chromosomes | Physical positions (bp) | SNPs | Gene accession IDs | Sequence components of genes | Putative functions |
|---|---|---|---|---|---|---|
| CWSNP3201 | Ca-Kabuli-Chr4 | 18117833 | [T/C] | - | URR | - |
| CWSNP3202 | Ca-Kabuli-Chr4 | 18231443 | [T/C] | Ca18659 | Intron | SET domain |
| CWSNP3203 | Ca-Kabuli-Chr4 | 18231463 | [T/C] | Ca18659 | Intron | SET domain |
| CWSNP3204 | Ca-Kabuli-Chr4 | 18231466 | [T/C] | Ca18659 | Intron | SET domain |
| CWSNP3205 | Ca-Kabuli-Chr4 | 18242020 | [C/G] | Ca18659 | Intron | SET domain |
| CWSNP3206 | Ca-Kabuli-Chr4 | 18241995 | [C/G] | Ca18659 | Intron | SET domain |
| CWSNP3207 | Ca-Kabuli-Chr4 | 18427385 | [T/G] | Ca21626 | Intron | LisHdimerisationmotif |
| CWSNP3208 | Ca-Kabuli-Chr4 | 18427384 | [C/T] | Ca21626 | Intron | LisHdimerisationmotif |
| CWSNP3209 | Ca-Kabuli-Chr4 | 18429139 | [C/T] | - | Intergenic | - |
| CWSNP3210 | Ca-Kabuli-Chr4 | 18489456 | [G/C] | - | Intergenic | - |
| CWSNP3211 | Ca-Kabuli-Chr4 | 18545489 | [A/T] | Ca21616 | Intron | Protein kinase, catalytic domain |
| CWSNP3212 | Ca-Kabuli-Chr4 | 18546209 | [T/G] | Ca21616 | Synonymous-CDS | Protein kinase, catalytic domain |
| CWSNP3213 | Ca-Kabuli-Chr4 | 18628582 | [A/G] | Ca21604 | Synonymous-CDS | Glycosyltransferase,family48 |
| CWSNP3214 | Ca-Kabuli-Chr4 | 18710941 | [T/G] | Ca13978 | Intron | - |
| CWSNP3215 | Ca-Kabuli-Chr4 | 18719417 | [A/G] | Ca13978 | Intron | - |
| CWSNP3216 | Ca-Kabuli-Chr4 | 18812610 | [T/C] | - | DRR | - |

| SNP IDs | Chromosomes | Physical positions (bp) | SNPs | Gene accession IDs | Sequence components of genes | Putative functions |
|---|---|---|---|---|---|---|
| CWSNP3217 | Ca-Kabuli-Chr4 | 18923641 | [G/T] | Ca13991 | Intron | Nucleotide-sugartransporter |
| CWSNP3218 | Ca-Kabuli-Chr4 | 19024699 | [T/C] | - | Intergenic | - |
| CWSNP3219 | Ca-Kabuli-Chr4 | 19227691 | [G/C] | Ca14021 | Non-Synonymous-CDS | Protein kinase, catalytic domain |
| CWSNP3220 | Ca-Kabuli-Chr4 | 19263967 | [T/G] | Ca14024 | Intron | - |
| CWSNP3221 | Ca-Kabuli-Chr4 | 19267581 | [A/G] | Ca14024 | Intron | - |
| CWSNP3222 | Ca-Kabuli-Chr4 | 19540632 | [A/C] | Ca14045 | Non-Synonymous-CDS | Spo11/DNA topoisomeraseVI,subunitA |
| CWSNP3223 | Ca-Kabuli-Chr4 | 19579442 | [C/A] | - | Intergenic | - |
| CWSNP3224 | Ca-Kabuli-Chr4 | 19668502 | [T/G] | Ca14053 | Synonymous-CDS | Pentatrico peptide repeat |
| CWSNP3225 | Ca-Kabuli-Chr4 | 19713311 | [T/G] | Ca14059 | Synonymous-CDS | - |
| CWSNP3226 | Ca-Kabuli-Chr4 | 19869729 | [G/T] | Ca17123 | Non-Synonymous-CDS | TargetSNAREcoiled-coildomain |
| CWSNP3227 | Ca-Kabuli-Chr4 | 20308992 | [C/A] | Ca17144 | Synonymous-CDS | Phenylalanine/histidineammonia-lyase |
| CWSNP3228 | Ca-Kabuli-Chr4 | 20398685 | [A/C] | Ca17149 | Non-Synonymous-CDS | - |
| CWSNP3229 | Ca-Kabuli-Chr4 | 20599050 | [A/G] | - | Intergenic | - |
| CWSNP3230 | Ca-Kabuli-Chr4 | 20599058 | [T/C] | - | Intergenic | - |
| CWSNP3231 | Ca-Kabuli-Chr4 | 20599081 | [C/T] | - | Intergenic | - |
| CWSNP3232 | Ca-Kabuli-Chr4 | 20599091 | [T/G] | - | Intergenic | - |

| SNP IDs | Chromosomes | Physical positions (bp) | SNPs | Gene accession IDs | Sequence components of genes | Putative functions |
|---|---|---|---|---|---|---|
| CWSNP3233 | Ca-Kabuli-Chr4 | 20656170 | [T/G] | Ca17165 | Synonymous-CDS | NucleoporinNup120/160 |
| CWSNP3234 | Ca-Kabuli-Chr4 | 20669174 | [T/C] | Ca17165 | Intron | NucleoporinNup120/160 |
| CWSNP3235 | Ca-Kabuli-Chr4 | 20669442 | [G/A] | Ca17165 | Intron | NucleoporinNup120/160 |
| CWSNP3236 | Ca-Kabuli-Chr4 | 20696952 | [G/T] | Ca17166 | Synonymous-CDS | ABC transporter, transmembrane domain |
| CWSNP3237 | Ca-Kabuli-Chr4 | 20774180 | [C/T] | Ca17170 | Intron | - |
| CWSNP3238 | Ca-Kabuli-Chr4 | 20905105 | [G/A] | Ca19045 | Intron | Serine/threoninedehydratase,pyridoxal-phosphate-binding site |
| CWSNP3239 | Ca-Kabuli-Chr4 | 21385308 | [A/T] | Ca19066 | Synonymous-CDS | PeptidaseA1 |
| CWSNP3240 | Ca-Kabuli-Chr4 | 21835459 | [T/A] | - | Intergenic | - |
| CWSNP3241 | Ca-Kabuli-Chr4 | 21835457 | [T/A] | - | Intergenic | - |
| CWSNP3242 | Ca-Kabuli-Chr4 | 21835454 | [C/A] | - | Intergenic | - |
| CWSNP3243 | Ca-Kabuli-Chr4 | 21835446 | [T/A] | - | Intergenic | - |
| CWSNP3244 | Ca-Kabuli-Chr4 | 21931559 | [A/G] | - | Intergenic | - |
| CWSNP3245 | Ca-Kabuli-Chr4 | 21931558 | [G/A] | - | Intergenic | - |
| CWSNP3246 | Ca-Kabuli-Chr4 | 21931541 | [C/T] | - | Intergenic | - |
| CWSNP3247 | Ca-Kabuli-Chr4 | 22293495 | [T/C] | - | URR | - |
| CWSNP3248 | Ca-Kabuli-Chr4 | 22850596 | [C/G] | Ca14467 | Non-Synonymous-CDS | - |

| SNP IDs | Chromosomes | Physical positions (bp) | SNPs | Gene accession IDs | Sequence components of genes | Putative functions |
|---|---|---|---|---|---|---|
| CWSNP3249 | Ca-Kabuli-Chr4 | 22859422 | [G/A] | - | DRR | - |
| CWSNP3250 | Ca-Kabuli-Chr4 | 22860281 | [A/C] | - | DRR | - |
| CWSNP3251 | Ca-Kabuli-Chr4 | 22865436 | [A/T] | - | DRR | - |
| CWSNP3252 | Ca-Kabuli-Chr4 | 22865390 | [G/A] | - | DRR | - |
| CWSNP3253 | Ca-Kabuli-Chr4 | 22865389 | [C/T] | - | DRR | - |
| CWSNP3254 | Ca-Kabuli-Chr4 | 22908360 | [A/G] | Ca14463 | Synonymous-CDS | Pentatrico peptide repeat |
| CWSNP3255 | Ca-Kabuli-Chr4 | 22971660 | [G/A] | Ca14460 | Non-Synonymous-CDS | - |
| CWSNP3256 | Ca-Kabuli-Chr4 | 22971766 | [A/G] | Ca14460 | Non-Synonymous-CDS | - |
| CWSNP3257 | Ca-Kabuli-Chr4 | 22971746 | [G/A] | Ca14460 | Synonymous-CDS | - |
| CWSNP3258 | Ca-Kabuli-Chr4 | 22971744 | [A/C] | Ca14460 | Non-Synonymous-CDS | - |
| CWSNP3259 | Ca-Kabuli-Chr4 | 22971847 | [C/A] | Ca14460 | Non-Synonymous-CDS | - |
| CWSNP3260 | Ca-Kabuli-Chr4 | 22971853 | [C/A] | Ca14460 | Non-Synonymous-CDS | - |
| CWSNP3261 | Ca-Kabuli-Chr4 | 22971884 | [G/A] | Ca14460 | Synonymous-CDS | - |
| CWSNP3262 | Ca-Kabuli-Chr4 | 23314665 | [A/C] | - | Intergenic | - |
| CWSNP3263 | Ca-Kabuli-Chr4 | 23337312 | [T/G] | - | Intergenic | - |
| CWSNP3264 | Ca-Kabuli-Chr4 | 23481919 | [G/A] | - | Intergenic | - |

| SNP IDs | Chromosomes | Physical positions (bp) | SNPs | Gene accession IDs | Sequence components of genes | Putative functions |
|---|---|---|---|---|---|---|
| CWSNP3265 | Ca-Kabuli-Chr4 | 23481927 | [G/T] | - | Intergenic | - |
| CWSNP3266 | Ca-Kabuli-Chr4 | 23481962 | [G/A] | - | Intergenic | - |
| CWSNP3267 | Ca-Kabuli-Chr4 | 23482033 | [A/G] | - | Intergenic | - |
| CWSNP3268 | Ca-Kabuli-Chr4 | 23481998 | [T/C] | - | Intergenic | - |
| CWSNP3269 | Ca-Kabuli-Chr4 | 23486812 | [A/G] | - | Intergenic | - |
| CWSNP3270 | Ca-Kabuli-Chr4 | 23486859 | [A/G] | - | Intergenic | - |
| CWSNP3271 | Ca-Kabuli-Chr4 | 23486824 | [T/C] | - | Intergenic | - |
| CWSNP3272 | Ca-Kabuli-Chr4 | 24120866 | [A/G] | - | Intergenic | - |
| CWSNP3273 | Ca-Kabuli-Chr4 | 24614582 | [G/T] | - | URR | - |
| CWSNP3274 | Ca-Kabuli-Chr4 | 24729302 | [T/C] | Ca20853 | Intron | Protein kinase, catalytic domain |
| CWSNP3275 | Ca-Kabuli-Chr4 | 24732991 | [G/T] | Ca20853 | Intron | Protein kinase, catalytic domain |
| CWSNP3276 | Ca-Kabuli-Chr4 | 24732990 | [T/C] | Ca20853 | Intron | Protein kinase, catalytic domain |
| CWSNP3277 | Ca-Kabuli-Chr4 | 24771737 | [T/A] | Ca20851 | Non-Synonymous-CDS | - |
| CWSNP3278 | Ca-Kabuli-Chr4 | 24782714 | [C/A] | Ca20850 | Non-Synonymous-CDS | - |
| CWSNP3279 | Ca-Kabuli-Chr4 | 24782715 | [A/G] | Ca20850 | Non-Synonymous-CDS | - |
| CWSNP3280 | Ca-Kabuli-Chr4 | 24856115 | [A/C] | Ca20847 | Non-Synonymous-CDS | Protein kinase, catalytic domain |

| SNP IDs | Chromosomes | Physical positions (bp) | SNPs | Gene accession IDs | Sequence components of genes | Putative functions |
|---|---|---|---|---|---|---|
| CWSNP3281 | Ca-Kabuli-Chr4 | 24857501 | [A/G] | Ca20847 | Intron | Protein kinase, catalytic domain |
| CWSNP3282 | Ca-Kabuli-Chr4 | 25401714 | [T/C] | - | Intergenic | - |
| CWSNP3283 | Ca-Kabuli-Chr4 | 25446684 | [T/C] | - | Intergenic | - |
| CWSNP3284 | Ca-Kabuli-Chr4 | 25455156 | [G/A] | - | Intergenic | - |
| CWSNP3285 | Ca-Kabuli-Chr4 | 25455204 | [A/G] | - | Intergenic | - |
| CWSNP3286 | Ca-Kabuli-Chr4 | 25592487 | [A/G] | - | Intergenic | - |
| CWSNP3287 | Ca-Kabuli-Chr4 | 25592484 | [A/C] | - | Intergenic | - |
| CWSNP3288 | Ca-Kabuli-Chr4 | 25729905 | [C/A] | - | Intergenic | - |
| CWSNP3289 | Ca-Kabuli-Chr4 | 25729977 | [C/T] | - | Intergenic | - |
| CWSNP3290 | Ca-Kabuli-Chr4 | 25747256 | [T/C] | - | Intergenic | - |
| CWSNP3291 | Ca-Kabuli-Chr4 | 25747262 | [G/A] | - | Intergenic | - |
| CWSNP3292 | Ca-Kabuli-Chr4 | 25747274 | [T/G] | - | Intergenic | - |
| CWSNP3293 | Ca-Kabuli-Chr4 | 25747275 | [G/A] | - | Intergenic | - |
| CWSNP3294 | Ca-Kabuli-Chr4 | 25747281 | [T/C] | - | Intergenic | - |
| CWSNP3295 | Ca-Kabuli-Chr4 | 25747295 | [G/A] | - | Intergenic | - |
| CWSNP3296 | Ca-Kabuli-Chr4 | 25747296 | [T/C] | - | Intergenic | - |

| SNP IDs | Chromosomes | Physical positions (bp) | SNPs | Gene accession IDs | Sequence components of genes | Putative functions |
|---|---|---|---|---|---|---|
| CWSNP3297 | Ca-Kabuli-Chr4 | 25809665 | [T/C] | Ca16599 | Intron | Amino glycosidephospho transferase |
| CWSNP3298 | Ca-Kabuli-Chr4 | 25921165 | [T/C] | Ca16606 | Intron | Thiolase |
| CWSNP3299 | Ca-Kabuli-Chr4 | 26000532 | [C/T] | - | DRR | - |
| CWSNP3300 | Ca-Kabuli-Chr4 | 26000483 | [T/A] | - | DRR | - |
| CWSNP3301 | Ca-Kabuli-Chr4 | 26002991 | [C/A] | Ca16610 | Intron | Aldo/ketoreductase |
| CWSNP3302 | Ca-Kabuli-Chr4 | 26008744 | [G/A] | - | URR | - |
| CWSNP3303 | Ca-Kabuli-Chr4 | 26008745 | [G/A] | - | URR | - |
| CWSNP3304 | Ca-Kabuli-Chr4 | 26008748 | [C/T] | - | URR | - |
| CWSNP3305 | Ca-Kabuli-Chr4 | 26008768 | [A/G] | - | URR | - |
| CWSNP3306 | Ca-Kabuli-Chr4 | 26008816 | [C/A] | - | URR | - |
| CWSNP3307 | Ca-Kabuli-Chr4 | 26009438 | [G/T] | - | Intergenic | - |
| CWSNP3308 | Ca-Kabuli-Chr4 | 26009439 | [G/T] | - | Intergenic | - |
| CWSNP3309 | Ca-Kabuli-Chr4 | 26222180 | [A/G] | - | Intergenic | - |
| CWSNP3310 | Ca-Kabuli-Chr4 | 26529606 | [C/T] | Ca25003 | Intron | RNA recognition motif domain |
| CWSNP3311 | Ca-Kabuli-Chr4 | 26532763 | [T/G] | Ca25003 | Intron | RNA recognition motif domain |
| CWSNP3312 | Ca-Kabuli-Chr4 | 26537053 | [C/T] | - | Intergenic | - |

| SNP IDs | Chromosomes | Physical positions (bp) | SNPs | Gene accession IDs | Sequence components of genes | Putative functions |
|---|---|---|---|---|---|---|
| CWSNP3313 | Ca-Kabuli-Chr4 | 26537258 | [A/C] | - | Intergenic | - |
| CWSNP3314 | Ca-Kabuli-Chr4 | 26915621 | [G/A] | - | URR | - |
| CWSNP3315 | Ca-Kabuli-Chr4 | 26915626 | [C/T] | - | URR | - |
| CWSNP3316 | Ca-Kabuli-Chr4 | 26989204 | [A/C] | Ca20453 | Non-Synonymous-CDS | Ribosomal proteinS3,C-terminal |
| CWSNP3317 | Ca-Kabuli-Chr4 | 27102817 | [A/C] | - | Intergenic | - |
| CWSNP3318 | Ca-Kabuli-Chr4 | 27102833 | [G/T] | - | Intergenic | - |
| CWSNP3319 | Ca-Kabuli-Chr4 | 27262893 | [G/A] | - | Intergenic | - |
| CWSNP3320 | Ca-Kabuli-Chr4 | 27268301 | [G/A] | - | Intergenic | - |
| CWSNP3321 | Ca-Kabuli-Chr4 | 27493010 | [G/T] | Ca21317 | Synonymous-CDS | Protein of unknown function DUF716,TMEM45 |
| CWSNP3322 | Ca-Kabuli-Chr4 | 27613450 | [C/T] | Ca21314 | Intron | PeptidaseC48,SUMO/Sentrin/Ubl1 |
| CWSNP3323 | Ca-Kabuli-Chr4 | 27617803 | [A/G] | - | DRR | - |
| CWSNP3324 | Ca-Kabuli-Chr4 | 27626559 | [G/A] | Ca21312 | Synonymous-CDS | Protein of unknown function DUF810 |
| CWSNP3325 | Ca-Kabuli-Chr4 | 27626999 | [C/T] | Ca21312 | Synonymous-CDS | Protein of unknown function DUF810 |
| CWSNP3326 | Ca-Kabuli-Chr4 | 27661241 | [T/G] | Ca21309 | Synonymous-CDS | - |
| CWSNP3327 | Ca-Kabuli-Chr4 | 27669829 | [C/G] | - | URR | - |
| CWSNP3328 | Ca-Kabuli-Chr4 | 27777968 | [A/C] | Ca21303 | Intron | MATH |

| SNP IDs | Chromosomes | Physical positions (bp) | SNPs | Gene accession IDs | Sequence components of genes | Putative functions |
|---|---|---|---|---|---|---|
| CWSNP3329 | Ca-Kabuli-Chr4 | 27786162 | [G/C] | Ca21303 | Intron | MATH |
| CWSNP3330 | Ca-Kabuli-Chr4 | 27970989 | [T/G] | Ca21299 | Synonymous-CDS | GAF |
| CWSNP3331 | Ca-Kabuli-Chr4 | 28061002 | [A/C] | Ca23178 | Synonymous-CDS | - |
| CWSNP3332 | Ca-Kabuli-Chr4 | 28092609 | [G/A] | Ca23175 | Non-Synonymous-CDS | - |
| CWSNP3333 | Ca-Kabuli-Chr4 | 28092727 | [T/A] | Ca23175 | Non-Synonymous-CDS | Crotonase,core |
| CWSNP3334 | Ca-Kabuli-Chr4 | 28092725 | [T/G] | Ca23175 | Non-Synonymous-CDS | Crotonase,core |
| CWSNP3335 | Ca-Kabuli-Chr4 | 28092724 | [T/A] | Ca23175 | Non-Synonymous-CDS | Crotonase,core |
| CWSNP3336 | Ca-Kabuli-Chr4 | 28289900 | [C/A] | - | Intergenic | - |
| CWSNP3337 | Ca-Kabuli-Chr4 | 28299238 | [G/A] | - | Intergenic | - |
| CWSNP3338 | Ca-Kabuli-Chr4 | 28301853 | [T/C] | - | Intergenic | - |
| CWSNP3339 | Ca-Kabuli-Chr4 | 28423079 | [T/C] | - | Intergenic | - |
| CWSNP3340 | Ca-Kabuli-Chr4 | 28423140 | [A/G] | - | Intergenic | - |
| CWSNP3341 | Ca-Kabuli-Chr4 | 28431706 | [G/A] | - | Intergenic | - |
| CWSNP3342 | Ca-Kabuli-Chr4 | 28790085 | [A/G] | - | DRR | - |
| CWSNP3343 | Ca-Kabuli-Chr4 | 29582296 | [C/T] | - | Intergenic | - |
| CWSNP3344 | Ca-Kabuli-Chr4 | 29582289 | [G/A] | - | Intergenic | - |

| SNP IDs | Chromosomes | Physical positions (bp) | SNPs | Gene accession IDs | Sequence components of genes | Putative functions |
|---|---|---|---|---|---|---|
| CWSNP3345 | Ca-Kabuli-Chr4 | 29582285 | [C/A] | - | Intergenic | - |
| CWSNP3346 | Ca-Kabuli-Chr4 | 29582282 | [C/A] | - | Intergenic | - |
| CWSNP3347 | Ca-Kabuli-Chr4 | 29582273 | [G/A] | - | Intergenic | - |
| CWSNP3348 | Ca-Kabuli-Chr4 | 29582268 | [C/G] | - | Intergenic | - |
| CWSNP3349 | Ca-Kabuli-Chr4 | 29582257 | [G/A] | - | Intergenic | - |
| CWSNP3350 | Ca-Kabuli-Chr4 | 29582255 | [C/T] | - | Intergenic | - |
| CWSNP3351 | Ca-Kabuli-Chr4 | 29582252 | [G/A] | - | Intergenic | - |
| CWSNP3352 | Ca-Kabuli-Chr4 | 29582249 | [C/T] | - | Intergenic | - |
| CWSNP3353 | Ca-Kabuli-Chr4 | 29582235 | [T/A] | - | Intergenic | - |
| CWSNP3354 | Ca-Kabuli-Chr4 | 29582232 | [C/T] | - | Intergenic | - |
| CWSNP3355 | Ca-Kabuli-Chr4 | 29582227 | [G/A] | - | Intergenic | - |
| CWSNP3356 | Ca-Kabuli-Chr4 | 29582219 | [A/C] | - | Intergenic | - |
| CWSNP3357 | Ca-Kabuli-Chr4 | 29582271 | [C/T] | - | Intergenic | - |
| CWSNP3358 | Ca-Kabuli-Chr4 | 29582286 | [T/A] | - | Intergenic | - |
| CWSNP3359 | Ca-Kabuli-Chr4 | 29582294 | [G/A] | - | Intergenic | - |
| CWSNP3360 | Ca-Kabuli-Chr4 | 29582301 | [G/T] | - | Intergenic | - |

| SNP IDs | Chromosomes | Physical positions (bp) | SNPs | Gene accession IDs | Sequence components of genes | Putative functions |
|---|---|---|---|---|---|---|
| CWSNP3361 | Ca-Kabuli-Chr4 | 29671563 | [G/A] | - | Intergenic | - |
| CWSNP3362 | Ca-Kabuli-Chr4 | 29671606 | [A/G] | - | Intergenic | - |
| CWSNP3363 | Ca-Kabuli-Chr4 | 29683718 | [A/T] | Ca14166 | Non-Synonymous-CDS | Kinesin , motor domain |
| CWSNP3364 | Ca-Kabuli-Chr4 | 29923380 | [G/A] | Ca14177 | Synonymous-CDS | Plant peroxidase |
| CWSNP3365 | Ca-Kabuli-Chr4 | 29958122 | [T/G] | - | Intergenic | - |
| CWSNP3366 | Ca-Kabuli-Chr4 | 29958113 | [G/A] | - | Intergenic | - |
| CWSNP3367 | Ca-Kabuli-Chr4 | 29972268 | [T/G] | - | Intergenic | - |
| CWSNP3368 | Ca-Kabuli-Chr4 | 30153390 | [G/A] | - | Intergenic | - |
| CWSNP3369 | Ca-Kabuli-Chr4 | 30153448 | [T/C] | - | Intergenic | - |
| CWSNP3370 | Ca-Kabuli-Chr4 | 30153505 | [C/G] | - | Intergenic | - |
| CWSNP3371 | Ca-Kabuli-Chr4 | 30258044 | [A/T] | - | Intergenic | - |
| CWSNP3372 | Ca-Kabuli-Chr4 | 30258045 | [A/T] | - | Intergenic | - |
| CWSNP3373 | Ca-Kabuli-Chr4 | 30258056 | [A/G] | - | Intergenic | - |
| CWSNP3374 | Ca-Kabuli-Chr4 | 30258484 | [G/C] | - | Intergenic | - |
| CWSNP3375 | Ca-Kabuli-Chr4 | 30258613 | [C/A] | - | Intergenic | - |
| CWSNP3376 | Ca-Kabuli-Chr4 | 30258607 | [C/A] | - | Intergenic | - |

| SNP IDs | Chromosomes | Physical positions (bp) | SNPs | Gene accession IDs | Sequence components of genes | Putative functions |
|---|---|---|---|---|---|---|
| CWSNP3377 | Ca-Kabuli-Chr4 | 30258604 | [C/A] | - | Intergenic | - |
| CWSNP3378 | Ca-Kabuli-Chr4 | 30258596 | [G/C] | - | Intergenic | - |
| CWSNP3379 | Ca-Kabuli-Chr4 | 30258567 | [C/A] | - | Intergenic | - |
| CWSNP3380 | Ca-Kabuli-Chr4 | 30260485 | [C/A] | - | Intergenic | - |
| CWSNP3381 | Ca-Kabuli-Chr4 | 30281991 | [A/G] | - | Intergenic | - |
| CWSNP3382 | Ca-Kabuli-Chr4 | 30301002 | [T/G] | Ca14192 | Synonymous-CDS | SNF2-related |
| CWSNP3383 | Ca-Kabuli-Chr4 | 30308719 | [A/C] | Ca14192 | Intron | SNF2-related |
| CWSNP3384 | Ca-Kabuli-Chr4 | 30308812 | [G/A] | Ca14192 | Intron | SNF2-related |
| CWSNP3385 | Ca-Kabuli-Chr4 | 30308788 | [T/C] | Ca14192 | Intron | SNF2-related |
| CWSNP3386 | Ca-Kabuli-Chr4 | 30463063 | [T/C] | - | URR | - |
| CWSNP3387 | Ca-Kabuli-Chr4 | 30463117 | [C/A] | - | URR | - |
| CWSNP3388 | Ca-Kabuli-Chr4 | 30599096 | [C/A] | Ca14207 | Synonymous-CDS | Protein kinase, catalytic domain |
| CWSNP3389 | Ca-Kabuli-Chr4 | 30599188 | [C/T] | Ca14207 | Intron | Protein kinase, catalytic domain |
| CWSNP3390 | Ca-Kabuli-Chr4 | 30668893 | [A/C] | - | Intergenic | - |
| CWSNP3391 | Ca-Kabuli-Chr4 | 30851027 | [A/T] | Ca14212 | Intron | Basic-leucine zipper (bZIP) Transcription factor |
| CWSNP3392 | Ca-Kabuli-Chr4 | 30851411 | [T/G] | Ca14212 | Intron | Basic-leucine zipper (bZIP) Transcription factor |

| SNP IDs | Chromosomes | Physical positions (bp) | SNPs | Gene accession IDs | Sequence components of genes | Putative functions |
|---|---|---|---|---|---|---|
| CWSNP3393 | Ca-Kabuli-Chr4 | 30851840 | [A/T] | Ca14212 | Intron | Basic-leucine zipper (bZIP) Transcription factor |
| CWSNP3394 | Ca-Kabuli-Chr4 | 30931586 | [G/T] | - | Intergenic | - |
| CWSNP3395 | Ca-Kabuli-Chr4 | 31218647 | [T/C] | Ca14226 | Intron | Ovariantumour,otubain |
| CWSNP3396 | Ca-Kabuli-Chr4 | 31221264 | [T/G] | - | Intergenic | - |
| CWSNP3397 | Ca-Kabuli-Chr4 | 31221253 | [G/A] | - | Intergenic | - |
| CWSNP3398 | Ca-Kabuli-Chr4 | 31221226 | [T/A] | - | Intergenic | - |
| CWSNP3399 | Ca-Kabuli-Chr4 | 31224001 | [G/A] | - | URR | - |
| CWSNP3400 | Ca-Kabuli-Chr4 | 31262596 | [T/A] | - | URR | - |
| CWSNP3401 | Ca-Kabuli-Chr4 | 31263205 | [T/A] | Ca14230 | Non-Synonymous-CDS | Pentatrico peptide repeat |
| CWSNP3402 | Ca-Kabuli-Chr4 | 31594126 | [A/C] | - | Intergenic | - |
| CWSNP3403 | Ca-Kabuli-Chr4 | 31618949 | [T/A] | - | Intergenic | - |
| CWSNP3404 | Ca-Kabuli-Chr4 | 31987890 | [A/C] | - | Intergenic | - |
| CWSNP3405 | Ca-Kabuli-Chr4 | 32042488 | [T/G] | Ca15459 | Synonymous-CDS | Domain of unknown function DUF828 |
| CWSNP3406 | Ca-Kabuli-Chr4 | 32068961 | [A/G] | Ca15461 | Synonymous-CDS | Ankyrin repeat |
| CWSNP3407 | Ca-Kabuli-Chr4 | 32073552 | [T/C] | - | DRR | - |
| CWSNP3408 | Ca-Kabuli-Chr4 | 32073550 | [C/G] | - | DRR | - |

| SNP IDs | Chromosomes | Physical positions (bp) | SNPs | Gene accession IDs | Sequence components of genes | Putative functions |
|---|---|---|---|---|---|---|
| CWSNP3409 | Ca-Kabuli-Chr4 | 32073549 | [G/C] | - | DRR | - |
| CWSNP3410 | Ca-Kabuli-Chr4 | 32166559 | [C/G] | - | Intergenic | - |
| CWSNP3411 | Ca-Kabuli-Chr4 | 32166557 | [T/A] | - | Intergenic | - |
| CWSNP3412 | Ca-Kabuli-Chr4 | 32183307 | [C/G] | Ca15464 | Synonymous-CDS | Pentatrico peptide repeat |
| CWSNP3413 | Ca-Kabuli-Chr4 | 32184628 | [A/G] | Ca15464 | Synonymous-CDS | Pentatrico peptide repeat |
| CWSNP3414 | Ca-Kabuli-Chr4 | 32212899 | [G/A] | - | Intergenic | - |
| CWSNP3415 | Ca-Kabuli-Chr4 | 32303394 | [C/T] | - | DRR | - |
| CWSNP3416 | Ca-Kabuli-Chr4 | 32429228 | [A/T] | Ca15476 | Non-Synonymous-CDS | Armadillo |
| CWSNP3417 | Ca-Kabuli-Chr4 | 32429222 | [A/C] | Ca15476 | Synonymous-CDS | Armadillo |
| CWSNP3418 | Ca-Kabuli-Chr4 | 32521346 | [T/C] | Ca15480 | Intron | - |
| CWSNP3419 | Ca-Kabuli-Chr4 | 32602658 | [C/T] | - | Intergenic | - |
| CWSNP3420 | Ca-Kabuli-Chr4 | 32604538 | [T/C] | Ca15484 | Synonymous-CDS | Pentatrico peptide repeat |
| CWSNP3421 | Ca-Kabuli-Chr4 | 32604510 | [A/C] | Ca15484 | Non-Synonymous-CDS | Pentatrico peptide repeat |
| CWSNP3422 | Ca-Kabuli-Chr4 | 32636315 | [T/C] | - | Intergenic | - |
| CWSNP3423 | Ca-Kabuli-Chr4 | 32636428 | [C/A] | - | Intergenic | - |
| CWSNP3424 | Ca-Kabuli-Chr4 | 32636459 | [A/G] | - | Intergenic | - |

| SNP IDs | Chromosomes | Physical positions (bp) | SNPs | Gene accession IDs | Sequence components of genes | Putative functions |
|---|---|---|---|---|---|---|
| CWSNP3425 | Ca-Kabuli-Chr4 | 32636690 | [C/T] | - | Intergenic | - |
| CWSNP3426 | Ca-Kabuli-Chr4 | 32639121 | [C/T] | - | Intergenic | - |
| CWSNP3427 | Ca-Kabuli-Chr4 | 32639188 | [C/G] | - | Intergenic | - |
| CWSNP3428 | Ca-Kabuli-Chr4 | 32639118 | [A/C] | - | Intergenic | - |
| CWSNP3429 | Ca-Kabuli-Chr4 | 32639315 | [A/G] | - | Intergenic | - |
| CWSNP3430 | Ca-Kabuli-Chr4 | 32639654 | [T/C] | - | Intergenic | - |
| CWSNP3431 | Ca-Kabuli-Chr4 | 32639681 | [C/T] | - | URR | - |
| CWSNP3432 | Ca-Kabuli-Chr4 | 32853834 | [T/C] | Ca15495 | Intron | DNA topoisomerase I,C-terminal |
| CWSNP3433 | Ca-Kabuli-Chr4 | 32853838 | [G/T] | Ca15495 | Intron | DNA topoisomerase I,C-terminal |
| CWSNP3434 | Ca-Kabuli-Chr4 | 33509759 | [A/C] | - | Intergenic | - |
| CWSNP3435 | Ca-Kabuli-Chr4 | 33509762 | [G/A] | - | Intergenic | - |
| CWSNP3436 | Ca-Kabuli-Chr4 | 33641757 | [C/T] | Ca24030 | Intron | SNF2-related |
| CWSNP3437 | Ca-Kabuli-Chr4 | 33820657 | [A/C] | - | DRR | - |
| CWSNP3438 | Ca-Kabuli-Chr4 | 33874346 | [T/C] | - | Intergenic | - |
| CWSNP3439 | Ca-Kabuli-Chr4 | 33874327 | [T/C] | - | Intergenic | - |
| CWSNP3440 | Ca-Kabuli-Chr4 | 33874313 | [G/A] | - | Intergenic | - |

| SNP IDs | Chromosomes | Physical positions (bp) | SNPs | Gene accession IDs | Sequence components of genes | Putative functions |
|---|---|---|---|---|---|---|
| CWSNP3441 | Ca-Kabuli-Chr4 | 33929777 | [C/T] | Ca20365 | Intron | Peptidase C19,ubiquitin carboxyl-terminalhydrolase 2 |
| CWSNP3442 | Ca-Kabuli-Chr4 | 34155084 | [C/G] | Ca20360 | Synonymous-CDS | PeptidaseC48,SUMO/Sentrin/Ubl1 |
| CWSNP3443 | Ca-Kabuli-Chr4 | 34222792 | [G/C] | - | Intergenic | - |
| CWSNP3444 | Ca-Kabuli-Chr4 | 34354519 | [G/T] | - | Intergenic | - |
| CWSNP3445 | Ca-Kabuli-Chr4 | 34487064 | [T/G] | Ca20350 | Intron | Domain of unknown function DUF676,hydrolase-like |
| CWSNP3446 | Ca-Kabuli-Chr4 | 34784285 | [G/A] | Ca19990 | Non-Synonymous-CDS | Ribosomal proteinL22/L17 |
| CWSNP3447 | Ca-Kabuli-Chr4 | 34798597 | [C/A] | Ca19992 | Intron | Pentatrico peptide repeat |
| CWSNP3448 | Ca-Kabuli-Chr4 | 34916032 | [C/T] | Ca19997 | Intron | Inosine/uridine-preferring nucleosidehydrolase domain |
| CWSNP3449 | Ca-Kabuli-Chr4 | 34924575 | [C/T] | Ca19998 | Non-Synonymous-CDS | Metallo-dependent phosphatase |
| CWSNP3450 | Ca-Kabuli-Chr4 | 34955953 | [A/G] | Ca19999 | Non-Synonymous-CDS | PeptidaseC48,SUMO/Sentrin/Ubl1 |
| CWSNP3451 | Ca-Kabuli-Chr4 | 34956006 | [C/T] | Ca19999 | Synonymous-CDS | PeptidaseC48,SUMO/Sentrin/Ubl1 |
| CWSNP3452 | Ca-Kabuli-Chr4 | 34956035 | [C/T] | Ca19999 | Non-Synonymous-CDS | PeptidaseC48,SUMO/Sentrin/Ubl1 |
| CWSNP3453 | Ca-Kabuli-Chr4 | 34956031 | [G/A] | Ca19999 | Non-Synonymous-CDS | PeptidaseC48,SUMO/Sentrin/Ubl1 |
| CWSNP3454 | Ca-Kabuli-Chr4 | 35141281 | [A/T] | Ca20010 | Non-Synonymous-CDS | Glycosidehydrolase,family9 |
| CWSNP3455 | Ca-Kabuli-Chr4 | 35141309 | [A/C] | Ca20010 | Synonymous-CDS | Glycosidehydrolase,family9 |
| CWSNP3456 | Ca-Kabuli-Chr4 | 35141340 | [G/A] | Ca20010 | Intron | Glycosidehydrolase,family9 |

| SNP IDs | Chromosomes | Physical positions (bp) | SNPs | Gene accession IDs | Sequence components of genes | Putative functions |
|---|---|---|---|---|---|---|
| CWSNP3457 | Ca-Kabuli-Chr4 | 35141353 | [C/G] | Ca20010 | Intron | Glycosidehydrolase,family9 |
| CWSNP3458 | Ca-Kabuli-Chr4 | 35141359 | [C/G] | Ca20010 | Intron | Glycosidehydrolase,family9 |
| CWSNP3459 | Ca-Kabuli-Chr4 | 35141375 | [A/G] | Ca20010 | Intron | Glycosidehydrolase,family9 |
| CWSNP3460 | Ca-Kabuli-Chr4 | 35141403 | [C/T] | Ca20010 | Intron | Glycosidehydrolase,family9 |
| CWSNP3461 | Ca-Kabuli-Chr4 | 35168915 | [C/T] | Ca20011 | Intron | - |
| CWSNP3462 | Ca-Kabuli-Chr4 | 35168939 | [C/T] | Ca20011 | Intron | - |
| CWSNP3463 | Ca-Kabuli-Chr4 | 35229966 | [C/T] | Ca20015 | Non-Synonymous-CDS | - |
| CWSNP3464 | Ca-Kabuli-Chr4 | 35231891 | [C/T] | - | Intergenic | - |
| CWSNP3465 | Ca-Kabuli-Chr4 | 35304102 | [C/T] | Ca20017 | Synonymous-CDS | - |
| CWSNP3466 | Ca-Kabuli-Chr4 | 35304108 | [C/A] | Ca20017 | Non-Synonymous-CDS | - |
| CWSNP3467 | Ca-Kabuli-Chr4 | 35309709 | [A/G] | Ca20017 | Intron | - |
| CWSNP3468 | Ca-Kabuli-Chr4 | 35311815 | [A/C] | Ca20017 | Synonymous-CDS | - |
| CWSNP3469 | Ca-Kabuli-Chr4 | 35337282 | [A/G] | Ca20018 | Intron | IQ motif, EF-hand binding site |
| CWSNP3470 | Ca-Kabuli-Chr4 | 35344356 | [A/G] | Ca20018 | Intron | IQ motif, EF-hand binding site |
| CWSNP3471 | Ca-Kabuli-Chr4 | 35344353 | [T/C] | Ca20018 | Intron | IQ motif, EF-hand binding site |
| CWSNP3472 | Ca-Kabuli-Chr4 | 35344323 | [C/T] | Ca20018 | Intron | IQ motif, EF-hand binding site |

| SNP IDs | Chromosomes | Physical positions (bp) | SNPs | Gene accession IDs | Sequence components of genes | Putative functions |
|---|---|---|---|---|---|---|
| CWSNP3473 | Ca-Kabuli-Chr4 | 35346417 | [C/T] | Ca20018 | Intron | IQ motif, EF-hand binding site |
| CWSNP3474 | Ca-Kabuli-Chr4 | 35346465 | [G/T] | Ca20018 | Intron | IQ motif, EF-hand binding site |
| CWSNP3475 | Ca-Kabuli-Chr4 | 35346558 | [G/T] | Ca20018 | Intron | IQ motif, EF-hand binding site |
| CWSNP3476 | Ca-Kabuli-Chr4 | 35346563 | [G/T] | Ca20018 | Intron | IQ motif, EF-hand binding site |
| CWSNP3477 | Ca-Kabuli-Chr4 | 35346576 | [C/G] | Ca20018 | Intron | IQ motif, EF-hand binding site |
| CWSNP3478 | Ca-Kabuli-Chr4 | 35346624 | [G/A] | Ca20018 | Intron | IQ motif, EF-hand binding site |
| CWSNP3479 | Ca-Kabuli-Chr4 | 35393505 | [G/T] | Ca20021 | Synonymous-CDS | Transcription factor,TCP |
| CWSNP3480 | Ca-Kabuli-Chr4 | 35393643 | [G/A] | Ca20021 | Synonymous-CDS | Transcription factor,TCP |
| CWSNP3481 | Ca-Kabuli-Chr4 | 35455095 | [A/C] | - | Intergenic | - |
| CWSNP3482 | Ca-Kabuli-Chr4 | 35455062 | [A/T] | - | Intergenic | - |
| CWSNP3483 | Ca-Kabuli-Chr4 | 35455708 | [G/A] | - | Intergenic | - |
| CWSNP3484 | Ca-Kabuli-Chr4 | 35469308 | [T/G] | Ca22431 | Non-Synonymous-CDS | Sugar/inositol transporter |
| CWSNP3485 | Ca-Kabuli-Chr4 | 35469304 | [G/A] | Ca22431 | Non-Synonymous-CDS | Sugar/inositol transporter |
| CWSNP3486 | Ca-Kabuli-Chr4 | 35487504 | [G/C] | - | URR | - |
| CWSNP3487 | Ca-Kabuli-Chr4 | 35487496 | [C/A] | - | URR | - |
| CWSNP3488 | Ca-Kabuli-Chr4 | 35487471 | [A/C] | - | URR | - |

| SNP IDs | Chromosomes | Physical positions (bp) | SNPs | Gene accession IDs | Sequence components of genes | Putative functions |
|---|---|---|---|---|---|---|
| CWSNP3489 | Ca-Kabuli-Chr4 | 35528758 | [C/A] | Ca22434 | Intron | - |
| CWSNP3490 | Ca-Kabuli-Chr4 | 35534777 | [G/C] | Ca22434 | Intron | - |
| CWSNP3491 | Ca-Kabuli-Chr4 | 35534877 | [T/C] | Ca22434 | Intron | - |
| CWSNP3492 | Ca-Kabuli-Chr4 | 35534891 | [C/T] | Ca22434 | Intron | - |
| CWSNP3493 | Ca-Kabuli-Chr4 | 35634712 | [A/G] | - | Intergenic | - |
| CWSNP3494 | Ca-Kabuli-Chr4 | 35634751 | [A/G] | - | Intergenic | - |
| CWSNP3495 | Ca-Kabuli-Chr4 | 35634873 | [G/T] | - | Intergenic | - |
| CWSNP3496 | Ca-Kabuli-Chr4 | 35961593 | [A/C] | - | URR | - |
| CWSNP3497 | Ca-Kabuli-Chr4 | 35977422 | [G/A] | - | URR | - |
| CWSNP3498 | Ca-Kabuli-Chr4 | 36005847 | [T/G] | - | Intergenic | - |
| CWSNP3499 | Ca-Kabuli-Chr4 | 36010756 | [T/G] | - | Intergenic | - |
| CWSNP3500 | Ca-Kabuli-Chr4 | 36277383 | [C/T] | Ca14843 | Non-Synonymous-CDS | Helicase,C-terminal |
| CWSNP3501 | Ca-Kabuli-Chr4 | 36445345 | [G/A] | Ca14833 | Synonymous-CDS | Armadillo |
| CWSNP3502 | Ca-Kabuli-Chr4 | 36445367 | [A/G] | Ca14833 | Non-Synonymous-CDS | Armadillo |
| CWSNP3503 | Ca-Kabuli-Chr4 | 36449101 | [C/T] | Ca14833 | Intron | Armadillo |
| CWSNP3504 | Ca-Kabuli-Chr4 | 36461836 | [A/G] | - | DRR | - |

| SNP IDs | Chromosomes | Physical positions (bp) | SNPs | Gene accession IDs | Sequence components of genes | Putative functions |
|---|---|---|---|---|---|---|
| CWSNP3505 | Ca-Kabuli-Chr4 | 36467928 | [C/T] | - | URR | - |
| CWSNP3506 | Ca-Kabuli-Chr4 | 36467943 | [A/G] | - | URR | - |
| CWSNP3507 | Ca-Kabuli-Chr4 | 36467964 | [G/A] | - | URR | - |
| CWSNP3508 | Ca-Kabuli-Chr4 | 36467967 | [T/C] | - | URR | - |
| CWSNP3509 | Ca-Kabuli-Chr4 | 36467980 | [C/A] | - | URR | - |
| CWSNP3510 | Ca-Kabuli-Chr4 | 36467988 | [C/T] | - | URR | - |
| CWSNP3511 | Ca-Kabuli-Chr4 | 36467990 | [T/G] | - | URR | - |
| CWSNP3512 | Ca-Kabuli-Chr4 | 36468003 | [T/A] | - | URR | - |
| CWSNP3513 | Ca-Kabuli-Chr4 | 36468053 | [A/G] | - | URR | - |
| CWSNP3514 | Ca-Kabuli-Chr4 | 36537118 | [C/T] | Ca14825 | Synonymous-CDS | Transcriptional factor B3 |
| CWSNP3515 | Ca-Kabuli-Chr4 | 36537124 | [C/T] | Ca14825 | Synonymous-CDS | Transcriptional factor B3 |
| CWSNP3516 | Ca-Kabuli-Chr4 | 36537170 | [C/T] | Ca14825 | Non-Synonymous-CDS | Transcriptional factor B3 |
| CWSNP3517 | Ca-Kabuli-Chr4 | 36541088 | [G/T] | - | DRR | - |
| CWSNP3518 | Ca-Kabuli-Chr4 | 36541087 | [T/C] | - | DRR | - |
| CWSNP3519 | Ca-Kabuli-Chr4 | 36541083 | [A/C] | - | DRR | - |
| CWSNP3520 | Ca-Kabuli-Chr4 | 36639333 | [A/T] | - | Intergenic | - |

| SNP IDs | Chromosomes | Physical positions (bp) | SNPs | Gene accession IDs | Sequence components of genes | Putative functions |
|---|---|---|---|---|---|---|
| CWSNP3521 | Ca-Kabuli-Chr4 | 36639382 | [T/C] | - | Intergenic | - |
| CWSNP3522 | Ca-Kabuli-Chr4 | 36673677 | [C/A] | - | Intergenic | - |
| CWSNP3523 | Ca-Kabuli-Chr4 | 36682215 | [G/A] | Ca14812 | Non-Synonymous-CDS | - |
| CWSNP3524 | Ca-Kabuli-Chr4 | 36682213 | [C/T] | Ca14812 | Non-Synonymous-CDS | - |
| CWSNP3525 | Ca-Kabuli-Chr4 | 36682211 | [G/A] | Ca14812 | Synonymous-CDS | - |
| CWSNP3526 | Ca-Kabuli-Chr4 | 36682253 | [T/G] | Ca14812 | Synonymous-CDS | - |
| CWSNP3527 | Ca-Kabuli-Chr4 | 36682231 | [G/T] | Ca14812 | Non-Synonymous-CDS | - |
| CWSNP3528 | Ca-Kabuli-Chr4 | 36709731 | [G/A] | - | DRR | - |
| CWSNP3529 | Ca-Kabuli-Chr4 | 36754121 | [T/C] | Ca14808 | Intron | Phosphofructokinase domain |
| CWSNP3530 | Ca-Kabuli-Chr4 | 36754290 | [T/A] | Ca14808 | Intron | Phosphofructokinase domain |
| CWSNP3531 | Ca-Kabuli-Chr4 | 36754378 | [C/T] | Ca14808 | Intron | Phosphofructokinase domain |
| CWSNP3532 | Ca-Kabuli-Chr4 | 36754377 | [T/A] | Ca14808 | Intron | Phosphofructokinase domain |
| CWSNP3533 | Ca-Kabuli-Chr4 | 36754348 | [C/T] | Ca14808 | Intron | Phosphofructokinase domain |
| CWSNP3534 | Ca-Kabuli-Chr4 | 36754343 | [A/C] | Ca14808 | Intron | Phosphofructokinase domain |
| CWSNP3535 | Ca-Kabuli-Chr4 | 36754327 | [G/A] | Ca14808 | Intron | Phosphofructokinase domain |
| CWSNP3536 | Ca-Kabuli-Chr4 | 36754468 | [G/A] | Ca14808 | Synonymous-CDS | Phosphofructokinase domain |

| SNP IDs | Chromosomes | Physical positions (bp) | SNPs | Gene accession IDs | Sequence components of genes | Putative functions |
|---|---|---|---|---|---|---|
| CWSNP3537 | Ca-Kabuli-Chr4 | 36754453 | [A/G] | Ca14808 | Synonymous-CDS | Phosphofructokinase domain |
| CWSNP3538 | Ca-Kabuli-Chr4 | 36799551 | [C/T] | Ca14807 | Synonymous-CDS | RNA recognition motif domain |
| CWSNP3539 | Ca-Kabuli-Chr4 | 36831199 | [G/A] | Ca14805 | Non-Synonymous-CDS | - |
| CWSNP3540 | Ca-Kabuli-Chr4 | 36831267 | [A/C] | Ca14805 | Synonymous-CDS | - |
| CWSNP3541 | Ca-Kabuli-Chr4 | 36955706 | [G/T] | Ca14797 | Non-Synonymous-CDS | Zinc finger,C2H2-type |
| CWSNP3542 | Ca-Kabuli-Chr4 | 36977192 | [C/T] | - | Intergenic | - |
| CWSNP3543 | Ca-Kabuli-Chr4 | 36977194 | [G/A] | - | Intergenic | - |
| CWSNP3544 | Ca-Kabuli-Chr4 | 36979926 | [G/T] | - | Intergenic | - |
| CWSNP3545 | Ca-Kabuli-Chr4 | 36979879 | [C/T] | - | Intergenic | - |
| CWSNP3546 | Ca-Kabuli-Chr4 | 36984727 | [C/G] | Ca14795 | Intron | SNF2-related |
| CWSNP3547 | Ca-Kabuli-Chr4 | 36984795 | [T/A] | Ca14795 | Intron | SNF2-related |
| CWSNP3548 | Ca-Kabuli-Chr4 | 36998201 | [A/C] | - | Intergenic | - |
| CWSNP3549 | Ca-Kabuli-Chr4 | 37064588 | [A/G] | - | Intergenic | - |
| CWSNP3550 | Ca-Kabuli-Chr4 | 37064630 | [G/A] | - | Intergenic | - |
| CWSNP3551 | Ca-Kabuli-Chr4 | 37185576 | [T/A] | Ca15153 | Intron | RNA-dependent RNA polymerase,eukaryotic-type |
| CWSNP3552 | Ca-Kabuli-Chr4 | 37185577 | [T/A] | Ca15153 | Intron | RNA-dependent RNA polymerase,eukaryotic-type |

| SNP IDs | Chromosomes | Physical positions (bp) | SNPs | Gene accession IDs | Sequence components of genes | Putative functions |
|---|---|---|---|---|---|---|
| CWSNP3553 | Ca-Kabuli-Chr4 | 37185578 | [T/A] | Ca15153 | Intron | RNA-dependent RNA polymerase,eukaryotic-type |
| CWSNP3554 | Ca-Kabuli-Chr4 | 37237211 | [G/A] | - | Intergenic | - |
| CWSNP3555 | Ca-Kabuli-Chr4 | 37237276 | [T/C] | - | Intergenic | - |
| CWSNP3556 | Ca-Kabuli-Chr4 | 37241235 | [A/G] | Ca15151 | Synonymous-CDS | Signal transduction response regulator,receiver domain |
| CWSNP3557 | Ca-Kabuli-Chr4 | 37241262 | [G/A] | Ca15151 | Synonymous-CDS | Signal transduction response regulator,receiver domain |
| CWSNP3558 | Ca-Kabuli-Chr4 | 37260873 | [A/G] | Ca15149 | Synonymous-CDS | Dynamin central domain |
| CWSNP3559 | Ca-Kabuli-Chr4 | 37260841 | [T/A] | Ca15149 | Intron | Dynamin central domain |
| CWSNP3560 | Ca-Kabuli-Chr4 | 37264628 | [G/A] | Ca15149 | Intron | Dynamin central domain |
| CWSNP3561 | Ca-Kabuli-Chr4 | 37286591 | [T/A] | Ca15147 | Intron | - |
| CWSNP3562 | Ca-Kabuli-Chr4 | 37286625 | [C/A] | Ca15147 | Intron | - |
| CWSNP3563 | Ca-Kabuli-Chr4 | 37286632 | [G/A] | Ca15147 | Intron | - |
| CWSNP3564 | Ca-Kabuli-Chr4 | 37286663 | [A/G] | Ca15147 | Intron | - |
| CWSNP3565 | Ca-Kabuli-Chr4 | 37293752 | [G/A] | - | Intergenic | - |
| CWSNP3566 | Ca-Kabuli-Chr4 | 37293780 | [T/A] | - | Intergenic | - |
| CWSNP3567 | Ca-Kabuli-Chr4 | 37293773 | [A/T] | - | Intergenic | - |
| CWSNP3568 | Ca-Kabuli-Chr4 | 37293769 | [C/A] | - | Intergenic | - |

| SNP IDs | Chromosomes | Physical positions (bp) | SNPs | Gene accession IDs | Sequence components of genes | Putative functions |
|---|---|---|---|---|---|---|
| CWSNP3569 | Ca-Kabuli-Chr4 | 37294720 | [A/G] | - | Intergenic | - |
| CWSNP3570 | Ca-Kabuli-Chr4 | 37316574 | [T/A] | Ca15144 | Synonymous-CDS | Zinc finger,RING-type |
| CWSNP3571 | Ca-Kabuli-Chr4 | 37316748 | [T/A] | Ca15144 | Synonymous-CDS | Zinc finger,RING-type |
| CWSNP3572 | Ca-Kabuli-Chr4 | 37316738 | [C/T] | Ca15144 | Non-Synonymous-CDS | Zinc finger,RING-type |
| CWSNP3573 | Ca-Kabuli-Chr4 | 37316713 | [A/T] | Ca15144 | Non-Synonymous-CDS | Zinc finger,RING-type |
| CWSNP3574 | Ca-Kabuli-Chr4 | 37316700 | [C/A] | Ca15144 | Non-Synonymous-CDS | Zinc finger,RING-type |
| CWSNP3575 | Ca-Kabuli-Chr4 | 37349071 | [T/C] | Ca15141 | Intron | MethyltransferaseTRM13 |
| CWSNP3576 | Ca-Kabuli-Chr4 | 37349212 | [A/C] | Ca15141 | Synonymous-CDS | MethyltransferaseTRM13 |
| CWSNP3577 | Ca-Kabuli-Chr4 | 37349239 | [C/G] | Ca15141 | Intron | MethyltransferaseTRM13 |
| CWSNP3578 | Ca-Kabuli-Chr4 | 37349304 | [T/C] | Ca15141 | Intron | MethyltransferaseTRM13 |
| CWSNP3579 | Ca-Kabuli-Chr4 | 37349331 | [C/T] | Ca15141 | Intron | MethyltransferaseTRM13 |
| CWSNP3580 | Ca-Kabuli-Chr4 | 37349321 | [T/C] | Ca15141 | Intron | MethyltransferaseTRM13 |
| CWSNP3581 | Ca-Kabuli-Chr4 | 37356865 | [C/A] | Ca15140 | Synonymous-CDS | HEAT |
| CWSNP3582 | Ca-Kabuli-Chr4 | 37356889 | [C/T] | Ca15140 | Intron | HEAT |
| CWSNP3583 | Ca-Kabuli-Chr4 | 37356892 | [C/T] | Ca15140 | Intron | HEAT |
| CWSNP3584 | Ca-Kabuli-Chr4 | 37356919 | [A/T] | Ca15140 | Intron | HEAT |

| SNP IDs | Chromosomes | Physical positions (bp) | SNPs | Gene accession IDs | Sequence components of genes | Putative functions |
|---|---|---|---|---|---|---|
| CWSNP3585 | Ca-Kabuli-Chr4 | 37356951 | [C/T] | Ca15140 | Intron | HEAT |
| CWSNP3586 | Ca-Kabuli-Chr4 | 37364980 | [G/A] | Ca15140 | Synonymous-CDS | HEAT |
| CWSNP3587 | Ca-Kabuli-Chr4 | 37371262 | [G/A] | Ca15139 | Synonymous-CDS | Ankyrin repeat |
| CWSNP3588 | Ca-Kabuli-Chr4 | 37371263 | [G/A] | Ca15139 | Non-Synonymous-CDS | Ankyrin repeat |
| CWSNP3589 | Ca-Kabuli-Chr4 | 37433459 | [G/T] | - | Intergenic | - |
| CWSNP3590 | Ca-Kabuli-Chr4 | 37450206 | [A/C] | Ca15131 | Synonymous-CDS | Oxoglutarate/iron-dependent oxygenase |
| CWSNP3591 | Ca-Kabuli-Chr4 | 37450326 | [A/T] | Ca15131 | Synonymous-CDS | Oxoglutarate/iron-dependent oxygenase |
| CWSNP3592 | Ca-Kabuli-Chr4 | 37450294 | [T/C] | Ca15131 | Synonymous-CDS | Oxoglutarate/iron-dependent oxygenase |
| CWSNP3593 | Ca-Kabuli-Chr4 | 37453497 | [C/G] | - | URR | - |
| CWSNP3594 | Ca-Kabuli-Chr4 | 37453449 | [A/G] | - | URR | - |
| CWSNP3595 | Ca-Kabuli-Chr4 | 37453590 | [C/A] | - | URR | - |
| CWSNP3596 | Ca-Kabuli-Chr4 | 37453586 | [C/A] | - | URR | - |
| CWSNP3597 | Ca-Kabuli-Chr4 | 37454296 | [A/G] | - | URR | - |
| CWSNP3598 | Ca-Kabuli-Chr4 | 37492422 | [C/A] | - | Intergenic | - |
| CWSNP3599 | Ca-Kabuli-Chr4 | 37549883 | [T/C] | Ca15125 | Intron | Mediator complex subunit Med23 |
| CWSNP3600 | Ca-Kabuli-Chr4 | 37549871 | [A/T] | Ca15125 | Intron | Mediator complex subunit Med23 |

| SNP IDs | Chromosomes | Physical positions (bp) | SNPs | Gene accession IDs | Sequence components of genes | Putative functions |
|---|---|---|---|---|---|---|
| CWSNP3601 | Ca-Kabuli-Chr4 | 37550122 | [G/A] | Ca15125 | Intron | Mediator complex subunit Med23 |
| CWSNP3602 | Ca-Kabuli-Chr4 | 37552973 | [C/T] | Ca15125 | Intron | Mediator complex subunit Med23 |
| CWSNP3603 | Ca-Kabuli-Chr4 | 37558880 | [C/T] | Ca15125 | Intron | Mediator complex subunit Med23 |
| CWSNP3604 | Ca-Kabuli-Chr4 | 37558881 | [G/T] | Ca15125 | Intron | Mediator complex subunit Med23 |
| CWSNP3605 | Ca-Kabuli-Chr4 | 37575636 | [T/C] | Ca15125 | Intron | Mediator complex subunit Med23 |
| CWSNP3606 | Ca-Kabuli-Chr4 | 37575670 | [C/T] | Ca15125 | Intron | Mediator complex subunit Med23 |
| CWSNP3607 | Ca-Kabuli-Chr4 | 37575709 | [C/T] | Ca15125 | Intron | Mediator complex subunit Med23 |
| CWSNP3608 | Ca-Kabuli-Chr4 | 37612154 | [A/G] | Ca15121 | Synonymous-CDS | Glycosidehydrolase,family17 |
| CWSNP3609 | Ca-Kabuli-Chr4 | 37630404 | [A/G] | - | DRR | - |
| CWSNP3610 | Ca-Kabuli-Chr4 | 37630445 | [A/G] | - | DRR | - |
| CWSNP3611 | Ca-Kabuli-Chr4 | 37630527 | [G/A] | - | DRR | - |
| CWSNP3612 | Ca-Kabuli-Chr4 | 37630520 | [G/C] | - | DRR | - |
| CWSNP3613 | Ca-Kabuli-Chr4 | 37635300 | [T/A] | - | Intergenic | - |
| CWSNP3614 | Ca-Kabuli-Chr4 | 37685824 | [T/C] | Ca15113 | Synonymous-CDS | Armadillo |
| CWSNP3615 | Ca-Kabuli-Chr4 | 37685842 | [T/G] | Ca15113 | Synonymous-CDS | Armadillo |
| CWSNP3616 | Ca-Kabuli-Chr4 | 37699176 | [G/A] | Ca15111 | Synonymous-CDS | ATP-graspfold |

| SNP IDs | Chromosomes | Physical positions (bp) | SNPs | Gene accession IDs | Sequence components of genes | Putative functions |
|---|---|---|---|---|---|---|
| CWSNP3617 | Ca-Kabuli-Chr4 | 37699141 | [T/G] | Ca15111 | Synonymous-CDS | ATP-graspfold |
| CWSNP3618 | Ca-Kabuli-Chr4 | 37699140 | [C/T] | Ca15111 | Synonymous-CDS | ATP-graspfold |
| CWSNP3619 | Ca-Kabuli-Chr4 | 37703653 | [A/C] | - | Intergenic | - |
| CWSNP3620 | Ca-Kabuli-Chr4 | 37703649 | [A/C] | - | Intergenic | - |
| CWSNP3621 | Ca-Kabuli-Chr4 | 37708260 | [A/G] | - | DRR | - |
| CWSNP3622 | Ca-Kabuli-Chr4 | 37708348 | [T/G] | - | DRR | - |
| CWSNP3623 | Ca-Kabuli-Chr4 | 37708334 | [T/A] | - | DRR | - |
| CWSNP3624 | Ca-Kabuli-Chr4 | 37753479 | [C/T] | Ca15104 | Intron | - |
| CWSNP3625 | Ca-Kabuli-Chr4 | 37806689 | [T/C] | - | DRR | - |
| CWSNP3626 | Ca-Kabuli-Chr4 | 37812917 | [G/C] | - | Intergenic | - |
| CWSNP3627 | Ca-Kabuli-Chr4 | 37859069 | [T/G] | Ca15089 | Synonymous-CDS | Auxin efflux carrier |
| CWSNP3628 | Ca-Kabuli-Chr4 | 37897261 | [G/T] | - | Intergenic | - |
| CWSNP3629 | Ca-Kabuli-Chr4 | 37897266 | [A/T] | - | Intergenic | - |
| CWSNP3630 | Ca-Kabuli-Chr4 | 37897351 | [C/A] | - | Intergenic | - |
| CWSNP3631 | Ca-Kabuli-Chr4 | 37898404 | [A/G] | - | Intergenic | - |
| CWSNP3632 | Ca-Kabuli-Chr4 | 37901584 | [C/T] | - | Intergenic | - |

| SNP IDs | Chromosomes | Physical positions (bp) | SNPs | Gene accession IDs | Sequence components of genes | Putative functions |
|---|---|---|---|---|---|---|
| CWSNP3633 | Ca-Kabuli-Chr4 | 38008062 | [T/A] | - | DRR | - |
| CWSNP3634 | Ca-Kabuli-Chr4 | 38008082 | [T/C] | - | DRR | - |
| CWSNP3635 | Ca-Kabuli-Chr4 | 38008243 | [C/T] | - | DRR | - |
| CWSNP3636 | Ca-Kabuli-Chr4 | 38008227 | [G/T] | - | DRR | - |
| CWSNP3637 | Ca-Kabuli-Chr4 | 38008213 | [C/T] | - | DRR | - |
| CWSNP3638 | Ca-Kabuli-Chr4 | 38008205 | [A/G] | - | DRR | - |
| CWSNP3639 | Ca-Kabuli-Chr4 | 38008204 | [G/A] | - | DRR | - |
| CWSNP3640 | Ca-Kabuli-Chr4 | 38008186 | [A/C] | - | DRR | - |
| CWSNP3641 | Ca-Kabuli-Chr4 | 38008174 | [A/G] | - | DRR | - |
| CWSNP3642 | Ca-Kabuli-Chr4 | 38029136 | [G/T] | Ca13142 | Non-Synonymous-CDS | ChaperoninTCP-1,conserved site |
| CWSNP3643 | Ca-Kabuli-Chr4 | 38038865 | [C/T] | Ca13141 | Non-Synonymous-CDS | Protein of unknown function DUF3326 |
| CWSNP3644 | Ca-Kabuli-Chr4 | 38038959 | [A/G] | Ca13141 | Intron | Protein of unknown function DUF3326 |
| CWSNP3645 | Ca-Kabuli-Chr4 | 38039037 | [T/C] | Ca13141 | Intron | Protein of unknown function DUF3326 |
| CWSNP3646 | Ca-Kabuli-Chr4 | 38039046 | [C/T] | Ca13141 | Intron | Protein of unknown function DUF3326 |
| CWSNP3647 | Ca-Kabuli-Chr4 | 38039076 | [A/G] | Ca13141 | Intron | Protein of unknown function DUF3326 |
| CWSNP3648 | Ca-Kabuli-Chr4 | 38039088 | [T/C] | Ca13141 | Intron | Protein of unknown function DUF3326 |

| SNP IDs | Chromosomes | Physical positions (bp) | SNPs | Gene accession IDs | Sequence components of genes | Putative functions |
|---|---|---|---|---|---|---|
| CWSNP3649 | Ca-Kabuli-Chr4 | 38039251 | [G/C] | Ca13141 | Intron | Protein of unknown function DUF3326 |
| CWSNP3650 | Ca-Kabuli-Chr4 | 38039289 | [G/A] | Ca13141 | Intron | Protein of unknown function DUF3326 |
| CWSNP3651 | Ca-Kabuli-Chr4 | 38039297 | [C/T] | Ca13141 | Non-Synonymous-CDS | Protein of unknown function DUF3326 |
| CWSNP3652 | Ca-Kabuli-Chr4 | 38153183 | [C/A] | Ca13138 | Synonymous-CDS | Tetrahydrofolate dehydrogenase/cyclohydrolase |
| CWSNP3653 | Ca-Kabuli-Chr4 | 38153195 | [T/C] | Ca13138 | Synonymous-CDS | Tetrahydrofolate dehydrogenase/cyclohydrolase |
| CWSNP3654 | Ca-Kabuli-Chr4 | 38153216 | [T/C] | Ca13138 | Synonymous-CDS | Tetrahydrofolate dehydrogenase/cyclohydrolase |
| CWSNP3655 | Ca-Kabuli-Chr4 | 38153277 | [A/G] | Ca13138 | Intron | Tetrahydrofolate dehydrogenase/cyclohydrolase |
| CWSNP3656 | Ca-Kabuli-Chr4 | 38153275 | [C/A] | Ca13138 | Intron | Tetrahydrofolate dehydrogenase/cyclohydrolase |
| CWSNP3657 | Ca-Kabuli-Chr4 | 38179583 | [A/C] | - | Intergenic | - |
| CWSNP3658 | Ca-Kabuli-Chr4 | 38179614 | [G/C] | - | Intergenic | - |
| CWSNP3659 | Ca-Kabuli-Chr4 | 38181292 | [G/A] | Ca13135 | Non-Synonymous-CDS | - |
| CWSNP3660 | Ca-Kabuli-Chr4 | 38195411 | [T/A] | Ca13134 | Non-Synonymous-CDS | Domain of unknown function DUF250 |
| CWSNP3661 | Ca-Kabuli-Chr4 | 38195438 | [C/T] | Ca13134 | Non-Synonymous-CDS | Domain of unknown function DUF250 |
| CWSNP3662 | Ca-Kabuli-Chr4 | 38215267 | [G/A] | Ca13132 | Intron | AMP-dependent synthetase/ligase |
| CWSNP3663 | Ca-Kabuli-Chr4 | 38221009 | [A/G] | Ca13131 | Synonymous-CDS | Lipase,GDSL |
| CWSNP3664 | Ca-Kabuli-Chr4 | 38288823 | [G/T] | Ca13128 | Synonymous-CDS | Protein of unknown function DUF869,plant |

| SNP IDs | Chromosomes | Physical positions (bp) | SNPs | Gene accession IDs | Sequence components of genes | Putative functions |
|---|---|---|---|---|---|---|
| CWSNP3665 | Ca-Kabuli-Chr4 | 38288877 | [C/G] | Ca13128 | Non-Synonymous-CDS | Protein of unknown function DUF869,plant |
| CWSNP3666 | Ca-Kabuli-Chr4 | 38289066 | [G/A] | Ca13128 | Synonymous-CDS | Protein of unknown function DUF869,plant |
| CWSNP3667 | Ca-Kabuli-Chr4 | 38289052 | [G/A] | Ca13128 | Synonymous-CDS | Protein of unknown function DUF869,plant |
| CWSNP3668 | Ca-Kabuli-Chr4 | 38288997 | [T/A] | Ca13128 | Synonymous-CDS | Protein of unknown function DUF869,plant |
| CWSNP3669 | Ca-Kabuli-Chr4 | 38343633 | [A/T] | - | Intergenic | - |
| CWSNP3670 | Ca-Kabuli-Chr4 | 38343750 | [C/T] | - | Intergenic | - |
| CWSNP3671 | Ca-Kabuli-Chr4 | 38343687 | [C/T] | - | Intergenic | - |
| CWSNP3672 | Ca-Kabuli-Chr4 | 38358067 | [A/G] | Ca13120 | Non-Synonymous-CDS | C2 calcium-dependent membrane targeting |
| CWSNP3673 | Ca-Kabuli-Chr4 | 38358398 | [A/G] | Ca13120 | Synonymous-CDS | C2 calcium-dependent membrane targeting |
| CWSNP3674 | Ca-Kabuli-Chr4 | 38358677 | [G/A] | Ca13120 | Synonymous-CDS | C2 calcium-dependent membrane targeting |
| CWSNP3675 | Ca-Kabuli-Chr4 | 38358887 | [G/A] | Ca13120 | Synonymous-CDS | C2 calcium-dependent membrane targeting |
| CWSNP3676 | Ca-Kabuli-Chr4 | 38362132 | [T/A] | Ca13120 | Synonymous-CDS | C2 calcium-dependent membrane targeting |
| CWSNP3677 | Ca-Kabuli-Chr4 | 38370848 | [A/G] | - | DRR | - |
| CWSNP3678 | Ca-Kabuli-Chr4 | 38370864 | [G/A] | - | DRR | - |
| CWSNP3679 | Ca-Kabuli-Chr4 | 38370941 | [A/G] | - | DRR | - |
| CWSNP3680 | Ca-Kabuli-Chr4 | 38370926 | [T/C] | - | DRR | - |

| SNP IDs | Chromosomes | Physical positions (bp) | SNPs | Gene accession IDs | Sequence components of genes | Putative functions |
|---|---|---|---|---|---|---|
| CWSNP3681 | Ca-Kabuli-Chr4 | 38370925 | [G/A] | - | DRR | - |
| CWSNP3682 | Ca-Kabuli-Chr4 | 38370921 | [T/G] | - | DRR | - |
| CWSNP3683 | Ca-Kabuli-Chr4 | 38382890 | [C/G] | Ca13119 | Synonymous-CDS | Amino acid transporter,transmembrane |
| CWSNP3684 | Ca-Kabuli-Chr4 | 38421798 | [A/C] | Ca13115 | Intron | Zinc finger,CCCH-type |
| CWSNP3685 | Ca-Kabuli-Chr4 | 38422037 | [C/T] | Ca13115 | Intron | Zinc finger,CCCH-type |
| CWSNP3686 | Ca-Kabuli-Chr4 | 38425675 | [A/G] | Ca13115 | Synonymous-CDS | Zinc finger,CCCH-type |
| CWSNP3687 | Ca-Kabuli-Chr4 | 38425720 | [T/A] | Ca13115 | Non-Synonymous-CDS | Zinc finger,CCCH-type |
| CWSNP3688 | Ca-Kabuli-Chr4 | 38473653 | [T/C] | Ca13112 | Intron | - |
| CWSNP3689 | Ca-Kabuli-Chr4 | 38474123 | [A/T] | Ca13112 | Intron | - |
| CWSNP3690 | Ca-Kabuli-Chr4 | 38474108 | [T/C] | Ca13112 | Intron | - |
| CWSNP3691 | Ca-Kabuli-Chr4 | 38474192 | [T/C] | Ca13112 | Intron | - |
| CWSNP3692 | Ca-Kabuli-Chr4 | 38475310 | [A/C] | Ca13112 | Intron | - |
| CWSNP3693 | Ca-Kabuli-Chr4 | 38475425 | [A/G] | Ca13112 | Intron | - |
| CWSNP3694 | Ca-Kabuli-Chr4 | 38475449 | [T/A] | Ca13112 | Intron | - |
| CWSNP3695 | Ca-Kabuli-Chr4 | 38475442 | [A/G] | Ca13112 | Intron | - |
| CWSNP3696 | Ca-Kabuli-Chr4 | 38475424 | [C/G] | Ca13112 | Intron | - |

| SNP IDs | Chromosomes | Physical positions (bp) | SNPs | Gene accession IDs | Sequence components of genes | Putative functions |
|---|---|---|---|---|---|---|
| CWSNP3697 | Ca-Kabuli-Chr4 | 38475547 | [C/T] | Ca13112 | Intron | - |
| CWSNP3698 | Ca-Kabuli-Chr4 | 38540201 | [C/T] | - | DRR | - |
| CWSNP3699 | Ca-Kabuli-Chr4 | 38540247 | [G/A] | - | DRR | - |
| CWSNP3700 | Ca-Kabuli-Chr4 | 38620579 | [C/T] | - | Intergenic | - |
| CWSNP3701 | Ca-Kabuli-Chr4 | 38620620 | [A/T] | - | Intergenic | - |
| CWSNP3702 | Ca-Kabuli-Chr4 | 38659377 | [T/C] | - | DRR | - |
| CWSNP3703 | Ca-Kabuli-Chr4 | 38659398 | [G/A] | - | DRR | - |
| CWSNP3704 | Ca-Kabuli-Chr4 | 38659534 | [T/A] | - | DRR | - |
| CWSNP3705 | Ca-Kabuli-Chr4 | 38679987 | [A/G] | Ca13099 | Intron | LIMbinding protein |
| CWSNP3706 | Ca-Kabuli-Chr4 | 38721154 | [A/G] | Ca13098 | Intron | Exostosin-like |
| CWSNP3707 | Ca-Kabuli-Chr4 | 38721160 | [A/G] | Ca13098 | Intron | Exostosin-like |
| CWSNP3708 | Ca-Kabuli-Chr4 | 38721207 | [A/C] | Ca13098 | Intron | Exostosin-like |
| CWSNP3709 | Ca-Kabuli-Chr4 | 38756507 | [T/C] | Ca13097 | Non-Synonymous-CDS | Auxin efflux carrier |
| CWSNP3710 | Ca-Kabuli-Chr4 | 38756510 | [C/G] | Ca13097 | Non-Synonymous-CDS | Auxin efflux carrier |
| CWSNP3711 | Ca-Kabuli-Chr4 | 38759640 | [A/C] | Ca13097 | Intron | Auxin efflux carrier |
| CWSNP3712 | Ca-Kabuli-Chr4 | 38770348 | [T/A] | Ca13096 | Non-Synonymous-CDS | Fattyaciddesaturase,type2 |

| **SNP IDs** | **Chromosomes** | **Physical positions (bp)** | **SNPs** | **Gene accession IDs** | **Sequence components of genes** | **Putative functions** |
|---|---|---|---|---|---|---|
| CWSNP3713 | Ca-Kabuli-Chr4 | 38770370 | [G/C] | Ca13096 | Non-Synonymous-CDS | Fattyaciddesaturase,type2 |
| CWSNP3714 | Ca-Kabuli-Chr4 | 38770450 | [C/G] | Ca13096 | Synonymous-CDS | Fattyaciddesaturase,type2 |
| CWSNP3715 | Ca-Kabuli-Chr4 | 38770510 | [G/A] | Ca13096 | Synonymous-CDS | Fattyaciddesaturase,type2 |
| CWSNP3716 | Ca-Kabuli-Chr4 | 38770606 | [G/A] | Ca13096 | Synonymous-CDS | Fattyaciddesaturase,type2 |
| CWSNP3717 | Ca-Kabuli-Chr4 | 38770582 | [G/A] | Ca13096 | Synonymous-CDS | Fattyaciddesaturase,type2 |
| CWSNP3718 | Ca-Kabuli-Chr4 | 38785535 | [G/A] | - | URR | - |
| CWSNP3719 | Ca-Kabuli-Chr4 | 38829959 | [T/G] | - | Intergenic | - |
| CWSNP3720 | Ca-Kabuli-Chr4 | 38833304 | [C/A] | - | Intergenic | - |
| CWSNP3721 | Ca-Kabuli-Chr4 | 38833280 | [C/T] | - | Intergenic | - |
| CWSNP3722 | Ca-Kabuli-Chr4 | 38833333 | [A/C] | - | Intergenic | - |
| CWSNP3723 | Ca-Kabuli-Chr4 | 38834383 | [G/A] | - | DRR | - |
| CWSNP3724 | Ca-Kabuli-Chr4 | 38834405 | [G/A] | - | DRR | - |
| CWSNP3725 | Ca-Kabuli-Chr4 | 38834423 | [G/C] | - | DRR | - |
| CWSNP3726 | Ca-Kabuli-Chr4 | 38834431 | [G/A] | - | DRR | - |
| CWSNP3727 | Ca-Kabuli-Chr4 | 38834477 | [A/G] | - | DRR | - |
| CWSNP3728 | Ca-Kabuli-Chr4 | 38834474 | [G/A] | - | DRR | - |

| SNP IDs | Chromosomes | Physical positions (bp) | SNPs | Gene accession IDs | Sequence components of genes | Putative functions |
|---|---|---|---|---|---|---|
| CWSNP3729 | Ca-Kabuli-Chr4 | 38888779 | [C/T] | Ca13087 | Synonymous-CDS | Protein kinase, catalytic domain |
| CWSNP3730 | Ca-Kabuli-Chr4 | 38888791 | [G/C] | Ca13087 | Synonymous-CDS | Protein kinase, catalytic domain |
| CWSNP3731 | Ca-Kabuli-Chr4 | 38889023 | [C/T] | Ca13087 | Non-Synonymous-CDS | Protein kinase, catalytic domain |
| CWSNP3732 | Ca-Kabuli-Chr4 | 38918394 | [A/T] | Ca13086 | Synonymous-CDS | General substrate transporter |
| CWSNP3733 | Ca-Kabuli-Chr4 | 38918424 | [G/A] | Ca13086 | Synonymous-CDS | General substrate transporter |
| CWSNP3734 | Ca-Kabuli-Chr4 | 38918430 | [C/A] | Ca13086 | Synonymous-CDS | General substrate transporter |
| CWSNP3735 | Ca-Kabuli-Chr4 | 38918628 | [C/T] | Ca13086 | Synonymous-CDS | General substrate transporter |
| CWSNP3736 | Ca-Kabuli-Chr4 | 38918547 | [T/C] | Ca13086 | Synonymous-CDS | General substrate transporter |
| CWSNP3737 | Ca-Kabuli-Chr4 | 38918649 | [G/C] | Ca13086 | Synonymous-CDS | General substrate transporter |
| CWSNP3738 | Ca-Kabuli-Chr4 | 38929681 | [A/G] | Ca13085 | Synonymous-CDS | - |
| CWSNP3739 | Ca-Kabuli-Chr4 | 38948414 | [C/A] | Ca13084 | Non-Synonymous-CDS | Pathogenesis-related transcriptional factor/ERF |
| CWSNP3740 | Ca-Kabuli-Chr4 | 38961521 | [G/A] | Ca13083 | Intron | Ubiquitin-conjugating enzyme,E2 |
| CWSNP3741 | Ca-Kabuli-Chr4 | 38978462 | [A/G] | Ca13081 | Synonymous-CDS | Folylpolyglutamate synthetase |
| CWSNP3742 | Ca-Kabuli-Chr4 | 39031801 | [C/T] | Ca13078 | Non-Synonymous-CDS | RNA recognition motif domain |
| CWSNP3743 | Ca-Kabuli-Chr4 | 39031823 | [G/A] | Ca13078 | Synonymous-CDS | RNA recognition motif domain |
| CWSNP3744 | Ca-Kabuli-Chr4 | 39042938 | [T/G] | Ca13077 | Intron | PeptidaseM41 |

| SNP IDs | Chromosomes | Physical positions (bp) | SNPs | Gene accession IDs | Sequence components of genes | Putative functions |
|---|---|---|---|---|---|---|
| CWSNP3745 | Ca-Kabuli-Chr4 | 39042921 | [T/C] | Ca13077 | Intron | PeptidaseM41 |
| CWSNP3746 | Ca-Kabuli-Chr4 | 39057991 | [T/C] | - | URR | - |
| CWSNP3747 | Ca-Kabuli-Chr4 | 39058011 | [G/C] | - | URR | - |
| CWSNP3748 | Ca-Kabuli-Chr4 | 39081951 | [A/G] | Ca13074 | Intron | Cyclicnucleotide-binding domain |
| CWSNP3749 | Ca-Kabuli-Chr4 | 39113327 | [G/A] | - | DRR | - |
| CWSNP3750 | Ca-Kabuli-Chr4 | 39113330 | [G/A] | - | DRR | - |
| CWSNP3751 | Ca-Kabuli-Chr4 | 39113401 | [A/T] | - | DRR | - |
| CWSNP3752 | Ca-Kabuli-Chr4 | 39136826 | [C/T] | - | Intergenic | - |
| CWSNP3753 | Ca-Kabuli-Chr4 | 39306900 | [G/A] | - | Intergenic | - |
| CWSNP3754 | Ca-Kabuli-Chr4 | 39635746 | [T/A] | - | Intergenic | - |
| CWSNP3755 | Ca-Kabuli-Chr4 | 39635721 | [A/G] | - | Intergenic | - |
| CWSNP3756 | Ca-Kabuli-Chr4 | 39635720 | [G/A] | - | Intergenic | - |
| CWSNP3757 | Ca-Kabuli-Chr4 | 39635718 | [C/T] | - | Intergenic | - |
| CWSNP3758 | Ca-Kabuli-Chr4 | 39650872 | [T/C] | - | Intergenic | - |
| CWSNP3759 | Ca-Kabuli-Chr4 | 39659757 | [C/T] | - | Intergenic | - |
| CWSNP3760 | Ca-Kabuli-Chr4 | 39659750 | [G/A] | - | Intergenic | - |

| SNP IDs | Chromosomes | Physical positions (bp) | SNPs | Gene accession IDs | Sequence components of genes | Putative functions |
|---|---|---|---|---|---|---|
| CWSNP3761 | Ca-Kabuli-Chr4 | 39715087 | [A/G] | Ca14929 | Synonymous-CDS | Glutamine amidotransferase,class-II |
| CWSNP3762 | Ca-Kabuli-Chr4 | 39719606 | [C/G] | Ca14929 | Synonymous-CDS | Glutamine amidotransferase,class-II |
| CWSNP3763 | Ca-Kabuli-Chr4 | 39719747 | [G/T] | Ca14929 | Synonymous-CDS | Glutamine amidotransferase,class-II |
| CWSNP3764 | Ca-Kabuli-Chr4 | 39803305 | [T/C] | Ca14925 | Synonymous-CDS | SANT domain, DNA binding |
| CWSNP3765 | Ca-Kabuli-Chr4 | 39964930 | [T/C] | Ca14914 | Synonymous-CDS | Exostosin-like |
| CWSNP3766 | Ca-Kabuli-Chr4 | 40010119 | [A/C] | Ca14911 | Non-Synonymous-CDS | Pathogenesis-related transcriptional factor/ERF, DNA-binding |
| CWSNP3767 | Ca-Kabuli-Chr4 | 40010201 | [A/G] | Ca14911 | Synonymous-CDS | Pathogenesis-related transcriptional factor/ERF, DNA-binding |
| CWSNP3768 | Ca-Kabuli-Chr4 | 40010177 | [G/A] | Ca14911 | Synonymous-CDS | Pathogenesis-related transcriptional factor/ERF, DNA-binding |
| CWSNP3769 | Ca-Kabuli-Chr4 | 40010165 | [A/C] | Ca14911 | Non-Synonymous-CDS | Pathogenesis-related transcriptional factor/ERF, DNA-binding |
| CWSNP3770 | Ca-Kabuli-Chr4 | 40060063 | [T/G] | Ca14910 | Synonymous-CDS | Nuclearpore complex protein,Nucleoporin Nup85-like |
| CWSNP3771 | Ca-Kabuli-Chr4 | 40061195 | [T/C] | Ca14910 | Intron | Nuclearpore complex protein,Nucleoporin Nup85-like |
| CWSNP3772 | Ca-Kabuli-Chr4 | 40061262 | [A/C] | Ca14910 | Intron | Nuclearpore complex protein,Nucleoporin Nup85-like |
| CWSNP3773 | Ca-Kabuli-Chr4 | 40065505 | [T/A] | Ca14910 | Intron | Nuclearpore complex protein,Nucleoporin Nup85-like |
| CWSNP3774 | Ca-Kabuli-Chr4 | 40134460 | [A/G] | Ca14906 | Intron | Exoribonuclease,phosphorolyticdomain1 |
| CWSNP3775 | Ca-Kabuli-Chr4 | 40134518 | [T/C] | Ca14906 | Intron | Exoribonuclease,phosphorolyticdomain1 |
| CWSNP3776 | Ca-Kabuli-Chr4 | 40149132 | [G/C] | Ca14905 | Intron | - |

| SNP IDs | Chromosomes | Physical positions (bp) | SNPs | Gene accession IDs | Sequence components of genes | Putative functions |
|---|---|---|---|---|---|---|
| CWSNP3777 | Ca-Kabuli-Chr4 | 40149324 | [G/C] | Ca14905 | Intron | - |
| CWSNP3778 | Ca-Kabuli-Chr4 | 40169176 | [T/G] | - | URR | - |
| CWSNP3779 | Ca-Kabuli-Chr4 | 40185606 | [A/G] | Ca14901 | Non-Synonymous-CDS | Amino acid transporter,transmembrane |
| CWSNP3780 | Ca-Kabuli-Chr4 | 40199692 | [A/C] | Ca14900 | Intron | Cofprotein |
| CWSNP3781 | Ca-Kabuli-Chr4 | 40218375 | [G/T] | - | URR | - |
| CWSNP3782 | Ca-Kabuli-Chr4 | 40218410 | [G/A] | - | URR | - |
| CWSNP3783 | Ca-Kabuli-Chr4 | 40267713 | [G/T] | - | Intergenic | - |
| CWSNP3784 | Ca-Kabuli-Chr4 | 40267719 | [G/A] | - | Intergenic | - |
| CWSNP3785 | Ca-Kabuli-Chr4 | 40279513 | [G/T] | - | Intergenic | - |
| CWSNP3786 | Ca-Kabuli-Chr4 | 40313721 | [A/G] | - | Intergenic | - |
| CWSNP3787 | Ca-Kabuli-Chr4 | 40349170 | [C/T] | - | Intergenic | - |
| CWSNP3788 | Ca-Kabuli-Chr4 | 40368278 | [T/C] | - | URR | - |
| CWSNP3789 | Ca-Kabuli-Chr4 | 40368765 | [G/A] | - | URR | - |
| CWSNP3790 | Ca-Kabuli-Chr4 | 40375386 | [C/T] | - | URR | - |
| CWSNP3791 | Ca-Kabuli-Chr4 | 40375361 | [A/G] | - | URR | - |
| CWSNP3792 | Ca-Kabuli-Chr4 | 40429387 | [G/A] | - | DRR | - |

| SNP IDs | Chromosomes | Physical positions (bp) | SNPs | Gene accession IDs | Sequence components of genes | Putative functions |
|---|---|---|---|---|---|---|
| CWSNP3793 | Ca-Kabuli-Chr4 | 40471412 | [G/C] | - | Intergenic | - |
| CWSNP3794 | Ca-Kabuli-Chr4 | 40472097 | [A/G] | - | Intergenic | - |
| CWSNP3795 | Ca-Kabuli-Chr4 | 40513733 | [C/T] | Ca14874 | Synonymous-CDS | IQ motif, EF-hand binding site |
| CWSNP3796 | Ca-Kabuli-Chr4 | 40583205 | [G/T] | Ca14868 | Non-Synonymous-CDS | Zinc finger,RING-type |
| CWSNP3797 | Ca-Kabuli-Chr4 | 40583206 | [A/T] | Ca14868 | Non-Synonymous-CDS | Zinc finger,RING-type |
| CWSNP3798 | Ca-Kabuli-Chr4 | 40583211 | [G/T] | Ca14868 | Non-Synonymous-CDS | Zinc finger,RING-type |
| CWSNP3799 | Ca-Kabuli-Chr4 | 40583214 | [G/A] | Ca14868 | Non-Synonymous-CDS | Zinc finger,RING-type |
| CWSNP3800 | Ca-Kabuli-Chr4 | 40583216 | [C/A] | Ca14868 | Non-Synonymous-CDS | Zinc finger,RING-type |
| CWSNP3801 | Ca-Kabuli-Chr4 | 40583217 | [G/A] | Ca14868 | Non-Synonymous-CDS | Zinc finger,RING-type |
| CWSNP3802 | Ca-Kabuli-Chr4 | 40583219 | [G/A] | Ca14868 | Synonymous-CDS | Zinc finger,RING-type |
| CWSNP3803 | Ca-Kabuli-Chr4 | 40597192 | [G/A] | - | DRR | - |
| CWSNP3804 | Ca-Kabuli-Chr4 | 40597534 | [A/G] | - | DRR | - |
| CWSNP3805 | Ca-Kabuli-Chr4 | 40597464 | [G/A] | - | DRR | - |
| CWSNP3806 | Ca-Kabuli-Chr4 | 40723208 | [C/G] | - | DRR | - |
| CWSNP3807 | Ca-Kabuli-Chr4 | 40723212 | [G/A] | - | DRR | - |
| CWSNP3808 | Ca-Kabuli-Chr4 | 40734572 | [T/G] | Ca23909 | Intron | PeptidaseS54,rhomboid |

| SNP IDs | Chromosomes | Physical positions (bp) | SNPs | Gene accession IDs | Sequence components of genes | Putative functions |
|---|---|---|---|---|---|---|
| CWSNP3809 | Ca-Kabuli-Chr4 | 40734765 | [G/A] | Ca23909 | Synonymous-CDS | PeptidaseS54,rhomboid |
| CWSNP3810 | Ca-Kabuli-Chr4 | 40738927 | [C/T] | Ca23908 | Synonymous-CDS | Pentatrico peptide repeat |
| CWSNP3811 | Ca-Kabuli-Chr4 | 40795060 | [G/A] | - | Intergenic | - |
| CWSNP3812 | Ca-Kabuli-Chr4 | 40795085 | [C/G] | - | Intergenic | - |
| CWSNP3813 | Ca-Kabuli-Chr4 | 40801783 | [T/C] | Ca23902 | Synonymous-CDS | DNA-directed DNA polymerase,family A,palm domain |
| CWSNP3814 | Ca-Kabuli-Chr4 | 40801791 | [C/G] | Ca23902 | Non-Synonymous-CDS | DNA-directed DNA polymerase,family A,palm domain |
| CWSNP3815 | Ca-Kabuli-Chr4 | 40805740 | [C/A] | Ca23902 | Synonymous-CDS | DNA-directed DNA polymerase,family A,palm domain |
| CWSNP3816 | Ca-Kabuli-Chr4 | 40806993 | [A/G] | Ca23902 | Synonymous-CDS | DNA-directed DNA polymerase,family A,palm domain |
| CWSNP3817 | Ca-Kabuli-Chr4 | 40839967 | [G/A] | - | DRR | - |
| CWSNP3818 | Ca-Kabuli-Chr4 | 40839949 | [G/A] | - | DRR | - |
| CWSNP3819 | Ca-Kabuli-Chr4 | 40852381 | [A/C] | Ca23897 | Synonymous-CDS | Globin,truncated bacterial-like |
| CWSNP3820 | Ca-Kabuli-Chr4 | 40852475 | [T/C] | Ca23897 | Intron | Globin,truncated bacterial-like |
| CWSNP3821 | Ca-Kabuli-Chr4 | 40912677 | [A/G] | - | Intergenic | - |
| CWSNP3822 | Ca-Kabuli-Chr4 | 40912681 | [A/G] | - | Intergenic | - |
| CWSNP3823 | Ca-Kabuli-Chr4 | 40912714 | [C/G] | - | Intergenic | - |
| CWSNP3824 | Ca-Kabuli-Chr4 | 40912731 | [C/T] | - | Intergenic | - |

| SNP IDs | Chromosomes | Physical positions (bp) | SNPs | Gene accession IDs | Sequence components of genes | Putative functions |
|---|---|---|---|---|---|---|
| CWSNP3825 | Ca-Kabuli-Chr4 | 40917776 | [C/A] | - | URR | - |
| CWSNP3826 | Ca-Kabuli-Chr4 | 40917777 | [T/A] | - | URR | - |
| CWSNP3827 | Ca-Kabuli-Chr4 | 40917807 | [C/T] | - | URR | - |
| CWSNP3828 | Ca-Kabuli-Chr4 | 40941283 | [A/G] | Ca11014 | Synonymous-CDS | Proteasome component (PCI) domain |
| CWSNP3829 | Ca-Kabuli-Chr4 | 41032564 | [A/G] | Ca11007 | Intron | GHMP kinase |
| CWSNP3830 | Ca-Kabuli-Chr4 | 41032590 | [C/A] | Ca11007 | Intron | GHMP kinase |
| CWSNP3831 | Ca-Kabuli-Chr4 | 41032599 | [T/C] | Ca11007 | Intron | GHMP kinase |
| CWSNP3832 | Ca-Kabuli-Chr4 | 41094082 | [T/C] | Ca10999 | Non-Synonymous-CDS | Protein kinase, catalytic domain |
| CWSNP3833 | Ca-Kabuli-Chr4 | 41094554 | [A/C] | Ca10999 | Intron | Protein kinase, catalytic domain |
| CWSNP3834 | Ca-Kabuli-Chr4 | 41117822 | [C/A] | Ca10997 | Intron | DNA topoisomerase,typeIA |
| CWSNP3835 | Ca-Kabuli-Chr4 | 41141086 | [G/T] | Ca10994 | Non-Synonymous-CDS | - |
| CWSNP3836 | Ca-Kabuli-Chr4 | 41142299 | [T/C] | Ca10994 | Synonymous-CDS | - |
| CWSNP3837 | Ca-Kabuli-Chr4 | 41142290 | [A/G] | Ca10994 | Synonymous-CDS | - |
| CWSNP3838 | Ca-Kabuli-Chr4 | 41142288 | [T/C] | Ca10994 | Non-Synonymous-CDS | - |
| CWSNP3839 | Ca-Kabuli-Chr4 | 41144277 | [T/A] | Ca10994 | Non-Synonymous-CDS | - |
| CWSNP3840 | Ca-Kabuli-Chr4 | 41145767 | [C/T] | Ca10994 | Synonymous-CDS | - |

| SNP IDs | Chromosomes | Physical positions (bp) | SNPs | Gene accession IDs | Sequence components of genes | Putative functions |
|---|---|---|---|---|---|---|
| CWSNP3841 | Ca-Kabuli-Chr4 | 41145875 | [G/A] | Ca10994 | Synonymous-CDS | - |
| CWSNP3842 | Ca-Kabuli-Chr4 | 41207356 | [C/T] | Ca10990 | Intron | Cystathioninebeta-synthase,core |
| CWSNP3843 | Ca-Kabuli-Chr4 | 41207354 | [A/C] | Ca10990 | Intron | Cystathioninebeta-synthase,core |
| CWSNP3844 | Ca-Kabuli-Chr4 | 41209711 | [C/A] | Ca10990 | Intron | Cystathioninebeta-synthase,core |
| CWSNP3845 | Ca-Kabuli-Chr4 | 41215850 | [A/C] | - | DRR | - |
| CWSNP3846 | Ca-Kabuli-Chr4 | 41229605 | [C/T] | - | DRR | - |
| CWSNP3847 | Ca-Kabuli-Chr4 | 41292152 | [T/C] | Ca10980 | Non-Synonymous-CDS | ATPase ,P-type, H+transporting proton pump |
| CWSNP3848 | Ca-Kabuli-Chr4 | 41427086 | [C/A] | - | Intergenic | - |
| CWSNP3849 | Ca-Kabuli-Chr4 | 41445225 | [A/T] | Ca10963 | Intron | Peptidase C19,ubiquitin carboxyl-terminalhydrolase 2 |
| CWSNP3850 | Ca-Kabuli-Chr4 | 41512840 | [G/A] | Ca10956 | Intron | Skb1methyltransferase |
| CWSNP3851 | Ca-Kabuli-Chr4 | 41568682 | [A/C] | - | Intergenic | - |
| CWSNP3852 | Ca-Kabuli-Chr4 | 41629852 | [T/A] | - | DRR | - |
| CWSNP3853 | Ca-Kabuli-Chr4 | 41648927 | [C/T] | Ca10946 | Synonymous-CDS | - |
| CWSNP3854 | Ca-Kabuli-Chr4 | 41648998 | [A/G] | Ca10946 | Synonymous-CDS | - |
| CWSNP3855 | Ca-Kabuli-Chr4 | 41698291 | [A/G] | - | Intergenic | - |
| CWSNP3856 | Ca-Kabuli-Chr4 | 41698386 | [A/G] | - | Intergenic | - |

| SNP IDs | Chromosomes | Physical positions (bp) | SNPs | Gene accession IDs | Sequence components of genes | Putative functions |
|---|---|---|---|---|---|---|
| CWSNP3857 | Ca-Kabuli-Chr4 | 41721289 | [G/A] | Ca10941 | Synonymous-CDS | - |
| CWSNP3858 | Ca-Kabuli-Chr4 | 41772690 | [C/T] | - | Intergenic | - |
| CWSNP3859 | Ca-Kabuli-Chr4 | 41772796 | [T/A] | - | Intergenic | - |
| CWSNP3860 | Ca-Kabuli-Chr4 | 41772794 | [T/C] | - | Intergenic | - |
| CWSNP3861 | Ca-Kabuli-Chr4 | 41864826 | [G/A] | Ca10932 | Synonymous-CDS | WD40 repeat |
| CWSNP3862 | Ca-Kabuli-Chr4 | 41864846 | [C/G] | Ca10932 | Non-Synonymous-CDS | WD40 repeat |
| CWSNP3863 | Ca-Kabuli-Chr4 | 41956050 | [C/G] | - | URR | - |
| CWSNP3864 | Ca-Kabuli-Chr4 | 41956085 | [T/C] | - | URR | - |
| CWSNP3865 | Ca-Kabuli-Chr4 | 41987193 | [A/G] | Ca10923 | Intron | Histone H2A |
| CWSNP3866 | Ca-Kabuli-Chr4 | 42092379 | [T/G] | Ca10914 | Intron | Tesmin/TSO1-like,CXC |
| CWSNP3867 | Ca-Kabuli-Chr4 | 42092381 | [A/G] | Ca10914 | Intron | Tesmin/TSO1-like,CXC |
| CWSNP3868 | Ca-Kabuli-Chr4 | 42092385 | [G/A] | Ca10914 | Intron | Tesmin/TSO1-like,CXC |
| CWSNP3869 | Ca-Kabuli-Chr4 | 42105737 | [C/T] | Ca10912 | Intron | ATPase,AAA+type,core |
| CWSNP3870 | Ca-Kabuli-Chr4 | 42105701 | [A/T] | Ca10912 | Intron | ATPase,AAA+type,core |
| CWSNP3871 | Ca-Kabuli-Chr4 | 42246527 | [C/A] | - | Intergenic | - |
| CWSNP3872 | Ca-Kabuli-Chr4 | 42246565 | [T/G] | - | Intergenic | - |

| SNP IDs | Chromosomes | Physical positions (bp) | SNPs | Gene accession IDs | Sequence components of genes | Putative functions |
|---|---|---|---|---|---|---|
| CWSNP3873 | Ca-Kabuli-Chr4 | 42273252 | [G/A] | Ca10903 | Intron | Methyltransferase type11 |
| CWSNP3874 | Ca-Kabuli-Chr4 | 42499699 | [A/T] | Ca10898 | Non-Synonymous-CDS | Protein kinase, catalytic domain |
| CWSNP3875 | Ca-Kabuli-Chr4 | 42768142 | [T/G] | - | Intergenic | - |
| CWSNP3876 | Ca-Kabuli-Chr4 | 42807483 | [G/A] | - | Intergenic | - |
| CWSNP3877 | Ca-Kabuli-Chr4 | 42807518 | [A/G] | - | Intergenic | - |
| CWSNP3878 | Ca-Kabuli-Chr4 | 43319846 | [A/G] | Ca23683 | Synonymous-CDS | Cyclin,C-terminal |
| CWSNP3879 | Ca-Kabuli-Chr4 | 43324547 | [G/C] | - | DRR | - |
| CWSNP3880 | Ca-Kabuli-Chr4 | 43431770 | [C/T] | - | Intergenic | - |
| CWSNP3881 | Ca-Kabuli-Chr4 | 43431772 | [T/A] | - | Intergenic | - |
| CWSNP3882 | Ca-Kabuli-Chr4 | 43431815 | [G/T] | - | Intergenic | - |
| CWSNP3883 | Ca-Kabuli-Chr4 | 43447262 | [A/T] | - | DRR | - |
| CWSNP3884 | Ca-Kabuli-Chr4 | 43476193 | [T/C] | Ca23692 | Synonymous-CDS | PollenOlee1allergen/extensin |
| CWSNP3885 | Ca-Kabuli-Chr4 | 43653225 | [A/G] | - | Intergenic | - |
| CWSNP3886 | Ca-Kabuli-Chr4 | 43653226 | [C/T] | - | Intergenic | - |
| CWSNP3887 | Ca-Kabuli-Chr4 | 43662224 | [A/C] | - | Intergenic | - |
| CWSNP3888 | Ca-Kabuli-Chr4 | 43677006 | [T/G] | Ca09052 | Non-Synonymous-CDS | Leucine-rich repeat |

| SNP IDs | Chromosomes | Physical positions (bp) | SNPs | Gene accession IDs | Sequence components of genes | Putative functions |
|---|---|---|---|---|---|---|
| CWSNP3889 | Ca-Kabuli-Chr4 | 43722728 | [T/C] | - | DRR | - |
| CWSNP3890 | Ca-Kabuli-Chr4 | 43722727 | [A/G] | - | DRR | - |
| CWSNP3891 | Ca-Kabuli-Chr4 | 43726406 | [C/T] | - | DRR | - |
| CWSNP3892 | Ca-Kabuli-Chr4 | 43807399 | [C/T] | Ca09060 | Synonymous-CDS | WD40 repeat |
| CWSNP3893 | Ca-Kabuli-Chr4 | 43807460 | [C/T] | Ca09060 | Intron | WD40 repeat |
| CWSNP3894 | Ca-Kabuli-Chr4 | 43840650 | [C/T] | - | URR | - |
| CWSNP3895 | Ca-Kabuli-Chr4 | 43979065 | [A/G] | - | DRR | - |
| CWSNP3896 | Ca-Kabuli-Chr4 | 44059060 | [C/T] | Ca09080 | Non-Synonymous-CDS | Signal transduction response regulator,receiver domain |
| CWSNP3897 | Ca-Kabuli-Chr4 | 44239883 | [A/T] | - | Intergenic | - |
| CWSNP3898 | Ca-Kabuli-Chr4 | 44250159 | [C/T] | - | DRR | - |
| CWSNP3899 | Ca-Kabuli-Chr4 | 44261184 | [A/C] | Ca09100 | Synonymous-CDS | Actin-binding FH2/DRF auto regulatory |
| CWSNP3900 | Ca-Kabuli-Chr4 | 44266692 | [T/A] | - | Intergenic | - |
| CWSNP3901 | Ca-Kabuli-Chr4 | 44353354 | [C/T] | Ca09105 | Intron | Protein of unknown function DUF1077,TMEM85 |
| CWSNP3902 | Ca-Kabuli-Chr4 | 44353343 | [G/A] | Ca09105 | Intron | Protein of unknown function DUF1077,TMEM85 |
| CWSNP3903 | Ca-Kabuli-Chr4 | 44375761 | [T/C] | Ca09108 | Synonymous-CDS | - |
| CWSNP3904 | Ca-Kabuli-Chr4 | 44375831 | [T/C] | Ca09108 | Intron | - |

| SNP IDs | Chromosomes | Physical positions (bp) | SNPs | Gene accession IDs | Sequence components of genes | Putative functions |
|---|---|---|---|---|---|---|
| CWSNP3905 | Ca-Kabuli-Chr4 | 44382515 | [A/G] | - | Intergenic | - |
| CWSNP3906 | Ca-Kabuli-Chr4 | 44382677 | [C/T] | - | Intergenic | - |
| CWSNP3907 | Ca-Kabuli-Chr4 | 44382671 | [C/G] | - | Intergenic | - |
| CWSNP3908 | Ca-Kabuli-Chr4 | 44382614 | [C/A] | - | Intergenic | - |
| CWSNP3909 | Ca-Kabuli-Chr4 | 44382602 | [A/G] | - | Intergenic | - |
| CWSNP3910 | Ca-Kabuli-Chr4 | 44386579 | [A/G] | Ca09109 | Synonymous-CDS | Protein phosphatase2C,manganese/magnesiuma spartate binding site |
| CWSNP3911 | Ca-Kabuli-Chr4 | 44431319 | [A/C] | Ca09114 | Intron | Peptidase C19,ubiquitin carboxyl-terminalhydrolase 2 |
| CWSNP3912 | Ca-Kabuli-Chr4 | 44439503 | [C/A] | - | DRR | - |
| CWSNP3913 | Ca-Kabuli-Chr4 | 44439495 | [G/A] | - | DRR | - |
| CWSNP3914 | Ca-Kabuli-Chr4 | 44439492 | [G/T] | - | DRR | - |
| CWSNP3915 | Ca-Kabuli-Chr4 | 44446468 | [G/A] | - | URR | - |
| CWSNP3916 | Ca-Kabuli-Chr4 | 44446575 | [G/A] | - | URR | - |
| CWSNP3917 | Ca-Kabuli-Chr4 | 44446503 | [T/G] | - | URR | - |
| CWSNP3918 | Ca-Kabuli-Chr4 | 44472278 | [A/G] | - | Intergenic | - |
| CWSNP3919 | Ca-Kabuli-Chr4 | 44472237 | [T/A] | - | Intergenic | - |
| CWSNP3920 | Ca-Kabuli-Chr4 | 44590652 | [T/C] | Ca09131 | Synonymous-CDS | Protein phosphatase2C,manganese/magnesiuma spartate binding site |

| SNP IDs | Chromosomes | Physical positions (bp) | SNPs | Gene accession IDs | Sequence components of genes | Putative functions |
|---|---|---|---|---|---|---|
| CWSNP3921 | Ca-Kabuli-Chr4 | 44590678 | [G/A] | Ca09131 | Non-Synonymous-CDS | Protein phosphatase2C,manganese/magnesiumaspartate binding site |
| CWSNP3922 | Ca-Kabuli-Chr4 | 44590697 | [A/C] | Ca09131 | Synonymous-CDS | Protein phosphatase2C,manganese/magnesiumaspartate binding site |
| CWSNP3923 | Ca-Kabuli-Chr4 | 44591405 | [G/T] | Ca09131 | Intron | Protein phosphatase2C,manganese/magnesiumaspartate binding site |
| CWSNP3924 | Ca-Kabuli-Chr4 | 44754355 | [C/T] | Ca09143 | Intron | F-boxdomain,cyclin-like |
| CWSNP3925 | Ca-Kabuli-Chr4 | 44802374 | [G/A] | Ca09145 | Intron | PhosphoribosyltransferaseC-terminal |
| CWSNP3926 | Ca-Kabuli-Chr4 | 44802364 | [A/C] | Ca09145 | Intron | PhosphoribosyltransferaseC-terminal |
| CWSNP3927 | Ca-Kabuli-Chr4 | 44803694 | [T/A] | Ca09145 | Intron | PhosphoribosyltransferaseC-terminal |
| CWSNP3928 | Ca-Kabuli-Chr4 | 44811519 | [G/A] | - | Intergenic | - |
| CWSNP3929 | Ca-Kabuli-Chr4 | 44814857 | [A/G] | - | URR | - |
| CWSNP3930 | Ca-Kabuli-Chr4 | 44814981 | [T/C] | - | URR | - |
| CWSNP3931 | Ca-Kabuli-Chr4 | 44814982 | [A/G] | - | URR | - |
| CWSNP3932 | Ca-Kabuli-Chr4 | 44824103 | [G/T] | - | Intergenic | - |
| CWSNP3933 | Ca-Kabuli-Chr4 | 44824105 | [T/G] | - | Intergenic | - |
| CWSNP3934 | Ca-Kabuli-Chr4 | 44832121 | [G/C] | - | Intergenic | - |
| CWSNP3935 | Ca-Kabuli-Chr4 | 44832144 | [T/C] | - | Intergenic | - |
| CWSNP3936 | Ca-Kabuli-Chr4 | 44906128 | [C/A] | Ca09153 | Intron | Protein of unknown function DUF803 |

| SNP IDs | Chromosomes | Physical positions (bp) | SNPs | Gene accession IDs | Sequence components of genes | Putative functions |
|---|---|---|---|---|---|---|
| CWSNP3937 | Ca-Kabuli-Chr4 | 44906255 | [A/G] | Ca09153 | Synonymous-CDS | Protein of unknown function DUF803 |
| CWSNP3938 | Ca-Kabuli-Chr4 | 44979108 | [G/A] | - | Intergenic | - |
| CWSNP3939 | Ca-Kabuli-Chr4 | 45019764 | [T/C] | - | Intergenic | - |
| CWSNP3940 | Ca-Kabuli-Chr4 | 45019778 | [T/C] | - | Intergenic | - |
| CWSNP3941 | Ca-Kabuli-Chr4 | 45019805 | [C/A] | - | Intergenic | - |
| CWSNP3942 | Ca-Kabuli-Chr4 | 45019807 | [C/T] | - | Intergenic | - |
| CWSNP3943 | Ca-Kabuli-Chr4 | 45019814 | [C/A] | - | Intergenic | - |
| CWSNP3944 | Ca-Kabuli-Chr4 | 45019820 | [C/T] | - | Intergenic | - |
| CWSNP3945 | Ca-Kabuli-Chr4 | 45019824 | [A/C] | - | Intergenic | - |
| CWSNP3946 | Ca-Kabuli-Chr4 | 45019867 | [C/G] | - | Intergenic | - |
| CWSNP3947 | Ca-Kabuli-Chr4 | 45019848 | [C/T] | - | Intergenic | - |
| CWSNP3948 | Ca-Kabuli-Chr4 | 45035589 | [G/T] | - | Intergenic | - |
| CWSNP3949 | Ca-Kabuli-Chr4 | 45035745 | [A/G] | - | Intergenic | - |
| CWSNP3950 | Ca-Kabuli-Chr4 | 45104767 | [G/A] | - | Intergenic | - |
| CWSNP3951 | Ca-Kabuli-Chr4 | 45104779 | [C/A] | - | Intergenic | - |
| CWSNP3952 | Ca-Kabuli-Chr4 | 45165191 | [T/C] | - | Intergenic | - |

| SNP IDs | Chromosomes | Physical positions (bp) | SNPs | Gene accession IDs | Sequence components of genes | Putative functions |
|---|---|---|---|---|---|---|
| CWSNP3953 | Ca-Kabuli-Chr4 | 45165240 | [T/C] | - | Intergenic | - |
| CWSNP3954 | Ca-Kabuli-Chr4 | 45165231 | [A/C] | - | Intergenic | - |
| CWSNP3955 | Ca-Kabuli-Chr4 | 45206209 | [G/C] | - | Intergenic | - |
| CWSNP3956 | Ca-Kabuli-Chr4 | 45220084 | [G/T] | Ca09184 | Synonymous-CDS | K Homology |
| CWSNP3957 | Ca-Kabuli-Chr4 | 45220916 | [T/C] | Ca09184 | Non-Synonymous-CDS | K Homology |
| CWSNP3958 | Ca-Kabuli-Chr4 | 45225063 | [T/C] | - | URR | - |
| CWSNP3959 | Ca-Kabuli-Chr4 | 45225059 | [G/A] | - | URR | - |
| CWSNP3960 | Ca-Kabuli-Chr4 | 45224997 | [G/C] | - | URR | - |
| CWSNP3961 | Ca-Kabuli-Chr4 | 45225077 | [A/T] | - | URR | - |
| CWSNP3962 | Ca-Kabuli-Chr4 | 45225093 | [G/A] | - | URR | - |
| CWSNP3963 | Ca-Kabuli-Chr4 | 45225145 | [T/A] | - | URR | - |
| CWSNP3964 | Ca-Kabuli-Chr4 | 45225155 | [C/A] | - | URR | - |
| CWSNP3965 | Ca-Kabuli-Chr4 | 45225165 | [T/C] | - | URR | - |
| CWSNP3966 | Ca-Kabuli-Chr4 | 45231839 | [T/C] | - | Intergenic | - |
| CWSNP3967 | Ca-Kabuli-Chr4 | 45231893 | [G/T] | - | Intergenic | - |
| CWSNP3968 | Ca-Kabuli-Chr4 | 45292918 | [T/G] | - | Intergenic | - |

| SNP IDs | Chromosomes | Physical positions (bp) | SNPs | Gene accession IDs | Sequence components of genes | Putative functions |
|---|---|---|---|---|---|---|
| CWSNP3969 | Ca-Kabuli-Chr4 | 45292981 | [G/T] | - | Intergenic | - |
| CWSNP3970 | Ca-Kabuli-Chr4 | 45292969 | [T/A] | - | Intergenic | - |
| CWSNP3971 | Ca-Kabuli-Chr4 | 45292960 | [A/T] | - | Intergenic | - |
| CWSNP3972 | Ca-Kabuli-Chr4 | 45645996 | [A/G] | - | Intergenic | - |
| CWSNP3973 | Ca-Kabuli-Chr4 | 45645957 | [A/C] | - | Intergenic | - |
| CWSNP3974 | Ca-Kabuli-Chr4 | 45708895 | [C/A] | - | Intergenic | - |
| CWSNP3975 | Ca-Kabuli-Chr4 | 45752844 | [G/A] | - | Intergenic | - |
| CWSNP3976 | Ca-Kabuli-Chr4 | 45752869 | [G/T] | - | Intergenic | - |
| CWSNP3977 | Ca-Kabuli-Chr4 | 45752874 | [A/T] | - | Intergenic | - |
| CWSNP3978 | Ca-Kabuli-Chr4 | 45918351 | [A/C] | - | DRR | - |
| CWSNP3979 | Ca-Kabuli-Chr4 | 45935594 | [A/C] | Ca18367 | Non-Synonymous-CDS | RNA recognition motif domain |
| CWSNP3980 | Ca-Kabuli-Chr4 | 46148985 | [A/T] | Ca18357 | Intron | Zinc finger,RING-type |
| CWSNP3981 | Ca-Kabuli-Chr4 | 46149013 | [A/G] | Ca18357 | Intron | Zinc finger,RING-type |
| CWSNP3982 | Ca-Kabuli-Chr4 | 46150670 | [T/C] | Ca18357 | Intron | Zinc finger,RING-type |
| CWSNP3983 | Ca-Kabuli-Chr4 | 46202862 | [A/G] | - | Intergenic | - |
| CWSNP3984 | Ca-Kabuli-Chr4 | 46612244 | [C/T] | - | Intergenic | - |

| SNP IDs | Chromosomes | Physical positions (bp) | SNPs | Gene accession IDs | Sequence components of genes | Putative functions |
|---|---|---|---|---|---|---|
| CWSNP3985 | Ca-Kabuli-Chr4 | 46613068 | [T/G] | - | Intergenic | - |
| CWSNP3986 | Ca-Kabuli-Chr4 | 46694919 | [T/A] | - | DRR | - |
| CWSNP3987 | Ca-Kabuli-Chr4 | 46695043 | [A/C] | - | DRR | - |
| CWSNP3988 | Ca-Kabuli-Chr4 | 46695008 | [T/C] | - | DRR | - |
| CWSNP3989 | Ca-Kabuli-Chr4 | 46705178 | [A/T] | Ca19247 | Synonymous-CDS | Patatin/PhospholipaseA2-related |
| CWSNP3990 | Ca-Kabuli-Chr4 | 46705225 | [A/C] | Ca19247 | Intron | Patatin/PhospholipaseA2-related |
| CWSNP3991 | Ca-Kabuli-Chr4 | 46713972 | [C/T] | Ca19246 | Non-Synonymous-CDS | PGAP1-like |
| CWSNP3992 | Ca-Kabuli-Chr4 | 46715350 | [T/C] | Ca19246 | Synonymous-CDS | PGAP1-like |
| CWSNP3993 | Ca-Kabuli-Chr4 | 46717153 | [C/T] | Ca19246 | Intron | PGAP1-like |
| CWSNP3994 | Ca-Kabuli-Chr4 | 46717200 | [C/T] | Ca19246 | Intron | PGAP1-like |
| CWSNP3995 | Ca-Kabuli-Chr4 | 46717508 | [G/A] | Ca19246 | Intron | PGAP1-like |
| CWSNP3996 | Ca-Kabuli-Chr4 | 46763540 | [C/T] | Ca19242 | Synonymous-CDS | RNA recognition motif domain |
| CWSNP3997 | Ca-Kabuli-Chr4 | 46763555 | [C/T] | Ca19242 | Synonymous-CDS | RNA recognition motif domain |
| CWSNP3998 | Ca-Kabuli-Chr4 | 46763868 | [T/C] | Ca19242 | Intron | RNA recognition motif domain |
| CWSNP3999 | Ca-Kabuli-Chr4 | 46763841 | [A/C] | Ca19242 | Intron | RNA recognition motif domain |
| CWSNP4000 | Ca-Kabuli-Chr4 | 47223016 | [T/C] | Ca10859 | Intron | Xanthine/uracil/vitaminCpermease |

| SNP IDs | Chromosomes | Physical positions (bp) | SNPs | Gene accession IDs | Sequence components of genes | Putative functions |
|---|---|---|---|---|---|---|
| CWSNP4001 | Ca-Kabuli-Chr4 | 47223047 | [A/G] | Ca10859 | Intron | Xanthine/uracil/vitaminCpermease |
| CWSNP4002 | Ca-Kabuli-Chr4 | 47223635 | [G/A] | Ca10859 | Synonymous-CDS | Xanthine/uracil/vitaminCpermease |
| CWSNP4003 | Ca-Kabuli-Chr4 | 47223668 | [T/G] | Ca10859 | Synonymous-CDS | Xanthine/uracil/vitaminCpermease |
| CWSNP4004 | Ca-Kabuli-Chr4 | 47379992 | [C/T] | - | Intergenic | - |
| CWSNP4005 | Ca-Kabuli-Chr4 | 47405685 | [T/A] | - | Intergenic | - |
| CWSNP4006 | Ca-Kabuli-Chr4 | 47419514 | [A/G] | - | DRR | - |
| CWSNP4007 | Ca-Kabuli-Chr4 | 47419517 | [C/T] | - | DRR | - |
| CWSNP4008 | Ca-Kabuli-Chr4 | 47428580 | [C/T] | Ca10845 | Intron | Multiantimicrobial extrusion protein |
| CWSNP4009 | Ca-Kabuli-Chr4 | 47428599 | [C/T] | Ca10845 | Intron | Multiantimicrobial extrusion protein |
| CWSNP4010 | Ca-Kabuli-Chr4 | 47428733 | [A/G] | Ca10845 | Synonymous-CDS | Multiantimicrobial extrusion protein |
| CWSNP4011 | Ca-Kabuli-Chr4 | 47450409 | [C/T] | - | Intergenic | - |
| CWSNP4012 | Ca-Kabuli-Chr4 | 47572228 | [G/A] | - | Intergenic | - |
| CWSNP4013 | Ca-Kabuli-Chr4 | 47572276 | [C/T] | - | Intergenic | - |
| CWSNP4014 | Ca-Kabuli-Chr4 | 47578556 | [T/G] | Ca10832 | Intron | MT-A70 |
| CWSNP4015 | Ca-Kabuli-Chr4 | 47578596 | [C/T] | Ca10832 | Intron | MT-A71 |
| CWSNP4016 | Ca-Kabuli-Chr4 | 47588760 | [G/A] | Ca10830 | Intron | Phospholipid/glycerolacyltransferase |

| SNP IDs | Chromosomes | Physical positions (bp) | SNPs | Gene accession IDs | Sequence components of genes | Putative functions |
|---|---|---|---|---|---|---|
| CWSNP4017 | Ca-Kabuli-Chr4 | 47612395 | [C/A] | - | Intergenic | - |
| CWSNP4018 | Ca-Kabuli-Chr4 | 47612396 | [G/T] | - | Intergenic | - |
| CWSNP4019 | Ca-Kabuli-Chr4 | 47622112 | [G/T] | - | URR | - |
| CWSNP4020 | Ca-Kabuli-Chr4 | 47798580 | [A/G] | - | Intergenic | - |
| CWSNP4021 | Ca-Kabuli-Chr4 | 48008987 | [T/C] | Ca10788 | Synonymous-CDS | Heat shock protein DnaJ,N-terminal |
| CWSNP4022 | Ca-Kabuli-Chr4 | 48103153 | [A/C] | Ca10777 | Synonymous-CDS | Plant peroxidase |
| CWSNP4023 | Ca-Kabuli-Chr4 | 48238919 | [C/T] | Ca10764 | Non-Synonymous-CDS | - |
| CWSNP4024 | Ca-Kabuli-Chr4 | 48239048 | [A/G] | Ca10764 | Intron | - |
| CWSNP4025 | Ca-Kabuli-Chr4 | 48263603 | [G/A] | Ca10761 | Synonymous-CDS | DNA-directed RNA polymerase, subunit 2,domain 6 |
| CWSNP4026 | Ca-Kabuli-Chr4 | 48269138 | [G/C] | Ca10760 | Intron | Amineoxidase |
| CWSNP4027 | Ca-Kabuli-Chr4 | 48275016 | [C/T] | Ca10760 | Synonymous-CDS | Amineoxidase |
| CWSNP4028 | Ca-Kabuli-Chr4 | 48322502 | [C/T] | - | Intergenic | - |
| CWSNP4029 | Ca-Kabuli-Chr4 | 48322507 | [C/T] | - | Intergenic | - |
| CWSNP4030 | Ca-Kabuli-Chr4 | 48331069 | [C/A] | Ca10753 | Non-Synonymous-CDS | Ribosomal protein L13 |
| CWSNP4031 | Ca-Kabuli-Chr4 | 48345604 | [A/G] | Ca10752 | Intron | IQ motif, EF-hand binding site |
| CWSNP4032 | Ca-Kabuli-Chr4 | 48345608 | [G/T] | Ca10752 | Intron | IQ motif, EF-hand binding site |

| SNP IDs | Chromosomes | Physical positions (bp) | SNPs | Gene accession IDs | Sequence components of genes | Putative functions |
|---|---|---|---|---|---|---|
| CWSNP4033 | Ca-Kabuli-Chr4 | 48345655 | [A/C] | Ca10752 | Intron | IQ motif, EF-hand binding site |
| CWSNP4034 | Ca-Kabuli-Chr4 | 48355358 | [A/T] | Ca10751 | Intron | Exonuclease |
| CWSNP4035 | Ca-Kabuli-Chr4 | 48355468 | [G/T] | Ca10751 | Intron | Exonuclease |
| CWSNP4036 | Ca-Kabuli-Chr4 | 48364170 | [G/A] | Ca10750 | Intron | Helicase,C-terminal |
| CWSNP4037 | Ca-Kabuli-Chr4 | 48365208 | [C/A] | - | Intergenic | - |
| CWSNP4038 | Ca-Kabuli-Chr4 | 48365388 | [G/A] | - | DRR | - |
| CWSNP4039 | Ca-Kabuli-Chr4 | 48382666 | [G/C] | Ca10748 | Synonymous-CDS | AUX/IAA protein |
| CWSNP4040 | Ca-Kabuli-Chr4 | 48464493 | [T/C] | Ca10746 | Non-Synonymous-CDS | Zinc finger,CCHC-type |
| CWSNP4041 | Ca-Kabuli-Chr4 | 48470477 | [A/G] | Ca10746 | Intron | Zinc finger,CCHC-type |
| CWSNP4042 | Ca-Kabuli-Chr4 | 48475447 | [T/C] | - | DRR | - |
| CWSNP4043 | Ca-Kabuli-Chr4 | 48475461 | [G/A] | - | DRR | - |
| CWSNP4044 | Ca-Kabuli-Chr4 | 48478018 | [A/G] | - | DRR | - |
| CWSNP4045 | Ca-Kabuli-Chr4 | 48478058 | [T/C] | - | DRR | - |
| CWSNP4046 | Ca-Kabuli-Chr4 | 48478303 | [G/A] | Ca10745 | Synonymous-CDS | Appr-1-pprocessing |
| CWSNP4047 | Ca-Kabuli-Chr4 | 48478524 | [C/T] | Ca10745 | Non-Synonymous-CDS | Appr-1-pprocessing |
| CWSNP4048 | Ca-Kabuli-Chr4 | 48484453 | [A/T] | - | DRR | - |

| SNP IDs | Chromosomes | Physical positions (bp) | SNPs | Gene accession IDs | Sequence components of genes | Putative functions |
|---|---|---|---|---|---|---|
| CWSNP4049 | Ca-Kabuli-Chr4 | 48497731 | [A/C] | - | DRR | - |
| CWSNP4050 | Ca-Kabuli-Chr4 | 48497725 | [A/C] | - | DRR | - |
| CWSNP4051 | Ca-Kabuli-Chr4 | 48497756 | [A/G] | - | DRR | - |
| CWSNP4052 | Ca-Kabuli-Chr4 | 48497765 | [G/A] | - | DRR | - |
| CWSNP4053 | Ca-Kabuli-Chr4 | 48497839 | [G/A] | - | DRR | - |
| CWSNP4054 | Ca-Kabuli-Chr4 | 48498133 | [C/T] | - | Intergenic | - |
| CWSNP4055 | Ca-Kabuli-Chr4 | 48498181 | [G/C] | - | Intergenic | - |
| CWSNP4056 | Ca-Kabuli-Chr4 | 48498166 | [A/G] | - | Intergenic | - |
| CWSNP4057 | Ca-Kabuli-Chr4 | 48498368 | [C/T] | - | Intergenic | - |
| CWSNP4058 | Ca-Kabuli-Chr4 | 48498334 | [T/G] | - | Intergenic | - |
| CWSNP4059 | Ca-Kabuli-Chr4 | 48498331 | [T/A] | - | Intergenic | - |
| CWSNP4060 | Ca-Kabuli-Chr4 | 48498312 | [T/C] | - | Intergenic | - |
| CWSNP4061 | Ca-Kabuli-Chr4 | 48501662 | [A/T] | Ca10741 | Synonymous-CDS | Eukaryotic initiation factor 3,gamma subunit |
| CWSNP4062 | Ca-Kabuli-Chr4 | 48502554 | [G/T] | Ca10741 | Intron | Eukaryotic initiation factor 3,gamma subunit |
| CWSNP4063 | Ca-Kabuli-Chr4 | 48502718 | [G/C] | Ca10741 | Intron | Eukaryotic initiation factor 3,gamma subunit |
| CWSNP4064 | Ca-Kabuli-Chr4 | 48502714 | [T/C] | Ca10741 | Intron | Eukaryotic initiation factor 3,gamma subunit |

| SNP IDs | Chromosomes | Physical positions (bp) | SNPs | Gene accession IDs | Sequence components of genes | Putative functions |
|---|---|---|---|---|---|---|
| CWSNP4065 | Ca-Kabuli-Chr4 | 48502687 | [G/A] | Ca10741 | Intron | Eukaryotic initiation factor 3,gamma subunit |
| CWSNP4066 | Ca-Kabuli-Chr4 | 48678692 | [T/C] | Ca23018 | Synonymous-CDS | Transcription factorIIS,N-terminal |
| CWSNP4067 | Ca-Kabuli-Chr4 | 48688766 | [A/G] | Ca23019 | Synonymous-CDS | C2 calcium-dependent membrane targeting |
| CWSNP4068 | Ca-Kabuli-Chr4 | 48714912 | [G/A] | Ca23021 | Intron | Vacuolar protein sorting-associated protein 35 |
| CWSNP4069 | Ca-Kabuli-Chr4 | 48715028 | [A/G] | Ca23021 | Intron | Vacuolar protein sorting-associated protein 35 |
| CWSNP4070 | Ca-Kabuli-Chr4 | 48720330 | [C/T] | - | URR | - |
| CWSNP4071 | Ca-Kabuli-Chr4 | 48720266 | [C/T] | - | URR | - |
| CWSNP4072 | Ca-Kabuli-Chr4 | 48784151 | [T/G] | - | Intergenic | - |
| CWSNP4073 | Ca-Kabuli-Chr4 | 48784176 | [T/C] | - | Intergenic | - |
| CWSNP4074 | Ca-Kabuli-Chr4 | 48784178 | [C/G] | - | Intergenic | - |
| CWSNP4075 | Ca-Kabuli-Chr4 | 48784179 | [G/A] | - | Intergenic | - |
| CWSNP4076 | Ca-Kabuli-Chr4 | 48784189 | [A/T] | - | Intergenic | - |
| CWSNP4077 | Ca-Kabuli-Chr4 | 48784202 | [A/C] | - | Intergenic | - |
| CWSNP4078 | Ca-Kabuli-Chr4 | 48784204 | [G/C] | - | Intergenic | - |
| CWSNP4079 | Ca-Kabuli-Chr4 | 48936819 | [C/T] | - | URR | - |
| CWSNP4080 | Ca-Kabuli-Chr4 | 49012756 | [C/T] | - | Intergenic | - |

| SNP IDs | Chromosomes | Physical positions (bp) | SNPs | Gene accession IDs | Sequence components of genes | Putative functions |
|---|---|---|---|---|---|---|
| CWSNP4081 | Ca-Kabuli-Chr5 | 131558 | [A/C] | - | Intergenic | - |
| CWSNP4082 | Ca-Kabuli-Chr5 | 154084 | [T/G] | - | Intergenic | - |
| CWSNP4083 | Ca-Kabuli-Chr5 | 154095 | [C/G] | - | Intergenic | - |
| CWSNP4084 | Ca-Kabuli-Chr5 | 154097 | [A/T] | - | Intergenic | - |
| CWSNP4085 | Ca-Kabuli-Chr5 | 154162 | [C/T] | - | Intergenic | - |
| CWSNP4086 | Ca-Kabuli-Chr5 | 154128 | [G/T] | - | Intergenic | - |
| CWSNP4087 | Ca-Kabuli-Chr5 | 166341 | [C/A] | Ca18171 | Non-Synonymous-CDS | No apical meristem (NAM) protein |
| CWSNP4088 | Ca-Kabuli-Chr5 | 166381 | [A/G] | Ca18171 | Synonymous-CDS | No apical meristem (NAM) protein |
| CWSNP4089 | Ca-Kabuli-Chr5 | 210649 | [C/A] | - | Intergenic | - |
| CWSNP4090 | Ca-Kabuli-Chr5 | 222651 | [A/C] | - | Intergenic | - |
| CWSNP4091 | Ca-Kabuli-Chr5 | 222641 | [C/T] | - | Intergenic | - |
| CWSNP4092 | Ca-Kabuli-Chr5 | 222653 | [A/C] | - | Intergenic | - |
| CWSNP4093 | Ca-Kabuli-Chr5 | 222657 | [G/A] | - | Intergenic | - |
| CWSNP4094 | Ca-Kabuli-Chr5 | 223384 | [C/G] | Ca18177 | Synonymous-CDS | ZF-HD homeobox protein,Cys/His-rich dimerisation domain |
| CWSNP4095 | Ca-Kabuli-Chr5 | 234247 | [T/C] | Ca18178 | Intron | Protein of unknown function DUF647 |
| CWSNP4096 | Ca-Kabuli-Chr5 | 234319 | [T/A] | Ca18178 | Intron | Protein of unknown function DUF647 |

| SNP IDs | Chromosomes | Physical positions (bp) | SNPs | Gene accession IDs | Sequence components of genes | Putative functions |
|---|---|---|---|---|---|---|
| CWSNP4097 | Ca-Kabuli-Chr5 | 234311 | [T/A] | Ca18178 | Intron | Protein of unknown function DUF647 |
| CWSNP4098 | Ca-Kabuli-Chr5 | 234305 | [T/A] | Ca18178 | Intron | Protein of unknown function DUF647 |
| CWSNP4099 | Ca-Kabuli-Chr5 | 234303 | [C/A] | Ca18178 | Intron | Protein of unknown function DUF647 |
| CWSNP4100 | Ca-Kabuli-Chr5 | 234410 | [A/C] | Ca18178 | Non-Synonymous-CDS | Protein of unknown function DUF647 |
| CWSNP4101 | Ca-Kabuli-Chr5 | 245850 | [T/G] | Ca18179 | Intron | HEAT |
| CWSNP4102 | Ca-Kabuli-Chr5 | 245863 | [A/T] | Ca18179 | Intron | HEAT |
| CWSNP4103 | Ca-Kabuli-Chr5 | 245896 | [A/T] | Ca18179 | Intron | HEAT |
| CWSNP4104 | Ca-Kabuli-Chr5 | 246466 | [A/T] | Ca18179 | Intron | HEAT |
| CWSNP4105 | Ca-Kabuli-Chr5 | 272972 | [T/A] | Ca18181 | Intron | Mitoticcheckpoint |
| CWSNP4106 | Ca-Kabuli-Chr5 | 277040 | [G/A] | Ca18181 | Synonymous-CDS | Mitoticcheckpoint |
| CWSNP4107 | Ca-Kabuli-Chr5 | 438448 | [C/A] | Ca18196 | Intron | - |
| CWSNP4108 | Ca-Kabuli-Chr5 | 438445 | [C/A] | Ca18196 | Intron | - |
| CWSNP4109 | Ca-Kabuli-Chr5 | 438442 | [T/C] | Ca18196 | Intron | - |
| CWSNP4110 | Ca-Kabuli-Chr5 | 443309 | [A/T] | - | URR | - |
| CWSNP4111 | Ca-Kabuli-Chr5 | 469397 | [G/A] | Ca18200 | Synonymous-CDS | Inositol polyphosphate-related phosphatase |
| CWSNP4112 | Ca-Kabuli-Chr5 | 805859 | [C/T] | - | Intergenic | - |

| SNP IDs | Chromosomes | Physical positions (bp) | SNPs | Gene accession IDs | Sequence components of genes | Putative functions |
|---|---|---|---|---|---|---|
| CWSNP4113 | Ca-Kabuli-Chr5 | 870867 | [A/G] | - | Intergenic | - |
| CWSNP4114 | Ca-Kabuli-Chr5 | 870909 | [G/A] | - | Intergenic | - |
| CWSNP4115 | Ca-Kabuli-Chr5 | 925554 | [T/C] | - | Intergenic | - |
| CWSNP4116 | Ca-Kabuli-Chr5 | 1141755 | [C/T] | Ca23234 | Non-Synonymous-CDS | - |
| CWSNP4117 | Ca-Kabuli-Chr5 | 1220867 | [A/G] | Ca26284 | Intron | YbaK/aminoacyl-tRNA synthetase-associated domain |
| CWSNP4118 | Ca-Kabuli-Chr5 | 1252388 | [A/T] | Ca26282 | Intron | MORNmotif |
| CWSNP4119 | Ca-Kabuli-Chr5 | 1270830 | [T/C] | Ca26280 | Synonymous-CDS | 1-aminocyclopropane-1-carboxylatesynthase |
| CWSNP4120 | Ca-Kabuli-Chr5 | 1272581 | [T/C] | - | Intergenic | - |
| CWSNP4121 | Ca-Kabuli-Chr5 | 1280977 | [T/G] | Ca26279 | Non-Synonymous-CDS | Transcription factor GRAS |
| CWSNP4122 | Ca-Kabuli-Chr5 | 1281017 | [G/A] | Ca26279 | Synonymous-CDS | Transcription factor GRAS |
| CWSNP4123 | Ca-Kabuli-Chr5 | 1281021 | [C/T] | Ca26279 | Synonymous-CDS | Transcription factor GRAS |
| CWSNP4124 | Ca-Kabuli-Chr5 | 2519977 | [C/T] | - | DRR | - |
| CWSNP4125 | Ca-Kabuli-Chr5 | 2519949 | [C/T] | - | DRR | - |
| CWSNP4126 | Ca-Kabuli-Chr5 | 2519961 | [G/A] | - | DRR | - |
| CWSNP4127 | Ca-Kabuli-Chr5 | 2520058 | [C/T] | - | DRR | - |
| CWSNP4128 | Ca-Kabuli-Chr5 | 2520015 | [C/G] | - | DRR | - |

| SNP IDs | Chromosomes | Physical positions (bp) | SNPs | Gene accession IDs | Sequence components of genes | Putative functions |
|---|---|---|---|---|---|---|
| CWSNP4129 | Ca-Kabuli-Chr5 | 2520049 | [C/T] | - | DRR | - |
| CWSNP4130 | Ca-Kabuli-Chr5 | 2520050 | [G/C] | - | DRR | - |
| CWSNP4131 | Ca-Kabuli-Chr5 | 2520051 | [G/T] | - | DRR | - |
| CWSNP4132 | Ca-Kabuli-Chr5 | 2520055 | [C/T] | - | DRR | - |
| CWSNP4133 | Ca-Kabuli-Chr5 | 2520068 | [T/A] | - | DRR | - |
| CWSNP4134 | Ca-Kabuli-Chr5 | 2520069 | [C/T] | - | DRR | - |
| CWSNP4135 | Ca-Kabuli-Chr5 | 2520094 | [G/A] | - | DRR | - |
| CWSNP4136 | Ca-Kabuli-Chr5 | 2520061 | [C/T] | - | DRR | - |
| CWSNP4137 | Ca-Kabuli-Chr5 | 2520093 | [G/A] | - | DRR | - |
| CWSNP4138 | Ca-Kabuli-Chr5 | 2520114 | [G/A] | - | DRR | - |
| CWSNP4139 | Ca-Kabuli-Chr5 | 2520109 | [G/A] | - | DRR | - |
| CWSNP4140 | Ca-Kabuli-Chr5 | 2520101 | [C/T] | - | DRR | - |
| CWSNP4141 | Ca-Kabuli-Chr5 | 2613423 | [T/G] | - | Intergenic | - |
| CWSNP4142 | Ca-Kabuli-Chr5 | 2948530 | [G/T] | Ca18681 | Intron | - |
| CWSNP4143 | Ca-Kabuli-Chr5 | 2948531 | [T/C] | Ca18681 | Intron | - |
| CWSNP4144 | Ca-Kabuli-Chr5 | 2948549 | [C/A] | Ca18681 | Intron | - |

| SNP IDs | Chromosomes | Physical positions (bp) | SNPs | Gene accession IDs | Sequence components of genes | Putative functions |
|---|---|---|---|---|---|---|
| CWSNP4145 | Ca-Kabuli-Chr5 | 2948592 | [T/C] | Ca18681 | Intron | - |
| CWSNP4146 | Ca-Kabuli-Chr5 | 2948616 | [G/A] | Ca18681 | Intron | - |
| CWSNP4147 | Ca-Kabuli-Chr5 | 3348310 | [A/G] | - | Intergenic | - |
| CWSNP4148 | Ca-Kabuli-Chr5 | 3348366 | [G/C] | - | Intergenic | - |
| CWSNP4149 | Ca-Kabuli-Chr5 | 3353052 | [A/G] | - | Intergenic | - |
| CWSNP4150 | Ca-Kabuli-Chr5 | 4122395 | [T/C] | - | Intergenic | - |
| CWSNP4151 | Ca-Kabuli-Chr5 | 4122342 | [T/C] | - | Intergenic | - |
| CWSNP4152 | Ca-Kabuli-Chr5 | 5133644 | [A/G] | Ca25551 | Intron | Zinc finger,U1-type |
| CWSNP4153 | Ca-Kabuli-Chr5 | 5404261 | [G/A] | - | Intergenic | - |
| CWSNP4154 | Ca-Kabuli-Chr5 | 6190494 | [T/C] | - | Intergenic | - |
| CWSNP4155 | Ca-Kabuli-Chr5 | 6225426 | [T/A] | - | Intergenic | - |
| CWSNP4156 | Ca-Kabuli-Chr5 | 6225415 | [G/C] | - | Intergenic | - |
| CWSNP4157 | Ca-Kabuli-Chr5 | 6225410 | [T/A] | - | Intergenic | - |
| CWSNP4158 | Ca-Kabuli-Chr5 | 6290453 | [G/A] | Ca20739 | Intron | RNA recognition motif domain |
| CWSNP4159 | Ca-Kabuli-Chr5 | 6300337 | [C/A] | Ca20739 | Intron | RNA recognition motif domain |
| CWSNP4160 | Ca-Kabuli-Chr5 | 6300502 | [C/A] | Ca20739 | Intron | RNA recognition motif domain |

| SNP IDs | Chromosomes | Physical positions (bp) | SNPs | Gene accession IDs | Sequence components of genes | Putative functions |
|---|---|---|---|---|---|---|
| CWSNP4161 | Ca-Kabuli-Chr5 | 6317014 | [C/T] | - | DRR | - |
| CWSNP4162 | Ca-Kabuli-Chr5 | 6931865 | [A/G] | - | Intergenic | - |
| CWSNP4163 | Ca-Kabuli-Chr5 | 7425116 | [C/T] | - | Intergenic | - |
| CWSNP4164 | Ca-Kabuli-Chr5 | 7664421 | [T/C] | Ca18734 | Non-Synonymous-CDS | Regulator of nonsense-mediated decay, UPF3 |
| CWSNP4165 | Ca-Kabuli-Chr5 | 7775298 | [A/T] | - | Intergenic | - |
| CWSNP4166 | Ca-Kabuli-Chr5 | 7775516 | [A/G] | - | Intergenic | - |
| CWSNP4167 | Ca-Kabuli-Chr5 | 7775497 | [G/C] | - | Intergenic | - |
| CWSNP4168 | Ca-Kabuli-Chr5 | 7775472 | [G/A] | - | Intergenic | - |
| CWSNP4169 | Ca-Kabuli-Chr5 | 7775456 | [T/C] | - | Intergenic | - |
| CWSNP4170 | Ca-Kabuli-Chr5 | 7775918 | [C/T] | - | Intergenic | - |
| CWSNP4171 | Ca-Kabuli-Chr5 | 7789021 | [G/T] | - | Intergenic | - |
| CWSNP4172 | Ca-Kabuli-Chr5 | 7789065 | [A/G] | - | Intergenic | - |
| CWSNP4173 | Ca-Kabuli-Chr5 | 7826338 | [T/G] | - | Intergenic | - |
| CWSNP4174 | Ca-Kabuli-Chr5 | 7826366 | [A/G] | - | Intergenic | - |
| CWSNP4175 | Ca-Kabuli-Chr5 | 8263483 | [A/G] | - | Intergenic | - |
| CWSNP4176 | Ca-Kabuli-Chr5 | 9519382 | [C/A] | - | Intergenic | - |

| SNP IDs | Chromosomes | Physical positions (bp) | SNPs | Gene accession IDs | Sequence components of genes | Putative functions |
|---|---|---|---|---|---|---|
| CWSNP4177 | Ca-Kabuli-Chr5 | 9519390 | [G/A] | - | Intergenic | - |
| CWSNP4178 | Ca-Kabuli-Chr5 | 9620998 | [A/C] | - | DRR | - |
| CWSNP4179 | Ca-Kabuli-Chr5 | 9728591 | [A/G] | - | Intergenic | - |
| CWSNP4180 | Ca-Kabuli-Chr5 | 9868796 | [T/C] | Ca20504 | Intron | Ankyrin repeat |
| CWSNP4181 | Ca-Kabuli-Chr5 | 9869005 | [T/G] | Ca20504 | Synonymous-CDS | Ankyrin repeat |
| CWSNP4182 | Ca-Kabuli-Chr5 | 9961380 | [C/T] | Ca20508 | Non-Synonymous-CDS | DNA-binding WRKY |
| CWSNP4183 | Ca-Kabuli-Chr5 | 9965848 | [T/C] | - | DRR | - |
| CWSNP4184 | Ca-Kabuli-Chr5 | 10004427 | [T/G] | Ca20512 | Synonymous-CDS | - |
| CWSNP4185 | Ca-Kabuli-Chr5 | 10004430 | [T/G] | Ca20512 | Synonymous-CDS | - |
| CWSNP4186 | Ca-Kabuli-Chr5 | 10004451 | [T/G] | Ca20512 | Synonymous-CDS | - |
| CWSNP4187 | Ca-Kabuli-Chr5 | 10072358 | [G/A] | - | Intergenic | - |
| CWSNP4188 | Ca-Kabuli-Chr5 | 10072403 | [G/C] | - | Intergenic | - |
| CWSNP4189 | Ca-Kabuli-Chr5 | 10406562 | [C/A] | - | DRR | - |
| CWSNP4190 | Ca-Kabuli-Chr5 | 10406602 | [C/T] | - | DRR | - |
| CWSNP4191 | Ca-Kabuli-Chr5 | 10406636 | [A/G] | - | DRR | - |
| CWSNP4192 | Ca-Kabuli-Chr5 | 10675252 | [A/G] | - | Intergenic | - |

| SNP IDs | Chromosomes | Physical positions (bp) | SNPs | Gene accession IDs | Sequence components of genes | Putative functions |
|---|---|---|---|---|---|---|
| CWSNP4193 | Ca-Kabuli-Chr5 | 10675279 | [C/G] | - | Intergenic | - |
| CWSNP4194 | Ca-Kabuli-Chr5 | 10675294 | [A/G] | - | Intergenic | - |
| CWSNP4195 | Ca-Kabuli-Chr5 | 10858490 | [C/T] | - | Intergenic | - |
| CWSNP4196 | Ca-Kabuli-Chr5 | 10918368 | [A/G] | Ca23750 | Non-Synonymous-CDS | MoeA,C-terminal,domainIV |
| CWSNP4197 | Ca-Kabuli-Chr5 | 11230617 | [C/G] | - | Intergenic | - |
| CWSNP4198 | Ca-Kabuli-Chr5 | 11230627 | [A/G] | - | Intergenic | - |
| CWSNP4199 | Ca-Kabuli-Chr5 | 11230648 | [G/A] | - | Intergenic | - |
| CWSNP4200 | Ca-Kabuli-Chr5 | 11230671 | [T/C] | - | Intergenic | - |
| CWSNP4201 | Ca-Kabuli-Chr5 | 11263571 | [C/G] | - | Intergenic | - |
| CWSNP4202 | Ca-Kabuli-Chr5 | 11263574 | [C/A] | - | Intergenic | - |
| CWSNP4203 | Ca-Kabuli-Chr5 | 11317487 | [C/T] | - | Intergenic | - |
| CWSNP4204 | Ca-Kabuli-Chr5 | 11317502 | [C/A] | - | Intergenic | - |
| CWSNP4205 | Ca-Kabuli-Chr5 | 11385780 | [A/C] | Ca23356 | Non-Synonymous-CDS | RNA recognition motif domain |
| CWSNP4206 | Ca-Kabuli-Chr5 | 11385874 | [A/G] | Ca23356 | Synonymous-CDS | RNA recognition motif domain |
| CWSNP4207 | Ca-Kabuli-Chr5 | 11777112 | [C/T] | - | URR | - |
| CWSNP4208 | Ca-Kabuli-Chr5 | 11853090 | [C/T] | Ca17111 | Intron | ABC transporter, transmembrane domain |

| SNP IDs | Chromosomes | Physical positions (bp) | SNPs | Gene accession IDs | Sequence components of genes | Putative functions |
|---|---|---|---|---|---|---|
| CWSNP4209 | Ca-Kabuli-Chr5 | 11864959 | [T/C] | Ca17110 | Non-Synonymous-CDS | Zinc finger,CCHC-type |
| CWSNP4210 | Ca-Kabuli-Chr5 | 12157678 | [A/G] | - | Intergenic | - |
| CWSNP4211 | Ca-Kabuli-Chr5 | 12157688 | [C/T] | - | Intergenic | - |
| CWSNP4212 | Ca-Kabuli-Chr5 | 12157699 | [G/T] | - | Intergenic | - |
| CWSNP4213 | Ca-Kabuli-Chr5 | 12162160 | [G/T] | - | Intergenic | - |
| CWSNP4214 | Ca-Kabuli-Chr5 | 12480948 | [T/G] | Ca17080 | Intron | Choline/ethanolaminekinase |
| CWSNP4215 | Ca-Kabuli-Chr5 | 12553469 | [A/C] | - | Intergenic | - |
| CWSNP4216 | Ca-Kabuli-Chr5 | 12553470 | [G/C] | - | Intergenic | - |
| CWSNP4217 | Ca-Kabuli-Chr5 | 12590478 | [T/C] | Ca17072 | Synonymous-CDS | Glycerophosphoryldiester phosphodiesterase |
| CWSNP4218 | Ca-Kabuli-Chr5 | 12662138 | [G/T] | Ca17066 | Intron | Protein kinase, catalytic domain |
| CWSNP4219 | Ca-Kabuli-Chr5 | 13982000 | [T/G] | Ca20821 | Non-Synonymous-CDS | Protein kinase, catalytic domain |
| CWSNP4220 | Ca-Kabuli-Chr5 | 13982041 | [C/T] | Ca20821 | Synonymous-CDS | Protein kinase, catalytic domain |
| CWSNP4221 | Ca-Kabuli-Chr5 | 15952395 | [C/T] | - | Intergenic | - |
| CWSNP4222 | Ca-Kabuli-Chr5 | 16227319 | [G/T] | Ca17915 | Synonymous-CDS | PeptidaseC48,SUMO/Sentrin/Ubl1 |
| CWSNP4223 | Ca-Kabuli-Chr5 | 16227353 | [G/A] | Ca17915 | Non-Synonymous-CDS | PeptidaseC48,SUMO/Sentrin/Ubl1 |
| CWSNP4224 | Ca-Kabuli-Chr5 | 16227401 | [G/A] | Ca17915 | Non-Synonymous-CDS | PeptidaseC48,SUMO/Sentrin/Ubl1 |

| SNP IDs | Chromosomes | Physical positions (bp) | SNPs | Gene accession IDs | Sequence components of genes | Putative functions |
|---|---|---|---|---|---|---|
| CWSNP4225 | Ca-Kabuli-Chr5 | 16426575 | [G/A] | - | URR | - |
| CWSNP4226 | Ca-Kabuli-Chr5 | 16426662 | [C/T] | - | URR | - |
| CWSNP4227 | Ca-Kabuli-Chr5 | 16426656 | [A/G] | - | URR | - |
| CWSNP4228 | Ca-Kabuli-Chr5 | 16426642 | [A/G] | - | URR | - |
| CWSNP4229 | Ca-Kabuli-Chr5 | 16751536 | [C/T] | - | DRR | - |
| CWSNP4230 | Ca-Kabuli-Chr5 | 16929903 | [C/G] | Ca17949 | Intron | tRNA-dihydrouridine synthase |
| CWSNP4231 | Ca-Kabuli-Chr5 | 16929897 | [A/G] | Ca17949 | Intron | tRNA-dihydrouridine synthase |
| CWSNP4232 | Ca-Kabuli-Chr5 | 17037918 | [G/T] | - | Intergenic | - |
| CWSNP4233 | Ca-Kabuli-Chr5 | 17044340 | [G/A] | - | DRR | - |
| CWSNP4234 | Ca-Kabuli-Chr5 | 17045235 | [T/C] | - | Intergenic | - |
| CWSNP4235 | Ca-Kabuli-Chr5 | 17045244 | [A/G] | - | Intergenic | - |
| CWSNP4236 | Ca-Kabuli-Chr5 | 17239612 | [T/C] | - | Intergenic | - |
| CWSNP4237 | Ca-Kabuli-Chr5 | 17494714 | [A/G] | - | Intergenic | - |
| CWSNP4238 | Ca-Kabuli-Chr5 | 17494702 | [G/A] | - | Intergenic | - |
| CWSNP4239 | Ca-Kabuli-Chr5 | 17494700 | [T/C] | - | Intergenic | - |
| CWSNP4240 | Ca-Kabuli-Chr5 | 17494697 | [C/T] | - | Intergenic | - |

| SNP IDs | Chromosomes | Physical positions (bp) | SNPs | Gene accession IDs | Sequence components of genes | Putative functions |
|---|---|---|---|---|---|---|
| CWSNP4241 | Ca-Kabuli-Chr5 | 17494672 | [C/T] | - | Intergenic | - |
| CWSNP4242 | Ca-Kabuli-Chr5 | 17494699 | [C/A] | - | Intergenic | - |
| CWSNP4243 | Ca-Kabuli-Chr5 | 18044808 | [C/G] | - | Intergenic | - |
| CWSNP4244 | Ca-Kabuli-Chr5 | 18664706 | [C/T] | Ca24483 | Non-Synonymous-CDS | - |
| CWSNP4245 | Ca-Kabuli-Chr5 | 18682788 | [A/C] | - | Intergenic | - |
| CWSNP4246 | Ca-Kabuli-Chr5 | 18682792 | [T/C] | - | Intergenic | - |
| CWSNP4247 | Ca-Kabuli-Chr5 | 18682793 | [A/G] | - | Intergenic | - |
| CWSNP4248 | Ca-Kabuli-Chr5 | 18682794 | [A/G] | - | Intergenic | - |
| CWSNP4249 | Ca-Kabuli-Chr5 | 18682812 | [A/G] | - | Intergenic | - |
| CWSNP4250 | Ca-Kabuli-Chr5 | 18682814 | [T/A] | - | Intergenic | - |
| CWSNP4251 | Ca-Kabuli-Chr5 | 18682822 | [A/G] | - | Intergenic | - |
| CWSNP4252 | Ca-Kabuli-Chr5 | 18682827 | [C/G] | - | Intergenic | - |
| CWSNP4253 | Ca-Kabuli-Chr5 | 18682830 | [G/A] | - | Intergenic | - |
| CWSNP4254 | Ca-Kabuli-Chr5 | 18684334 | [C/T] | - | Intergenic | - |
| CWSNP4255 | Ca-Kabuli-Chr5 | 18971085 | [G/A] | - | DRR | - |
| CWSNP4256 | Ca-Kabuli-Chr5 | 18971088 | [C/A] | - | DRR | - |

| SNP IDs | Chromosomes | Physical positions (bp) | SNPs | Gene accession IDs | Sequence components of genes | Putative functions |
|---|---|---|---|---|---|---|
| CWSNP4257 | Ca-Kabuli-Chr5 | 19421820 | [C/T] | Ca23514 | Non-Synonymous-CDS | PeptidaseC48,SUMO/Sentrin/Ubl1 |
| CWSNP4258 | Ca-Kabuli-Chr5 | 19421868 | [C/T] | Ca23514 | Non-Synonymous-CDS | PeptidaseC48,SUMO/Sentrin/Ubl1 |
| CWSNP4259 | Ca-Kabuli-Chr5 | 19828124 | [G/A] | - | Intergenic | - |
| CWSNP4260 | Ca-Kabuli-Chr5 | 19828151 | [C/A] | - | Intergenic | - |
| CWSNP4261 | Ca-Kabuli-Chr5 | 19828163 | [C/T] | - | Intergenic | - |
| CWSNP4262 | Ca-Kabuli-Chr5 | 19828166 | [C/T] | - | Intergenic | - |
| CWSNP4263 | Ca-Kabuli-Chr5 | 19828167 | [A/C] | - | Intergenic | - |
| CWSNP4264 | Ca-Kabuli-Chr5 | 19828226 | [G/A] | - | Intergenic | - |
| CWSNP4265 | Ca-Kabuli-Chr5 | 19828190 | [T/C] | - | Intergenic | - |
| CWSNP4266 | Ca-Kabuli-Chr5 | 19828172 | [T/G] | - | Intergenic | - |
| CWSNP4267 | Ca-Kabuli-Chr5 | 19884132 | [C/T] | Ca22815 | Intron | Vps16,C-terminal |
| CWSNP4268 | Ca-Kabuli-Chr5 | 19884124 | [A/G] | Ca22815 | Intron | Vps16,C-terminal |
| CWSNP4269 | Ca-Kabuli-Chr5 | 20354352 | [T/C] | - | Intergenic | - |
| CWSNP4270 | Ca-Kabuli-Chr5 | 20460859 | [T/G] | Ca18208 | Non-Synonymous-CDS | TetratricopeptideTPR-1 |
| CWSNP4271 | Ca-Kabuli-Chr5 | 20546402 | [T/C] | - | DRR | - |
| CWSNP4272 | Ca-Kabuli-Chr5 | 20546385 | [T/G] | - | DRR | - |

| SNP IDs | Chromosomes | Physical positions (bp) | SNPs | Gene accession IDs | Sequence components of genes | Putative functions |
|---|---|---|---|---|---|---|
| CWSNP4273 | Ca-Kabuli-Chr5 | 20644984 | [C/A] | Ca18219 | Non-Synonymous-CDS | Amidase |
| CWSNP4274 | Ca-Kabuli-Chr5 | 21163371 | [G/A] | - | DRR | - |
| CWSNP4275 | Ca-Kabuli-Chr5 | 21385948 | [T/C] | Ca18243 | Synonymous-CDS | Signal transduction response regulator,receiver domain |
| CWSNP4276 | Ca-Kabuli-Chr5 | 21495583 | [C/A] | - | URR | - |
| CWSNP4277 | Ca-Kabuli-Chr5 | 21495631 | [C/T] | - | URR | - |
| CWSNP4278 | Ca-Kabuli-Chr5 | 21495672 | [G/A] | - | URR | - |
| CWSNP4279 | Ca-Kabuli-Chr5 | 21495707 | [A/G] | - | URR | - |
| CWSNP4280 | Ca-Kabuli-Chr5 | 21495679 | [G/A] | - | URR | - |
| CWSNP4281 | Ca-Kabuli-Chr5 | 21495674 | [G/A] | - | URR | - |
| CWSNP4282 | Ca-Kabuli-Chr5 | 22065518 | [C/T] | Ca21161 | Intron | - |
| CWSNP4283 | Ca-Kabuli-Chr5 | 22065859 | [G/A] | Ca21161 | Synonymous-CDS | - |
| CWSNP4284 | Ca-Kabuli-Chr5 | 22116961 | [T/G] | - | DRR | - |
| CWSNP4285 | Ca-Kabuli-Chr5 | 22302350 | [A/C] | Ca21151 | Synonymous-CDS | Ribosome60SbiogenesisN-terminal |
| CWSNP4286 | Ca-Kabuli-Chr5 | 22344868 | [A/G] | Ca21149 | Synonymous-CDS | - |
| CWSNP4287 | Ca-Kabuli-Chr5 | 22679380 | [C/A] | - | Intergenic | - |
| CWSNP4288 | Ca-Kabuli-Chr5 | 23049683 | [C/A] | - | Intergenic | - |

| SNP IDs | Chromosomes | Physical positions (bp) | SNPs | Gene accession IDs | Sequence components of genes | Putative functions |
|---|---|---|---|---|---|---|
| CWSNP4289 | Ca-Kabuli-Chr5 | 23161492 | [A/T] | Ca21977 | Synonymous-CDS | Zinc finger,PHD-type |
| CWSNP4290 | Ca-Kabuli-Chr5 | 23562595 | [G/A] | Ca17690 | Non-Synonymous-CDS | - |
| CWSNP4291 | Ca-Kabuli-Chr5 | 23562827 | [G/A] | Ca17690 | Intron | - |
| CWSNP4292 | Ca-Kabuli-Chr5 | 23674814 | [C/G] | - | Intergenic | - |
| CWSNP4293 | Ca-Kabuli-Chr5 | 23674815 | [C/T] | - | Intergenic | - |
| CWSNP4294 | Ca-Kabuli-Chr5 | 23674845 | [G/C] | - | Intergenic | - |
| CWSNP4295 | Ca-Kabuli-Chr5 | 24090533 | [C/T] | - | Intergenic | - |
| CWSNP4296 | Ca-Kabuli-Chr5 | 24090523 | [A/C] | - | Intergenic | - |
| CWSNP4297 | Ca-Kabuli-Chr5 | 24090515 | [A/C] | - | Intergenic | - |
| CWSNP4298 | Ca-Kabuli-Chr5 | 24090749 | [A/C] | - | Intergenic | - |
| CWSNP4299 | Ca-Kabuli-Chr5 | 24153205 | [T/C] | Ca17653 | Non-Synonymous-CDS | Protein of unknown function DUF3437 |
| CWSNP4300 | Ca-Kabuli-Chr5 | 24153273 | [T/C] | Ca17653 | Non-Synonymous-CDS | Protein of unknown function DUF3437 |
| CWSNP4301 | Ca-Kabuli-Chr5 | 24153202 | [A/C] | Ca17653 | Non-Synonymous-CDS | Protein of unknown function DUF3437 |
| CWSNP4302 | Ca-Kabuli-Chr5 | 24153199 | [C/T] | Ca17653 | Non-Synonymous-CDS | Protein of unknown function DUF3437 |
| CWSNP4303 | Ca-Kabuli-Chr5 | 24166103 | [C/T] | Ca17652 | Intron | Protein of unknown function DUF3437 |
| CWSNP4304 | Ca-Kabuli-Chr5 | 24166095 | [G/A] | Ca17652 | Intron | Protein of unknown function DUF3437 |

| SNP IDs | Chromosomes | Physical positions (bp) | SNPs | Gene accession IDs | Sequence components of genes | Putative functions |
|---|---|---|---|---|---|---|
| CWSNP4305 | Ca-Kabuli-Chr5 | 24166046 | [G/A] | Ca17652 | Intron | Protein of unknown function DUF3437 |
| CWSNP4306 | Ca-Kabuli-Chr5 | 24166047 | [C/T] | Ca17652 | Intron | Protein of unknown function DUF3437 |
| CWSNP4307 | Ca-Kabuli-Chr5 | 24166082 | [G/A] | Ca17652 | Intron | Protein of unknown function DUF3437 |
| CWSNP4308 | Ca-Kabuli-Chr5 | 24181232 | [C/A] | Ca17652 | Intron | Protein of unknown function DUF3437 |
| CWSNP4309 | Ca-Kabuli-Chr5 | 24181228 | [C/A] | Ca17652 | Intron | Protein of unknown function DUF3437 |
| CWSNP4310 | Ca-Kabuli-Chr5 | 24245312 | [C/A] | Ca15567 | Intron | Protein of unknown function DUF3437 |
| CWSNP4311 | Ca-Kabuli-Chr5 | 24245308 | [C/A] | Ca15567 | Intron | Protein of unknown function DUF3437 |
| CWSNP4312 | Ca-Kabuli-Chr5 | 24576960 | [C/T] | - | Intergenic | - |
| CWSNP4313 | Ca-Kabuli-Chr5 | 24576970 | [A/G] | - | Intergenic | - |
| CWSNP4314 | Ca-Kabuli-Chr5 | 24576993 | [T/G] | - | Intergenic | - |
| CWSNP4315 | Ca-Kabuli-Chr5 | 24843015 | [G/T] | - | Intergenic | - |
| CWSNP4316 | Ca-Kabuli-Chr5 | 25399115 | [T/G] | Ca09042 | Synonymous-CDS | Zinc finger,CCCH-type |
| CWSNP4317 | Ca-Kabuli-Chr5 | 25678975 | [G/T] | - | DRR | - |
| CWSNP4318 | Ca-Kabuli-Chr5 | 25697487 | [C/G] | - | URR | - |
| CWSNP4319 | Ca-Kabuli-Chr5 | 25805020 | [A/G] | Ca09020 | Intron | PeptidaseC48,SUMO/Sentrin/Ubl1 |
| CWSNP4320 | Ca-Kabuli-Chr5 | 26025356 | [A/T] | - | Intergenic | - |

| SNP IDs | Chromosomes | Physical positions (bp) | SNPs | Gene accession IDs | Sequence components of genes | Putative functions |
|---|---|---|---|---|---|---|
| CWSNP4321 | Ca-Kabuli-Chr5 | 26124240 | [T/C] | Ca09005 | Intron | - |
| CWSNP4322 | Ca-Kabuli-Chr5 | 26384636 | [C/G] | - | DRR | - |
| CWSNP4323 | Ca-Kabuli-Chr5 | 26474910 | [A/C] | Ca08972 | Intron | - |
| CWSNP4324 | Ca-Kabuli-Chr5 | 26496197 | [A/G] | Ca08970 | Non-Synonymous-CDS | Aminotransferase class-III |
| CWSNP4325 | Ca-Kabuli-Chr5 | 26626855 | [T/G] | Ca08960 | Non-Synonymous-CDS | Aminoacyl-tRNA synthetase,classI,conserved site |
| CWSNP4326 | Ca-Kabuli-Chr5 | 26679836 | [C/T] | - | DRR | - |
| CWSNP4327 | Ca-Kabuli-Chr5 | 26696412 | [C/A] | - | URR | - |
| CWSNP4328 | Ca-Kabuli-Chr5 | 26696547 | [C/T] | - | URR | - |
| CWSNP4329 | Ca-Kabuli-Chr5 | 26696533 | [C/T] | - | URR | - |
| CWSNP4330 | Ca-Kabuli-Chr5 | 26696578 | [G/A] | - | URR | - |
| CWSNP4331 | Ca-Kabuli-Chr5 | 26696624 | [G/A] | - | URR | - |
| CWSNP4332 | Ca-Kabuli-Chr5 | 26785140 | [G/A] | - | URR | - |
| CWSNP4333 | Ca-Kabuli-Chr5 | 26997335 | [T/G] | - | DRR | - |
| CWSNP4334 | Ca-Kabuli-Chr5 | 26999793 | [C/T] | - | Intergenic | - |
| CWSNP4335 | Ca-Kabuli-Chr5 | 27000127 | [C/G] | - | Intergenic | - |
| CWSNP4336 | Ca-Kabuli-Chr5 | 27000104 | [C/A] | - | Intergenic | - |

| SNP IDs | Chromosomes | Physical positions (bp) | SNPs | Gene accession IDs | Sequence components of genes | Putative functions |
|---|---|---|---|---|---|---|
| CWSNP4337 | Ca-Kabuli-Chr5 | 27000278 | [T/A] | - | Intergenic | - |
| CWSNP4338 | Ca-Kabuli-Chr5 | 27000280 | [T/G] | - | Intergenic | - |
| CWSNP4339 | Ca-Kabuli-Chr5 | 27117953 | [A/G] | Ca08929 | Intron | Basic-leucine zipper (bZIP) Transcription factor |
| CWSNP4340 | Ca-Kabuli-Chr5 | 27150550 | [A/G] | Ca08924 | Intron | Transcriptional factor B3 |
| CWSNP4341 | Ca-Kabuli-Chr5 | 27334056 | [A/C] | - | DRR | - |
| CWSNP4342 | Ca-Kabuli-Chr5 | 27334057 | [G/C] | - | DRR | - |
| CWSNP4343 | Ca-Kabuli-Chr5 | 27334061 | [G/C] | - | DRR | - |
| CWSNP4344 | Ca-Kabuli-Chr5 | 27361579 | [T/G] | Ca08907 | Synonymous-CDS | BTB/POZ-like |
| CWSNP4345 | Ca-Kabuli-Chr5 | 27362570 | [G/T] | Ca08907 | Non-Synonymous-CDS | BTB/POZ-like |
| CWSNP4346 | Ca-Kabuli-Chr5 | 27362573 | [A/G] | Ca08907 | Non-Synonymous-CDS | BTB/POZ-like |
| CWSNP4347 | Ca-Kabuli-Chr5 | 27362584 | [T/G] | Ca08907 | Synonymous-CDS | BTB/POZ-like |
| CWSNP4348 | Ca-Kabuli-Chr5 | 27452484 | [C/G] | Ca08906 | Synonymous-CDS | Protein kinase, catalytic domain |
| CWSNP4349 | Ca-Kabuli-Chr5 | 27552106 | [A/C] | - | URR | - |
| CWSNP4350 | Ca-Kabuli-Chr5 | 27568095 | [C/A] | Ca08898 | Non-Synonymous-CDS | - |
| CWSNP4351 | Ca-Kabuli-Chr5 | 27568064 | [A/C] | Ca08898 | Non-Synonymous-CDS | - |
| CWSNP4352 | Ca-Kabuli-Chr5 | 27568059 | [C/T] | Ca08898 | Non-Synonymous-CDS | - |

| SNP IDs | Chromosomes | Physical positions (bp) | SNPs | Gene accession IDs | Sequence components of genes | Putative functions |
|---|---|---|---|---|---|---|
| CWSNP4353 | Ca-Kabuli-Chr5 | 27568028 | [A/T] | Ca08898 | Non-Synonymous-CDS | - |
| CWSNP4354 | Ca-Kabuli-Chr5 | 27568033 | [G/A] | Ca08898 | Synonymous-CDS | - |
| CWSNP4355 | Ca-Kabuli-Chr5 | 27568082 | [G/A] | Ca08898 | Non-Synonymous-CDS | - |
| CWSNP4356 | Ca-Kabuli-Chr5 | 27568097 | [G/A] | Ca08898 | Non-Synonymous-CDS | - |
| CWSNP4357 | Ca-Kabuli-Chr5 | 27568098 | [C/T] | Ca08898 | Non-Synonymous-CDS | - |
| CWSNP4358 | Ca-Kabuli-Chr5 | 27568118 | [A/G] | Ca08898 | Non-Synonymous-CDS | - |
| CWSNP4359 | Ca-Kabuli-Chr5 | 27568133 | [G/T] | Ca08898 | Non-Synonymous-CDS | - |
| CWSNP4360 | Ca-Kabuli-Chr5 | 27568081 | [G/C] | Ca08898 | Synonymous-CDS | - |
| CWSNP4361 | Ca-Kabuli-Chr5 | 27568107 | [C/G] | Ca08898 | Non-Synonymous-CDS | - |
| CWSNP4362 | Ca-Kabuli-Chr5 | 27568156 | [C/G] | Ca08898 | Non-Synonymous-CDS | - |
| CWSNP4363 | Ca-Kabuli-Chr5 | 27568141 | [C/T] | Ca08898 | Synonymous-CDS | - |
| CWSNP4364 | Ca-Kabuli-Chr5 | 27568180 | [C/T] | Ca08898 | Synonymous-CDS | - |
| CWSNP4365 | Ca-Kabuli-Chr5 | 27568176 | [T/C] | Ca08898 | Non-Synonymous-CDS | - |
| CWSNP4366 | Ca-Kabuli-Chr5 | 27605037 | [A/T] | - | Intergenic | - |
| CWSNP4367 | Ca-Kabuli-Chr5 | 27605063 | [T/A] | - | Intergenic | - |
| CWSNP4368 | Ca-Kabuli-Chr5 | 27605081 | [C/T] | - | Intergenic | - |

| SNP IDs | Chromosomes | Physical positions (bp) | SNPs | Gene accession IDs | Sequence components of genes | Putative functions |
|---|---|---|---|---|---|---|
| CWSNP4369 | Ca-Kabuli-Chr5 | 27605095 | [G/C] | - | Intergenic | - |
| CWSNP4370 | Ca-Kabuli-Chr5 | 27692818 | [C/T] | - | Intergenic | - |
| CWSNP4371 | Ca-Kabuli-Chr5 | 27692822 | [C/T] | - | Intergenic | - |
| CWSNP4372 | Ca-Kabuli-Chr5 | 27692823 | [A/T] | - | Intergenic | - |
| CWSNP4373 | Ca-Kabuli-Chr5 | 27692831 | [A/T] | - | Intergenic | - |
| CWSNP4374 | Ca-Kabuli-Chr5 | 27694205 | [C/A] | - | DRR | - |
| CWSNP4375 | Ca-Kabuli-Chr5 | 27786025 | [T/C] | Ca08871 | Intron | - |
| CWSNP4376 | Ca-Kabuli-Chr5 | 27838003 | [T/C] | - | Intergenic | - |
| CWSNP4377 | Ca-Kabuli-Chr5 | 27939162 | [G/A] | - | Intergenic | - |
| CWSNP4378 | Ca-Kabuli-Chr5 | 27939135 | [G/C] | - | Intergenic | - |
| CWSNP4379 | Ca-Kabuli-Chr5 | 27939134 | [A/T] | - | Intergenic | - |
| CWSNP4380 | Ca-Kabuli-Chr5 | 27939128 | [A/G] | - | Intergenic | - |
| CWSNP4381 | Ca-Kabuli-Chr5 | 27974691 | [G/T] | Ca13421 | Intron | ABC-1 |
| CWSNP4382 | Ca-Kabuli-Chr5 | 28016628 | [C/T] | - | DRR | - |
| CWSNP4383 | Ca-Kabuli-Chr5 | 28016669 | [A/G] | - | DRR | - |
| CWSNP4384 | Ca-Kabuli-Chr5 | 28071467 | [A/G] | - | Intergenic | - |

| SNP IDs | Chromosomes | Physical positions (bp) | SNPs | Gene accession IDs | Sequence components of genes | Putative functions |
|---|---|---|---|---|---|---|
| CWSNP4385 | Ca-Kabuli-Chr5 | 28116988 | [A/T] | - | Intergenic | - |
| CWSNP4386 | Ca-Kabuli-Chr5 | 28117011 | [C/T] | - | Intergenic | - |
| CWSNP4387 | Ca-Kabuli-Chr5 | 28164985 | [G/C] | Ca13404 | Intron | Clathrin,heavy chain/VPS,7-fold repeat |
| CWSNP4388 | Ca-Kabuli-Chr5 | 28168910 | [A/G] | - | URR | - |
| CWSNP4389 | Ca-Kabuli-Chr5 | 28169475 | [T/C] | - | URR | - |
| CWSNP4390 | Ca-Kabuli-Chr5 | 28175039 | [A/G] | Ca13403 | Non-Synonymous-CDS | - |
| CWSNP4391 | Ca-Kabuli-Chr5 | 28266046 | [T/G] | Ca13399 | Synonymous-CDS | - |
| CWSNP4392 | Ca-Kabuli-Chr5 | 28271638 | [G/C] | Ca13398 | Intron | Bacterial surface antigen (D15) |
| CWSNP4393 | Ca-Kabuli-Chr5 | 28271732 | [C/T] | Ca13398 | Intron | Bacterial surface antigen (D15) |
| CWSNP4394 | Ca-Kabuli-Chr5 | 28271742 | [G/A] | Ca13398 | Intron | Bacterial surface antigen (D15) |
| CWSNP4395 | Ca-Kabuli-Chr5 | 28271767 | [G/C] | Ca13398 | Intron | Bacterial surface antigen (D15) |
| CWSNP4396 | Ca-Kabuli-Chr5 | 28327832 | [T/G] | - | URR | - |
| CWSNP4397 | Ca-Kabuli-Chr5 | 28327883 | [G/A] | - | URR | - |
| CWSNP4398 | Ca-Kabuli-Chr5 | 28327870 | [T/C] | - | URR | - |
| CWSNP4399 | Ca-Kabuli-Chr5 | 28327868 | [G/A] | - | URR | - |
| CWSNP4400 | Ca-Kabuli-Chr5 | 28327856 | [T/C] | - | URR | - |

| SNP IDs | Chromosomes | Physical positions (bp) | SNPs | Gene accession IDs | Sequence components of genes | Putative functions |
|---|---|---|---|---|---|---|
| CWSNP4401 | Ca-Kabuli-Chr5 | 28335455 | [A/C] | Ca13389 | Synonymous-CDS | Histidine phosphatase superfamily, clade-2 |
| CWSNP4402 | Ca-Kabuli-Chr5 | 28346497 | [C/T] | Ca13388 | Synonymous-CDS | Lateral organ boundaries, LOB |
| CWSNP4403 | Ca-Kabuli-Chr5 | 28360495 | [C/T] | - | Intergenic | - |
| CWSNP4404 | Ca-Kabuli-Chr5 | 28360492 | [C/T] | - | Intergenic | - |
| CWSNP4405 | Ca-Kabuli-Chr5 | 28360585 | [A/G] | - | URR | - |
| CWSNP4406 | Ca-Kabuli-Chr5 | 28383374 | [G/T] | Ca13385 | Intron | Autophagy-relatedprotein 27 |
| CWSNP4407 | Ca-Kabuli-Chr5 | 28562734 | [G/A] | - | Intergenic | - |
| CWSNP4408 | Ca-Kabuli-Chr5 | 28581814 | [A/G] | - | URR | - |
| CWSNP4409 | Ca-Kabuli-Chr5 | 28581815 | [C/G] | - | URR | - |
| CWSNP4410 | Ca-Kabuli-Chr5 | 28581827 | [T/C] | - | URR | - |
| CWSNP4411 | Ca-Kabuli-Chr5 | 28581866 | [T/C] | - | URR | - |
| CWSNP4412 | Ca-Kabuli-Chr5 | 28582525 | [C/T] | - | URR | - |
| CWSNP4413 | Ca-Kabuli-Chr5 | 28582806 | [T/C] | - | URR | - |
| CWSNP4414 | Ca-Kabuli-Chr5 | 28582763 | [A/G] | - | URR | - |
| CWSNP4415 | Ca-Kabuli-Chr5 | 28583402 | [G/A] | Ca13367 | Intron | Sel1-like |
| CWSNP4416 | Ca-Kabuli-Chr5 | 28583375 | [C/G] | Ca13367 | Intron | Sel1-like |

| SNP IDs | Chromosomes | Physical positions (bp) | SNPs | Gene accession IDs | Sequence components of genes | Putative functions |
|---|---|---|---|---|---|---|
| CWSNP4417 | Ca-Kabuli-Chr5 | 28717362 | [C/T] | Ca13358 | Non-Synonymous-CDS | Oligopeptide transporter |
| CWSNP4418 | Ca-Kabuli-Chr5 | 28717384 | [C/T] | Ca13358 | Synonymous-CDS | Oligopeptide transporter |
| CWSNP4419 | Ca-Kabuli-Chr5 | 28721236 | [C/T] | - | DRR | - |
| CWSNP4420 | Ca-Kabuli-Chr5 | 28721237 | [A/G] | - | DRR | - |
| CWSNP4421 | Ca-Kabuli-Chr5 | 28721386 | [T/C] | - | DRR | - |
| CWSNP4422 | Ca-Kabuli-Chr5 | 28721383 | [A/C] | - | DRR | - |
| CWSNP4423 | Ca-Kabuli-Chr5 | 28746139 | [A/G] | Ca13355 | Non-Synonymous-CDS | Haloacid dehalogenase-like hydrolase |
| CWSNP4424 | Ca-Kabuli-Chr5 | 28757467 | [A/T] | Ca13354 | Non-Synonymous-CDS | Protein of unknown function DUF827,plant |
| CWSNP4425 | Ca-Kabuli-Chr5 | 28757485 | [A/C] | Ca13354 | Non-Synonymous-CDS | Protein of unknown function DUF827,plant |
| CWSNP4426 | Ca-Kabuli-Chr5 | 28757507 | [A/T] | Ca13354 | Non-Synonymous-CDS | Protein of unknown function DUF827,plant |
| CWSNP4427 | Ca-Kabuli-Chr5 | 28758723 | [A/G] | Ca13354 | Intron | Protein of unknown function DUF827,plant |
| CWSNP4428 | Ca-Kabuli-Chr5 | 28817832 | [A/G] | - | Intergenic | - |
| CWSNP4429 | Ca-Kabuli-Chr5 | 28827467 | [A/G] | - | Intergenic | - |
| CWSNP4430 | Ca-Kabuli-Chr5 | 28854486 | [C/T] | - | Intergenic | - |
| CWSNP4431 | Ca-Kabuli-Chr5 | 28905203 | [A/C] | Ca13344 | Non-Synonymous-CDS | Drug/metabolite transporter |
| CWSNP4432 | Ca-Kabuli-Chr5 | 29007895 | [T/C] | Ca23834 | Synonymous-CDS | Camphor resistance CrcB protein |

| SNP IDs | Chromosomes | Physical positions (bp) | SNPs | Gene accession IDs | Sequence components of genes | Putative functions |
|---|---|---|---|---|---|---|
| CWSNP4433 | Ca-Kabuli-Chr5 | 29007886 | [C/A] | Ca23834 | Synonymous-CDS | Camphor resistance CrcB protein |
| CWSNP4434 | Ca-Kabuli-Chr5 | 29007884 | [A/C] | Ca23834 | Non-Synonymous-CDS | Camphor resistance CrcB protein |
| CWSNP4435 | Ca-Kabuli-Chr5 | 29007882 | [A/C] | Ca23834 | Non-Synonymous-CDS | Camphor resistance CrcB protein |
| CWSNP4436 | Ca-Kabuli-Chr5 | 29007879 | [T/C] | Ca23834 | Non-Synonymous-CDS | Camphor resistance CrcB protein |
| CWSNP4437 | Ca-Kabuli-Chr5 | 29007847 | [A/C] | Ca23834 | Synonymous-CDS | Camphor resistance CrcB protein |
| CWSNP4438 | Ca-Kabuli-Chr5 | 29007844 | [T/C] | Ca23834 | Synonymous-CDS | Camphor resistance CrcB protein |
| CWSNP4439 | Ca-Kabuli-Chr5 | 29080060 | [C/T] | Ca23829 | Synonymous-CDS | Protein kinase, catalytic domain |
| CWSNP4440 | Ca-Kabuli-Chr5 | 29354269 | [A/T] | - | URR | - |
| CWSNP4441 | Ca-Kabuli-Chr5 | 29437139 | [C/T] | - | Intergenic | - |
| CWSNP4442 | Ca-Kabuli-Chr5 | 29437146 | [C/A] | - | Intergenic | - |
| CWSNP4443 | Ca-Kabuli-Chr5 | 29437162 | [A/C] | - | Intergenic | - |
| CWSNP4444 | Ca-Kabuli-Chr5 | 29437171 | [A/T] | - | Intergenic | - |
| CWSNP4445 | Ca-Kabuli-Chr5 | 29437181 | [A/C] | - | Intergenic | - |
| CWSNP4446 | Ca-Kabuli-Chr5 | 29437193 | [A/C] | - | Intergenic | - |
| CWSNP4447 | Ca-Kabuli-Chr5 | 29528904 | [T/G] | Ca16640 | Non-Synonymous-CDS | Sugar/inositol transporter |
| CWSNP4448 | Ca-Kabuli-Chr5 | 29567676 | [T/C] | - | DRR | - |

| SNP IDs | Chromosomes | Physical positions (bp) | SNPs | Gene accession IDs | Sequence components of genes | Putative functions |
|---|---|---|---|---|---|---|
| CWSNP4449 | Ca-Kabuli-Chr5 | 29567820 | [T/C] | - | DRR | - |
| CWSNP4450 | Ca-Kabuli-Chr5 | 29576716 | [A/C] | - | Intergenic | - |
| CWSNP4451 | Ca-Kabuli-Chr5 | 29608657 | [T/C] | - | Intergenic | - |
| CWSNP4452 | Ca-Kabuli-Chr5 | 29641659 | [T/C] | - | DRR | - |
| CWSNP4453 | Ca-Kabuli-Chr5 | 29658280 | [A/C] | Ca16650 | Intron | Protein of unknown function DUF248,methyltransferase putative |
| CWSNP4454 | Ca-Kabuli-Chr5 | 29824218 | [T/G] | - | URR | - |
| CWSNP4455 | Ca-Kabuli-Chr5 | 29999414 | [C/T] | - | Intergenic | - |
| CWSNP4456 | Ca-Kabuli-Chr5 | 29999433 | [C/A] | - | Intergenic | - |
| CWSNP4457 | Ca-Kabuli-Chr5 | 29999465 | [T/G] | - | Intergenic | - |
| CWSNP4458 | Ca-Kabuli-Chr5 | 29999476 | [G/A] | - | Intergenic | - |
| CWSNP4459 | Ca-Kabuli-Chr5 | 30035763 | [A/T] | Ca04680 | Synonymous-CDS | Sugar/inositol transporter |
| CWSNP4460 | Ca-Kabuli-Chr5 | 30049265 | [G/A] | - | URR | - |
| CWSNP4461 | Ca-Kabuli-Chr5 | 30053540 | [C/G] | - | Intergenic | - |
| CWSNP4462 | Ca-Kabuli-Chr5 | 30060160 | [C/T] | - | Intergenic | - |
| CWSNP4463 | Ca-Kabuli-Chr5 | 30086232 | [A/C] | - | URR | - |
| CWSNP4464 | Ca-Kabuli-Chr5 | 30090906 | [A/G] | - | DRR | - |

| SNP IDs | Chromosomes | Physical positions (bp) | SNPs | Gene accession IDs | Sequence components of genes | Putative functions |
|---|---|---|---|---|---|---|
| CWSNP4465 | Ca-Kabuli-Chr5 | 30091063 | [G/A] | - | DRR | - |
| CWSNP4466 | Ca-Kabuli-Chr5 | 30191389 | [A/C] | - | Intergenic | - |
| CWSNP4467 | Ca-Kabuli-Chr5 | 30248447 | [C/A] | - | URR | - |
| CWSNP4468 | Ca-Kabuli-Chr5 | 30367349 | [G/T] | Ca04714 | Non-Synonymous-CDS | HECT |
| CWSNP4469 | Ca-Kabuli-Chr5 | 30373542 | [A/G] | Ca04715 | Non-Synonymous-CDS | Armadillo |
| CWSNP4470 | Ca-Kabuli-Chr5 | 30373783 | [T/A] | Ca04715 | Synonymous-CDS | Armadillo |
| CWSNP4471 | Ca-Kabuli-Chr5 | 30562524 | [G/T] | - | DRR | - |
| CWSNP4472 | Ca-Kabuli-Chr5 | 30632697 | [A/G] | Ca04756 | Intron | - |
| CWSNP4473 | Ca-Kabuli-Chr5 | 30656058 | [A/T] | Ca04757 | Intron | Kinesin , motor domain |
| CWSNP4474 | Ca-Kabuli-Chr5 | 30690891 | [T/G] | - | DRR | - |
| CWSNP4475 | Ca-Kabuli-Chr5 | 30690885 | [A/G] | - | DRR | - |
| CWSNP4476 | Ca-Kabuli-Chr5 | 30691510 | [A/G] | - | DRR | - |
| CWSNP4477 | Ca-Kabuli-Chr5 | 30691515 | [G/A] | - | DRR | - |
| CWSNP4478 | Ca-Kabuli-Chr5 | 30691543 | [A/G] | - | DRR | - |
| CWSNP4479 | Ca-Kabuli-Chr5 | 30697511 | [G/A] | - | DRR | - |
| CWSNP4480 | Ca-Kabuli-Chr5 | 30811250 | [A/T] | - | URR | - |

| SNP IDs | Chromosomes | Physical positions (bp) | SNPs | Gene accession IDs | Sequence components of genes | Putative functions |
|---|---|---|---|---|---|---|
| CWSNP4481 | Ca-Kabuli-Chr5 | 30868695 | [G/C] | - | Intergenic | - |
| CWSNP4482 | Ca-Kabuli-Chr5 | 31025764 | [G/T] | - | Intergenic | - |
| CWSNP4483 | Ca-Kabuli-Chr5 | 31126125 | [C/T] | - | URR | - |
| CWSNP4484 | Ca-Kabuli-Chr5 | 31131830 | [T/C] | Ca04815 | Intron | - |
| CWSNP4485 | Ca-Kabuli-Chr5 | 31132568 | [A/T] | Ca04815 | Synonymous-CDS | - |
| CWSNP4486 | Ca-Kabuli-Chr5 | 31162533 | [G/A] | - | DRR | - |
| CWSNP4487 | Ca-Kabuli-Chr5 | 31164387 | [G/C] | Ca04820 | Intron | - |
| CWSNP4488 | Ca-Kabuli-Chr5 | 31164539 | [T/C] | Ca04820 | Synonymous-CDS | Armadillo |
| CWSNP4489 | Ca-Kabuli-Chr5 | 31165048 | [T/C] | Ca04820 | Synonymous-CDS | Armadillo |
| CWSNP4490 | Ca-Kabuli-Chr5 | 31165039 | [T/C] | Ca04820 | Synonymous-CDS | Armadillo |
| CWSNP4491 | Ca-Kabuli-Chr5 | 31165035 | [T/C] | Ca04820 | Non-Synonymous-CDS | Armadillo |
| CWSNP4492 | Ca-Kabuli-Chr5 | 31165002 | [A/C] | Ca04820 | Non-Synonymous-CDS | Armadillo |
| CWSNP4493 | Ca-Kabuli-Chr5 | 31176844 | [G/A] | Ca04822 | Intron | EPS15 homology (EH) |
| CWSNP4494 | Ca-Kabuli-Chr5 | 31194974 | [T/C] | - | Intergenic | - |
| CWSNP4495 | Ca-Kabuli-Chr5 | 31215109 | [C/T] | - | DRR | - |
| CWSNP4496 | Ca-Kabuli-Chr5 | 31314012 | [A/C] | Ca04834 | Synonymous-CDS | Phox/Bem1p |

| SNP IDs | Chromosomes | Physical positions (bp) | SNPs | Gene accession IDs | Sequence components of genes | Putative functions |
|---|---|---|---|---|---|---|
| CWSNP4497 | Ca-Kabuli-Chr5 | 31314056 | [A/C] | Ca04834 | Synonymous-CDS | Phox/Bem1p |
| CWSNP4498 | Ca-Kabuli-Chr5 | 31390319 | [C/T] | Ca04841 | Intron | - |
| CWSNP4499 | Ca-Kabuli-Chr5 | 31687891 | [A/C] | Ca04871 | Non-Synonymous-CDS | RNA-binding proteinLupusLa |
| CWSNP4500 | Ca-Kabuli-Chr5 | 31750242 | [A/G] | - | DRR | - |
| CWSNP4501 | Ca-Kabuli-Chr5 | 31935674 | [T/C] | Ca04898 | Non-Synonymous-CDS | Protein of unknown function DUF869,plant |
| CWSNP4502 | Ca-Kabuli-Chr5 | 32011781 | [G/T] | Ca04904 | Intron | Kinesin , motor domain |
| CWSNP4503 | Ca-Kabuli-Chr5 | 32011792 | [T/G] | Ca04904 | Intron | Kinesin , motor domain |
| CWSNP4504 | Ca-Kabuli-Chr5 | 32171632 | [G/A] | Ca04917 | Intron | Tetrapyrrolemethylase |
| CWSNP4505 | Ca-Kabuli-Chr5 | 32291158 | [C/T] | Ca04927 | Non-Synonymous-CDS | Aminotransferase-like,plant mobile domain |
| CWSNP4506 | Ca-Kabuli-Chr5 | 32291118 | [G/A] | - | Intergenic | - |
| CWSNP4507 | Ca-Kabuli-Chr5 | 32357511 | [G/A] | Ca04934 | Non-Synonymous-CDS | Pseudouridine synthase/archaeosine transglycosylase |
| CWSNP4508 | Ca-Kabuli-Chr5 | 32368485 | [G/C] | - | DRR | - |
| CWSNP4509 | Ca-Kabuli-Chr5 | 32399391 | [T/A] | - | URR | - |
| CWSNP4510 | Ca-Kabuli-Chr5 | 32516773 | [T/A] | Ca04958 | Intron | - |
| CWSNP4511 | Ca-Kabuli-Chr5 | 32524646 | [T/C] | Ca04959 | Intron | - |
| CWSNP4512 | Ca-Kabuli-Chr5 | 32598221 | [C/A] | - | Intergenic | - |

| SNP IDs | Chromosomes | Physical positions (bp) | SNPs | Gene accession IDs | Sequence components of genes | Putative functions |
|---|---|---|---|---|---|---|
| CWSNP4513 | Ca-Kabuli-Chr5 | 32816626 | [G/T] | - | Intergenic | - |
| CWSNP4514 | Ca-Kabuli-Chr5 | 32840522 | [T/C] | Ca04983 | Non-Synonymous-CDS | Quinate/shikimate5-dehydrogenase/glutamyl-tRNAreductase |
| CWSNP4515 | Ca-Kabuli-Chr5 | 32840541 | [T/G] | Ca04983 | Non-Synonymous-CDS | Quinate/shikimate5-dehydrogenase/glutamyl-tRNAreductase |
| CWSNP4516 | Ca-Kabuli-Chr5 | 32859504 | [T/G] | Ca04985 | Intron | Protein of unknown function DUF3453 |
| CWSNP4517 | Ca-Kabuli-Chr5 | 32859515 | [T/G] | Ca04985 | Intron | Protein of unknown function DUF3453 |
| CWSNP4518 | Ca-Kabuli-Chr5 | 32860087 | [C/T] | Ca04985 | Intron | Protein of unknown function DUF3453 |
| CWSNP4519 | Ca-Kabuli-Chr5 | 32978975 | [A/T] | - | Intergenic | - |
| CWSNP4520 | Ca-Kabuli-Chr5 | 33052329 | [C/G] | - | Intergenic | - |
| CWSNP4521 | Ca-Kabuli-Chr5 | 33255823 | [T/G] | - | DRR | - |
| CWSNP4522 | Ca-Kabuli-Chr5 | 33422813 | [C/T] | - | DRR | - |
| CWSNP4523 | Ca-Kabuli-Chr5 | 33523676 | [T/C] | - | Intergenic | - |
| CWSNP4524 | Ca-Kabuli-Chr5 | 33523686 | [G/A] | - | Intergenic | - |
| CWSNP4525 | Ca-Kabuli-Chr5 | 33532807 | [A/G] | - | URR | - |
| CWSNP4526 | Ca-Kabuli-Chr5 | 33627132 | [T/C] | - | Intergenic | - |
| CWSNP4527 | Ca-Kabuli-Chr5 | 33631108 | [A/G] | - | Intergenic | - |
| CWSNP4528 | Ca-Kabuli-Chr5 | 33631266 | [T/A] | - | Intergenic | - |

| SNP IDs | Chromosomes | Physical positions (bp) | SNPs | Gene accession IDs | Sequence components of genes | Putative functions |
|---|---|---|---|---|---|---|
| CWSNP4529 | Ca-Kabuli-Chr5 | 33673894 | [T/C] | - | URR | - |
| CWSNP4530 | Ca-Kabuli-Chr5 | 33812213 | [A/C] | Ca01876 | Intron | - |
| CWSNP4531 | Ca-Kabuli-Chr5 | 33812363 | [T/A] | Ca01876 | Intron | - |
| CWSNP4532 | Ca-Kabuli-Chr5 | 33818742 | [T/A] | Ca01875 | Non-Synonymous-CDS | RNA recognition motif domain |
| CWSNP4533 | Ca-Kabuli-Chr5 | 33818823 | [A/G] | Ca01875 | Non-Synonymous-CDS | RNA recognition motif domain |
| CWSNP4534 | Ca-Kabuli-Chr5 | 33892596 | [A/C] | Ca01864 | Non-Synonymous-CDS | Proteasome component (PCI) domain |
| CWSNP4535 | Ca-Kabuli-Chr5 | 33937307 | [G/A] | - | DRR | - |
| CWSNP4536 | Ca-Kabuli-Chr5 | 33937316 | [G/A] | - | DRR | - |
| CWSNP4537 | Ca-Kabuli-Chr5 | 33963525 | [G/C] | - | Intergenic | - |
| CWSNP4538 | Ca-Kabuli-Chr5 | 33986253 | [A/C] | Ca01852 | Synonymous-CDS | Protein kinase, catalytic domain |
| CWSNP4539 | Ca-Kabuli-Chr5 | 34202075 | [T/G] | Ca01827 | Non-Synonymous-CDS | Peptidase C19,ubiquitin carboxyl-terminalhydrolase 2 |
| CWSNP4540 | Ca-Kabuli-Chr5 | 34441581 | [A/C] | Ca01800 | Non-Synonymous-CDS | Transcription elongation factorS-II,centraldomain |
| CWSNP4541 | Ca-Kabuli-Chr5 | 34531222 | [A/G] | Ca01787 | Intron | ABC transporter-like |
| CWSNP4542 | Ca-Kabuli-Chr5 | 34535758 | [T/C] | Ca01787 | Non-Synonymous-CDS | ABC transporter-like |
| CWSNP4543 | Ca-Kabuli-Chr5 | 34535893 | [G/T] | - | Intergenic | - |
| CWSNP4544 | Ca-Kabuli-Chr5 | 34824749 | [A/T] | - | Intergenic | - |

| SNP IDs | Chromosomes | Physical positions (bp) | SNPs | Gene accession IDs | Sequence components of genes | Putative functions |
|---|---|---|---|---|---|---|
| CWSNP4545 | Ca-Kabuli-Chr5 | 34845398 | [T/G] | - | DRR | - |
| CWSNP4546 | Ca-Kabuli-Chr5 | 34974703 | [C/T] | - | DRR | - |
| CWSNP4547 | Ca-Kabuli-Chr5 | 35191371 | [C/G] | Ca01716 | Non-Synonymous-CDS | Proteasome component (PCI) domain |
| CWSNP4548 | Ca-Kabuli-Chr5 | 35500751 | [T/C] | Ca01682 | Synonymous-CDS | Sterilealphamotifdomain |
| CWSNP4549 | Ca-Kabuli-Chr5 | 35555184 | [A/G] | - | Intergenic | - |
| CWSNP4550 | Ca-Kabuli-Chr5 | 35617798 | [A/C] | - | DRR | - |
| CWSNP4551 | Ca-Kabuli-Chr5 | 35690996 | [G/A] | - | URR | - |
| CWSNP4552 | Ca-Kabuli-Chr5 | 35715571 | [A/C] | - | Intergenic | - |
| CWSNP4553 | Ca-Kabuli-Chr5 | 35715548 | [T/C] | - | Intergenic | - |
| CWSNP4554 | Ca-Kabuli-Chr5 | 35773044 | [A/G] | - | URR | - |
| CWSNP4555 | Ca-Kabuli-Chr5 | 35789373 | [T/A] | Ca01646 | Intron | Protein kinase, catalytic domain |
| CWSNP4556 | Ca-Kabuli-Chr5 | 35839233 | [G/A] | Ca01642 | Synonymous-CDS | Mitochondrial carrier protein |
| CWSNP4557 | Ca-Kabuli-Chr5 | 35844295 | [G/T] | - | DRR | - |
| CWSNP4558 | Ca-Kabuli-Chr5 | 35877505 | [T/C] | Ca01638 | Synonymous-CDS | Mitochondrial carrier protein |
| CWSNP4559 | Ca-Kabuli-Chr5 | 35937842 | [G/T] | Ca01631 | Non-Synonymous-CDS | NADH:ubiquinone oxidoreductase-like,20 kDa subunit |
| CWSNP4560 | Ca-Kabuli-Chr5 | 36128655 | [G/T] | Ca01611 | Intron | - |

| SNP IDs | Chromosomes | Physical positions (bp) | SNPs | Gene accession IDs | Sequence components of genes | Putative functions |
|---|---|---|---|---|---|---|
| CWSNP4561 | Ca-Kabuli-Chr5 | 36131156 | [A/C] | Ca01611 | Intron | - |
| CWSNP4562 | Ca-Kabuli-Chr5 | 36238656 | [G/A] | Ca01601 | Intron | ATPase,P-type,K/Mg/Cd/Cu/Zn/Na/Ca/Na/H-transporter |
| CWSNP4563 | Ca-Kabuli-Chr5 | 36238655 | [G/C] | Ca01601 | Intron | ATPase,P-type,K/Mg/Cd/Cu/Zn/Na/Ca/Na/H-transporter |
| CWSNP4564 | Ca-Kabuli-Chr5 | 36408719 | [G/A] | - | Intergenic | - |
| CWSNP4565 | Ca-Kabuli-Chr5 | 36534056 | [G/C] | Ca01566 | Intron | MRG |
| CWSNP4566 | Ca-Kabuli-Chr5 | 36534063 | [C/T] | Ca01566 | Intron | MRG |
| CWSNP4567 | Ca-Kabuli-Chr5 | 36619837 | [A/G] | Ca01558 | Intron | Kinesin , motor domain |
| CWSNP4568 | Ca-Kabuli-Chr5 | 36621872 | [A/T] | Ca01558 | Non-Synonymous-CDS | Kinesin , motor domain |
| CWSNP4569 | Ca-Kabuli-Chr5 | 36672147 | [C/T] | Ca01555 | Non-Synonymous-CDS | RNA recognition motif domain |
| CWSNP4570 | Ca-Kabuli-Chr5 | 36673950 | [C/A] | - | URR | - |
| CWSNP4571 | Ca-Kabuli-Chr5 | 36735459 | [A/T] | Ca01550 | Non-Synonymous-CDS | Helix-loop-helix DNA-binding domain |
| CWSNP4572 | Ca-Kabuli-Chr5 | 36751625 | [C/T] | - | Intergenic | - |
| CWSNP4573 | Ca-Kabuli-Chr5 | 36751851 | [T/A] | - | Intergenic | - |
| CWSNP4574 | Ca-Kabuli-Chr5 | 36757408 | [G/A] | - | Intergenic | - |
| CWSNP4575 | Ca-Kabuli-Chr5 | 36797187 | [G/T] | - | DRR | - |
| CWSNP4576 | Ca-Kabuli-Chr5 | 36999074 | [G/C] | Ca01518 | Non-Synonymous-CDS | Protein kinase, catalytic domain |

| SNP IDs | Chromosomes | Physical positions (bp) | SNPs | Gene accession IDs | Sequence components of genes | Putative functions |
|---|---|---|---|---|---|---|
| CWSNP4577 | Ca-Kabuli-Chr5 | 37016704 | [C/G] | - | DRR | - |
| CWSNP4578 | Ca-Kabuli-Chr5 | 37039246 | [A/T] | - | URR | - |
| CWSNP4579 | Ca-Kabuli-Chr5 | 37039300 | [A/G] | - | URR | - |
| CWSNP4580 | Ca-Kabuli-Chr5 | 37040045 | [G/A] | - | URR | - |
| CWSNP4581 | Ca-Kabuli-Chr5 | 37067293 | [A/G] | Ca01509 | Intron | Aminoacyl-tRNA synthetase,classII(G/H/P/S),conserved domain |
| CWSNP4582 | Ca-Kabuli-Chr5 | 37083276 | [T/G] | Ca01507 | Intron | GRAM |
| CWSNP4583 | Ca-Kabuli-Chr5 | 37083275 | [G/T] | Ca01507 | Intron | GRAM |
| CWSNP4584 | Ca-Kabuli-Chr5 | 37126371 | [A/G] | - | DRR | - |
| CWSNP4585 | Ca-Kabuli-Chr5 | 37197321 | [C/A] | - | Intergenic | - |
| CWSNP4586 | Ca-Kabuli-Chr5 | 37274572 | [C/T] | - | Intergenic | - |
| CWSNP4587 | Ca-Kabuli-Chr5 | 37274583 | [C/T] | - | Intergenic | - |
| CWSNP4588 | Ca-Kabuli-Chr5 | 37274616 | [C/G] | - | Intergenic | - |
| CWSNP4589 | Ca-Kabuli-Chr5 | 37309935 | [T/G] | Ca01488 | Non-Synonymous-CDS | GDP-fucose proteinO-fucosyltransferase |
| CWSNP4590 | Ca-Kabuli-Chr5 | 37375575 | [C/G] | Ca01480 | Intron | Flavodoxin |
| CWSNP4591 | Ca-Kabuli-Chr5 | 37460980 | [A/G] | - | DRR | - |
| CWSNP4592 | Ca-Kabuli-Chr5 | 37555846 | [T/A] | Ca01465 | Intron | Domain of unknown function FMP27,domain-4 |

| SNP IDs | Chromosomes | Physical positions (bp) | SNPs | Gene accession IDs | Sequence components of genes | Putative functions |
|---|---|---|---|---|---|---|
| CWSNP4593 | Ca-Kabuli-Chr5 | 37578999 | [T/G] | Ca01463 | Intron | Protein of unknown function DUF914,eukaryotic |
| CWSNP4594 | Ca-Kabuli-Chr5 | 37604030 | [T/C] | - | URR | - |
| CWSNP4595 | Ca-Kabuli-Chr5 | 37874725 | [A/C] | - | Intergenic | - |
| CWSNP4596 | Ca-Kabuli-Chr5 | 37886417 | [T/G] | - | Intergenic | - |
| CWSNP4597 | Ca-Kabuli-Chr5 | 37899098 | [T/C] | - | URR | - |
| CWSNP4598 | Ca-Kabuli-Chr5 | 38018552 | [A/T] | - | Intergenic | - |
| CWSNP4599 | Ca-Kabuli-Chr5 | 38018558 | [G/C] | - | Intergenic | - |
| CWSNP4600 | Ca-Kabuli-Chr5 | 38057582 | [C/T] | Ca01417 | Non-Synonymous-CDS | Zinc finger,DHHC-type,palmitoyltransferase |
| CWSNP4601 | Ca-Kabuli-Chr5 | 38080226 | [A/G] | Ca01411 | Synonymous-CDS | Protein kinase, catalytic domain |
| CWSNP4602 | Ca-Kabuli-Chr5 | 38080349 | [C/T] | Ca01411 | Synonymous-CDS | Protein kinase, catalytic domain |
| CWSNP4603 | Ca-Kabuli-Chr5 | 38092606 | [G/A] | - | Intergenic | - |
| CWSNP4604 | Ca-Kabuli-Chr5 | 38092732 | [C/T] | - | Intergenic | - |
| CWSNP4605 | Ca-Kabuli-Chr5 | 38092735 | [C/T] | - | Intergenic | - |
| CWSNP4606 | Ca-Kabuli-Chr5 | 38092793 | [C/T] | - | Intergenic | - |
| CWSNP4607 | Ca-Kabuli-Chr5 | 38102941 | [T/G] | Ca01408 | Synonymous-CDS | - |
| CWSNP4608 | Ca-Kabuli-Chr5 | 38108396 | [G/A] | Ca01407 | Non-Synonymous-CDS | - |

| SNP IDs | Chromosomes | Physical positions (bp) | SNPs | Gene accession IDs | Sequence components of genes | Putative functions |
|---|---|---|---|---|---|---|
| CWSNP4609 | Ca-Kabuli-Chr5 | 38110219 | [T/G] | - | DRR | - |
| CWSNP4610 | Ca-Kabuli-Chr5 | 38220843 | [G/A] | - | Intergenic | - |
| CWSNP4611 | Ca-Kabuli-Chr5 | 38307036 | [G/A] | Ca01392 | Synonymous-CDS | Glycosidehydrolase,family38,core |
| CWSNP4612 | Ca-Kabuli-Chr5 | 38347201 | [A/C] | Ca01389 | Synonymous-CDS | WD40 repeat |
| CWSNP4613 | Ca-Kabuli-Chr5 | 38455563 | [A/C] | Ca07405 | Synonymous-CDS | Telomere length regulation protein, conserved domain |
| CWSNP4614 | Ca-Kabuli-Chr5 | 38509657 | [A/C] | Ca07410 | Intron | Transcriptional factor B3 |
| CWSNP4615 | Ca-Kabuli-Chr5 | 38538904 | [A/G] | Ca07411 | Intron | Glycosyltransferase,family48 |
| CWSNP4616 | Ca-Kabuli-Chr5 | 38551438 | [C/T] | - | Intergenic | - |
| CWSNP4617 | Ca-Kabuli-Chr5 | 38783604 | [C/A] | Ca07428 | Non-Synonymous-CDS | Mediator complex subunit Med28 |
| CWSNP4618 | Ca-Kabuli-Chr5 | 38857904 | [T/C] | - | Intergenic | - |
| CWSNP4619 | Ca-Kabuli-Chr5 | 38892604 | [A/C] | - | Intergenic | - |
| CWSNP4620 | Ca-Kabuli-Chr5 | 38898971 | [G/A] | - | DRR | - |
| CWSNP4621 | Ca-Kabuli-Chr5 | 38909701 | [C/T] | Ca07443 | Non-Synonymous-CDS | Drought induced19/RING finger protein 114 |
| CWSNP4622 | Ca-Kabuli-Chr5 | 39283856 | [G/A] | Ca07474 | Intron | - |
| CWSNP4623 | Ca-Kabuli-Chr5 | 39326933 | [G/T] | - | Intergenic | - |
| CWSNP4624 | Ca-Kabuli-Chr5 | 39364522 | [T/C] | - | Intergenic | - |

| SNP IDs | Chromosomes | Physical positions (bp) | SNPs | Gene accession IDs | Sequence components of genes | Putative functions |
|---|---|---|---|---|---|---|
| CWSNP4625 | Ca-Kabuli-Chr5 | 39364569 | [G/A] | - | Intergenic | - |
| CWSNP4626 | Ca-Kabuli-Chr5 | 39364571 | [C/A] | - | Intergenic | - |
| CWSNP4627 | Ca-Kabuli-Chr5 | 39364720 | [A/T] | - | Intergenic | - |
| CWSNP4628 | Ca-Kabuli-Chr5 | 39364700 | [C/A] | - | Intergenic | - |
| CWSNP4629 | Ca-Kabuli-Chr5 | 39364695 | [A/C] | - | Intergenic | - |
| CWSNP4630 | Ca-Kabuli-Chr5 | 39364667 | [T/A] | - | Intergenic | - |
| CWSNP4631 | Ca-Kabuli-Chr5 | 39364741 | [A/T] | - | Intergenic | - |
| CWSNP4632 | Ca-Kabuli-Chr5 | 39364752 | [G/T] | - | Intergenic | - |
| CWSNP4633 | Ca-Kabuli-Chr5 | 39364895 | [A/C] | - | Intergenic | - |
| CWSNP4634 | Ca-Kabuli-Chr5 | 39364856 | [A/G] | - | Intergenic | - |
| CWSNP4635 | Ca-Kabuli-Chr5 | 39364848 | [C/T] | - | Intergenic | - |
| CWSNP4636 | Ca-Kabuli-Chr5 | 39364841 | [A/T] | - | Intergenic | - |
| CWSNP4637 | Ca-Kabuli-Chr5 | 39366353 | [C/G] | - | Intergenic | - |
| CWSNP4638 | Ca-Kabuli-Chr5 | 39385603 | [T/G] | - | Intergenic | - |
| CWSNP4639 | Ca-Kabuli-Chr5 | 39385630 | [C/T] | - | Intergenic | - |
| CWSNP4640 | Ca-Kabuli-Chr5 | 39410329 | [T/G] | Ca07478 | Intron | Glycosyltransferase,family3 |

| SNP IDs | Chromosomes | Physical positions (bp) | SNPs | Gene accession IDs | Sequence components of genes | Putative functions |
|---|---|---|---|---|---|---|
| CWSNP4641 | Ca-Kabuli-Chr5 | 39543929 | [C/T] | Ca07489 | Non-Synonymous-CDS | Phosphatidyl inositol 3-/4-kinase, catalytic |
| CWSNP4642 | Ca-Kabuli-Chr5 | 39576823 | [C/T] | Ca07489 | Intron | Phosphatidyl inositol 3-/4-kinase, catalytic |
| CWSNP4643 | Ca-Kabuli-Chr5 | 39576836 | [T/A] | Ca07489 | Intron | Phosphatidyl inositol 3-/4-kinase, catalytic |
| CWSNP4644 | Ca-Kabuli-Chr5 | 39576839 | [C/T] | Ca07489 | Intron | Phosphatidyl inositol 3-/4-kinase, catalytic |
| CWSNP4645 | Ca-Kabuli-Chr5 | 39576864 | [C/A] | Ca07489 | Intron | Phosphatidyl inositol 3-/4-kinase, catalytic |
| CWSNP4646 | Ca-Kabuli-Chr5 | 39576870 | [C/T] | Ca07489 | Intron | Phosphatidyl inositol 3-/4-kinase, catalytic |
| CWSNP4647 | Ca-Kabuli-Chr5 | 39576874 | [G/T] | Ca07489 | Intron | Phosphatidyl inositol 3-/4-kinase, catalytic |
| CWSNP4648 | Ca-Kabuli-Chr5 | 39576841 | [G/A] | Ca07489 | Intron | Phosphatidyl inositol 3-/4-kinase, catalytic |
| CWSNP4649 | Ca-Kabuli-Chr5 | 39597485 | [C/T] | Ca07489 | Intron | Phosphatidyl inositol 3-/4-kinase, catalytic |
| CWSNP4650 | Ca-Kabuli-Chr5 | 39608204 | [A/G] | Ca07489 | Synonymous-CDS | Phosphatidyl inositol 3-/4-kinase, catalytic |
| CWSNP4651 | Ca-Kabuli-Chr5 | 39608209 | [G/T] | Ca07489 | Intron | Phosphatidyl inositol 3-/4-kinase, catalytic |
| CWSNP4652 | Ca-Kabuli-Chr5 | 39776818 | [C/T] | - | Intergenic | - |
| CWSNP4653 | Ca-Kabuli-Chr5 | 39869398 | [C/T] | Ca07512 | Synonymous-CDS | Xanthine/uracil/vitaminCpermease |
| CWSNP4654 | Ca-Kabuli-Chr5 | 39943298 | [C/T] | - | Intergenic | - |
| CWSNP4655 | Ca-Kabuli-Chr5 | 39985332 | [A/G] | - | URR | - |
| CWSNP4656 | Ca-Kabuli-Chr5 | 40018264 | [A/C] | - | URR | - |

| SNP IDs | Chromosomes | Physical positions (bp) | SNPs | Gene accession IDs | Sequence components of genes | Putative functions |
|---|---|---|---|---|---|---|
| CWSNP4657 | Ca-Kabuli-Chr5 | 40025376 | [A/G] | Ca07531 | Intron | Argonaute/Dicerprotein,PAZ |
| CWSNP4658 | Ca-Kabuli-Chr5 | 40025377 | [T/A] | Ca07531 | Intron | Argonaute/Dicerprotein,PAZ |
| CWSNP4659 | Ca-Kabuli-Chr5 | 40162755 | [T/C] | - | Intergenic | - |
| CWSNP4660 | Ca-Kabuli-Chr5 | 40164176 | [C/G] | - | Intergenic | - |
| CWSNP4661 | Ca-Kabuli-Chr5 | 40464608 | [A/G] | - | DRR | - |
| CWSNP4662 | Ca-Kabuli-Chr5 | 40507018 | [T/A] | Ca07571 | Intron | Zinc finger,CCHC-type |
| CWSNP4663 | Ca-Kabuli-Chr5 | 40532435 | [A/G] | - | Intergenic | - |
| CWSNP4664 | Ca-Kabuli-Chr5 | 40666174 | [C/T] | Ca07586 | Intron | Sugar/inositol transporter |
| CWSNP4665 | Ca-Kabuli-Chr5 | 40700318 | [C/A] | Ca07589 | Non-Synonymous-CDS | Glycosidehydrolase,family17 |
| CWSNP4666 | Ca-Kabuli-Chr5 | 40736291 | [C/G] | Ca07594 | Intron | Oligopeptide transporterOPTsuperfamily |
| CWSNP4667 | Ca-Kabuli-Chr5 | 40828566 | [A/C] | - | URR | - |
| CWSNP4668 | Ca-Kabuli-Chr5 | 40828567 | [G/C] | - | URR | - |
| CWSNP4669 | Ca-Kabuli-Chr5 | 40841690 | [C/T] | Ca07604 | Non-Synonymous-CDS | Oligopeptide transporterOPTsuperfamily |
| CWSNP4670 | Ca-Kabuli-Chr5 | 40883438 | [C/A] | - | Intergenic | - |
| CWSNP4671 | Ca-Kabuli-Chr5 | 41063657 | [C/T] | - | Intergenic | - |
| CWSNP4672 | Ca-Kabuli-Chr5 | 41066644 | [C/G] | - | Intergenic | - |

| SNP IDs | Chromosomes | Physical positions (bp) | SNPs | Gene accession IDs | Sequence components of genes | Putative functions |
|---|---|---|---|---|---|---|
| CWSNP4673 | Ca-Kabuli-Chr5 | 41066664 | [T/C] | - | Intergenic | - |
| CWSNP4674 | Ca-Kabuli-Chr5 | 41066667 | [C/A] | - | Intergenic | - |
| CWSNP4675 | Ca-Kabuli-Chr5 | 41197909 | [A/T] | - | Intergenic | - |
| CWSNP4676 | Ca-Kabuli-Chr5 | 41283253 | [T/C] | Ca07642 | Non-Synonymous-CDS | Protein of unknown function DUF1296 |
| CWSNP4677 | Ca-Kabuli-Chr5 | 41283453 | [A/C] | Ca07642 | Non-Synonymous-CDS | Protein of unknown function DUF1296 |
| CWSNP4678 | Ca-Kabuli-Chr5 | 41407253 | [T/C] | - | URR | - |
| CWSNP4679 | Ca-Kabuli-Chr5 | 41678144 | [C/T] | Ca19215 | Synonymous-CDS | Argonaute/Dicerprotein,PAZ |
| CWSNP4680 | Ca-Kabuli-Chr5 | 41945308 | [G/A] | Ca11418 | Non-Synonymous-CDS | Helicase,C-terminal |
| CWSNP4681 | Ca-Kabuli-Chr5 | 42004600 | [T/G] | Ca11412 | Intron | Rab-GAP/TBCdomain |
| CWSNP4682 | Ca-Kabuli-Chr5 | 42004602 | [A/G] | Ca11412 | Intron | Rab-GAP/TBCdomain |
| CWSNP4683 | Ca-Kabuli-Chr5 | 42225634 | [G/A] | - | Intergenic | - |
| CWSNP4684 | Ca-Kabuli-Chr5 | 42413430 | [A/C] | Ca11377 | Intron | Zinc finger,Sec23/Sec24-type |
| CWSNP4685 | Ca-Kabuli-Chr5 | 42436972 | [A/G] | - | Intergenic | - |
| CWSNP4686 | Ca-Kabuli-Chr5 | 42436976 | [A/T] | - | Intergenic | - |
| CWSNP4687 | Ca-Kabuli-Chr5 | 42528744 | [T/G] | Ca11361 | Non-Synonymous-CDS | IQ motif, EF-hand binding site |
| CWSNP4688 | Ca-Kabuli-Chr5 | 42528934 | [A/G] | Ca11361 | Non-Synonymous-CDS | IQ motif, EF-hand binding site |

| SNP IDs | Chromosomes | Physical positions (bp) | SNPs | Gene accession IDs | Sequence components of genes | Putative functions |
|---|---|---|---|---|---|---|
| CWSNP4689 | Ca-Kabuli-Chr5 | 42749499 | [C/T] | Ca11334 | Intron | - |
| CWSNP4690 | Ca-Kabuli-Chr5 | 42863366 | [C/T] | - | DRR | - |
| CWSNP4691 | Ca-Kabuli-Chr5 | 42883163 | [T/C] | - | URR | - |
| CWSNP4692 | Ca-Kabuli-Chr5 | 42910653 | [A/G] | - | DRR | - |
| CWSNP4693 | Ca-Kabuli-Chr5 | 42928986 | [T/G] | Ca11315 | Synonymous-CDS | SANT domain, DNA binding |
| CWSNP4694 | Ca-Kabuli-Chr5 | 42929087 | [A/C] | - | Intergenic | - |
| CWSNP4695 | Ca-Kabuli-Chr5 | 43037863 | [G/A] | Ca11305 | Non-Synonymous-CDS | Regulator of G protein signalling |
| CWSNP4696 | Ca-Kabuli-Chr5 | 43067905 | [A/C] | Ca11300 | Synonymous-CDS | Membrane insertion protein, OxaA/YidC |
| CWSNP4697 | Ca-Kabuli-Chr5 | 43080493 | [G/A] | Ca11299 | Non-Synonymous-CDS | Regulator of chromosome condensation, RCC1 |
| CWSNP4698 | Ca-Kabuli-Chr5 | 43231220 | [T/C] | Ca12734 | Synonymous-CDS | Protein kinase, catalytic domain |
| CWSNP4699 | Ca-Kabuli-Chr5 | 43275472 | [T/C] | - | DRR | - |
| CWSNP4700 | Ca-Kabuli-Chr5 | 43710359 | [T/G] | - | Intergenic | - |
| CWSNP4701 | Ca-Kabuli-Chr5 | 43710362 | [A/T] | - | Intergenic | - |
| CWSNP4702 | Ca-Kabuli-Chr5 | 43710366 | [G/T] | - | Intergenic | - |
| CWSNP4703 | Ca-Kabuli-Chr5 | 43710391 | [A/G] | - | Intergenic | - |
| CWSNP4704 | Ca-Kabuli-Chr5 | 43742448 | [C/T] | - | Intergenic | - |

| SNP IDs | Chromosomes | Physical positions (bp) | SNPs | Gene accession IDs | Sequence components of genes | Putative functions |
|---|---|---|---|---|---|---|
| CWSNP4705 | Ca-Kabuli-Chr5 | 43742456 | [G/A] | - | Intergenic | - |
| CWSNP4706 | Ca-Kabuli-Chr5 | 43759059 | [A/C] | Ca12676 | Non-Synonymous-CDS | C2 calcium-dependent membrane targeting |
| CWSNP4707 | Ca-Kabuli-Chr5 | 43904489 | [T/A] | - | Intergenic | - |
| CWSNP4708 | Ca-Kabuli-Chr5 | 43919009 | [C/T] | - | Intergenic | - |
| CWSNP4709 | Ca-Kabuli-Chr5 | 43919017 | [T/G] | - | Intergenic | - |
| CWSNP4710 | Ca-Kabuli-Chr5 | 43919022 | [G/C] | - | Intergenic | - |
| CWSNP4711 | Ca-Kabuli-Chr5 | 43919028 | [A/G] | - | Intergenic | - |
| CWSNP4712 | Ca-Kabuli-Chr5 | 44109260 | [G/T] | Ca12646 | Non-Synonymous-CDS | - |
| CWSNP4713 | Ca-Kabuli-Chr5 | 44159237 | [C/G] | - | URR | - |
| CWSNP4714 | Ca-Kabuli-Chr5 | 44373968 | [G/T] | Ca03885 | Non-Synonymous-CDS | - |
| CWSNP4715 | Ca-Kabuli-Chr5 | 44378178 | [C/A] | Ca03885 | Intron | - |
| CWSNP4716 | Ca-Kabuli-Chr5 | 44555718 | [A/C] | - | Intergenic | - |
| CWSNP4717 | Ca-Kabuli-Chr5 | 44656921 | [G/A] | - | DRR | - |
| CWSNP4718 | Ca-Kabuli-Chr5 | 44667297 | [C/T] | - | URR | - |
| CWSNP4719 | Ca-Kabuli-Chr5 | 44760403 | [A/G] | Ca03920 | Synonymous-CDS | Transcription factor GRAS |
| CWSNP4720 | Ca-Kabuli-Chr5 | 44760347 | [G/T] | Ca03920 | Non-Synonymous-CDS | Transcription factor GRAS |

| SNP IDs | Chromosomes | Physical positions (bp) | SNPs | Gene accession IDs | Sequence components of genes | Putative functions |
|---|---|---|---|---|---|---|
| CWSNP4721 | Ca-Kabuli-Chr5 | 44760469 | [C/T] | Ca03920 | Synonymous-CDS | Transcription factor GRAS |
| CWSNP4722 | Ca-Kabuli-Chr5 | 44772326 | [G/A] | - | DRR | - |
| CWSNP4723 | Ca-Kabuli-Chr5 | 44887105 | [T/G] | Ca03932 | Intron | Sec1-like protein |
| CWSNP4724 | Ca-Kabuli-Chr5 | 44887241 | [T/C] | Ca03932 | Intron | Sec1-like protein |
| CWSNP4725 | Ca-Kabuli-Chr5 | 44887234 | [A/C] | Ca03932 | Intron | Sec1-like protein |
| CWSNP4726 | Ca-Kabuli-Chr5 | 44891426 | [G/C] | - | Intergenic | - |
| CWSNP4727 | Ca-Kabuli-Chr5 | 44911842 | [C/T] | - | Intergenic | - |
| CWSNP4728 | Ca-Kabuli-Chr5 | 44918947 | [C/A] | - | Intergenic | - |
| CWSNP4729 | Ca-Kabuli-Chr5 | 44938814 | [A/T] | - | DRR | - |
| CWSNP4730 | Ca-Kabuli-Chr5 | 44939058 | [C/G] | - | DRR | - |
| CWSNP4731 | Ca-Kabuli-Chr5 | 44939033 | [G/A] | - | DRR | - |
| CWSNP4732 | Ca-Kabuli-Chr5 | 45087170 | [G/A] | - | Intergenic | - |
| CWSNP4733 | Ca-Kabuli-Chr5 | 45087156 | [A/G] | - | Intergenic | - |
| CWSNP4734 | Ca-Kabuli-Chr5 | 45087146 | [C/G] | - | Intergenic | - |
| CWSNP4735 | Ca-Kabuli-Chr5 | 45087140 | [C/A] | - | Intergenic | - |
| CWSNP4736 | Ca-Kabuli-Chr5 | 45087202 | [G/C] | - | Intergenic | - |

| SNP IDs | Chromosomes | Physical positions (bp) | SNPs | Gene accession IDs | Sequence components of genes | Putative functions |
|---|---|---|---|---|---|---|
| CWSNP4737 | Ca-Kabuli-Chr5 | 45254112 | [T/G] | Ca03969 | Non-Synonymous-CDS | Zinc finger,C2H2-typematrin |
| CWSNP4738 | Ca-Kabuli-Chr5 | 45337307 | [C/T] | Ca03976 | Synonymous-CDS | Pectinesterase,catalytic |
| CWSNP4739 | Ca-Kabuli-Chr5 | 45407044 | [A/C] | Ca03983 | Intron | - |
| CWSNP4740 | Ca-Kabuli-Chr5 | 45480175 | [T/G] | - | Intergenic | - |
| CWSNP4741 | Ca-Kabuli-Chr5 | 45501379 | [C/T] | - | Intergenic | - |
| CWSNP4742 | Ca-Kabuli-Chr5 | 45694453 | [T/G] | Ca04017 | Synonymous-CDS | ATPase,AAA+type,core |
| CWSNP4743 | Ca-Kabuli-Chr5 | 45710509 | [G/A] | - | Intergenic | - |
| CWSNP4744 | Ca-Kabuli-Chr5 | 45710476 | [A/G] | - | Intergenic | - |
| CWSNP4745 | Ca-Kabuli-Chr5 | 45750121 | [A/T] | Ca04024 | Intron | Glucosamine/galactosamine-6-phosphateisomerase |
| CWSNP4746 | Ca-Kabuli-Chr5 | 45842438 | [A/C] | - | URR | - |
| CWSNP4747 | Ca-Kabuli-Chr5 | 45845081 | [A/C] | - | Intergenic | - |
| CWSNP4748 | Ca-Kabuli-Chr5 | 46018939 | [C/T] | - | DRR | - |
| CWSNP4749 | Ca-Kabuli-Chr5 | 46022566 | [C/T] | - | URR | - |
| CWSNP4750 | Ca-Kabuli-Chr5 | 46022598 | [T/C] | - | URR | - |
| CWSNP4751 | Ca-Kabuli-Chr5 | 46077302 | [G/A] | Ca04056 | Non-Synonymous-CDS | Signal transduction response regulator,receiver domain |
| CWSNP4752 | Ca-Kabuli-Chr5 | 46130289 | [A/C] | - | DRR | - |

| SNP IDs | Chromosomes | Physical positions (bp) | SNPs | Gene accession IDs | Sequence components of genes | Putative functions |
|---|---|---|---|---|---|---|
| CWSNP4753 | Ca-Kabuli-Chr5 | 46176443 | [G/A] | - | URR | - |
| CWSNP4754 | Ca-Kabuli-Chr5 | 46252643 | [T/A] | - | Intergenic | - |
| CWSNP4755 | Ca-Kabuli-Chr5 | 46252605 | [C/A] | - | Intergenic | - |
| CWSNP4756 | Ca-Kabuli-Chr5 | 46301639 | [A/G] | Ca04083 | Synonymous-CDS | - |
| CWSNP4757 | Ca-Kabuli-Chr5 | 46328627 | [A/G] | - | Intergenic | - |
| CWSNP4758 | Ca-Kabuli-Chr5 | 46328895 | [T/C] | - | Intergenic | - |
| CWSNP4759 | Ca-Kabuli-Chr5 | 46422869 | [A/T] | - | Intergenic | - |
| CWSNP4760 | Ca-Kabuli-Chr5 | 46526963 | [A/C] | Ca04110 | Synonymous-CDS | Protein of unknown function DUF2044,membrane |
| CWSNP4761 | Ca-Kabuli-Chr5 | 47073011 | [A/T] | - | URR | - |
| CWSNP4762 | Ca-Kabuli-Chr5 | 47298345 | [A/G] | Ca04186 | Intron | Phosphatidyl inositol 3-/4-kinase, catalytic |
| CWSNP4763 | Ca-Kabuli-Chr5 | 47329979 | [T/G] | Ca04190 | Non-Synonymous-CDS | Glycosyltransferase,family48 |
| CWSNP4764 | Ca-Kabuli-Chr5 | 47362666 | [T/G] | - | Intergenic | - |
| CWSNP4765 | Ca-Kabuli-Chr5 | 47641628 | [T/C] | Ca04224 | Synonymous-CDS | Heat shock protein DnaJ,N-terminal |
| CWSNP4766 | Ca-Kabuli-Chr5 | 47641567 | [A/G] | Ca04224 | Non-Synonymous-CDS | Heat shock protein DnaJ,N-terminal |
| CWSNP4767 | Ca-Kabuli-Chr5 | 47643992 | [G/T] | Ca04224 | Intron | Heat shock protein DnaJ,N-terminal |
| CWSNP4768 | Ca-Kabuli-Chr5 | 47670649 | [G/A] | Ca04227 | Non-Synonymous-CDS | Amino acid transporter,transmembrane |

| SNP IDs | Chromosomes | Physical positions (bp) | SNPs | Gene accession IDs | Sequence components of genes | Putative functions |
|---|---|---|---|---|---|---|
| CWSNP4769 | Ca-Kabuli-Chr5 | 47718830 | [A/G] | Ca04232 | Intron | Glycosidehydrolase,family35 |
| CWSNP4770 | Ca-Kabuli-Chr5 | 47770909 | [T/G] | Ca04238 | Intron | Amidase |
| CWSNP4771 | Ca-Kabuli-Chr5 | 47812041 | [C/T] | - | Intergenic | - |
| CWSNP4772 | Ca-Kabuli-Chr5 | 47829331 | [T/A] | - | Intergenic | - |
| CWSNP4773 | Ca-Kabuli-Chr5 | 47965279 | [G/A] | Ca04258 | Non-Synonymous-CDS | - |
| CWSNP4774 | Ca-Kabuli-Chr5 | 47994780 | [A/C] | Ca04259 | Intron | Cofprotein |
| CWSNP4775 | Ca-Kabuli-Chr5 | 48069833 | [G/A] | - | Intergenic | - |
| CWSNP4776 | Ca-Kabuli-Chr6 | 152513 | [C/T] | - | Intergenic | - |
| CWSNP4777 | Ca-Kabuli-Chr6 | 166823 | [G/A] | - | URR | - |
| CWSNP4778 | Ca-Kabuli-Chr6 | 166806 | [C/T] | - | URR | - |
| CWSNP4779 | Ca-Kabuli-Chr6 | 166959 | [G/A] | - | URR | - |
| CWSNP4780 | Ca-Kabuli-Chr6 | 166943 | [G/A] | - | URR | - |
| CWSNP4781 | Ca-Kabuli-Chr6 | 166938 | [G/T] | - | URR | - |
| CWSNP4782 | Ca-Kabuli-Chr6 | 166934 | [G/A] | - | URR | - |
| CWSNP4783 | Ca-Kabuli-Chr6 | 166932 | [G/A] | - | URR | - |
| CWSNP4784 | Ca-Kabuli-Chr6 | 204368 | [G/A] | Ca13250 | Intron | Flavin-containing amineoxidase |

| SNP IDs | Chromosomes | Physical positions (bp) | SNPs | Gene accession IDs | Sequence components of genes | Putative functions |
|---|---|---|---|---|---|---|
| CWSNP4785 | Ca-Kabuli-Chr6 | 204412 | [G/C] | Ca13250 | Intron | Flavin-containing amineoxidase |
| CWSNP4786 | Ca-Kabuli-Chr6 | 204438 | [T/C] | Ca13250 | Intron | Flavin-containing amineoxidase |
| CWSNP4787 | Ca-Kabuli-Chr6 | 324301 | [C/T] | Ca13257 | Non-Synonymous-CDS | Zinc finger,FYVE-type |
| CWSNP4788 | Ca-Kabuli-Chr6 | 375684 | [T/G] | Ca13262 | Synonymous-CDS | Pyridoxalphosphate-dependent decarboxylase |
| CWSNP4789 | Ca-Kabuli-Chr6 | 386250 | [A/G] | - | Intergenic | - |
| CWSNP4790 | Ca-Kabuli-Chr6 | 474489 | [C/A] | - | DRR | - |
| CWSNP4791 | Ca-Kabuli-Chr6 | 505875 | [T/C] | Ca13271 | Non-Synonymous-CDS | - |
| CWSNP4792 | Ca-Kabuli-Chr6 | 551130 | [T/G] | Ca13276 | Non-Synonymous-CDS | Nicastrin |
| CWSNP4793 | Ca-Kabuli-Chr6 | 580616 | [G/C] | - | Intergenic | - |
| CWSNP4794 | Ca-Kabuli-Chr6 | 647322 | [A/C] | Ca13283 | Synonymous-CDS | - |
| CWSNP4795 | Ca-Kabuli-Chr6 | 697168 | [C/T] | - | Intergenic | - |
| CWSNP4796 | Ca-Kabuli-Chr6 | 697147 | [A/G] | - | Intergenic | - |
| CWSNP4797 | Ca-Kabuli-Chr6 | 961805 | [C/T] | - | DRR | - |
| CWSNP4798 | Ca-Kabuli-Chr6 | 997997 | [T/A] | - | DRR | - |
| CWSNP4799 | Ca-Kabuli-Chr6 | 1220460 | [G/C] | - | Intergenic | - |
| CWSNP4800 | Ca-Kabuli-Chr6 | 1290413 | [C/G] | Ca13334 | Intron | Regulator of chromosome condensation, RCC1 |

| SNP IDs | Chromosomes | Physical positions (bp) | SNPs | Gene accession IDs | Sequence components of genes | Putative functions |
|---|---|---|---|---|---|---|
| CWSNP4801 | Ca-Kabuli-Chr6 | 1315583 | [G/C] | Ca13338 | Intron | Aspartate/glutamate/uridylatekinase |
| CWSNP4802 | Ca-Kabuli-Chr6 | 1626723 | [T/C] | - | Intergenic | - |
| CWSNP4803 | Ca-Kabuli-Chr6 | 1626712 | [C/T] | - | Intergenic | - |
| CWSNP4804 | Ca-Kabuli-Chr6 | 1817599 | [C/T] | Ca10308 | Intron | Zinc finger,LIM-type |
| CWSNP4805 | Ca-Kabuli-Chr6 | 1817602 | [A/T] | Ca10308 | Intron | Zinc finger,LIM-type |
| CWSNP4806 | Ca-Kabuli-Chr6 | 1817607 | [T/C] | Ca10308 | Intron | Zinc finger,LIM-type |
| CWSNP4807 | Ca-Kabuli-Chr6 | 1932175 | [G/A] | Ca10319 | Intron | Major intrinsic protein |
| CWSNP4808 | Ca-Kabuli-Chr6 | 1966137 | [T/C] | - | Intergenic | - |
| CWSNP4809 | Ca-Kabuli-Chr6 | 1978864 | [A/C] | Ca10322 | Intron | - |
| CWSNP4810 | Ca-Kabuli-Chr6 | 1978867 | [T/A] | Ca10322 | Intron | - |
| CWSNP4811 | Ca-Kabuli-Chr6 | 2037620 | [T/G] | - | URR | - |
| CWSNP4812 | Ca-Kabuli-Chr6 | 2061459 | [A/G] | - | URR | - |
| CWSNP4813 | Ca-Kabuli-Chr6 | 2079137 | [T/G] | - | DRR | - |
| CWSNP4814 | Ca-Kabuli-Chr6 | 2079186 | [G/T] | - | DRR | - |
| CWSNP4815 | Ca-Kabuli-Chr6 | 2083805 | [G/A] | Ca10330 | Synonymous-CDS | K+ potassium transporter |
| CWSNP4816 | Ca-Kabuli-Chr6 | 2083813 | [T/C] | Ca10330 | Non-Synonymous-CDS | K+ potassium transporter |

| SNP IDs | Chromosomes | Physical positions (bp) | SNPs | Gene accession IDs | Sequence components of genes | Putative functions |
|---|---|---|---|---|---|---|
| CWSNP4817 | Ca-Kabuli-Chr6 | 2126667 | [C/T] | - | DRR | - |
| CWSNP4818 | Ca-Kabuli-Chr6 | 2140189 | [G/T] | Ca10336 | Non-Synonymous-CDS | Leucine-rich repeat |
| CWSNP4819 | Ca-Kabuli-Chr6 | 2140406 | [G/A] | Ca10336 | Synonymous-CDS | Leucine-rich repeat |
| CWSNP4820 | Ca-Kabuli-Chr6 | 2193748 | [T/A] | Ca10344 | Intron | Auxin efflux carrier |
| CWSNP4821 | Ca-Kabuli-Chr6 | 2193763 | [A/C] | Ca10344 | Intron | Auxin efflux carrier |
| CWSNP4822 | Ca-Kabuli-Chr6 | 2193803 | [G/A] | Ca10344 | Synonymous-CDS | Auxin efflux carrier |
| CWSNP4823 | Ca-Kabuli-Chr6 | 2214131 | [T/A] | Ca10348 | Intron | Amidase |
| CWSNP4824 | Ca-Kabuli-Chr6 | 2214178 | [T/C] | Ca10348 | Intron | Amidase |
| CWSNP4825 | Ca-Kabuli-Chr6 | 2214247 | [A/G] | Ca10348 | Intron | Amidase |
| CWSNP4826 | Ca-Kabuli-Chr6 | 2223544 | [A/T] | - | Intergenic | - |
| CWSNP4827 | Ca-Kabuli-Chr6 | 2223578 | [T/G] | - | Intergenic | - |
| CWSNP4828 | Ca-Kabuli-Chr6 | 2223664 | [A/C] | - | Intergenic | - |
| CWSNP4829 | Ca-Kabuli-Chr6 | 2244901 | [A/C] | - | Intergenic | - |
| CWSNP4830 | Ca-Kabuli-Chr6 | 2275505 | [A/G] | Ca10356 | Non-Synonymous-CDS | - |
| CWSNP4831 | Ca-Kabuli-Chr6 | 2275524 | [G/A] | Ca10356 | Synonymous-CDS | - |
| CWSNP4832 | Ca-Kabuli-Chr6 | 2275553 | [A/G] | Ca10356 | Non-Synonymous-CDS | - |

| SNP IDs | Chromosomes | Physical positions (bp) | SNPs | Gene accession IDs | Sequence components of genes | Putative functions |
|---|---|---|---|---|---|---|
| CWSNP4833 | Ca-Kabuli-Chr6 | 2280734 | [T/A] | Ca10356 | Intron | - |
| CWSNP4834 | Ca-Kabuli-Chr6 | 2280752 | [A/G] | Ca10356 | Intron | - |
| CWSNP4835 | Ca-Kabuli-Chr6 | 2286237 | [C/T] | - | Intergenic | - |
| CWSNP4836 | Ca-Kabuli-Chr6 | 2294911 | [T/G] | - | Intergenic | - |
| CWSNP4837 | Ca-Kabuli-Chr6 | 2303123 | [T/G] | Ca10360 | Intron | Protein kinase, catalytic domain |
| CWSNP4838 | Ca-Kabuli-Chr6 | 2313683 | [A/C] | Ca10361 | Non-Synonymous-CDS | HistoneH1/H5 |
| CWSNP4839 | Ca-Kabuli-Chr6 | 2313828 | [G/A] | Ca10361 | Non-Synonymous-CDS | HistoneH1/H5 |
| CWSNP4840 | Ca-Kabuli-Chr6 | 2314029 | [T/C] | Ca10361 | Non-Synonymous-CDS | HistoneH1/H5 |
| CWSNP4841 | Ca-Kabuli-Chr6 | 2462481 | [C/T] | - | DRR | - |
| CWSNP4842 | Ca-Kabuli-Chr6 | 2502485 | [A/G] | - | URR | - |
| CWSNP4843 | Ca-Kabuli-Chr6 | 2527768 | [A/G] | - | Intergenic | - |
| CWSNP4844 | Ca-Kabuli-Chr6 | 2542942 | [A/T] | - | Intergenic | - |
| CWSNP4845 | Ca-Kabuli-Chr6 | 2542981 | [T/C] | - | Intergenic | - |
| CWSNP4846 | Ca-Kabuli-Chr6 | 2543096 | [C/T] | - | Intergenic | - |
| CWSNP4847 | Ca-Kabuli-Chr6 | 2543062 | [A/G] | - | Intergenic | - |
| CWSNP4848 | Ca-Kabuli-Chr6 | 2548412 | [A/G] | Ca10387 | Synonymous-CDS | Zinc finger,RING-type |

| SNP IDs | Chromosomes | Physical positions (bp) | SNPs | Gene accession IDs | Sequence components of genes | Putative functions |
|---|---|---|---|---|---|---|
| CWSNP4849 | Ca-Kabuli-Chr6 | 2549991 | [T/C] | Ca10387 | Synonymous-CDS | Zinc finger,RING-type |
| CWSNP4850 | Ca-Kabuli-Chr6 | 2550154 | [A/C] | Ca10387 | Synonymous-CDS | Zinc finger,RING-type |
| CWSNP4851 | Ca-Kabuli-Chr6 | 2625793 | [C/G] | Ca10393 | Intron | THOcomplex,subunitTHOC2,N-terminal |
| CWSNP4852 | Ca-Kabuli-Chr6 | 2639558 | [T/A] | - | URR | - |
| CWSNP4853 | Ca-Kabuli-Chr6 | 2639606 | [A/G] | - | URR | - |
| CWSNP4854 | Ca-Kabuli-Chr6 | 2639607 | [T/C] | - | URR | - |
| CWSNP4855 | Ca-Kabuli-Chr6 | 2714897 | [C/A] | - | DRR | - |
| CWSNP4856 | Ca-Kabuli-Chr6 | 2717150 | [G/A] | - | Intergenic | - |
| CWSNP4857 | Ca-Kabuli-Chr6 | 2740765 | [A/G] | - | Intergenic | - |
| CWSNP4858 | Ca-Kabuli-Chr6 | 2740784 | [T/C] | - | Intergenic | - |
| CWSNP4859 | Ca-Kabuli-Chr6 | 2802942 | [C/A] | Ca10415 | Synonymous-CDS | - |
| CWSNP4860 | Ca-Kabuli-Chr6 | 2811345 | [A/G] | Ca10416 | Non-Synonymous-CDS | Nucleoporin,Nup155-like |
| CWSNP4861 | Ca-Kabuli-Chr6 | 2812794 | [A/C] | Ca10416 | Synonymous-CDS | Nucleoporin,Nup155-like |
| CWSNP4862 | Ca-Kabuli-Chr6 | 2812719 | [G/A] | Ca10416 | Synonymous-CDS | Nucleoporin,Nup155-like |
| CWSNP4863 | Ca-Kabuli-Chr6 | 2813244 | [G/A] | Ca10416 | Synonymous-CDS | Nucleoporin,Nup155-like |
| CWSNP4864 | Ca-Kabuli-Chr6 | 2821836 | [G/T] | Ca10417 | Intron | HEAT |

| SNP IDs | Chromosomes | Physical positions (bp) | SNPs | Gene accession IDs | Sequence components of genes | Putative functions |
|---|---|---|---|---|---|---|
| CWSNP4865 | Ca-Kabuli-Chr6 | 2855691 | [C/G] | Ca10421 | Intron | 4Fe-4Sbinding domain |
| CWSNP4866 | Ca-Kabuli-Chr6 | 2864713 | [G/A] | - | DRR | - |
| CWSNP4867 | Ca-Kabuli-Chr6 | 2919355 | [A/C] | Ca10430 | Intron | UBX |
| CWSNP4868 | Ca-Kabuli-Chr6 | 2925908 | [A/G] | - | URR | - |
| CWSNP4869 | Ca-Kabuli-Chr6 | 2926006 | [T/C] | - | URR | - |
| CWSNP4870 | Ca-Kabuli-Chr6 | 2927271 | [G/A] | - | URR | - |
| CWSNP4871 | Ca-Kabuli-Chr6 | 2927386 | [C/G] | - | Intergenic | - |
| CWSNP4872 | Ca-Kabuli-Chr6 | 2927387 | [T/C] | - | Intergenic | - |
| CWSNP4873 | Ca-Kabuli-Chr6 | 2930459 | [C/T] | - | DRR | - |
| CWSNP4874 | Ca-Kabuli-Chr6 | 2930504 | [T/C] | - | DRR | - |
| CWSNP4875 | Ca-Kabuli-Chr6 | 2932179 | [T/G] | - | Intergenic | - |
| CWSNP4876 | Ca-Kabuli-Chr6 | 2932168 | [A/C] | - | Intergenic | - |
| CWSNP4877 | Ca-Kabuli-Chr6 | 2932143 | [C/A] | - | Intergenic | - |
| CWSNP4878 | Ca-Kabuli-Chr6 | 2972319 | [C/T] | Ca10439 | Intron | Alpha-D-phosphohexomutase |
| CWSNP4879 | Ca-Kabuli-Chr6 | 2972301 | [T/C] | Ca10439 | Intron | Alpha-D-phosphohexomutase |
| CWSNP4880 | Ca-Kabuli-Chr6 | 2973756 | [A/C] | Ca10439 | Intron | Alpha-D-phosphohexomutase |

| SNP IDs | Chromosomes | Physical positions (bp) | SNPs | Gene accession IDs | Sequence components of genes | Putative functions |
|---|---|---|---|---|---|---|
| CWSNP4881 | Ca-Kabuli-Chr6 | 3060508 | [G/A] | - | Intergenic | - |
| CWSNP4882 | Ca-Kabuli-Chr6 | 3060502 | [G/A] | - | Intergenic | - |
| CWSNP4883 | Ca-Kabuli-Chr6 | 3288481 | [C/A] | - | Intergenic | - |
| CWSNP4884 | Ca-Kabuli-Chr6 | 3288444 | [G/A] | - | Intergenic | - |
| CWSNP4885 | Ca-Kabuli-Chr6 | 3288441 | [A/G] | - | Intergenic | - |
| CWSNP4886 | Ca-Kabuli-Chr6 | 3288505 | [G/T] | - | Intergenic | - |
| CWSNP4887 | Ca-Kabuli-Chr6 | 3297086 | [G/A] | Ca25243 | Intron | Peptidyl-tRNAhydrolase |
| CWSNP4888 | Ca-Kabuli-Chr6 | 3298952 | [A/T] | Ca25243 | Intron | Peptidyl-tRNAhydrolase |
| CWSNP4889 | Ca-Kabuli-Chr6 | 3395024 | [A/G] | - | DRR | - |
| CWSNP4890 | Ca-Kabuli-Chr6 | 3395253 | [G/A] | - | DRR | - |
| CWSNP4891 | Ca-Kabuli-Chr6 | 3395370 | [C/T] | - | DRR | - |
| CWSNP4892 | Ca-Kabuli-Chr6 | 3397650 | [A/G] | - | Intergenic | - |
| CWSNP4893 | Ca-Kabuli-Chr6 | 3482411 | [A/G] | - | URR | - |
| CWSNP4894 | Ca-Kabuli-Chr6 | 3505662 | [C/A] | Ca05959 | Non-Synonymous-CDS | Oligopeptide transporter |
| CWSNP4895 | Ca-Kabuli-Chr6 | 3530094 | [T/C] | Ca05957 | Intron | FAD-dependent pyridine nucleotide-disulphide oxidoreductase |
| CWSNP4896 | Ca-Kabuli-Chr6 | 3530137 | [A/G] | Ca05957 | Intron | FAD-dependent pyridine nucleotide-disulphide oxidoreductase |

| SNP IDs | Chromosomes | Physical positions (bp) | SNPs | Gene accession IDs | Sequence components of genes | Putative functions |
|---|---|---|---|---|---|---|
| CWSNP4897 | Ca-Kabuli-Chr6 | 3548121 | [C/A] | - | DRR | - |
| CWSNP4898 | Ca-Kabuli-Chr6 | 3548114 | [A/C] | - | DRR | - |
| CWSNP4899 | Ca-Kabuli-Chr6 | 3786843 | [A/G] | - | DRR | - |
| CWSNP4900 | Ca-Kabuli-Chr6 | 3787239 | [C/A] | - | DRR | - |
| CWSNP4901 | Ca-Kabuli-Chr6 | 3829070 | [C/A] | Ca05920 | Non-Synonymous-CDS | Glycosidehydrolase,family17 |
| CWSNP4902 | Ca-Kabuli-Chr6 | 3962516 | [A/C] | - | Intergenic | - |
| CWSNP4903 | Ca-Kabuli-Chr6 | 3985519 | [G/A] | - | Intergenic | - |
| CWSNP4904 | Ca-Kabuli-Chr6 | 3992419 | [C/T] | - | Intergenic | - |
| CWSNP4905 | Ca-Kabuli-Chr6 | 4112085 | [T/C] | - | Intergenic | - |
| CWSNP4906 | Ca-Kabuli-Chr6 | 4226947 | [A/T] | - | Intergenic | - |
| CWSNP4907 | Ca-Kabuli-Chr6 | 4226924 | [G/T] | - | Intergenic | - |
| CWSNP4908 | Ca-Kabuli-Chr6 | 4228884 | [T/C] | - | Intergenic | - |
| CWSNP4909 | Ca-Kabuli-Chr6 | 4344604 | [T/C] | Ca05866 | Synonymous-CDS | Srchomology-3domain |
| CWSNP4910 | Ca-Kabuli-Chr6 | 4350822 | [A/T] | Ca05866 | Non-Synonymous-CDS | Srchomology-3domain |
| CWSNP4911 | Ca-Kabuli-Chr6 | 4350867 | [A/C] | Ca05866 | Non-Synonymous-CDS | Srchomology-3domain |
| CWSNP4912 | Ca-Kabuli-Chr6 | 4350889 | [A/C] | Ca05866 | Synonymous-CDS | Srchomology-3domain |

| SNP IDs | Chromosomes | Physical positions (bp) | SNPs | Gene accession IDs | Sequence components of genes | Putative functions |
|---|---|---|---|---|---|---|
| CWSNP4913 | Ca-Kabuli-Chr6 | 4440034 | [T/G] | - | URR | - |
| CWSNP4914 | Ca-Kabuli-Chr6 | 4562263 | [C/A] | - | URR | - |
| CWSNP4915 | Ca-Kabuli-Chr6 | 4562264 | [C/G] | - | URR | - |
| CWSNP4916 | Ca-Kabuli-Chr6 | 4601303 | [A/C] | Ca05837 | Synonymous-CDS | ATPase,AAA+type,core |
| CWSNP4917 | Ca-Kabuli-Chr6 | 4678523 | [A/C] | - | Intergenic | - |
| CWSNP4918 | Ca-Kabuli-Chr6 | 4743563 | [C/G] | - | Intergenic | - |
| CWSNP4919 | Ca-Kabuli-Chr6 | 4743608 | [C/T] | - | Intergenic | - |
| CWSNP4920 | Ca-Kabuli-Chr6 | 4807908 | [G/C] | - | URR | - |
| CWSNP4921 | Ca-Kabuli-Chr6 | 4861184 | [T/G] | Ca05808 | Synonymous-CDS | - |
| CWSNP4922 | Ca-Kabuli-Chr6 | 4981078 | [G/T] | - | URR | - |
| CWSNP4923 | Ca-Kabuli-Chr6 | 5185380 | [A/G] | - | URR | - |
| CWSNP4924 | Ca-Kabuli-Chr6 | 5341510 | [C/A] | Ca05766 | Non-Synonymous-CDS | ARID/BRIGHTDNA-binding domain |
| CWSNP4925 | Ca-Kabuli-Chr6 | 5392185 | [T/C] | Ca05761 | Synonymous-CDS | Peptidase M1,alanine aminopeptidase/leukotriene A4 hydrolase |
| CWSNP4926 | Ca-Kabuli-Chr6 | 5396888 | [T/G] | Ca05761 | Intron | Peptidase M1,alanine aminopeptidase/leukotriene A4 hydrolase |
| CWSNP4927 | Ca-Kabuli-Chr6 | 5396890 | [T/G] | Ca05761 | Intron | Peptidase M1,alanine aminopeptidase/leukotriene A4 hydrolase |
| CWSNP4928 | Ca-Kabuli-Chr6 | 5396897 | [T/G] | Ca05761 | Intron | Peptidase M1,alanine aminopeptidase/leukotriene A4 hydrolase |

| SNP IDs | Chromosomes | Physical positions (bp) | SNPs | Gene accession IDs | Sequence components of genes | Putative functions |
|---|---|---|---|---|---|---|
| CWSNP4929 | Ca-Kabuli-Chr6 | 5453512 | [A/G] | Ca05756 | Non-Synonymous-CDS | Protein kinase, catalytic domain |
| CWSNP4930 | Ca-Kabuli-Chr6 | 5498898 | [C/T] | Ca05751 | Synonymous-CDS | GMPsynthase,C-terminal |
| CWSNP4931 | Ca-Kabuli-Chr6 | 5589373 | [T/C] | - | DRR | - |
| CWSNP4932 | Ca-Kabuli-Chr6 | 5628956 | [T/G] | - | DRR | - |
| CWSNP4933 | Ca-Kabuli-Chr6 | 5694397 | [G/T] | Ca05725 | Intron | Ribosomal proteinS1,RNA-binding domain |
| CWSNP4934 | Ca-Kabuli-Chr6 | 5704845 | [T/C] | - | DRR | - |
| CWSNP4935 | Ca-Kabuli-Chr6 | 5706649 | [C/T] | - | DRR | - |
| CWSNP4936 | Ca-Kabuli-Chr6 | 6110805 | [T/C] | Ca05684 | Non-Synonymous-CDS | PeptidaseS10,serine carboxypeptidase |
| CWSNP4937 | Ca-Kabuli-Chr6 | 6193105 | [G/C] | Ca05679 | Non-Synonymous-CDS | - |
| CWSNP4938 | Ca-Kabuli-Chr6 | 6415818 | [A/G] | - | Intergenic | - |
| CWSNP4939 | Ca-Kabuli-Chr6 | 6517785 | [T/C] | - | Intergenic | - |
| CWSNP4940 | Ca-Kabuli-Chr6 | 6779147 | [G/C] | Ca16938 | Non-Synonymous-CDS | Protein of unknown function DUF869,plant |
| CWSNP4941 | Ca-Kabuli-Chr6 | 6802259 | [C/T] | Ca16940 | Intron | Pyridinenucleotide-disulphideoxidoreductase,NAD-bindingregion |
| CWSNP4942 | Ca-Kabuli-Chr6 | 6815594 | [G/A] | - | Intergenic | - |
| CWSNP4943 | Ca-Kabuli-Chr6 | 6877088 | [C/A] | Ca16950 | Non-Synonymous-CDS | Heat shock protein DnaJ,N-terminal |
| CWSNP4944 | Ca-Kabuli-Chr6 | 6877163 | [T/C] | Ca16950 | Non-Synonymous-CDS | Heat shock protein DnaJ,N-terminal |

| SNP IDs | Chromosomes | Physical positions (bp) | SNPs | Gene accession IDs | Sequence components of genes | Putative functions |
|---|---|---|---|---|---|---|
| CWSNP4945 | Ca-Kabuli-Chr6 | 6877284 | [C/T] | Ca16950 | Synonymous-CDS | Heat shock protein DnaJ,N-terminal |
| CWSNP4946 | Ca-Kabuli-Chr6 | 6913622 | [A/C] | - | URR | - |
| CWSNP4947 | Ca-Kabuli-Chr6 | 6913695 | [G/A] | - | URR | - |
| CWSNP4948 | Ca-Kabuli-Chr6 | 7026481 | [G/C] | - | Intergenic | - |
| CWSNP4949 | Ca-Kabuli-Chr6 | 7349517 | [A/C] | - | Intergenic | - |
| CWSNP4950 | Ca-Kabuli-Chr6 | 7406219 | [A/G] | - | Intergenic | - |
| CWSNP4951 | Ca-Kabuli-Chr6 | 7571950 | [T/C] | Ca09627 | Non-Synonymous-CDS | - |
| CWSNP4952 | Ca-Kabuli-Chr6 | 7582726 | [G/T] | - | DRR | - |
| CWSNP4953 | Ca-Kabuli-Chr6 | 7600817 | [G/A] | Ca09624 | Intron | Domain of unknown function DUF699,exodeoxyribonuclease V alpha chain |
| CWSNP4954 | Ca-Kabuli-Chr6 | 7603195 | [G/A] | Ca09624 | Non-Synonymous-CDS | Domain of unknown function DUF699,exodeoxyribonuclease V alpha chain |
| CWSNP4955 | Ca-Kabuli-Chr6 | 7603178 | [A/C] | Ca09624 | Intron | Domain of unknown function DUF699,exodeoxyribonuclease V alpha chain |
| CWSNP4956 | Ca-Kabuli-Chr6 | 7647907 | [T/G] | Ca09618 | Synonymous-CDS | Ras GTPase |
| CWSNP4957 | Ca-Kabuli-Chr6 | 7647931 | [G/A] | Ca09618 | Synonymous-CDS | Ras GTPase |
| CWSNP4958 | Ca-Kabuli-Chr6 | 7661930 | [T/G] | - | DRR | - |
| CWSNP4959 | Ca-Kabuli-Chr6 | 7661924 | [T/C] | - | DRR | - |
| CWSNP4960 | Ca-Kabuli-Chr6 | 7667301 | [T/C] | - | URR | - |

| SNP IDs | Chromosomes | Physical positions (bp) | SNPs | Gene accession IDs | Sequence components of genes | Putative functions |
|---|---|---|---|---|---|---|
| CWSNP4961 | Ca-Kabuli-Chr6 | 7667328 | [A/T] | - | URR | - |
| CWSNP4962 | Ca-Kabuli-Chr6 | 7694609 | [A/T] | - | Intergenic | - |
| CWSNP4963 | Ca-Kabuli-Chr6 | 7694696 | [T/C] | - | Intergenic | - |
| CWSNP4964 | Ca-Kabuli-Chr6 | 7744730 | [A/G] | Ca09604 | Intron | ABC-1 |
| CWSNP4965 | Ca-Kabuli-Chr6 | 7745641 | [A/G] | Ca09604 | Intron | ABC-1 |
| CWSNP4966 | Ca-Kabuli-Chr6 | 7747254 | [G/A] | Ca09604 | Synonymous-CDS | ABC-1 |
| CWSNP4967 | Ca-Kabuli-Chr6 | 7770357 | [C/T] | - | DRR | - |
| CWSNP4968 | Ca-Kabuli-Chr6 | 7822666 | [C/A] | - | URR | - |
| CWSNP4969 | Ca-Kabuli-Chr6 | 7829485 | [G/A] | - | DRR | - |
| CWSNP4970 | Ca-Kabuli-Chr6 | 7829627 | [T/G] | - | DRR | - |
| CWSNP4971 | Ca-Kabuli-Chr6 | 7831214 | [C/T] | Ca09595 | Synonymous-CDS | C2 calcium-dependent membrane targeting |
| CWSNP4972 | Ca-Kabuli-Chr6 | 7831409 | [C/T] | Ca09595 | Synonymous-CDS | C2 calcium-dependent membrane targeting |
| CWSNP4973 | Ca-Kabuli-Chr6 | 7831874 | [A/G] | Ca09595 | Synonymous-CDS | C2 calcium-dependent membrane targeting |
| CWSNP4974 | Ca-Kabuli-Chr6 | 7831899 | [G/A] | Ca09595 | Non-Synonymous-CDS | C2 calcium-dependent membrane targeting |
| CWSNP4975 | Ca-Kabuli-Chr6 | 7832029 | [C/T] | Ca09595 | Non-Synonymous-CDS | C2 calcium-dependent membrane targeting |
| CWSNP4976 | Ca-Kabuli-Chr6 | 7832023 | [C/T] | Ca09595 | Non-Synonymous-CDS | C2 calcium-dependent membrane targeting |

| SNP IDs | Chromosomes | Physical positions (bp) | SNPs | Gene accession IDs | Sequence components of genes | Putative functions |
|---|---|---|---|---|---|---|
| CWSNP4977 | Ca-Kabuli-Chr6 | 7834595 | [C/T] | Ca09595 | Synonymous-CDS | C2 calcium-dependent membrane targeting |
| CWSNP4978 | Ca-Kabuli-Chr6 | 7834922 | [G/A] | Ca09595 | Synonymous-CDS | C2 calcium-dependent membrane targeting |
| CWSNP4979 | Ca-Kabuli-Chr6 | 7834895 | [G/T] | Ca09595 | Synonymous-CDS | C2 calcium-dependent membrane targeting |
| CWSNP4980 | Ca-Kabuli-Chr6 | 7834976 | [G/A] | Ca09595 | Synonymous-CDS | C2 calcium-dependent membrane targeting |
| CWSNP4981 | Ca-Kabuli-Chr6 | 7835427 | [G/A] | Ca09595 | Non-Synonymous-CDS | C2 calcium-dependent membrane targeting |
| CWSNP4982 | Ca-Kabuli-Chr6 | 7835738 | [C/T] | Ca09595 | Synonymous-CDS | C2 calcium-dependent membrane targeting |
| CWSNP4983 | Ca-Kabuli-Chr6 | 7835984 | [T/A] | Ca09595 | Non-Synonymous-CDS | C2 calcium-dependent membrane targeting |
| CWSNP4984 | Ca-Kabuli-Chr6 | 7851000 | [G/C] | - | Intergenic | - |
| CWSNP4985 | Ca-Kabuli-Chr6 | 7929263 | [C/T] | - | DRR | - |
| CWSNP4986 | Ca-Kabuli-Chr6 | 7929338 | [T/C] | - | DRR | - |
| CWSNP4987 | Ca-Kabuli-Chr6 | 7929339 | [G/A] | - | DRR | - |
| CWSNP4988 | Ca-Kabuli-Chr6 | 7929348 | [C/T] | - | DRR | - |
| CWSNP4989 | Ca-Kabuli-Chr6 | 7929384 | [T/C] | - | DRR | - |
| CWSNP4990 | Ca-Kabuli-Chr6 | 7929628 | [C/T] | - | DRR | - |
| CWSNP4991 | Ca-Kabuli-Chr6 | 7929607 | [A/C] | - | DRR | - |
| CWSNP4992 | Ca-Kabuli-Chr6 | 7939281 | [T/G] | Ca09580 | Non-Synonymous-CDS | - |

| SNP IDs | Chromosomes | Physical positions (bp) | SNPs | Gene accession IDs | Sequence components of genes | Putative functions |
|---|---|---|---|---|---|---|
| CWSNP4993 | Ca-Kabuli-Chr6 | 7939465 | [A/G] | Ca09580 | Intron | - |
| CWSNP4994 | Ca-Kabuli-Chr6 | 7994732 | [G/T] | - | URR | - |
| CWSNP4995 | Ca-Kabuli-Chr6 | 8011685 | [G/T] | - | Intergenic | - |
| CWSNP4996 | Ca-Kabuli-Chr6 | 8029226 | [C/T] | Ca09571 | Synonymous-CDS | - |
| CWSNP4997 | Ca-Kabuli-Chr6 | 8052987 | [G/C] | Ca09568 | Intron | TLDc |
| CWSNP4998 | Ca-Kabuli-Chr6 | 8076394 | [T/C] | - | URR | - |
| CWSNP4999 | Ca-Kabuli-Chr6 | 8076446 | [G/A] | - | URR | - |
| CWSNP5000 | Ca-Kabuli-Chr6 | 8081800 | [C/G] | - | DRR | - |
| CWSNP5001 | Ca-Kabuli-Chr6 | 8084950 | [T/A] | - | Intergenic | - |
| CWSNP5002 | Ca-Kabuli-Chr6 | 8090739 | [T/A] | Ca09562 | Non-Synonymous-CDS | IQ motif, EF-hand binding site |
| CWSNP5003 | Ca-Kabuli-Chr6 | 8090980 | [A/G] | Ca09562 | Synonymous-CDS | IQ motif, EF-hand binding site |
| CWSNP5004 | Ca-Kabuli-Chr6 | 8091050 | [A/G] | Ca09562 | Non-Synonymous-CDS | IQ motif, EF-hand binding site |
| CWSNP5005 | Ca-Kabuli-Chr6 | 8139446 | [C/A] | - | DRR | - |
| CWSNP5006 | Ca-Kabuli-Chr6 | 8166317 | [T/C] | - | URR | - |
| CWSNP5007 | Ca-Kabuli-Chr6 | 8166339 | [T/C] | - | URR | - |
| CWSNP5008 | Ca-Kabuli-Chr6 | 8166435 | [A/G] | - | URR | - |

| SNP IDs | Chromosomes | Physical positions (bp) | SNPs | Gene accession IDs | Sequence components of genes | Putative functions |
|---|---|---|---|---|---|---|
| CWSNP5009 | Ca-Kabuli-Chr6 | 8166462 | [A/T] | - | URR | - |
| CWSNP5010 | Ca-Kabuli-Chr6 | 8170583 | [C/T] | Ca09552 | Intron | Enhancerofpolycomb-like,N-terminal |
| CWSNP5011 | Ca-Kabuli-Chr6 | 8170633 | [A/G] | Ca09552 | Intron | Enhancerofpolycomb-like,N-terminal |
| CWSNP5012 | Ca-Kabuli-Chr6 | 8179815 | [T/C] | - | Intergenic | - |
| CWSNP5013 | Ca-Kabuli-Chr6 | 8221204 | [C/A] | Ca09549 | Non-Synonymous-CDS | DNA-dependent ATPase MCM |
| CWSNP5014 | Ca-Kabuli-Chr6 | 8223986 | [C/G] | Ca09549 | Non-Synonymous-CDS | DNA-dependent ATPase MCM |
| CWSNP5015 | Ca-Kabuli-Chr6 | 8237286 | [A/G] | Ca09547 | Intron | RNA recognition motif domain |
| CWSNP5016 | Ca-Kabuli-Chr6 | 8246187 | [A/C] | - | Intergenic | - |
| CWSNP5017 | Ca-Kabuli-Chr6 | 8710064 | [A/T] | - | Intergenic | - |
| CWSNP5018 | Ca-Kabuli-Chr6 | 8787557 | [T/C] | Ca16756 | Non-Synonymous-CDS | Protein kinase, catalytic domain |
| CWSNP5019 | Ca-Kabuli-Chr6 | 8787562 | [C/T] | Ca16756 | Non-Synonymous-CDS | Protein kinase, catalytic domain |
| CWSNP5020 | Ca-Kabuli-Chr6 | 8787564 | [G/T] | Ca16756 | Synonymous-CDS | Protein kinase, catalytic domain |
| CWSNP5021 | Ca-Kabuli-Chr6 | 8787568 | [G/T] | Ca16756 | Non-Synonymous-CDS | Protein kinase, catalytic domain |
| CWSNP5022 | Ca-Kabuli-Chr6 | 8787572 | [T/A] | Ca16756 | Non-Synonymous-CDS | Protein kinase, catalytic domain |
| CWSNP5023 | Ca-Kabuli-Chr6 | 8787573 | [G/A] | Ca16756 | Synonymous-CDS | Protein kinase, catalytic domain |
| CWSNP5024 | Ca-Kabuli-Chr6 | 8968068 | [G/A] | Ca16741 | Intron | Phosphatidylinositol-4-phosphate5-kinase,core |

| SNP IDs | Chromosomes | Physical positions (bp) | SNPs | Gene accession IDs | Sequence components of genes | Putative functions |
|---|---|---|---|---|---|---|
| CWSNP5025 | Ca-Kabuli-Chr6 | 9227767 | [C/A] | - | Intergenic | - |
| CWSNP5026 | Ca-Kabuli-Chr6 | 9228004 | [C/A] | - | Intergenic | - |
| CWSNP5027 | Ca-Kabuli-Chr6 | 9324122 | [A/C] | Ca08667 | Non-Synonymous-CDS | Homeobox |
| CWSNP5028 | Ca-Kabuli-Chr6 | 9523228 | [T/C] | Ca08648 | Intron | RNA-processing protein,HAT helix |
| CWSNP5029 | Ca-Kabuli-Chr6 | 9814378 | [G/T] | Ca08611 | Intron | ProteinTransporter,Pam16 |
| CWSNP5030 | Ca-Kabuli-Chr6 | 9834199 | [T/C] | Ca08608 | Synonymous-CDS | WD40 repeat |
| CWSNP5031 | Ca-Kabuli-Chr6 | 9834198 | [C/A] | Ca08608 | Non-Synonymous-CDS | WD40 repeat |
| CWSNP5032 | Ca-Kabuli-Chr6 | 9834187 | [A/T] | Ca08608 | Non-Synonymous-CDS | WD40 repeat |
| CWSNP5033 | Ca-Kabuli-Chr6 | 9834184 | [A/T] | Ca08608 | Synonymous-CDS | WD40 repeat |
| CWSNP5034 | Ca-Kabuli-Chr6 | 9834183 | [C/T] | Ca08608 | Non-Synonymous-CDS | WD40 repeat |
| CWSNP5035 | Ca-Kabuli-Chr6 | 9841836 | [G/A] | - | DRR | - |
| CWSNP5036 | Ca-Kabuli-Chr6 | 9872657 | [C/T] | Ca08602 | Non-Synonymous-CDS | Xanthine/uracil/vitaminCpermease |
| CWSNP5037 | Ca-Kabuli-Chr6 | 9872988 | [A/T] | Ca08602 | Intron | Xanthine/uracil/vitaminCpermease |
| CWSNP5038 | Ca-Kabuli-Chr6 | 9959686 | [T/C] | Ca08594 | Synonymous-CDS | Preprotein translocase Sec, Sec61-beta subunit |
| CWSNP5039 | Ca-Kabuli-Chr6 | 10020177 | [C/G] | - | DRR | - |
| CWSNP5040 | Ca-Kabuli-Chr6 | 10020187 | [A/G] | - | DRR | - |

| SNP IDs | Chromosomes | Physical positions (bp) | SNPs | Gene accession IDs | Sequence components of genes | Putative functions |
|---|---|---|---|---|---|---|
| CWSNP5041 | Ca-Kabuli-Chr6 | 10025115 | [A/C] | - | DRR | - |
| CWSNP5042 | Ca-Kabuli-Chr6 | 10025113 | [C/G] | - | DRR | - |
| CWSNP5043 | Ca-Kabuli-Chr6 | 10028504 | [A/G] | - | DRR | - |
| CWSNP5044 | Ca-Kabuli-Chr6 | 10028508 | [G/C] | - | Intergenic | - |
| CWSNP5045 | Ca-Kabuli-Chr6 | 10045644 | [A/C] | Ca08587 | Synonymous-CDS | AMP-dependent synthetase/ligase |
| CWSNP5046 | Ca-Kabuli-Chr6 | 10045844 | [A/G] | - | Intergenic | - |
| CWSNP5047 | Ca-Kabuli-Chr6 | 10088984 | [A/G] | - | DRR | - |
| CWSNP5048 | Ca-Kabuli-Chr6 | 10100863 | [C/G] | Ca08581 | Synonymous-CDS | HEAT |
| CWSNP5049 | Ca-Kabuli-Chr6 | 10111488 | [C/G] | Ca08580 | Intron | Peptidyl-tRNAhydrolase |
| CWSNP5050 | Ca-Kabuli-Chr6 | 10111520 | [G/C] | Ca08580 | Intron | Peptidyl-tRNAhydrolase |
| CWSNP5051 | Ca-Kabuli-Chr6 | 10111870 | [A/G] | Ca08580 | Intron | Peptidyl-tRNAhydrolase |
| CWSNP5052 | Ca-Kabuli-Chr6 | 10116403 | [A/T] | Ca08580 | Intron | Peptidyl-tRNAhydrolase |
| CWSNP5053 | Ca-Kabuli-Chr6 | 10116475 | [C/A] | Ca08580 | Intron | Peptidyl-tRNAhydrolase |
| CWSNP5054 | Ca-Kabuli-Chr6 | 10151973 | [A/G] | Ca08577 | Intron | Protein of unknown function DUF616 |
| CWSNP5055 | Ca-Kabuli-Chr6 | 10158850 | [T/C] | Ca08576 | Intron | Pheophorbideaoxygenase |
| CWSNP5056 | Ca-Kabuli-Chr6 | 10188400 | [G/T] | - | Intergenic | - |

| SNP IDs | Chromosomes | Physical positions (bp) | SNPs | Gene accession IDs | Sequence components of genes | Putative functions |
|---|---|---|---|---|---|---|
| CWSNP5057 | Ca-Kabuli-Chr6 | 10215262 | [T/G] | - | DRR | - |
| CWSNP5058 | Ca-Kabuli-Chr6 | 10230617 | [A/G] | Ca08566 | Intron | Metallo-dependent phosphatase |
| CWSNP5059 | Ca-Kabuli-Chr6 | 10230657 | [A/G] | Ca08566 | Intron | Metallo-dependent phosphatase |
| CWSNP5060 | Ca-Kabuli-Chr6 | 10231092 | [T/G] | Ca08566 | Synonymous-CDS | Metallo-dependent phosphatase |
| CWSNP5061 | Ca-Kabuli-Chr6 | 10231095 | [G/T] | Ca08566 | Synonymous-CDS | Metallo-dependent phosphatase |
| CWSNP5062 | Ca-Kabuli-Chr6 | 10234241 | [A/G] | Ca08566 | Intron | Metallo-dependent phosphatase |
| CWSNP5063 | Ca-Kabuli-Chr6 | 10259614 | [C/T] | - | DRR | - |
| CWSNP5064 | Ca-Kabuli-Chr6 | 10260274 | [C/T] | Ca08563 | Synonymous-CDS | Mediator complex,subunit Med10 |
| CWSNP5065 | Ca-Kabuli-Chr6 | 10314993 | [C/G] | - | Intergenic | - |
| CWSNP5066 | Ca-Kabuli-Chr6 | 10395411 | [G/A] | Ca08553 | Intron | Small ubiquitin-related modifier,SUMO |
| CWSNP5067 | Ca-Kabuli-Chr6 | 10395379 | [T/C] | Ca08553 | Intron | Small ubiquitin-related modifier,SUMO |
| CWSNP5068 | Ca-Kabuli-Chr6 | 10395356 | [T/A] | Ca08553 | Intron | Small ubiquitin-related modifier,SUMO |
| CWSNP5069 | Ca-Kabuli-Chr6 | 10441736 | [A/C] | Ca08551 | Non-Synonymous-CDS | - |
| CWSNP5070 | Ca-Kabuli-Chr6 | 10441705 | [T/C] | - | Intergenic | - |
| CWSNP5071 | Ca-Kabuli-Chr6 | 10442148 | [A/C] | Ca08551 | Synonymous-CDS | - |
| CWSNP5072 | Ca-Kabuli-Chr6 | 10445185 | [C/T] | Ca08551 | Intron | - |

| SNP IDs | Chromosomes | Physical positions (bp) | SNPs | Gene accession IDs | Sequence components of genes | Putative functions |
|---|---|---|---|---|---|---|
| CWSNP5073 | Ca-Kabuli-Chr6 | 10445274 | [G/A] | Ca08551 | Intron | - |
| CWSNP5074 | Ca-Kabuli-Chr6 | 10456774 | [A/G] | Ca08551 | Synonymous-CDS | - |
| CWSNP5075 | Ca-Kabuli-Chr6 | 10456854 | [G/T] | Ca08551 | Synonymous-CDS | - |
| CWSNP5076 | Ca-Kabuli-Chr6 | 10494134 | [C/T] | Ca08547 | Intron | D-galactoside/L-rhamnose binding SUEL lectin domain |
| CWSNP5077 | Ca-Kabuli-Chr6 | 10494057 | [C/A] | Ca08547 | Intron | D-galactoside/L-rhamnose binding SUEL lectin domain |
| CWSNP5078 | Ca-Kabuli-Chr6 | 10494055 | [T/A] | Ca08547 | Intron | D-galactoside/L-rhamnose binding SUEL lectin domain |
| CWSNP5079 | Ca-Kabuli-Chr6 | 10495747 | [G/T] | Ca08547 | Intron | D-galactoside/L-rhamnose binding SUEL lectin domain |
| CWSNP5080 | Ca-Kabuli-Chr6 | 10510996 | [T/C] | - | URR | - |
| CWSNP5081 | Ca-Kabuli-Chr6 | 10520958 | [C/T] | Ca08543 | Intron | RNA recognition motif domain |
| CWSNP5082 | Ca-Kabuli-Chr6 | 10531399 | [C/T] | Ca08542 | Intron | RNA recognition motif domain |
| CWSNP5083 | Ca-Kabuli-Chr6 | 10532182 | [A/T] | Ca08542 | Non-Synonymous-CDS | RNA recognition motif domain |
| CWSNP5084 | Ca-Kabuli-Chr6 | 10532739 | [A/G] | Ca08542 | Synonymous-CDS | RNA recognition motif domain |
| CWSNP5085 | Ca-Kabuli-Chr6 | 10539918 | [A/C] | - | Intergenic | - |
| CWSNP5086 | Ca-Kabuli-Chr6 | 10539977 | [C/T] | - | Intergenic | - |
| CWSNP5087 | Ca-Kabuli-Chr6 | 10539994 | [A/C] | - | Intergenic | - |
| CWSNP5088 | Ca-Kabuli-Chr6 | 10567518 | [A/G] | - | Intergenic | - |

| SNP IDs | Chromosomes | Physical positions (bp) | SNPs | Gene accession IDs | Sequence components of genes | Putative functions |
|---|---|---|---|---|---|---|
| CWSNP5089 | Ca-Kabuli-Chr6 | 10588030 | [C/T] | - | Intergenic | - |
| CWSNP5090 | Ca-Kabuli-Chr6 | 10613546 | [A/G] | Ca08536 | Synonymous-CDS | Heatshockfactor(HSF)-type,DNA-binding |
| CWSNP5091 | Ca-Kabuli-Chr6 | 10613612 | [A/G] | Ca08536 | Synonymous-CDS | Heatshockfactor(HSF)-type,DNA-binding |
| CWSNP5092 | Ca-Kabuli-Chr6 | 10667559 | [T/C] | - | Intergenic | - |
| CWSNP5093 | Ca-Kabuli-Chr6 | 10670368 | [G/A] | Ca08530 | Intron | Aspartate/glutamate/uridylatekinase |
| CWSNP5094 | Ca-Kabuli-Chr6 | 10670409 | [G/A] | Ca08530 | Intron | Aspartate/glutamate/uridylatekinase |
| CWSNP5095 | Ca-Kabuli-Chr6 | 10670482 | [C/T] | Ca08530 | Intron | Aspartate/glutamate/uridylatekinase |
| CWSNP5096 | Ca-Kabuli-Chr6 | 10670582 | [G/A] | Ca08530 | Intron | Aspartate/glutamate/uridylatekinase |
| CWSNP5097 | Ca-Kabuli-Chr6 | 10670694 | [G/A] | Ca08530 | Intron | Aspartate/glutamate/uridylatekinase |
| CWSNP5098 | Ca-Kabuli-Chr6 | 10670773 | [A/C] | Ca08530 | Intron | Aspartate/glutamate/uridylatekinase |
| CWSNP5099 | Ca-Kabuli-Chr6 | 10670959 | [T/C] | Ca08530 | Intron | Aspartate/glutamate/uridylatekinase |
| CWSNP5100 | Ca-Kabuli-Chr6 | 10671458 | [T/C] | Ca08530 | Intron | Aspartate/glutamate/uridylatekinase |
| CWSNP5101 | Ca-Kabuli-Chr6 | 10671444 | [A/C] | Ca08530 | Intron | Aspartate/glutamate/uridylatekinase |
| CWSNP5102 | Ca-Kabuli-Chr6 | 10672468 | [C/T] | Ca08530 | Intron | Aspartate/glutamate/uridylatekinase |
| CWSNP5103 | Ca-Kabuli-Chr6 | 10677469 | [A/G] | Ca08530 | Intron | Aspartate/glutamate/uridylatekinase |
| CWSNP5104 | Ca-Kabuli-Chr6 | 10677501 | [C/T] | Ca08530 | Intron | Aspartate/glutamate/uridylatekinase |

| SNP IDs | Chromosomes | Physical positions (bp) | SNPs | Gene accession IDs | Sequence components of genes | Putative functions |
|---|---|---|---|---|---|---|
| CWSNP5105 | Ca-Kabuli-Chr6 | 10682221 | [G/T] | - | Intergenic | - |
| CWSNP5106 | Ca-Kabuli-Chr6 | 10682201 | [C/G] | - | Intergenic | - |
| CWSNP5107 | Ca-Kabuli-Chr6 | 10683193 | [C/T] | - | DRR | - |
| CWSNP5108 | Ca-Kabuli-Chr6 | 10696986 | [A/T] | Ca08528 | Intron | Protein kinase, catalytic domain |
| CWSNP5109 | Ca-Kabuli-Chr6 | 10696990 | [T/C] | Ca08528 | Intron | Protein kinase, catalytic domain |
| CWSNP5110 | Ca-Kabuli-Chr6 | 10697064 | [T/A] | Ca08528 | Intron | Protein kinase, catalytic domain |
| CWSNP5111 | Ca-Kabuli-Chr6 | 10716986 | [G/A] | Ca08526 | Synonymous-CDS | - |
| CWSNP5112 | Ca-Kabuli-Chr6 | 10716968 | [G/A] | Ca08526 | Synonymous-CDS | - |
| CWSNP5113 | Ca-Kabuli-Chr6 | 10744029 | [C/A] | - | Intergenic | - |
| CWSNP5114 | Ca-Kabuli-Chr6 | 10743996 | [A/T] | - | Intergenic | - |
| CWSNP5115 | Ca-Kabuli-Chr6 | 10785480 | [T/G] | - | Intergenic | - |
| CWSNP5116 | Ca-Kabuli-Chr6 | 10870187 | [T/G] | Ca08511 | Non-Synonymous-CDS | IQ motif, EF-hand binding site |
| CWSNP5117 | Ca-Kabuli-Chr6 | 10870534 | [A/G] | Ca08511 | Synonymous-CDS | IQ motif, EF-hand binding site |
| CWSNP5118 | Ca-Kabuli-Chr6 | 10880791 | [C/T] | Ca08509 | Intron | Signal transduction response regulator,receiver domain |
| CWSNP5119 | Ca-Kabuli-Chr6 | 10880819 | [T/G] | Ca08509 | Intron | Signal transduction response regulator,receiver domain |
| CWSNP5120 | Ca-Kabuli-Chr6 | 10881002 | [A/G] | Ca08509 | Intron | Signal transduction response regulator,receiver domain |

| SNP IDs | Chromosomes | Physical positions (bp) | SNPs | Gene accession IDs | Sequence components of genes | Putative functions |
|---|---|---|---|---|---|---|
| CWSNP5121 | Ca-Kabuli-Chr6 | 10880985 | [C/T] | Ca08509 | Intron | Signal transduction response regulator,receiver domain |
| CWSNP5122 | Ca-Kabuli-Chr6 | 10880962 | [G/T] | Ca08509 | Intron | Signal transduction response regulator,receiver domain |
| CWSNP5123 | Ca-Kabuli-Chr6 | 10991652 | [C/A] | Ca20467 | Non-Synonymous-CDS | Zinc finger,MYND-type |
| CWSNP5124 | Ca-Kabuli-Chr6 | 10991717 | [T/A] | Ca20467 | Non-Synonymous-CDS | Zinc finger,MYND-type |
| CWSNP5125 | Ca-Kabuli-Chr6 | 10991737 | [A/C] | Ca20467 | Synonymous-CDS | Zinc finger,MYND-type |
| CWSNP5126 | Ca-Kabuli-Chr6 | 11011847 | [C/G] | Ca20469 | Non-Synonymous-CDS | Rubber elongation factor |
| CWSNP5127 | Ca-Kabuli-Chr6 | 11131010 | [T/A] | Ca20479 | Intron | Glycosidehydrolase,family63 |
| CWSNP5128 | Ca-Kabuli-Chr6 | 11131464 | [G/A] | Ca20479 | Intron | Glycosidehydrolase,family63 |
| CWSNP5129 | Ca-Kabuli-Chr6 | 11188803 | [G/A] | Ca20485 | Non-Synonymous-CDS | Protein kinase, catalytic domain |
| CWSNP5130 | Ca-Kabuli-Chr6 | 11189051 | [A/G] | Ca20485 | Synonymous-CDS | Protein kinase, catalytic domain |
| CWSNP5131 | Ca-Kabuli-Chr6 | 11229102 | [C/T] | Ca20489 | Synonymous-CDS | Ankyrin repeat |
| CWSNP5132 | Ca-Kabuli-Chr6 | 11229302 | [A/G] | Ca20489 | Non-Synonymous-CDS | Ankyrin repeat |
| CWSNP5133 | Ca-Kabuli-Chr6 | 11229388 | [C/T] | Ca20489 | Non-Synonymous-CDS | Ankyrin repeat |
| CWSNP5134 | Ca-Kabuli-Chr6 | 11300549 | [A/C] | Ca20493 | Synonymous-CDS | Signal recognition particle,SRP72 subunit,RNA-binding |
| CWSNP5135 | Ca-Kabuli-Chr6 | 11302037 | [A/C] | Ca20493 | Non-Synonymous-CDS | Signal recognition particle,SRP72 subunit,RNA-binding |
| CWSNP5136 | Ca-Kabuli-Chr6 | 11302033 | [T/C] | Ca20493 | Non-Synonymous-CDS | Signal recognition particle,SRP72 subunit,RNA-binding |

| SNP IDs | Chromosomes | Physical positions (bp) | SNPs | Gene accession IDs | Sequence components of genes | Putative functions |
|---|---|---|---|---|---|---|
| CWSNP5137 | Ca-Kabuli-Chr6 | 11314581 | [T/C] | Ca20494 | Synonymous-CDS | Pentatrico peptide repeat |
| CWSNP5138 | Ca-Kabuli-Chr6 | 11508828 | [C/T] | Ca23431 | Non-Synonymous-CDS | Heat shock protein DnaJ,N-terminal |
| CWSNP5139 | Ca-Kabuli-Chr6 | 11508824 | [T/G] | Ca23431 | Non-Synonymous-CDS | Heat shock protein DnaJ,N-terminal |
| CWSNP5140 | Ca-Kabuli-Chr6 | 11508817 | [G/A] | Ca23431 | Non-Synonymous-CDS | Heat shock protein DnaJ,N-terminal |
| CWSNP5141 | Ca-Kabuli-Chr6 | 11508816 | [A/C] | Ca23431 | Non-Synonymous-CDS | Heat shock protein DnaJ,N-terminal |
| CWSNP5142 | Ca-Kabuli-Chr6 | 11543618 | [T/G] | - | URR | - |
| CWSNP5143 | Ca-Kabuli-Chr6 | 11665233 | [G/A] | Ca25061 | Synonymous-CDS | Amineoxidase |
| CWSNP5144 | Ca-Kabuli-Chr6 | 11665338 | [T/G] | Ca25061 | Synonymous-CDS | Amineoxidase |
| CWSNP5145 | Ca-Kabuli-Chr6 | 12437275 | [A/T] | Ca05025 | Non-Synonymous-CDS | AUX/IAA protein |
| CWSNP5146 | Ca-Kabuli-Chr6 | 12437285 | [A/G] | Ca05025 | Synonymous-CDS | AUX/IAA protein |
| CWSNP5147 | Ca-Kabuli-Chr6 | 12437288 | [G/A] | Ca05025 | Synonymous-CDS | AUX/IAA protein |
| CWSNP5148 | Ca-Kabuli-Chr6 | 12437319 | [C/T] | Ca05025 | Non-Synonymous-CDS | AUX/IAA protein |
| CWSNP5149 | Ca-Kabuli-Chr6 | 12532947 | [T/A] | - | Intergenic | - |
| CWSNP5150 | Ca-Kabuli-Chr6 | 12535733 | [C/G] | Ca05031 | Non-Synonymous-CDS | Sugar/inositol transporter |
| CWSNP5151 | Ca-Kabuli-Chr6 | 12799890 | [T/G] | - | DRR | - |
| CWSNP5152 | Ca-Kabuli-Chr6 | 12893335 | [C/A] | - | Intergenic | - |

| SNP IDs | Chromosomes | Physical positions (bp) | SNPs | Gene accession IDs | Sequence components of genes | Putative functions |
|---|---|---|---|---|---|---|
| CWSNP5153 | Ca-Kabuli-Chr6 | 12893424 | [C/T] | - | Intergenic | - |
| CWSNP5154 | Ca-Kabuli-Chr6 | 12906599 | [G/A] | Ca05064 | Intron | Protein kinase, catalytic domain |
| CWSNP5155 | Ca-Kabuli-Chr6 | 12906680 | [T/C] | Ca05064 | Intron | Protein kinase, catalytic domain |
| CWSNP5156 | Ca-Kabuli-Chr6 | 12906669 | [G/A] | Ca05064 | Intron | Protein kinase, catalytic domain |
| CWSNP5157 | Ca-Kabuli-Chr6 | 12973765 | [A/G] | - | Intergenic | - |
| CWSNP5158 | Ca-Kabuli-Chr6 | 12977129 | [A/G] | - | Intergenic | - |
| CWSNP5159 | Ca-Kabuli-Chr6 | 12977196 | [A/G] | - | Intergenic | - |
| CWSNP5160 | Ca-Kabuli-Chr6 | 12977204 | [T/G] | - | Intergenic | - |
| CWSNP5161 | Ca-Kabuli-Chr6 | 12985896 | [A/C] | - | URR | - |
| CWSNP5162 | Ca-Kabuli-Chr6 | 12985908 | [C/G] | - | URR | - |
| CWSNP5163 | Ca-Kabuli-Chr6 | 12985910 | [C/T] | - | URR | - |
| CWSNP5164 | Ca-Kabuli-Chr6 | 12986776 | [A/G] | - | URR | - |
| CWSNP5165 | Ca-Kabuli-Chr6 | 12986833 | [A/C] | - | URR | - |
| CWSNP5166 | Ca-Kabuli-Chr6 | 13136955 | [G/A] | Ca05086 | Intron | Cyclicnucleotide-binding domain |
| CWSNP5167 | Ca-Kabuli-Chr6 | 13188119 | [A/C] | - | URR | - |
| CWSNP5168 | Ca-Kabuli-Chr6 | 13340012 | [A/G] | - | DRR | - |

| SNP IDs | Chromosomes | Physical positions (bp) | SNPs | Gene accession IDs | Sequence components of genes | Putative functions |
|---|---|---|---|---|---|---|
| CWSNP5169 | Ca-Kabuli-Chr6 | 13340762 | [G/A] | - | DRR | - |
| CWSNP5170 | Ca-Kabuli-Chr6 | 13413848 | [C/G] | - | Intergenic | - |
| CWSNP5171 | Ca-Kabuli-Chr6 | 13483841 | [T/G] | - | Intergenic | - |
| CWSNP5172 | Ca-Kabuli-Chr6 | 13565990 | [C/T] | Ca05127 | Non-Synonymous-CDS | HECT |
| CWSNP5173 | Ca-Kabuli-Chr6 | 13566007 | [C/T] | Ca05127 | Synonymous-CDS | HECT |
| CWSNP5174 | Ca-Kabuli-Chr6 | 13566012 | [T/G] | Ca05127 | Non-Synonymous-CDS | HECT |
| CWSNP5175 | Ca-Kabuli-Chr6 | 13566041 | [C/T] | Ca05127 | Non-Synonymous-CDS | HECT |
| CWSNP5176 | Ca-Kabuli-Chr6 | 13566110 | [A/G] | Ca05127 | Non-Synonymous-CDS | HECT |
| CWSNP5177 | Ca-Kabuli-Chr6 | 13566136 | [A/C] | Ca05127 | Synonymous-CDS | HECT |
| CWSNP5178 | Ca-Kabuli-Chr6 | 13572236 | [A/T] | Ca05127 | Intron | HECT |
| CWSNP5179 | Ca-Kabuli-Chr6 | 13572221 | [A/C] | Ca05127 | Intron | HECT |
| CWSNP5180 | Ca-Kabuli-Chr6 | 13576963 | [T/A] | Ca05127 | Intron | HECT |
| CWSNP5181 | Ca-Kabuli-Chr6 | 13591590 | [T/G] | - | URR | - |
| CWSNP5182 | Ca-Kabuli-Chr6 | 13593600 | [T/C] | - | URR | - |
| CWSNP5183 | Ca-Kabuli-Chr6 | 13619538 | [C/T] | Ca05131 | Non-Synonymous-CDS | HECT |
| CWSNP5184 | Ca-Kabuli-Chr6 | 13619555 | [C/T] | Ca05131 | Synonymous-CDS | HECT |

| SNP IDs | Chromosomes | Physical positions (bp) | SNPs | Gene accession IDs | Sequence components of genes | Putative functions |
|---|---|---|---|---|---|---|
| CWSNP5185 | Ca-Kabuli-Chr6 | 13619560 | [T/G] | Ca05131 | Non-Synonymous-CDS | HECT |
| CWSNP5186 | Ca-Kabuli-Chr6 | 13619589 | [C/T] | Ca05131 | Non-Synonymous-CDS | HECT |
| CWSNP5187 | Ca-Kabuli-Chr6 | 13619658 | [A/G] | Ca05131 | Non-Synonymous-CDS | HECT |
| CWSNP5188 | Ca-Kabuli-Chr6 | 13619684 | [A/C] | Ca05131 | Synonymous-CDS | HECT |
| CWSNP5189 | Ca-Kabuli-Chr6 | 13622292 | [C/T] | Ca05131 | Non-Synonymous-CDS | HECT |
| CWSNP5190 | Ca-Kabuli-Chr6 | 13625388 | [A/G] | - | Intergenic | - |
| CWSNP5191 | Ca-Kabuli-Chr6 | 13668916 | [T/A] | - | Intergenic | - |
| CWSNP5192 | Ca-Kabuli-Chr6 | 13668995 | [A/G] | - | Intergenic | - |
| CWSNP5193 | Ca-Kabuli-Chr6 | 13669052 | [G/C] | - | Intergenic | - |
| CWSNP5194 | Ca-Kabuli-Chr6 | 13669049 | [C/T] | - | Intergenic | - |
| CWSNP5195 | Ca-Kabuli-Chr6 | 13669048 | [T/A] | - | Intergenic | - |
| CWSNP5196 | Ca-Kabuli-Chr6 | 13669042 | [A/G] | - | Intergenic | - |
| CWSNP5197 | Ca-Kabuli-Chr6 | 13669018 | [G/A] | - | Intergenic | - |
| CWSNP5198 | Ca-Kabuli-Chr6 | 13669006 | [G/C] | - | Intergenic | - |
| CWSNP5199 | Ca-Kabuli-Chr6 | 13827484 | [C/T] | Ca05146 | Synonymous-CDS | Aspartate carbamoyltransferase,eukaryotic |
| CWSNP5200 | Ca-Kabuli-Chr6 | 13827452 | [C/G] | Ca05146 | Intron | Aspartate carbamoyltransferase,eukaryotic |

| SNP IDs | Chromosomes | Physical positions (bp) | SNPs | Gene accession IDs | Sequence components of genes | Putative functions |
|---|---|---|---|---|---|---|
| CWSNP5201 | Ca-Kabuli-Chr6 | 13913539 | [A/G] | Ca05156 | Intron | DNA-dependent ATPase MCM |
| CWSNP5202 | Ca-Kabuli-Chr6 | 13948732 | [G/T] | - | Intergenic | - |
| CWSNP5203 | Ca-Kabuli-Chr6 | 13948778 | [A/C] | - | Intergenic | - |
| CWSNP5204 | Ca-Kabuli-Chr6 | 13986559 | [A/G] | - | DRR | - |
| CWSNP5205 | Ca-Kabuli-Chr6 | 14042859 | [A/T] | - | Intergenic | - |
| CWSNP5206 | Ca-Kabuli-Chr6 | 14067312 | [A/C] | - | DRR | - |
| CWSNP5207 | Ca-Kabuli-Chr6 | 14067326 | [C/T] | - | DRR | - |
| CWSNP5208 | Ca-Kabuli-Chr6 | 14112542 | [A/G] | Ca05177 | Intron | Stressup-regulatedNod19 |
| CWSNP5209 | Ca-Kabuli-Chr6 | 14148771 | [A/G] | - | Intergenic | - |
| CWSNP5210 | Ca-Kabuli-Chr6 | 14155543 | [C/T] | - | URR | - |
| CWSNP5211 | Ca-Kabuli-Chr6 | 14155662 | [T/C] | - | URR | - |
| CWSNP5212 | Ca-Kabuli-Chr6 | 14155643 | [A/C] | - | URR | - |
| CWSNP5213 | Ca-Kabuli-Chr6 | 14161084 | [G/C] | Ca05182 | Intron | N-acetylglucosaminyl phosphatidyl inositoldeacetylase |
| CWSNP5214 | Ca-Kabuli-Chr6 | 14201001 | [T/C] | - | URR | - |
| CWSNP5215 | Ca-Kabuli-Chr6 | 14200997 | [G/A] | - | URR | - |
| CWSNP5216 | Ca-Kabuli-Chr6 | 14204400 | [T/C] | - | URR | - |

| SNP IDs | Chromosomes | Physical positions (bp) | SNPs | Gene accession IDs | Sequence components of genes | Putative functions |
|---|---|---|---|---|---|---|
| CWSNP5217 | Ca-Kabuli-Chr6 | 14204469 | [G/A] | - | URR | - |
| CWSNP5218 | Ca-Kabuli-Chr6 | 14254394 | [A/G] | - | DRR | - |
| CWSNP5219 | Ca-Kabuli-Chr6 | 14309042 | [C/T] | - | DRR | - |
| CWSNP5220 | Ca-Kabuli-Chr6 | 14330533 | [T/C] | - | URR | - |
| CWSNP5221 | Ca-Kabuli-Chr6 | 14332018 | [A/C] | - | URR | - |
| CWSNP5222 | Ca-Kabuli-Chr6 | 14332404 | [A/G] | - | URR | - |
| CWSNP5223 | Ca-Kabuli-Chr6 | 14353598 | [C/T] | - | DRR | - |
| CWSNP5224 | Ca-Kabuli-Chr6 | 14353624 | [G/A] | - | DRR | - |
| CWSNP5225 | Ca-Kabuli-Chr6 | 14371493 | [C/T] | Ca05207 | Synonymous-CDS | Sec1-like protein |
| CWSNP5226 | Ca-Kabuli-Chr6 | 14371530 | [C/T] | Ca05207 | Synonymous-CDS | Sec1-like protein |
| CWSNP5227 | Ca-Kabuli-Chr6 | 14375135 | [G/A] | - | Intergenic | - |
| CWSNP5228 | Ca-Kabuli-Chr6 | 14375141 | [G/C] | - | Intergenic | - |
| CWSNP5229 | Ca-Kabuli-Chr6 | 14375172 | [A/G] | - | Intergenic | - |
| CWSNP5230 | Ca-Kabuli-Chr6 | 14375196 | [G/A] | - | Intergenic | - |
| CWSNP5231 | Ca-Kabuli-Chr6 | 14375207 | [C/T] | - | Intergenic | - |
| CWSNP5232 | Ca-Kabuli-Chr6 | 14416997 | [A/G] | Ca05213 | Non-Synonymous-CDS | Domain of unknown function DUF676,hydrolase-like |

| SNP IDs | Chromosomes | Physical positions (bp) | SNPs | Gene accession IDs | Sequence components of genes | Putative functions |
|---|---|---|---|---|---|---|
| CWSNP5233 | Ca-Kabuli-Chr6 | 14416981 | [A/G] | Ca05213 | Synonymous-CDS | Domain of unknown function DUF676,hydrolase-like |
| CWSNP5234 | Ca-Kabuli-Chr6 | 14419832 | [C/T] | Ca05213 | Synonymous-CDS | Domain of unknown function DUF676,hydrolase-like |
| CWSNP5235 | Ca-Kabuli-Chr6 | 14526540 | [A/T] | - | DRR | - |
| CWSNP5236 | Ca-Kabuli-Chr6 | 14589480 | [T/C] | - | Intergenic | - |
| CWSNP5237 | Ca-Kabuli-Chr6 | 14589520 | [G/C] | - | Intergenic | - |
| CWSNP5238 | Ca-Kabuli-Chr6 | 14589744 | [C/T] | Ca05228 | Intron | Dynamin,GTPase domain |
| CWSNP5239 | Ca-Kabuli-Chr6 | 14589807 | [T/G] | Ca05228 | Synonymous-CDS | Dynamin,GTPase domain |
| CWSNP5240 | Ca-Kabuli-Chr6 | 14589873 | [T/C] | Ca05228 | Synonymous-CDS | Dynamin,GTPase domain |
| CWSNP5241 | Ca-Kabuli-Chr6 | 14606892 | [A/C] | - | URR | - |
| CWSNP5242 | Ca-Kabuli-Chr6 | 14628213 | [A/T] | Ca05233 | Synonymous-CDS | Glycosidehydrolase,family3,N-terminal |
| CWSNP5243 | Ca-Kabuli-Chr6 | 14628215 | [T/C] | Ca05233 | Non-Synonymous-CDS | Glycosidehydrolase,family3,N-terminal |
| CWSNP5244 | Ca-Kabuli-Chr6 | 14628216 | [A/C] | Ca05233 | Synonymous-CDS | Glycosidehydrolase,family3,N-terminal |
| CWSNP5245 | Ca-Kabuli-Chr6 | 14753191 | [C/T] | - | Intergenic | - |
| CWSNP5246 | Ca-Kabuli-Chr6 | 14753190 | [G/A] | - | Intergenic | - |
| CWSNP5247 | Ca-Kabuli-Chr6 | 14753158 | [A/G] | - | Intergenic | - |
| CWSNP5248 | Ca-Kabuli-Chr6 | 14753113 | [C/A] | - | Intergenic | - |

| SNP IDs | Chromosomes | Physical positions (bp) | SNPs | Gene accession IDs | Sequence components of genes | Putative functions |
|---|---|---|---|---|---|---|
| CWSNP5249 | Ca-Kabuli-Chr6 | 14753145 | [C/A] | - | Intergenic | - |
| CWSNP5250 | Ca-Kabuli-Chr6 | 14762493 | [A/G] | Ca05249 | Non-Synonymous-CDS | K+ potassium transporter |
| CWSNP5251 | Ca-Kabuli-Chr6 | 14786015 | [T/G] | - | Intergenic | - |
| CWSNP5252 | Ca-Kabuli-Chr6 | 14857722 | [C/A] | - | URR | - |
| CWSNP5253 | Ca-Kabuli-Chr6 | 14857709 | [A/G] | - | URR | - |
| CWSNP5254 | Ca-Kabuli-Chr6 | 14857680 | [G/A] | - | URR | - |
| CWSNP5255 | Ca-Kabuli-Chr6 | 14861487 | [T/A] | - | Intergenic | - |
| CWSNP5256 | Ca-Kabuli-Chr6 | 14884604 | [T/G] | - | Intergenic | - |
| CWSNP5257 | Ca-Kabuli-Chr6 | 14983391 | [C/T] | Ca05273 | Synonymous-CDS | BTB/POZ-like |
| CWSNP5258 | Ca-Kabuli-Chr6 | 15051094 | [T/C] | - | DRR | - |
| CWSNP5259 | Ca-Kabuli-Chr6 | 15061717 | [G/A] | Ca05286 | Intron | Protein of unknown function DUF3506 |
| CWSNP5260 | Ca-Kabuli-Chr6 | 15123739 | [C/T] | Ca05293 | Intron | Dynamin central domain |
| CWSNP5261 | Ca-Kabuli-Chr6 | 15294332 | [T/G] | - | Intergenic | - |
| CWSNP5262 | Ca-Kabuli-Chr6 | 15294331 | [C/T] | - | Intergenic | - |
| CWSNP5263 | Ca-Kabuli-Chr6 | 15294329 | [A/C] | - | Intergenic | - |
| CWSNP5264 | Ca-Kabuli-Chr6 | 15301137 | [C/T] | - | DRR | - |

| SNP IDs | Chromosomes | Physical positions (bp) | SNPs | Gene accession IDs | Sequence components of genes | Putative functions |
|---|---|---|---|---|---|---|
| CWSNP5265 | Ca-Kabuli-Chr6 | 15313655 | [G/T] | Ca05316 | Non-Synonymous-CDS | Regulato rof K+ conductance,N-terminal |
| CWSNP5266 | Ca-Kabuli-Chr6 | 15488309 | [G/A] | Ca05332 | Intron | Kinesin , motor domain |
| CWSNP5267 | Ca-Kabuli-Chr6 | 15537908 | [A/C] | Ca05338 | Synonymous-CDS | - |
| CWSNP5268 | Ca-Kabuli-Chr6 | 15543295 | [C/G] | Ca05339 | Non-Synonymous-CDS | Tetratricopeptide repeat |
| CWSNP5269 | Ca-Kabuli-Chr6 | 15543293 | [T/G] | Ca05339 | Non-Synonymous-CDS | Tetratricopeptide repeat |
| CWSNP5270 | Ca-Kabuli-Chr6 | 15543286 | [G/A] | Ca05339 | Non-Synonymous-CDS | Tetratricopeptide repeat |
| CWSNP5271 | Ca-Kabuli-Chr6 | 15589885 | [G/T] | Ca05341 | Intron | Terpenesynthase-like |
| CWSNP5272 | Ca-Kabuli-Chr6 | 15644412 | [A/T] | Ca05343 | Intron | Terpenesynthase-like |
| CWSNP5273 | Ca-Kabuli-Chr6 | 15644796 | [A/T] | Ca05343 | Intron | Terpenesynthase-like |
| CWSNP5274 | Ca-Kabuli-Chr6 | 15861278 | [G/A] | - | Intergenic | - |
| CWSNP5275 | Ca-Kabuli-Chr6 | 16113523 | [T/C] | - | URR | - |
| CWSNP5276 | Ca-Kabuli-Chr6 | 16113522 | [A/T] | - | URR | - |
| CWSNP5277 | Ca-Kabuli-Chr6 | 16113521 | [G/T] | - | URR | - |
| CWSNP5278 | Ca-Kabuli-Chr6 | 16148277 | [A/C] | Ca19676 | Intron | Calmodulin binding protein-like |
| CWSNP5279 | Ca-Kabuli-Chr6 | 16189692 | [C/A] | Ca19671 | Non-Synonymous-CDS | S1/P1nuclease |
| CWSNP5280 | Ca-Kabuli-Chr6 | 16189693 | [G/T] | Ca19671 | Synonymous-CDS | S1/P1nuclease |

| SNP IDs | Chromosomes | Physical positions (bp) | SNPs | Gene accession IDs | Sequence components of genes | Putative functions |
|---|---|---|---|---|---|---|
| CWSNP5281 | Ca-Kabuli-Chr6 | 16196582 | [G/C] | Ca19670 | Synonymous-CDS | Sugar/inositol transporter |
| CWSNP5282 | Ca-Kabuli-Chr6 | 16332377 | [G/A] | Ca19665 | Intron | Protein of unknown function DUF707 |
| CWSNP5283 | Ca-Kabuli-Chr6 | 16460495 | [A/C] | Ca19651 | Synonymous-CDS | - |
| CWSNP5284 | Ca-Kabuli-Chr6 | 16635061 | [C/T] | Ca06293 | Intron | Double-stranded RNA-binding |
| CWSNP5285 | Ca-Kabuli-Chr6 | 16646713 | [T/G] | - | Intergenic | - |
| CWSNP5286 | Ca-Kabuli-Chr6 | 16719918 | [C/A] | Ca06303 | Non-Synonymous-CDS | DNA mismatch repair protein MutS, C-terminaldomain |
| CWSNP5287 | Ca-Kabuli-Chr6 | 16719919 | [T/G] | Ca06303 | Synonymous-CDS | DNA mismatch repair protein MutS, C-terminaldomain |
| CWSNP5288 | Ca-Kabuli-Chr6 | 17096214 | [C/T] | Ca06327 | Intron | Mitochondrial substrate/solute carrier |
| CWSNP5289 | Ca-Kabuli-Chr6 | 17096224 | [C/T] | Ca06327 | Intron | Mitochondrial substrate/solute carrier |
| CWSNP5290 | Ca-Kabuli-Chr6 | 17129574 | [A/G] | - | DRR | - |
| CWSNP5291 | Ca-Kabuli-Chr6 | 17175334 | [C/A] | - | Intergenic | - |
| CWSNP5292 | Ca-Kabuli-Chr6 | 17258886 | [T/G] | Ca06343 | Non-Synonymous-CDS | Homeobox |
| CWSNP5293 | Ca-Kabuli-Chr6 | 17262283 | [C/T] | Ca06343 | Synonymous-CDS | Homeobox |
| CWSNP5294 | Ca-Kabuli-Chr6 | 17263126 | [G/A] | Ca06343 | Synonymous-CDS | Homeobox |
| CWSNP5295 | Ca-Kabuli-Chr6 | 17369613 | [A/G] | Ca06352 | Synonymous-CDS | Transcriptional factor B3 |
| CWSNP5296 | Ca-Kabuli-Chr6 | 17445945 | [T/G] | - | DRR | - |

| SNP IDs | Chromosomes | Physical positions (bp) | SNPs | Gene accession IDs | Sequence components of genes | Putative functions |
|---|---|---|---|---|---|---|
| CWSNP5297 | Ca-Kabuli-Chr6 | 17477741 | [A/G] | Ca06361 | Synonymous-CDS | - |
| CWSNP5298 | Ca-Kabuli-Chr6 | 17478220 | [G/A] | Ca06361 | Synonymous-CDS | - |
| CWSNP5299 | Ca-Kabuli-Chr6 | 17480486 | [G/C] | Ca06361 | Synonymous-CDS | - |
| CWSNP5300 | Ca-Kabuli-Chr6 | 17481288 | [T/C] | - | DRR | - |
| CWSNP5301 | Ca-Kabuli-Chr6 | 17481307 | [C/A] | - | DRR | - |
| CWSNP5302 | Ca-Kabuli-Chr6 | 17481403 | [T/G] | - | Intergenic | - |
| CWSNP5303 | Ca-Kabuli-Chr6 | 17481392 | [A/G] | - | Intergenic | - |
| CWSNP5304 | Ca-Kabuli-Chr6 | 17482626 | [A/G] | - | Intergenic | - |
| CWSNP5305 | Ca-Kabuli-Chr6 | 17482640 | [C/T] | - | Intergenic | - |
| CWSNP5306 | Ca-Kabuli-Chr6 | 17482649 | [A/G] | - | Intergenic | - |
| CWSNP5307 | Ca-Kabuli-Chr6 | 17482718 | [G/A] | - | Intergenic | - |
| CWSNP5308 | Ca-Kabuli-Chr6 | 17482697 | [A/G] | - | Intergenic | - |
| CWSNP5309 | Ca-Kabuli-Chr6 | 17573309 | [A/G] | Ca06374 | Intron | Apoptosis inhibitory5 |
| CWSNP5310 | Ca-Kabuli-Chr6 | 17659067 | [A/C] | - | DRR | - |
| CWSNP5311 | Ca-Kabuli-Chr6 | 17741078 | [G/A] | Ca06393 | Synonymous-CDS | WW/Rsp5/WWP |
| CWSNP5312 | Ca-Kabuli-Chr6 | 18027391 | [G/A] | Ca06422 | Intron | Myb,DNA-binding |

| SNP IDs | Chromosomes | Physical positions (bp) | SNPs | Gene accession IDs | Sequence components of genes | Putative functions |
|---|---|---|---|---|---|---|
| CWSNP5313 | Ca-Kabuli-Chr6 | 18094691 | [C/A] | - | Intergenic | - |
| CWSNP5314 | Ca-Kabuli-Chr6 | 18125613 | [C/A] | - | DRR | - |
| CWSNP5315 | Ca-Kabuli-Chr6 | 18389704 | [T/G] | Ca06455 | Intron | Glycosyltransferase,family10 |
| CWSNP5316 | Ca-Kabuli-Chr6 | 18389661 | [A/C] | Ca06455 | Intron | Glycosyltransferase,family10 |
| CWSNP5317 | Ca-Kabuli-Chr6 | 18444725 | [T/C] | - | DRR | - |
| CWSNP5318 | Ca-Kabuli-Chr6 | 18987220 | [T/G] | - | DRR | - |
| CWSNP5319 | Ca-Kabuli-Chr6 | 19125254 | [G/A] | - | DRR | - |
| CWSNP5320 | Ca-Kabuli-Chr6 | 19450845 | [T/A] | Ca06550 | Intron | ATPase,AAA+type,core |
| CWSNP5321 | Ca-Kabuli-Chr6 | 19551459 | [G/A] | Ca06563 | Intron | Asparaginesynthase |
| CWSNP5322 | Ca-Kabuli-Chr6 | 19551504 | [G/C] | Ca06563 | Intron | Asparaginesynthase |
| CWSNP5323 | Ca-Kabuli-Chr6 | 20505641 | [A/T] | Ca16381 | Non-Synonymous-CDS | Cullin,N-terminal |
| CWSNP5324 | Ca-Kabuli-Chr6 | 20766377 | [C/A] | Ca16355 | Non-Synonymous-CDS | Helix-loop-helix DNA-binding domain |
| CWSNP5325 | Ca-Kabuli-Chr6 | 20830353 | [T/G] | - | Intergenic | - |
| CWSNP5326 | Ca-Kabuli-Chr6 | 20917713 | [A/C] | - | Intergenic | - |
| CWSNP5327 | Ca-Kabuli-Chr6 | 21025677 | [T/A] | - | Intergenic | - |
| CWSNP5328 | Ca-Kabuli-Chr6 | 21025742 | [T/G] | - | Intergenic | - |

| SNP IDs | Chromosomes | Physical positions (bp) | SNPs | Gene accession IDs | Sequence components of genes | Putative functions |
|---|---|---|---|---|---|---|
| CWSNP5329 | Ca-Kabuli-Chr6 | 21025706 | [C/T] | - | Intergenic | - |
| CWSNP5330 | Ca-Kabuli-Chr6 | 21025673 | [C/T] | - | Intergenic | - |
| CWSNP5331 | Ca-Kabuli-Chr6 | 21025894 | [G/T] | - | Intergenic | - |
| CWSNP5332 | Ca-Kabuli-Chr6 | 21135764 | [C/T] | Ca19889 | Non-Synonymous-CDS | - |
| CWSNP5333 | Ca-Kabuli-Chr6 | 21139038 | [C/G] | - | Intergenic | - |
| CWSNP5334 | Ca-Kabuli-Chr6 | 21165999 | [G/T] | - | Intergenic | - |
| CWSNP5335 | Ca-Kabuli-Chr6 | 21238707 | [G/T] | - | DRR | - |
| CWSNP5336 | Ca-Kabuli-Chr6 | 21276479 | [G/T] | - | Intergenic | - |
| CWSNP5337 | Ca-Kabuli-Chr6 | 21323438 | [T/C] | Ca19873 | Synonymous-CDS | Phosphoenol pyruvatecarboxylase,active site |
| CWSNP5338 | Ca-Kabuli-Chr6 | 21385915 | [C/A] | - | URR | - |
| CWSNP5339 | Ca-Kabuli-Chr6 | 21478820 | [G/T] | - | URR | - |
| CWSNP5340 | Ca-Kabuli-Chr6 | 21478816 | [C/T] | - | URR | - |
| CWSNP5341 | Ca-Kabuli-Chr6 | 21483425 | [T/G] | - | Intergenic | - |
| CWSNP5342 | Ca-Kabuli-Chr6 | 21483429 | [C/T] | - | Intergenic | - |
| CWSNP5343 | Ca-Kabuli-Chr6 | 21483494 | [T/G] | - | Intergenic | - |
| CWSNP5344 | Ca-Kabuli-Chr6 | 21718674 | [G/T] | Ca11268 | Non-Synonymous-CDS | Myb,DNA-binding |

| SNP IDs | Chromosomes | Physical positions (bp) | SNPs | Gene accession IDs | Sequence components of genes | Putative functions |
|---|---|---|---|---|---|---|
| CWSNP5345 | Ca-Kabuli-Chr6 | 21763468 | [G/A] | - | Intergenic | - |
| CWSNP5346 | Ca-Kabuli-Chr6 | 21940793 | [T/C] | - | DRR | - |
| CWSNP5347 | Ca-Kabuli-Chr6 | 22038857 | [A/G] | - | Intergenic | - |
| CWSNP5348 | Ca-Kabuli-Chr6 | 22137464 | [G/A] | - | Intergenic | - |
| CWSNP5349 | Ca-Kabuli-Chr6 | 22192503 | [G/A] | Ca11219 | Synonymous-CDS | Exostosin-like |
| CWSNP5350 | Ca-Kabuli-Chr6 | 22192498 | [A/C] | Ca11219 | Non-Synonymous-CDS | Exostosin-like |
| CWSNP5351 | Ca-Kabuli-Chr6 | 22194387 | [C/A] | Ca11219 | Non-Synonymous-CDS | Exostosin-like |
| CWSNP5352 | Ca-Kabuli-Chr6 | 22208125 | [G/A] | - | URR | - |
| CWSNP5353 | Ca-Kabuli-Chr6 | 22208093 | [G/A] | - | URR | - |
| CWSNP5354 | Ca-Kabuli-Chr6 | 22211823 | [T/C] | - | DRR | - |
| CWSNP5355 | Ca-Kabuli-Chr6 | 22217158 | [A/G] | - | Intergenic | - |
| CWSNP5356 | Ca-Kabuli-Chr6 | 22217157 | [T/G] | - | Intergenic | - |
| CWSNP5357 | Ca-Kabuli-Chr6 | 22225288 | [T/G] | - | DRR | - |
| CWSNP5358 | Ca-Kabuli-Chr6 | 22252149 | [G/A] | - | Intergenic | - |
| CWSNP5359 | Ca-Kabuli-Chr6 | 22263388 | [A/G] | - | Intergenic | - |
| CWSNP5360 | Ca-Kabuli-Chr6 | 22352976 | [T/C] | - | URR | - |

| SNP IDs | Chromosomes | Physical positions (bp) | SNPs | Gene accession IDs | Sequence components of genes | Putative functions |
|---|---|---|---|---|---|---|
| CWSNP5361 | Ca-Kabuli-Chr6 | 22370242 | [C/T] | - | DRR | - |
| CWSNP5362 | Ca-Kabuli-Chr6 | 22446610 | [G/A] | Ca11195 | Non-Synonymous-CDS | PeptidaseC48,SUMO/Sentrin/Ubl1 |
| CWSNP5363 | Ca-Kabuli-Chr6 | 22446649 | [C/T] | Ca11195 | Non-Synonymous-CDS | PeptidaseC48,SUMO/Sentrin/Ubl1 |
| CWSNP5364 | Ca-Kabuli-Chr6 | 22482366 | [A/C] | Ca11192 | Intron | Nitrite/sulphite reductase,hemoprotein beta-component,ferrodoxin-like |
| CWSNP5365 | Ca-Kabuli-Chr6 | 23039165 | [T/C] | - | Intergenic | - |
| CWSNP5366 | Ca-Kabuli-Chr6 | 23039147 | [G/A] | - | Intergenic | - |
| CWSNP5367 | Ca-Kabuli-Chr6 | 23039145 | [A/G] | - | Intergenic | - |
| CWSNP5368 | Ca-Kabuli-Chr6 | 23039125 | [G/A] | - | Intergenic | - |
| CWSNP5369 | Ca-Kabuli-Chr6 | 23039097 | [C/T] | - | Intergenic | - |
| CWSNP5370 | Ca-Kabuli-Chr6 | 23766943 | [C/T] | Ca11079 | Intron | Tetratricopeptide repeat-containing |
| CWSNP5371 | Ca-Kabuli-Chr6 | 23973410 | [G/A] | Ca11064 | Intron | RNA recognition motif domain |
| CWSNP5372 | Ca-Kabuli-Chr6 | 24007185 | [C/T] | - | DRR | - |
| CWSNP5373 | Ca-Kabuli-Chr6 | 24403316 | [G/T] | Ca11029 | Non-Synonymous-CDS | PeptidaseS8/S53,subtilisin/kexin/sedolisin |
| CWSNP5374 | Ca-Kabuli-Chr6 | 24516532 | [C/T] | Ca27822 | Synonymous-CDS | - |
| CWSNP5375 | Ca-Kabuli-Chr6 | 24661895 | [G/A] | - | DRR | - |
| CWSNP5376 | Ca-Kabuli-Chr6 | 24661916 | [C/T] | - | DRR | - |

| SNP IDs | Chromosomes | Physical positions (bp) | SNPs | Gene accession IDs | Sequence components of genes | Putative functions |
|---|---|---|---|---|---|---|
| CWSNP5377 | Ca-Kabuli-Chr6 | 24661937 | [C/T] | - | DRR | - |
| CWSNP5378 | Ca-Kabuli-Chr6 | 24661945 | [T/A] | - | DRR | - |
| CWSNP5379 | Ca-Kabuli-Chr6 | 24661986 | [G/A] | - | DRR | - |
| CWSNP5380 | Ca-Kabuli-Chr6 | 25575097 | [T/C] | Ca24008 | Synonymous-CDS | - |
| CWSNP5381 | Ca-Kabuli-Chr6 | 25575078 | [C/T] | Ca24008 | Non-Synonymous-CDS | - |
| CWSNP5382 | Ca-Kabuli-Chr6 | 25575062 | [G/A] | Ca24008 | Non-Synonymous-CDS | - |
| CWSNP5383 | Ca-Kabuli-Chr6 | 25575045 | [C/A] | Ca24008 | Non-Synonymous-CDS | - |
| CWSNP5384 | Ca-Kabuli-Chr6 | 25604942 | [C/A] | - | Intergenic | - |
| CWSNP5385 | Ca-Kabuli-Chr6 | 25604936 | [C/T] | - | Intergenic | - |
| CWSNP5386 | Ca-Kabuli-Chr6 | 25604931 | [C/A] | - | Intergenic | - |
| CWSNP5387 | Ca-Kabuli-Chr6 | 25604922 | [A/G] | - | Intergenic | - |
| CWSNP5388 | Ca-Kabuli-Chr6 | 25604954 | [C/T] | - | Intergenic | - |
| CWSNP5389 | Ca-Kabuli-Chr6 | 25604962 | [G/A] | - | Intergenic | - |
| CWSNP5390 | Ca-Kabuli-Chr6 | 25605006 | [C/A] | - | Intergenic | - |
| CWSNP5391 | Ca-Kabuli-Chr6 | 25605023 | [C/A] | - | Intergenic | - |
| CWSNP5392 | Ca-Kabuli-Chr6 | 25605022 | [T/C] | - | Intergenic | - |

| SNP IDs | Chromosomes | Physical positions (bp) | SNPs | Gene accession IDs | Sequence components of genes | Putative functions |
|---|---|---|---|---|---|---|
| CWSNP5393 | Ca-Kabuli-Chr6 | 25850736 | [C/T] | Ca24011 | Non-Synonymous-CDS | - |
| CWSNP5394 | Ca-Kabuli-Chr6 | 25850759 | [C/A] | Ca24011 | Non-Synonymous-CDS | - |
| CWSNP5395 | Ca-Kabuli-Chr6 | 25850783 | [G/A] | Ca24011 | Non-Synonymous-CDS | - |
| CWSNP5396 | Ca-Kabuli-Chr6 | 25850816 | [C/A] | Ca24011 | Non-Synonymous-CDS | - |
| CWSNP5397 | Ca-Kabuli-Chr6 | 25850813 | [C/T] | Ca24011 | Non-Synonymous-CDS | - |
| CWSNP5398 | Ca-Kabuli-Chr6 | 25850866 | [C/G] | Ca24011 | Synonymous-CDS | - |
| CWSNP5399 | Ca-Kabuli-Chr6 | 25850827 | [C/T] | Ca24011 | Synonymous-CDS | - |
| CWSNP5400 | Ca-Kabuli-Chr6 | 26349434 | [G/A] | - | DRR | - |
| CWSNP5401 | Ca-Kabuli-Chr6 | 26349451 | [T/C] | - | DRR | - |
| CWSNP5402 | Ca-Kabuli-Chr6 | 26353512 | [C/T] | - | DRR | - |
| CWSNP5403 | Ca-Kabuli-Chr6 | 26353740 | [G/C] | - | DRR | - |
| CWSNP5404 | Ca-Kabuli-Chr6 | 26357149 | [C/G] | - | URR | - |
| CWSNP5405 | Ca-Kabuli-Chr6 | 26360367 | [C/A] | Ca16679 | Synonymous-CDS | Oxysterol-binding protein |
| CWSNP5406 | Ca-Kabuli-Chr6 | 26385432 | [T/C] | - | Intergenic | - |
| CWSNP5407 | Ca-Kabuli-Chr6 | 26426239 | [C/A] | - | DRR | - |
| CWSNP5408 | Ca-Kabuli-Chr6 | 26426252 | [A/G] | - | DRR | - |

| SNP IDs | Chromosomes | Physical positions (bp) | SNPs | Gene accession IDs | Sequence components of genes | Putative functions |
|---|---|---|---|---|---|---|
| CWSNP5409 | Ca-Kabuli-Chr6 | 26426283 | [G/A] | - | DRR | - |
| CWSNP5410 | Ca-Kabuli-Chr6 | 26527161 | [C/G] | Ca16688 | Intron | Zinc finger,RING-type |
| CWSNP5411 | Ca-Kabuli-Chr6 | 26549168 | [T/G] | Ca16689 | Intron | Armadillo |
| CWSNP5412 | Ca-Kabuli-Chr6 | 26567360 | [A/G] | Ca16690 | Synonymous-CDS | Zinc finger,Dof-type |
| CWSNP5413 | Ca-Kabuli-Chr6 | 26698265 | [A/C] | Ca16698 | Synonymous-CDS | Cellular retinaldehyde-binding/triple function,C-terminal |
| CWSNP5414 | Ca-Kabuli-Chr6 | 26769087 | [C/A] | Ca16707 | Non-Synonymous-CDS | Ras GTPase |
| CWSNP5415 | Ca-Kabuli-Chr6 | 27310342 | [A/T] | Ca14618 | Intron | Pathogenesis-related transcriptional factor/ERF, DNA-binding |
| CWSNP5416 | Ca-Kabuli-Chr6 | 27310341 | [G/T] | Ca14618 | Intron | Pathogenesis-related transcriptional factor/ERF, DNA-binding |
| CWSNP5417 | Ca-Kabuli-Chr6 | 27310336 | [A/T] | Ca14618 | Intron | Pathogenesis-related transcriptional factor/ERF, DNA-binding |
| CWSNP5418 | Ca-Kabuli-Chr6 | 27310333 | [C/T] | Ca14618 | Intron | Pathogenesis-related transcriptional factor/ERF, DNA-binding |
| CWSNP5419 | Ca-Kabuli-Chr6 | 27310328 | [T/A] | Ca14618 | Intron | Pathogenesis-related transcriptional factor/ERF, DNA-binding |
| CWSNP5420 | Ca-Kabuli-Chr6 | 27310327 | [A/T] | Ca14618 | Intron | Pathogenesis-related transcriptional factor/ERF, DNA-binding |
| CWSNP5421 | Ca-Kabuli-Chr6 | 27310325 | [G/T] | Ca14618 | Intron | Pathogenesis-related transcriptional factor/ERF, DNA-binding |
| CWSNP5422 | Ca-Kabuli-Chr6 | 27310315 | [C/T] | Ca14618 | Intron | Pathogenesis-related transcriptional factor/ERF, DNA-binding |
| CWSNP5423 | Ca-Kabuli-Chr6 | 27331165 | [T/C] | Ca14617 | Non-Synonymous-CDS | RNA recognition motif domain |
| CWSNP5424 | Ca-Kabuli-Chr6 | 27383746 | [A/T] | - | DRR | - |

| SNP IDs | Chromosomes | Physical positions (bp) | SNPs | Gene accession IDs | Sequence components of genes | Putative functions |
|---|---|---|---|---|---|---|
| CWSNP5425 | Ca-Kabuli-Chr6 | 27383745 | [G/T] | - | DRR | - |
| CWSNP5426 | Ca-Kabuli-Chr6 | 27383744 | [G/T] | - | DRR | - |
| CWSNP5427 | Ca-Kabuli-Chr6 | 27515293 | [G/A] | Ca14606 | Synonymous-CDS | Tetraspanin,subgroup |
| CWSNP5428 | Ca-Kabuli-Chr6 | 27655605 | [A/G] | Ca14592 | Non-Synonymous-CDS | CytochromeB561-related |
| CWSNP5429 | Ca-Kabuli-Chr6 | 27710531 | [T/G] | - | Intergenic | - |
| CWSNP5430 | Ca-Kabuli-Chr6 | 27986719 | [A/G] | Ca14567 | Non-Synonymous-CDS | Ribosomal proteinL11 |
| CWSNP5431 | Ca-Kabuli-Chr6 | 28021899 | [C/T] | Ca14563 | Non-Synonymous-CDS | DNA-directed DNA polymerase,familyB,exonuclease domain |
| CWSNP5432 | Ca-Kabuli-Chr6 | 28044830 | [C/A] | Ca14563 | Non-Synonymous-CDS | DNA-directed DNA polymerase,familyB,exonuclease domain |
| CWSNP5433 | Ca-Kabuli-Chr6 | 28052463 | [T/G] | Ca14562 | Synonymous-CDS | Vacuolar fusion protein MON1 |
| CWSNP5434 | Ca-Kabuli-Chr6 | 28325392 | [C/T] | Ca25968 | Non-Synonymous-CDS | RibonucleaseT2 |
| CWSNP5435 | Ca-Kabuli-Chr6 | 28372130 | [C/T] | - | Intergenic | - |
| CWSNP5436 | Ca-Kabuli-Chr6 | 28728293 | [A/C] | Ca17478 | Non-Synonymous-CDS | ChaperoninCpn60 |
| CWSNP5437 | Ca-Kabuli-Chr6 | 28757208 | [C/A] | Ca17477 | Intron | - |
| CWSNP5438 | Ca-Kabuli-Chr6 | 28757204 | [G/A] | Ca17477 | Intron | - |
| CWSNP5439 | Ca-Kabuli-Chr6 | 28757139 | [C/A] | Ca17477 | Intron | - |
| CWSNP5440 | Ca-Kabuli-Chr6 | 28887188 | [G/T] | - | Intergenic | - |

| SNP IDs | Chromosomes | Physical positions (bp) | SNPs | Gene accession IDs | Sequence components of genes | Putative functions |
|---|---|---|---|---|---|---|
| CWSNP5441 | Ca-Kabuli-Chr6 | 28947529 | [A/C] | Ca17471 | Non-Synonymous-CDS | Glyoxalase/bleomycin resistance protein/dioxygenase |
| CWSNP5442 | Ca-Kabuli-Chr6 | 29085558 | [T/C] | - | Intergenic | - |
| CWSNP5443 | Ca-Kabuli-Chr6 | 29163667 | [C/A] | - | DRR | - |
| CWSNP5444 | Ca-Kabuli-Chr6 | 29163642 | [G/A] | - | DRR | - |
| CWSNP5445 | Ca-Kabuli-Chr6 | 29163640 | [A/C] | - | DRR | - |
| CWSNP5446 | Ca-Kabuli-Chr6 | 29205260 | [A/G] | Ca17457 | Intron | DNAligase,ATP-dependent,N-terminal |
| CWSNP5447 | Ca-Kabuli-Chr6 | 29205940 | [A/G] | Ca17457 | Non-Synonymous-CDS | DNAligase,ATP-dependent,N-terminal |
| CWSNP5448 | Ca-Kabuli-Chr6 | 29495856 | [C/T] | Ca17443 | Synonymous-CDS | - |
| CWSNP5449 | Ca-Kabuli-Chr6 | 30257284 | [A/C] | Ca16442 | Intron | PeptidaseC48,SUMO/Sentrin/Ubl1 |
| CWSNP5450 | Ca-Kabuli-Chr6 | 30676031 | [G/T] | Ca16465 | Synonymous-CDS | Translation elongation facto rEFG/EF2,C-terminal |
| CWSNP5451 | Ca-Kabuli-Chr6 | 30676248 | [C/T] | Ca16465 | Synonymous-CDS | Translation elongation facto rEFG/EF2,C-terminal |
| CWSNP5452 | Ca-Kabuli-Chr6 | 30926084 | [G/A] | Ca16485 | Non-Synonymous-CDS | - |
| CWSNP5453 | Ca-Kabuli-Chr6 | 30927221 | [C/G] | Ca16485 | Non-Synonymous-CDS | - |
| CWSNP5454 | Ca-Kabuli-Chr6 | 31048949 | [T/C] | Ca16497 | Synonymous-CDS | Late embryogenesis abundant protein, group2 |
| CWSNP5455 | Ca-Kabuli-Chr6 | 31057806 | [C/T] | - | URR | - |
| CWSNP5456 | Ca-Kabuli-Chr6 | 31324962 | [A/C] | - | Intergenic | - |

| SNP IDs | Chromosomes | Physical positions (bp) | SNPs | Gene accession IDs | Sequence components of genes | Putative functions |
|---|---|---|---|---|---|---|
| CWSNP5457 | Ca-Kabuli-Chr6 | 31324976 | [A/G] | - | Intergenic | - |
| CWSNP5458 | Ca-Kabuli-Chr6 | 31895264 | [C/T] | Ca15200 | Intron | Metallo-dependent phosphatase |
| CWSNP5459 | Ca-Kabuli-Chr6 | 31900781 | [T/G] | Ca15200 | Intron | Metallo-dependent phosphatase |
| CWSNP5460 | Ca-Kabuli-Chr6 | 31900785 | [T/G] | Ca15200 | Intron | Metallo-dependent phosphatase |
| CWSNP5461 | Ca-Kabuli-Chr6 | 31900790 | [A/C] | Ca15200 | Intron | Metallo-dependent phosphatase |
| CWSNP5462 | Ca-Kabuli-Chr6 | 31992907 | [A/G] | Ca15204 | Intron | - |
| CWSNP5463 | Ca-Kabuli-Chr6 | 32140428 | [C/A] | Ca15214 | Non-Synonymous-CDS | BRCT |
| CWSNP5464 | Ca-Kabuli-Chr6 | 32140429 | [G/T] | Ca15214 | Synonymous-CDS | BRCT |
| CWSNP5465 | Ca-Kabuli-Chr6 | 32262807 | [T/C] | - | DRR | - |
| CWSNP5466 | Ca-Kabuli-Chr6 | 32262855 | [C/T] | - | DRR | - |
| CWSNP5467 | Ca-Kabuli-Chr6 | 32328468 | [T/C] | Ca15224 | Non-Synonymous-CDS | DNA-binding WRKY |
| CWSNP5468 | Ca-Kabuli-Chr6 | 32717450 | [G/A] | - | DRR | - |
| CWSNP5469 | Ca-Kabuli-Chr6 | 32717665 | [C/A] | - | DRR | - |
| CWSNP5470 | Ca-Kabuli-Chr6 | 32825567 | [C/A] | - | Intergenic | - |
| CWSNP5471 | Ca-Kabuli-Chr6 | 32825561 | [C/T] | - | Intergenic | - |
| CWSNP5472 | Ca-Kabuli-Chr6 | 32825547 | [A/G] | - | Intergenic | - |

| SNP IDs | Chromosomes | Physical positions (bp) | SNPs | Gene accession IDs | Sequence components of genes | Putative functions |
|---|---|---|---|---|---|---|
| CWSNP5473 | Ca-Kabuli-Chr6 | 32825587 | [G/A] | - | Intergenic | - |
| CWSNP5474 | Ca-Kabuli-Chr6 | 32825619 | [T/C] | - | Intergenic | - |
| CWSNP5475 | Ca-Kabuli-Chr6 | 32825631 | [C/A] | - | Intergenic | - |
| CWSNP5476 | Ca-Kabuli-Chr6 | 32914927 | [C/G] | Ca22229 | Intron | PeptidaseM48 |
| CWSNP5477 | Ca-Kabuli-Chr6 | 33502994 | [C/A] | - | Intergenic | - |
| CWSNP5478 | Ca-Kabuli-Chr6 | 33503007 | [T/C] | - | Intergenic | - |
| CWSNP5479 | Ca-Kabuli-Chr6 | 33503032 | [A/C] | - | Intergenic | - |
| CWSNP5480 | Ca-Kabuli-Chr6 | 33503051 | [G/C] | - | Intergenic | - |
| CWSNP5481 | Ca-Kabuli-Chr6 | 33503063 | [C/G] | - | Intergenic | - |
| CWSNP5482 | Ca-Kabuli-Chr6 | 33503065 | [C/A] | - | Intergenic | - |
| CWSNP5483 | Ca-Kabuli-Chr6 | 33503071 | [A/G] | - | Intergenic | - |
| CWSNP5484 | Ca-Kabuli-Chr6 | 33598926 | [G/A] | Ca22069 | Intron | - |
| CWSNP5485 | Ca-Kabuli-Chr6 | 33704792 | [C/T] | - | Intergenic | - |
| CWSNP5486 | Ca-Kabuli-Chr6 | 33704807 | [C/A] | - | Intergenic | - |
| CWSNP5487 | Ca-Kabuli-Chr6 | 33733259 | [A/G] | Ca22063 | Synonymous-CDS | Zinc finger,RING-type |
| CWSNP5488 | Ca-Kabuli-Chr6 | 34407309 | [T/C] | - | Intergenic | - |

| SNP IDs | Chromosomes | Physical positions (bp) | SNPs | Gene accession IDs | Sequence components of genes | Putative functions |
|---|---|---|---|---|---|---|
| CWSNP5489 | Ca-Kabuli-Chr6 | 34481266 | [A/G] | Ca15818 | Intron | - |
| CWSNP5490 | Ca-Kabuli-Chr6 | 34481256 | [C/T] | Ca15818 | Intron | - |
| CWSNP5491 | Ca-Kabuli-Chr6 | 34596792 | [A/G] | - | Intergenic | - |
| CWSNP5492 | Ca-Kabuli-Chr6 | 34744433 | [A/G] | - | Intergenic | - |
| CWSNP5493 | Ca-Kabuli-Chr6 | 34744438 | [T/G] | - | Intergenic | - |
| CWSNP5494 | Ca-Kabuli-Chr6 | 34744681 | [T/G] | - | Intergenic | - |
| CWSNP5495 | Ca-Kabuli-Chr6 | 34744665 | [G/A] | - | Intergenic | - |
| CWSNP5496 | Ca-Kabuli-Chr6 | 35129588 | [G/A] | Ca15788 | Synonymous-CDS | Zinc finger,PHD-type |
| CWSNP5497 | Ca-Kabuli-Chr6 | 35596064 | [T/C] | - | Intergenic | - |
| CWSNP5498 | Ca-Kabuli-Chr6 | 35596070 | [G/A] | - | Intergenic | - |
| CWSNP5499 | Ca-Kabuli-Chr6 | 35596161 | [C/T] | - | Intergenic | - |
| CWSNP5500 | Ca-Kabuli-Chr6 | 35596184 | [A/G] | - | Intergenic | - |
| CWSNP5501 | Ca-Kabuli-Chr6 | 35596195 | [G/C] | - | Intergenic | - |
| CWSNP5502 | Ca-Kabuli-Chr6 | 35596197 | [G/A] | - | Intergenic | - |
| CWSNP5503 | Ca-Kabuli-Chr6 | 35596207 | [G/T] | - | Intergenic | - |
| CWSNP5504 | Ca-Kabuli-Chr6 | 35596218 | [T/C] | - | Intergenic | - |

| SNP IDs | Chromosomes | Physical positions (bp) | SNPs | Gene accession IDs | Sequence components of genes | Putative functions |
|---|---|---|---|---|---|---|
| CWSNP5505 | Ca-Kabuli-Chr6 | 35596220 | [G/A] | - | Intergenic | - |
| CWSNP5506 | Ca-Kabuli-Chr6 | 35596230 | [T/A] | - | Intergenic | - |
| CWSNP5507 | Ca-Kabuli-Chr6 | 35962309 | [C/T] | - | Intergenic | - |
| CWSNP5508 | Ca-Kabuli-Chr6 | 35962320 | [T/C] | - | Intergenic | - |
| CWSNP5509 | Ca-Kabuli-Chr6 | 35962360 | [T/C] | - | Intergenic | - |
| CWSNP5510 | Ca-Kabuli-Chr6 | 35962364 | [T/G] | - | Intergenic | - |
| CWSNP5511 | Ca-Kabuli-Chr6 | 35962365 | [T/C] | - | Intergenic | - |
| CWSNP5512 | Ca-Kabuli-Chr6 | 35962366 | [G/A] | - | Intergenic | - |
| CWSNP5513 | Ca-Kabuli-Chr6 | 36574836 | [A/G] | - | Intergenic | - |
| CWSNP5514 | Ca-Kabuli-Chr6 | 36790467 | [G/A] | Ca19812 | Non-Synonymous-CDS | - |
| CWSNP5515 | Ca-Kabuli-Chr6 | 36790458 | [G/A] | Ca19812 | Non-Synonymous-CDS | - |
| CWSNP5516 | Ca-Kabuli-Chr6 | 36790428 | [G/A] | Ca19812 | Non-Synonymous-CDS | - |
| CWSNP5517 | Ca-Kabuli-Chr6 | 36790455 | [C/T] | Ca19812 | Non-Synonymous-CDS | - |
| CWSNP5518 | Ca-Kabuli-Chr6 | 37139425 | [C/T] | Ca19803 | Synonymous-CDS | PeptidaseC48,SUMO/Sentrin/Ubl1 |
| CWSNP5519 | Ca-Kabuli-Chr6 | 37139466 | [G/C] | Ca19803 | Synonymous-CDS | PeptidaseC48,SUMO/Sentrin/Ubl1 |
| CWSNP5520 | Ca-Kabuli-Chr6 | 37139484 | [T/C] | Ca19803 | Non-Synonymous-CDS | PeptidaseC48,SUMO/Sentrin/Ubl1 |

| SNP IDs | Chromosomes | Physical positions (bp) | SNPs | Gene accession IDs | Sequence components of genes | Putative functions |
|---|---|---|---|---|---|---|
| CWSNP5521 | Ca-Kabuli-Chr6 | 37139514 | [C/T] | Ca19803 | Non-Synonymous-CDS | PeptidaseC48,SUMO/Sentrin/Ubl1 |
| CWSNP5522 | Ca-Kabuli-Chr6 | 37139499 | [C/G] | Ca19803 | Non-Synonymous-CDS | PeptidaseC48,SUMO/Sentrin/Ubl1 |
| CWSNP5523 | Ca-Kabuli-Chr6 | 37139610 | [T/G] | Ca19803 | Non-Synonymous-CDS | PeptidaseC48,SUMO/Sentrin/Ubl1 |
| CWSNP5524 | Ca-Kabuli-Chr6 | 37139604 | [G/T] | Ca19803 | Non-Synonymous-CDS | PeptidaseC48,SUMO/Sentrin/Ubl1 |
| CWSNP5525 | Ca-Kabuli-Chr6 | 37476426 | [C/T] | - | Intergenic | - |
| CWSNP5526 | Ca-Kabuli-Chr6 | 37476516 | [G/T] | - | Intergenic | - |
| CWSNP5527 | Ca-Kabuli-Chr6 | 37476465 | [G/A] | - | Intergenic | - |
| CWSNP5528 | Ca-Kabuli-Chr6 | 37484467 | [C/T] | - | Intergenic | - |
| CWSNP5529 | Ca-Kabuli-Chr6 | 37484468 | [G/T] | - | Intergenic | - |
| CWSNP5530 | Ca-Kabuli-Chr6 | 37484470 | [C/A] | - | Intergenic | - |
| CWSNP5531 | Ca-Kabuli-Chr6 | 37484489 | [C/G] | - | Intergenic | - |
| CWSNP5532 | Ca-Kabuli-Chr6 | 37484513 | [T/C] | - | Intergenic | - |
| CWSNP5533 | Ca-Kabuli-Chr6 | 38816345 | [C/T] | Ca15898 | Synonymous-CDS | Transcription factor GRAS |
| CWSNP5534 | Ca-Kabuli-Chr6 | 38838320 | [A/G] | Ca15899 | Non-Synonymous-CDS | Calcium-binding EF-hand |
| CWSNP5535 | Ca-Kabuli-Chr6 | 38840279 | [G/A] | - | Intergenic | - |
| CWSNP5536 | Ca-Kabuli-Chr6 | 38840273 | [G/A] | - | Intergenic | - |

| SNP IDs | Chromosomes | Physical positions (bp) | SNPs | Gene accession IDs | Sequence components of genes | Putative functions |
|---|---|---|---|---|---|---|
| CWSNP5537 | Ca-Kabuli-Chr6 | 38881909 | [C/A] | - | Intergenic | - |
| CWSNP5538 | Ca-Kabuli-Chr6 | 38886735 | [C/A] | - | Intergenic | - |
| CWSNP5539 | Ca-Kabuli-Chr6 | 38994879 | [A/G] | Ca15903 | Non-Synonymous-CDS | - |
| CWSNP5540 | Ca-Kabuli-Chr6 | 38994925 | [G/T] | Ca15903 | Non-Synonymous-CDS | - |
| CWSNP5541 | Ca-Kabuli-Chr6 | 38994954 | [A/G] | Ca15903 | Non-Synonymous-CDS | - |
| CWSNP5542 | Ca-Kabuli-Chr6 | 38994962 | [G/A] | Ca15903 | Synonymous-CDS | - |
| CWSNP5543 | Ca-Kabuli-Chr6 | 39107187 | [C/T] | - | Intergenic | - |
| CWSNP5544 | Ca-Kabuli-Chr6 | 39317016 | [T/G] | Ca15914 | Intron | PAP/25A-associated |
| CWSNP5545 | Ca-Kabuli-Chr6 | 39380625 | [A/C] | Ca15918 | Intron | Resolvase,holliday junction-type,YqgF-like |
| CWSNP5546 | Ca-Kabuli-Chr6 | 39908314 | [T/G] | - | DRR | - |
| CWSNP5547 | Ca-Kabuli-Chr6 | 40047823 | [A/G] | Ca15938 | Non-Synonymous-CDS | Chromodomain |
| CWSNP5548 | Ca-Kabuli-Chr6 | 40128644 | [T/C] | - | Intergenic | - |
| CWSNP5549 | Ca-Kabuli-Chr6 | 40468356 | [T/A] | Ca21602 | Intron | - |
| CWSNP5550 | Ca-Kabuli-Chr6 | 40477285 | [G/A] | Ca21600 | Non-Synonymous-CDS | Nitroreductase-like |
| CWSNP5551 | Ca-Kabuli-Chr6 | 40657695 | [A/C] | Ca21596 | Synonymous-CDS | PeptidaseM22,glycoprotease |
| CWSNP5552 | Ca-Kabuli-Chr6 | 40735186 | [C/A] | - | Intergenic | - |

| SNP IDs | Chromosomes | Physical positions (bp) | SNPs | Gene accession IDs | Sequence components of genes | Putative functions |
|---|---|---|---|---|---|---|
| CWSNP5553 | Ca-Kabuli-Chr6 | 40767566 | [A/G] | - | Intergenic | - |
| CWSNP5554 | Ca-Kabuli-Chr6 | 40821354 | [C/T] | Ca21586 | Intron | Transketolase,N-terminal |
| CWSNP5555 | Ca-Kabuli-Chr6 | 40935441 | [A/T] | Ca21581 | Intron | Exo cystcomplex component Sec6 |
| CWSNP5556 | Ca-Kabuli-Chr6 | 41121330 | [G/A] | Ca23143 | Non-Synonymous-CDS | - |
| CWSNP5557 | Ca-Kabuli-Chr6 | 41682983 | [T/C] | - | Intergenic | - |
| CWSNP5558 | Ca-Kabuli-Chr6 | 41682966 | [C/T] | - | Intergenic | - |
| CWSNP5559 | Ca-Kabuli-Chr6 | 41682943 | [T/C] | - | Intergenic | - |
| CWSNP5560 | Ca-Kabuli-Chr6 | 41697812 | [T/G] | Ca21940 | Intron | RibonucleaseII/R |
| CWSNP5561 | Ca-Kabuli-Chr6 | 41708309 | [G/A] | - | Intergenic | - |
| CWSNP5562 | Ca-Kabuli-Chr6 | 41708306 | [G/A] | - | Intergenic | - |
| CWSNP5563 | Ca-Kabuli-Chr6 | 41708523 | [T/G] | - | Intergenic | - |
| CWSNP5564 | Ca-Kabuli-Chr6 | 41708474 | [G/A] | - | Intergenic | - |
| CWSNP5565 | Ca-Kabuli-Chr6 | 41708530 | [C/A] | - | Intergenic | - |
| CWSNP5566 | Ca-Kabuli-Chr6 | 41907459 | [C/G] | - | Intergenic | - |
| CWSNP5567 | Ca-Kabuli-Chr6 | 42004851 | [A/G] | Ca21951 | Non-Synonymous-CDS | TGS |
| CWSNP5568 | Ca-Kabuli-Chr6 | 42004829 | [T/G] | Ca21951 | Synonymous-CDS | TGS |

| SNP IDs | Chromosomes | Physical positions (bp) | SNPs | Gene accession IDs | Sequence components of genes | Putative functions |
|---|---|---|---|---|---|---|
| CWSNP5569 | Ca-Kabuli-Chr6 | 42004818 | [C/G] | Ca21951 | Synonymous-CDS | TGS |
| CWSNP5570 | Ca-Kabuli-Chr6 | 42004813 | [C/T] | Ca21951 | Synonymous-CDS | TGS |
| CWSNP5571 | Ca-Kabuli-Chr6 | 42161857 | [C/G] | - | URR | - |
| CWSNP5572 | Ca-Kabuli-Chr6 | 42161866 | [T/G] | - | URR | - |
| CWSNP5573 | Ca-Kabuli-Chr6 | 42521162 | [G/T] | Ca23238 | Intron | UDP-glucuronosyl/UDP-glucosyltransferase |
| CWSNP5574 | Ca-Kabuli-Chr6 | 42855091 | [T/G] | Ca23252 | Non-Synonymous-CDS | BTB/POZ-like |
| CWSNP5575 | Ca-Kabuli-Chr6 | 42905012 | [G/A] | Ca23254 | Synonymous-CDS | PeptidaseC48,SUMO/Sentrin/Ubl1 |
| CWSNP5576 | Ca-Kabuli-Chr6 | 42905039 | [C/T] | Ca23254 | Non-Synonymous-CDS | PeptidaseC48,SUMO/Sentrin/Ubl1 |
| CWSNP5577 | Ca-Kabuli-Chr6 | 42905035 | [T/A] | Ca23254 | Non-Synonymous-CDS | PeptidaseC48,SUMO/Sentrin/Ubl1 |
| CWSNP5578 | Ca-Kabuli-Chr6 | 42905094 | [C/T] | Ca23254 | Non-Synonymous-CDS | PeptidaseC48,SUMO/Sentrin/Ubl1 |
| CWSNP5579 | Ca-Kabuli-Chr6 | 42905090 | [G/A] | Ca23254 | Non-Synonymous-CDS | PeptidaseC48,SUMO/Sentrin/Ubl1 |
| CWSNP5580 | Ca-Kabuli-Chr6 | 42905086 | [T/C] | Ca23254 | Synonymous-CDS | PeptidaseC48,SUMO/Sentrin/Ubl1 |
| CWSNP5581 | Ca-Kabuli-Chr6 | 42905058 | [C/G] | Ca23254 | Non-Synonymous-CDS | PeptidaseC48,SUMO/Sentrin/Ubl1 |
| CWSNP5582 | Ca-Kabuli-Chr6 | 42905059 | [A/G] | Ca23254 | Synonymous-CDS | PeptidaseC48,SUMO/Sentrin/Ubl1 |
| CWSNP5583 | Ca-Kabuli-Chr6 | 42905115 | [G/T] | Ca23254 | Non-Synonymous-CDS | PeptidaseC48,SUMO/Sentrin/Ubl1 |
| CWSNP5584 | Ca-Kabuli-Chr6 | 42905116 | [A/T] | Ca23254 | Synonymous-CDS | PeptidaseC48,SUMO/Sentrin/Ubl1 |

| SNP IDs | Chromosomes | Physical positions (bp) | SNPs | Gene accession IDs | Sequence components of genes | Putative functions |
|---|---|---|---|---|---|---|
| CWSNP5585 | Ca-Kabuli-Chr6 | 42923969 | [A/C] | - | Intergenic | - |
| CWSNP5586 | Ca-Kabuli-Chr6 | 43148784 | [C/G] | - | Intergenic | - |
| CWSNP5587 | Ca-Kabuli-Chr6 | 43905509 | [A/T] | - | Intergenic | - |
| CWSNP5588 | Ca-Kabuli-Chr6 | 43907445 | [T/C] | - | Intergenic | - |
| CWSNP5589 | Ca-Kabuli-Chr6 | 44530888 | [C/A] | Ca24241 | Non-Synonymous-CDS | Aspartate/glutamate/uridylatekinase |
| CWSNP5590 | Ca-Kabuli-Chr6 | 44530889 | [A/G] | Ca24241 | Synonymous-CDS | Aspartate/glutamate/uridylatekinase |
| CWSNP5591 | Ca-Kabuli-Chr6 | 44707313 | [T/A] | - | Intergenic | - |
| CWSNP5592 | Ca-Kabuli-Chr6 | 44711166 | [A/G] | - | Intergenic | - |
| CWSNP5593 | Ca-Kabuli-Chr6 | 44944917 | [G/A] | Ca13813 | Non-Synonymous-CDS | Carbohydrate/puinekinase,PfkB,conserved site |
| CWSNP5594 | Ca-Kabuli-Chr6 | 45178060 | [G/A] | - | Intergenic | - |
| CWSNP5595 | Ca-Kabuli-Chr6 | 45178044 | [G/A] | - | Intergenic | - |
| CWSNP5596 | Ca-Kabuli-Chr6 | 45178061 | [C/T] | - | Intergenic | - |
| CWSNP5597 | Ca-Kabuli-Chr6 | 45293271 | [T/C] | - | Intergenic | - |
| CWSNP5598 | Ca-Kabuli-Chr6 | 45500961 | [T/A] | Ca13841 | Non-Synonymous-CDS | Ankyrin repeat |
| CWSNP5599 | Ca-Kabuli-Chr6 | 45505942 | [A/G] | Ca13841 | Non-Synonymous-CDS | Ankyrin repeat |
| CWSNP5600 | Ca-Kabuli-Chr6 | 45550031 | [C/A] | - | Intergenic | - |

| SNP IDs | Chromosomes | Physical positions (bp) | SNPs | Gene accession IDs | Sequence components of genes | Putative functions |
|---|---|---|---|---|---|---|
| CWSNP5601 | Ca-Kabuli-Chr6 | 45554040 | [G/A] | - | Intergenic | - |
| CWSNP5602 | Ca-Kabuli-Chr6 | 45637292 | [C/A] | Ca13850 | Non-Synonymous-CDS | - |
| CWSNP5603 | Ca-Kabuli-Chr6 | 45637293 | [T/C] | Ca13850 | Synonymous-CDS | - |
| CWSNP5604 | Ca-Kabuli-Chr6 | 45637322 | [C/A] | Ca13850 | Non-Synonymous-CDS | - |
| CWSNP5605 | Ca-Kabuli-Chr6 | 45637309 | [G/A] | Ca13850 | Non-Synonymous-CDS | - |
| CWSNP5606 | Ca-Kabuli-Chr6 | 45637308 | [G/T] | Ca13850 | Synonymous-CDS | - |
| CWSNP5607 | Ca-Kabuli-Chr6 | 45693275 | [G/A] | - | Intergenic | - |
| CWSNP5608 | Ca-Kabuli-Chr6 | 45978263 | [G/A] | - | Intergenic | - |
| CWSNP5609 | Ca-Kabuli-Chr6 | 46734706 | [C/G] | - | DRR | - |
| CWSNP5610 | Ca-Kabuli-Chr6 | 46734725 | [G/A] | - | DRR | - |
| CWSNP5611 | Ca-Kabuli-Chr6 | 46734726 | [T/C] | - | DRR | - |
| CWSNP5612 | Ca-Kabuli-Chr6 | 46765659 | [G/A] | Ca17737 | Non-Synonymous-CDS | PeptidaseC48,SUMO/Sentrin/Ubl1 |
| CWSNP5613 | Ca-Kabuli-Chr6 | 46765661 | [A/T] | Ca17737 | Synonymous-CDS | PeptidaseC48,SUMO/Sentrin/Ubl1 |
| CWSNP5614 | Ca-Kabuli-Chr6 | 46765673 | [G/A] | Ca17737 | Synonymous-CDS | PeptidaseC48,SUMO/Sentrin/Ubl1 |
| CWSNP5615 | Ca-Kabuli-Chr6 | 46765725 | [C/T] | Ca17737 | Non-Synonymous-CDS | PeptidaseC48,SUMO/Sentrin/Ubl1 |
| CWSNP5616 | Ca-Kabuli-Chr6 | 46765778 | [C/A] | Ca17737 | Synonymous-CDS | PeptidaseC48,SUMO/Sentrin/Ubl1 |

| SNP IDs | Chromosomes | Physical positions (bp) | SNPs | Gene accession IDs | Sequence components of genes | Putative functions |
|---|---|---|---|---|---|---|
| CWSNP5617 | Ca-Kabuli-Chr6 | 46765696 | [C/T] | Ca17737 | Non-Synonymous-CDS | PeptidaseC48,SUMO/Sentrin/Ubl1 |
| CWSNP5618 | Ca-Kabuli-Chr6 | 46896903 | [C/T] | - | Intergenic | - |
| CWSNP5619 | Ca-Kabuli-Chr6 | 46896910 | [A/C] | - | Intergenic | - |
| CWSNP5620 | Ca-Kabuli-Chr6 | 46898225 | [A/C] | - | Intergenic | - |
| CWSNP5621 | Ca-Kabuli-Chr6 | 46898305 | [T/G] | - | Intergenic | - |
| CWSNP5622 | Ca-Kabuli-Chr6 | 46898316 | [T/G] | - | Intergenic | - |
| CWSNP5623 | Ca-Kabuli-Chr6 | 46898317 | [C/T] | - | Intergenic | - |
| CWSNP5624 | Ca-Kabuli-Chr6 | 46899229 | [G/A] | - | Intergenic | - |
| CWSNP5625 | Ca-Kabuli-Chr6 | 46979240 | [C/T] | - | Intergenic | - |
| CWSNP5626 | Ca-Kabuli-Chr6 | 46979267 | [C/A] | - | Intergenic | - |
| CWSNP5627 | Ca-Kabuli-Chr6 | 47906683 | [G/A] | Ca23445 | Intron | Zinc finger,CCHC-type |
| CWSNP5628 | Ca-Kabuli-Chr6 | 47906680 | [C/T] | Ca23445 | Intron | Zinc finger,CCHC-type |
| CWSNP5629 | Ca-Kabuli-Chr6 | 47906629 | [T/G] | Ca23445 | Intron | Zinc finger,CCHC-type |
| CWSNP5630 | Ca-Kabuli-Chr6 | 48088550 | [G/A] | - | Intergenic | - |
| CWSNP5631 | Ca-Kabuli-Chr6 | 48088592 | [C/T] | - | Intergenic | - |
| CWSNP5632 | Ca-Kabuli-Chr6 | 48255392 | [G/A] | - | Intergenic | - |

| SNP IDs | Chromosomes | Physical positions (bp) | SNPs | Gene accession IDs | Sequence components of genes | Putative functions |
|---|---|---|---|---|---|---|
| CWSNP5633 | Ca-Kabuli-Chr6 | 48255538 | [A/G] | - | Intergenic | - |
| CWSNP5634 | Ca-Kabuli-Chr6 | 48255558 | [C/T] | - | Intergenic | - |
| CWSNP5635 | Ca-Kabuli-Chr6 | 48255559 | [A/G] | - | Intergenic | - |
| CWSNP5636 | Ca-Kabuli-Chr6 | 48255568 | [A/G] | - | Intergenic | - |
| CWSNP5637 | Ca-Kabuli-Chr6 | 48255599 | [C/A] | - | Intergenic | - |
| CWSNP5638 | Ca-Kabuli-Chr6 | 48314052 | [T/A] | Ca24606 | Synonymous-CDS | - |
| CWSNP5639 | Ca-Kabuli-Chr6 | 48315194 | [C/A] | - | Intergenic | - |
| CWSNP5640 | Ca-Kabuli-Chr6 | 48315340 | [C/A] | - | Intergenic | - |
| CWSNP5641 | Ca-Kabuli-Chr6 | 48315343 | [G/T] | - | Intergenic | - |
| CWSNP5642 | Ca-Kabuli-Chr6 | 48864625 | [G/A] | - | Intergenic | - |
| CWSNP5643 | Ca-Kabuli-Chr6 | 48864639 | [A/C] | - | Intergenic | - |
| CWSNP5644 | Ca-Kabuli-Chr6 | 48864699 | [T/C] | - | Intergenic | - |
| CWSNP5645 | Ca-Kabuli-Chr6 | 48869577 | [C/T] | - | Intergenic | - |
| CWSNP5646 | Ca-Kabuli-Chr6 | 48869539 | [A/G] | - | Intergenic | - |
| CWSNP5647 | Ca-Kabuli-Chr6 | 48869529 | [T/C] | - | Intergenic | - |
| CWSNP5648 | Ca-Kabuli-Chr6 | 48869512 | [G/C] | - | Intergenic | - |

| SNP IDs | Chromosomes | Physical positions (bp) | SNPs | Gene accession IDs | Sequence components of genes | Putative functions |
|---|---|---|---|---|---|---|
| CWSNP5649 | Ca-Kabuli-Chr6 | 48940386 | [A/C] | Ca20710 | Synonymous-CDS | Clathrin adaptor complex,small chain |
| CWSNP5650 | Ca-Kabuli-Chr6 | 49044772 | [A/C] | - | Intergenic | - |
| CWSNP5651 | Ca-Kabuli-Chr6 | 49044785 | [C/T] | - | Intergenic | - |
| CWSNP5652 | Ca-Kabuli-Chr6 | 49044794 | [T/A] | - | Intergenic | - |
| CWSNP5653 | Ca-Kabuli-Chr6 | 49044802 | [T/C] | - | Intergenic | - |
| CWSNP5654 | Ca-Kabuli-Chr6 | 49044805 | [A/G] | - | Intergenic | - |
| CWSNP5655 | Ca-Kabuli-Chr6 | 49044814 | [G/T] | - | Intergenic | - |
| CWSNP5656 | Ca-Kabuli-Chr6 | 49044824 | [C/T] | - | Intergenic | - |
| CWSNP5657 | Ca-Kabuli-Chr6 | 49044839 | [T/A] | - | Intergenic | - |
| CWSNP5658 | Ca-Kabuli-Chr6 | 49044850 | [A/C] | - | Intergenic | - |
| CWSNP5659 | Ca-Kabuli-Chr6 | 49044847 | [G/A] | - | Intergenic | - |
| CWSNP5660 | Ca-Kabuli-Chr6 | 49044842 | [G/A] | - | Intergenic | - |
| CWSNP5661 | Ca-Kabuli-Chr6 | 49044838 | [C/A] | - | Intergenic | - |
| CWSNP5662 | Ca-Kabuli-Chr6 | 49044835 | [C/A] | - | Intergenic | - |
| CWSNP5663 | Ca-Kabuli-Chr6 | 49044826 | [G/A] | - | Intergenic | - |
| CWSNP5664 | Ca-Kabuli-Chr6 | 49044821 | [C/G] | - | Intergenic | - |

| SNP IDs | Chromosomes | Physical positions (bp) | SNPs | Gene accession IDs | Sequence components of genes | Putative functions |
|---|---|---|---|---|---|---|
| CWSNP5665 | Ca-Kabuli-Chr6 | 49044810 | [G/A] | - | Intergenic | - |
| CWSNP5666 | Ca-Kabuli-Chr6 | 49044808 | [C/T] | - | Intergenic | - |
| CWSNP5667 | Ca-Kabuli-Chr6 | 49044788 | [T/A] | - | Intergenic | - |
| CWSNP5668 | Ca-Kabuli-Chr6 | 49044780 | [G/A] | - | Intergenic | - |
| CWSNP5669 | Ca-Kabuli-Chr6 | 49044915 | [A/G] | - | Intergenic | - |
| CWSNP5670 | Ca-Kabuli-Chr6 | 49044928 | [C/A] | - | Intergenic | - |
| CWSNP5671 | Ca-Kabuli-Chr6 | 49044937 | [A/G] | - | Intergenic | - |
| CWSNP5672 | Ca-Kabuli-Chr6 | 49044944 | [A/G] | - | Intergenic | - |
| CWSNP5673 | Ca-Kabuli-Chr6 | 49167341 | [T/G] | - | URR | - |
| CWSNP5674 | Ca-Kabuli-Chr6 | 49222291 | [G/A] | - | Intergenic | - |
| CWSNP5675 | Ca-Kabuli-Chr6 | 49222290 | [C/T] | - | Intergenic | - |
| CWSNP5676 | Ca-Kabuli-Chr6 | 49222275 | [A/C] | - | Intergenic | - |
| CWSNP5677 | Ca-Kabuli-Chr6 | 49222268 | [T/A] | - | Intergenic | - |
| CWSNP5678 | Ca-Kabuli-Chr6 | 49222251 | [G/A] | - | Intergenic | - |
| CWSNP5679 | Ca-Kabuli-Chr6 | 49222235 | [G/C] | - | Intergenic | - |
| CWSNP5680 | Ca-Kabuli-Chr6 | 49377275 | [G/A] | - | Intergenic | - |

| SNP IDs | Chromosomes | Physical positions (bp) | SNPs | Gene accession IDs | Sequence components of genes | Putative functions |
|---|---|---|---|---|---|---|
| CWSNP5681 | Ca-Kabuli-Chr6 | 49524179 | [A/T] | - | DRR | - |
| CWSNP5682 | Ca-Kabuli-Chr6 | 49565934 | [C/T] | Ca13509 | Non-Synonymous-CDS | Ribosomal proteinL37e |
| CWSNP5683 | Ca-Kabuli-Chr6 | 49726734 | [C/G] | - | Intergenic | - |
| CWSNP5684 | Ca-Kabuli-Chr6 | 49726729 | [G/A] | - | Intergenic | - |
| CWSNP5685 | Ca-Kabuli-Chr6 | 49726711 | [C/T] | - | Intergenic | - |
| CWSNP5686 | Ca-Kabuli-Chr6 | 49726723 | [G/A] | - | Intergenic | - |
| CWSNP5687 | Ca-Kabuli-Chr6 | 49830299 | [A/G] | Ca13490 | Non-Synonymous-CDS | Heat shock protein Hsp70 |
| CWSNP5688 | Ca-Kabuli-Chr6 | 49846303 | [T/G] | Ca13488 | Synonymous-CDS | Heat shock protein Hsp70 |
| CWSNP5689 | Ca-Kabuli-Chr6 | 50152053 | [C/T] | - | Intergenic | - |
| CWSNP5690 | Ca-Kabuli-Chr6 | 50152124 | [C/T] | - | Intergenic | - |
| CWSNP5691 | Ca-Kabuli-Chr6 | 50477672 | [A/C] | Ca13459 | Synonymous-CDS | - |
| CWSNP5692 | Ca-Kabuli-Chr6 | 50617932 | [G/A] | Ca13450 | Synonymous-CDS | - |
| CWSNP5693 | Ca-Kabuli-Chr6 | 50723595 | [T/C] | - | DRR | - |
| CWSNP5694 | Ca-Kabuli-Chr6 | 51157939 | [A/C] | Ca19183 | Non-Synonymous-CDS | - |
| CWSNP5695 | Ca-Kabuli-Chr6 | 51471573 | [T/C] | Ca19165 | Non-Synonymous-CDS | - |
| CWSNP5696 | Ca-Kabuli-Chr6 | 52007211 | [T/C] | - | Intergenic | - |

| SNP IDs | Chromosomes | Physical positions (bp) | SNPs | Gene accession IDs | Sequence components of genes | Putative functions |
|---|---|---|---|---|---|---|
| CWSNP5697 | Ca-Kabuli-Chr6 | 52007471 | [C/T] | - | Intergenic | - |
| CWSNP5698 | Ca-Kabuli-Chr6 | 52007464 | [C/G] | - | Intergenic | - |
| CWSNP5699 | Ca-Kabuli-Chr6 | 52007440 | [C/T] | - | Intergenic | - |
| CWSNP5700 | Ca-Kabuli-Chr6 | 52065393 | [C/A] | - | Intergenic | - |
| CWSNP5701 | Ca-Kabuli-Chr6 | 52065527 | [T/A] | - | Intergenic | - |
| CWSNP5702 | Ca-Kabuli-Chr6 | 52164571 | [C/A] | Ca17518 | Intron | ATPase,P-type,K/Mg/Cd/Cu/Zn/Na/Ca/Na/H-transporter |
| CWSNP5703 | Ca-Kabuli-Chr6 | 52567474 | [A/C] | Ca17540 | Non-Synonymous-CDS | Transferase |
| CWSNP5704 | Ca-Kabuli-Chr6 | 52858359 | [A/G] | - | Intergenic | - |
| CWSNP5705 | Ca-Kabuli-Chr6 | 52993231 | [A/G] | - | Intergenic | - |
| CWSNP5706 | Ca-Kabuli-Chr6 | 53097833 | [G/C] | - | Intergenic | - |
| CWSNP5707 | Ca-Kabuli-Chr6 | 53097803 | [A/G] | - | Intergenic | - |
| CWSNP5708 | Ca-Kabuli-Chr6 | 53124182 | [G/T] | Ca22912 | Non-Synonymous-CDS | 3-Oxoacyl-[acyl-carrier-protein(ACP)]synthaseIIIC-terminal |
| CWSNP5709 | Ca-Kabuli-Chr6 | 53348993 | [C/A] | Ca18877 | Intron | Sodium/sulphate symporter |
| CWSNP5710 | Ca-Kabuli-Chr6 | 53349034 | [G/A] | Ca18877 | Intron | Sodium/sulphate symporter |
| CWSNP5711 | Ca-Kabuli-Chr6 | 53461929 | [C/A] | - | Intergenic | - |
| CWSNP5712 | Ca-Kabuli-Chr6 | 53461883 | [A/G] | - | Intergenic | - |

| SNP IDs | Chromosomes | Physical positions (bp) | SNPs | Gene accession IDs | Sequence components of genes | Putative functions |
|---|---|---|---|---|---|---|
| CWSNP5713 | Ca-Kabuli-Chr6 | 53541031 | [G/A] | - | Intergenic | - |
| CWSNP5714 | Ca-Kabuli-Chr6 | 53541094 | [C/G] | - | Intergenic | - |
| CWSNP5715 | Ca-Kabuli-Chr6 | 53541111 | [T/G] | - | Intergenic | - |
| CWSNP5716 | Ca-Kabuli-Chr6 | 53551878 | [C/A] | - | URR | - |
| CWSNP5717 | Ca-Kabuli-Chr6 | 53558430 | [A/C] | - | Intergenic | - |
| CWSNP5718 | Ca-Kabuli-Chr6 | 53566496 | [G/A] | Ca18894 | Non-Synonymous-CDS | Pathogenesis-related transcriptional factor/ERF, DNA-binding |
| CWSNP5719 | Ca-Kabuli-Chr6 | 53625264 | [A/C] | Ca18898 | Synonymous-CDS | Leucine-rich repeat |
| CWSNP5720 | Ca-Kabuli-Chr6 | 53683753 | [T/C] | Ca18901 | Intron | - |
| CWSNP5721 | Ca-Kabuli-Chr6 | 53683891 | [C/A] | Ca18901 | Intron | - |
| CWSNP5722 | Ca-Kabuli-Chr6 | 53683885 | [C/T] | Ca18901 | Intron | - |
| CWSNP5723 | Ca-Kabuli-Chr6 | 53683876 | [C/T] | Ca18901 | Intron | - |
| CWSNP5724 | Ca-Kabuli-Chr6 | 53692784 | [G/A] | - | DRR | - |
| CWSNP5725 | Ca-Kabuli-Chr6 | 53769160 | [C/T] | - | DRR | - |
| CWSNP5726 | Ca-Kabuli-Chr6 | 54151902 | [T/G] | - | Intergenic | - |
| CWSNP5727 | Ca-Kabuli-Chr6 | 54151966 | [G/C] | - | Intergenic | - |
| CWSNP5728 | Ca-Kabuli-Chr6 | 54151969 | [T/G] | - | Intergenic | - |

| SNP IDs | Chromosomes | Physical positions (bp) | SNPs | Gene accession IDs | Sequence components of genes | Putative functions |
|---|---|---|---|---|---|---|
| CWSNP5729 | Ca-Kabuli-Chr6 | 54203356 | [G/A] | Ca16276 | Non-Synonymous-CDS | NmrA-like |
| CWSNP5730 | Ca-Kabuli-Chr6 | 54203400 | [C/T] | Ca16276 | Synonymous-CDS | NmrA-like |
| CWSNP5731 | Ca-Kabuli-Chr6 | 54229449 | [G/T] | - | Intergenic | - |
| CWSNP5732 | Ca-Kabuli-Chr6 | 54237764 | [A/C] | - | Intergenic | - |
| CWSNP5733 | Ca-Kabuli-Chr6 | 54250702 | [C/T] | Ca16283 | Non-Synonymous-CDS | Pumilio RNA-binding repeat |
| CWSNP5734 | Ca-Kabuli-Chr6 | 54368267 | [G/A] | - | Intergenic | - |
| CWSNP5735 | Ca-Kabuli-Chr6 | 55024071 | [C/T] | Ca21213 | Non-Synonymous-CDS | - |
| CWSNP5736 | Ca-Kabuli-Chr6 | 55024146 | [C/T] | Ca21213 | Non-Synonymous-CDS | - |
| CWSNP5737 | Ca-Kabuli-Chr6 | 55024140 | [G/A] | Ca21213 | Non-Synonymous-CDS | - |
| CWSNP5738 | Ca-Kabuli-Chr6 | 55024130 | [T/A] | Ca21213 | Non-Synonymous-CDS | - |
| CWSNP5739 | Ca-Kabuli-Chr6 | 55024112 | [C/G] | Ca21213 | Non-Synonymous-CDS | - |
| CWSNP5740 | Ca-Kabuli-Chr6 | 55024093 | [G/C] | Ca21213 | Non-Synonymous-CDS | - |
| CWSNP5741 | Ca-Kabuli-Chr6 | 55024108 | [C/T] | Ca21213 | Synonymous-CDS | - |
| CWSNP5742 | Ca-Kabuli-Chr6 | 55024159 | [T/A] | Ca21213 | Synonymous-CDS | - |
| CWSNP5743 | Ca-Kabuli-Chr6 | 55024198 | [T/G] | Ca21213 | Synonymous-CDS | - |
| CWSNP5744 | Ca-Kabuli-Chr6 | 55074979 | [T/A] | - | Intergenic | - |

| SNP IDs | Chromosomes | Physical positions (bp) | SNPs | Gene accession IDs | Sequence components of genes | Putative functions |
|---|---|---|---|---|---|---|
| CWSNP5745 | Ca-Kabuli-Chr6 | 55083311 | [T/A] | Ca21214 | Intron | Oligopeptide transporter |
| CWSNP5746 | Ca-Kabuli-Chr6 | 55083721 | [T/A] | Ca21214 | Synonymous-CDS | Oligopeptide transporter |
| CWSNP5747 | Ca-Kabuli-Chr6 | 55332515 | [G/A] | Ca21219 | Synonymous-CDS | Homeobox |
| CWSNP5748 | Ca-Kabuli-Chr6 | 55922843 | [T/A] | Ca19023 | Non-Synonymous-CDS | Protein of unknown functionwound-induced |
| CWSNP5749 | Ca-Kabuli-Chr6 | 55942105 | [A/G] | - | Intergenic | - |
| CWSNP5750 | Ca-Kabuli-Chr6 | 55953320 | [T/A] | Ca19026 | Non-Synonymous-CDS | Protein of unknown functionwound-induced |
| CWSNP5751 | Ca-Kabuli-Chr6 | 56137878 | [T/C] | - | Intergenic | - |
| CWSNP5752 | Ca-Kabuli-Chr6 | 56553930 | [G/A] | Ca17393 | Non-Synonymous-CDS | TetratricopeptideTPR-1 |
| CWSNP5753 | Ca-Kabuli-Chr6 | 56564249 | [G/C] | - | URR | - |
| CWSNP5754 | Ca-Kabuli-Chr6 | 56587094 | [C/G] | - | Intergenic | - |
| CWSNP5755 | Ca-Kabuli-Chr6 | 56593121 | [T/G] | Ca17397 | Synonymous-CDS | Helicase,C-terminal |
| CWSNP5756 | Ca-Kabuli-Chr6 | 56624316 | [A/T] | Ca17398 | Intron | Proline dehydrogenase |
| CWSNP5757 | Ca-Kabuli-Chr6 | 56624674 | [T/C] | Ca17398 | Intron | Proline dehydrogenase |
| CWSNP5758 | Ca-Kabuli-Chr6 | 56624718 | [C/A] | Ca17398 | Intron | Proline dehydrogenase |
| CWSNP5759 | Ca-Kabuli-Chr6 | 56694898 | [T/C] | Ca17408 | Non-Synonymous-CDS | ATPase,AAA+type,core |
| CWSNP5760 | Ca-Kabuli-Chr6 | 56793077 | [G/A] | - | Intergenic | - |

| SNP IDs | Chromosomes | Physical positions (bp) | SNPs | Gene accession IDs | Sequence components of genes | Putative functions |
|---|---|---|---|---|---|---|
| CWSNP5761 | Ca-Kabuli-Chr6 | 56793037 | [A/T] | - | Intergenic | - |
| CWSNP5762 | Ca-Kabuli-Chr6 | 56793021 | [G/T] | - | Intergenic | - |
| CWSNP5763 | Ca-Kabuli-Chr6 | 56793122 | [C/T] | - | Intergenic | - |
| CWSNP5764 | Ca-Kabuli-Chr6 | 56822084 | [G/T] | Ca17420 | Intron | Domain of unknown function DUF231,plant |
| CWSNP5765 | Ca-Kabuli-Chr6 | 56822060 | [G/A] | Ca17420 | Intron | Domain of unknown function DUF231,plant |
| CWSNP5766 | Ca-Kabuli-Chr6 | 56840030 | [A/C] | - | Intergenic | - |
| CWSNP5767 | Ca-Kabuli-Chr6 | 56840063 | [T/A] | - | Intergenic | - |
| CWSNP5768 | Ca-Kabuli-Chr6 | 56854753 | [T/C] | Ca17422 | Synonymous-CDS | - |
| CWSNP5769 | Ca-Kabuli-Chr6 | 56950487 | [G/T] | - | DRR | - |
| CWSNP5770 | Ca-Kabuli-Chr6 | 56980313 | [T/C] | Ca17429 | Intron | GDP-fucose proteinO-fucosyltransferase |
| CWSNP5771 | Ca-Kabuli-Chr6 | 56980286 | [T/C] | Ca17429 | Intron | GDP-fucose proteinO-fucosyltransferase |
| CWSNP5772 | Ca-Kabuli-Chr6 | 56980321 | [T/C] | Ca17429 | Intron | GDP-fucose proteinO-fucosyltransferase |
| CWSNP5773 | Ca-Kabuli-Chr6 | 57198837 | [G/A] | Ca17442 | Intron | - |
| CWSNP5774 | Ca-Kabuli-Chr6 | 57272604 | [A/G] | - | Intergenic | - |
| CWSNP5775 | Ca-Kabuli-Chr6 | 57272607 | [G/A] | - | Intergenic | - |
| CWSNP5776 | Ca-Kabuli-Chr6 | 57272608 | [C/A] | - | Intergenic | - |

| SNP IDs | Chromosomes | Physical positions (bp) | SNPs | Gene accession IDs | Sequence components of genes | Putative functions |
|---|---|---|---|---|---|---|
| CWSNP5777 | Ca-Kabuli-Chr6 | 57277440 | [C/T] | - | Intergenic | - |
| CWSNP5778 | Ca-Kabuli-Chr6 | 57382220 | [G/A] | Ca13705 | Non-Synonymous-CDS | Zinc finger,RING-type |
| CWSNP5779 | Ca-Kabuli-Chr6 | 57436320 | [T/G] | Ca13700 | Non-Synonymous-CDS | Bromodomain |
| CWSNP5780 | Ca-Kabuli-Chr6 | 57437904 | [A/G] | Ca13700 | Synonymous-CDS | Bromodomain |
| CWSNP5781 | Ca-Kabuli-Chr6 | 57449892 | [A/G] | Ca13699 | Non-Synonymous-CDS | - |
| CWSNP5782 | Ca-Kabuli-Chr6 | 57533087 | [G/A] | - | DRR | - |
| CWSNP5783 | Ca-Kabuli-Chr6 | 57579770 | [G/T] | Ca13686 | Synonymous-CDS | RNA recognition motif domain |
| CWSNP5784 | Ca-Kabuli-Chr6 | 57579768 | [C/T] | Ca13686 | Synonymous-CDS | RNA recognition motif domain |
| CWSNP5785 | Ca-Kabuli-Chr6 | 57603257 | [T/A] | Ca13685 | Intron | 3-betahydroxy steroid dehydrogenase/isomerase |
| CWSNP5786 | Ca-Kabuli-Chr6 | 57625134 | [G/A] | Ca13683 | Intron | Calreticulin/calnexin |
| CWSNP5787 | Ca-Kabuli-Chr6 | 57625320 | [G/A] | Ca13683 | Synonymous-CDS | Calreticulin/calnexin |
| CWSNP5788 | Ca-Kabuli-Chr6 | 57632486 | [C/T] | Ca13682 | Synonymous-CDS | Protein of unknown function DUF936,plant |
| CWSNP5789 | Ca-Kabuli-Chr6 | 57634387 | [G/T] | Ca13682 | Non-Synonymous-CDS | Protein of unknown function DUF936,plant |
| CWSNP5790 | Ca-Kabuli-Chr6 | 57634677 | [G/A] | Ca13682 | Synonymous-CDS | Protein of unknown function DUF936,plant |
| CWSNP5791 | Ca-Kabuli-Chr6 | 57637091 | [G/A] | - | URR | - |
| CWSNP5792 | Ca-Kabuli-Chr6 | 57637083 | [T/G] | - | URR | - |

| SNP IDs | Chromosomes | Physical positions (bp) | SNPs | Gene accession IDs | Sequence components of genes | Putative functions |
|---|---|---|---|---|---|---|
| CWSNP5793 | Ca-Kabuli-Chr6 | 57637075 | [C/T] | - | URR | - |
| CWSNP5794 | Ca-Kabuli-Chr6 | 57667515 | [A/T] | - | DRR | - |
| CWSNP5795 | Ca-Kabuli-Chr6 | 57704057 | [C/T] | - | URR | - |
| CWSNP5796 | Ca-Kabuli-Chr6 | 57704802 | [T/C] | - | Intergenic | - |
| CWSNP5797 | Ca-Kabuli-Chr6 | 57705011 | [G/A] | - | Intergenic | - |
| CWSNP5798 | Ca-Kabuli-Chr6 | 57707716 | [C/T] | - | Intergenic | - |
| CWSNP5799 | Ca-Kabuli-Chr6 | 57707724 | [A/C] | - | Intergenic | - |
| CWSNP5800 | Ca-Kabuli-Chr6 | 57707727 | [T/G] | - | Intergenic | - |
| CWSNP5801 | Ca-Kabuli-Chr6 | 57707848 | [A/G] | - | DRR | - |
| CWSNP5802 | Ca-Kabuli-Chr6 | 57707846 | [G/A] | - | DRR | - |
| CWSNP5803 | Ca-Kabuli-Chr6 | 57707912 | [G/C] | - | DRR | - |
| CWSNP5804 | Ca-Kabuli-Chr6 | 57708019 | [A/G] | - | DRR | - |
| CWSNP5805 | Ca-Kabuli-Chr6 | 57708130 | [T/C] | - | DRR | - |
| CWSNP5806 | Ca-Kabuli-Chr6 | 57708101 | [T/G] | - | DRR | - |
| CWSNP5807 | Ca-Kabuli-Chr6 | 57708198 | [C/T] | - | DRR | - |
| CWSNP5808 | Ca-Kabuli-Chr6 | 57710133 | [C/G] | - | DRR | - |

| SNP IDs | Chromosomes | Physical positions (bp) | SNPs | Gene accession IDs | Sequence components of genes | Putative functions |
|---|---|---|---|---|---|---|
| CWSNP5809 | Ca-Kabuli-Chr6 | 57720446 | [T/C] | Ca13671 | Synonymous-CDS | Transcriptional factor B3 |
| CWSNP5810 | Ca-Kabuli-Chr6 | 57746989 | [C/T] | Ca13666 | Non-Synonymous-CDS | Transcriptional factor B3 |
| CWSNP5811 | Ca-Kabuli-Chr6 | 57747101 | [C/T] | Ca13666 | Synonymous-CDS | Transcriptional factor B3 |
| CWSNP5812 | Ca-Kabuli-Chr6 | 57748565 | [A/G] | Ca13666 | Synonymous-CDS | Transcriptional factor B3 |
| CWSNP5813 | Ca-Kabuli-Chr6 | 57748602 | [A/T] | Ca13666 | Non-Synonymous-CDS | Transcriptional factor B3 |
| CWSNP5814 | Ca-Kabuli-Chr6 | 57753076 | [T/C] | - | Intergenic | - |
| CWSNP5815 | Ca-Kabuli-Chr6 | 57756699 | [G/A] | Ca13665 | Intron | Phosphatidyl inositol 3-/4-kinase, catalytic |
| CWSNP5816 | Ca-Kabuli-Chr6 | 57760176 | [C/T] | Ca13665 | Intron | Phosphatidyl inositol 3-/4-kinase, catalytic |
| CWSNP5817 | Ca-Kabuli-Chr6 | 57760227 | [C/T] | Ca13665 | Synonymous-CDS | Phosphatidyl inositol 3-/4-kinase, catalytic |
| CWSNP5818 | Ca-Kabuli-Chr6 | 57760360 | [A/G] | Ca13665 | Intron | Phosphatidyl inositol 3-/4-kinase, catalytic |
| CWSNP5819 | Ca-Kabuli-Chr6 | 57761758 | [G/A] | Ca13665 | Non-Synonymous-CDS | Phosphatidyl inositol 3-/4-kinase, catalytic |
| CWSNP5820 | Ca-Kabuli-Chr6 | 57766444 | [C/T] | - | DRR | - |
| CWSNP5821 | Ca-Kabuli-Chr6 | 57766570 | [C/T] | - | DRR | - |
| CWSNP5822 | Ca-Kabuli-Chr6 | 57766588 | [G/A] | - | DRR | - |
| CWSNP5823 | Ca-Kabuli-Chr6 | 57766598 | [G/A] | - | DRR | - |
| CWSNP5824 | Ca-Kabuli-Chr6 | 57801985 | [A/C] | Ca13661 | Intron | Proteasome assembly chaperone2 |

| SNP IDs | Chromosomes | Physical positions (bp) | SNPs | Gene accession IDs | Sequence components of genes | Putative functions |
|---|---|---|---|---|---|---|
| CWSNP5825 | Ca-Kabuli-Chr6 | 57831050 | [G/T] | - | DRR | - |
| CWSNP5826 | Ca-Kabuli-Chr6 | 57831049 | [C/T] | - | DRR | - |
| CWSNP5827 | Ca-Kabuli-Chr6 | 57831040 | [T/C] | - | DRR | - |
| CWSNP5828 | Ca-Kabuli-Chr6 | 57835093 | [T/A] | - | Intergenic | - |
| CWSNP5829 | Ca-Kabuli-Chr6 | 57835120 | [G/A] | - | Intergenic | - |
| CWSNP5830 | Ca-Kabuli-Chr6 | 57835917 | [G/C] | - | DRR | - |
| CWSNP5831 | Ca-Kabuli-Chr6 | 57835899 | [C/T] | - | DRR | - |
| CWSNP5832 | Ca-Kabuli-Chr6 | 57836024 | [G/A] | - | DRR | - |
| CWSNP5833 | Ca-Kabuli-Chr6 | 57836237 | [A/G] | - | DRR | - |
| CWSNP5834 | Ca-Kabuli-Chr6 | 57836284 | [G/A] | - | DRR | - |
| CWSNP5835 | Ca-Kabuli-Chr6 | 57836314 | [T/A] | - | DRR | - |
| CWSNP5836 | Ca-Kabuli-Chr6 | 57836316 | [G/A] | - | DRR | - |
| CWSNP5837 | Ca-Kabuli-Chr6 | 57870893 | [C/A] | - | Intergenic | - |
| CWSNP5838 | Ca-Kabuli-Chr6 | 57877446 | [C/A] | - | URR | - |
| CWSNP5839 | Ca-Kabuli-Chr6 | 57894377 | [A/G] | - | Intergenic | - |
| CWSNP5840 | Ca-Kabuli-Chr6 | 57894495 | [C/G] | - | URR | - |

| SNP IDs | Chromosomes | Physical positions (bp) | SNPs | Gene accession IDs | Sequence components of genes | Putative functions |
|---|---|---|---|---|---|---|
| CWSNP5841 | Ca-Kabuli-Chr6 | 57894590 | [G/C] | - | URR | - |
| CWSNP5842 | Ca-Kabuli-Chr6 | 57894585 | [T/G] | - | URR | - |
| CWSNP5843 | Ca-Kabuli-Chr6 | 57943585 | [A/T] | - | Intergenic | - |
| CWSNP5844 | Ca-Kabuli-Chr6 | 57943626 | [G/A] | - | Intergenic | - |
| CWSNP5845 | Ca-Kabuli-Chr6 | 57943628 | [A/C] | - | Intergenic | - |
| CWSNP5846 | Ca-Kabuli-Chr6 | 57968924 | [A/T] | Ca13640 | Intron | WD40 repeat |
| CWSNP5847 | Ca-Kabuli-Chr6 | 58036471 | [C/T] | - | Intergenic | - |
| CWSNP5848 | Ca-Kabuli-Chr6 | 58174702 | [T/C] | Ca13623 | Intron | Glycosidehydrolase,family17 |
| CWSNP5849 | Ca-Kabuli-Chr6 | 58187822 | [T/G] | Ca13622 | Intron | Adenylatecyclase-associatedCAP |
| CWSNP5850 | Ca-Kabuli-Chr6 | 58187957 | [A/G] | Ca13622 | Synonymous-CDS | Adenylatecyclase-associatedCAP |
| CWSNP5851 | Ca-Kabuli-Chr6 | 58224023 | [G/T] | - | DRR | - |
| CWSNP5852 | Ca-Kabuli-Chr6 | 58227006 | [T/C] | - | DRR | - |
| CWSNP5853 | Ca-Kabuli-Chr6 | 58237503 | [A/T] | - | URR | - |
| CWSNP5854 | Ca-Kabuli-Chr6 | 58243505 | [G/A] | Ca15368 | Synonymous-CDS | Mitochodrial transcription termination factor-related |
| CWSNP5855 | Ca-Kabuli-Chr6 | 58326344 | [A/T] | - | Intergenic | - |
| CWSNP5856 | Ca-Kabuli-Chr6 | 58380367 | [T/C] | Ca15376 | Synonymous-CDS | Ribosomal RNA methyltransferase RrmJ/FtsJ |

| SNP IDs | Chromosomes | Physical positions (bp) | SNPs | Gene accession IDs | Sequence components of genes | Putative functions |
|---|---|---|---|---|---|---|
| CWSNP5857 | Ca-Kabuli-Chr6 | 58380394 | [G/A] | Ca15376 | Synonymous-CDS | Ribosomal RNA methyltransferase RrmJ/FtsJ |
| CWSNP5858 | Ca-Kabuli-Chr6 | 58380563 | [G/A] | Ca15376 | Non-Synonymous-CDS | Ribosomal RNA methyltransferase RrmJ/FtsJ |
| CWSNP5859 | Ca-Kabuli-Chr6 | 58434217 | [C/G] | Ca15379 | Non-Synonymous-CDS | - |
| CWSNP5860 | Ca-Kabuli-Chr6 | 58452843 | [A/C] | - | Intergenic | - |
| CWSNP5861 | Ca-Kabuli-Chr6 | 58569576 | [G/T] | Ca15398 | Non-Synonymous-CDS | Pre-mRNA processing ribonucleo protein,snoRNA-binding domain |
| CWSNP5862 | Ca-Kabuli-Chr6 | 58569569 | [T/C] | Ca15398 | Synonymous-CDS | Pre-mRNA processing ribonucleo protein,snoRNA-binding domain |
| CWSNP5863 | Ca-Kabuli-Chr6 | 58571660 | [T/C] | Ca15398 | Synonymous-CDS | Pre-mRNA processing ribonucleo protein,snoRNA-binding domain |
| CWSNP5864 | Ca-Kabuli-Chr6 | 58596821 | [T/G] | - | DRR | - |
| CWSNP5865 | Ca-Kabuli-Chr6 | 58596878 | [C/A] | - | DRR | - |
| CWSNP5866 | Ca-Kabuli-Chr6 | 58623095 | [A/G] | Ca15407 | Intron | ATPase,V0/A0complex,116 kDa subunit |
| CWSNP5867 | Ca-Kabuli-Chr6 | 58623096 | [A/T] | Ca15407 | Intron | ATPase,V0/A0complex,116 kDa subunit |
| CWSNP5868 | Ca-Kabuli-Chr6 | 58624183 | [G/A] | Ca15407 | Intron | ATPase,V0/A0complex,116 kDa subunit |
| CWSNP5869 | Ca-Kabuli-Chr6 | 58624233 | [C/A] | Ca15407 | Intron | ATPase,V0/A0complex,116 kDa subunit |
| CWSNP5870 | Ca-Kabuli-Chr6 | 58624299 | [A/G] | Ca15407 | Intron | ATPase,V0/A0complex,116 kDa subunit |
| CWSNP5871 | Ca-Kabuli-Chr6 | 58650318 | [A/C] | Ca15410 | Synonymous-CDS | WD40 repeat |
| CWSNP5872 | Ca-Kabuli-Chr6 | 58650459 | [A/T] | Ca15410 | Non-Synonymous-CDS | WD40 repeat |

| SNP IDs | Chromosomes | Physical positions (bp) | SNPs | Gene accession IDs | Sequence components of genes | Putative functions |
|---|---|---|---|---|---|---|
| CWSNP5873 | Ca-Kabuli-Chr6 | 58650491 | [A/G] | Ca15410 | Non-Synonymous-CDS | WD40 repeat |
| CWSNP5874 | Ca-Kabuli-Chr6 | 58652881 | [A/G] | Ca15410 | Synonymous-CDS | WD40 repeat |
| CWSNP5875 | Ca-Kabuli-Chr6 | 58672317 | [A/G] | - | DRR | - |
| CWSNP5876 | Ca-Kabuli-Chr6 | 58675749 | [T/C] | Ca15413 | Synonymous-CDS | Kinesin , motor domain |
| CWSNP5877 | Ca-Kabuli-Chr6 | 58676507 | [A/G] | Ca15413 | Synonymous-CDS | Kinesin , motor domain |
| CWSNP5878 | Ca-Kabuli-Chr6 | 58676517 | [A/C] | Ca15413 | Non-Synonymous-CDS | Kinesin , motor domain |
| CWSNP5879 | Ca-Kabuli-Chr6 | 58682397 | [G/A] | Ca15413 | Intron | Kinesin , motor domain |
| CWSNP5880 | Ca-Kabuli-Chr6 | 58732424 | [G/T] | Ca15417 | Intron | Multiantimicrobial extrusion protein |
| CWSNP5881 | Ca-Kabuli-Chr6 | 58732423 | [C/T] | Ca15417 | Intron | Multiantimicrobial extrusion protein |
| CWSNP5882 | Ca-Kabuli-Chr6 | 58732451 | [T/C] | Ca15417 | Synonymous-CDS | Multiantimicrobial extrusion protein |
| CWSNP5883 | Ca-Kabuli-Chr6 | 58732475 | [G/A] | Ca15417 | Synonymous-CDS | Multiantimicrobial extrusion protein |
| CWSNP5884 | Ca-Kabuli-Chr6 | 58745356 | [A/G] | Ca15419 | Synonymous-CDS | Multiantimicrobial extrusion protein |
| CWSNP5885 | Ca-Kabuli-Chr6 | 58745473 | [A/G] | Ca15419 | Synonymous-CDS | Multiantimicrobial extrusion protein |
| CWSNP5886 | Ca-Kabuli-Chr6 | 58745426 | [T/C] | Ca15419 | Intron | Multiantimicrobial extrusion protein |
| CWSNP5887 | Ca-Kabuli-Chr6 | 58745583 | [C/T] | Ca15419 | Non-Synonymous-CDS | Multiantimicrobial extrusion protein |
| CWSNP5888 | Ca-Kabuli-Chr6 | 58761879 | [G/A] | - | Intergenic | - |

| SNP IDs | Chromosomes | Physical positions (bp) | SNPs | Gene accession IDs | Sequence components of genes | Putative functions |
|---|---|---|---|---|---|---|
| CWSNP5889 | Ca-Kabuli-Chr6 | 58779344 | [A/G] | Ca15421 | Intron | Srchomology-3domain |
| CWSNP5890 | Ca-Kabuli-Chr6 | 58779389 | [T/G] | Ca15421 | Synonymous-CDS | Srchomology-3domain |
| CWSNP5891 | Ca-Kabuli-Chr6 | 58820632 | [A/C] | - | Intergenic | - |
| CWSNP5892 | Ca-Kabuli-Chr6 | 58844717 | [T/C] | - | Intergenic | - |
| CWSNP5893 | Ca-Kabuli-Chr6 | 58844776 | [C/G] | - | Intergenic | - |
| CWSNP5894 | Ca-Kabuli-Chr6 | 58878604 | [C/G] | Ca15428 | Synonymous-CDS | Ornithine/DAP/Argdecarboxylase |
| CWSNP5895 | Ca-Kabuli-Chr6 | 58879570 | [T/C] | Ca15428 | Synonymous-CDS | Ornithine/DAP/Argdecarboxylase |
| CWSNP5896 | Ca-Kabuli-Chr6 | 58903346 | [G/A] | Ca15431 | Intron | Aldo/ketoreductase |
| CWSNP5897 | Ca-Kabuli-Chr6 | 58903381 | [G/A] | Ca15431 | Intron | Aldo/ketoreductase |
| CWSNP5898 | Ca-Kabuli-Chr6 | 58903395 | [A/G] | Ca15431 | Intron | Aldo/ketoreductase |
| CWSNP5899 | Ca-Kabuli-Chr6 | 58903416 | [T/G] | Ca15431 | Intron | Aldo/ketoreductase |
| CWSNP5900 | Ca-Kabuli-Chr6 | 58903497 | [G/C] | Ca15431 | Intron | Aldo/ketoreductase |
| CWSNP5901 | Ca-Kabuli-Chr6 | 58903538 | [G/A] | Ca15431 | Intron | Aldo/ketoreductase |
| CWSNP5902 | Ca-Kabuli-Chr6 | 58908024 | [G/A] | Ca15431 | Synonymous-CDS | Aldo/ketoreductase |
| CWSNP5903 | Ca-Kabuli-Chr6 | 58908069 | [G/A] | Ca15431 | Synonymous-CDS | Aldo/ketoreductase |
| CWSNP5904 | Ca-Kabuli-Chr6 | 58951079 | [T/C] | Ca18874 | Synonymous-CDS | Clathrin/coatomer adaptor,adaptin-like,N-terminal |

| SNP IDs | Chromosomes | Physical positions (bp) | SNPs | Gene accession IDs | Sequence components of genes | Putative functions |
|---|---|---|---|---|---|---|
| CWSNP5905 | Ca-Kabuli-Chr6 | 58953219 | [A/G] | Ca18874 | Intron | Clathrin/coatomer adaptor,adaptin-like,N-terminal |
| CWSNP5906 | Ca-Kabuli-Chr6 | 59003792 | [A/C] | Ca18871 | Intron | RNA recognition motif domain |
| CWSNP5907 | Ca-Kabuli-Chr6 | 59064798 | [G/T] | - | Intergenic | - |
| CWSNP5908 | Ca-Kabuli-Chr6 | 59064815 | [C/T] | - | Intergenic | - |
| CWSNP5909 | Ca-Kabuli-Chr6 | 59081169 | [C/T] | Ca18866 | Synonymous-CDS | Glycosyltransferase,family2 |
| CWSNP5910 | Ca-Kabuli-Chr6 | 59107027 | [A/T] | Ca18863 | Non-Synonymous-CDS | RNA-binding,CRM domain |
| CWSNP5911 | Ca-Kabuli-Chr6 | 59107007 | [G/T] | Ca18863 | Non-Synonymous-CDS | RNA-binding,CRM domain |
| CWSNP5912 | Ca-Kabuli-Chr6 | 59135944 | [A/C] | Ca18861 | Synonymous-CDS | Protein kinase, catalytic domain |
| CWSNP5913 | Ca-Kabuli-Chr6 | 59194015 | [C/G] | Ca18855 | Synonymous-CDS | - |
| CWSNP5914 | Ca-Kabuli-Chr6 | 59193994 | [G/T] | Ca18855 | Synonymous-CDS | - |
| CWSNP5915 | Ca-Kabuli-Chr6 | 59194081 | [T/A] | Ca18855 | Synonymous-CDS | - |
| CWSNP5916 | Ca-Kabuli-Chr6 | 59219099 | [T/C] | Ca18853 | Synonymous-CDS | Ergosterolbio synthesis ERG4/ERG24 |
| CWSNP5917 | Ca-Kabuli-Chr6 | 59222848 | [C/T] | - | DRR | - |
| CWSNP5918 | Ca-Kabuli-Chr6 | 59296368 | [C/T] | Ca18847 | Non-Synonymous-CDS | YTH domain |
| CWSNP5919 | Ca-Kabuli-Chr6 | 59407761 | [G/A] | - | URR | - |
| CWSNP5920 | Ca-Kabuli-Chr7 | 16881 | [A/G] | Ca20246 | Non-Synonymous-CDS | RNA helicase, ATP-dependent, DEAD-box,conserved site |

| SNP IDs | Chromosomes | Physical positions (bp) | SNPs | Gene accession IDs | Sequence components of genes | Putative functions |
|---|---|---|---|---|---|---|
| CWSNP5921 | Ca-Kabuli-Chr7 | 419301 | [C/T] | - | Intergenic | - |
| CWSNP5922 | Ca-Kabuli-Chr7 | 419316 | [A/G] | - | Intergenic | - |
| CWSNP5923 | Ca-Kabuli-Chr7 | 419334 | [A/C] | - | Intergenic | - |
| CWSNP5924 | Ca-Kabuli-Chr7 | 419385 | [T/G] | - | Intergenic | - |
| CWSNP5925 | Ca-Kabuli-Chr7 | 419628 | [A/C] | - | Intergenic | - |
| CWSNP5926 | Ca-Kabuli-Chr7 | 528521 | [G/A] | - | Intergenic | - |
| CWSNP5927 | Ca-Kabuli-Chr7 | 601563 | [T/A] | - | Intergenic | - |
| CWSNP5928 | Ca-Kabuli-Chr7 | 601551 | [T/C] | - | Intergenic | - |
| CWSNP5929 | Ca-Kabuli-Chr7 | 601545 | [C/T] | - | Intergenic | - |
| CWSNP5930 | Ca-Kabuli-Chr7 | 601538 | [C/A] | - | Intergenic | - |
| CWSNP5931 | Ca-Kabuli-Chr7 | 601521 | [G/A] | - | Intergenic | - |
| CWSNP5932 | Ca-Kabuli-Chr7 | 601516 | [G/A] | - | Intergenic | - |
| CWSNP5933 | Ca-Kabuli-Chr7 | 601513 | [C/G] | - | Intergenic | - |
| CWSNP5934 | Ca-Kabuli-Chr7 | 601502 | [G/T] | - | Intergenic | - |
| CWSNP5935 | Ca-Kabuli-Chr7 | 601496 | [C/T] | - | Intergenic | - |
| CWSNP5936 | Ca-Kabuli-Chr7 | 601586 | [C/T] | - | Intergenic | - |

| SNP IDs | Chromosomes | Physical positions (bp) | SNPs | Gene accession IDs | Sequence components of genes | Putative functions |
|---|---|---|---|---|---|---|
| CWSNP5937 | Ca-Kabuli-Chr7 | 601601 | [C/T] | - | Intergenic | - |
| CWSNP5938 | Ca-Kabuli-Chr7 | 601612 | [A/G] | - | Intergenic | - |
| CWSNP5939 | Ca-Kabuli-Chr7 | 601643 | [G/T] | - | Intergenic | - |
| CWSNP5940 | Ca-Kabuli-Chr7 | 601622 | [G/C] | - | Intergenic | - |
| CWSNP5941 | Ca-Kabuli-Chr7 | 601657 | [C/T] | - | Intergenic | - |
| CWSNP5942 | Ca-Kabuli-Chr7 | 601719 | [G/T] | - | Intergenic | - |
| CWSNP5943 | Ca-Kabuli-Chr7 | 601718 | [G/C] | - | Intergenic | - |
| CWSNP5944 | Ca-Kabuli-Chr7 | 601714 | [A/T] | - | Intergenic | - |
| CWSNP5945 | Ca-Kabuli-Chr7 | 601709 | [A/C] | - | Intergenic | - |
| CWSNP5946 | Ca-Kabuli-Chr7 | 601647 | [C/T] | - | Intergenic | - |
| CWSNP5947 | Ca-Kabuli-Chr7 | 610737 | [C/A] | Ca24417 | Non-Synonymous-CDS | Transcription factor GRAS |
| CWSNP5948 | Ca-Kabuli-Chr7 | 610723 | [G/T] | Ca24417 | Non-Synonymous-CDS | Transcription factor GRAS |
| CWSNP5949 | Ca-Kabuli-Chr7 | 674791 | [A/T] | Ca03417 | Intron | HECT |
| CWSNP5950 | Ca-Kabuli-Chr7 | 676785 | [G/T] | - | Intergenic | - |
| CWSNP5951 | Ca-Kabuli-Chr7 | 676786 | [G/A] | - | Intergenic | - |
| CWSNP5952 | Ca-Kabuli-Chr7 | 676790 | [T/C] | - | Intergenic | - |

| SNP IDs | Chromosomes | Physical positions (bp) | SNPs | Gene accession IDs | Sequence components of genes | Putative functions |
|---|---|---|---|---|---|---|
| CWSNP5953 | Ca-Kabuli-Chr7 | 767708 | [G/A] | Ca03402 | Non-Synonymous-CDS | EPS15 homology (EH) |
| CWSNP5954 | Ca-Kabuli-Chr7 | 781409 | [A/G] | - | DRR | - |
| CWSNP5955 | Ca-Kabuli-Chr7 | 807110 | [T/C] | Ca03397 | Intron | Phosphofructokinase domain |
| CWSNP5956 | Ca-Kabuli-Chr7 | 815526 | [C/A] | Ca03396 | Non-Synonymous-CDS | - |
| CWSNP5957 | Ca-Kabuli-Chr7 | 838581 | [C/A] | - | URR | - |
| CWSNP5958 | Ca-Kabuli-Chr7 | 838651 | [A/C] | - | URR | - |
| CWSNP5959 | Ca-Kabuli-Chr7 | 841988 | [T/C] | Ca03393 | Intron | Domain of unknown function DUF221 |
| CWSNP5960 | Ca-Kabuli-Chr7 | 975236 | [T/A] | Ca03381 | Intron | Multi copper oxidase, type1 |
| CWSNP5961 | Ca-Kabuli-Chr7 | 1096379 | [C/T] | Ca03366 | Synonymous-CDS | IQ motif, EF-hand binding site |
| CWSNP5962 | Ca-Kabuli-Chr7 | 1153267 | [A/C] | - | URR | - |
| CWSNP5963 | Ca-Kabuli-Chr7 | 1293601 | [C/G] | Ca03339 | Synonymous-CDS | Protein kinase, catalytic domain |
| CWSNP5964 | Ca-Kabuli-Chr7 | 1350605 | [C/T] | Ca03334 | Non-Synonymous-CDS | Peptidase C19,ubiquitin carboxyl-terminalhydrolase 2 |
| CWSNP5965 | Ca-Kabuli-Chr7 | 1414206 | [C/A] | - | Intergenic | - |
| CWSNP5966 | Ca-Kabuli-Chr7 | 1511252 | [C/T] | Ca03315 | Intron | Pentatrico peptide repeat |
| CWSNP5967 | Ca-Kabuli-Chr7 | 1585622 | [T/C] | - | Intergenic | - |
| CWSNP5968 | Ca-Kabuli-Chr7 | 1704780 | [T/G] | - | URR | - |

| SNP IDs | Chromosomes | Physical positions (bp) | SNPs | Gene accession IDs | Sequence components of genes | Putative functions |
|---|---|---|---|---|---|---|
| CWSNP5969 | Ca-Kabuli-Chr7 | 1705197 | [A/C] | Ca03296 | Non-Synonymous-CDS | Cytochromecoxidase,subunitVIb |
| CWSNP5970 | Ca-Kabuli-Chr7 | 1707283 | [G/T] | Ca03296 | Non-Synonymous-CDS | Cytochromecoxidase,subunitVIb |
| CWSNP5971 | Ca-Kabuli-Chr7 | 2103626 | [C/T] | Ca03262 | Intron | Calmodulin binding protein-like |
| CWSNP5972 | Ca-Kabuli-Chr7 | 2609831 | [C/A] | - | DRR | - |
| CWSNP5973 | Ca-Kabuli-Chr7 | 2684213 | [A/G] | Ca03210 | Intron | LisHdimerisationmotif |
| CWSNP5974 | Ca-Kabuli-Chr7 | 2734680 | [A/G] | Ca03205 | Intron | Protein kinase, catalytic domain |
| CWSNP5975 | Ca-Kabuli-Chr7 | 2745665 | [A/C] | - | Intergenic | - |
| CWSNP5976 | Ca-Kabuli-Chr7 | 2745656 | [A/C] | - | Intergenic | - |
| CWSNP5977 | Ca-Kabuli-Chr7 | 2757255 | [G/C] | Ca03202 | Non-Synonymous-CDS | Transcription factor jumonji/aspartyl beta-hydroxylase |
| CWSNP5978 | Ca-Kabuli-Chr7 | 2757253 | [T/G] | Ca03202 | Non-Synonymous-CDS | Transcription factor jumonji/aspartyl beta-hydroxylase |
| CWSNP5979 | Ca-Kabuli-Chr7 | 2762396 | [G/A] | - | Intergenic | - |
| CWSNP5980 | Ca-Kabuli-Chr7 | 2832369 | [C/T] | Ca03193 | Synonymous-CDS | Zinc finger,GATA-type |
| CWSNP5981 | Ca-Kabuli-Chr7 | 2857630 | [T/G] | Ca03190 | Intron | Protein kinase, catalytic domain |
| CWSNP5982 | Ca-Kabuli-Chr7 | 2915375 | [C/G] | - | Intergenic | - |
| CWSNP5983 | Ca-Kabuli-Chr7 | 3023154 | [T/C] | Ca03170 | Intron | - |
| CWSNP5984 | Ca-Kabuli-Chr7 | 3070531 | [A/C] | - | DRR | - |

| SNP IDs | Chromosomes | Physical positions (bp) | SNPs | Gene accession IDs | Sequence components of genes | Putative functions |
|---|---|---|---|---|---|---|
| CWSNP5985 | Ca-Kabuli-Chr7 | 3094087 | [A/G] | Ca03163 | Synonymous-CDS | IQ motif, EF-hand binding site |
| CWSNP5986 | Ca-Kabuli-Chr7 | 3109250 | [T/C] | - | Intergenic | - |
| CWSNP5987 | Ca-Kabuli-Chr7 | 3109352 | [T/C] | - | Intergenic | - |
| CWSNP5988 | Ca-Kabuli-Chr7 | 3130070 | [A/G] | Ca03158 | Non-Synonymous-CDS | PhotosystemIIPsbX |
| CWSNP5989 | Ca-Kabuli-Chr7 | 3130069 | [A/G] | Ca03158 | Non-Synonymous-CDS | PhotosystemIIPsbX |
| CWSNP5990 | Ca-Kabuli-Chr7 | 3143409 | [C/T] | - | URR | - |
| CWSNP5991 | Ca-Kabuli-Chr7 | 3186341 | [C/T] | - | URR | - |
| CWSNP5992 | Ca-Kabuli-Chr7 | 3186262 | [T/G] | - | URR | - |
| CWSNP5993 | Ca-Kabuli-Chr7 | 3315157 | [A/G] | - | URR | - |
| CWSNP5994 | Ca-Kabuli-Chr7 | 3356399 | [G/A] | Ca03128 | Intron | AUX/IAA protein |
| CWSNP5995 | Ca-Kabuli-Chr7 | 3357452 | [G/A] | Ca03128 | Non-Synonymous-CDS | AUX/IAA protein |
| CWSNP5996 | Ca-Kabuli-Chr7 | 3504900 | [G/A] | - | Intergenic | - |
| CWSNP5997 | Ca-Kabuli-Chr7 | 3505012 | [A/C] | - | Intergenic | - |
| CWSNP5998 | Ca-Kabuli-Chr7 | 3518048 | [G/A] | Ca03116 | Synonymous-CDS | Synaptojanin,N-terminal |
| CWSNP5999 | Ca-Kabuli-Chr7 | 3519510 | [A/G] | Ca03116 | Intron | Synaptojanin,N-terminal |
| CWSNP6000 | Ca-Kabuli-Chr7 | 3519701 | [A/C] | Ca03116 | Intron | Synaptojanin,N-terminal |

| SNP IDs | Chromosomes | Physical positions (bp) | SNPs | Gene accession IDs | Sequence components of genes | Putative functions |
|---|---|---|---|---|---|---|
| CWSNP6001 | Ca-Kabuli-Chr7 | 3521150 | [A/G] | Ca03116 | Intron | Synaptojanin,N-terminal |
| CWSNP6002 | Ca-Kabuli-Chr7 | 3521257 | [G/A] | Ca03116 | Intron | Synaptojanin,N-terminal |
| CWSNP6003 | Ca-Kabuli-Chr7 | 3521192 | [C/A] | Ca03116 | Intron | Synaptojanin,N-terminal |
| CWSNP6004 | Ca-Kabuli-Chr7 | 3536194 | [C/T] | - | URR | - |
| CWSNP6005 | Ca-Kabuli-Chr7 | 3536233 | [T/C] | - | URR | - |
| CWSNP6006 | Ca-Kabuli-Chr7 | 3543765 | [A/G] | - | URR | - |
| CWSNP6007 | Ca-Kabuli-Chr7 | 3543768 | [T/C] | - | URR | - |
| CWSNP6008 | Ca-Kabuli-Chr7 | 3557570 | [C/T] | Ca03113 | Intron | Zinc finger,FYVE-type |
| CWSNP6009 | Ca-Kabuli-Chr7 | 3560182 | [G/A] | Ca03113 | Non-Synonymous-CDS | Zinc finger,FYVE-type |
| CWSNP6010 | Ca-Kabuli-Chr7 | 3572963 | [T/C] | Ca03112 | Intron | Sec34-like protein |
| CWSNP6011 | Ca-Kabuli-Chr7 | 3581123 | [G/A] | Ca03112 | Intron | Sec34-like protein |
| CWSNP6012 | Ca-Kabuli-Chr7 | 3600826 | [C/T] | - | DRR | - |
| CWSNP6013 | Ca-Kabuli-Chr7 | 3600833 | [T/G] | - | DRR | - |
| CWSNP6014 | Ca-Kabuli-Chr7 | 3600866 | [T/C] | - | DRR | - |
| CWSNP6015 | Ca-Kabuli-Chr7 | 3600867 | [C/T] | - | DRR | - |
| CWSNP6016 | Ca-Kabuli-Chr7 | 3600987 | [A/C] | - | DRR | - |

| SNP IDs | Chromosomes | Physical positions (bp) | SNPs | Gene accession IDs | Sequence components of genes | Putative functions |
|---|---|---|---|---|---|---|
| CWSNP6017 | Ca-Kabuli-Chr7 | 3628086 | [G/A] | Ca03106 | Intron | Saccharopine dehydrogenase/Homospermidine synthase |
| CWSNP6018 | Ca-Kabuli-Chr7 | 3635307 | [G/A] | Ca03106 | Intron | Saccharopine dehydrogenase/Homospermidine synthase |
| CWSNP6019 | Ca-Kabuli-Chr7 | 3635309 | [C/T] | Ca03106 | Intron | Saccharopine dehydrogenase/Homospermidine synthase |
| CWSNP6020 | Ca-Kabuli-Chr7 | 3635318 | [T/A] | Ca03106 | Intron | Saccharopine dehydrogenase/Homospermidine synthase |
| CWSNP6021 | Ca-Kabuli-Chr7 | 3635325 | [T/C] | Ca03106 | Intron | Saccharopine dehydrogenase/Homospermidine synthase |
| CWSNP6022 | Ca-Kabuli-Chr7 | 3635369 | [G/T] | Ca03106 | Intron | Saccharopine dehydrogenase/Homospermidine synthase |
| CWSNP6023 | Ca-Kabuli-Chr7 | 3651051 | [T/C] | Ca03103 | Intron | SEC7-like |
| CWSNP6024 | Ca-Kabuli-Chr7 | 3665651 | [G/C] | Ca03103 | Synonymous-CDS | SEC7-like |
| CWSNP6025 | Ca-Kabuli-Chr7 | 3672192 | [T/C] | Ca03102 | Intron | Zinc finger,FYVE-type |
| CWSNP6026 | Ca-Kabuli-Chr7 | 3672236 | [G/A] | Ca03102 | Intron | Zinc finger,FYVE-type |
| CWSNP6027 | Ca-Kabuli-Chr7 | 3796826 | [A/G] | Ca03085 | Intron | SNF2-related |
| CWSNP6028 | Ca-Kabuli-Chr7 | 3802188 | [T/A] | - | URR | - |
| CWSNP6029 | Ca-Kabuli-Chr7 | 3817733 | [T/G] | Ca03082 | Non-Synonymous-CDS | ABC transporter, transmembrane domain |
| CWSNP6030 | Ca-Kabuli-Chr7 | 3817740 | [G/T] | Ca03082 | Non-Synonymous-CDS | ABC transporter, transmembrane domain |
| CWSNP6031 | Ca-Kabuli-Chr7 | 3916849 | [A/C] | - | URR | - |
| CWSNP6032 | Ca-Kabuli-Chr7 | 4017780 | [C/T] | Ca03061 | Intron | - |

| SNP IDs | Chromosomes | Physical positions (bp) | SNPs | Gene accession IDs | Sequence components of genes | Putative functions |
|---|---|---|---|---|---|---|
| CWSNP6033 | Ca-Kabuli-Chr7 | 4017851 | [T/C] | Ca03061 | Intron | - |
| CWSNP6034 | Ca-Kabuli-Chr7 | 4095293 | [A/G] | Ca03057 | Non-Synonymous-CDS | Galectin,carbohydraterecognitiondomain |
| CWSNP6035 | Ca-Kabuli-Chr7 | 4194809 | [T/A] | - | Intergenic | - |
| CWSNP6036 | Ca-Kabuli-Chr7 | 4194890 | [C/A] | - | Intergenic | - |
| CWSNP6037 | Ca-Kabuli-Chr7 | 4194891 | [C/G] | - | Intergenic | - |
| CWSNP6038 | Ca-Kabuli-Chr7 | 4198812 | [A/C] | - | Intergenic | - |
| CWSNP6039 | Ca-Kabuli-Chr7 | 4209425 | [C/T] | - | URR | - |
| CWSNP6040 | Ca-Kabuli-Chr7 | 4209465 | [A/C] | - | URR | - |
| CWSNP6041 | Ca-Kabuli-Chr7 | 4300650 | [T/C] | - | Intergenic | - |
| CWSNP6042 | Ca-Kabuli-Chr7 | 4300645 | [C/T] | - | Intergenic | - |
| CWSNP6043 | Ca-Kabuli-Chr7 | 4300592 | [G/A] | - | Intergenic | - |
| CWSNP6044 | Ca-Kabuli-Chr7 | 4300584 | [C/T] | - | Intergenic | - |
| CWSNP6045 | Ca-Kabuli-Chr7 | 4305424 | [G/T] | - | URR | - |
| CWSNP6046 | Ca-Kabuli-Chr7 | 4431138 | [A/G] | Ca03015 | Non-Synonymous-CDS | - |
| CWSNP6047 | Ca-Kabuli-Chr7 | 4461990 | [A/C] | - | Intergenic | - |
| CWSNP6048 | Ca-Kabuli-Chr7 | 4552790 | [T/C] | - | DRR | - |

| SNP IDs | Chromosomes | Physical positions (bp) | SNPs | Gene accession IDs | Sequence components of genes | Putative functions |
|---|---|---|---|---|---|---|
| CWSNP6049 | Ca-Kabuli-Chr7 | 4573236 | [G/A] | - | URR | - |
| CWSNP6050 | Ca-Kabuli-Chr7 | 4625723 | [C/T] | Ca02989 | Synonymous-CDS | Signal transduction response regulator,receiver domain |
| CWSNP6051 | Ca-Kabuli-Chr7 | 4652083 | [G/A] | Ca02988 | Intron | Protein of unknown function DUF789 |
| CWSNP6052 | Ca-Kabuli-Chr7 | 4749212 | [T/G] | - | DRR | - |
| CWSNP6053 | Ca-Kabuli-Chr7 | 4834177 | [G/T] | - | Intergenic | - |
| CWSNP6054 | Ca-Kabuli-Chr7 | 4981846 | [T/C] | - | Intergenic | - |
| CWSNP6055 | Ca-Kabuli-Chr7 | 5122536 | [T/G] | - | DRR | - |
| CWSNP6056 | Ca-Kabuli-Chr7 | 5383564 | [G/C] | - | DRR | - |
| CWSNP6057 | Ca-Kabuli-Chr7 | 5383566 | [A/T] | - | DRR | - |
| CWSNP6058 | Ca-Kabuli-Chr7 | 5383671 | [G/T] | - | DRR | - |
| CWSNP6059 | Ca-Kabuli-Chr7 | 5383695 | [T/G] | - | DRR | - |
| CWSNP6060 | Ca-Kabuli-Chr7 | 5383767 | [G/A] | - | DRR | - |
| CWSNP6061 | Ca-Kabuli-Chr7 | 5384447 | [G/C] | - | Intergenic | - |
| CWSNP6062 | Ca-Kabuli-Chr7 | 5406524 | [T/G] | - | URR | - |
| CWSNP6063 | Ca-Kabuli-Chr7 | 5464941 | [G/A] | - | Intergenic | - |
| CWSNP6064 | Ca-Kabuli-Chr7 | 5464942 | [A/G] | - | Intergenic | - |

| SNP IDs | Chromosomes | Physical positions (bp) | SNPs | Gene accession IDs | Sequence components of genes | Putative functions |
|---|---|---|---|---|---|---|
| CWSNP6065 | Ca-Kabuli-Chr7 | 5467744 | [G/C] | - | DRR | - |
| CWSNP6066 | Ca-Kabuli-Chr7 | 5508162 | [A/C] | Ca06816 | Synonymous-CDS | SNF2-related |
| CWSNP6067 | Ca-Kabuli-Chr7 | 5520720 | [T/G] | Ca06814 | Non-Synonymous-CDS | Heat shock protein DnaJ,N-terminal |
| CWSNP6068 | Ca-Kabuli-Chr7 | 5520934 | [T/C] | Ca06814 | Non-Synonymous-CDS | Heat shock protein DnaJ,N-terminal |
| CWSNP6069 | Ca-Kabuli-Chr7 | 5521596 | [T/C] | Ca06814 | Non-Synonymous-CDS | Heat shock protein DnaJ,N-terminal |
| CWSNP6070 | Ca-Kabuli-Chr7 | 5521659 | [G/T] | Ca06814 | Non-Synonymous-CDS | Heat shock protein DnaJ,N-terminal |
| CWSNP6071 | Ca-Kabuli-Chr7 | 5529577 | [T/G] | - | DRR | - |
| CWSNP6072 | Ca-Kabuli-Chr7 | 5529729 | [A/G] | - | DRR | - |
| CWSNP6073 | Ca-Kabuli-Chr7 | 5529888 | [A/G] | - | DRR | - |
| CWSNP6074 | Ca-Kabuli-Chr7 | 5529844 | [C/A] | - | DRR | - |
| CWSNP6075 | Ca-Kabuli-Chr7 | 5537851 | [G/C] | Ca06811 | Intron | ProtamineP1 |
| CWSNP6076 | Ca-Kabuli-Chr7 | 5537880 | [G/T] | Ca06811 | Intron | ProtamineP2 |
| CWSNP6077 | Ca-Kabuli-Chr7 | 5561607 | [G/T] | Ca06808 | Intron | Inosine/uridine-preferring nucleosidehydrolase domain |
| CWSNP6078 | Ca-Kabuli-Chr7 | 5568114 | [C/A] | - | Intergenic | - |
| CWSNP6079 | Ca-Kabuli-Chr7 | 5577467 | [G/T] | Ca06805 | Synonymous-CDS | - |
| CWSNP6080 | Ca-Kabuli-Chr7 | 5577439 | [A/G] | Ca06805 | Non-Synonymous-CDS | - |

| SNP IDs | Chromosomes | Physical positions (bp) | SNPs | Gene accession IDs | Sequence components of genes | Putative functions |
|---|---|---|---|---|---|---|
| CWSNP6081 | Ca-Kabuli-Chr7 | 5581968 | [G/C] | - | DRR | - |
| CWSNP6082 | Ca-Kabuli-Chr7 | 5658445 | [G/T] | - | DRR | - |
| CWSNP6083 | Ca-Kabuli-Chr7 | 5671902 | [G/T] | - | Intergenic | - |
| CWSNP6084 | Ca-Kabuli-Chr7 | 5672156 | [T/C] | - | Intergenic | - |
| CWSNP6085 | Ca-Kabuli-Chr7 | 5683412 | [T/A] | Ca06789 | Intron | RNA polymerase I specific transcription initiation factor RRN3 |
| CWSNP6086 | Ca-Kabuli-Chr7 | 5713977 | [T/C] | - | Intergenic | - |
| CWSNP6087 | Ca-Kabuli-Chr7 | 5737305 | [G/A] | Ca06783 | Synonymous-CDS | Protein kinase, catalytic domain |
| CWSNP6088 | Ca-Kabuli-Chr7 | 5756269 | [C/T] | - | Intergenic | - |
| CWSNP6089 | Ca-Kabuli-Chr7 | 5756832 | [A/C] | - | Intergenic | - |
| CWSNP6090 | Ca-Kabuli-Chr7 | 5774218 | [T/A] | Ca06781 | Intron | Dienelactonehydrolase |
| CWSNP6091 | Ca-Kabuli-Chr7 | 5774219 | [T/A] | Ca06781 | Intron | Dienelactonehydrolase |
| CWSNP6092 | Ca-Kabuli-Chr7 | 5774220 | [A/T] | Ca06781 | Intron | Dienelactonehydrolase |
| CWSNP6093 | Ca-Kabuli-Chr7 | 5774221 | [A/T] | Ca06781 | Intron | Dienelactonehydrolase |
| CWSNP6094 | Ca-Kabuli-Chr7 | 5774230 | [G/A] | Ca06781 | Intron | Dienelactonehydrolase |
| CWSNP6095 | Ca-Kabuli-Chr7 | 5774257 | [C/T] | Ca06781 | Intron | Dienelactonehydrolase |
| CWSNP6096 | Ca-Kabuli-Chr7 | 5774310 | [A/G] | Ca06781 | Intron | Dienelactonehydrolase |

| SNP IDs | Chromosomes | Physical positions (bp) | SNPs | Gene accession IDs | Sequence components of genes | Putative functions |
|---|---|---|---|---|---|---|
| CWSNP6097 | Ca-Kabuli-Chr7 | 5811097 | [G/A] | - | Intergenic | - |
| CWSNP6098 | Ca-Kabuli-Chr7 | 5836421 | [A/T] | - | URR | - |
| CWSNP6099 | Ca-Kabuli-Chr7 | 5846477 | [A/G] | - | DRR | - |
| CWSNP6100 | Ca-Kabuli-Chr7 | 5849936 | [C/T] | - | Intergenic | - |
| CWSNP6101 | Ca-Kabuli-Chr7 | 5849904 | [C/A] | - | Intergenic | - |
| CWSNP6102 | Ca-Kabuli-Chr7 | 5850058 | [T/C] | - | Intergenic | - |
| CWSNP6103 | Ca-Kabuli-Chr7 | 5854598 | [C/T] | - | DRR | - |
| CWSNP6104 | Ca-Kabuli-Chr7 | 5929009 | [C/G] | Ca06764 | Intron | ATPase ,P-type, H+transporting proton pump |
| CWSNP6105 | Ca-Kabuli-Chr7 | 5946113 | [G/A] | - | DRR | - |
| CWSNP6106 | Ca-Kabuli-Chr7 | 5950352 | [T/A] | Ca06762 | Intron | DNA-dependent ATPase MCM |
| CWSNP6107 | Ca-Kabuli-Chr7 | 5950492 | [T/C] | Ca06762 | Synonymous-CDS | DNA-dependent ATPase MCM |
| CWSNP6108 | Ca-Kabuli-Chr7 | 5957696 | [T/C] | Ca06761 | Intron | RibonucleaseIII |
| CWSNP6109 | Ca-Kabuli-Chr7 | 5963611 | [C/G] | Ca06761 | Intron | RibonucleaseIII |
| CWSNP6110 | Ca-Kabuli-Chr7 | 5963609 | [C/A] | Ca06761 | Intron | RibonucleaseIII |
| CWSNP6111 | Ca-Kabuli-Chr7 | 6012561 | [A/C] | - | DRR | - |
| CWSNP6112 | Ca-Kabuli-Chr7 | 6012611 | [A/C] | - | DRR | - |

| SNP IDs | Chromosomes | Physical positions (bp) | SNPs | Gene accession IDs | Sequence components of genes | Putative functions |
|---|---|---|---|---|---|---|
| CWSNP6113 | Ca-Kabuli-Chr7 | 6029990 | [C/G] | - | URR | - |
| CWSNP6114 | Ca-Kabuli-Chr7 | 6066322 | [G/A] | - | DRR | - |
| CWSNP6115 | Ca-Kabuli-Chr7 | 6074408 | [T/A] | Ca06752 | Synonymous-CDS | Phox/Bem1p |
| CWSNP6116 | Ca-Kabuli-Chr7 | 6075124 | [T/G] | Ca06752 | Non-Synonymous-CDS | Phox/Bem1p |
| CWSNP6117 | Ca-Kabuli-Chr7 | 6075218 | [C/G] | Ca06752 | Non-Synonymous-CDS | Phox/Bem1p |
| CWSNP6118 | Ca-Kabuli-Chr7 | 6096238 | [C/A] | - | DRR | - |
| CWSNP6119 | Ca-Kabuli-Chr7 | 6110019 | [T/C] | - | DRR | - |
| CWSNP6120 | Ca-Kabuli-Chr7 | 6132021 | [G/A] | Ca06744 | Intron | Ubiquitin |
| CWSNP6121 | Ca-Kabuli-Chr7 | 6232777 | [T/C] | - | Intergenic | - |
| CWSNP6122 | Ca-Kabuli-Chr7 | 6299248 | [A/G] | - | URR | - |
| CWSNP6123 | Ca-Kabuli-Chr7 | 6299249 | [C/G] | - | URR | - |
| CWSNP6124 | Ca-Kabuli-Chr7 | 6393366 | [G/A] | Ca06712 | Intron | Metallo-dependent phosphatase |
| CWSNP6125 | Ca-Kabuli-Chr7 | 6467903 | [C/T] | Ca06705 | Synonymous-CDS | Mak10 subunit,NatCN(alpha)-terminal acetyltransferase |
| CWSNP6126 | Ca-Kabuli-Chr7 | 6540624 | [A/G] | Ca06700 | Intron | Rap1-interactingfactor1N-terminal |
| CWSNP6127 | Ca-Kabuli-Chr7 | 6540602 | [G/A] | Ca06700 | Intron | Rap1-interactingfactor1N-terminal |
| CWSNP6128 | Ca-Kabuli-Chr7 | 6540601 | [G/C] | Ca06700 | Intron | Rap1-interactingfactor1N-terminal |

| SNP IDs | Chromosomes | Physical positions (bp) | SNPs | Gene accession IDs | Sequence components of genes | Putative functions |
|---|---|---|---|---|---|---|
| CWSNP6129 | Ca-Kabuli-Chr7 | 6566322 | [T/C] | - | Intergenic | - |
| CWSNP6130 | Ca-Kabuli-Chr7 | 6682454 | [A/G] | - | Intergenic | - |
| CWSNP6131 | Ca-Kabuli-Chr7 | 6682453 | [G/T] | - | Intergenic | - |
| CWSNP6132 | Ca-Kabuli-Chr7 | 6682448 | [T/C] | - | Intergenic | - |
| CWSNP6133 | Ca-Kabuli-Chr7 | 6682443 | [C/T] | - | Intergenic | - |
| CWSNP6134 | Ca-Kabuli-Chr7 | 6682439 | [T/A] | - | Intergenic | - |
| CWSNP6135 | Ca-Kabuli-Chr7 | 6682455 | [G/T] | - | Intergenic | - |
| CWSNP6136 | Ca-Kabuli-Chr7 | 6703849 | [G/T] | Ca06691 | Non-Synonymous-CDS | WD40 repeat |
| CWSNP6137 | Ca-Kabuli-Chr7 | 6792606 | [T/G] | - | Intergenic | - |
| CWSNP6138 | Ca-Kabuli-Chr7 | 6859511 | [C/G] | - | Intergenic | - |
| CWSNP6139 | Ca-Kabuli-Chr7 | 7026516 | [T/C] | - | DRR | - |
| CWSNP6140 | Ca-Kabuli-Chr7 | 7146159 | [C/G] | - | Intergenic | - |
| CWSNP6141 | Ca-Kabuli-Chr7 | 7265386 | [T/A] | - | Intergenic | - |
| CWSNP6142 | Ca-Kabuli-Chr7 | 7423419 | [G/A] | Ca06617 | Non-Synonymous-CDS | Heat shock protein DnaJ,N-terminal |
| CWSNP6143 | Ca-Kabuli-Chr7 | 7576668 | [A/C] | - | Intergenic | - |
| CWSNP6144 | Ca-Kabuli-Chr7 | 7580158 | [T/C] | - | Intergenic | - |

| SNP IDs | Chromosomes | Physical positions (bp) | SNPs | Gene accession IDs | Sequence components of genes | Putative functions |
|---|---|---|---|---|---|---|
| CWSNP6145 | Ca-Kabuli-Chr7 | 7706816 | [A/G] | - | Intergenic | - |
| CWSNP6146 | Ca-Kabuli-Chr7 | 7756455 | [C/T] | Ca13243 | Non-Synonymous-CDS | SNF2-related |
| CWSNP6147 | Ca-Kabuli-Chr7 | 7756566 | [G/A] | Ca13243 | Intron | SNF2-related |
| CWSNP6148 | Ca-Kabuli-Chr7 | 7809179 | [T/G] | Ca13237 | Intron | PeptidaseS49 |
| CWSNP6149 | Ca-Kabuli-Chr7 | 7809192 | [A/C] | Ca13237 | Intron | PeptidaseS49 |
| CWSNP6150 | Ca-Kabuli-Chr7 | 8061808 | [A/G] | Ca13209 | Synonymous-CDS | Nucleic acid binding,OB-fold,tRNA/helicase-type |
| CWSNP6151 | Ca-Kabuli-Chr7 | 8169947 | [T/C] | - | URR | - |
| CWSNP6152 | Ca-Kabuli-Chr7 | 8187181 | [G/A] | Ca13195 | Synonymous-CDS | Chaperonin ClpA/B |
| CWSNP6153 | Ca-Kabuli-Chr7 | 8188004 | [C/G] | Ca13195 | Non-Synonymous-CDS | Chaperonin ClpA/B |
| CWSNP6154 | Ca-Kabuli-Chr7 | 8261062 | [C/A] | - | DRR | - |
| CWSNP6155 | Ca-Kabuli-Chr7 | 8569632 | [A/G] | - | URR | - |
| CWSNP6156 | Ca-Kabuli-Chr7 | 8583890 | [T/C] | Ca15359 | Synonymous-CDS | Zinc finger,C2HC5-type |
| CWSNP6157 | Ca-Kabuli-Chr7 | 8626875 | [A/T] | - | URR | - |
| CWSNP6158 | Ca-Kabuli-Chr7 | 8835601 | [G/A] | - | DRR | - |
| CWSNP6159 | Ca-Kabuli-Chr7 | 8835602 | [A/G] | - | DRR | - |
| CWSNP6160 | Ca-Kabuli-Chr7 | 8835610 | [G/A] | - | DRR | - |

| SNP IDs | Chromosomes | Physical positions (bp) | SNPs | Gene accession IDs | Sequence components of genes | Putative functions |
|---|---|---|---|---|---|---|
| CWSNP6161 | Ca-Kabuli-Chr7 | 8835659 | [G/T] | - | DRR | - |
| CWSNP6162 | Ca-Kabuli-Chr7 | 8835757 | [T/C] | - | DRR | - |
| CWSNP6163 | Ca-Kabuli-Chr7 | 8835718 | [T/G] | - | DRR | - |
| CWSNP6164 | Ca-Kabuli-Chr7 | 8835705 | [T/A] | - | DRR | - |
| CWSNP6165 | Ca-Kabuli-Chr7 | 9128044 | [A/G] | - | Intergenic | - |
| CWSNP6166 | Ca-Kabuli-Chr7 | 9128050 | [C/A] | - | Intergenic | - |
| CWSNP6167 | Ca-Kabuli-Chr7 | 9147367 | [A/T] | Ca15301 | Intron | Transcription factor E2F/dimerisationpartner (TDP) |
| CWSNP6168 | Ca-Kabuli-Chr7 | 9147366 | [T/G] | Ca15301 | Intron | Transcription factor E2F/dimerisationpartner (TDP) |
| CWSNP6169 | Ca-Kabuli-Chr7 | 9147502 | [C/T] | Ca15301 | Intron | Transcription factor E2F/dimerisationpartner (TDP) |
| CWSNP6170 | Ca-Kabuli-Chr7 | 9151709 | [T/G] | - | DRR | - |
| CWSNP6171 | Ca-Kabuli-Chr7 | 9151755 | [C/T] | - | DRR | - |
| CWSNP6172 | Ca-Kabuli-Chr7 | 9153104 | [C/A] | - | DRR | - |
| CWSNP6173 | Ca-Kabuli-Chr7 | 9153062 | [G/A] | - | DRR | - |
| CWSNP6174 | Ca-Kabuli-Chr7 | 9214457 | [T/A] | - | Intergenic | - |
| CWSNP6175 | Ca-Kabuli-Chr7 | 9214422 | [C/T] | - | Intergenic | - |
| CWSNP6176 | Ca-Kabuli-Chr7 | 9301825 | [G/C] | Ca17330 | Non-Synonymous-CDS | rRNA small subunit methyltransferase G |

| SNP IDs | Chromosomes | Physical positions (bp) | SNPs | Gene accession IDs | Sequence components of genes | Putative functions |
|---|---|---|---|---|---|---|
| CWSNP6177 | Ca-Kabuli-Chr7 | 9301881 | [C/T] | - | Intergenic | - |
| CWSNP6178 | Ca-Kabuli-Chr7 | 9348281 | [T/C] | - | DRR | - |
| CWSNP6179 | Ca-Kabuli-Chr7 | 9350506 | [T/G] | - | DRR | - |
| CWSNP6180 | Ca-Kabuli-Chr7 | 9350535 | [A/C] | - | DRR | - |
| CWSNP6181 | Ca-Kabuli-Chr7 | 9351783 | [C/G] | Ca17325 | Intron | Glycosidehydrolase,family47 |
| CWSNP6182 | Ca-Kabuli-Chr7 | 9351863 | [G/A] | Ca17325 | Non-Synonymous-CDS | Glycosidehydrolase,family47 |
| CWSNP6183 | Ca-Kabuli-Chr7 | 9475275 | [T/G] | - | DRR | - |
| CWSNP6184 | Ca-Kabuli-Chr7 | 9523902 | [C/T] | - | URR | - |
| CWSNP6185 | Ca-Kabuli-Chr7 | 9612558 | [A/C] | - | DRR | - |
| CWSNP6186 | Ca-Kabuli-Chr7 | 9671210 | [T/C] | - | DRR | - |
| CWSNP6187 | Ca-Kabuli-Chr7 | 9696620 | [A/G] | - | Intergenic | - |
| CWSNP6188 | Ca-Kabuli-Chr7 | 9744436 | [G/T] | - | Intergenic | - |
| CWSNP6189 | Ca-Kabuli-Chr7 | 9897698 | [T/G] | - | DRR | - |
| CWSNP6190 | Ca-Kabuli-Chr7 | 9914751 | [G/A] | - | Intergenic | - |
| CWSNP6191 | Ca-Kabuli-Chr7 | 9914764 | [G/A] | - | Intergenic | - |
| CWSNP6192 | Ca-Kabuli-Chr7 | 9914785 | [T/C] | - | Intergenic | - |

| SNP IDs | Chromosomes | Physical positions (bp) | SNPs | Gene accession IDs | Sequence components of genes | Putative functions |
|---|---|---|---|---|---|---|
| CWSNP6193 | Ca-Kabuli-Chr7 | 9914794 | [G/A] | - | Intergenic | - |
| CWSNP6194 | Ca-Kabuli-Chr7 | 9914745 | [T/C] | - | Intergenic | - |
| CWSNP6195 | Ca-Kabuli-Chr7 | 9914802 | [T/G] | - | Intergenic | - |
| CWSNP6196 | Ca-Kabuli-Chr7 | 10208029 | [T/C] | - | Intergenic | - |
| CWSNP6197 | Ca-Kabuli-Chr7 | 10223068 | [G/T] | - | URR | - |
| CWSNP6198 | Ca-Kabuli-Chr7 | 10223069 | [G/T] | - | URR | - |
| CWSNP6199 | Ca-Kabuli-Chr7 | 10223071 | [T/A] | - | URR | - |
| CWSNP6200 | Ca-Kabuli-Chr7 | 10223074 | [G/T] | - | URR | - |
| CWSNP6201 | Ca-Kabuli-Chr7 | 10223075 | [C/T] | - | URR | - |
| CWSNP6202 | Ca-Kabuli-Chr7 | 10322726 | [T/C] | Ca12794 | Synonymous-CDS | tRNA/rRNAmethyltransferase,SpoU |
| CWSNP6203 | Ca-Kabuli-Chr7 | 10435983 | [C/A] | Ca12803 | Non-Synonymous-CDS | CaseinkinaseII,regulatory subunit |
| CWSNP6204 | Ca-Kabuli-Chr7 | 10484200 | [G/A] | Ca12810 | Intron | Protein of unknown function DUF702 |
| CWSNP6205 | Ca-Kabuli-Chr7 | 10521449 | [C/A] | Ca12811 | Non-Synonymous-CDS | Zinc finger,RING-type |
| CWSNP6206 | Ca-Kabuli-Chr7 | 10688189 | [T/G] | Ca12825 | Intron | Signal transduction response regulator,receiver domain |
| CWSNP6207 | Ca-Kabuli-Chr7 | 10703569 | [A/T] | - | Intergenic | - |
| CWSNP6208 | Ca-Kabuli-Chr7 | 10703704 | [G/A] | - | Intergenic | - |

| SNP IDs | Chromosomes | Physical positions (bp) | SNPs | Gene accession IDs | Sequence components of genes | Putative functions |
|---|---|---|---|---|---|---|
| CWSNP6209 | Ca-Kabuli-Chr7 | 10712231 | [G/A] | - | Intergenic | - |
| CWSNP6210 | Ca-Kabuli-Chr7 | 10775881 | [C/T] | - | Intergenic | - |
| CWSNP6211 | Ca-Kabuli-Chr7 | 10775860 | [A/G] | - | Intergenic | - |
| CWSNP6212 | Ca-Kabuli-Chr7 | 10780680 | [C/G] | - | Intergenic | - |
| CWSNP6213 | Ca-Kabuli-Chr7 | 10780682 | [G/A] | - | Intergenic | - |
| CWSNP6214 | Ca-Kabuli-Chr7 | 10780686 | [T/C] | - | Intergenic | - |
| CWSNP6215 | Ca-Kabuli-Chr7 | 10797204 | [T/C] | Ca12832 | Intron | Protein kinase, catalytic domain |
| CWSNP6216 | Ca-Kabuli-Chr7 | 10816523 | [T/G] | - | DRR | - |
| CWSNP6217 | Ca-Kabuli-Chr7 | 10900910 | [T/C] | - | DRR | - |
| CWSNP6218 | Ca-Kabuli-Chr7 | 11105847 | [C/G] | Ca09376 | Non-Synonymous-CDS | Hexokinase |
| CWSNP6219 | Ca-Kabuli-Chr7 | 11192703 | [A/C] | Ca09371 | Synonymous-CDS | Homeobox |
| CWSNP6220 | Ca-Kabuli-Chr7 | 11261924 | [G/A] | - | URR | - |
| CWSNP6221 | Ca-Kabuli-Chr7 | 11393795 | [A/G] | - | Intergenic | - |
| CWSNP6222 | Ca-Kabuli-Chr7 | 11545846 | [A/G] | Ca09342 | Non-Synonymous-CDS | Lateral organ boundaries, LOB |
| CWSNP6223 | Ca-Kabuli-Chr7 | 11568963 | [T/A] | - | Intergenic | - |
| CWSNP6224 | Ca-Kabuli-Chr7 | 11630509 | [A/G] | - | URR | - |

| SNP IDs | Chromosomes | Physical positions (bp) | SNPs | Gene accession IDs | Sequence components of genes | Putative functions |
|---|---|---|---|---|---|---|
| CWSNP6225 | Ca-Kabuli-Chr7 | 11735368 | [G/C] | - | URR | - |
| CWSNP6226 | Ca-Kabuli-Chr7 | 11803091 | [T/G] | - | Intergenic | - |
| CWSNP6227 | Ca-Kabuli-Chr7 | 11858425 | [C/T] | - | DRR | - |
| CWSNP6228 | Ca-Kabuli-Chr7 | 11886954 | [A/G] | - | Intergenic | - |
| CWSNP6229 | Ca-Kabuli-Chr7 | 12010432 | [G/A] | - | Intergenic | - |
| CWSNP6230 | Ca-Kabuli-Chr7 | 12010471 | [C/T] | - | Intergenic | - |
| CWSNP6231 | Ca-Kabuli-Chr7 | 12010504 | [G/A] | - | Intergenic | - |
| CWSNP6232 | Ca-Kabuli-Chr7 | 12010499 | [C/T] | - | Intergenic | - |
| CWSNP6233 | Ca-Kabuli-Chr7 | 12010494 | [G/A] | - | Intergenic | - |
| CWSNP6234 | Ca-Kabuli-Chr7 | 12010491 | [G/T] | - | Intergenic | - |
| CWSNP6235 | Ca-Kabuli-Chr7 | 12010483 | [C/T] | - | Intergenic | - |
| CWSNP6236 | Ca-Kabuli-Chr7 | 12010457 | [C/T] | - | Intergenic | - |
| CWSNP6237 | Ca-Kabuli-Chr7 | 12010489 | [C/T] | - | Intergenic | - |
| CWSNP6238 | Ca-Kabuli-Chr7 | 12030080 | [A/C] | Ca09301 | Synonymous-CDS | tRNA-dihydrouridine synthase |
| CWSNP6239 | Ca-Kabuli-Chr7 | 12071764 | [T/C] | Ca09297 | Intron | Kinesin , motor domain |
| CWSNP6240 | Ca-Kabuli-Chr7 | 12244259 | [G/A] | Ca09285 | Intron | Nicotinatephosphoribosyltransferase-related |

| SNP IDs | Chromosomes | Physical positions (bp) | SNPs | Gene accession IDs | Sequence components of genes | Putative functions |
|---|---|---|---|---|---|---|
| CWSNP6241 | Ca-Kabuli-Chr7 | 12390774 | [G/A] | Ca09275 | Synonymous-CDS | - |
| CWSNP6242 | Ca-Kabuli-Chr7 | 12481556 | [G/C] | - | DRR | - |
| CWSNP6243 | Ca-Kabuli-Chr7 | 12482953 | [A/C] | - | DRR | - |
| CWSNP6244 | Ca-Kabuli-Chr7 | 12483290 | [A/C] | - | DRR | - |
| CWSNP6245 | Ca-Kabuli-Chr7 | 12718820 | [C/G] | - | Intergenic | - |
| CWSNP6246 | Ca-Kabuli-Chr7 | 12718853 | [G/A] | - | Intergenic | - |
| CWSNP6247 | Ca-Kabuli-Chr7 | 12730600 | [C/A] | - | DRR | - |
| CWSNP6248 | Ca-Kabuli-Chr7 | 12737516 | [T/C] | - | DRR | - |
| CWSNP6249 | Ca-Kabuli-Chr7 | 12893655 | [C/T] | - | DRR | - |
| CWSNP6250 | Ca-Kabuli-Chr7 | 13059710 | [C/A] | Ca09222 | Non-Synonymous-CDS | BTB/POZ-like |
| CWSNP6251 | Ca-Kabuli-Chr7 | 13059712 | [C/A] | Ca09222 | Non-Synonymous-CDS | BTB/POZ-like |
| CWSNP6252 | Ca-Kabuli-Chr7 | 13059852 | [G/A] | Ca09222 | Synonymous-CDS | BTB/POZ-like |
| CWSNP6253 | Ca-Kabuli-Chr7 | 13514074 | [C/T] | Ca16037 | Synonymous-CDS | Protein of unknown function DUF677 |
| CWSNP6254 | Ca-Kabuli-Chr7 | 13605200 | [A/C] | Ca16044 | Synonymous-CDS | Zinc finger,C2H2-type |
| CWSNP6255 | Ca-Kabuli-Chr7 | 13605191 | [A/C] | Ca16044 | Synonymous-CDS | Zinc finger,C2H2-type |
| CWSNP6256 | Ca-Kabuli-Chr7 | 13605188 | [A/C] | Ca16044 | Synonymous-CDS | Zinc finger,C2H2-type |

| SNP IDs | Chromosomes | Physical positions (bp) | SNPs | Gene accession IDs | Sequence components of genes | Putative functions |
|---|---|---|---|---|---|---|
| CWSNP6257 | Ca-Kabuli-Chr7 | 13605578 | [C/G] | Ca16044 | Synonymous-CDS | Zinc finger,C2H2-type |
| CWSNP6258 | Ca-Kabuli-Chr7 | 13903980 | [T/G] | Ca16064 | Non-Synonymous-CDS | Tubby,C-terminal |
| CWSNP6259 | Ca-Kabuli-Chr7 | 13949259 | [A/G] | Ca16067 | Non-Synonymous-CDS | Heat shock protein DnaJ,N-terminal |
| CWSNP6260 | Ca-Kabuli-Chr7 | 14051146 | [G/T] | Ca16074 | Intron | - |
| CWSNP6261 | Ca-Kabuli-Chr7 | 14281993 | [G/T] | Ca23042 | Non-Synonymous-CDS | Protein of unknown function DUF702 |
| CWSNP6262 | Ca-Kabuli-Chr7 | 14328649 | [T/C] | - | Intergenic | - |
| CWSNP6263 | Ca-Kabuli-Chr7 | 14329126 | [C/T] | - | Intergenic | - |
| CWSNP6264 | Ca-Kabuli-Chr7 | 14329175 | [G/A] | - | Intergenic | - |
| CWSNP6265 | Ca-Kabuli-Chr7 | 14329196 | [G/A] | - | Intergenic | - |
| CWSNP6266 | Ca-Kabuli-Chr7 | 14329217 | [C/T] | - | Intergenic | - |
| CWSNP6267 | Ca-Kabuli-Chr7 | 14329167 | [A/T] | - | Intergenic | - |
| CWSNP6268 | Ca-Kabuli-Chr7 | 14345799 | [T/A] | Ca23045 | Non-Synonymous-CDS | D-galactoside/L-rhamnose binding SUEL lectin domain |
| CWSNP6269 | Ca-Kabuli-Chr7 | 14470549 | [C/G] | Ca09992 | Non-Synonymous-CDS | Forkhead-associated (FHA) domain |
| CWSNP6270 | Ca-Kabuli-Chr7 | 14685011 | [G/C] | - | Intergenic | - |
| CWSNP6271 | Ca-Kabuli-Chr7 | 14685017 | [C/T] | - | Intergenic | - |
| CWSNP6272 | Ca-Kabuli-Chr7 | 14685024 | [A/G] | - | Intergenic | - |

| **SNP IDs** | **Chromosomes** | **Physical positions (bp)** | **SNPs** | **Gene accession IDs** | **Sequence components of genes** | **Putative functions** |
|---|---|---|---|---|---|---|
| CWSNP6273 | Ca-Kabuli-Chr7 | 14712518 | [C/A] | - | Intergenic | - |
| CWSNP6274 | Ca-Kabuli-Chr7 | 14712568 | [C/A] | - | Intergenic | - |
| CWSNP6275 | Ca-Kabuli-Chr7 | 14712596 | [T/C] | - | Intergenic | - |
| CWSNP6276 | Ca-Kabuli-Chr7 | 14712601 | [C/T] | - | Intergenic | - |
| CWSNP6277 | Ca-Kabuli-Chr7 | 14748257 | [T/A] | - | Intergenic | - |
| CWSNP6278 | Ca-Kabuli-Chr7 | 14997794 | [G/A] | Ca09954 | Non-Synonymous-CDS | RNA helicase, ATP-dependent, DEAD-box,conserved site |
| CWSNP6279 | Ca-Kabuli-Chr7 | 15039606 | [T/A] | - | Intergenic | - |
| CWSNP6280 | Ca-Kabuli-Chr7 | 15070091 | [T/A] | Ca09947 | Intron | Uncharacterised protein familyCys-rich |
| CWSNP6281 | Ca-Kabuli-Chr7 | 15125638 | [G/C] | Ca09942 | Non-Synonymous-CDS | PeptidaseA1 |
| CWSNP6282 | Ca-Kabuli-Chr7 | 15399421 | [T/C] | Ca09920 | Non-Synonymous-CDS | Glycosidehydrolase,family19,catalytic |
| CWSNP6283 | Ca-Kabuli-Chr7 | 15463183 | [G/T] | Ca09916 | Non-Synonymous-CDS | AMP-dependent synthetase/ligase |
| CWSNP6284 | Ca-Kabuli-Chr7 | 15463169 | [G/A] | Ca09916 | Synonymous-CDS | AMP-dependent synthetase/ligase |
| CWSNP6285 | Ca-Kabuli-Chr7 | 15530329 | [G/T] | Ca09913 | Non-Synonymous-CDS | AMP-dependent synthetase/ligase |
| CWSNP6286 | Ca-Kabuli-Chr7 | 15530315 | [G/A] | Ca09913 | Synonymous-CDS | AMP-dependent synthetase/ligase |
| CWSNP6287 | Ca-Kabuli-Chr7 | 15568060 | [G/T] | - | DRR | - |
| CWSNP6288 | Ca-Kabuli-Chr7 | 15568046 | [G/A] | - | DRR | - |

| SNP IDs | Chromosomes | Physical positions (bp) | SNPs | Gene accession IDs | Sequence components of genes | Putative functions |
|---|---|---|---|---|---|---|
| CWSNP6289 | Ca-Kabuli-Chr7 | 15826588 | [A/C] | Ca09889 | Synonymous-CDS | Lipid-bindingSTART |
| CWSNP6290 | Ca-Kabuli-Chr7 | 15966680 | [A/G] | Ca09880 | Synonymous-CDS | Sugar/inositol transporter |
| CWSNP6291 | Ca-Kabuli-Chr7 | 15966714 | [G/A] | Ca09880 | Non-Synonymous-CDS | Sugar/inositol transporter |
| CWSNP6292 | Ca-Kabuli-Chr7 | 16289794 | [G/T] | Ca09857 | Non-Synonymous-CDS | Tafazzin |
| CWSNP6293 | Ca-Kabuli-Chr7 | 16324985 | [T/C] | Ca09853 | Intron | Drug/metabolite transporter |
| CWSNP6294 | Ca-Kabuli-Chr7 | 16626047 | [T/G] | Ca20562 | Synonymous-CDS | Nodulin-like |
| CWSNP6295 | Ca-Kabuli-Chr7 | 16665577 | [A/G] | Ca20568 | Synonymous-CDS | Tetratricopeptide repeat-containing |
| CWSNP6296 | Ca-Kabuli-Chr7 | 16735437 | [G/A] | - | Intergenic | - |
| CWSNP6297 | Ca-Kabuli-Chr7 | 16821274 | [A/C] | Ca20581 | Non-Synonymous-CDS | - |
| CWSNP6298 | Ca-Kabuli-Chr7 | 16936123 | [C/G] | Ca15885 | Non-Synonymous-CDS | H/ACA ribonucleoprotein complex,subunit Nop10 |
| CWSNP6299 | Ca-Kabuli-Chr7 | 17140664 | [C/T] | - | Intergenic | - |
| CWSNP6300 | Ca-Kabuli-Chr7 | 17228904 | [T/G] | - | URR | - |
| CWSNP6301 | Ca-Kabuli-Chr7 | 17318174 | [G/T] | Ca15859 | Non-Synonymous-CDS | SET domain |
| CWSNP6302 | Ca-Kabuli-Chr7 | 17766952 | [T/A] | - | Intergenic | - |
| CWSNP6303 | Ca-Kabuli-Chr7 | 17766914 | [C/A] | - | Intergenic | - |
| CWSNP6304 | Ca-Kabuli-Chr7 | 17779414 | [A/G] | Ca15839 | Non-Synonymous-CDS | Basic-leucine zipper (bZIP) Transcription factor |

| SNP IDs | Chromosomes | Physical positions (bp) | SNPs | Gene accession IDs | Sequence components of genes | Putative functions |
|---|---|---|---|---|---|---|
| CWSNP6305 | Ca-Kabuli-Chr7 | 18011604 | [G/C] | Ca15826 | Non-Synonymous-CDS | Protein kinase, catalytic domain |
| CWSNP6306 | Ca-Kabuli-Chr7 | 18090289 | [T/A] | - | Intergenic | - |
| CWSNP6307 | Ca-Kabuli-Chr7 | 18319049 | [T/G] | - | Intergenic | - |
| CWSNP6308 | Ca-Kabuli-Chr7 | 18319073 | [T/G] | - | Intergenic | - |
| CWSNP6309 | Ca-Kabuli-Chr7 | 18365218 | [G/A] | - | Intergenic | - |
| CWSNP6310 | Ca-Kabuli-Chr7 | 18469422 | [C/T] | Ca22127 | Non-Synonymous-CDS | - |
| CWSNP6311 | Ca-Kabuli-Chr7 | 18638565 | [A/G] | Ca12302 | Non-Synonymous-CDS | - |
| CWSNP6312 | Ca-Kabuli-Chr7 | 18642538 | [A/G] | Ca12302 | Non-Synonymous-CDS | - |
| CWSNP6313 | Ca-Kabuli-Chr7 | 18658563 | [C/G] | Ca12303 | Synonymous-CDS | Phosphatetransporter |
| CWSNP6314 | Ca-Kabuli-Chr7 | 18696645 | [G/A] | - | Intergenic | - |
| CWSNP6315 | Ca-Kabuli-Chr7 | 18910468 | [A/G] | - | Intergenic | - |
| CWSNP6316 | Ca-Kabuli-Chr7 | 19042183 | [C/T] | Ca12316 | Intron | Kinesin , motor domain |
| CWSNP6317 | Ca-Kabuli-Chr7 | 19217501 | [A/C] | Ca12328 | Non-Synonymous-CDS | Pathogenesis-related transcriptional factor/ERF, DNA-binding |
| CWSNP6318 | Ca-Kabuli-Chr7 | 19472689 | [T/A] | - | DRR | - |
| CWSNP6319 | Ca-Kabuli-Chr7 | 19818789 | [C/A] | - | Intergenic | - |
| CWSNP6320 | Ca-Kabuli-Chr7 | 19818770 | [T/C] | - | Intergenic | - |

| SNP IDs | Chromosomes | Physical positions (bp) | SNPs | Gene accession IDs | Sequence components of genes | Putative functions |
|---|---|---|---|---|---|---|
| CWSNP6321 | Ca-Kabuli-Chr7 | 19818759 | [G/C] | - | Intergenic | - |
| CWSNP6322 | Ca-Kabuli-Chr7 | 19818757 | [G/C] | - | Intergenic | - |
| CWSNP6323 | Ca-Kabuli-Chr7 | 20031805 | [T/C] | - | Intergenic | - |
| CWSNP6324 | Ca-Kabuli-Chr7 | 20032205 | [A/C] | Ca12371 | Synonymous-CDS | General substrate transporter |
| CWSNP6325 | Ca-Kabuli-Chr7 | 20252133 | [T/C] | - | Intergenic | - |
| CWSNP6326 | Ca-Kabuli-Chr7 | 20252134 | [G/A] | - | Intergenic | - |
| CWSNP6327 | Ca-Kabuli-Chr7 | 20252135 | [A/G] | - | Intergenic | - |
| CWSNP6328 | Ca-Kabuli-Chr7 | 20252144 | [G/A] | - | Intergenic | - |
| CWSNP6329 | Ca-Kabuli-Chr7 | 20252145 | [A/G] | - | Intergenic | - |
| CWSNP6330 | Ca-Kabuli-Chr7 | 20252181 | [C/T] | - | Intergenic | - |
| CWSNP6331 | Ca-Kabuli-Chr7 | 20252192 | [G/A] | - | Intergenic | - |
| CWSNP6332 | Ca-Kabuli-Chr7 | 20252201 | [C/A] | - | Intergenic | - |
| CWSNP6333 | Ca-Kabuli-Chr7 | 20252233 | [C/T] | - | Intergenic | - |
| CWSNP6334 | Ca-Kabuli-Chr7 | 20252221 | [T/C] | - | Intergenic | - |
| CWSNP6335 | Ca-Kabuli-Chr7 | 20252309 | [C/T] | - | Intergenic | - |
| CWSNP6336 | Ca-Kabuli-Chr7 | 20252334 | [A/T] | - | Intergenic | - |

| SNP IDs | Chromosomes | Physical positions (bp) | SNPs | Gene accession IDs | Sequence components of genes | Putative functions |
|---|---|---|---|---|---|---|
| CWSNP6337 | Ca-Kabuli-Chr7 | 20252340 | [A/G] | - | Intergenic | - |
| CWSNP6338 | Ca-Kabuli-Chr7 | 20414703 | [C/A] | - | Intergenic | - |
| CWSNP6339 | Ca-Kabuli-Chr7 | 20496378 | [G/A] | Ca12400 | Synonymous-CDS | Pathogenesis-related transcriptional factor/ERF, DNA-binding |
| CWSNP6340 | Ca-Kabuli-Chr7 | 20496303 | [C/T] | Ca12400 | Synonymous-CDS | Pathogenesis-related transcriptional factor/ERF, DNA-binding |
| CWSNP6341 | Ca-Kabuli-Chr7 | 20496438 | [C/T] | Ca12400 | Synonymous-CDS | Pathogenesis-related transcriptional factor/ERF, DNA-binding |
| CWSNP6342 | Ca-Kabuli-Chr7 | 20498552 | [T/C] | Ca12400 | Intron | Pathogenesis-related transcriptional factor/ERF, DNA-binding |
| CWSNP6343 | Ca-Kabuli-Chr7 | 20498626 | [T/G] | Ca12400 | Intron | Pathogenesis-related transcriptional factor/ERF, DNA-binding |
| CWSNP6344 | Ca-Kabuli-Chr7 | 20540780 | [C/T] | - | Intergenic | - |
| CWSNP6345 | Ca-Kabuli-Chr7 | 20540791 | [G/A] | - | Intergenic | - |
| CWSNP6346 | Ca-Kabuli-Chr7 | 20543119 | [T/C] | - | DRR | - |
| CWSNP6347 | Ca-Kabuli-Chr7 | 20550563 | [G/A] | - | Intergenic | - |
| CWSNP6348 | Ca-Kabuli-Chr7 | 20550575 | [C/G] | - | Intergenic | - |
| CWSNP6349 | Ca-Kabuli-Chr7 | 20552041 | [G/A] | Ca12405 | Synonymous-CDS | SGT1 |
| CWSNP6350 | Ca-Kabuli-Chr7 | 20552053 | [C/G] | Ca12405 | Synonymous-CDS | SGT1 |
| CWSNP6351 | Ca-Kabuli-Chr7 | 20556466 | [G/C] | - | Intergenic | - |
| CWSNP6352 | Ca-Kabuli-Chr7 | 20556576 | [C/T] | - | Intergenic | - |

| SNP IDs | Chromosomes | Physical positions (bp) | SNPs | Gene accession IDs | Sequence components of genes | Putative functions |
|---|---|---|---|---|---|---|
| CWSNP6353 | Ca-Kabuli-Chr7 | 20563931 | [G/A] | Ca12406 | Synonymous-CDS | KIP1-like |
| CWSNP6354 | Ca-Kabuli-Chr7 | 20564024 | [G/A] | Ca12406 | Synonymous-CDS | KIP1-like |
| CWSNP6355 | Ca-Kabuli-Chr7 | 20564266 | [G/A] | Ca12406 | Non-Synonymous-CDS | KIP1-like |
| CWSNP6356 | Ca-Kabuli-Chr7 | 20564269 | [C/T] | Ca12406 | Non-Synonymous-CDS | KIP1-like |
| CWSNP6357 | Ca-Kabuli-Chr7 | 20564297 | [A/G] | Ca12406 | Synonymous-CDS | KIP1-like |
| CWSNP6358 | Ca-Kabuli-Chr7 | 20579963 | [G/T] | - | Intergenic | - |
| CWSNP6359 | Ca-Kabuli-Chr7 | 20579974 | [A/T] | - | Intergenic | - |
| CWSNP6360 | Ca-Kabuli-Chr7 | 20605389 | [G/A] | - | Intergenic | - |
| CWSNP6361 | Ca-Kabuli-Chr7 | 20605384 | [C/G] | - | Intergenic | - |
| CWSNP6362 | Ca-Kabuli-Chr7 | 20605373 | [C/A] | - | Intergenic | - |
| CWSNP6363 | Ca-Kabuli-Chr7 | 20633040 | [A/G] | - | Intergenic | - |
| CWSNP6364 | Ca-Kabuli-Chr7 | 20637152 | [G/A] | Ca12415 | Synonymous-CDS | Protein synthesis factor, GTP-binding |
| CWSNP6365 | Ca-Kabuli-Chr7 | 20917747 | [A/G] | - | URR | - |
| CWSNP6366 | Ca-Kabuli-Chr7 | 20917743 | [T/G] | - | URR | - |
| CWSNP6367 | Ca-Kabuli-Chr7 | 20917740 | [A/G] | - | URR | - |
| CWSNP6368 | Ca-Kabuli-Chr7 | 21073385 | [A/C] | Ca14490 | Non-Synonymous-CDS | Alcohol dehydrogenase superfamily, zinc-containing |

| SNP IDs | Chromosomes | Physical positions (bp) | SNPs | Gene accession IDs | Sequence components of genes | Putative functions |
|---|---|---|---|---|---|---|
| CWSNP6369 | Ca-Kabuli-Chr7 | 21099782 | [G/T] | - | Intergenic | - |
| CWSNP6370 | Ca-Kabuli-Chr7 | 21402075 | [A/G] | Ca14503 | Synonymous-CDS | Aldehydedehydrogenase domain |
| CWSNP6371 | Ca-Kabuli-Chr7 | 21402117 | [A/C] | Ca14503 | Intron | Aldehydedehydrogenase domain |
| CWSNP6372 | Ca-Kabuli-Chr7 | 21595525 | [C/T] | - | DRR | - |
| CWSNP6373 | Ca-Kabuli-Chr7 | 21616285 | [A/G] | Ca14512 | Intron | Clathrin/coatomer adaptor,adaptin-like,N-terminal |
| CWSNP6374 | Ca-Kabuli-Chr7 | 21616287 | [T/G] | Ca14512 | Intron | Clathrin/coatomer adaptor,adaptin-like,N-terminal |
| CWSNP6375 | Ca-Kabuli-Chr7 | 21616294 | [T/G] | Ca14512 | Intron | Clathrin/coatomer adaptor,adaptin-like,N-terminal |
| CWSNP6376 | Ca-Kabuli-Chr7 | 21633103 | [C/G] | - | Intergenic | - |
| CWSNP6377 | Ca-Kabuli-Chr7 | 21639234 | [G/A] | Ca14515 | Intron | Clathrin/coatomer adaptor,adaptin-like,N-terminal |
| CWSNP6378 | Ca-Kabuli-Chr7 | 21639238 | [G/A] | Ca14515 | Intron | Clathrin/coatomer adaptor,adaptin-like,N-terminal |
| CWSNP6379 | Ca-Kabuli-Chr7 | 21639244 | [T/G] | Ca14515 | Intron | Clathrin/coatomer adaptor,adaptin-like,N-terminal |
| CWSNP6380 | Ca-Kabuli-Chr7 | 21639251 | [T/G] | Ca14515 | Intron | Clathrin/coatomer adaptor,adaptin-like,N-terminal |
| CWSNP6381 | Ca-Kabuli-Chr7 | 21639254 | [T/G] | Ca14515 | Intron | Clathrin/coatomer adaptor,adaptin-like,N-terminal |
| CWSNP6382 | Ca-Kabuli-Chr7 | 21639375 | [T/G] | Ca14515 | Intron | Clathrin/coatomer adaptor,adaptin-like,N-terminal |
| CWSNP6383 | Ca-Kabuli-Chr7 | 21644279 | [C/T] | - | Intergenic | - |
| CWSNP6384 | Ca-Kabuli-Chr7 | 21644342 | [G/A] | - | Intergenic | - |

| SNP IDs | Chromosomes | Physical positions (bp) | SNPs | Gene accession IDs | Sequence components of genes | Putative functions |
|---|---|---|---|---|---|---|
| CWSNP6385 | Ca-Kabuli-Chr7 | 21713147 | [T/G] | Ca14519 | Intron | - |
| CWSNP6386 | Ca-Kabuli-Chr7 | 21744514 | [T/C] | - | DRR | - |
| CWSNP6387 | Ca-Kabuli-Chr7 | 22721356 | [A/C] | Ca19530 | Synonymous-CDS | Haloacid dehalogenase-like hydrolase |
| CWSNP6388 | Ca-Kabuli-Chr7 | 22738152 | [C/G] | Ca19530 | Intron | Haloacid dehalogenase-like hydrolase |
| CWSNP6389 | Ca-Kabuli-Chr7 | 22738482 | [A/G] | Ca19530 | Intron | Haloacid dehalogenase-like hydrolase |
| CWSNP6390 | Ca-Kabuli-Chr7 | 22808696 | [A/G] | - | Intergenic | - |
| CWSNP6391 | Ca-Kabuli-Chr7 | 22808687 | [C/T] | - | Intergenic | - |
| CWSNP6392 | Ca-Kabuli-Chr7 | 22808717 | [C/T] | - | Intergenic | - |
| CWSNP6393 | Ca-Kabuli-Chr7 | 22839776 | [G/A] | - | Intergenic | - |
| CWSNP6394 | Ca-Kabuli-Chr7 | 23574823 | [C/T] | - | DRR | - |
| CWSNP6395 | Ca-Kabuli-Chr7 | 23575629 | [G/A] | Ca26017 | Non-Synonymous-CDS | Phosphatidyl inositol 3-/4-kinase, catalytic |
| CWSNP6396 | Ca-Kabuli-Chr7 | 23616050 | [A/G] | Ca26014 | Intron | Translin |
| CWSNP6397 | Ca-Kabuli-Chr7 | 24021690 | [T/A] | Ca19916 | Non-Synonymous-CDS | ZF-HD homeobox protein,Cys/His-rich dimerisation domain |
| CWSNP6398 | Ca-Kabuli-Chr7 | 24181321 | [G/A] | Ca19912 | Non-Synonymous-CDS | Zinc finger,RING-CH-type |
| CWSNP6399 | Ca-Kabuli-Chr7 | 24190633 | [G/A] | - | Intergenic | - |
| CWSNP6400 | Ca-Kabuli-Chr7 | 24190636 | [G/A] | - | Intergenic | - |

| SNP IDs | Chromosomes | Physical positions (bp) | SNPs | Gene accession IDs | Sequence components of genes | Putative functions |
|---|---|---|---|---|---|---|
| CWSNP6401 | Ca-Kabuli-Chr7 | 24190645 | [A/T] | - | Intergenic | - |
| CWSNP6402 | Ca-Kabuli-Chr7 | 24190650 | [G/A] | - | Intergenic | - |
| CWSNP6403 | Ca-Kabuli-Chr7 | 24190671 | [C/T] | - | Intergenic | - |
| CWSNP6404 | Ca-Kabuli-Chr7 | 24245096 | [C/T] | - | Intergenic | - |
| CWSNP6405 | Ca-Kabuli-Chr7 | 25218407 | [C/T] | - | Intergenic | - |
| CWSNP6406 | Ca-Kabuli-Chr7 | 25362272 | [T/C] | - | Intergenic | - |
| CWSNP6407 | Ca-Kabuli-Chr7 | 25428518 | [T/C] | - | Intergenic | - |
| CWSNP6408 | Ca-Kabuli-Chr7 | 25428548 | [G/T] | - | Intergenic | - |
| CWSNP6409 | Ca-Kabuli-Chr7 | 25428566 | [T/C] | - | Intergenic | - |
| CWSNP6410 | Ca-Kabuli-Chr7 | 25428660 | [G/A] | - | Intergenic | - |
| CWSNP6411 | Ca-Kabuli-Chr7 | 25569665 | [T/G] | Ca23609 | Intron | Zinc finger,PHD-type |
| CWSNP6412 | Ca-Kabuli-Chr7 | 25569657 | [T/C] | Ca23609 | Intron | Zinc finger,PHD-type |
| CWSNP6413 | Ca-Kabuli-Chr7 | 26070513 | [G/T] | - | Intergenic | - |
| CWSNP6414 | Ca-Kabuli-Chr7 | 26070515 | [A/G] | - | Intergenic | - |
| CWSNP6415 | Ca-Kabuli-Chr7 | 26094562 | [G/T] | - | Intergenic | - |
| CWSNP6416 | Ca-Kabuli-Chr7 | 26094567 | [G/A] | - | Intergenic | - |

| SNP IDs | Chromosomes | Physical positions (bp) | SNPs | Gene accession IDs | Sequence components of genes | Putative functions |
|---|---|---|---|---|---|---|
| CWSNP6417 | Ca-Kabuli-Chr7 | 26137315 | [T/C] | Ca23640 | Synonymous-CDS | IQ motif, EF-hand binding site |
| CWSNP6418 | Ca-Kabuli-Chr7 | 26146779 | [C/T] | Ca23640 | Intron | IQ motif, EF-hand binding site |
| CWSNP6419 | Ca-Kabuli-Chr7 | 26226146 | [G/C] | - | Intergenic | - |
| CWSNP6420 | Ca-Kabuli-Chr7 | 26692131 | [T/G] | - | DRR | - |
| CWSNP6421 | Ca-Kabuli-Chr7 | 26692130 | [C/T] | - | DRR | - |
| CWSNP6422 | Ca-Kabuli-Chr7 | 26692128 | [A/C] | - | DRR | - |
| CWSNP6423 | Ca-Kabuli-Chr7 | 26692194 | [C/A] | - | Intergenic | - |
| CWSNP6424 | Ca-Kabuli-Chr7 | 26692195 | [G/T] | - | Intergenic | - |
| CWSNP6425 | Ca-Kabuli-Chr7 | 26694365 | [G/A] | - | URR | - |
| CWSNP6426 | Ca-Kabuli-Chr7 | 26694440 | [C/A] | - | URR | - |
| CWSNP6427 | Ca-Kabuli-Chr7 | 26694441 | [G/A] | - | URR | - |
| CWSNP6428 | Ca-Kabuli-Chr7 | 26897910 | [A/G] | - | Intergenic | - |
| CWSNP6429 | Ca-Kabuli-Chr7 | 26897920 | [A/G] | - | Intergenic | - |
| CWSNP6430 | Ca-Kabuli-Chr7 | 26897923 | [G/A] | - | Intergenic | - |
| CWSNP6431 | Ca-Kabuli-Chr7 | 26897948 | [A/T] | - | Intergenic | - |
| CWSNP6432 | Ca-Kabuli-Chr7 | 26940917 | [G/A] | Ca16240 | Non-Synonymous-CDS | Plastocyanin-like |

| SNP IDs | Chromosomes | Physical positions (bp) | SNPs | Gene accession IDs | Sequence components of genes | Putative functions |
|---|---|---|---|---|---|---|
| CWSNP6433 | Ca-Kabuli-Chr7 | 26940967 | [A/G] | Ca16240 | Synonymous-CDS | Plastocyanin-like |
| CWSNP6434 | Ca-Kabuli-Chr7 | 26940979 | [A/G] | Ca16240 | Synonymous-CDS | Plastocyanin-like |
| CWSNP6435 | Ca-Kabuli-Chr7 | 26940985 | [T/G] | Ca16240 | Synonymous-CDS | Plastocyanin-like |
| CWSNP6436 | Ca-Kabuli-Chr7 | 27050259 | [C/G] | Ca16234 | Non-Synonymous-CDS | Dynamin,GTPase domain |
| CWSNP6437 | Ca-Kabuli-Chr7 | 27176481 | [C/A] | Ca16229 | Non-Synonymous-CDS | Lactate/malatedehydrogenase,N-terminal |
| CWSNP6438 | Ca-Kabuli-Chr7 | 27210919 | [C/T] | - | URR | - |
| CWSNP6439 | Ca-Kabuli-Chr7 | 27210881 | [C/T] | - | URR | - |
| CWSNP6440 | Ca-Kabuli-Chr7 | 27210871 | [C/A] | - | URR | - |
| CWSNP6441 | Ca-Kabuli-Chr7 | 27210858 | [C/T] | - | URR | - |
| CWSNP6442 | Ca-Kabuli-Chr7 | 27210853 | [A/G] | - | URR | - |
| CWSNP6443 | Ca-Kabuli-Chr7 | 27210848 | [A/T] | - | URR | - |
| CWSNP6444 | Ca-Kabuli-Chr7 | 27210988 | [C/T] | - | URR | - |
| CWSNP6445 | Ca-Kabuli-Chr7 | 27210989 | [C/T] | - | URR | - |
| CWSNP6446 | Ca-Kabuli-Chr7 | 27210992 | [C/A] | - | URR | - |
| CWSNP6447 | Ca-Kabuli-Chr7 | 27211004 | [T/C] | - | URR | - |
| CWSNP6448 | Ca-Kabuli-Chr7 | 27211036 | [G/A] | - | URR | - |

| SNP IDs | Chromosomes | Physical positions (bp) | SNPs | Gene accession IDs | Sequence components of genes | Putative functions |
|---|---|---|---|---|---|---|
| CWSNP6449 | Ca-Kabuli-Chr7 | 27211051 | [C/A] | - | URR | - |
| CWSNP6450 | Ca-Kabuli-Chr7 | 27633960 | [C/A] | - | Intergenic | - |
| CWSNP6451 | Ca-Kabuli-Chr7 | 27735234 | [A/G] | Ca16212 | Intron | Cyclin,C-terminal |
| CWSNP6452 | Ca-Kabuli-Chr7 | 27899782 | [T/C] | - | Intergenic | - |
| CWSNP6453 | Ca-Kabuli-Chr7 | 28931212 | [A/C] | - | DRR | - |
| CWSNP6454 | Ca-Kabuli-Chr7 | 28931223 | [C/A] | - | DRR | - |
| CWSNP6455 | Ca-Kabuli-Chr7 | 29150927 | [A/C] | Ca11791 | Intron | - |
| CWSNP6456 | Ca-Kabuli-Chr7 | 29153994 | [T/C] | Ca11791 | Synonymous-CDS | - |
| CWSNP6457 | Ca-Kabuli-Chr7 | 29157286 | [A/G] | Ca11791 | Synonymous-CDS | - |
| CWSNP6458 | Ca-Kabuli-Chr7 | 29168982 | [G/A] | - | DRR | - |
| CWSNP6459 | Ca-Kabuli-Chr7 | 29168980 | [C/T] | - | DRR | - |
| CWSNP6460 | Ca-Kabuli-Chr7 | 29190659 | [T/G] | Ca11786 | Non-Synonymous-CDS | Glutamyl/glutaminyl-tRNA synthetase,classIc |
| CWSNP6461 | Ca-Kabuli-Chr7 | 29225385 | [T/G] | - | URR | - |
| CWSNP6462 | Ca-Kabuli-Chr7 | 29225351 | [T/G] | - | URR | - |
| CWSNP6463 | Ca-Kabuli-Chr7 | 29225348 | [G/C] | - | URR | - |
| CWSNP6464 | Ca-Kabuli-Chr7 | 29229965 | [A/G] | - | URR | - |

| SNP IDs | Chromosomes | Physical positions (bp) | SNPs | Gene accession IDs | Sequence components of genes | Putative functions |
|---|---|---|---|---|---|---|
| CWSNP6465 | Ca-Kabuli-Chr7 | 29313080 | [T/C] | - | Intergenic | - |
| CWSNP6466 | Ca-Kabuli-Chr7 | 29313067 | [G/T] | - | Intergenic | - |
| CWSNP6467 | Ca-Kabuli-Chr7 | 29313059 | [G/A] | - | Intergenic | - |
| CWSNP6468 | Ca-Kabuli-Chr7 | 29313058 | [T/C] | - | Intergenic | - |
| CWSNP6469 | Ca-Kabuli-Chr7 | 29313051 | [T/C] | - | Intergenic | - |
| CWSNP6470 | Ca-Kabuli-Chr7 | 29313257 | [G/A] | - | Intergenic | - |
| CWSNP6471 | Ca-Kabuli-Chr7 | 29482556 | [G/A] | - | Intergenic | - |
| CWSNP6472 | Ca-Kabuli-Chr7 | 29484304 | [G/A] | - | Intergenic | - |
| CWSNP6473 | Ca-Kabuli-Chr7 | 29607943 | [C/T] | - | URR | - |
| CWSNP6474 | Ca-Kabuli-Chr7 | 29613699 | [C/T] | Ca11764 | Synonymous-CDS | F-boxdomain,cyclin-like |
| CWSNP6475 | Ca-Kabuli-Chr7 | 29625884 | [T/C] | - | Intergenic | - |
| CWSNP6476 | Ca-Kabuli-Chr7 | 29705531 | [T/G] | Ca11752 | Non-Synonymous-CDS | - |
| CWSNP6477 | Ca-Kabuli-Chr7 | 29705499 | [T/C] | Ca11752 | Non-Synonymous-CDS | - |
| CWSNP6478 | Ca-Kabuli-Chr7 | 29796564 | [G/T] | Ca11746 | Non-Synonymous-CDS | Spc97/Spc98 |
| CWSNP6479 | Ca-Kabuli-Chr7 | 29801148 | [A/G] | Ca11746 | Non-Synonymous-CDS | Spc97/Spc98 |
| CWSNP6480 | Ca-Kabuli-Chr7 | 29801154 | [T/C] | Ca11746 | Non-Synonymous-CDS | Spc97/Spc98 |

| SNP IDs | Chromosomes | Physical positions (bp) | SNPs | Gene accession IDs | Sequence components of genes | Putative functions |
|---|---|---|---|---|---|---|
| CWSNP6481 | Ca-Kabuli-Chr7 | 29837035 | [G/A] | - | DRR | - |
| CWSNP6482 | Ca-Kabuli-Chr7 | 29838073 | [C/T] | - | DRR | - |
| CWSNP6483 | Ca-Kabuli-Chr7 | 29848248 | [T/C] | Ca11739 | Intron | Lipase,class3 |
| CWSNP6484 | Ca-Kabuli-Chr7 | 29848433 | [C/T] | Ca11739 | Synonymous-CDS | Lipase,class3 |
| CWSNP6485 | Ca-Kabuli-Chr7 | 29849509 | [C/T] | Ca11739 | Non-Synonymous-CDS | Lipase,class3 |
| CWSNP6486 | Ca-Kabuli-Chr7 | 29849544 | [A/G] | Ca11739 | Synonymous-CDS | Lipase,class3 |
| CWSNP6487 | Ca-Kabuli-Chr7 | 29884719 | [G/T] | Ca11736 | Non-Synonymous-CDS | TetratricopeptideTPR-1 |
| CWSNP6488 | Ca-Kabuli-Chr7 | 29884718 | [C/A] | Ca11736 | Synonymous-CDS | TetratricopeptideTPR-1 |
| CWSNP6489 | Ca-Kabuli-Chr7 | 29925008 | [G/A] | - | DRR | - |
| CWSNP6490 | Ca-Kabuli-Chr7 | 30004767 | [C/T] | - | Intergenic | - |
| CWSNP6491 | Ca-Kabuli-Chr7 | 30004934 | [A/C] | - | Intergenic | - |
| CWSNP6492 | Ca-Kabuli-Chr7 | 30026039 | [T/G] | Ca11727 | Intron | Glycosyltransferase,family2 |
| CWSNP6493 | Ca-Kabuli-Chr7 | 30026017 | [C/T] | Ca11727 | Intron | Glycosyltransferase,family2 |
| CWSNP6494 | Ca-Kabuli-Chr7 | 30029793 | [A/C] | - | DRR | - |
| CWSNP6495 | Ca-Kabuli-Chr7 | 30029820 | [T/C] | - | DRR | - |
| CWSNP6496 | Ca-Kabuli-Chr7 | 30060299 | [C/A] | Ca11722 | Synonymous-CDS | ATPase,P-type,K/Mg/Cd/Cu/Zn/Na/Ca/Na/H-transporter |

| SNP IDs | Chromosomes | Physical positions (bp) | SNPs | Gene accession IDs | Sequence components of genes | Putative functions |
|---|---|---|---|---|---|---|
| CWSNP6497 | Ca-Kabuli-Chr7 | 30085847 | [C/T] | Ca11718 | Non-Synonymous-CDS | Domain of unknown function DUF828 |
| CWSNP6498 | Ca-Kabuli-Chr7 | 30085883 | [T/G] | Ca11718 | Non-Synonymous-CDS | Domain of unknown function DUF828 |
| CWSNP6499 | Ca-Kabuli-Chr7 | 30085906 | [C/T] | - | Intergenic | - |
| CWSNP6500 | Ca-Kabuli-Chr7 | 30085910 | [T/A] | - | Intergenic | - |
| CWSNP6501 | Ca-Kabuli-Chr7 | 30099003 | [C/T] | - | Intergenic | - |
| CWSNP6502 | Ca-Kabuli-Chr7 | 30107340 | [A/G] | - | Intergenic | - |
| CWSNP6503 | Ca-Kabuli-Chr7 | 30403618 | [G/A] | - | Intergenic | - |
| CWSNP6504 | Ca-Kabuli-Chr7 | 30691324 | [A/C] | Ca23327 | Non-Synonymous-CDS | - |
| CWSNP6505 | Ca-Kabuli-Chr7 | 30691318 | [A/C] | Ca23327 | Non-Synonymous-CDS | - |
| CWSNP6506 | Ca-Kabuli-Chr7 | 30768315 | [G/A] | - | URR | - |
| CWSNP6507 | Ca-Kabuli-Chr7 | 30850950 | [A/C] | - | Intergenic | - |
| CWSNP6508 | Ca-Kabuli-Chr7 | 31190059 | [G/A] | Ca10139 | Synonymous-CDS | RNApolymeraseII-associated,Paf1 |
| CWSNP6509 | Ca-Kabuli-Chr7 | 31193786 | [T/G] | Ca10139 | Synonymous-CDS | RNApolymeraseII-associated,Paf1 |
| CWSNP6510 | Ca-Kabuli-Chr7 | 31314421 | [T/A] | - | DRR | - |
| CWSNP6511 | Ca-Kabuli-Chr7 | 31324279 | [A/G] | - | Intergenic | - |
| CWSNP6512 | Ca-Kabuli-Chr7 | 31401807 | [T/C] | - | URR | - |

| SNP IDs | Chromosomes | Physical positions (bp) | SNPs | Gene accession IDs | Sequence components of genes | Putative functions |
|---|---|---|---|---|---|---|
| CWSNP6513 | Ca-Kabuli-Chr7 | 31442425 | [A/C] | Ca10114 | Intron | PWWP |
| CWSNP6514 | Ca-Kabuli-Chr7 | 31612697 | [T/A] | Ca10100 | Non-Synonymous-CDS | ABC transporter, transmembrane domain |
| CWSNP6515 | Ca-Kabuli-Chr7 | 31639701 | [C/T] | Ca10099 | Intron | Domain of unknown function DUF250 |
| CWSNP6516 | Ca-Kabuli-Chr7 | 31830238 | [A/G] | - | Intergenic | - |
| CWSNP6517 | Ca-Kabuli-Chr7 | 31851096 | [T/A] | Ca10079 | Non-Synonymous-CDS | Prenyltransferase/squaleneoxidase |
| CWSNP6518 | Ca-Kabuli-Chr7 | 31873079 | [G/T] | - | Intergenic | - |
| CWSNP6519 | Ca-Kabuli-Chr7 | 31996345 | [A/C] | - | Intergenic | - |
| CWSNP6520 | Ca-Kabuli-Chr7 | 32017288 | [G/T] | - | Intergenic | - |
| CWSNP6521 | Ca-Kabuli-Chr7 | 32017286 | [T/G] | - | Intergenic | - |
| CWSNP6522 | Ca-Kabuli-Chr7 | 32017252 | [G/A] | - | Intergenic | - |
| CWSNP6523 | Ca-Kabuli-Chr7 | 32017238 | [C/T] | - | Intergenic | - |
| CWSNP6524 | Ca-Kabuli-Chr7 | 32017228 | [G/A] | - | Intergenic | - |
| CWSNP6525 | Ca-Kabuli-Chr7 | 32017221 | [C/T] | - | Intergenic | - |
| CWSNP6526 | Ca-Kabuli-Chr7 | 32127288 | [T/A] | - | URR | - |
| CWSNP6527 | Ca-Kabuli-Chr7 | 32283585 | [T/G] | Ca10045 | Intron | vonWillebrandfactor,typeA |
| CWSNP6528 | Ca-Kabuli-Chr7 | 32309397 | [A/T] | Ca10045 | Intron | vonWillebrandfactor,typeA |

| **SNP IDs** | **Chromosomes** | **Physical positions (bp)** | **SNPs** | **Gene accession IDs** | **Sequence components of genes** | **Putative functions** |
|---|---|---|---|---|---|---|
| CWSNP6529 | Ca-Kabuli-Chr7 | 32309411 | [G/T] | Ca10045 | Intron | vonWillebrandfactor,typeA |
| CWSNP6530 | Ca-Kabuli-Chr7 | 32309415 | [C/T] | Ca10045 | Intron | vonWillebrandfactor,typeA |
| CWSNP6531 | Ca-Kabuli-Chr7 | 32309429 | [C/T] | Ca10045 | Intron | vonWillebrandfactor,typeA |
| CWSNP6532 | Ca-Kabuli-Chr7 | 32309377 | [C/T] | Ca10045 | Intron | vonWillebrandfactor,typeA |
| CWSNP6533 | Ca-Kabuli-Chr7 | 32309376 | [C/G] | Ca10045 | Intron | vonWillebrandfactor,typeA |
| CWSNP6534 | Ca-Kabuli-Chr7 | 32399759 | [C/T] | Ca10038 | Synonymous-CDS | LsmADdomain |
| CWSNP6535 | Ca-Kabuli-Chr7 | 32404932 | [T/C] | - | URR | - |
| CWSNP6536 | Ca-Kabuli-Chr7 | 32451377 | [A/C] | Ca10034 | Intron | Protein of unknown function DUF1296 |
| CWSNP6537 | Ca-Kabuli-Chr7 | 32522201 | [A/G] | Ca10028 | Synonymous-CDS | SANT domain, DNA binding |
| CWSNP6538 | Ca-Kabuli-Chr7 | 32522203 | [A/G] | Ca10028 | Non-Synonymous-CDS | SANT domain, DNA binding |
| CWSNP6539 | Ca-Kabuli-Chr7 | 32522207 | [T/G] | Ca10028 | Synonymous-CDS | SANT domain, DNA binding |
| CWSNP6540 | Ca-Kabuli-Chr7 | 32522213 | [A/G] | Ca10028 | Synonymous-CDS | SANT domain, DNA binding |
| CWSNP6541 | Ca-Kabuli-Chr7 | 32522230 | [T/G] | Ca10028 | Non-Synonymous-CDS | SANT domain, DNA binding |
| CWSNP6542 | Ca-Kabuli-Chr7 | 32557599 | [G/A] | - | Intergenic | - |
| CWSNP6543 | Ca-Kabuli-Chr7 | 32669570 | [A/G] | - | Intergenic | - |
| CWSNP6544 | Ca-Kabuli-Chr7 | 32754633 | [T/G] | Ca10017 | Intron | - |

| SNP IDs | Chromosomes | Physical positions (bp) | SNPs | Gene accession IDs | Sequence components of genes | Putative functions |
|---|---|---|---|---|---|---|
| CWSNP6545 | Ca-Kabuli-Chr7 | 32755662 | [G/T] | Ca10017 | Non-Synonymous-CDS | - |
| CWSNP6546 | Ca-Kabuli-Chr7 | 32784903 | [T/G] | Ca10015 | Synonymous-CDS | TranscriptioninitiationfactorTFIID |
| CWSNP6547 | Ca-Kabuli-Chr7 | 32799037 | [A/G] | - | Intergenic | - |
| CWSNP6548 | Ca-Kabuli-Chr7 | 32807457 | [T/A] | - | Intergenic | - |
| CWSNP6549 | Ca-Kabuli-Chr7 | 32807499 | [A/G] | - | Intergenic | - |
| CWSNP6550 | Ca-Kabuli-Chr7 | 32874847 | [C/T] | - | Intergenic | - |
| CWSNP6551 | Ca-Kabuli-Chr7 | 32874840 | [C/T] | - | Intergenic | - |
| CWSNP6552 | Ca-Kabuli-Chr7 | 32875799 | [T/A] | Ca10011 | Synonymous-CDS | Germin |
| CWSNP6553 | Ca-Kabuli-Chr7 | 32942199 | [A/G] | - | DRR | - |
| CWSNP6554 | Ca-Kabuli-Chr7 | 32942951 | [T/G] | - | Intergenic | - |
| CWSNP6555 | Ca-Kabuli-Chr7 | 32989796 | [T/A] | Ca10000 | Synonymous-CDS | Protein kinase, catalytic domain |
| CWSNP6556 | Ca-Kabuli-Chr7 | 32989838 | [C/T] | Ca10000 | Synonymous-CDS | Protein kinase, catalytic domain |
| CWSNP6557 | Ca-Kabuli-Chr7 | 32989903 | [A/G] | Ca10000 | Intron | Protein kinase, catalytic domain |
| CWSNP6558 | Ca-Kabuli-Chr7 | 32989902 | [C/A] | Ca10000 | Intron | Protein kinase, catalytic domain |
| CWSNP6559 | Ca-Kabuli-Chr7 | 33065611 | [G/T] | - | Intergenic | - |
| CWSNP6560 | Ca-Kabuli-Chr7 | 33121674 | [G/C] | - | DRR | - |

| SNP IDs | Chromosomes | Physical positions (bp) | SNPs | Gene accession IDs | Sequence components of genes | Putative functions |
|---|---|---|---|---|---|---|
| CWSNP6561 | Ca-Kabuli-Chr7 | 33121640 | [C/A] | - | DRR | - |
| CWSNP6562 | Ca-Kabuli-Chr7 | 33122473 | [A/C] | - | DRR | - |
| CWSNP6563 | Ca-Kabuli-Chr7 | 33182932 | [G/A] | Ca16149 | Synonymous-CDS | Lipase,class3 |
| CWSNP6564 | Ca-Kabuli-Chr7 | 33267485 | [T/C] | - | URR | - |
| CWSNP6565 | Ca-Kabuli-Chr7 | 33267496 | [T/A] | - | URR | - |
| CWSNP6566 | Ca-Kabuli-Chr7 | 33335978 | [C/A] | Ca16158 | Non-Synonymous-CDS | RNA recognition motif domain |
| CWSNP6567 | Ca-Kabuli-Chr7 | 33364329 | [C/T] | - | DRR | - |
| CWSNP6568 | Ca-Kabuli-Chr7 | 33364354 | [G/A] | - | DRR | - |
| CWSNP6569 | Ca-Kabuli-Chr7 | 33378427 | [C/T] | Ca16163 | Intron | Spt20family |
| CWSNP6570 | Ca-Kabuli-Chr7 | 33411746 | [C/A] | Ca16164 | Intron | Zinc finger,CCCH-type |
| CWSNP6571 | Ca-Kabuli-Chr7 | 33465828 | [C/A] | - | URR | - |
| CWSNP6572 | Ca-Kabuli-Chr7 | 33465818 | [G/A] | - | URR | - |
| CWSNP6573 | Ca-Kabuli-Chr7 | 33465805 | [T/C] | - | URR | - |
| CWSNP6574 | Ca-Kabuli-Chr7 | 33465802 | [C/A] | - | URR | - |
| CWSNP6575 | Ca-Kabuli-Chr7 | 33465800 | [T/A] | - | URR | - |
| CWSNP6576 | Ca-Kabuli-Chr7 | 33465781 | [G/T] | - | URR | - |

| SNP IDs | Chromosomes | Physical positions (bp) | SNPs | Gene accession IDs | Sequence components of genes | Putative functions |
|---|---|---|---|---|---|---|
| CWSNP6577 | Ca-Kabuli-Chr7 | 33465855 | [A/C] | - | URR | - |
| CWSNP6578 | Ca-Kabuli-Chr7 | 33480940 | [C/T] | - | Intergenic | - |
| CWSNP6579 | Ca-Kabuli-Chr7 | 33573605 | [C/T] | Ca16177 | Synonymous-CDS | CCAAT-binding factor |
| CWSNP6580 | Ca-Kabuli-Chr7 | 33573655 | [A/C] | Ca16177 | Non-Synonymous-CDS | CCAAT-binding factor |
| CWSNP6581 | Ca-Kabuli-Chr7 | 33619268 | [G/C] | - | Intergenic | - |
| CWSNP6582 | Ca-Kabuli-Chr7 | 33641799 | [G/T] | Ca16180 | Synonymous-CDS | Pathogenesis-related transcriptional factor/ERF, DNA-binding |
| CWSNP6583 | Ca-Kabuli-Chr7 | 33641882 | [A/T] | Ca16180 | Intron | Pathogenesis-related transcriptional factor/ERF, DNA-binding |
| CWSNP6584 | Ca-Kabuli-Chr7 | 33674454 | [T/C] | Ca16181 | Synonymous-CDS | PeptidaseS8/S53,subtilisin/kexin/sedolisin |
| CWSNP6585 | Ca-Kabuli-Chr7 | 33744521 | [C/G] | Ca16182 | Intron | ARID/BRIGHTDNA-binding domain |
| CWSNP6586 | Ca-Kabuli-Chr7 | 33753917 | [G/A] | Ca16183 | Synonymous-CDS | Initiation factor2B-related |
| CWSNP6587 | Ca-Kabuli-Chr7 | 33761944 | [G/A] | Ca16184 | Synonymous-CDS | - |
| CWSNP6588 | Ca-Kabuli-Chr7 | 33762053 | [T/G] | Ca16184 | Non-Synonymous-CDS | - |
| CWSNP6589 | Ca-Kabuli-Chr7 | 33821658 | [C/T] | Ca16190 | Intron | 1-aminocyclopropane-1-carboxylatesynthase |
| CWSNP6590 | Ca-Kabuli-Chr7 | 33865463 | [T/C] | Ca16193 | Intron | Exocyst complex component Sec10 |
| CWSNP6591 | Ca-Kabuli-Chr7 | 33865475 | [G/A] | Ca16193 | Intron | Exocyst complex component Sec10 |
| CWSNP6592 | Ca-Kabuli-Chr7 | 33867334 | [C/T] | Ca16193 | Intron | Exocyst complex component Sec10 |

| SNP IDs | Chromosomes | Physical positions (bp) | SNPs | Gene accession IDs | Sequence components of genes | Putative functions |
|---|---|---|---|---|---|---|
| CWSNP6593 | Ca-Kabuli-Chr7 | 33867491 | [C/T] | Ca16193 | Intron | Exocyst complex component Sec10 |
| CWSNP6594 | Ca-Kabuli-Chr7 | 33867674 | [C/T] | Ca16193 | Intron | Exocyst complex component Sec10 |
| CWSNP6595 | Ca-Kabuli-Chr7 | 33869367 | [G/A] | Ca16193 | Synonymous-CDS | Exocyst complex component Sec10 |
| CWSNP6596 | Ca-Kabuli-Chr7 | 33910324 | [C/A] | - | Intergenic | - |
| CWSNP6597 | Ca-Kabuli-Chr7 | 33910870 | [C/A] | - | Intergenic | - |
| CWSNP6598 | Ca-Kabuli-Chr7 | 33912719 | [T/G] | - | Intergenic | - |
| CWSNP6599 | Ca-Kabuli-Chr7 | 33912866 | [T/C] | - | Intergenic | - |
| CWSNP6600 | Ca-Kabuli-Chr7 | 33912899 | [T/A] | - | Intergenic | - |
| CWSNP6601 | Ca-Kabuli-Chr7 | 33912902 | [A/T] | - | Intergenic | - |
| CWSNP6602 | Ca-Kabuli-Chr7 | 33912913 | [A/T] | - | Intergenic | - |
| CWSNP6603 | Ca-Kabuli-Chr7 | 33912916 | [G/T] | - | Intergenic | - |
| CWSNP6604 | Ca-Kabuli-Chr7 | 33912921 | [T/G] | - | Intergenic | - |
| CWSNP6605 | Ca-Kabuli-Chr7 | 33912950 | [C/T] | - | Intergenic | - |
| CWSNP6606 | Ca-Kabuli-Chr7 | 33912966 | [T/C] | - | Intergenic | - |
| CWSNP6607 | Ca-Kabuli-Chr7 | 33913094 | [A/G] | - | Intergenic | - |
| CWSNP6608 | Ca-Kabuli-Chr7 | 33913047 | [C/T] | - | Intergenic | - |

| SNP IDs | Chromosomes | Physical positions (bp) | SNPs | Gene accession IDs | Sequence components of genes | Putative functions |
|---|---|---|---|---|---|---|
| CWSNP6609 | Ca-Kabuli-Chr7 | 33913045 | [G/T] | - | Intergenic | - |
| CWSNP6610 | Ca-Kabuli-Chr7 | 34107603 | [C/A] | - | Intergenic | - |
| CWSNP6611 | Ca-Kabuli-Chr7 | 34152124 | [G/C] | Ca21006 | Non-Synonymous-CDS | DNA-directed RNA polymerase, subunit 2,domain 6 |
| CWSNP6612 | Ca-Kabuli-Chr7 | 34271486 | [A/G] | - | DRR | - |
| CWSNP6613 | Ca-Kabuli-Chr7 | 34277014 | [G/T] | - | URR | - |
| CWSNP6614 | Ca-Kabuli-Chr7 | 34277010 | [A/G] | - | URR | - |
| CWSNP6615 | Ca-Kabuli-Chr7 | 34279673 | [G/A] | - | URR | - |
| CWSNP6616 | Ca-Kabuli-Chr7 | 34279725 | [A/G] | - | URR | - |
| CWSNP6617 | Ca-Kabuli-Chr7 | 34279715 | [A/C] | - | URR | - |
| CWSNP6618 | Ca-Kabuli-Chr7 | 34318860 | [C/T] | - | Intergenic | - |
| CWSNP6619 | Ca-Kabuli-Chr7 | 34318862 | [A/G] | - | Intergenic | - |
| CWSNP6620 | Ca-Kabuli-Chr7 | 34474614 | [A/C] | Ca17602 | Non-Synonymous-CDS | SSXT |
| CWSNP6621 | Ca-Kabuli-Chr7 | 34801839 | [A/T] | Ca17614 | Synonymous-CDS | - |
| CWSNP6622 | Ca-Kabuli-Chr7 | 34857693 | [C/T] | Ca17617 | Non-Synonymous-CDS | Armadillo |
| CWSNP6623 | Ca-Kabuli-Chr7 | 34947743 | [A/C] | Ca17626 | Intron | Phosphoribosyltransferase |
| CWSNP6624 | Ca-Kabuli-Chr7 | 35016516 | [G/C] | - | Intergenic | - |

| SNP IDs | Chromosomes | Physical positions (bp) | SNPs | Gene accession IDs | Sequence components of genes | Putative functions |
|---|---|---|---|---|---|---|
| CWSNP6625 | Ca-Kabuli-Chr7 | 35072425 | [A/T] | - | Intergenic | - |
| CWSNP6626 | Ca-Kabuli-Chr7 | 35074362 | [T/C] | - | Intergenic | - |
| CWSNP6627 | Ca-Kabuli-Chr7 | 35112785 | [T/C] | Ca17637 | Synonymous-CDS | Importin-beta,N-terminal |
| CWSNP6628 | Ca-Kabuli-Chr7 | 35114049 | [C/T] | Ca17637 | Non-Synonymous-CDS | Importin-beta,N-terminal |
| CWSNP6629 | Ca-Kabuli-Chr7 | 35134809 | [A/C] | Ca17638 | Non-Synonymous-CDS | Pathogenesis-related transcriptional factor/ERF, DNA-binding |
| CWSNP6630 | Ca-Kabuli-Chr7 | 35134863 | [T/C] | Ca17638 | Synonymous-CDS | Pathogenesis-related transcriptional factor/ERF, DNA-binding |
| CWSNP6631 | Ca-Kabuli-Chr7 | 35181619 | [T/G] | Ca17639 | Non-Synonymous-CDS | Helix-loop-helix DNA-binding domain |
| CWSNP6632 | Ca-Kabuli-Chr7 | 35181661 | [T/C] | Ca17639 | Synonymous-CDS | Helix-loop-helix DNA-binding domain |
| CWSNP6633 | Ca-Kabuli-Chr7 | 35181660 | [G/A] | Ca17639 | Non-Synonymous-CDS | Helix-loop-helix DNA-binding domain |
| CWSNP6634 | Ca-Kabuli-Chr7 | 35181635 | [G/C] | Ca17639 | Non-Synonymous-CDS | Helix-loop-helix DNA-binding domain |
| CWSNP6635 | Ca-Kabuli-Chr7 | 35227794 | [A/C] | - | URR | - |
| CWSNP6636 | Ca-Kabuli-Chr7 | 35258029 | [T/G] | Ca17644 | Intron | Dynamin central domain |
| CWSNP6637 | Ca-Kabuli-Chr7 | 35263711 | [A/T] | - | Intergenic | - |
| CWSNP6638 | Ca-Kabuli-Chr7 | 35263712 | [G/T] | - | Intergenic | - |
| CWSNP6639 | Ca-Kabuli-Chr7 | 35263736 | [C/G] | - | Intergenic | - |
| CWSNP6640 | Ca-Kabuli-Chr7 | 35263758 | [A/G] | - | Intergenic | - |

| SNP IDs | Chromosomes | Physical positions (bp) | SNPs | Gene accession IDs | Sequence components of genes | Putative functions |
|---|---|---|---|---|---|---|
| CWSNP6641 | Ca-Kabuli-Chr7 | 35274368 | [A/G] | - | Intergenic | - |
| CWSNP6642 | Ca-Kabuli-Chr7 | 35274859 | [T/G] | - | Intergenic | - |
| CWSNP6643 | Ca-Kabuli-Chr7 | 35274858 | [G/A] | - | Intergenic | - |
| CWSNP6644 | Ca-Kabuli-Chr7 | 35274828 | [A/G] | - | Intergenic | - |
| CWSNP6645 | Ca-Kabuli-Chr7 | 35279675 | [A/G] | Ca17646 | Non-Synonymous-CDS | - |
| CWSNP6646 | Ca-Kabuli-Chr7 | 35280189 | [G/A] | Ca17646 | Synonymous-CDS | - |
| CWSNP6647 | Ca-Kabuli-Chr7 | 35280167 | [A/G] | Ca17646 | Synonymous-CDS | - |
| CWSNP6648 | Ca-Kabuli-Chr7 | 35576427 | [C/A] | Ca17765 | Non-Synonymous-CDS | Reticulon |
| CWSNP6649 | Ca-Kabuli-Chr7 | 35619487 | [G/A] | - | Intergenic | - |
| CWSNP6650 | Ca-Kabuli-Chr7 | 35619518 | [G/C] | - | Intergenic | - |
| CWSNP6651 | Ca-Kabuli-Chr7 | 35622168 | [A/G] | Ca17769 | Non-Synonymous-CDS | Ketopantoate hydroxy methyltransferase |
| CWSNP6652 | Ca-Kabuli-Chr7 | 35680423 | [A/C] | - | Intergenic | - |
| CWSNP6653 | Ca-Kabuli-Chr7 | 35911620 | [T/G] | - | URR | - |
| CWSNP6654 | Ca-Kabuli-Chr7 | 35911617 | [A/C] | - | URR | - |
| CWSNP6655 | Ca-Kabuli-Chr7 | 35911615 | [C/T] | - | URR | - |
| CWSNP6656 | Ca-Kabuli-Chr7 | 35911612 | [T/G] | - | URR | - |

| SNP IDs | Chromosomes | Physical positions (bp) | SNPs | Gene accession IDs | Sequence components of genes | Putative functions |
|---|---|---|---|---|---|---|
| CWSNP6657 | Ca-Kabuli-Chr7 | 35911606 | [T/C] | - | URR | - |
| CWSNP6658 | Ca-Kabuli-Chr7 | 35947529 | [T/C] | Ca17800 | Non-Synonymous-CDS | Transposase,Ptta/En/Spm,plant |
| CWSNP6659 | Ca-Kabuli-Chr7 | 35947550 | [C/T] | Ca17800 | Non-Synonymous-CDS | Transposase,Ptta/En/Spm,plant |
| CWSNP6660 | Ca-Kabuli-Chr7 | 35947551 | [A/G] | Ca17800 | Synonymous-CDS | Transposase,Ptta/En/Spm,plant |
| CWSNP6661 | Ca-Kabuli-Chr7 | 35959825 | [G/A] | - | Intergenic | - |
| CWSNP6662 | Ca-Kabuli-Chr7 | 35959878 | [G/A] | - | Intergenic | - |
| CWSNP6663 | Ca-Kabuli-Chr7 | 36711954 | [C/A] | Ca13756 | Non-Synonymous-CDS | Mediator complex,subunit Med12 |
| CWSNP6664 | Ca-Kabuli-Chr7 | 36715249 | [T/C] | Ca13756 | Synonymous-CDS | Mediator complex,subunit Med12 |
| CWSNP6665 | Ca-Kabuli-Chr7 | 36745439 | [T/G] | - | Intergenic | - |
| CWSNP6666 | Ca-Kabuli-Chr7 | 36916273 | [T/A] | - | Intergenic | - |
| CWSNP6667 | Ca-Kabuli-Chr7 | 36998096 | [T/C] | Ca13784 | Synonymous-CDS | PeptidaseC48,SUMO/Sentrin/Ubl1 |
| CWSNP6668 | Ca-Kabuli-Chr7 | 36998113 | [C/T] | Ca13784 | Synonymous-CDS | PeptidaseC48,SUMO/Sentrin/Ubl1 |
| CWSNP6669 | Ca-Kabuli-Chr7 | 37058979 | [C/T] | Ca13790 | Non-Synonymous-CDS | Hydroxy methylglutaryl-CoAlyase,active site |
| CWSNP6670 | Ca-Kabuli-Chr7 | 37187439 | [A/G] | - | Intergenic | - |
| CWSNP6671 | Ca-Kabuli-Chr7 | 37626729 | [G/T] | Ca22806 | Non-Synonymous-CDS | Transposase,Ptta/En/Spm,plant |
| CWSNP6672 | Ca-Kabuli-Chr7 | 37626653 | [A/C] | Ca22806 | Non-Synonymous-CDS | Transposase,Ptta/En/Spm,plant |

| SNP IDs | Chromosomes | Physical positions (bp) | SNPs | Gene accession IDs | Sequence components of genes | Putative functions |
|---|---|---|---|---|---|---|
| CWSNP6673 | Ca-Kabuli-Chr7 | 37626687 | [A/G] | Ca22806 | Non-Synonymous-CDS | Transposase,Ptta/En/Spm,plant |
| CWSNP6674 | Ca-Kabuli-Chr7 | 37626666 | [C/T] | Ca22806 | Non-Synonymous-CDS | Transposase,Ptta/En/Spm,plant |
| CWSNP6675 | Ca-Kabuli-Chr7 | 37703967 | [G/A] | - | Intergenic | - |
| CWSNP6676 | Ca-Kabuli-Chr7 | 37703966 | [C/T] | - | Intergenic | - |
| CWSNP6677 | Ca-Kabuli-Chr7 | 37703965 | [C/T] | - | Intergenic | - |
| CWSNP6678 | Ca-Kabuli-Chr7 | 37703921 | [G/T] | - | Intergenic | - |
| CWSNP6679 | Ca-Kabuli-Chr7 | 37703912 | [G/A] | - | Intergenic | - |
| CWSNP6680 | Ca-Kabuli-Chr7 | 37703913 | [C/G] | - | Intergenic | - |
| CWSNP6681 | Ca-Kabuli-Chr7 | 37703936 | [C/T] | - | Intergenic | - |
| CWSNP6682 | Ca-Kabuli-Chr7 | 37857936 | [G/T] | - | DRR | - |
| CWSNP6683 | Ca-Kabuli-Chr7 | 37857928 | [A/T] | - | DRR | - |
| CWSNP6684 | Ca-Kabuli-Chr7 | 38047374 | [A/G] | - | Intergenic | - |
| CWSNP6685 | Ca-Kabuli-Chr7 | 38047378 | [C/A] | - | Intergenic | - |
| CWSNP6686 | Ca-Kabuli-Chr7 | 38143361 | [A/G] | Ca16396 | Intron | RNA recognition motif domain |
| CWSNP6687 | Ca-Kabuli-Chr7 | 38143524 | [T/A] | Ca16396 | Intron | RNA recognition motif domain |
| CWSNP6688 | Ca-Kabuli-Chr7 | 38143452 | [G/A] | Ca16396 | Intron | RNA recognition motif domain |

| SNP IDs | Chromosomes | Physical positions (bp) | SNPs | Gene accession IDs | Sequence components of genes | Putative functions |
|---|---|---|---|---|---|---|
| CWSNP6689 | Ca-Kabuli-Chr7 | 38460464 | [G/A] | - | Intergenic | - |
| CWSNP6690 | Ca-Kabuli-Chr7 | 38460452 | [C/T] | - | Intergenic | - |
| CWSNP6691 | Ca-Kabuli-Chr7 | 38460425 | [G/A] | - | Intergenic | - |
| CWSNP6692 | Ca-Kabuli-Chr7 | 39182564 | [A/T] | Ca19581 | Non-Synonymous-CDS | BEACH domain |
| CWSNP6693 | Ca-Kabuli-Chr7 | 39339510 | [T/G] | Ca19588 | Non-Synonymous-CDS | WRC |
| CWSNP6694 | Ca-Kabuli-Chr7 | 39339509 | [G/A] | Ca19588 | Synonymous-CDS | WRC |
| CWSNP6695 | Ca-Kabuli-Chr7 | 39339508 | [G/A] | Ca19588 | Non-Synonymous-CDS | WRC |
| CWSNP6696 | Ca-Kabuli-Chr7 | 39339503 | [C/A] | Ca19588 | Synonymous-CDS | WRC |
| CWSNP6697 | Ca-Kabuli-Chr7 | 39339502 | [C/A] | Ca19588 | Non-Synonymous-CDS | WRC |
| CWSNP6698 | Ca-Kabuli-Chr7 | 39339501 | [G/C] | Ca19588 | Non-Synonymous-CDS | WRC |
| CWSNP6699 | Ca-Kabuli-Chr7 | 39339500 | [G/T] | Ca19588 | Synonymous-CDS | WRC |
| CWSNP6700 | Ca-Kabuli-Chr7 | 39339499 | [C/T] | Ca19588 | Non-Synonymous-CDS | WRC |
| CWSNP6701 | Ca-Kabuli-Chr7 | 39339498 | [G/T] | Ca19588 | Non-Synonymous-CDS | WRC |
| CWSNP6702 | Ca-Kabuli-Chr7 | 39339495 | [G/T] | Ca19588 | Non-Synonymous-CDS | WRC |
| CWSNP6703 | Ca-Kabuli-Chr7 | 39399280 | [T/C] | Ca19592 | Synonymous-CDS | Domain of unknown function DUF296 |
| CWSNP6704 | Ca-Kabuli-Chr7 | 39680221 | [A/C] | Ca19607 | Intron | Domain of unknown function DUF296 |

| SNP IDs | Chromosomes | Physical positions (bp) | SNPs | Gene accession IDs | Sequence components of genes | Putative functions |
|---|---|---|---|---|---|---|
| CWSNP6705 | Ca-Kabuli-Chr7 | 39680222 | [G/A] | Ca19607 | Intron | Domain of unknown function DUF296 |
| CWSNP6706 | Ca-Kabuli-Chr7 | 39944444 | [T/C] | Ca25106 | Synonymous-CDS | Mitochondrial carrier protein |
| CWSNP6707 | Ca-Kabuli-Chr7 | 40092198 | [G/A] | Ca24269 | Non-Synonymous-CDS | - |
| CWSNP6708 | Ca-Kabuli-Chr7 | 40092181 | [C/T] | Ca24269 | Non-Synonymous-CDS | - |
| CWSNP6709 | Ca-Kabuli-Chr7 | 40092218 | [A/T] | Ca24269 | Non-Synonymous-CDS | - |
| CWSNP6710 | Ca-Kabuli-Chr7 | 40092213 | [G/A] | Ca24269 | Non-Synonymous-CDS | - |
| CWSNP6711 | Ca-Kabuli-Chr7 | 40092201 | [G/A] | Ca24269 | Non-Synonymous-CDS | - |
| CWSNP6712 | Ca-Kabuli-Chr7 | 40092211 | [C/T] | Ca24269 | Non-Synonymous-CDS | - |
| CWSNP6713 | Ca-Kabuli-Chr7 | 40092214 | [C/G] | Ca24269 | Non-Synonymous-CDS | - |
| CWSNP6714 | Ca-Kabuli-Chr7 | 40092229 | [C/A] | Ca24269 | Non-Synonymous-CDS | - |
| CWSNP6715 | Ca-Kabuli-Chr7 | 40092237 | [C/T] | Ca24269 | Synonymous-CDS | - |
| CWSNP6716 | Ca-Kabuli-Chr7 | 40092250 | [A/C] | Ca24269 | Non-Synonymous-CDS | - |
| CWSNP6717 | Ca-Kabuli-Chr7 | 40092253 | [C/T] | Ca24269 | Non-Synonymous-CDS | - |
| CWSNP6718 | Ca-Kabuli-Chr7 | 40092256 | [T/C] | Ca24269 | Non-Synonymous-CDS | - |
| CWSNP6719 | Ca-Kabuli-Chr7 | 40092267 | [G/C] | Ca24269 | Non-Synonymous-CDS | - |
| CWSNP6720 | Ca-Kabuli-Chr7 | 40183449 | [C/A] | - | Intergenic | - |

| SNP IDs | Chromosomes | Physical positions (bp) | SNPs | Gene accession IDs | Sequence components of genes | Putative functions |
|---|---|---|---|---|---|---|
| CWSNP6721 | Ca-Kabuli-Chr7 | 40183452 | [A/C] | - | Intergenic | - |
| CWSNP6722 | Ca-Kabuli-Chr7 | 40242765 | [A/G] | Ca24261 | Intron | RNA recognition motif domain |
| CWSNP6723 | Ca-Kabuli-Chr7 | 40323213 | [C/A] | - | Intergenic | - |
| CWSNP6724 | Ca-Kabuli-Chr7 | 40323251 | [T/A] | - | Intergenic | - |
| CWSNP6725 | Ca-Kabuli-Chr7 | 40429548 | [A/G] | - | DRR | - |
| CWSNP6726 | Ca-Kabuli-Chr7 | 40429552 | [C/T] | - | DRR | - |
| CWSNP6727 | Ca-Kabuli-Chr7 | 40429564 | [G/T] | - | DRR | - |
| CWSNP6728 | Ca-Kabuli-Chr7 | 40429573 | [G/T] | - | DRR | - |
| CWSNP6729 | Ca-Kabuli-Chr7 | 40429629 | [G/T] | - | DRR | - |
| CWSNP6730 | Ca-Kabuli-Chr7 | 40429621 | [G/T] | - | DRR | - |
| CWSNP6731 | Ca-Kabuli-Chr7 | 40429604 | [C/T] | - | DRR | - |
| CWSNP6732 | Ca-Kabuli-Chr7 | 40429600 | [C/T] | - | DRR | - |
| CWSNP6733 | Ca-Kabuli-Chr7 | 40429596 | [G/A] | - | DRR | - |
| CWSNP6734 | Ca-Kabuli-Chr7 | 40429567 | [G/A] | - | DRR | - |
| CWSNP6735 | Ca-Kabuli-Chr7 | 40456574 | [T/C] | - | DRR | - |
| CWSNP6736 | Ca-Kabuli-Chr7 | 40889596 | [A/C] | - | DRR | - |

| SNP IDs | Chromosomes | Physical positions (bp) | SNPs | Gene accession IDs | Sequence components of genes | Putative functions |
|---|---|---|---|---|---|---|
| CWSNP6737 | Ca-Kabuli-Chr7 | 40889598 | [C/G] | - | DRR | - |
| CWSNP6738 | Ca-Kabuli-Chr7 | 40889632 | [C/T] | - | DRR | - |
| CWSNP6739 | Ca-Kabuli-Chr7 | 40889633 | [G/A] | - | DRR | - |
| CWSNP6740 | Ca-Kabuli-Chr7 | 40889640 | [G/A] | - | DRR | - |
| CWSNP6741 | Ca-Kabuli-Chr7 | 40889641 | [C/T] | - | DRR | - |
| CWSNP6742 | Ca-Kabuli-Chr7 | 40889643 | [G/A] | - | DRR | - |
| CWSNP6743 | Ca-Kabuli-Chr7 | 40889673 | [C/A] | - | DRR | - |
| CWSNP6744 | Ca-Kabuli-Chr7 | 40889670 | [C/T] | - | DRR | - |
| CWSNP6745 | Ca-Kabuli-Chr7 | 40889723 | [C/G] | - | DRR | - |
| CWSNP6746 | Ca-Kabuli-Chr7 | 40889684 | [C/T] | - | DRR | - |
| CWSNP6747 | Ca-Kabuli-Chr7 | 41282883 | [A/T] | - | Intergenic | - |
| CWSNP6748 | Ca-Kabuli-Chr7 | 41282961 | [G/T] | - | Intergenic | - |
| CWSNP6749 | Ca-Kabuli-Chr7 | 41282960 | [A/C] | - | Intergenic | - |
| CWSNP6750 | Ca-Kabuli-Chr7 | 41282957 | [G/A] | - | Intergenic | - |
| CWSNP6751 | Ca-Kabuli-Chr7 | 41325886 | [A/G] | Ca18002 | Synonymous-CDS | Orotidine5'-phosphate decarboxylase domain |
| CWSNP6752 | Ca-Kabuli-Chr7 | 41387475 | [A/C] | Ca17996 | Non-Synonymous-CDS | WD40 repeat |

| SNP IDs | Chromosomes | Physical positions (bp) | SNPs | Gene accession IDs | Sequence components of genes | Putative functions |
|---|---|---|---|---|---|---|
| CWSNP6753 | Ca-Kabuli-Chr7 | 41657180 | [G/T] | Ca17988 | Intron | RNA polymerase,alpha subunit |
| CWSNP6754 | Ca-Kabuli-Chr7 | 41657241 | [T/C] | Ca17988 | Non-Synonymous-CDS | RNA polymerase,alpha subunit |
| CWSNP6755 | Ca-Kabuli-Chr7 | 41821410 | [T/C] | - | Intergenic | - |
| CWSNP6756 | Ca-Kabuli-Chr7 | 42272939 | [G/C] | - | DRR | - |
| CWSNP6757 | Ca-Kabuli-Chr7 | 42355002 | [T/G] | - | Intergenic | - |
| CWSNP6758 | Ca-Kabuli-Chr7 | 42354938 | [C/T] | - | Intergenic | - |
| CWSNP6759 | Ca-Kabuli-Chr7 | 42354999 | [C/T] | - | Intergenic | - |
| CWSNP6760 | Ca-Kabuli-Chr7 | 42355011 | [G/T] | - | Intergenic | - |
| CWSNP6761 | Ca-Kabuli-Chr7 | 43210821 | [A/T] | - | Intergenic | - |
| CWSNP6762 | Ca-Kabuli-Chr7 | 44232239 | [G/A] | Ca21369 | Synonymous-CDS | - |
| CWSNP6763 | Ca-Kabuli-Chr7 | 44232204 | [T/A] | Ca21369 | Synonymous-CDS | - |
| CWSNP6764 | Ca-Kabuli-Chr7 | 44232221 | [C/T] | Ca21369 | Non-Synonymous-CDS | - |
| CWSNP6765 | Ca-Kabuli-Chr7 | 44232266 | [C/T] | Ca21369 | Non-Synonymous-CDS | - |
| CWSNP6766 | Ca-Kabuli-Chr7 | 44540553 | [T/G] | - | Intergenic | - |
| CWSNP6767 | Ca-Kabuli-Chr7 | 44540596 | [C/A] | - | Intergenic | - |
| CWSNP6768 | Ca-Kabuli-Chr7 | 44540629 | [G/A] | - | Intergenic | - |

| **SNP IDs** | **Chromosomes** | **Physical positions (bp)** | **SNPs** | **Gene accession IDs** | **Sequence components of genes** | **Putative functions** |
|---|---|---|---|---|---|---|
| CWSNP6769 | Ca-Kabuli-Chr7 | 44540616 | [C/A] | - | Intergenic | - |
| CWSNP6770 | Ca-Kabuli-Chr7 | 44612651 | [C/A] | Ca15697 | Non-Synonymous-CDS | Ethyleneinsensitive3 |
| CWSNP6771 | Ca-Kabuli-Chr7 | 44747668 | [C/T] | Ca15701 | Synonymous-CDS | - |
| CWSNP6772 | Ca-Kabuli-Chr7 | 44765450 | [C/T] | Ca15703 | Synonymous-CDS | Transposase,Ptta/En/Spm,plant |
| CWSNP6773 | Ca-Kabuli-Chr7 | 44765522 | [A/C] | Ca15703 | Synonymous-CDS | Transposase,Ptta/En/Spm,plant |
| CWSNP6774 | Ca-Kabuli-Chr7 | 44877324 | [A/C] | Ca15708 | Non-Synonymous-CDS | Zinc finger,C2H2-type |
| CWSNP6775 | Ca-Kabuli-Chr7 | 44926768 | [C/T] | - | Intergenic | - |
| CWSNP6776 | Ca-Kabuli-Chr7 | 44926783 | [C/A] | - | Intergenic | - |
| CWSNP6777 | Ca-Kabuli-Chr7 | 45280659 | [T/A] | - | Intergenic | - |
| CWSNP6778 | Ca-Kabuli-Chr7 | 45936581 | [C/A] | Ca15757 | Non-Synonymous-CDS | Dedicatorofcytokinesis |
| CWSNP6779 | Ca-Kabuli-Chr7 | 46053109 | [G/A] | Ca15758 | Non-Synonymous-CDS | PeptidaseC48,SUMO/Sentrin/Ubl1 |
| CWSNP6780 | Ca-Kabuli-Chr7 | 46485934 | [A/G] | Ca22756 | Intron | - |
| CWSNP6781 | Ca-Kabuli-Chr7 | 46747255 | [A/C] | - | Intergenic | - |
| CWSNP6782 | Ca-Kabuli-Chr7 | 46747283 | [T/C] | - | Intergenic | - |
| CWSNP6783 | Ca-Kabuli-Chr7 | 46747290 | [G/T] | - | Intergenic | - |
| CWSNP6784 | Ca-Kabuli-Chr7 | 46747295 | [G/C] | - | Intergenic | - |

| SNP IDs | Chromosomes | Physical positions (bp) | SNPs | Gene accession IDs | Sequence components of genes | Putative functions |
|---|---|---|---|---|---|---|
| CWSNP6785 | Ca-Kabuli-Chr7 | 46747309 | [C/T] | - | Intergenic | - |
| CWSNP6786 | Ca-Kabuli-Chr7 | 46747330 | [G/A] | - | Intergenic | - |
| CWSNP6787 | Ca-Kabuli-Chr7 | 46826036 | [T/A] | Ca19475 | Synonymous-CDS | Glycosidehydrolase,family18,catalytic domain |
| CWSNP6788 | Ca-Kabuli-Chr7 | 46826030 | [T/C] | Ca19475 | Synonymous-CDS | Glycosidehydrolase,family18,catalytic domain |
| CWSNP6789 | Ca-Kabuli-Chr7 | 46826028 | [C/A] | Ca19475 | Non-Synonymous-CDS | Glycosidehydrolase,family18,catalytic domain |
| CWSNP6790 | Ca-Kabuli-Chr7 | 46826020 | [G/A] | Ca19475 | Non-Synonymous-CDS | Glycosidehydrolase,family18,catalytic domain |
| CWSNP6791 | Ca-Kabuli-Chr7 | 46826015 | [G/A] | Ca19475 | Synonymous-CDS | Glycosidehydrolase,family18,catalytic domain |
| CWSNP6792 | Ca-Kabuli-Chr7 | 46826008 | [A/T] | Ca19475 | Non-Synonymous-CDS | Glycosidehydrolase,family18,catalytic domain |
| CWSNP6793 | Ca-Kabuli-Chr7 | 46825997 | [A/G] | Ca19475 | Synonymous-CDS | Glycosidehydrolase,family18,catalytic domain |
| CWSNP6794 | Ca-Kabuli-Chr7 | 46825992 | [T/G] | Ca19475 | Non-Synonymous-CDS | Glycosidehydrolase,family18,catalytic domain |
| CWSNP6795 | Ca-Kabuli-Chr7 | 46825985 | [T/G] | Ca19475 | Synonymous-CDS | Glycosidehydrolase,family18,catalytic domain |
| CWSNP6796 | Ca-Kabuli-Chr7 | 46825957 | [A/T] | Ca19475 | Non-Synonymous-CDS | Glycosidehydrolase,family18,catalytic domain |
| CWSNP6797 | Ca-Kabuli-Chr7 | 46825952 | [A/G] | Ca19475 | Synonymous-CDS | Glycosidehydrolase,family18,catalytic domain |
| CWSNP6798 | Ca-Kabuli-Chr7 | 47127331 | [T/G] | - | Intergenic | - |
| CWSNP6799 | Ca-Kabuli-Chr7 | 47127342 | [T/G] | - | Intergenic | - |
| CWSNP6800 | Ca-Kabuli-Chr7 | 47127343 | [C/T] | - | Intergenic | - |

| SNP IDs | Chromosomes | Physical positions (bp) | SNPs | Gene accession IDs | Sequence components of genes | Putative functions |
|---|---|---|---|---|---|---|
| CWSNP6801 | Ca-Kabuli-Chr7 | 47335940 | [G/A] | - | Intergenic | - |
| CWSNP6802 | Ca-Kabuli-Chr7 | 47335916 | [C/G] | - | Intergenic | - |
| CWSNP6803 | Ca-Kabuli-Chr7 | 47524173 | [A/C] | Ca19499 | Non-Synonymous-CDS | Protein kinase, catalytic domain |
| CWSNP6804 | Ca-Kabuli-Chr7 | 47694706 | [A/C] | - | Intergenic | - |
| CWSNP6805 | Ca-Kabuli-Chr7 | 47839952 | [G/T] | Ca22546 | Non-Synonymous-CDS | Ubiquitin-conjugating enzyme,E2 |
| CWSNP6806 | Ca-Kabuli-Chr7 | 47840092 | [A/C] | Ca22546 | Synonymous-CDS | Ubiquitin-conjugating enzyme,E2 |
| CWSNP6807 | Ca-Kabuli-Chr7 | 48200865 | [A/G] | - | Intergenic | - |
| CWSNP6808 | Ca-Kabuli-Chr7 | 48200822 | [G/A] | - | Intergenic | - |
| CWSNP6809 | Ca-Kabuli-Chr7 | 48200823 | [G/A] | - | Intergenic | - |
| CWSNP6810 | Ca-Kabuli-Chr7 | 48200848 | [T/G] | - | Intergenic | - |
| CWSNP6811 | Ca-Kabuli-Chr8 | 54095 | [A/C] | Ca11935 | Synonymous-CDS | Heat shock protein DnaJ,N-terminal |
| CWSNP6812 | Ca-Kabuli-Chr8 | 281622 | [G/A] | - | Intergenic | - |
| CWSNP6813 | Ca-Kabuli-Chr8 | 281630 | [A/C] | - | Intergenic | - |
| CWSNP6814 | Ca-Kabuli-Chr8 | 356295 | [A/T] | Ca11899 | Intron | HEAT |
| CWSNP6815 | Ca-Kabuli-Chr8 | 499819 | [A/C] | Ca11887 | Intron | Protein phosphatase2C,manganese/magnesiumaspartate binding site |
| CWSNP6816 | Ca-Kabuli-Chr8 | 560746 | [A/G] | Ca11882 | Intron | Cystathioninebeta-synthase,core |

| SNP IDs | Chromosomes | Physical positions (bp) | SNPs | Gene accession IDs | Sequence components of genes | Putative functions |
|---|---|---|---|---|---|---|
| CWSNP6817 | Ca-Kabuli-Chr8 | 566094 | [A/G] | - | DRR | - |
| CWSNP6818 | Ca-Kabuli-Chr8 | 614324 | [G/A] | - | Intergenic | - |
| CWSNP6819 | Ca-Kabuli-Chr8 | 614301 | [C/A] | - | Intergenic | - |
| CWSNP6820 | Ca-Kabuli-Chr8 | 619605 | [T/C] | - | Intergenic | - |
| CWSNP6821 | Ca-Kabuli-Chr8 | 634862 | [C/T] | - | Intergenic | - |
| CWSNP6822 | Ca-Kabuli-Chr8 | 649491 | [A/C] | Ca11866 | Synonymous-CDS | Protein of unknown function DUF1423,plant |
| CWSNP6823 | Ca-Kabuli-Chr8 | 660610 | [T/G] | - | Intergenic | - |
| CWSNP6824 | Ca-Kabuli-Chr8 | 666294 | [G/A] | - | DRR | - |
| CWSNP6825 | Ca-Kabuli-Chr8 | 666288 | [A/T] | - | DRR | - |
| CWSNP6826 | Ca-Kabuli-Chr8 | 666284 | [T/A] | - | DRR | - |
| CWSNP6827 | Ca-Kabuli-Chr8 | 666274 | [T/G] | - | DRR | - |
| CWSNP6828 | Ca-Kabuli-Chr8 | 673820 | [A/G] | Ca11861 | Non-Synonymous-CDS | KIP1-like |
| CWSNP6829 | Ca-Kabuli-Chr8 | 701803 | [C/T] | Ca11858 | Intron | Protein kinase, catalytic domain |
| CWSNP6830 | Ca-Kabuli-Chr8 | 701811 | [T/G] | Ca11858 | Intron | Protein kinase, catalytic domain |
| CWSNP6831 | Ca-Kabuli-Chr8 | 701837 | [G/A] | Ca11858 | Intron | Protein kinase, catalytic domain |
| CWSNP6832 | Ca-Kabuli-Chr8 | 706514 | [T/C] | - | DRR | - |

| SNP IDs | Chromosomes | Physical positions (bp) | SNPs | Gene accession IDs | Sequence components of genes | Putative functions |
|---|---|---|---|---|---|---|
| CWSNP6833 | Ca-Kabuli-Chr8 | 706482 | [G/C] | - | DRR | - |
| CWSNP6834 | Ca-Kabuli-Chr8 | 706614 | [C/T] | - | DRR | - |
| CWSNP6835 | Ca-Kabuli-Chr8 | 706793 | [A/G] | - | DRR | - |
| CWSNP6836 | Ca-Kabuli-Chr8 | 706800 | [G/A] | - | DRR | - |
| CWSNP6837 | Ca-Kabuli-Chr8 | 709063 | [T/C] | - | Intergenic | - |
| CWSNP6838 | Ca-Kabuli-Chr8 | 709094 | [T/C] | - | Intergenic | - |
| CWSNP6839 | Ca-Kabuli-Chr8 | 709124 | [A/T] | - | Intergenic | - |
| CWSNP6840 | Ca-Kabuli-Chr8 | 714967 | [A/G] | - | URR | - |
| CWSNP6841 | Ca-Kabuli-Chr8 | 714974 | [G/A] | - | URR | - |
| CWSNP6842 | Ca-Kabuli-Chr8 | 822302 | [A/G] | Ca11839 | Non-Synonymous-CDS | Zinc finger,PHD-type |
| CWSNP6843 | Ca-Kabuli-Chr8 | 875236 | [T/G] | Ca11831 | Intron | PeptidaseM16,C-terminal |
| CWSNP6844 | Ca-Kabuli-Chr8 | 894184 | [C/A] | - | URR | - |
| CWSNP6845 | Ca-Kabuli-Chr8 | 898089 | [C/T] | - | URR | - |
| CWSNP6846 | Ca-Kabuli-Chr8 | 924715 | [A/G] | - | DRR | - |
| CWSNP6847 | Ca-Kabuli-Chr8 | 1011320 | [G/T] | - | URR | - |
| CWSNP6848 | Ca-Kabuli-Chr8 | 1194886 | [G/A] | Ca15060 | Intron | Alpha/beta hydrolase fold-1 |

| SNP IDs | Chromosomes | Physical positions (bp) | SNPs | Gene accession IDs | Sequence components of genes | Putative functions |
|---|---|---|---|---|---|---|
| CWSNP6849 | Ca-Kabuli-Chr8 | 1241739 | [C/T] | - | URR | - |
| CWSNP6850 | Ca-Kabuli-Chr8 | 1534461 | [A/C] | - | DRR | - |
| CWSNP6851 | Ca-Kabuli-Chr8 | 1544916 | [C/T] | - | Intergenic | - |
| CWSNP6852 | Ca-Kabuli-Chr8 | 1573877 | [G/T] | Ca15012 | Non-Synonymous-CDS | ATPase,AAA+type,core |
| CWSNP6853 | Ca-Kabuli-Chr8 | 1708860 | [T/C] | Ca02436 | Synonymous-CDS | - |
| CWSNP6854 | Ca-Kabuli-Chr8 | 1782034 | [C/G] | - | DRR | - |
| CWSNP6855 | Ca-Kabuli-Chr8 | 1794755 | [C/A] | Ca02426 | Non-Synonymous-CDS | ATPase,P-type,K/Mg/Cd/Cu/Zn/Na/Ca/Na/H-transporter |
| CWSNP6856 | Ca-Kabuli-Chr8 | 1794756 | [G/T] | Ca02426 | Synonymous-CDS | ATPase,P-type,K/Mg/Cd/Cu/Zn/Na/Ca/Na/H-transporter |
| CWSNP6857 | Ca-Kabuli-Chr8 | 1794759 | [C/T] | Ca02426 | Synonymous-CDS | ATPase,P-type,K/Mg/Cd/Cu/Zn/Na/Ca/Na/H-transporter |
| CWSNP6858 | Ca-Kabuli-Chr8 | 1817388 | [T/C] | - | DRR | - |
| CWSNP6859 | Ca-Kabuli-Chr8 | 1932975 | [T/C] | - | URR | - |
| CWSNP6860 | Ca-Kabuli-Chr8 | 1932981 | [T/C] | - | URR | - |
| CWSNP6861 | Ca-Kabuli-Chr8 | 1932990 | [A/C] | - | URR | - |
| CWSNP6862 | Ca-Kabuli-Chr8 | 1932993 | [C/T] | - | URR | - |
| CWSNP6863 | Ca-Kabuli-Chr8 | 1932997 | [G/T] | - | URR | - |
| CWSNP6864 | Ca-Kabuli-Chr8 | 1933026 | [G/A] | - | URR | - |

| SNP IDs | Chromosomes | Physical positions (bp) | SNPs | Gene accession IDs | Sequence components of genes | Putative functions |
|---|---|---|---|---|---|---|
| CWSNP6865 | Ca-Kabuli-Chr8 | 1933064 | [G/C] | - | URR | - |
| CWSNP6866 | Ca-Kabuli-Chr8 | 1933053 | [G/A] | - | URR | - |
| CWSNP6867 | Ca-Kabuli-Chr8 | 1933052 | [T/C] | - | URR | - |
| CWSNP6868 | Ca-Kabuli-Chr8 | 1933039 | [G/T] | - | URR | - |
| CWSNP6869 | Ca-Kabuli-Chr8 | 1933035 | [T/A] | - | URR | - |
| CWSNP6870 | Ca-Kabuli-Chr8 | 2088892 | [T/G] | Ca02391 | Non-Synonymous-CDS | Cellcycleregulatedmicrotubuleassociatedprotein |
| CWSNP6871 | Ca-Kabuli-Chr8 | 2090852 | [A/G] | - | Intergenic | - |
| CWSNP6872 | Ca-Kabuli-Chr8 | 2095607 | [A/G] | - | DRR | - |
| CWSNP6873 | Ca-Kabuli-Chr8 | 2482834 | [A/G] | - | DRR | - |
| CWSNP6874 | Ca-Kabuli-Chr8 | 2524188 | [A/G] | - | DRR | - |
| CWSNP6875 | Ca-Kabuli-Chr8 | 2524148 | [A/G] | - | DRR | - |
| CWSNP6876 | Ca-Kabuli-Chr8 | 2543156 | [A/G] | Ca02330 | Non-Synonymous-CDS | - |
| CWSNP6877 | Ca-Kabuli-Chr8 | 2604759 | [C/T] | Ca02321 | Intron | Polycombprotein,VEFS-Box |
| CWSNP6878 | Ca-Kabuli-Chr8 | 2621973 | [T/G] | Ca02319 | Synonymous-CDS | Monooxygenase,FAD-binding |
| CWSNP6879 | Ca-Kabuli-Chr8 | 2722673 | [C/A] | - | DRR | - |
| CWSNP6880 | Ca-Kabuli-Chr8 | 2827984 | [T/C] | Ca02297 | Intron | Pleckstrin homology domain |

| SNP IDs | Chromosomes | Physical positions (bp) | SNPs | Gene accession IDs | Sequence components of genes | Putative functions |
|---|---|---|---|---|---|---|
| CWSNP6881 | Ca-Kabuli-Chr8 | 2838179 | [T/A] | Ca02297 | Intron | Pleckstrin homology domain |
| CWSNP6882 | Ca-Kabuli-Chr8 | 2871095 | [G/A] | Ca02297 | Intron | Pleckstrin homology domain |
| CWSNP6883 | Ca-Kabuli-Chr8 | 2871064 | [G/A] | Ca02297 | Intron | Pleckstrin homology domain |
| CWSNP6884 | Ca-Kabuli-Chr8 | 2888353 | [G/T] | Ca02296 | Intron | PeptidaseS16,Lonprotease,C-terminal |
| CWSNP6885 | Ca-Kabuli-Chr8 | 2943631 | [G/T] | - | DRR | - |
| CWSNP6886 | Ca-Kabuli-Chr8 | 3041947 | [A/G] | Ca02278 | Synonymous-CDS | Protein of unknown function DUF947 |
| CWSNP6887 | Ca-Kabuli-Chr8 | 3050452 | [T/C] | - | URR | - |
| CWSNP6888 | Ca-Kabuli-Chr8 | 3171324 | [C/T] | - | Intergenic | - |
| CWSNP6889 | Ca-Kabuli-Chr8 | 3250707 | [C/T] | - | DRR | - |
| CWSNP6890 | Ca-Kabuli-Chr8 | 3269088 | [A/C] | Ca02251 | Synonymous-CDS | Homeobox |
| CWSNP6891 | Ca-Kabuli-Chr8 | 3269085 | [A/C] | Ca02251 | Synonymous-CDS | Homeobox |
| CWSNP6892 | Ca-Kabuli-Chr8 | 3297410 | [T/C] | Ca02249 | Non-Synonymous-CDS | SANT domain, DNA binding |
| CWSNP6893 | Ca-Kabuli-Chr8 | 3394931 | [T/C] | - | Intergenic | - |
| CWSNP6894 | Ca-Kabuli-Chr8 | 3539690 | [A/G] | - | Intergenic | - |
| CWSNP6895 | Ca-Kabuli-Chr8 | 3539745 | [T/C] | - | Intergenic | - |
| CWSNP6896 | Ca-Kabuli-Chr8 | 3730369 | [A/C] | - | DRR | - |

| SNP IDs | Chromosomes | Physical positions (bp) | SNPs | Gene accession IDs | Sequence components of genes | Putative functions |
|---|---|---|---|---|---|---|
| CWSNP6897 | Ca-Kabuli-Chr8 | 3837306 | [G/C] | - | URR | - |
| CWSNP6898 | Ca-Kabuli-Chr8 | 3951767 | [C/A] | - | DRR | - |
| CWSNP6899 | Ca-Kabuli-Chr8 | 3951768 | [A/G] | - | DRR | - |
| CWSNP6900 | Ca-Kabuli-Chr8 | 4042166 | [A/C] | - | URR | - |
| CWSNP6901 | Ca-Kabuli-Chr8 | 4042424 | [G/A] | - | URR | - |
| CWSNP6902 | Ca-Kabuli-Chr8 | 4073528 | [C/T] | Ca02163 | Non-Synonymous-CDS | Zinc finger,RING-type |
| CWSNP6903 | Ca-Kabuli-Chr8 | 4073603 | [A/G] | Ca02163 | Non-Synonymous-CDS | Zinc finger,RING-type |
| CWSNP6904 | Ca-Kabuli-Chr8 | 4106644 | [C/T] | - | URR | - |
| CWSNP6905 | Ca-Kabuli-Chr8 | 4172368 | [T/C] | Ca02150 | Synonymous-CDS | Forkhead-associated (FHA) domain |
| CWSNP6906 | Ca-Kabuli-Chr8 | 4198214 | [A/C] | Ca02148 | Non-Synonymous-CDS | Splicing factor,suppressor of white apricot |
| CWSNP6907 | Ca-Kabuli-Chr8 | 4344459 | [C/A] | - | DRR | - |
| CWSNP6908 | Ca-Kabuli-Chr8 | 4346749 | [A/G] | - | Intergenic | - |
| CWSNP6909 | Ca-Kabuli-Chr8 | 4347535 | [A/C] | - | Intergenic | - |
| CWSNP6910 | Ca-Kabuli-Chr8 | 4351751 | [G/A] | - | URR | - |
| CWSNP6911 | Ca-Kabuli-Chr8 | 4406956 | [C/G] | Ca02125 | Synonymous-CDS | Lateral organ boundaries, LOB |
| CWSNP6912 | Ca-Kabuli-Chr8 | 4484606 | [T/A] | Ca02117 | Intron | Zinc finger,LIM-type |

| SNP IDs | Chromosomes | Physical positions (bp) | SNPs | Gene accession IDs | Sequence components of genes | Putative functions |
|---|---|---|---|---|---|---|
| CWSNP6913 | Ca-Kabuli-Chr8 | 4512311 | [T/C] | - | DRR | - |
| CWSNP6914 | Ca-Kabuli-Chr8 | 4611094 | [C/T] | Ca02096 | Non-Synonymous-CDS | SET domain |
| CWSNP6915 | Ca-Kabuli-Chr8 | 4773205 | [A/C] | - | Intergenic | - |
| CWSNP6916 | Ca-Kabuli-Chr8 | 4858262 | [C/A] | Ca02071 | Non-Synonymous-CDS | NADPH-dependentFMNreductase |
| CWSNP6917 | Ca-Kabuli-Chr8 | 4899983 | [G/A] | - | DRR | - |
| CWSNP6918 | Ca-Kabuli-Chr8 | 4980949 | [A/T] | - | Intergenic | - |
| CWSNP6919 | Ca-Kabuli-Chr8 | 4980916 | [C/T] | - | Intergenic | - |
| CWSNP6920 | Ca-Kabuli-Chr8 | 5115522 | [G/C] | - | DRR | - |
| CWSNP6921 | Ca-Kabuli-Chr8 | 5134706 | [A/C] | - | Intergenic | - |
| CWSNP6922 | Ca-Kabuli-Chr8 | 5184227 | [C/G] | Ca02036 | Non-Synonymous-CDS | Sugar/inositol transporter |
| CWSNP6923 | Ca-Kabuli-Chr8 | 5197004 | [G/T] | - | DRR | - |
| CWSNP6924 | Ca-Kabuli-Chr8 | 5390602 | [T/C] | - | DRR | - |
| CWSNP6925 | Ca-Kabuli-Chr8 | 5399503 | [G/A] | - | URR | - |
| CWSNP6926 | Ca-Kabuli-Chr8 | 5400188 | [A/G] | - | DRR | - |
| CWSNP6927 | Ca-Kabuli-Chr8 | 5412489 | [T/C] | Ca02004 | Synonymous-CDS | Protein of unknown function DUF702 |
| CWSNP6928 | Ca-Kabuli-Chr8 | 5487769 | [G/A] | - | Intergenic | - |

| SNP IDs | Chromosomes | Physical positions (bp) | SNPs | Gene accession IDs | Sequence components of genes | Putative functions |
|---|---|---|---|---|---|---|
| CWSNP6929 | Ca-Kabuli-Chr8 | 5538696 | [A/C] | Ca01989 | Intron | Protein of unknown function DUF726 |
| CWSNP6930 | Ca-Kabuli-Chr8 | 5538695 | [T/C] | Ca01989 | Intron | Protein of unknown function DUF726 |
| CWSNP6931 | Ca-Kabuli-Chr8 | 5538683 | [T/C] | Ca01989 | Intron | Protein of unknown function DUF726 |
| CWSNP6932 | Ca-Kabuli-Chr8 | 5870664 | [T/C] | Ca01965 | Non-Synonymous-CDS | Armadillo |
| CWSNP6933 | Ca-Kabuli-Chr8 | 5935435 | [A/C] | Ca01957 | Synonymous-CDS | Senescence/spartin-associated |
| CWSNP6934 | Ca-Kabuli-Chr8 | 6084594 | [G/A] | - | DRR | - |
| CWSNP6935 | Ca-Kabuli-Chr8 | 6192944 | [T/G] | Ca01927 | Synonymous-CDS | Pentatrico peptide repeat |
| CWSNP6936 | Ca-Kabuli-Chr8 | 6193078 | [G/A] | Ca01927 | Non-Synonymous-CDS | Pentatrico peptide repeat |
| CWSNP6937 | Ca-Kabuli-Chr8 | 6372230 | [G/A] | - | Intergenic | - |
| CWSNP6938 | Ca-Kabuli-Chr8 | 6664509 | [T/A] | - | DRR | - |
| CWSNP6939 | Ca-Kabuli-Chr8 | 6748294 | [T/C] | Ca10708 | Non-Synonymous-CDS | Sugar/inositol transporter |
| CWSNP6940 | Ca-Kabuli-Chr8 | 6780592 | [T/G] | Ca10705 | Non-Synonymous-CDS | ATPase-like,ATP-binding domain |
| CWSNP6941 | Ca-Kabuli-Chr8 | 6942651 | [G/T] | Ca10698 | Intron | ARID/BRIGHTDNA-binding domain |
| CWSNP6942 | Ca-Kabuli-Chr8 | 6987609 | [G/T] | Ca10694 | Non-Synonymous-CDS | PeptidaseS8/S53,subtilisin/kexin/sedolisin |
| CWSNP6943 | Ca-Kabuli-Chr8 | 6987615 | [A/C] | Ca10694 | Non-Synonymous-CDS | PeptidaseS8/S53,subtilisin/kexin/sedolisin |
| CWSNP6944 | Ca-Kabuli-Chr8 | 7041901 | [A/C] | - | URR | - |

| SNP IDs | Chromosomes | Physical positions (bp) | SNPs | Gene accession IDs | Sequence components of genes | Putative functions |
|---|---|---|---|---|---|---|
| CWSNP6945 | Ca-Kabuli-Chr8 | 7162826 | [T/G] | - | Intergenic | - |
| CWSNP6946 | Ca-Kabuli-Chr8 | 7174066 | [C/T] | Ca10671 | Synonymous-CDS | F-boxdomain,cyclin-like |
| CWSNP6947 | Ca-Kabuli-Chr8 | 7175086 | [A/C] | Ca10671 | Intron | F-boxdomain,cyclin-like |
| CWSNP6948 | Ca-Kabuli-Chr8 | 7357621 | [A/G] | - | URR | - |
| CWSNP6949 | Ca-Kabuli-Chr8 | 7357583 | [T/C] | - | URR | - |
| CWSNP6950 | Ca-Kabuli-Chr8 | 7563385 | [C/G] | - | Intergenic | - |
| CWSNP6951 | Ca-Kabuli-Chr8 | 7563370 | [T/C] | - | Intergenic | - |
| CWSNP6952 | Ca-Kabuli-Chr8 | 7563373 | [T/A] | - | Intergenic | - |
| CWSNP6953 | Ca-Kabuli-Chr8 | 7665291 | [T/C] | - | Intergenic | - |
| CWSNP6954 | Ca-Kabuli-Chr8 | 7694747 | [A/G] | - | DRR | - |
| CWSNP6955 | Ca-Kabuli-Chr8 | 7733573 | [A/C] | - | DRR | - |
| CWSNP6956 | Ca-Kabuli-Chr8 | 7733574 | [G/A] | - | DRR | - |
| CWSNP6957 | Ca-Kabuli-Chr8 | 7733670 | [C/T] | - | DRR | - |
| CWSNP6958 | Ca-Kabuli-Chr8 | 7733647 | [G/T] | - | DRR | - |
| CWSNP6959 | Ca-Kabuli-Chr8 | 7776466 | [G/A] | - | URR | - |
| CWSNP6960 | Ca-Kabuli-Chr8 | 7776592 | [T/C] | - | URR | - |

| SNP IDs | Chromosomes | Physical positions (bp) | SNPs | Gene accession IDs | Sequence components of genes | Putative functions |
|---|---|---|---|---|---|---|
| CWSNP6961 | Ca-Kabuli-Chr8 | 7776897 | [A/T] | - | URR | - |
| CWSNP6962 | Ca-Kabuli-Chr8 | 7776934 | [G/T] | - | URR | - |
| CWSNP6963 | Ca-Kabuli-Chr8 | 7776970 | [T/G] | - | URR | - |
| CWSNP6964 | Ca-Kabuli-Chr8 | 7777023 | [G/A] | - | URR | - |
| CWSNP6965 | Ca-Kabuli-Chr8 | 8066006 | [C/A] | - | Intergenic | - |
| CWSNP6966 | Ca-Kabuli-Chr8 | 8066005 | [A/G] | - | Intergenic | - |
| CWSNP6967 | Ca-Kabuli-Chr8 | 8104884 | [T/A] | - | URR | - |
| CWSNP6968 | Ca-Kabuli-Chr8 | 8194232 | [C/A] | Ca11454 | Non-Synonymous-CDS | Aspartyl/Asparaginyl-tRNA synthetase,classIIb |
| CWSNP6969 | Ca-Kabuli-Chr8 | 8194234 | [C/A] | Ca11454 | Non-Synonymous-CDS | Aspartyl/Asparaginyl-tRNA synthetase,classIIb |
| CWSNP6970 | Ca-Kabuli-Chr8 | 8389117 | [C/T] | Ca11471 | Non-Synonymous-CDS | Transcription factor jumonji/aspartyl beta-hydroxylase |
| CWSNP6971 | Ca-Kabuli-Chr8 | 8458383 | [G/A] | - | DRR | - |
| CWSNP6972 | Ca-Kabuli-Chr8 | 8533479 | [G/T] | - | Intergenic | - |
| CWSNP6973 | Ca-Kabuli-Chr8 | 8533978 | [G/A] | - | Intergenic | - |
| CWSNP6974 | Ca-Kabuli-Chr8 | 8560330 | [C/A] | - | Intergenic | - |
| CWSNP6975 | Ca-Kabuli-Chr8 | 8560331 | [C/G] | - | Intergenic | - |
| CWSNP6976 | Ca-Kabuli-Chr8 | 8560332 | [A/C] | - | Intergenic | - |

| SNP IDs | Chromosomes | Physical positions (bp) | SNPs | Gene accession IDs | Sequence components of genes | Putative functions |
|---|---|---|---|---|---|---|
| CWSNP6977 | Ca-Kabuli-Chr8 | 8560326 | [G/T] | - | Intergenic | - |
| CWSNP6978 | Ca-Kabuli-Chr8 | 8661327 | [T/C] | - | Intergenic | - |
| CWSNP6979 | Ca-Kabuli-Chr8 | 8670242 | [T/G] | Ca11498 | Non-Synonymous-CDS | Tetratricopeptide repeat-containing |
| CWSNP6980 | Ca-Kabuli-Chr8 | 8772131 | [C/G] | - | Intergenic | - |
| CWSNP6981 | Ca-Kabuli-Chr8 | 8772124 | [G/A] | - | Intergenic | - |
| CWSNP6982 | Ca-Kabuli-Chr8 | 8853602 | [G/A] | - | DRR | - |
| CWSNP6983 | Ca-Kabuli-Chr8 | 8853664 | [A/G] | - | DRR | - |
| CWSNP6984 | Ca-Kabuli-Chr8 | 8856046 | [C/T] | - | DRR | - |
| CWSNP6985 | Ca-Kabuli-Chr8 | 8856047 | [C/T] | - | DRR | - |
| CWSNP6986 | Ca-Kabuli-Chr8 | 8856150 | [A/G] | - | DRR | - |
| CWSNP6987 | Ca-Kabuli-Chr8 | 8856143 | [G/A] | - | DRR | - |
| CWSNP6988 | Ca-Kabuli-Chr8 | 8864079 | [C/T] | Ca11517 | Synonymous-CDS | VQ |
| CWSNP6989 | Ca-Kabuli-Chr8 | 8864157 | [G/A] | Ca11517 | Synonymous-CDS | VQ |
| CWSNP6990 | Ca-Kabuli-Chr8 | 8888412 | [A/G] | - | URR | - |
| CWSNP6991 | Ca-Kabuli-Chr8 | 8888414 | [G/T] | - | URR | - |
| CWSNP6992 | Ca-Kabuli-Chr8 | 8888426 | [T/C] | - | URR | - |

| SNP IDs | Chromosomes | Physical positions (bp) | SNPs | Gene accession IDs | Sequence components of genes | Putative functions |
|---|---|---|---|---|---|---|
| CWSNP6993 | Ca-Kabuli-Chr8 | 8888476 | [A/C] | - | URR | - |
| CWSNP6994 | Ca-Kabuli-Chr8 | 8904346 | [C/A] | Ca11521 | Intron | Major facilitator superfamily MFS-1 |
| CWSNP6995 | Ca-Kabuli-Chr8 | 8904366 | [A/G] | Ca11521 | Intron | Major facilitator superfamily MFS-1 |
| CWSNP6996 | Ca-Kabuli-Chr8 | 8914018 | [A/C] | Ca11522 | Intron | Starchsynthase,catalytic domain |
| CWSNP6997 | Ca-Kabuli-Chr8 | 9097585 | [A/C] | - | URR | - |
| CWSNP6998 | Ca-Kabuli-Chr8 | 9099798 | [G/T] | - | URR | - |
| CWSNP6999 | Ca-Kabuli-Chr8 | 9100111 | [C/T] | - | URR | - |
| CWSNP7000 | Ca-Kabuli-Chr8 | 9109896 | [T/A] | - | Intergenic | - |
| CWSNP7001 | Ca-Kabuli-Chr8 | 9109907 | [A/T] | - | Intergenic | - |
| CWSNP7002 | Ca-Kabuli-Chr8 | 9109920 | [C/A] | - | Intergenic | - |
| CWSNP7003 | Ca-Kabuli-Chr8 | 9112178 | [G/A] | Ca11534 | Synonymous-CDS | Protein of unknown function DUF1399 |
| CWSNP7004 | Ca-Kabuli-Chr8 | 9112172 | [A/C] | Ca11534 | Synonymous-CDS | Protein of unknown function DUF1399 |
| CWSNP7005 | Ca-Kabuli-Chr8 | 9118582 | [C/T] | - | Intergenic | - |
| CWSNP7006 | Ca-Kabuli-Chr8 | 9118667 | [A/G] | - | Intergenic | - |
| CWSNP7007 | Ca-Kabuli-Chr8 | 9143840 | [C/A] | - | DRR | - |
| CWSNP7008 | Ca-Kabuli-Chr8 | 9151722 | [G/T] | Ca11539 | Synonymous-CDS | PeptidaseM3A/M3B,thimet/oligopeptidaseF |

| SNP IDs | Chromosomes | Physical positions (bp) | SNPs | Gene accession IDs | Sequence components of genes | Putative functions |
|---|---|---|---|---|---|---|
| CWSNP7009 | Ca-Kabuli-Chr8 | 9389347 | [G/A] | - | Intergenic | - |
| CWSNP7010 | Ca-Kabuli-Chr8 | 10004481 | [T/C] | - | Intergenic | - |
| CWSNP7011 | Ca-Kabuli-Chr8 | 10155981 | [A/C] | Ca18411 | Synonymous-CDS | NADH:flavinoxidoreductase/NADHoxidase,N-terminal |
| CWSNP7012 | Ca-Kabuli-Chr8 | 10258892 | [T/G] | Ca18416 | Synonymous-CDS | Zinc finger,PHD-type |
| CWSNP7013 | Ca-Kabuli-Chr8 | 10668422 | [A/C] | - | Intergenic | - |
| CWSNP7014 | Ca-Kabuli-Chr8 | 10733404 | [A/G] | Ca19724 | Synonymous-CDS | Ankyrin repeat |
| CWSNP7015 | Ca-Kabuli-Chr8 | 10733503 | [C/T] | Ca19724 | Synonymous-CDS | Ankyrin repeat |
| CWSNP7016 | Ca-Kabuli-Chr8 | 10733661 | [G/A] | Ca19724 | Synonymous-CDS | Ankyrin repeat |
| CWSNP7017 | Ca-Kabuli-Chr8 | 10733747 | [C/T] | Ca19724 | Non-Synonymous-CDS | Ankyrin repeat |
| CWSNP7018 | Ca-Kabuli-Chr8 | 10763656 | [A/T] | - | DRR | - |
| CWSNP7019 | Ca-Kabuli-Chr8 | 10953767 | [C/T] | - | Intergenic | - |
| CWSNP7020 | Ca-Kabuli-Chr8 | 11015680 | [G/T] | - | Intergenic | - |
| CWSNP7021 | Ca-Kabuli-Chr8 | 11015678 | [C/G] | - | Intergenic | - |
| CWSNP7022 | Ca-Kabuli-Chr8 | 11015666 | [G/C] | - | Intergenic | - |
| CWSNP7023 | Ca-Kabuli-Chr8 | 11066644 | [C/T] | Ca16843 | Synonymous-CDS | Multiantimicrobial extrusion protein |
| CWSNP7024 | Ca-Kabuli-Chr8 | 11066728 | [G/A] | Ca16843 | Intron | Multiantimicrobial extrusion protein |

| SNP IDs | Chromosomes | Physical positions (bp) | SNPs | Gene accession IDs | Sequence components of genes | Putative functions |
|---|---|---|---|---|---|---|
| CWSNP7025 | Ca-Kabuli-Chr8 | 11087143 | [A/G] | - | Intergenic | - |
| CWSNP7026 | Ca-Kabuli-Chr8 | 11087302 | [A/T] | - | Intergenic | - |
| CWSNP7027 | Ca-Kabuli-Chr8 | 11087250 | [T/C] | - | Intergenic | - |
| CWSNP7028 | Ca-Kabuli-Chr8 | 11087247 | [T/C] | - | Intergenic | - |
| CWSNP7029 | Ca-Kabuli-Chr8 | 11186986 | [A/C] | Ca16839 | Synonymous-CDS | Zinc finger,RanBP2-type |
| CWSNP7030 | Ca-Kabuli-Chr8 | 11231812 | [A/G] | Ca16835 | Synonymous-CDS | Protein kinase, catalytic domain |
| CWSNP7031 | Ca-Kabuli-Chr8 | 11240258 | [C/A] | - | URR | - |
| CWSNP7032 | Ca-Kabuli-Chr8 | 11243263 | [A/C] | Ca16834 | Non-Synonymous-CDS | HistoneH1/H5 |
| CWSNP7033 | Ca-Kabuli-Chr8 | 11243256 | [A/C] | Ca16834 | Synonymous-CDS | HistoneH1/H5 |
| CWSNP7034 | Ca-Kabuli-Chr8 | 11266447 | [G/A] | - | DRR | - |
| CWSNP7035 | Ca-Kabuli-Chr8 | 11310460 | [G/A] | - | Intergenic | - |
| CWSNP7036 | Ca-Kabuli-Chr8 | 11310480 | [T/A] | - | Intergenic | - |
| CWSNP7037 | Ca-Kabuli-Chr8 | 11332934 | [C/T] | - | Intergenic | - |
| CWSNP7038 | Ca-Kabuli-Chr8 | 11332940 | [T/C] | - | Intergenic | - |
| CWSNP7039 | Ca-Kabuli-Chr8 | 11333057 | [C/G] | - | Intergenic | - |
| CWSNP7040 | Ca-Kabuli-Chr8 | 11334190 | [A/T] | - | Intergenic | - |

| SNP IDs | Chromosomes | Physical positions (bp) | SNPs | Gene accession IDs | Sequence components of genes | Putative functions |
|---|---|---|---|---|---|---|
| CWSNP7041 | Ca-Kabuli-Chr8 | 11334210 | [C/T] | - | Intergenic | - |
| CWSNP7042 | Ca-Kabuli-Chr8 | 11349891 | [T/A] | - | DRR | - |
| CWSNP7043 | Ca-Kabuli-Chr8 | 11349995 | [A/G] | - | DRR | - |
| CWSNP7044 | Ca-Kabuli-Chr8 | 11403805 | [G/A] | Ca16820 | Synonymous-CDS | SANT domain, DNA binding |
| CWSNP7045 | Ca-Kabuli-Chr8 | 11403754 | [G/A] | Ca16820 | Non-Synonymous-CDS | SANT domain, DNA binding |
| CWSNP7046 | Ca-Kabuli-Chr8 | 11562814 | [A/C] | - | Intergenic | - |
| CWSNP7047 | Ca-Kabuli-Chr8 | 11631836 | [C/T] | Ca16809 | Non-Synonymous-CDS | Ketose-bisphosphatealdolase,class-II |
| CWSNP7048 | Ca-Kabuli-Chr8 | 11656700 | [G/T] | - | DRR | - |
| CWSNP7049 | Ca-Kabuli-Chr8 | 11656687 | [A/G] | - | DRR | - |
| CWSNP7050 | Ca-Kabuli-Chr8 | 11656675 | [T/C] | - | DRR | - |
| CWSNP7051 | Ca-Kabuli-Chr8 | 11656670 | [A/G] | - | DRR | - |
| CWSNP7052 | Ca-Kabuli-Chr8 | 11735882 | [A/C] | Ca16799 | Synonymous-CDS | Heat shock protein DnaJ,cysteine-richdomain |
| CWSNP7053 | Ca-Kabuli-Chr8 | 12875217 | [A/G] | - | Intergenic | - |
| CWSNP7054 | Ca-Kabuli-Chr8 | 13248268 | [T/C] | Ca20108 | Synonymous-CDS | C2 calcium-dependent membrane targeting |
| CWSNP7055 | Ca-Kabuli-Chr8 | 13745335 | [A/G] | - | Intergenic | - |
| CWSNP7056 | Ca-Kabuli-Chr8 | 13840865 | [A/C] | - | Intergenic | - |

| SNP IDs | Chromosomes | Physical positions (bp) | SNPs | Gene accession IDs | Sequence components of genes | Putative functions |
|---|---|---|---|---|---|---|
| CWSNP7057 | Ca-Kabuli-Chr8 | 13864026 | [T/G] | - | DRR | - |
| CWSNP7058 | Ca-Kabuli-Chr8 | 13998102 | [A/G] | - | Intergenic | - |
| CWSNP7059 | Ca-Kabuli-Chr8 | 14006709 | [G/A] | Ca22742 | Intron | - |
| CWSNP7060 | Ca-Kabuli-Chr8 | 14007431 | [A/G] | Ca22742 | Intron | - |
| CWSNP7061 | Ca-Kabuli-Chr8 | 14124588 | [C/A] | - | URR | - |
| CWSNP7062 | Ca-Kabuli-Chr8 | 14129040 | [G/A] | Ca13047 | Non-Synonymous-CDS | Protein phosphatase2C-like |
| CWSNP7063 | Ca-Kabuli-Chr8 | 14325980 | [C/A] | Ca13032 | Intron | Kinesin , motor domain |
| CWSNP7064 | Ca-Kabuli-Chr8 | 14444424 | [G/A] | - | URR | - |
| CWSNP7065 | Ca-Kabuli-Chr8 | 14444438 | [A/C] | - | URR | - |
| CWSNP7066 | Ca-Kabuli-Chr8 | 14445205 | [C/A] | - | URR | - |
| CWSNP7067 | Ca-Kabuli-Chr8 | 14528966 | [C/G] | - | DRR | - |
| CWSNP7068 | Ca-Kabuli-Chr8 | 14613302 | [G/A] | Ca13006 | Intron | Endonuclease/exonuclease/phosphatase |
| CWSNP7069 | Ca-Kabuli-Chr8 | 14640967 | [A/C] | Ca13004 | Intron | Toprimdomain |
| CWSNP7070 | Ca-Kabuli-Chr8 | 14715495 | [T/G] | Ca12997 | Intron | HEAT |
| CWSNP7071 | Ca-Kabuli-Chr8 | 14759784 | [G/T] | - | DRR | - |
| CWSNP7072 | Ca-Kabuli-Chr8 | 14760479 | [C/G] | - | DRR | - |

| SNP IDs | Chromosomes | Physical positions (bp) | SNPs | Gene accession IDs | Sequence components of genes | Putative functions |
|---|---|---|---|---|---|---|
| CWSNP7073 | Ca-Kabuli-Chr8 | 14760497 | [G/A] | - | DRR | - |
| CWSNP7074 | Ca-Kabuli-Chr8 | 14821416 | [G/A] | Ca12987 | Synonymous-CDS | Mrp,conserved site |
| CWSNP7075 | Ca-Kabuli-Chr8 | 15000109 | [A/G] | Ca12972 | Non-Synonymous-CDS | Aldehydedehydrogenase domain |
| CWSNP7076 | Ca-Kabuli-Chr8 | 15034465 | [T/C] | - | Intergenic | - |
| CWSNP7077 | Ca-Kabuli-Chr8 | 15131175 | [G/A] | Ca12965 | Synonymous-CDS | Sugar/inositol transporter |
| CWSNP7078 | Ca-Kabuli-Chr8 | 15555771 | [A/C] | Ca17888 | Synonymous-CDS | Protein of unknown function DUF1675 |
| CWSNP7079 | Ca-Kabuli-Chr8 | 15699180 | [T/C] | Ca17876 | Intron | RNA-dependent RNA polymerase,eukaryotic-type |
| CWSNP7080 | Ca-Kabuli-Chr8 | 15764874 | [G/A] | Ca17866 | Synonymous-CDS | Chromodomain |
| CWSNP7081 | Ca-Kabuli-Chr8 | 15857192 | [A/G] | - | Intergenic | - |
| CWSNP7082 | Ca-Kabuli-Chr8 | 15857359 | [T/G] | - | Intergenic | - |
| CWSNP7083 | Ca-Kabuli-Chr8 | 15873071 | [A/C] | Ca17857 | Synonymous-CDS | HEAT |
| CWSNP7084 | Ca-Kabuli-Chr8 | 15873133 | [A/G] | Ca17857 | Non-Synonymous-CDS | HEAT |
| CWSNP7085 | Ca-Kabuli-Chr8 | 16004976 | [G/C] | - | Intergenic | - |
| CWSNP7086 | Ca-Kabuli-Chr8 | 16005046 | [C/T] | - | Intergenic | - |
| CWSNP7087 | Ca-Kabuli-Chr8 | 16005078 | [A/G] | - | Intergenic | - |
| CWSNP7088 | Ca-Kabuli-Chr8 | 16020239 | [C/A] | - | DRR | - |

| SNP IDs | Chromosomes | Physical positions (bp) | SNPs | Gene accession IDs | Sequence components of genes | Putative functions |
|---|---|---|---|---|---|---|
| CWSNP7089 | Ca-Kabuli-Chr8 | 16020277 | [T/G] | - | DRR | - |
| CWSNP7090 | Ca-Kabuli-Chr8 | 16020323 | [G/T] | - | DRR | - |
| CWSNP7091 | Ca-Kabuli-Chr8 | 16022432 | [C/T] | - | Intergenic | - |
| CWSNP7092 | Ca-Kabuli-Chr8 | 16027974 | [A/T] | - | URR | - |
| CWSNP7093 | Ca-Kabuli-Chr8 | 16028064 | [G/T] | - | URR | - |
| CWSNP7094 | Ca-Kabuli-Chr8 | 16028062 | [G/A] | - | URR | - |
| CWSNP7095 | Ca-Kabuli-Chr8 | 16028040 | [A/G] | - | URR | - |
| CWSNP7096 | Ca-Kabuli-Chr8 | 16076161 | [C/G] | Ca15517 | Non-Synonymous-CDS | Gamma-glutamyltranspeptidase |
| CWSNP7097 | Ca-Kabuli-Chr8 | 16076168 | [A/G] | Ca15517 | Synonymous-CDS | Gamma-glutamyltranspeptidase |
| CWSNP7098 | Ca-Kabuli-Chr8 | 16102414 | [G/T] | - | Intergenic | - |
| CWSNP7099 | Ca-Kabuli-Chr8 | 16144279 | [T/A] | - | URR | - |
| CWSNP7100 | Ca-Kabuli-Chr8 | 16145275 | [A/G] | Ca15527 | Intron | Domain of unknown function DUF292,eukaryotic |
| CWSNP7101 | Ca-Kabuli-Chr8 | 16145411 | [C/G] | Ca15527 | Non-Synonymous-CDS | Domain of unknown function DUF292,eukaryotic |
| CWSNP7102 | Ca-Kabuli-Chr8 | 16145370 | [C/A] | Ca15527 | Intron | Domain of unknown function DUF292,eukaryotic |
| CWSNP7103 | Ca-Kabuli-Chr8 | 16264310 | [G/T] | - | DRR | - |
| CWSNP7104 | Ca-Kabuli-Chr8 | 16278758 | [T/C] | - | URR | - |

| SNP IDs | Chromosomes | Physical positions (bp) | SNPs | Gene accession IDs | Sequence components of genes | Putative functions |
|---|---|---|---|---|---|---|
| CWSNP7105 | Ca-Kabuli-Chr8 | 16283660 | [C/T] | - | URR | - |
| CWSNP7106 | Ca-Kabuli-Chr8 | 16283661 | [A/G] | - | URR | - |
| CWSNP7107 | Ca-Kabuli-Chr8 | 16283722 | [A/G] | - | URR | - |
| CWSNP7108 | Ca-Kabuli-Chr8 | 16283738 | [G/C] | - | URR | - |
| CWSNP7109 | Ca-Kabuli-Chr8 | 16411550 | [A/G] | Ca15562 | Intron | - |
| CWSNP7110 | Ca-Kabuli-Chr8 | 16421407 | [G/A] | - | DRR | - |
| CWSNP7111 | Ca-Kabuli-Chr8 | 16421441 | [C/A] | Ca15563 | Non-Synonymous-CDS | - |
| CWSNP7112 | Ca-Kabuli-Chr8 | 16421442 | [C/G] | Ca15563 | Synonymous-CDS | - |
| CWSNP7113 | Ca-Kabuli-Chr8 | 16421497 | [C/T] | Ca15563 | Non-Synonymous-CDS | - |
| CWSNP7114 | Ca-Kabuli-Chr8 | 16421595 | [A/C] | Ca15563 | Synonymous-CDS | - |
| CWSNP7115 | Ca-Kabuli-Chr8 | 16432907 | [T/G] | Ca15563 | Intron | - |
| CWSNP7116 | Ca-Kabuli-Chr8 | 16472919 | [T/A] | - | Intergenic | - |
| CWSNP7117 | Ca-Kabuli-Chr8 | 16472932 | [C/T] | - | Intergenic | - |
| CWSNP7118 | Ca-Kabuli-Chr8 | 16472933 | [G/C] | - | Intergenic | - |
| CWSNP7119 | Ca-Kabuli-Chr8 | 16473139 | [C/T] | - | Intergenic | - |
| CWSNP7120 | Ca-Kabuli-Chr8 | 16473157 | [C/T] | - | Intergenic | - |

| SNP IDs | Chromosomes | Physical positions (bp) | SNPs | Gene accession IDs | Sequence components of genes | Putative functions |
|---|---|---|---|---|---|---|
| CWSNP7121 | Ca-Kabuli-Chr8 | 16473290 | [A/G] | - | Intergenic | - |
| CWSNP7122 | scaffold1006 | 40651 | [T/A] | - | Intergenic | - |
| CWSNP7123 | scaffold1006 | 382579 | [C/T] | - | URR | - |
| CWSNP7124 | scaffold1006 | 436923 | [T/C] | - | Intergenic | - |
| CWSNP7125 | scaffold1006 | 644893 | [A/C] | Ca18050 | Non-Synonymous-CDS | CoA-binding |
| CWSNP7126 | scaffold1006 | 667620 | [A/G] | Ca18053 | Intron | - |
| CWSNP7127 | scaffold1006 | 667713 | [G/A] | Ca18053 | Intron | - |
| CWSNP7128 | scaffold1006 | 674495 | [A/G] | - | DRR | - |
| CWSNP7129 | scaffold1006 | 674823 | [G/A] | - | DRR | - |
| CWSNP7130 | scaffold1006 | 674826 | [C/T] | - | DRR | - |
| CWSNP7131 | scaffold1006 | 674844 | [T/C] | - | DRR | - |
| CWSNP7132 | scaffold1006 | 674847 | [T/G] | - | DRR | - |
| CWSNP7133 | scaffold1006 | 674862 | [G/A] | - | DRR | - |
| CWSNP7134 | scaffold1006 | 674865 | [T/C] | - | DRR | - |
| CWSNP7135 | scaffold1006 | 674871 | [A/G] | - | DRR | - |
| CWSNP7136 | scaffold1006 | 674881 | [G/A] | - | DRR | - |

| **SNP IDs** | **Chromosomes** | **Physical positions (bp)** | **SNPs** | **Gene accession IDs** | **Sequence components of genes** | **Putative functions** |
|---|---|---|---|---|---|---|
| CWSNP7137 | scaffold1010 | 90092 | [G/A] | - | DRR | - |
| CWSNP7138 | scaffold1013 | 56140 | [G/A] | - | Intergenic | - |
| CWSNP7139 | scaffold1013 | 56116 | [C/T] | - | Intergenic | - |
| CWSNP7140 | scaffold1013 | 56114 | [C/A] | - | Intergenic | - |
| CWSNP7141 | scaffold1013 | 56075 | [T/A] | - | Intergenic | - |
| CWSNP7142 | scaffold1013 | 56074 | [C/T] | - | Intergenic | - |
| CWSNP7143 | scaffold1013 | 56069 | [C/T] | - | Intergenic | - |
| CWSNP7144 | scaffold10186 | 410 | [A/C] | - | Intergenic | - |
| CWSNP7145 | scaffold10186 | 378 | [A/T] | - | Intergenic | - |
| CWSNP7146 | scaffold10186 | 397 | [G/A] | - | Intergenic | - |
| CWSNP7147 | scaffold10186 | 573 | [T/G] | - | Intergenic | - |
| CWSNP7148 | scaffold1027 | 256612 | [A/C] | - | Intergenic | - |
| CWSNP7149 | scaffold1034 | 73684 | [G/A] | Ca27683 | Non-Synonymous-CDS | - |
| CWSNP7150 | scaffold10420 | 40193 | [G/T] | - | URR | - |
| CWSNP7151 | scaffold1043 | 16328 | [A/T] | - | Intergenic | - |
| CWSNP7152 | scaffold1043 | 16333 | [A/T] | - | Intergenic | - |

| SNP IDs | Chromosomes | Physical positions (bp) | SNPs | Gene accession IDs | Sequence components of genes | Putative functions |
|---|---|---|---|---|---|---|
| CWSNP7153 | scaffold1047 | 235352 | [T/C] | Ca17194 | Intron | Engulfment/cellmotility,ELMO |
| CWSNP7154 | scaffold1047 | 577874 | [T/G] | - | Intergenic | - |
| CWSNP7155 | scaffold1050 | 3415 | [C/T] | Ca25913 | Non-Synonymous-CDS | - |
| CWSNP7156 | scaffold1050 | 3463 | [C/T] | Ca25913 | Non-Synonymous-CDS | PeptidaseC48,SUMO/Sentrin/Ubl1 |
| CWSNP7157 | scaffold1050 | 126986 | [T/G] | Ca25916 | Intron | Synaptojanin,N-terminal |
| CWSNP7158 | scaffold1050 | 127022 | [G/A] | Ca25916 | Intron | Synaptojanin,N-terminal |
| CWSNP7159 | scaffold1050 | 127023 | [G/T] | Ca25916 | Intron | Synaptojanin,N-terminal |
| CWSNP7160 | scaffold1050 | 127054 | [C/T] | Ca25916 | Intron | Synaptojanin,N-terminal |
| CWSNP7161 | scaffold1055 | 344459 | [G/A] | - | Intergenic | - |
| CWSNP7162 | scaffold1060 | 394867 | [C/T] | Ca21793 | Intron | Transposase,Ptta/En/Spm,plant |
| CWSNP7163 | scaffold1060 | 395029 | [C/T] | Ca21793 | Intron | Transposase,Ptta/En/Spm,plant |
| CWSNP7164 | scaffold1061 | 63613 | [A/G] | - | Intergenic | - |
| CWSNP7165 | scaffold1061 | 63620 | [C/T] | - | Intergenic | - |
| CWSNP7166 | scaffold1065 | 142678 | [A/C] | - | Intergenic | - |
| CWSNP7167 | scaffold1089 | 1154 | [C/A] | - | Intergenic | - |
| CWSNP7168 | scaffold1089 | 1240 | [T/G] | Ca24272 | Non-Synonymous-CDS | - |

| SNP IDs | Chromosomes | Physical positions (bp) | SNPs | Gene accession IDs | Sequence components of genes | Putative functions |
|---|---|---|---|---|---|---|
| CWSNP7169 | scaffold1089 | 1281 | [G/A] | Ca24272 | Non-Synonymous-CDS | - |
| CWSNP7170 | scaffold1089 | 3160 | [C/A] | - | Intergenic | - |
| CWSNP7171 | scaffold1089 | 3236 | [T/G] | - | Intergenic | - |
| CWSNP7172 | scaffold1089 | 3249 | [T/G] | - | Intergenic | - |
| CWSNP7173 | scaffold1089 | 3248 | [C/T] | - | Intergenic | - |
| CWSNP7174 | scaffold1089 | 3240 | [G/C] | - | Intergenic | - |
| CWSNP7175 | scaffold1089 | 3228 | [C/T] | - | Intergenic | - |
| CWSNP7176 | scaffold1089 | 3191 | [G/A] | - | Intergenic | - |
| CWSNP7177 | scaffold109_1 | 156485 | [C/T] | Ca11572 | Intron | MembraneboundO-acyltransferase,MBOAT |
| CWSNP7178 | scaffold109_1 | 328757 | [C/T] | Ca11580 | Non-Synonymous-CDS | - |
| CWSNP7179 | scaffold109_1 | 385653 | [C/T] | - | DRR | - |
| CWSNP7180 | scaffold109_1 | 696869 | [A/C] | Ca11606 | Intron | TetratricopeptideTPR-1 |
| CWSNP7181 | scaffold109_1 | 741607 | [G/A] | Ca11609 | Synonymous-CDS | uDENN |
| CWSNP7182 | scaffold1109 | 150672 | [G/A] | Ca25534 | Non-Synonymous-CDS | - |
| CWSNP7183 | scaffold1109 | 150720 | [G/A] | Ca25534 | Non-Synonymous-CDS | - |
| CWSNP7184 | scaffold1109 | 150676 | [C/T] | Ca25534 | Non-Synonymous-CDS | - |

| SNP IDs | Chromosomes | Physical positions (bp) | SNPs | Gene accession IDs | Sequence components of genes | Putative functions |
|---|---|---|---|---|---|---|
| CWSNP7185 | scaffold1115 | 153349 | [T/C] | - | DRR | - |
| CWSNP7186 | scaffold1118 | 2215 | [T/C] | - | Intergenic | - |
| CWSNP7187 | scaffold1118 | 2224 | [T/A] | - | Intergenic | - |
| CWSNP7188 | scaffold1118 | 2225 | [C/T] | - | Intergenic | - |
| CWSNP7189 | scaffold1118 | 2226 | [T/G] | - | Intergenic | - |
| CWSNP7190 | scaffold1118 | 2315 | [T/C] | - | Intergenic | - |
| CWSNP7191 | scaffold1118 | 9568 | [A/G] | Ca27404 | Intron | Inositol polyphosphate-related phosphatase |
| CWSNP7192 | scaffold1118 | 9556 | [C/T] | Ca27404 | Intron | - |
| CWSNP7193 | scaffold1128 | 94196 | [T/A] | Ca23728 | Intron | ABC transporter-like |
| CWSNP7194 | scaffold1128 | 112702 | [G/A] | - | Intergenic | - |
| CWSNP7195 | scaffold1128 | 341416 | [T/A] | - | Intergenic | - |
| CWSNP7196 | scaffold1128 | 360567 | [T/A] | - | Intergenic | - |
| CWSNP7197 | scaffold1128 | 360529 | [A/C] | - | Intergenic | - |
| CWSNP7198 | scaffold1128 | 423553 | [A/G] | - | Intergenic | - |
| CWSNP7199 | scaffold1132 | 26497 | [C/T] | - | Intergenic | - |
| CWSNP7200 | scaffold1132 | 26492 | [G/A] | - | Intergenic | - |

| SNP IDs | Chromosomes | Physical positions (bp) | SNPs | Gene accession IDs | Sequence components of genes | Putative functions |
|---|---|---|---|---|---|---|
| CWSNP7201 | scaffold1151 | 228312 | [C/G] | - | Intergenic | - |
| CWSNP7202 | scaffold1151 | 228304 | [A/T] | - | Intergenic | - |
| CWSNP7203 | scaffold1151 | 228291 | [C/T] | - | Intergenic | - |
| CWSNP7204 | scaffold1176_2 | 52146 | [T/C] | - | Intergenic | - |
| CWSNP7205 | scaffold1176_2 | 52142 | [G/T] | - | Intergenic | - |
| CWSNP7206 | scaffold1176_2 | 52125 | [C/T] | - | Intergenic | - |
| CWSNP7207 | scaffold1176_2 | 52124 | [C/T] | - | Intergenic | - |
| CWSNP7208 | scaffold1176_2 | 330840 | [C/A] | - | Intergenic | - |
| CWSNP7209 | scaffold118 | 206562 | [A/G] | Ca22950 | Non-Synonymous-CDS | - |
| CWSNP7210 | scaffold118 | 303784 | [A/C] | Ca22952 | Intron | Sulfatase |
| CWSNP7211 | scaffold118 | 332673 | [T/C] | - | URR | - |
| CWSNP7212 | scaffold1180 | 114508 | [C/A] | Ca25739 | Synonymous-CDS | Staphylococcal nuclease (SNase-like) |
| CWSNP7213 | scaffold1185 | 944 | [G/A] | - | Intergenic | - |
| CWSNP7214 | scaffold1185 | 1110 | [G/A] | - | Intergenic | - |
| CWSNP7215 | scaffold1185 | 1134 | [T/A] | - | Intergenic | - |
| CWSNP7216 | scaffold1185 | 1098 | [T/C] | - | Intergenic | - |

| SNP IDs | Chromosomes | Physical positions (bp) | SNPs | Gene accession IDs | Sequence components of genes | Putative functions |
|---|---|---|---|---|---|---|
| CWSNP7217 | scaffold119 | 12259 | [A/G] | - | Intergenic | - |
| CWSNP7218 | scaffold119 | 12263 | [C/A] | - | Intergenic | - |
| CWSNP7219 | scaffold119 | 12279 | [G/A] | - | Intergenic | - |
| CWSNP7220 | scaffold1196 | 70506 | [G/A] | - | Intergenic | - |
| CWSNP7221 | scaffold1196 | 70552 | [A/C] | - | Intergenic | - |
| CWSNP7222 | scaffold1196 | 70549 | [G/A] | - | Intergenic | - |
| CWSNP7223 | scaffold1196 | 70547 | [G/C] | - | Intergenic | - |
| CWSNP7224 | scaffold1196 | 70535 | [G/A] | - | Intergenic | - |
| CWSNP7225 | scaffold1196 | 70526 | [T/C] | - | Intergenic | - |
| CWSNP7226 | scaffold1197 | 344239 | [A/C] | - | Intergenic | - |
| CWSNP7227 | scaffold1197 | 407641 | [G/A] | Ca24073 | Synonymous-CDS | TGS |
| CWSNP7228 | scaffold1197 | 407658 | [C/G] | Ca24073 | Non-Synonymous-CDS | TGS |
| CWSNP7229 | scaffold1197 | 407691 | [T/C] | Ca24073 | Non-Synonymous-CDS | TGS |
| CWSNP7230 | scaffold1197 | 407695 | [T/C] | Ca24073 | Synonymous-CDS | TGS |
| CWSNP7231 | scaffold1197 | 447890 | [C/T] | - | DRR | - |
| CWSNP7232 | scaffold1197 | 520164 | [T/A] | - | Intergenic | - |

| SNP IDs | Chromosomes | Physical positions (bp) | SNPs | Gene accession IDs | Sequence components of genes | Putative functions |
|---|---|---|---|---|---|---|
| CWSNP7233 | scaffold1197 | 520153 | [C/G] | - | Intergenic | - |
| CWSNP7234 | scaffold1197 | 520148 | [A/T] | - | Intergenic | - |
| CWSNP7235 | scaffold12 | 200573 | [G/C] | - | Intergenic | - |
| CWSNP7236 | scaffold12 | 331890 | [A/G] | - | URR | - |
| CWSNP7237 | scaffold12 | 332909 | [C/T] | - | URR | - |
| CWSNP7238 | scaffold12 | 332921 | [G/A] | - | URR | - |
| CWSNP7239 | scaffold12 | 332963 | [C/T] | - | URR | - |
| CWSNP7240 | scaffold1202 | 46904 | [C/T] | Ca24659 | Intron | CDP-alcoholphosphatidyltransferase |
| CWSNP7241 | scaffold1215 | 40741 | [G/T] | Ca28164 | Synonymous-CDS | PeptidaseS8/S53,subtilisin/kexin/sedolisin |
| CWSNP7242 | scaffold1215 | 40793 | [A/G] | Ca28164 | Synonymous-CDS | PeptidaseS8/S53,subtilisin/kexin/sedolisin |
| CWSNP7243 | scaffold1215 | 40797 | [A/T] | Ca28164 | Non-Synonymous-CDS | PeptidaseS8/S53,subtilisin/kexin/sedolisin |
| CWSNP7244 | scaffold1215 | 40817 | [G/A] | Ca28164 | Non-Synonymous-CDS | PeptidaseS8/S53,subtilisin/kexin/sedolisin |
| CWSNP7245 | scaffold1215 | 40814 | [A/T] | Ca28164 | Non-Synonymous-CDS | PeptidaseS8/S53,subtilisin/kexin/sedolisin |
| CWSNP7246 | scaffold1219 | 34791 | [G/A] | - | Intergenic | - |
| CWSNP7247 | scaffold1219 | 85015 | [A/C] | - | Intergenic | - |
| CWSNP7248 | scaffold1259 | 71428 | [C/T] | - | Intergenic | - |

| SNP IDs | Chromosomes | Physical positions (bp) | SNPs | Gene accession IDs | Sequence components of genes | Putative functions |
|---|---|---|---|---|---|---|
| CWSNP7249 | scaffold1259 | 71649 | [C/T] | - | Intergenic | - |
| CWSNP7250 | scaffold127 | 28809 | [A/T] | - | Intergenic | - |
| CWSNP7251 | scaffold127 | 28776 | [T/G] | - | Intergenic | - |
| CWSNP7252 | scaffold127 | 28772 | [G/A] | - | Intergenic | - |
| CWSNP7253 | scaffold1272 | 57303 | [C/A] | Ca26853 | Intron | Glycosyltransferase,family20 |
| CWSNP7254 | scaffold128 | 52855 | [G/C] | - | Intergenic | - |
| CWSNP7255 | scaffold128 | 52844 | [A/T] | - | Intergenic | - |
| CWSNP7256 | scaffold128 | 263586 | [T/A] | - | Intergenic | - |
| CWSNP7257 | scaffold128 | 263572 | [T/A] | - | Intergenic | - |
| CWSNP7258 | scaffold128 | 334180 | [G/T] | - | Intergenic | - |
| CWSNP7259 | scaffold128 | 334129 | [G/A] | - | Intergenic | - |
| CWSNP7260 | scaffold128 | 593782 | [T/C] | - | Intergenic | - |
| CWSNP7261 | scaffold128 | 860975 | [G/A] | Ca21452 | Intron | Helicase,C-terminal |
| CWSNP7262 | scaffold128 | 860981 | [T/A] | Ca21452 | Intron | Helicase,C-terminal |
| CWSNP7263 | scaffold128 | 860983 | [A/T] | Ca21452 | Intron | Helicase,C-terminal |
| CWSNP7264 | scaffold128 | 860987 | [C/T] | Ca21452 | Intron | Helicase,C-terminal |

| SNP IDs | Chromosomes | Physical positions (bp) | SNPs | Gene accession IDs | Sequence components of genes | Putative functions |
|---|---|---|---|---|---|---|
| CWSNP7265 | scaffold128 | 861003 | [C/A] | Ca21452 | Intron | Helicase,C-terminal |
| CWSNP7266 | scaffold128 | 861010 | [C/T] | Ca21452 | Intron | Helicase,C-terminal |
| CWSNP7267 | scaffold128 | 861022 | [T/C] | Ca21452 | Intron | Helicase,C-terminal |
| CWSNP7268 | scaffold128 | 861028 | [T/C] | Ca21452 | Intron | Helicase,C-terminal |
| CWSNP7269 | scaffold128 | 861053 | [G/A] | Ca21452 | Intron | Helicase,C-terminal |
| CWSNP7270 | scaffold128 | 861015 | [C/T] | Ca21452 | Intron | Helicase,C-terminal |
| CWSNP7271 | scaffold1281 | 244153 | [A/C] | Ca21517 | Synonymous-CDS | Protein kinase, catalytic domain |
| CWSNP7272 | scaffold1281 | 316037 | [A/G] | - | URR | - |
| CWSNP7273 | scaffold1281 | 398729 | [T/G] | Ca21525 | Non-Synonymous-CDS | Armadillo |
| CWSNP7274 | scaffold1281 | 526446 | [C/T] | - | Intergenic | - |
| CWSNP7275 | scaffold1281 | 526442 | [T/C] | - | Intergenic | - |
| CWSNP7276 | scaffold1281 | 527012 | [C/T] | - | Intergenic | - |
| CWSNP7277 | scaffold1281 | 527008 | [T/C] | - | Intergenic | - |
| CWSNP7278 | scaffold1285 | 68330 | [G/A] | Ca24805 | Intron | PeptidaseC48,SUMO/Sentrin/Ubl1 |
| CWSNP7279 | scaffold1285 | 68327 | [G/T] | Ca24805 | Intron | PeptidaseC48,SUMO/Sentrin/Ubl1 |
| CWSNP7280 | scaffold1285 | 68324 | [T/C] | Ca24805 | Intron | PeptidaseC48,SUMO/Sentrin/Ubl1 |

| SNP IDs | Chromosomes | Physical positions (bp) | SNPs | Gene accession IDs | Sequence components of genes | Putative functions |
|---|---|---|---|---|---|---|
| CWSNP7281 | scaffold1285 | 68311 | [G/A] | Ca24805 | Synonymous-CDS | PeptidaseC48,SUMO/Sentrin/Ubl1 |
| CWSNP7282 | scaffold1285 | 96310 | [A/C] | - | Intergenic | - |
| CWSNP7283 | scaffold1285 | 96358 | [T/C] | - | Intergenic | - |
| CWSNP7284 | scaffold1285 | 96429 | [C/T] | - | Intergenic | - |
| CWSNP7285 | scaffold1285 | 96428 | [G/A] | - | Intergenic | - |
| CWSNP7286 | scaffold1285 | 96414 | [C/T] | - | Intergenic | - |
| CWSNP7287 | scaffold1285 | 96411 | [G/A] | - | Intergenic | - |
| CWSNP7288 | scaffold1285 | 96409 | [C/T] | - | Intergenic | - |
| CWSNP7289 | scaffold1285 | 96399 | [C/T] | - | Intergenic | - |
| CWSNP7290 | scaffold1285 | 105553 | [T/C] | - | Intergenic | - |
| CWSNP7291 | scaffold1285 | 105507 | [A/T] | - | Intergenic | - |
| CWSNP7292 | scaffold1285 | 105504 | [G/A] | - | Intergenic | - |
| CWSNP7293 | scaffold1285 | 192068 | [T/G] | - | Intergenic | - |
| CWSNP7294 | scaffold1285 | 240762 | [C/T] | Ca24811 | Intron | - |
| CWSNP7295 | scaffold1285 | 240735 | [G/A] | Ca24811 | Intron | - |
| CWSNP7296 | scaffold1285 | 240730 | [T/C] | Ca24811 | Intron | - |

| SNP IDs | Chromosomes | Physical positions (bp) | SNPs | Gene accession IDs | Sequence components of genes | Putative functions |
|---|---|---|---|---|---|---|
| CWSNP7297 | scaffold1285 | 240723 | [C/T] | Ca24811 | Intron | - |
| CWSNP7298 | scaffold1301_1 | 45565 | [A/C] | Ca11165 | Synonymous-CDS | - |
| CWSNP7299 | scaffold1301_1 | 45566 | [G/A] | Ca11165 | Synonymous-CDS | - |
| CWSNP7300 | scaffold1301_1 | 58243 | [A/G] | - | Intergenic | - |
| CWSNP7301 | scaffold1301_1 | 154342 | [A/C] | - | Intergenic | - |
| CWSNP7302 | scaffold1308 | 44260 | [A/G] | - | Intergenic | - |
| CWSNP7303 | scaffold1309 | 49259 | [G/A] | Ca26762 | Synonymous-CDS | Transposase,Ptta/En/Spm,plant |
| CWSNP7304 | scaffold1312 | 98400 | [T/C] | - | Intergenic | - |
| CWSNP7305 | scaffold1313 | 18173 | [A/G] | Ca25873 | Intron | Homeobox |
| CWSNP7306 | scaffold1315 | 115471 | [G/A] | - | Intergenic | - |
| CWSNP7307 | scaffold1315 | 115446 | [C/A] | - | Intergenic | - |
| CWSNP7308 | scaffold132 | 153364 | [A/G] | - | Intergenic | - |
| CWSNP7309 | scaffold132 | 153361 | [A/G] | - | Intergenic | - |
| CWSNP7310 | scaffold1324 | 228151 | [G/A] | Ca19765 | Synonymous-CDS | - |
| CWSNP7311 | scaffold1324 | 228159 | [A/C] | Ca19765 | Non-Synonymous-CDS | - |
| CWSNP7312 | scaffold1324 | 262208 | [C/G] | Ca19766 | Non-Synonymous-CDS | - |

| SNP IDs | Chromosomes | Physical positions (bp) | SNPs | Gene accession IDs | Sequence components of genes | Putative functions |
|---|---|---|---|---|---|---|
| CWSNP7313 | scaffold134 | 218352 | [T/G] | Ca22581 | Non-Synonymous-CDS | SET domain |
| CWSNP7314 | scaffold134 | 218336 | [T/G] | Ca22581 | Non-Synonymous-CDS | SET domain |
| CWSNP7315 | scaffold134 | 223318 | [C/T] | Ca22581 | Intron | SET domain |
| CWSNP7316 | scaffold1348_1 | 134572 | [G/A] | - | Intergenic | - |
| CWSNP7317 | scaffold1348_1 | 380225 | [T/G] | Ca08725 | Synonymous-CDS | PeptidaseS8/S53,subtilisin/kexin/sedolisin |
| CWSNP7318 | scaffold1348_1 | 452011 | [G/T] | Ca08730 | Non-Synonymous-CDS | Glucose-methanol-cholineoxidoreductase,N-terminal |
| CWSNP7319 | scaffold1348_1 | 454342 | [G/T] | - | URR | - |
| CWSNP7320 | scaffold1348_1 | 535984 | [G/T] | Ca08741 | Non-Synonymous-CDS | IQ motif, EF-hand binding site |
| CWSNP7321 | scaffold1348_1 | 553863 | [G/T] | - | DRR | - |
| CWSNP7322 | scaffold1348_1 | 554134 | [A/G] | - | DRR | - |
| CWSNP7323 | scaffold1348_1 | 652067 | [A/G] | Ca08753 | Non-Synonymous-CDS | PeptidaseS8/S53,subtilisin/kexin/sedolisin |
| CWSNP7324 | scaffold1348_1 | 667541 | [A/C] | Ca08754 | Synonymous-CDS | PeptidaseS8/S53,subtilisin/kexin/sedolisin |
| CWSNP7325 | scaffold1348_1 | 732450 | [A/C] | Ca08761 | Synonymous-CDS | Forkhead-associated (FHA) domain |
| CWSNP7326 | scaffold1348_1 | 732454 | [T/G] | Ca08761 | Intron | Forkhead-associated (FHA) domain |
| CWSNP7327 | scaffold1348_1 | 732460 | [T/A] | Ca08761 | Intron | Forkhead-associated (FHA) domain |
| CWSNP7328 | scaffold1348_1 | 732496 | [G/T] | Ca08761 | Intron | Forkhead-associated (FHA) domain |

| SNP IDs | Chromosomes | Physical positions (bp) | SNPs | Gene accession IDs | Sequence components of genes | Putative functions |
|---|---|---|---|---|---|---|
| CWSNP7329 | scaffold1348_1 | 750213 | [C/A] | Ca08762 | Intron | COGcomplexcomponent,COG2 |
| CWSNP7330 | scaffold1348_1 | 909909 | [A/G] | - | DRR | - |
| CWSNP7331 | scaffold1348_1 | 959608 | [A/C] | Ca08787 | Non-Synonymous-CDS | Transcription factor jumonji/aspartyl beta-hydroxylase |
| CWSNP7332 | scaffold1348_1 | 959828 | [G/A] | Ca08787 | Synonymous-CDS | Transcription factor jumonji/aspartyl beta-hydroxylase |
| CWSNP7333 | scaffold1348_1 | 1066818 | [A/C] | Ca08793 | Intron | Peptidase,cysteinepeptidaseactivesite |
| CWSNP7334 | scaffold1348_1 | 1072750 | [C/T] | Ca08794 | Synonymous-CDS | WD40 repeat |
| CWSNP7335 | scaffold1348_1 | 1265627 | [T/C] | Ca08816 | Synonymous-CDS | Protein kinase, catalytic domain |
| CWSNP7336 | scaffold1348_1 | 1265635 | [T/G] | Ca08816 | Non-Synonymous-CDS | Protein kinase, catalytic domain |
| CWSNP7337 | scaffold1348_1 | 1273341 | [G/A] | - | Intergenic | - |
| CWSNP7338 | scaffold1348_1 | 1288448 | [A/G] | Ca08820 | Intron | RNA helicase, ATP-dependent, DEAD-box,conserved site |
| CWSNP7339 | scaffold1348_1 | 1296453 | [G/A] | - | DRR | - |
| CWSNP7340 | scaffold1348_1 | 1330288 | [A/G] | - | DRR | - |
| CWSNP7341 | scaffold1348_1 | 1330400 | [A/G] | - | DRR | - |
| CWSNP7342 | scaffold1348_1 | 1403480 | [A/G] | - | DRR | - |
| CWSNP7343 | scaffold1351 | 177159 | [G/A] | - | Intergenic | - |
| CWSNP7344 | scaffold1351 | 177172 | [G/A] | - | Intergenic | - |

| SNP IDs | Chromosomes | Physical positions (bp) | SNPs | Gene accession IDs | Sequence components of genes | Putative functions |
|---|---|---|---|---|---|---|
| CWSNP7345 | scaffold1351 | 177178 | [T/C] | - | Intergenic | - |
| CWSNP7346 | scaffold1351 | 177171 | [T/G] | - | Intergenic | - |
| CWSNP7347 | scaffold1351 | 177193 | [T/C] | - | Intergenic | - |
| CWSNP7348 | scaffold1351 | 177256 | [T/G] | - | Intergenic | - |
| CWSNP7349 | scaffold1351 | 177248 | [G/T] | - | Intergenic | - |
| CWSNP7350 | scaffold1351 | 177247 | [G/A] | - | Intergenic | - |
| CWSNP7351 | scaffold1351 | 177242 | [G/A] | - | Intergenic | - |
| CWSNP7352 | scaffold1351 | 177241 | [T/G] | - | Intergenic | - |
| CWSNP7353 | scaffold1351 | 177240 | [C/A] | - | Intergenic | - |
| CWSNP7354 | scaffold1351 | 177235 | [G/T] | - | Intergenic | - |
| CWSNP7355 | scaffold1351 | 177234 | [A/C] | - | Intergenic | - |
| CWSNP7356 | scaffold1351 | 177210 | [T/A] | - | Intergenic | - |
| CWSNP7357 | scaffold1351 | 177200 | [T/G] | - | Intergenic | - |
| CWSNP7358 | scaffold1351 | 177191 | [C/T] | - | Intergenic | - |
| CWSNP7359 | scaffold1351 | 177188 | [G/T] | - | Intergenic | - |
| CWSNP7360 | scaffold1351 | 204656 | [C/A] | - | Intergenic | - |

| SNP IDs | Chromosomes | Physical positions (bp) | SNPs | Gene accession IDs | Sequence components of genes | Putative functions |
|---|---|---|---|---|---|---|
| CWSNP7361 | scaffold1351 | 335227 | [T/C] | Ca22005 | Synonymous-CDS | - |
| CWSNP7362 | scaffold1351 | 335239 | [G/T] | Ca22005 | Non-Synonymous-CDS | - |
| CWSNP7363 | scaffold1351 | 367576 | [A/G] | - | Intergenic | - |
| CWSNP7364 | scaffold1351 | 367578 | [G/A] | - | Intergenic | - |
| CWSNP7365 | scaffold1351 | 367745 | [G/A] | - | Intergenic | - |
| CWSNP7366 | scaffold1369 | 8115 | [T/G] | - | Intergenic | - |
| CWSNP7367 | scaffold1369 | 8154 | [T/G] | - | Intergenic | - |
| CWSNP7368 | scaffold1369 | 8221 | [T/G] | - | Intergenic | - |
| CWSNP7369 | scaffold1369 | 31000 | [A/G] | - | Intergenic | - |
| CWSNP7370 | scaffold1390 | 98354 | [A/G] | - | Intergenic | - |
| CWSNP7371 | scaffold1417 | 4906 | [G/A] | Ca25454 | Non-Synonymous-CDS | - |
| CWSNP7372 | scaffold1417 | 4917 | [G/T] | Ca25454 | Non-Synonymous-CDS | - |
| CWSNP7373 | scaffold1417 | 76627 | [A/C] | - | Intergenic | - |
| CWSNP7374 | scaffold1417 | 230210 | [T/G] | - | Intergenic | - |
| CWSNP7375 | scaffold1419 | 24845 | [C/T] | - | DRR | - |
| CWSNP7376 | scaffold143 | 18492 | [A/G] | - | Intergenic | - |

| SNP IDs | Chromosomes | Physical positions (bp) | SNPs | Gene accession IDs | Sequence components of genes | Putative functions |
|---|---|---|---|---|---|---|
| CWSNP7377 | scaffold1439 | 242972 | [A/G] | Ca23658 | Synonymous-CDS | - |
| CWSNP7378 | scaffold1448 | 47306 | [A/G] | Ca26687 | Intron | Importin-beta,N-terminal |
| CWSNP7379 | scaffold1448 | 74436 | [C/T] | Ca26687 | Intron | Importin-beta,N-terminal |
| CWSNP7380 | scaffold1448 | 74434 | [C/T] | Ca26687 | Intron | Importin-beta,N-terminal |
| CWSNP7381 | scaffold1448 | 74529 | [C/T] | Ca26687 | Intron | Importin-beta,N-terminal |
| CWSNP7382 | scaffold1448 | 76809 | [G/A] | Ca26687 | Intron | Importin-beta,N-terminal |
| CWSNP7383 | scaffold1449 | 55474 | [T/G] | Ca28129 | Synonymous-CDS | - |
| CWSNP7384 | scaffold1449 | 55532 | [G/A] | Ca28129 | Non-Synonymous-CDS | - |
| CWSNP7385 | scaffold1452 | 3501 | [C/A] | - | Intergenic | - |
| CWSNP7386 | scaffold1452 | 3496 | [A/T] | - | Intergenic | - |
| CWSNP7387 | scaffold1452 | 3495 | [C/A] | - | Intergenic | - |
| CWSNP7388 | scaffold1452 | 3737 | [C/A] | - | Intergenic | - |
| CWSNP7389 | scaffold1462 | 1556 | [T/G] | Ca28230 | Intron | Protein kinase, catalytic domain |
| CWSNP7390 | scaffold1466 | 90846 | [C/A] | - | Intergenic | - |
| CWSNP7391 | scaffold1466 | 322287 | [G/A] | - | Intergenic | - |
| CWSNP7392 | scaffold1466 | 322315 | [G/T] | - | Intergenic | - |

| SNP IDs | Chromosomes | Physical positions (bp) | SNPs | Gene accession IDs | Sequence components of genes | Putative functions |
|---|---|---|---|---|---|---|
| CWSNP7393 | scaffold1466 | 322450 | [G/T] | - | Intergenic | - |
| CWSNP7394 | scaffold1466 | 322448 | [T/G] | - | Intergenic | - |
| CWSNP7395 | scaffold1466 | 322415 | [G/A] | - | Intergenic | - |
| CWSNP7396 | scaffold1466 | 322414 | [G/A] | - | Intergenic | - |
| CWSNP7397 | scaffold1466 | 322400 | [C/T] | - | Intergenic | - |
| CWSNP7398 | scaffold1466 | 322390 | [G/A] | - | Intergenic | - |
| CWSNP7399 | scaffold1466 | 322383 | [C/T] | - | Intergenic | - |
| CWSNP7400 | scaffold1467 | 205803 | [C/T] | - | DRR | - |
| CWSNP7401 | scaffold1467 | 205833 | [G/A] | - | DRR | - |
| CWSNP7402 | scaffold1467 | 205845 | [A/G] | - | DRR | - |
| CWSNP7403 | scaffold1467 | 206121 | [G/A] | - | DRR | - |
| CWSNP7404 | scaffold1467 | 206118 | [C/T] | - | DRR | - |
| CWSNP7405 | scaffold1467 | 206100 | [G/A] | - | DRR | - |
| CWSNP7406 | scaffold1467 | 206094 | [T/C] | - | DRR | - |
| CWSNP7407 | scaffold1467 | 206085 | [T/C] | - | DRR | - |
| CWSNP7408 | scaffold1467 | 206067 | [T/G] | - | DRR | - |

| SNP IDs | Chromosomes | Physical positions (bp) | SNPs | Gene accession IDs | Sequence components of genes | Putative functions |
|---|---|---|---|---|---|---|
| CWSNP7409 | scaffold1475 | 4820 | [G/A] | - | Intergenic | - |
| CWSNP7410 | scaffold1475 | 4796 | [C/T] | - | Intergenic | - |
| CWSNP7411 | scaffold1475 | 4794 | [C/A] | - | Intergenic | - |
| CWSNP7412 | scaffold1475 | 4755 | [T/A] | - | Intergenic | - |
| CWSNP7413 | scaffold1475 | 4754 | [C/T] | - | Intergenic | - |
| CWSNP7414 | scaffold1475 | 4749 | [C/T] | - | Intergenic | - |
| CWSNP7415 | scaffold1475 | 4845 | [A/T] | - | Intergenic | - |
| CWSNP7416 | scaffold1483 | 56686 | [A/T] | - | DRR | - |
| CWSNP7417 | scaffold1493 | 81740 | [C/T] | Ca26514 | Intron | PeptidaseC48,SUMO/Sentrin/Ubl1 |
| CWSNP7418 | scaffold1493 | 81784 | [C/A] | Ca26514 | Intron | PeptidaseC48,SUMO/Sentrin/Ubl1 |
| CWSNP7419 | scaffold1493 | 81789 | [G/A] | Ca26514 | Intron | PeptidaseC48,SUMO/Sentrin/Ubl1 |
| CWSNP7420 | scaffold1496 | 289997 | [G/T] | - | Intergenic | - |
| CWSNP7421 | scaffold1496 | 290018 | [C/A] | - | Intergenic | - |
| CWSNP7422 | scaffold1498 | 3474 | [A/C] | Ca28063 | Synonymous-CDS | Multiantimicrobial extrusion protein |
| CWSNP7423 | scaffold1498 | 3641 | [T/C] | Ca28063 | Intron | Multiantimicrobial extrusion protein |
| CWSNP7424 | scaffold1504 | 160623 | [G/T] | Ca24092 | Synonymous-CDS | - |

| SNP IDs | Chromosomes | Physical positions (bp) | SNPs | Gene accession IDs | Sequence components of genes | Putative functions |
|---|---|---|---|---|---|---|
| CWSNP7425 | scaffold1504 | 189700 | [A/G] | - | Intergenic | - |
| CWSNP7426 | scaffold1504 | 189699 | [C/T] | - | Intergenic | - |
| CWSNP7427 | scaffold1504 | 189681 | [C/T] | - | Intergenic | - |
| CWSNP7428 | scaffold1504 | 189664 | [G/A] | - | Intergenic | - |
| CWSNP7429 | scaffold1504 | 189663 | [G/A] | - | Intergenic | - |
| CWSNP7430 | scaffold1504 | 197789 | [G/A] | - | Intergenic | - |
| CWSNP7431 | scaffold1504 | 197790 | [G/A] | - | Intergenic | - |
| CWSNP7432 | scaffold1504 | 197819 | [T/G] | - | Intergenic | - |
| CWSNP7433 | scaffold1505 | 33297 | [A/C] | Ca28120 | Synonymous-CDS | - |
| CWSNP7434 | scaffold151 | 267060 | [T/G] | Ca25631 | Synonymous-CDS | LETM1-like |
| CWSNP7435 | scaffold151 | 275743 | [T/A] | Ca25631 | Non-Synonymous-CDS | LETM1-like |
| CWSNP7436 | scaffold1521 | 20779 | [T/C] | - | Intergenic | - |
| CWSNP7437 | scaffold1545 | 111476 | [G/A] | Ca23856 | Non-Synonymous-CDS | Phosphoenol pyruvatecarboxylase,active site |
| CWSNP7438 | scaffold1553 | 5115 | [C/T] | Ca27877 | Non-Synonymous-CDS | PeptidaseC48,SUMO/Sentrin/Ubl1 |
| CWSNP7439 | scaffold157 | 100228 | [A/G] | Ca21065 | Synonymous-CDS | RNA recognition motif domain |
| CWSNP7440 | scaffold157 | 100529 | [T/C] | Ca21065 | Non-Synonymous-CDS | RNA recognition motif domain |

| SNP IDs | Chromosomes | Physical positions (bp) | SNPs | Gene accession IDs | Sequence components of genes | Putative functions |
|---|---|---|---|---|---|---|
| CWSNP7441 | scaffold157 | 106331 | [G/A] | Ca21065 | Intron | RNA recognition motif domain |
| CWSNP7442 | scaffold157 | 106349 | [C/T] | Ca21065 | Intron | RNA recognition motif domain |
| CWSNP7443 | scaffold157 | 151081 | [G/A] | Ca21070 | Non-Synonymous-CDS | Zinc finger,DHHC-type,palmitoyltransferase |
| CWSNP7444 | scaffold157 | 151199 | [T/A] | Ca21070 | Intron | Zinc finger,DHHC-type,palmitoyltransferase |
| CWSNP7445 | scaffold157 | 151192 | [T/C] | Ca21070 | Intron | Zinc finger,DHHC-type,palmitoyltransferase |
| CWSNP7446 | scaffold157 | 151177 | [A/G] | Ca21070 | Intron | Zinc finger,DHHC-type,palmitoyltransferase |
| CWSNP7447 | scaffold157 | 151131 | [T/A] | Ca21070 | Synonymous-CDS | Zinc finger,DHHC-type,palmitoyltransferase |
| CWSNP7448 | scaffold157 | 213380 | [C/G] | Ca21071 | Non-Synonymous-CDS | Mitochondrial substrate/solute carrier |
| CWSNP7449 | scaffold157 | 213398 | [T/G] | Ca21071 | Non-Synonymous-CDS | Mitochondrial substrate/solute carrier |
| CWSNP7450 | scaffold157 | 213404 | [T/G] | Ca21071 | Synonymous-CDS | Mitochondrial substrate/solute carrier |
| CWSNP7451 | scaffold157 | 213518 | [T/C] | Ca21071 | Intron | Mitochondrial substrate/solute carrier |
| CWSNP7452 | scaffold157 | 213487 | [G/C] | Ca21071 | Intron | Mitochondrial substrate/solute carrier |
| CWSNP7453 | scaffold157 | 213477 | [G/A] | Ca21071 | Intron | Mitochondrial substrate/solute carrier |
| CWSNP7454 | scaffold157 | 229917 | [G/A] | - | Intergenic | - |
| CWSNP7455 | scaffold157 | 229900 | [G/A] | - | Intergenic | - |
| CWSNP7456 | scaffold157 | 321386 | [C/T] | - | Intergenic | - |

| SNP IDs | Chromosomes | Physical positions (bp) | SNPs | Gene accession IDs | Sequence components of genes | Putative functions |
|---|---|---|---|---|---|---|
| CWSNP7457 | scaffold157 | 326434 | [A/G] | Ca21078 | Intron | Protein of unknown function DUF3755 |
| CWSNP7458 | scaffold157 | 326467 | [C/T] | Ca21078 | Intron | Protein of unknown function DUF3755 |
| CWSNP7459 | scaffold157 | 326494 | [A/G] | Ca21078 | Intron | Protein of unknown function DUF3755 |
| CWSNP7460 | scaffold157 | 326497 | [A/G] | Ca21078 | Intron | Protein of unknown function DUF3755 |
| CWSNP7461 | scaffold157 | 352687 | [C/T] | Ca21079 | Intron | DEPdomain |
| CWSNP7462 | scaffold157 | 352712 | [G/A] | Ca21079 | Intron | DEPdomain |
| CWSNP7463 | scaffold157 | 352747 | [T/C] | Ca21079 | Intron | DEPdomain |
| CWSNP7464 | scaffold157 | 355222 | [G/A] | Ca21079 | Intron | DEPdomain |
| CWSNP7465 | scaffold157 | 469198 | [A/C] | - | Intergenic | - |
| CWSNP7466 | scaffold157 | 535057 | [T/G] | - | Intergenic | - |
| CWSNP7467 | scaffold1575 | 2230 | [C/T] | - | Intergenic | - |
| CWSNP7468 | scaffold1580 | 71861 | [G/T] | - | Intergenic | - |
| CWSNP7469 | scaffold1603 | 11828 | [C/G] | - | Intergenic | - |
| CWSNP7470 | scaffold1613 | 48917 | [T/G] | Ca27472 | Intron | Protein kinase, catalytic domain |
| CWSNP7471 | scaffold1613 | 107750 | [T/A] | Ca27474 | Non-Synonymous-CDS | Photosyntheticreactioncentre,L/M |
| CWSNP7472 | scaffold1635 | 83227 | [G/C] | - | Intergenic | - |

| SNP IDs | Chromosomes | Physical positions (bp) | SNPs | Gene accession IDs | Sequence components of genes | Putative functions |
|---|---|---|---|---|---|---|
| CWSNP7473 | scaffold1635 | 83345 | [A/T] | - | Intergenic | - |
| CWSNP7474 | scaffold1654 | 26432 | [G/A] | - | Intergenic | - |
| CWSNP7475 | scaffold1659 | 303945 | [T/G] | - | Intergenic | - |
| CWSNP7476 | scaffold1661 | 70434 | [A/G] | - | Intergenic | - |
| CWSNP7477 | scaffold1664 | 94984 | [A/T] | - | Intergenic | - |
| CWSNP7478 | scaffold167 | 11018 | [T/A] | - | Intergenic | - |
| CWSNP7479 | scaffold167 | 11009 | [A/T] | - | Intergenic | - |
| CWSNP7480 | scaffold167 | 137869 | [C/T] | - | Intergenic | - |
| CWSNP7481 | scaffold167 | 137848 | [A/G] | - | Intergenic | - |
| CWSNP7482 | scaffold167 | 239687 | [G/A] | - | Intergenic | - |
| CWSNP7483 | scaffold1687 | 82935 | [A/C] | Ca26679 | Synonymous-CDS | Plant lipidtransfer protein/Parallergen |
| CWSNP7484 | scaffold1699 | 27556 | [A/C] | - | Intergenic | - |
| CWSNP7485 | scaffold1699 | 27607 | [T/G] | - | Intergenic | - |
| CWSNP7486 | scaffold171 | 96763 | [T/C] | Ca25945 | Synonymous-CDS | - |
| CWSNP7487 | scaffold171 | 183718 | [T/C] | Ca25948 | Intron | Short-chain dehydrogenase/reductaseSDR |
| CWSNP7488 | scaffold171 | 183720 | [C/T] | Ca25948 | Intron | Short-chain dehydrogenase/reductaseSDR |

| SNP IDs | Chromosomes | Physical positions (bp) | SNPs | Gene accession IDs | Sequence components of genes | Putative functions |
|---|---|---|---|---|---|---|
| CWSNP7489 | scaffold171 | 183721 | [G/A] | Ca25948 | Intron | Short-chain dehydrogenase/reductaseSDR |
| CWSNP7490 | scaffold171 | 183727 | [C/T] | Ca25948 | Intron | Short-chain dehydrogenase/reductaseSDR |
| CWSNP7491 | scaffold171 | 183759 | [T/C] | Ca25948 | Intron | Short-chain dehydrogenase/reductaseSDR |
| CWSNP7492 | scaffold171 | 186125 | [C/A] | Ca25948 | Non-Synonymous-CDS | Short-chain dehydrogenase/reductaseSDR |
| CWSNP7493 | scaffold171 | 208050 | [A/G] | - | Intergenic | - |
| CWSNP7494 | scaffold171 | 209964 | [G/T] | - | Intergenic | - |
| CWSNP7495 | scaffold1718 | 302 | [G/A] | - | Intergenic | - |
| CWSNP7496 | scaffold1718 | 295 | [G/A] | - | Intergenic | - |
| CWSNP7497 | scaffold1718 | 268 | [C/G] | - | Intergenic | - |
| CWSNP7498 | scaffold1718 | 3898 | [G/C] | - | Intergenic | - |
| CWSNP7499 | scaffold1718 | 21521 | [A/G] | Ca27100 | Intron | Protein kinase, catalytic domain |
| CWSNP7500 | scaffold1718 | 40669 | [T/C] | - | Intergenic | - |
| CWSNP7501 | scaffold1722 | 65999 | [C/A] | Ca26630 | Intron | Ribulose-phosphate3-epimerase |
| CWSNP7502 | scaffold1722 | 135589 | [C/T] | - | Intergenic | - |
| CWSNP7503 | scaffold1728 | 33301 | [A/C] | - | Intergenic | - |
| CWSNP7504 | scaffold174 | 44014 | [T/G] | - | URR | - |

| SNP IDs | Chromosomes | Physical positions (bp) | SNPs | Gene accession IDs | Sequence components of genes | Putative functions |
|---|---|---|---|---|---|---|
| CWSNP7505 | scaffold174 | 61454 | [T/C] | Ca21706 | Non-Synonymous-CDS | Protein kinase, catalytic domain |
| CWSNP7506 | scaffold174 | 263899 | [G/A] | Ca21715 | Non-Synonymous-CDS | RNA polymerase,alpha subunit |
| CWSNP7507 | scaffold174 | 335077 | [C/T] | Ca21719 | Intron | RNA polymerase,alpha subunit |
| CWSNP7508 | scaffold174 | 341322 | [C/T] | Ca21719 | Intron | RNA polymerase,alpha subunit |
| CWSNP7509 | scaffold174 | 457627 | [G/A] | Ca21723 | Intron | - |
| CWSNP7510 | scaffold1750 | 15510 | [C/T] | Ca24921 | Synonymous-CDS | UBA/THIF-typeNAD/FAD binding fold |
| CWSNP7511 | scaffold1751 | 192417 | [C/A] | - | Intergenic | - |
| CWSNP7512 | scaffold1751 | 192713 | [G/T] | - | Intergenic | - |
| CWSNP7513 | scaffold1751 | 193013 | [T/G] | - | Intergenic | - |
| CWSNP7514 | scaffold1751 | 266583 | [G/A] | - | Intergenic | - |
| CWSNP7515 | scaffold1751 | 496626 | [G/A] | Ca23938 | Non-Synonymous-CDS | - |
| CWSNP7516 | scaffold1751 | 528896 | [A/C] | - | Intergenic | - |
| CWSNP7517 | scaffold1751 | 528979 | [T/C] | - | Intergenic | - |
| CWSNP7518 | scaffold1751 | 625362 | [C/T] | - | Intergenic | - |
| CWSNP7519 | scaffold1751 | 625355 | [C/T] | - | Intergenic | - |
| CWSNP7520 | scaffold1751 | 625342 | [C/T] | - | Intergenic | - |

| SNP IDs | Chromosomes | Physical positions (bp) | SNPs | Gene accession IDs | Sequence components of genes | Putative functions |
|---|---|---|---|---|---|---|
| CWSNP7521 | scaffold1751 | 625335 | [G/A] | - | Intergenic | - |
| CWSNP7522 | scaffold1751 | 625395 | [A/G] | - | Intergenic | - |
| CWSNP7523 | scaffold1751 | 625394 | [C/T] | - | Intergenic | - |
| CWSNP7524 | scaffold1751 | 625389 | [A/C] | - | Intergenic | - |
| CWSNP7525 | scaffold1751 | 625378 | [G/A] | - | Intergenic | - |
| CWSNP7526 | scaffold1751 | 625375 | [G/A] | - | Intergenic | - |
| CWSNP7527 | scaffold1751 | 625366 | [T/C] | - | Intergenic | - |
| CWSNP7528 | scaffold1751 | 625365 | [T/C] | - | Intergenic | - |
| CWSNP7529 | scaffold1751 | 625332 | [A/C] | - | Intergenic | - |
| CWSNP7530 | scaffold1751 | 625396 | [G/A] | - | Intergenic | - |
| CWSNP7531 | scaffold1777 | 164730 | [T/G] | - | Intergenic | - |
| CWSNP7532 | scaffold1777 | 169037 | [G/T] | - | Intergenic | - |
| CWSNP7533 | scaffold1777 | 343173 | [C/T] | - | Intergenic | - |
| CWSNP7534 | scaffold1777 | 343185 | [G/A] | - | Intergenic | - |
| CWSNP7535 | scaffold1777 | 448166 | [A/G] | - | Intergenic | - |
| CWSNP7536 | scaffold1777 | 448176 | [C/T] | - | Intergenic | - |

| SNP IDs | Chromosomes | Physical positions (bp) | SNPs | Gene accession IDs | Sequence components of genes | Putative functions |
|---|---|---|---|---|---|---|
| CWSNP7537 | scaffold1777 | 448187 | [G/T] | - | Intergenic | - |
| CWSNP7538 | scaffold1777 | 448216 | [C/T] | - | Intergenic | - |
| CWSNP7539 | scaffold1779 | 957 | [G/A] | - | DRR | - |
| CWSNP7540 | scaffold1779 | 890 | [G/A] | - | DRR | - |
| CWSNP7541 | scaffold1802 | 17068 | [A/G] | - | Intergenic | - |
| CWSNP7542 | scaffold1802 | 17033 | [A/G] | - | Intergenic | - |
| CWSNP7543 | scaffold1802 | 17111 | [C/T] | - | Intergenic | - |
| CWSNP7544 | scaffold1802 | 17117 | [T/C] | - | Intergenic | - |
| CWSNP7545 | scaffold1802 | 17120 | [C/T] | - | Intergenic | - |
| CWSNP7546 | scaffold1802 | 17123 | [G/T] | - | Intergenic | - |
| CWSNP7547 | scaffold1802 | 17132 | [C/T] | - | Intergenic | - |
| CWSNP7548 | scaffold1802 | 17150 | [T/C] | - | Intergenic | - |
| CWSNP7549 | scaffold1802 | 17151 | [T/C] | - | Intergenic | - |
| CWSNP7550 | scaffold1802 | 17177 | [T/A] | - | Intergenic | - |
| CWSNP7551 | scaffold1835 | 9398 | [C/A] | - | Intergenic | - |
| CWSNP7552 | scaffold1835 | 10541 | [C/G] | - | Intergenic | - |

| SNP IDs | Chromosomes | Physical positions (bp) | SNPs | Gene accession IDs | Sequence components of genes | Putative functions |
|---|---|---|---|---|---|---|
| CWSNP7553 | scaffold1835 | 10563 | [T/C] | - | Intergenic | - |
| CWSNP7554 | scaffold1844 | 41899 | [C/A] | - | DRR | - |
| CWSNP7555 | scaffold1845 | 16292 | [C/G] | - | Intergenic | - |
| CWSNP7556 | scaffold1845 | 16317 | [C/G] | - | Intergenic | - |
| CWSNP7557 | scaffold1845 | 40886 | [T/A] | - | Intergenic | - |
| CWSNP7558 | scaffold1845 | 40872 | [T/G] | - | Intergenic | - |
| CWSNP7559 | scaffold1845 | 40871 | [T/A] | - | Intergenic | - |
| CWSNP7560 | scaffold1845 | 40842 | [T/G] | - | Intergenic | - |
| CWSNP7561 | scaffold1845 | 40821 | [T/G] | - | Intergenic | - |
| CWSNP7562 | scaffold1848 | 583 | [A/G] | - | Intergenic | - |
| CWSNP7563 | scaffold1848 | 565 | [T/C] | - | Intergenic | - |
| CWSNP7564 | scaffold1848 | 562 | [G/C] | - | Intergenic | - |
| CWSNP7565 | scaffold1848 | 532 | [G/C] | - | Intergenic | - |
| CWSNP7566 | scaffold186 | 11965 | [A/G] | - | Intergenic | - |
| CWSNP7567 | scaffold186 | 279942 | [A/G] | - | Intergenic | - |
| CWSNP7568 | scaffold186 | 280062 | [T/C] | - | Intergenic | - |

| SNP IDs | Chromosomes | Physical positions (bp) | SNPs | Gene accession IDs | Sequence components of genes | Putative functions |
|---|---|---|---|---|---|---|
| CWSNP7569 | scaffold186 | 280047 | [A/G] | - | Intergenic | - |
| CWSNP7570 | scaffold186 | 280023 | [C/T] | - | Intergenic | - |
| CWSNP7571 | scaffold186 | 280015 | [T/C] | - | Intergenic | - |
| CWSNP7572 | scaffold186 | 280009 | [G/T] | - | Intergenic | - |
| CWSNP7573 | scaffold1899 | 187 | [A/G] | - | Intergenic | - |
| CWSNP7574 | scaffold1918 | 216231 | [T/G] | - | Intergenic | - |
| CWSNP7575 | scaffold1918 | 216180 | [C/A] | - | Intergenic | - |
| CWSNP7576 | scaffold1918 | 245929 | [T/C] | Ca24715 | Synonymous-CDS | Trehalose-phosphatase |
| CWSNP7577 | scaffold1928 | 103092 | [A/G] | - | Intergenic | - |
| CWSNP7578 | scaffold1928 | 103114 | [G/A] | - | Intergenic | - |
| CWSNP7579 | scaffold193 | 38279 | [C/A] | - | URR | - |
| CWSNP7580 | scaffold193 | 186538 | [C/T] | - | Intergenic | - |
| CWSNP7581 | scaffold193 | 186523 | [T/C] | - | Intergenic | - |
| CWSNP7582 | scaffold193 | 186515 | [T/G] | - | Intergenic | - |
| CWSNP7583 | scaffold193 | 186510 | [C/A] | - | Intergenic | - |
| CWSNP7584 | scaffold193 | 186582 | [C/A] | - | Intergenic | - |

| SNP IDs | Chromosomes | Physical positions (bp) | SNPs | Gene accession IDs | Sequence components of genes | Putative functions |
|---|---|---|---|---|---|---|
| CWSNP7585 | scaffold193 | 186587 | [G/T] | - | Intergenic | - |
| CWSNP7586 | scaffold193 | 186588 | [C/G] | - | Intergenic | - |
| CWSNP7587 | scaffold193 | 186595 | [C/T] | - | Intergenic | - |
| CWSNP7588 | scaffold193 | 186598 | [A/G] | - | Intergenic | - |
| CWSNP7589 | scaffold193 | 186611 | [G/A] | - | Intergenic | - |
| CWSNP7590 | scaffold193 | 186639 | [C/T] | - | Intergenic | - |
| CWSNP7591 | scaffold193 | 186651 | [T/C] | - | Intergenic | - |
| CWSNP7592 | scaffold193 | 186657 | [G/A] | - | Intergenic | - |
| CWSNP7593 | scaffold193 | 186661 | [T/C] | - | Intergenic | - |
| CWSNP7594 | scaffold193 | 242763 | [T/C] | - | Intergenic | - |
| CWSNP7595 | scaffold193 | 242733 | [G/T] | - | Intergenic | - |
| CWSNP7596 | scaffold1943 | 163784 | [G/A] | - | Intergenic | - |
| CWSNP7597 | scaffold1943 | 163780 | [C/T] | - | Intergenic | - |
| CWSNP7598 | scaffold1959 | 84160 | [G/A] | - | Intergenic | - |
| CWSNP7599 | scaffold1964 | 54613 | [G/A] | - | Intergenic | - |
| CWSNP7600 | scaffold1964 | 54604 | [T/C] | - | Intergenic | - |

| SNP IDs | Chromosomes | Physical positions (bp) | SNPs | Gene accession IDs | Sequence components of genes | Putative functions |
|---|---|---|---|---|---|---|
| CWSNP7601 | scaffold198 | 631160 | [G/A] | - | Intergenic | - |
| CWSNP7602 | scaffold198 | 631174 | [C/T] | - | Intergenic | - |
| CWSNP7603 | scaffold198 | 631185 | [G/A] | - | Intergenic | - |
| CWSNP7604 | scaffold198 | 641266 | [C/T] | - | Intergenic | - |
| CWSNP7605 | scaffold198 | 641252 | [A/G] | - | Intergenic | - |
| CWSNP7606 | scaffold198 | 641243 | [A/G] | - | Intergenic | - |
| CWSNP7607 | scaffold1981 | 265110 | [A/C] | - | Intergenic | - |
| CWSNP7608 | scaffold1981 | 265126 | [G/T] | - | Intergenic | - |
| CWSNP7609 | scaffold1981 | 265216 | [A/G] | - | Intergenic | - |
| CWSNP7610 | scaffold1981 | 269879 | [A/G] | - | Intergenic | - |
| CWSNP7611 | scaffold1981 | 385683 | [T/A] | Ca21269 | Synonymous-CDS | Nucleotidyltransferase domain |
| CWSNP7612 | scaffold1984 | 212011 | [G/A] | Ca25764 | Non-Synonymous-CDS | Leo1-like protein |
| CWSNP7613 | scaffold1984 | 215969 | [G/C] | Ca25764 | Intron | Leo1-like protein |
| CWSNP7614 | scaffold1984 | 292507 | [A/C] | - | Intergenic | - |
| CWSNP7615 | scaffold1984 | 292511 | [G/C] | - | Intergenic | - |
| CWSNP7616 | scaffold1985 | 98611 | [C/T] | - | DRR | - |

| SNP IDs | Chromosomes | Physical positions (bp) | SNPs | Gene accession IDs | Sequence components of genes | Putative functions |
|---|---|---|---|---|---|---|
| CWSNP7617 | scaffold1991 | 62009 | [C/A] | - | Intergenic | - |
| CWSNP7618 | scaffold1991 | 62121 | [G/A] | - | Intergenic | - |
| CWSNP7619 | scaffold1991 | 62085 | [A/C] | - | Intergenic | - |
| CWSNP7620 | scaffold1991 | 62131 | [G/A] | - | Intergenic | - |
| CWSNP7621 | scaffold1991 | 62154 | [T/A] | - | Intergenic | - |
| CWSNP7622 | scaffold1991 | 62147 | [G/A] | - | Intergenic | - |
| CWSNP7623 | scaffold2 | 137309 | [T/G] | - | Intergenic | - |
| CWSNP7624 | scaffold2 | 504258 | [C/T] | - | Intergenic | - |
| CWSNP7625 | scaffold2 | 504253 | [C/T] | - | Intergenic | - |
| CWSNP7626 | scaffold2 | 504248 | [G/A] | - | Intergenic | - |
| CWSNP7627 | scaffold202 | 92532 | [C/A] | Ca27126 | Intron | Vacuolar protein sorting-associated protein |
| CWSNP7628 | scaffold202 | 96456 | [T/C] | Ca27126 | Intron | Vacuolar protein sorting-associated protein |
| CWSNP7629 | scaffold202 | 96444 | [G/A] | Ca27126 | Intron | Vacuolar protein sorting-associated protein |
| CWSNP7630 | scaffold202 | 96532 | [G/T] | Ca27126 | Intron | Vacuolar protein sorting-associated protein |
| CWSNP7631 | scaffold202 | 96589 | [A/T] | Ca27126 | Intron | Vacuolar protein sorting-associated protein |
| CWSNP7632 | scaffold202 | 96580 | [T/G] | Ca27126 | Intron | Vacuolar protein sorting-associated protein |

| SNP IDs | Chromosomes | Physical positions (bp) | SNPs | Gene accession IDs | Sequence components of genes | Putative functions |
|---|---|---|---|---|---|---|
| CWSNP7633 | scaffold202 | 99123 | [C/T] | - | Intergenic | - |
| CWSNP7634 | scaffold2027 | 152763 | [C/A] | - | DRR | - |
| CWSNP7635 | scaffold2030 | 13855 | [G/A] | - | Intergenic | - |
| CWSNP7636 | scaffold2030 | 13807 | [G/A] | - | Intergenic | - |
| CWSNP7637 | scaffold2030 | 13889 | [C/T] | - | Intergenic | - |
| CWSNP7638 | scaffold2030 | 13874 | [G/T] | - | Intergenic | - |
| CWSNP7639 | scaffold2033 | 118779 | [G/T] | Ca26141 | Intron | - |
| CWSNP7640 | scaffold2040 | 139597 | [A/G] | - | Intergenic | - |
| CWSNP7641 | scaffold2040 | 139584 | [G/A] | - | Intergenic | - |
| CWSNP7642 | scaffold2040 | 139581 | [G/A] | - | Intergenic | - |
| CWSNP7643 | scaffold2040 | 139560 | [G/T] | - | Intergenic | - |
| CWSNP7644 | scaffold2040 | 139548 | [C/T] | - | Intergenic | - |
| CWSNP7645 | scaffold2040 | 139544 | [C/A] | - | Intergenic | - |
| CWSNP7646 | scaffold2040 | 139541 | [T/C] | - | Intergenic | - |
| CWSNP7647 | scaffold2040 | 139538 | [G/A] | - | Intergenic | - |
| CWSNP7648 | scaffold2040 | 139545 | [G/A] | - | Intergenic | - |

| SNP IDs | Chromosomes | Physical positions (bp) | SNPs | Gene accession IDs | Sequence components of genes | Putative functions |
|---|---|---|---|---|---|---|
| CWSNP7649 | scaffold2040 | 139603 | [C/T] | - | Intergenic | - |
| CWSNP7650 | scaffold205 | 74448 | [T/G] | - | Intergenic | - |
| CWSNP7651 | scaffold205 | 74430 | [A/G] | - | Intergenic | - |
| CWSNP7652 | scaffold205 | 74397 | [G/A] | - | Intergenic | - |
| CWSNP7653 | scaffold205 | 74399 | [C/T] | - | Intergenic | - |
| CWSNP7654 | scaffold205 | 74424 | [T/C] | - | Intergenic | - |
| CWSNP7655 | scaffold205 | 74432 | [G/A] | - | Intergenic | - |
| CWSNP7656 | scaffold206 | 58349 | [C/A] | Ca27283 | Intron | PeptidaseC48,SUMO/Sentrin/Ubl1 |
| CWSNP7657 | scaffold206 | 62985 | [G/A] | Ca27283 | Non-Synonymous-CDS | PeptidaseC48,SUMO/Sentrin/Ubl1 |
| CWSNP7658 | scaffold208 | 344503 | [G/T] | - | Intergenic | - |
| CWSNP7659 | scaffold208 | 344515 | [A/G] | - | Intergenic | - |
| CWSNP7660 | scaffold208 | 344519 | [A/T] | - | Intergenic | - |
| CWSNP7661 | scaffold208 | 344533 | [C/T] | - | Intergenic | - |
| CWSNP7662 | scaffold208 | 344556 | [T/G] | - | Intergenic | - |
| CWSNP7663 | scaffold210 | 293799 | [G/A] | Ca21905 | Non-Synonymous-CDS | - |
| CWSNP7664 | scaffold210 | 355284 | [G/T] | Ca21906 | Non-Synonymous-CDS | Mitochondrial carrier protein |

| SNP IDs | Chromosomes | Physical positions (bp) | SNPs | Gene accession IDs | Sequence components of genes | Putative functions |
|---|---|---|---|---|---|---|
| CWSNP7665 | scaffold210 | 533416 | [G/A] | Ca21912 | Non-Synonymous-CDS | PeptidaseC48,SUMO/Sentrin/Ubl1 |
| CWSNP7666 | scaffold2104 | 61757 | [T/C] | - | Intergenic | - |
| CWSNP7667 | scaffold2104 | 61745 | [T/G] | - | Intergenic | - |
| CWSNP7668 | scaffold2104 | 61723 | [G/A] | - | Intergenic | - |
| CWSNP7669 | scaffold2104 | 61710 | [C/T] | - | Intergenic | - |
| CWSNP7670 | scaffold2104 | 61705 | [C/T] | - | Intergenic | - |
| CWSNP7671 | scaffold2152 | 372404 | [A/T] | - | Intergenic | - |
| CWSNP7672 | scaffold2166 | 228653 | [G/C] | - | Intergenic | - |
| CWSNP7673 | scaffold2170 | 4821 | [G/A] | - | Intergenic | - |
| CWSNP7674 | scaffold221 | 53841 | [T/G] | Ca27612 | Synonymous-CDS | AMP-dependent synthetase/ligase |
| CWSNP7675 | scaffold2248 | 5538 | [A/T] | - | Intergenic | - |
| CWSNP7676 | scaffold2248 | 5562 | [C/T] | - | Intergenic | - |
| CWSNP7677 | scaffold2248 | 5570 | [T/C] | - | Intergenic | - |
| CWSNP7678 | scaffold2248 | 5520 | [C/A] | - | Intergenic | - |
| CWSNP7679 | scaffold2249 | 4446 | [A/C] | - | DRR | - |
| CWSNP7680 | scaffold2269 | 58091 | [A/T] | - | URR | - |

| SNP IDs | Chromosomes | Physical positions (bp) | SNPs | Gene accession IDs | Sequence components of genes | Putative functions |
|---|---|---|---|---|---|---|
| CWSNP7681 | scaffold2269 | 123799 | [A/C] | Ca27079 | Synonymous-CDS | RNA recognition motif domain |
| CWSNP7682 | scaffold2269 | 126013 | [A/C] | Ca27079 | Synonymous-CDS | RNA recognition motif domain |
| CWSNP7683 | scaffold2301 | 70721 | [A/G] | - | Intergenic | - |
| CWSNP7684 | scaffold2330 | 65482 | [T/G] | - | URR | - |
| CWSNP7685 | scaffold2330 | 65617 | [G/A] | - | Intergenic | - |
| CWSNP7686 | scaffold2330 | 83112 | [A/G] | - | DRR | - |
| CWSNP7687 | scaffold2371 | 139149 | [A/G] | - | Intergenic | - |
| CWSNP7688 | scaffold2373 | 3815 | [T/A] | - | Intergenic | - |
| CWSNP7689 | scaffold2373 | 3811 | [C/T] | - | Intergenic | - |
| CWSNP7690 | scaffold2373 | 3793 | [C/T] | - | Intergenic | - |
| CWSNP7691 | scaffold2373 | 3923 | [T/C] | - | Intergenic | - |
| CWSNP7692 | scaffold2373 | 3871 | [A/T] | - | Intergenic | - |
| CWSNP7693 | scaffold2373 | 3862 | [C/T] | - | Intergenic | - |
| CWSNP7694 | scaffold2373 | 12916 | [C/A] | - | Intergenic | - |
| CWSNP7695 | scaffold2373 | 12969 | [G/C] | - | Intergenic | - |
| CWSNP7696 | scaffold2373 | 12978 | [A/T] | - | Intergenic | - |

| SNP IDs | Chromosomes | Physical positions (bp) | SNPs | Gene accession IDs | Sequence components of genes | Putative functions |
|---|---|---|---|---|---|---|
| CWSNP7697 | scaffold2373 | 62495 | [C/T] | - | Intergenic | - |
| CWSNP7698 | scaffold2373 | 62494 | [A/G] | - | Intergenic | - |
| CWSNP7699 | scaffold2373 | 62473 | [G/A] | - | Intergenic | - |
| CWSNP7700 | scaffold2373 | 62433 | [A/G] | - | Intergenic | - |
| CWSNP7701 | scaffold2373 | 127884 | [A/C] | - | Intergenic | - |
| CWSNP7702 | scaffold2373 | 127851 | [C/T] | - | Intergenic | - |
| CWSNP7703 | scaffold2373 | 127905 | [C/T] | - | Intergenic | - |
| CWSNP7704 | scaffold2373 | 127870 | [T/G] | - | Intergenic | - |
| CWSNP7705 | scaffold2373 | 127855 | [A/C] | - | Intergenic | - |
| CWSNP7706 | scaffold2392 | 35402 | [A/C] | Ca25129 | Intron | - |
| CWSNP7707 | scaffold2392 | 198120 | [G/T] | - | DRR | - |
| CWSNP7708 | scaffold2392 | 314322 | [T/C] | - | Intergenic | - |
| CWSNP7709 | scaffold2392 | 314323 | [G/A] | - | Intergenic | - |
| CWSNP7710 | scaffold2392 | 314343 | [G/A] | - | Intergenic | - |
| CWSNP7711 | scaffold2392 | 314344 | [T/C] | - | Intergenic | - |
| CWSNP7712 | scaffold2392 | 314349 | [C/G] | - | Intergenic | - |

| SNP IDs | Chromosomes | Physical positions (bp) | SNPs | Gene accession IDs | Sequence components of genes | Putative functions |
|---|---|---|---|---|---|---|
| CWSNP7713 | scaffold2392 | 314353 | [G/A] | - | Intergenic | - |
| CWSNP7714 | scaffold2392 | 314401 | [A/C] | - | Intergenic | - |
| CWSNP7715 | scaffold240 | 6231 | [T/A] | - | Intergenic | - |
| CWSNP7716 | scaffold240 | 6193 | [G/A] | - | Intergenic | - |
| CWSNP7717 | scaffold240 | 6152 | [C/T] | - | Intergenic | - |
| CWSNP7718 | scaffold240 | 119157 | [G/A] | Ca25659 | Synonymous-CDS | PeptidaseC48,SUMO/Sentrin/Ubl1 |
| CWSNP7719 | scaffold240 | 119301 | [T/C] | Ca25659 | Synonymous-CDS | PeptidaseC48,SUMO/Sentrin/Ubl1 |
| CWSNP7720 | scaffold240 | 119297 | [C/A] | Ca25659 | Non-Synonymous-CDS | PeptidaseC48,SUMO/Sentrin/Ubl1 |
| CWSNP7721 | scaffold240 | 119278 | [T/C] | Ca25659 | Non-Synonymous-CDS | PeptidaseC48,SUMO/Sentrin/Ubl1 |
| CWSNP7722 | scaffold240 | 119269 | [C/T] | Ca25659 | Non-Synonymous-CDS | PeptidaseC48,SUMO/Sentrin/Ubl1 |
| CWSNP7723 | scaffold240 | 119267 | [G/C] | Ca25659 | Non-Synonymous-CDS | PeptidaseC48,SUMO/Sentrin/Ubl1 |
| CWSNP7724 | scaffold240 | 119265 | [C/G] | Ca25659 | Synonymous-CDS | PeptidaseC48,SUMO/Sentrin/Ubl1 |
| CWSNP7725 | scaffold242 | 293872 | [A/G] | Ca22265 | Intron | Raffinosesynthase |
| CWSNP7726 | scaffold250 | 38270 | [A/G] | Ca27037 | Intron | SH2motif |
| CWSNP7727 | scaffold2501 | 287 | [C/T] | - | Intergenic | - |
| CWSNP7728 | scaffold2501 | 282 | [C/A] | - | Intergenic | - |

| SNP IDs | Chromosomes | Physical positions (bp) | SNPs | Gene accession IDs | Sequence components of genes | Putative functions |
|---|---|---|---|---|---|---|
| CWSNP7729 | scaffold2501 | 251 | [T/G] | - | Intergenic | - |
| CWSNP7730 | scaffold2501 | 242 | [C/G] | - | Intergenic | - |
| CWSNP7731 | scaffold2516 | 58947 | [T/C] | Ca26451 | Synonymous-CDS | Domain of unknown function DUF125,transmembrane |
| CWSNP7732 | scaffold2521 | 57762 | [T/C] | Ca27549 | Synonymous-CDS | - |
| CWSNP7733 | scaffold2521 | 57725 | [G/A] | Ca27549 | Non-Synonymous-CDS | - |
| CWSNP7734 | scaffold2521 | 57709 | [G/T] | Ca27549 | Non-Synonymous-CDS | - |
| CWSNP7735 | scaffold2557 | 110378 | [C/T] | Ca26665 | Non-Synonymous-CDS | Domain of unknown function DUF828 |
| CWSNP7736 | scaffold2557 | 110414 | [T/G] | Ca26665 | Non-Synonymous-CDS | Domain of unknown function DUF828 |
| CWSNP7737 | scaffold2557 | 110437 | [C/T] | - | Intergenic | - |
| CWSNP7738 | scaffold2557 | 110441 | [T/A] | - | Intergenic | - |
| CWSNP7739 | scaffold2569 | 58152 | [T/G] | Ca26161 | Synonymous-CDS | WD40 repeat |
| CWSNP7740 | scaffold2617 | 467848 | [C/T] | - | DRR | - |
| CWSNP7741 | scaffold263 | 85006 | [G/A] | - | Intergenic | - |
| CWSNP7742 | scaffold263 | 84970 | [T/C] | - | Intergenic | - |
| CWSNP7743 | scaffold263 | 84952 | [T/G] | - | Intergenic | - |
| CWSNP7744 | scaffold2720 | 177550 | [G/T] | - | Intergenic | - |

| SNP IDs | Chromosomes | Physical positions (bp) | SNPs | Gene accession IDs | Sequence components of genes | Putative functions |
|---|---|---|---|---|---|---|
| CWSNP7745 | scaffold2720 | 177578 | [T/C] | - | Intergenic | - |
| CWSNP7746 | scaffold2722 | 52416 | [G/T] | - | Intergenic | - |
| CWSNP7747 | scaffold2722 | 52397 | [G/T] | - | Intergenic | - |
| CWSNP7748 | scaffold2722 | 75609 | [A/G] | Ca27216 | Non-Synonymous-CDS | Glycosidehydrolase,family28 |
| CWSNP7749 | scaffold273 | 202514 | [T/G] | - | Intergenic | - |
| CWSNP7750 | scaffold2738 | 1307 | [G/A] | - | Intergenic | - |
| CWSNP7751 | scaffold275 | 208067 | [C/A] | - | Intergenic | - |
| CWSNP7752 | scaffold275 | 208786 | [G/C] | - | Intergenic | - |
| CWSNP7753 | scaffold275 | 230606 | [A/G] | - | Intergenic | - |
| CWSNP7754 | scaffold275 | 347422 | [C/A] | - | Intergenic | - |
| CWSNP7755 | scaffold275 | 347384 | [G/A] | - | Intergenic | - |
| CWSNP7756 | scaffold275 | 347361 | [C/T] | - | Intergenic | - |
| CWSNP7757 | scaffold275 | 479497 | [C/T] | - | Intergenic | - |
| CWSNP7758 | scaffold2763 | 4688 | [A/C] | Ca24392 | Synonymous-CDS | Glycosidehydrolase,family9 |
| CWSNP7759 | scaffold2763 | 4706 | [G/T] | Ca24392 | Non-Synonymous-CDS | Glycosidehydrolase,family9 |
| CWSNP7760 | scaffold2763 | 4719 | [G/A] | Ca24392 | Intron | Glycosidehydrolase,family9 |

| SNP IDs | Chromosomes | Physical positions (bp) | SNPs | Gene accession IDs | Sequence components of genes | Putative functions |
|---|---|---|---|---|---|---|
| CWSNP7761 | scaffold2763 | 4732 | [C/G] | Ca24392 | Intron | Glycosidehydrolase,family9 |
| CWSNP7762 | scaffold2763 | 4738 | [C/G] | Ca24392 | Intron | Glycosidehydrolase,family9 |
| CWSNP7763 | scaffold2763 | 4782 | [C/T] | Ca24392 | Intron | Glycosidehydrolase,family9 |
| CWSNP7764 | scaffold2763 | 25756 | [T/G] | Ca24394 | Synonymous-CDS | - |
| CWSNP7765 | scaffold2763 | 25769 | [A/G] | Ca24394 | Non-Synonymous-CDS | - |
| CWSNP7766 | scaffold2763 | 25771 | [T/G] | Ca24394 | Non-Synonymous-CDS | - |
| CWSNP7767 | scaffold2763 | 43207 | [G/A] | - | URR | - |
| CWSNP7768 | scaffold2763 | 49533 | [T/A] | Ca24396 | Intron | Zinc finger,RING-type |
| CWSNP7769 | scaffold2763 | 82193 | [C/A] | - | Intergenic | - |
| CWSNP7770 | scaffold2763 | 82232 | [C/T] | - | Intergenic | - |
| CWSNP7771 | scaffold2763 | 82249 | [G/A] | - | Intergenic | - |
| CWSNP7772 | scaffold2763 | 82250 | [T/C] | - | Intergenic | - |
| CWSNP7773 | scaffold2763 | 82253 | [G/C] | - | Intergenic | - |
| CWSNP7774 | scaffold2763 | 82262 | [G/C] | - | Intergenic | - |
| CWSNP7775 | scaffold2763 | 82261 | [C/G] | - | Intergenic | - |
| CWSNP7776 | scaffold2763 | 82302 | [T/A] | Ca24399 | Non-Synonymous-CDS | - |

| SNP IDs | Chromosomes | Physical positions (bp) | SNPs | Gene accession IDs | Sequence components of genes | Putative functions |
|---|---|---|---|---|---|---|
| CWSNP7777 | scaffold2763 | 82319 | [T/C] | Ca24399 | Synonymous-CDS | - |
| CWSNP7778 | scaffold2763 | 82328 | [A/G] | Ca24399 | Synonymous-CDS | - |
| CWSNP7779 | scaffold2763 | 82342 | [C/T] | Ca24399 | Non-Synonymous-CDS | - |
| CWSNP7780 | scaffold2763 | 82369 | [G/A] | Ca24399 | Non-Synonymous-CDS | - |
| CWSNP7781 | scaffold2763 | 82417 | [C/T] | Ca24399 | Non-Synonymous-CDS | - |
| CWSNP7782 | scaffold2763 | 82397 | [T/C] | Ca24399 | Synonymous-CDS | - |
| CWSNP7783 | scaffold2763 | 95823 | [G/A] | - | Intergenic | - |
| CWSNP7784 | scaffold2763 | 99364 | [A/G] | - | DRR | - |
| CWSNP7785 | scaffold2763 | 99368 | [C/G] | - | DRR | - |
| CWSNP7786 | scaffold2763 | 99549 | [C/A] | - | Intergenic | - |
| CWSNP7787 | scaffold2763 | 104549 | [G/C] | - | DRR | - |
| CWSNP7788 | scaffold2763 | 104583 | [T/C] | Ca24401 | Non-Synonymous-CDS | - |
| CWSNP7789 | scaffold2763 | 107087 | [A/G] | Ca24401 | Intron | - |
| CWSNP7790 | scaffold2763 | 107033 | [T/A] | Ca24401 | Intron | - |
| CWSNP7791 | scaffold2763 | 107437 | [T/C] | Ca24401 | Intron | - |
| CWSNP7792 | scaffold2763 | 107376 | [C/T] | Ca24401 | Intron | - |

| SNP IDs | Chromosomes | Physical positions (bp) | SNPs | Gene accession IDs | Sequence components of genes | Putative functions |
|---|---|---|---|---|---|---|
| CWSNP7793 | scaffold2763 | 107413 | [A/G] | Ca24401 | Intron | - |
| CWSNP7794 | scaffold2763 | 108560 | [T/G] | Ca24401 | Intron | - |
| CWSNP7795 | scaffold2763 | 108599 | [T/A] | Ca24401 | Intron | - |
| CWSNP7796 | scaffold2763 | 108763 | [T/G] | Ca24401 | Intron | - |
| CWSNP7797 | scaffold2763 | 117440 | [C/T] | - | URR | - |
| CWSNP7798 | scaffold2763 | 117430 | [A/C] | - | URR | - |
| CWSNP7799 | scaffold2763 | 125716 | [C/T] | Ca24402 | Synonymous-CDS | - |
| CWSNP7800 | scaffold280 | 90875 | [G/A] | - | Intergenic | - |
| CWSNP7801 | scaffold280 | 90950 | [C/A] | - | Intergenic | - |
| CWSNP7802 | scaffold280 | 99357 | [A/C] | - | Intergenic | - |
| CWSNP7803 | scaffold280 | 111160 | [C/A] | - | Intergenic | - |
| CWSNP7804 | scaffold2812 | 56702 | [G/A] | - | DRR | - |
| CWSNP7805 | scaffold2812 | 89940 | [C/T] | Ca26646 | Synonymous-CDS | Lactate/malatedehydrogenase,N-terminal |
| CWSNP7806 | scaffold2815 | 25626 | [C/G] | - | Intergenic | - |
| CWSNP7807 | scaffold2815 | 25655 | [T/G] | - | Intergenic | - |
| CWSNP7808 | scaffold2815 | 25668 | [T/G] | - | Intergenic | - |

| SNP IDs | Chromosomes | Physical positions (bp) | SNPs | Gene accession IDs | Sequence components of genes | Putative functions |
|---|---|---|---|---|---|---|
| CWSNP7809 | scaffold2827 | 210 | [A/G] | - | Intergenic | - |
| CWSNP7810 | scaffold2827 | 285 | [T/G] | - | Intergenic | - |
| CWSNP7811 | scaffold2827 | 258 | [T/C] | - | Intergenic | - |
| CWSNP7812 | scaffold2827 | 240 | [T/G] | - | Intergenic | - |
| CWSNP7813 | scaffold284 | 522764 | [T/G] | Ca20600 | Synonymous-CDS | Oxidoreductase,molybdopterin-binding domain |
| CWSNP7814 | scaffold284 | 613764 | [G/A] | - | Intergenic | - |
| CWSNP7815 | scaffold284 | 613861 | [C/T] | - | Intergenic | - |
| CWSNP7816 | scaffold284 | 613850 | [C/T] | - | Intergenic | - |
| CWSNP7817 | scaffold284 | 613811 | [G/A] | - | Intergenic | - |
| CWSNP7818 | scaffold284 | 826690 | [T/C] | - | Intergenic | - |
| CWSNP7819 | scaffold284 | 826644 | [G/A] | - | Intergenic | - |
| CWSNP7820 | scaffold2848 | 2403 | [C/A] | Ca27862 | Non-Synonymous-CDS | PeptidaseC48,SUMO/Sentrin/Ubl1 |
| CWSNP7821 | scaffold2848 | 2444 | [C/A] | Ca27862 | Non-Synonymous-CDS | PeptidaseC48,SUMO/Sentrin/Ubl1 |
| CWSNP7822 | scaffold2848 | 2517 | [G/A] | Ca27862 | Non-Synonymous-CDS | PeptidaseC48,SUMO/Sentrin/Ubl1 |
| CWSNP7823 | scaffold2848 | 2464 | [A/G] | Ca27862 | Synonymous-CDS | PeptidaseC48,SUMO/Sentrin/Ubl1 |
| CWSNP7824 | scaffold2853 | 14852 | [C/G] | Ca28160 | Intron | - |

| SNP IDs | Chromosomes | Physical positions (bp) | SNPs | Gene accession IDs | Sequence components of genes | Putative functions |
|---|---|---|---|---|---|---|
| CWSNP7825 | scaffold2853 | 14916 | [A/G] | Ca28160 | Intron | - |
| CWSNP7826 | scaffold2853 | 14917 | [G/A] | Ca28160 | Intron | - |
| CWSNP7827 | scaffold2853 | 15086 | [G/A] | Ca28160 | Intron | - |
| CWSNP7828 | scaffold287 | 3224 | [G/A] | - | Intergenic | - |
| CWSNP7829 | scaffold287 | 163591 | [C/A] | - | Intergenic | - |
| CWSNP7830 | scaffold290 | 179998 | [T/C] | Ca19830 | Intron | Xanthine/uracil/vitaminCpermease |
| CWSNP7831 | scaffold290 | 179993 | [C/T] | Ca19830 | Intron | Xanthine/uracil/vitaminCpermease |
| CWSNP7832 | scaffold290 | 179940 | [G/A] | Ca19830 | Intron | Xanthine/uracil/vitaminCpermease |
| CWSNP7833 | scaffold290 | 179969 | [A/T] | Ca19830 | Intron | Xanthine/uracil/vitaminCpermease |
| CWSNP7834 | scaffold290 | 211634 | [A/C] | Ca19830 | Intron | Xanthine/uracil/vitaminCpermease |
| CWSNP7835 | scaffold290 | 212536 | [A/C] | - | Intergenic | - |
| CWSNP7836 | scaffold290 | 212514 | [C/T] | - | Intergenic | - |
| CWSNP7837 | scaffold290 | 255161 | [G/A] | Ca19833 | Intron | Villinheadpiece |
| CWSNP7838 | scaffold290 | 728010 | [T/A] | - | Intergenic | - |
| CWSNP7839 | scaffold290 | 824906 | [C/T] | - | Intergenic | - |
| CWSNP7840 | scaffold2907 | 31202 | [G/A] | Ca27010 | Non-Synonymous-CDS | - |

| SNP IDs | Chromosomes | Physical positions (bp) | SNPs | Gene accession IDs | Sequence components of genes | Putative functions |
|---|---|---|---|---|---|---|
| CWSNP7841 | scaffold2950 | 32650 | [C/T] | - | Intergenic | - |
| CWSNP7842 | scaffold2950 | 32703 | [A/C] | - | Intergenic | - |
| CWSNP7843 | scaffold2950 | 32739 | [C/A] | - | Intergenic | - |
| CWSNP7844 | scaffold2950 | 32776 | [T/A] | - | Intergenic | - |
| CWSNP7845 | scaffold2950 | 33081 | [A/G] | - | Intergenic | - |
| CWSNP7846 | scaffold2950 | 33207 | [C/T] | - | Intergenic | - |
| CWSNP7847 | scaffold296 | 41422 | [T/G] | Ca20526 | Intron | Glycosylhydrolase,family13,catalytic domain |
| CWSNP7848 | scaffold296 | 59883 | [A/C] | Ca20530 | Synonymous-CDS | Alanineracemase,N-terminal |
| CWSNP7849 | scaffold299 | 199050 | [A/T] | - | Intergenic | - |
| CWSNP7850 | scaffold299 | 199067 | [A/T] | - | URR | - |
| CWSNP7851 | scaffold299 | 199068 | [G/T] | - | Intergenic | - |
| CWSNP7852 | scaffold299 | 199073 | [G/A] | - | Intergenic | - |
| CWSNP7853 | scaffold299 | 204540 | [G/A] | - | DRR | - |
| CWSNP7854 | scaffold300 | 111914 | [C/T] | Ca27306 | Non-Synonymous-CDS | - |
| CWSNP7855 | scaffold300 | 111924 | [C/T] | Ca27306 | Non-Synonymous-CDS | - |
| CWSNP7856 | scaffold300 | 111926 | [G/A] | Ca27306 | Non-Synonymous-CDS | - |

| SNP IDs | Chromosomes | Physical positions (bp) | SNPs | Gene accession IDs | Sequence components of genes | Putative functions |
|---|---|---|---|---|---|---|
| CWSNP7857 | scaffold300 | 111933 | [C/T] | Ca27306 | Non-Synonymous-CDS | - |
| CWSNP7858 | scaffold300 | 111936 | [C/A] | Ca27306 | Non-Synonymous-CDS | - |
| CWSNP7859 | scaffold300 | 111972 | [A/T] | Ca27306 | Non-Synonymous-CDS | - |
| CWSNP7860 | scaffold300 | 111973 | [A/T] | Ca27306 | Non-Synonymous-CDS | - |
| CWSNP7861 | scaffold300 | 111976 | [C/T] | Ca27306 | Synonymous-CDS | - |
| CWSNP7862 | scaffold300 | 112011 | [T/C] | Ca27306 | Non-Synonymous-CDS | - |
| CWSNP7863 | scaffold300 | 112006 | [G/A] | Ca27306 | Synonymous-CDS | - |
| CWSNP7864 | scaffold300 | 112005 | [G/A] | Ca27306 | Non-Synonymous-CDS | - |
| CWSNP7865 | scaffold300 | 111959 | [C/T] | Ca27306 | Non-Synonymous-CDS | - |
| CWSNP7866 | scaffold300 | 111964 | [C/T] | Ca27306 | Synonymous-CDS | - |
| CWSNP7867 | scaffold3016 | 32790 | [C/T] | Ca26723 | Synonymous-CDS | - |
| CWSNP7868 | scaffold303 | 18213 | [G/A] | - | Intergenic | - |
| CWSNP7869 | scaffold305 | 49263 | [G/A] | - | Intergenic | - |
| CWSNP7870 | scaffold305 | 49266 | [T/G] | - | Intergenic | - |
| CWSNP7871 | scaffold305 | 138929 | [G/A] | - | Intergenic | - |
| CWSNP7872 | scaffold306 | 19215 | [C/A] | - | DRR | - |

| SNP IDs | Chromosomes | Physical positions (bp) | SNPs | Gene accession IDs | Sequence components of genes | Putative functions |
|---|---|---|---|---|---|---|
| CWSNP7873 | scaffold306 | 85993 | [T/A] | - | Intergenic | - |
| CWSNP7874 | scaffold306 | 88389 | [T/A] | - | Intergenic | - |
| CWSNP7875 | scaffold3084 | 4409 | [G/A] | - | Intergenic | - |
| CWSNP7876 | scaffold308_2 | 143813 | [C/G] | Ca10872 | Non-Synonymous-CDS | Nucleoporin,Nsp1-like,C-terminal |
| CWSNP7877 | scaffold308_2 | 144354 | [C/T] | Ca10872 | Synonymous-CDS | Nucleoporin,Nsp1-like,C-terminal |
| CWSNP7878 | scaffold308_2 | 152576 | [G/T] | - | Intergenic | - |
| CWSNP7879 | scaffold311 | 86677 | [C/T] | - | Intergenic | - |
| CWSNP7880 | scaffold3116 | 159 | [T/G] | - | Intergenic | - |
| CWSNP7881 | scaffold314 | 432453 | [T/C] | - | Intergenic | - |
| CWSNP7882 | scaffold314 | 432471 | [A/G] | - | Intergenic | - |
| CWSNP7883 | scaffold314 | 535269 | [A/C] | Ca21812 | Non-Synonymous-CDS | Dual specificity phosphatase,catalytic domain |
| CWSNP7884 | scaffold314 | 535266 | [A/C] | Ca21812 | Non-Synonymous-CDS | Dual specificity phosphatase,catalytic domain |
| CWSNP7885 | scaffold3155 | 7438 | [A/C] | - | Intergenic | - |
| CWSNP7886 | scaffold3155 | 7442 | [T/C] | - | Intergenic | - |
| CWSNP7887 | scaffold3155 | 7443 | [A/G] | - | Intergenic | - |
| CWSNP7888 | scaffold3155 | 7444 | [A/G] | - | Intergenic | - |

| SNP IDs | Chromosomes | Physical positions (bp) | SNPs | Gene accession IDs | Sequence components of genes | Putative functions |
|---|---|---|---|---|---|---|
| CWSNP7889 | scaffold3155 | 7462 | [A/G] | - | Intergenic | - |
| CWSNP7890 | scaffold3155 | 7464 | [T/A] | - | Intergenic | - |
| CWSNP7891 | scaffold3155 | 7472 | [A/G] | - | Intergenic | - |
| CWSNP7892 | scaffold3155 | 7477 | [C/G] | - | Intergenic | - |
| CWSNP7893 | scaffold3155 | 7480 | [G/A] | - | Intergenic | - |
| CWSNP7894 | scaffold3155 | 9404 | [C/T] | Ca27112 | Non-Synonymous-CDS | - |
| CWSNP7895 | scaffold322 | 55475 | [G/A] | - | Intergenic | - |
| CWSNP7896 | scaffold322 | 55479 | [G/T] | - | Intergenic | - |
| CWSNP7897 | scaffold322 | 55589 | [A/T] | - | Intergenic | - |
| CWSNP7898 | scaffold3228 | 2926 | [C/T] | - | Intergenic | - |
| CWSNP7899 | scaffold3228 | 23138 | [A/G] | - | DRR | - |
| CWSNP7900 | scaffold3228 | 23190 | [G/C] | - | DRR | - |
| CWSNP7901 | scaffold3228 | 23315 | [C/A] | - | DRR | - |
| CWSNP7902 | scaffold3228 | 23270 | [A/C] | - | DRR | - |
| CWSNP7903 | scaffold324 | 15569 | [A/C] | Ca24910 | Non-Synonymous-CDS | - |
| CWSNP7904 | scaffold324 | 15574 | [G/A] | Ca24910 | Synonymous-CDS | - |

| SNP IDs | Chromosomes | Physical positions (bp) | SNPs | Gene accession IDs | Sequence components of genes | Putative functions |
|---|---|---|---|---|---|---|
| CWSNP7905 | scaffold324 | 15581 | [G/C] | Ca24910 | Non-Synonymous-CDS | - |
| CWSNP7906 | scaffold324 | 15590 | [G/T] | Ca24910 | Non-Synonymous-CDS | - |
| CWSNP7907 | scaffold324 | 15593 | [G/A] | Ca24910 | Non-Synonymous-CDS | - |
| CWSNP7908 | scaffold324 | 15594 | [C/A] | Ca24910 | Non-Synonymous-CDS | - |
| CWSNP7909 | scaffold324 | 15614 | [C/T] | Ca24910 | Non-Synonymous-CDS | - |
| CWSNP7910 | scaffold324 | 15636 | [G/A] | Ca24910 | Non-Synonymous-CDS | - |
| CWSNP7911 | scaffold3254_2 | 15616 | [A/G] | Ca16296 | Synonymous-CDS | PeptidaseA1 |
| CWSNP7912 | scaffold3254_2 | 186218 | [G/A] | - | Intergenic | - |
| CWSNP7913 | scaffold3254_2 | 186203 | [C/G] | - | Intergenic | - |
| CWSNP7914 | scaffold3254_2 | 186243 | [A/C] | - | Intergenic | - |
| CWSNP7915 | scaffold332 | 203691 | [T/A] | - | Intergenic | - |
| CWSNP7916 | scaffold332 | 204783 | [A/G] | - | Intergenic | - |
| CWSNP7917 | scaffold332 | 204785 | [G/A] | - | DRR | - |
| CWSNP7918 | scaffold332 | 204952 | [G/A] | - | Intergenic | - |
| CWSNP7919 | scaffold3321 | 17935 | [C/A] | - | Intergenic | - |
| CWSNP7920 | scaffold3337 | 17963 | [C/T] | Ca28178 | Non-Synonymous-CDS | Domain of unknown function DUF828 |

| SNP IDs | Chromosomes | Physical positions (bp) | SNPs | Gene accession IDs | Sequence components of genes | Putative functions |
|---|---|---|---|---|---|---|
| CWSNP7921 | scaffold3337 | 17999 | [T/G] | Ca28178 | Non-Synonymous-CDS | Domain of unknown function DUF828 |
| CWSNP7922 | scaffold3337 | 18022 | [C/T] | - | Intergenic | - |
| CWSNP7923 | scaffold3337 | 18026 | [T/A] | - | Intergenic | - |
| CWSNP7924 | scaffold3337 | 17959 | [A/G] | Ca28178 | Non-Synonymous-CDS | Domain of unknown function DUF828 |
| CWSNP7925 | scaffold3337 | 17956 | [G/A] | Ca28178 | Non-Synonymous-CDS | Domain of unknown function DUF828 |
| CWSNP7926 | scaffold334 | 43160 | [G/A] | Ca25777 | Non-Synonymous-CDS | - |
| CWSNP7927 | scaffold335 | 60951 | [T/C] | - | DRR | - |
| CWSNP7928 | scaffold335 | 60952 | [T/C] | - | DRR | - |
| CWSNP7929 | scaffold335 | 60956 | [T/C] | - | DRR | - |
| CWSNP7930 | scaffold335 | 60957 | [T/C] | - | DRR | - |
| CWSNP7931 | scaffold335 | 60960 | [T/C] | - | DRR | - |
| CWSNP7932 | scaffold335 | 60962 | [T/G] | - | DRR | - |
| CWSNP7933 | scaffold335 | 60906 | [T/G] | - | DRR | - |
| CWSNP7934 | scaffold335 | 136017 | [C/G] | Ca26029 | Non-Synonymous-CDS | PeptidaseC48,SUMO/Sentrin/Ubl1 |
| CWSNP7935 | scaffold335 | 135969 | [C/T] | Ca26029 | Non-Synonymous-CDS | PeptidaseC48,SUMO/Sentrin/Ubl1 |
| CWSNP7936 | scaffold335 | 135976 | [C/T] | Ca26029 | Synonymous-CDS | PeptidaseC48,SUMO/Sentrin/Ubl1 |

| SNP IDs | Chromosomes | Physical positions (bp) | SNPs | Gene accession IDs | Sequence components of genes | Putative functions |
|---|---|---|---|---|---|---|
| CWSNP7937 | scaffold335 | 136077 | [G/C] | Ca26029 | Synonymous-CDS | PeptidaseC48,SUMO/Sentrin/Ubl1 |
| CWSNP7938 | scaffold335 | 136076 | [G/C] | Ca26029 | Non-Synonymous-CDS | PeptidaseC48,SUMO/Sentrin/Ubl1 |
| CWSNP7939 | scaffold335 | 136073 | [G/A] | Ca26029 | Non-Synonymous-CDS | PeptidaseC48,SUMO/Sentrin/Ubl1 |
| CWSNP7940 | scaffold335 | 136051 | [T/A] | Ca26029 | Non-Synonymous-CDS | PeptidaseC48,SUMO/Sentrin/Ubl1 |
| CWSNP7941 | scaffold335 | 136030 | [G/A] | Ca26029 | Synonymous-CDS | PeptidaseC48,SUMO/Sentrin/Ubl1 |
| CWSNP7942 | scaffold335 | 136129 | [T/C] | Ca26029 | Synonymous-CDS | PeptidaseC48,SUMO/Sentrin/Ubl1 |
| CWSNP7943 | scaffold335 | 136109 | [G/T] | Ca26029 | Non-Synonymous-CDS | PeptidaseC48,SUMO/Sentrin/Ubl1 |
| CWSNP7944 | scaffold335 | 136106 | [G/A] | Ca26029 | Non-Synonymous-CDS | PeptidaseC48,SUMO/Sentrin/Ubl1 |
| CWSNP7945 | scaffold335 | 136096 | [T/C] | Ca26029 | Synonymous-CDS | PeptidaseC48,SUMO/Sentrin/Ubl1 |
| CWSNP7946 | scaffold335 | 136091 | [G/A] | Ca26029 | Non-Synonymous-CDS | PeptidaseC48,SUMO/Sentrin/Ubl1 |
| CWSNP7947 | scaffold336 | 468863 | [T/A] | Ca21282 | Non-Synonymous-CDS | Protein kinase, catalytic domain |
| CWSNP7948 | scaffold336 | 563363 | [C/T] | - | Intergenic | - |
| CWSNP7949 | scaffold336 | 618427 | [C/T] | Ca21291 | Non-Synonymous-CDS | ABC transporter, transmembrane domain |
| CWSNP7950 | scaffold336 | 771169 | [C/T] | Ca21296 | Non-Synonymous-CDS | PeptidaseC48,SUMO/Sentrin/Ubl1 |
| CWSNP7951 | scaffold3362 | 69878 | [C/A] | Ca26010 | Intron | Protein kinase, catalytic domain |
| CWSNP7952 | scaffold3397 | 45363 | [T/C] | Ca26273 | Non-Synonymous-CDS | WD40 repeat |

| SNP IDs | Chromosomes | Physical positions (bp) | SNPs | Gene accession IDs | Sequence components of genes | Putative functions |
|---|---|---|---|---|---|---|
| CWSNP7953 | scaffold342 | 311411 | [G/A] | - | Intergenic | - |
| CWSNP7954 | scaffold342 | 311394 | [A/C] | - | Intergenic | - |
| CWSNP7955 | scaffold342 | 359376 | [A/C] | Ca24729 | Intron | - |
| CWSNP7956 | scaffold3422 | 13376 | [T/A] | - | Intergenic | - |
| CWSNP7957 | scaffold346_1 | 105461 | [A/G] | - | Intergenic | - |
| CWSNP7958 | scaffold346_1 | 105521 | [A/C] | - | Intergenic | - |
| CWSNP7959 | scaffold346_1 | 105523 | [G/A] | - | Intergenic | - |
| CWSNP7960 | scaffold346_1 | 105546 | [C/T] | - | Intergenic | - |
| CWSNP7961 | scaffold349 | 51381 | [A/T] | - | Intergenic | - |
| CWSNP7962 | scaffold349 | 51396 | [G/A] | - | Intergenic | - |
| CWSNP7963 | scaffold349 | 51414 | [C/T] | - | Intergenic | - |
| CWSNP7964 | scaffold349 | 51435 | [C/T] | - | Intergenic | - |
| CWSNP7965 | scaffold349 | 51548 | [T/C] | - | Intergenic | - |
| CWSNP7966 | scaffold349 | 51637 | [T/A] | - | Intergenic | - |
| CWSNP7967 | scaffold349 | 51624 | [G/A] | - | Intergenic | - |
| CWSNP7968 | scaffold349 | 174039 | [A/T] | - | Intergenic | - |

| SNP IDs | Chromosomes | Physical positions (bp) | SNPs | Gene accession IDs | Sequence components of genes | Putative functions |
|---|---|---|---|---|---|---|
| CWSNP7969 | scaffold3514 | 42134 | [G/A] | Ca26926 | Synonymous-CDS | Lipase,GDSL |
| CWSNP7970 | scaffold3514 | 42135 | [G/T] | Ca26926 | Synonymous-CDS | Lipase,GDSL |
| CWSNP7971 | scaffold3514 | 42156 | [G/A] | Ca26926 | Synonymous-CDS | Lipase,GDSL |
| CWSNP7972 | scaffold3514 | 42163 | [T/C] | Ca26926 | Synonymous-CDS | Lipase,GDSL |
| CWSNP7973 | scaffold3514 | 42164 | [A/G] | Ca26926 | Synonymous-CDS | Lipase,GDSL |
| CWSNP7974 | scaffold3514 | 42175 | [G/A] | Ca26926 | Synonymous-CDS | Lipase,GDSL |
| CWSNP7975 | scaffold352 | 22672 | [C/T] | - | Intergenic | - |
| CWSNP7976 | scaffold352 | 22675 | [G/A] | - | Intergenic | - |
| CWSNP7977 | scaffold352 | 22693 | [C/T] | - | Intergenic | - |
| CWSNP7978 | scaffold352 | 22708 | [A/G] | - | Intergenic | - |
| CWSNP7979 | scaffold352 | 22777 | [T/G] | - | Intergenic | - |
| CWSNP7980 | scaffold352 | 22726 | [A/C] | - | Intergenic | - |
| CWSNP7981 | scaffold36 | 167556 | [C/T] | - | DRR | - |
| CWSNP7982 | scaffold36 | 226511 | [A/G] | Ca25864 | Synonymous-CDS | - |
| CWSNP7983 | scaffold362 | 84943 | [A/G] | - | Intergenic | - |
| CWSNP7984 | scaffold362 | 84945 | [T/C] | - | Intergenic | - |

| SNP IDs | Chromosomes | Physical positions (bp) | SNPs | Gene accession IDs | Sequence components of genes | Putative functions |
|---|---|---|---|---|---|---|
| CWSNP7985 | scaffold362 | 84972 | [C/A] | - | Intergenic | - |
| CWSNP7986 | scaffold362 | 84988 | [T/G] | - | Intergenic | - |
| CWSNP7987 | scaffold362 | 84994 | [G/A] | - | Intergenic | - |
| CWSNP7988 | scaffold362 | 164620 | [C/T] | - | URR | - |
| CWSNP7989 | scaffold362 | 164789 | [T/C] | - | URR | - |
| CWSNP7990 | scaffold362 | 164787 | [C/T] | - | URR | - |
| CWSNP7991 | scaffold362 | 196556 | [G/A] | Ca24993 | Non-Synonymous-CDS | RNA recognition motif domain |
| CWSNP7992 | scaffold362 | 219176 | [T/C] | - | Intergenic | - |
| CWSNP7993 | scaffold3693 | 81068 | [C/T] | - | Intergenic | - |
| CWSNP7994 | scaffold3693 | 81080 | [C/A] | - | Intergenic | - |
| CWSNP7995 | scaffold3724 | 68378 | [G/A] | Ca27523 | Intron | Pseudouridine-5'-phosphateglycosidase |
| CWSNP7996 | scaffold374 | 132907 | [A/C] | - | Intergenic | - |
| CWSNP7997 | scaffold374 | 132896 | [T/C] | - | Intergenic | - |
| CWSNP7998 | scaffold374 | 132873 | [A/G] | - | Intergenic | - |
| CWSNP7999 | scaffold374 | 132859 | [C/T] | - | Intergenic | - |
| CWSNP8000 | scaffold374 | 132858 | [G/A] | - | Intergenic | - |

| SNP IDs | Chromosomes | Physical positions (bp) | SNPs | Gene accession IDs | Sequence components of genes | Putative functions |
|---|---|---|---|---|---|---|
| CWSNP8001 | scaffold374 | 132849 | [A/C] | - | Intergenic | - |
| CWSNP8002 | scaffold374 | 132843 | [C/T] | - | Intergenic | - |
| CWSNP8003 | scaffold377 | 393535 | [G/T] | - | Intergenic | - |
| CWSNP8004 | scaffold379 | 69075 | [A/C] | Ca26154 | Non-Synonymous-CDS | PGAP1-like |
| CWSNP8005 | scaffold379 | 144442 | [G/A] | - | Intergenic | - |
| CWSNP8006 | scaffold379 | 177306 | [T/C] | - | Intergenic | - |
| CWSNP8007 | scaffold379 | 177354 | [G/A] | - | Intergenic | - |
| CWSNP8008 | scaffold379 | 177340 | [C/T] | - | Intergenic | - |
| CWSNP8009 | scaffold379 | 227608 | [A/T] | - | Intergenic | - |
| CWSNP8010 | scaffold38 | 69499 | [A/G] | - | Intergenic | - |
| CWSNP8011 | scaffold38 | 413691 | [G/A] | - | Intergenic | - |
| CWSNP8012 | scaffold38 | 413713 | [A/C] | - | Intergenic | - |
| CWSNP8013 | scaffold38 | 413719 | [G/A] | - | Intergenic | - |
| CWSNP8014 | scaffold38 | 457392 | [C/A] | Ca23625 | Synonymous-CDS | Armadillo |
| CWSNP8015 | scaffold38 | 457596 | [T/A] | Ca23625 | Intron | Armadillo |
| CWSNP8016 | scaffold38 | 520372 | [T/G] | - | Intergenic | - |

| SNP IDs | Chromosomes | Physical positions (bp) | SNPs | Gene accession IDs | Sequence components of genes | Putative functions |
|---|---|---|---|---|---|---|
| CWSNP8017 | scaffold38 | 520311 | [G/A] | - | Intergenic | - |
| CWSNP8018 | scaffold38 | 520377 | [T/C] | - | Intergenic | - |
| CWSNP8019 | scaffold38 | 537059 | [A/T] | - | Intergenic | - |
| CWSNP8020 | scaffold382 | 36686 | [A/T] | Ca27060 | Intron | - |
| CWSNP8021 | scaffold382 | 36703 | [A/T] | Ca27060 | Intron | - |
| CWSNP8022 | scaffold382 | 36704 | [G/T] | Ca27060 | Intron | - |
| CWSNP8023 | scaffold382 | 36709 | [G/A] | Ca27060 | Intron | - |
| CWSNP8024 | scaffold3865 | 65093 | [T/C] | - | Intergenic | - |
| CWSNP8025 | scaffold3865 | 65127 | [C/A] | - | Intergenic | - |
| CWSNP8026 | scaffold3865 | 65129 | [T/A] | - | Intergenic | - |
| CWSNP8027 | scaffold387 | 3263 | [G/A] | - | Intergenic | - |
| CWSNP8028 | scaffold387 | 3309 | [T/C] | - | Intergenic | - |
| CWSNP8029 | scaffold3945 | 3367 | [G/A] | Ca26968 | Intron | - |
| CWSNP8030 | scaffold3945 | 3371 | [G/A] | Ca26968 | Intron | - |
| CWSNP8031 | scaffold3945 | 3880 | [G/T] | - | Intergenic | - |
| CWSNP8032 | scaffold3945 | 3879 | [T/C] | - | Intergenic | - |

| SNP IDs | Chromosomes | Physical positions (bp) | SNPs | Gene accession IDs | Sequence components of genes | Putative functions |
|---|---|---|---|---|---|---|
| CWSNP8033 | scaffold3945 | 7260 | [T/A] | - | DRR | - |
| CWSNP8034 | scaffold3945 | 13468 | [C/T] | Ca26970 | Intron | - |
| CWSNP8035 | scaffold3945 | 13440 | [C/T] | Ca26970 | Intron | - |
| CWSNP8036 | scaffold3945 | 13892 | [C/G] | Ca26970 | Intron | - |
| CWSNP8037 | scaffold3945 | 19359 | [G/A] | Ca26970 | Intron | - |
| CWSNP8038 | scaffold3945 | 19366 | [G/T] | Ca26970 | Intron | - |
| CWSNP8039 | scaffold3945 | 19367 | [A/T] | Ca26970 | Intron | - |
| CWSNP8040 | scaffold3945 | 19368 | [A/C] | Ca26970 | Intron | - |
| CWSNP8041 | scaffold396 | 130671 | [A/G] | - | Intergenic | - |
| CWSNP8042 | scaffold396 | 130639 | [C/A] | - | Intergenic | - |
| CWSNP8043 | scaffold396 | 130636 | [G/A] | - | Intergenic | - |
| CWSNP8044 | scaffold396 | 130635 | [G/T] | - | Intergenic | - |
| CWSNP8045 | scaffold396 | 130624 | [A/G] | - | Intergenic | - |
| CWSNP8046 | scaffold396 | 130621 | [C/A] | - | Intergenic | - |
| CWSNP8047 | scaffold396 | 195890 | [A/G] | - | Intergenic | - |
| CWSNP8048 | scaffold396 | 297325 | [G/C] | Ca23551 | Intron | SecYprotein |

| SNP IDs | Chromosomes | Physical positions (bp) | SNPs | Gene accession IDs | Sequence components of genes | Putative functions |
|---|---|---|---|---|---|---|
| CWSNP8049 | scaffold398 | 441570 | [C/T] | - | Intergenic | - |
| CWSNP8050 | scaffold398 | 441605 | [C/A] | - | Intergenic | - |
| CWSNP8051 | scaffold40 | 386189 | [A/C] | - | Intergenic | - |
| CWSNP8052 | scaffold40 | 497729 | [T/C] | Ca18973 | Non-Synonymous-CDS | F-boxdomain,cyclin-like |
| CWSNP8053 | scaffold40 | 542951 | [C/G] | Ca18975 | Intron | - |
| CWSNP8054 | scaffold40 | 548453 | [A/G] | Ca18975 | Intron | - |
| CWSNP8055 | scaffold40 | 871303 | [A/C] | - | Intergenic | - |
| CWSNP8056 | scaffold40 | 911602 | [T/G] | Ca18994 | Intron | - |
| CWSNP8057 | scaffold40 | 968798 | [G/A] | Ca18995 | Synonymous-CDS | - |
| CWSNP8058 | scaffold40 | 996604 | [T/C] | Ca18996 | Synonymous-CDS | - |
| CWSNP8059 | scaffold40 | 1014875 | [G/A] | - | Intergenic | - |
| CWSNP8060 | scaffold40 | 1014834 | [T/A] | - | Intergenic | - |
| CWSNP8061 | scaffold40 | 1014826 | [C/T] | - | Intergenic | - |
| CWSNP8062 | scaffold40 | 1014805 | [C/T] | - | Intergenic | - |
| CWSNP8063 | scaffold40 | 1039218 | [T/C] | - | Intergenic | - |
| CWSNP8064 | scaffold40 | 1039233 | [A/T] | - | Intergenic | - |

| SNP IDs | Chromosomes | Physical positions (bp) | SNPs | Gene accession IDs | Sequence components of genes | Putative functions |
|---|---|---|---|---|---|---|
| CWSNP8065 | scaffold40 | 1049594 | [G/A] | Ca19000 | Intron | SANT domain, DNA binding |
| CWSNP8066 | scaffold40 | 1050164 | [C/T] | Ca19000 | Intron | SANT domain, DNA binding |
| CWSNP8067 | scaffold40 | 1050175 | [G/C] | Ca19000 | Intron | SANT domain, DNA binding |
| CWSNP8068 | scaffold40 | 1052597 | [T/C] | Ca19000 | Intron | SANT domain, DNA binding |
| CWSNP8069 | scaffold40 | 1052983 | [G/A] | Ca19000 | Intron | SANT domain, DNA binding |
| CWSNP8070 | scaffold40 | 1053215 | [G/A] | Ca19000 | Intron | SANT domain, DNA binding |
| CWSNP8071 | scaffold401 | 49065 | [G/A] | Ca25591 | Non-Synonymous-CDS | - |
| CWSNP8072 | scaffold4011 | 96223 | [G/C] | Ca26888 | Synonymous-CDS | Protein of unknown function DUF1296 |
| CWSNP8073 | scaffold404 | 273203 | [C/A] | - | Intergenic | - |
| CWSNP8074 | scaffold404 | 273209 | [C/A] | - | Intergenic | - |
| CWSNP8075 | scaffold404 | 273220 | [G/A] | - | Intergenic | - |
| CWSNP8076 | scaffold404 | 273245 | [C/T] | - | Intergenic | - |
| CWSNP8077 | scaffold404 | 285952 | [T/C] | - | Intergenic | - |
| CWSNP8078 | scaffold404 | 285958 | [C/G] | - | Intergenic | - |
| CWSNP8079 | scaffold404 | 285999 | [T/G] | - | Intergenic | - |
| CWSNP8080 | scaffold404 | 355859 | [G/A] | Ca24364 | Non-Synonymous-CDS | - |

| SNP IDs | Chromosomes | Physical positions (bp) | SNPs | Gene accession IDs | Sequence components of genes | Putative functions |
|---|---|---|---|---|---|---|
| CWSNP8081 | scaffold4057 | 16503 | [C/G] | Ca27489 | Non-Synonymous-CDS | PeptidaseA1 |
| CWSNP8082 | scaffold4102 | 1160 | [T/C] | - | Intergenic | - |
| CWSNP8083 | scaffold411 | 60078 | [C/T] | - | Intergenic | - |
| CWSNP8084 | scaffold411 | 323122 | [T/C] | - | Intergenic | - |
| CWSNP8085 | scaffold411 | 323140 | [T/C] | - | Intergenic | - |
| CWSNP8086 | scaffold411 | 323141 | [G/A] | - | Intergenic | - |
| CWSNP8087 | scaffold411 | 323147 | [T/C] | - | Intergenic | - |
| CWSNP8088 | scaffold411 | 323161 | [G/A] | - | Intergenic | - |
| CWSNP8089 | scaffold411 | 323162 | [T/C] | - | Intergenic | - |
| CWSNP8090 | scaffold411 | 323167 | [C/G] | - | Intergenic | - |
| CWSNP8091 | scaffold415 | 8479 | [A/C] | Ca28252 | Non-Synonymous-CDS | - |
| CWSNP8092 | scaffold418 | 6092 | [C/G] | Ca24670 | Intron | PeptidaseM48 |
| CWSNP8093 | scaffold418 | 13769 | [T/G] | - | Intergenic | - |
| CWSNP8094 | scaffold418 | 13811 | [G/A] | - | Intergenic | - |
| CWSNP8095 | scaffold418 | 13812 | [C/A] | - | Intergenic | - |
| CWSNP8096 | scaffold418 | 151205 | [C/T] | - | DRR | - |

| SNP IDs | Chromosomes | Physical positions (bp) | SNPs | Gene accession IDs | Sequence components of genes | Putative functions |
|---|---|---|---|---|---|---|
| CWSNP8097 | scaffold418 | 151256 | [G/A] | - | DRR | - |
| CWSNP8098 | scaffold419 | 73537 | [T/G] | - | Intergenic | - |
| CWSNP8099 | scaffold420 | 34145 | [T/G] | Ca22195 | Non-Synonymous-CDS | Zinc finger,PHD-type |
| CWSNP8100 | scaffold420 | 42532 | [G/A] | Ca22196 | Synonymous-CDS | Ubiquitin |
| CWSNP8101 | scaffold420 | 42739 | [C/T] | Ca22196 | Synonymous-CDS | Ubiquitin |
| CWSNP8102 | scaffold420 | 42811 | [C/T] | Ca22196 | Synonymous-CDS | Ubiquitin |
| CWSNP8103 | scaffold420 | 42877 | [T/C] | Ca22196 | Synonymous-CDS | Ubiquitin |
| CWSNP8104 | scaffold420 | 43372 | [C/T] | Ca22196 | Synonymous-CDS | Ubiquitin |
| CWSNP8105 | scaffold420 | 92756 | [C/T] | - | URR | - |
| CWSNP8106 | scaffold420 | 92759 | [A/G] | - | URR | - |
| CWSNP8107 | scaffold420 | 92784 | [C/T] | - | URR | - |
| CWSNP8108 | scaffold420 | 92813 | [G/T] | - | URR | - |
| CWSNP8109 | scaffold420 | 149744 | [C/G] | Ca22199 | Intron | Protein kinase, catalytic domain |
| CWSNP8110 | scaffold420 | 242503 | [C/T] | - | Intergenic | - |
| CWSNP8111 | scaffold420 | 242487 | [A/G] | - | Intergenic | - |
| CWSNP8112 | scaffold420 | 242922 | [A/C] | - | Intergenic | - |

| SNP IDs | Chromosomes | Physical positions (bp) | SNPs | Gene accession IDs | Sequence components of genes | Putative functions |
|---|---|---|---|---|---|---|
| CWSNP8113 | scaffold420 | 254661 | [T/G] | Ca22205 | Intron | Fatty acid desaturase,type1 |
| CWSNP8114 | scaffold420 | 341184 | [A/G] | Ca22212 | Intron | Glycosidehydrolase,family1 |
| CWSNP8115 | scaffold420 | 341202 | [T/G] | Ca22212 | Intron | Glycosidehydrolase,family1 |
| CWSNP8116 | scaffold420 | 341206 | [G/A] | Ca22212 | Intron | Glycosidehydrolase,family1 |
| CWSNP8117 | scaffold421_2 | 142859 | [G/C] | - | Intergenic | - |
| CWSNP8118 | scaffold421_2 | 341136 | [C/A] | - | DRR | - |
| CWSNP8119 | scaffold424 | 32402 | [C/G] | Ca27938 | Non-Synonymous-CDS | RNA-binding,CRM domain |
| CWSNP8120 | scaffold44 | 66280 | [G/A] | Ca25409 | Non-Synonymous-CDS | - |
| CWSNP8121 | scaffold44 | 87412 | [G/A] | Ca25411 | Non-Synonymous-CDS | - |
| CWSNP8122 | scaffold4452 | 20003 | [G/A] | Ca26054 | Intron | Aminotransferase-like,plant mobile domain |
| CWSNP8123 | scaffold4452 | 20005 | [G/A] | Ca26054 | Intron | Aminotransferase-like,plant mobile domain |
| CWSNP8124 | scaffold4452 | 20038 | [A/G] | Ca26054 | Intron | Aminotransferase-like,plant mobile domain |
| CWSNP8125 | scaffold4452 | 20010 | [G/A] | Ca26054 | Intron | Aminotransferase-like,plant mobile domain |
| CWSNP8126 | scaffold4452 | 29414 | [G/T] | Ca26055 | Intron | Zinc finger,PHD-type |
| CWSNP8127 | scaffold4452 | 36715 | [A/T] | Ca26055 | Intron | Zinc finger,PHD-type |
| CWSNP8128 | scaffold4452 | 70041 | [T/C] | Ca26056 | Synonymous-CDS | - |

| SNP IDs | Chromosomes | Physical positions (bp) | SNPs | Gene accession IDs | Sequence components of genes | Putative functions |
|---|---|---|---|---|---|---|
| CWSNP8129 | scaffold450 | 132427 | [A/T] | Ca23794 | Synonymous-CDS | - |
| CWSNP8130 | scaffold450 | 197287 | [G/A] | - | Intergenic | - |
| CWSNP8131 | scaffold450 | 197272 | [G/A] | - | Intergenic | - |
| CWSNP8132 | scaffold450 | 197262 | [T/G] | - | Intergenic | - |
| CWSNP8133 | scaffold450 | 197230 | [C/A] | - | Intergenic | - |
| CWSNP8134 | scaffold450 | 197226 | [G/A] | - | Intergenic | - |
| CWSNP8135 | scaffold450 | 250303 | [G/T] | - | Intergenic | - |
| CWSNP8136 | scaffold451 | 103555 | [G/C] | - | Intergenic | - |
| CWSNP8137 | scaffold451 | 103596 | [A/G] | - | Intergenic | - |
| CWSNP8138 | scaffold4511 | 40014 | [C/T] | Ca28219 | Non-Synonymous-CDS | PeptidaseC48,SUMO/Sentrin/Ubl1 |
| CWSNP8139 | scaffold4511 | 40089 | [C/T] | Ca28219 | Non-Synonymous-CDS | PeptidaseC48,SUMO/Sentrin/Ubl1 |
| CWSNP8140 | scaffold452 | 1892 | [G/A] | - | Intergenic | - |
| CWSNP8141 | scaffold452 | 1881 | [A/G] | - | Intergenic | - |
| CWSNP8142 | scaffold452 | 1841 | [A/G] | - | Intergenic | - |
| CWSNP8143 | scaffold452 | 1837 | [A/C] | - | Intergenic | - |
| CWSNP8144 | scaffold452 | 1836 | [A/G] | - | Intergenic | - |

| SNP IDs | Chromosomes | Physical positions (bp) | SNPs | Gene accession IDs | Sequence components of genes | Putative functions |
|---|---|---|---|---|---|---|
| CWSNP8145 | scaffold452 | 1835 | [C/T] | - | DRR | - |
| CWSNP8146 | scaffold453 | 303193 | [T/C] | - | Intergenic | - |
| CWSNP8147 | scaffold453 | 303201 | [G/C] | - | Intergenic | - |
| CWSNP8148 | scaffold453 | 303246 | [A/G] | - | Intergenic | - |
| CWSNP8149 | scaffold453 | 303248 | [C/T] | - | Intergenic | - |
| CWSNP8150 | scaffold453 | 303259 | [C/G] | - | Intergenic | - |
| CWSNP8151 | scaffold46 | 39438 | [C/A] | - | Intergenic | - |
| CWSNP8152 | scaffold46 | 39470 | [C/T] | - | Intergenic | - |
| CWSNP8153 | scaffold461 | 20603 | [C/T] | - | Intergenic | - |
| CWSNP8154 | scaffold4620 | 2699 | [T/C] | - | Intergenic | - |
| CWSNP8155 | scaffold4620 | 23442 | [T/C] | - | DRR | - |
| CWSNP8156 | scaffold4641 | 511 | [G/A] | - | Intergenic | - |
| CWSNP8157 | scaffold4641 | 547 | [A/C] | - | Intergenic | - |
| CWSNP8158 | scaffold4641 | 553 | [C/A] | - | Intergenic | - |
| CWSNP8159 | scaffold4641 | 556 | [G/C] | - | Intergenic | - |
| CWSNP8160 | scaffold4662 | 7683 | [T/C] | - | Intergenic | - |

| SNP IDs | Chromosomes | Physical positions (bp) | SNPs | Gene accession IDs | Sequence components of genes | Putative functions |
|---|---|---|---|---|---|---|
| CWSNP8161 | scaffold4662 | 7651 | [T/C] | - | Intergenic | - |
| CWSNP8162 | scaffold4662 | 7638 | [C/T] | - | Intergenic | - |
| CWSNP8163 | scaffold4662 | 7632 | [A/G] | - | Intergenic | - |
| CWSNP8164 | scaffold4695 | 5685 | [A/G] | - | Intergenic | - |
| CWSNP8165 | scaffold475 | 135286 | [C/A] | - | Intergenic | - |
| CWSNP8166 | scaffold475 | 135290 | [G/A] | - | Intergenic | - |
| CWSNP8167 | scaffold477 | 51898 | [C/T] | - | Intergenic | - |
| CWSNP8168 | scaffold4777 | 10546 | [G/T] | Ca26707 | Non-Synonymous-CDS | IQ motif, EF-hand binding site |
| CWSNP8169 | scaffold4777 | 16224 | [A/T] | Ca26707 | Non-Synonymous-CDS | IQ motif, EF-hand binding site |
| CWSNP8170 | scaffold4777 | 16239 | [G/T] | Ca26707 | Non-Synonymous-CDS | IQ motif, EF-hand binding site |
| CWSNP8171 | scaffold4777 | 55347 | [G/T] | Ca26708 | Non-Synonymous-CDS | PWWP |
| CWSNP8172 | scaffold4777 | 72624 | [G/A] | Ca26709 | Intron | Peptidoglycan-binding Lysin subgroup |
| CWSNP8173 | scaffold4777 | 76950 | [C/A] | - | URR | - |
| CWSNP8174 | scaffold4777 | 106820 | [A/G] | Ca26711 | Intron | WD40 repeat |
| CWSNP8175 | scaffold4777 | 112193 | [A/C] | Ca26711 | Intron | WD40 repeat |
| CWSNP8176 | scaffold48 | 261719 | [A/G] | - | Intergenic | - |

| SNP IDs | Chromosomes | Physical positions (bp) | SNPs | Gene accession IDs | Sequence components of genes | Putative functions |
|---|---|---|---|---|---|---|
| CWSNP8177 | scaffold480 | 67375 | [T/C] | Ca26870 | Intron | PeptidaseM24,structural domain |
| CWSNP8178 | scaffold4836 | 5866 | [T/A] | Ca28200 | Non-Synonymous-CDS | - |
| CWSNP8179 | scaffold4836 | 5891 | [A/T] | Ca28200 | Synonymous-CDS | - |
| CWSNP8180 | scaffold484 | 209786 | [A/C] | - | DRR | - |
| CWSNP8181 | scaffold484 | 647868 | [C/T] | - | Intergenic | - |
| CWSNP8182 | scaffold484 | 647866 | [A/G] | - | Intergenic | - |
| CWSNP8183 | scaffold484 | 647809 | [A/T] | - | Intergenic | - |
| CWSNP8184 | scaffold484 | 660749 | [C/G] | - | Intergenic | - |
| CWSNP8185 | scaffold485 | 123578 | [T/C] | - | Intergenic | - |
| CWSNP8186 | scaffold495 | 128200 | [A/T] | Ca26761 | Intron | Pathogenesis-related transcriptional factor/ERF, DNA-binding |
| CWSNP8187 | scaffold496 | 53652 | [G/A] | - | Intergenic | - |
| CWSNP8188 | scaffold496 | 53657 | [A/T] | - | Intergenic | - |
| CWSNP8189 | scaffold496 | 175493 | [G/A] | Ca27202 | Synonymous-CDS | PeptidaseC48,SUMO/Sentrin/Ubl1 |
| CWSNP8190 | scaffold50 | 191625 | [T/C] | Ca24567 | Non-Synonymous-CDS | - |
| CWSNP8191 | scaffold50 | 201219 | [C/G] | - | Intergenic | - |
| CWSNP8192 | scaffold50 | 448866 | [G/A] | Ca24573 | Intron | - |

| SNP IDs | Chromosomes | Physical positions (bp) | SNPs | Gene accession IDs | Sequence components of genes | Putative functions |
|---|---|---|---|---|---|---|
| CWSNP8193 | scaffold510 | 12383 | [G/T] | - | Intergenic | - |
| CWSNP8194 | scaffold510 | 12394 | [C/A] | - | Intergenic | - |
| CWSNP8195 | scaffold510 | 12414 | [C/T] | - | Intergenic | - |
| CWSNP8196 | scaffold511 | 10450 | [T/G] | Ca26652 | Synonymous-CDS | - |
| CWSNP8197 | scaffold511 | 10520 | [G/T] | Ca26652 | Intron | - |
| CWSNP8198 | scaffold513 | 7188 | [A/C] | - | Intergenic | - |
| CWSNP8199 | scaffold5163 | 2301 | [A/G] | - | Intergenic | - |
| CWSNP8200 | scaffold5185 | 1091 | [G/A] | Ca28010 | Non-Synonymous-CDS | - |
| CWSNP8201 | scaffold520 | 143359 | [C/T] | - | Intergenic | - |
| CWSNP8202 | scaffold520 | 143358 | [G/A] | - | Intergenic | - |
| CWSNP8203 | scaffold520 | 143336 | [T/C] | - | Intergenic | - |
| CWSNP8204 | scaffold520 | 143334 | [G/A] | - | Intergenic | - |
| CWSNP8205 | scaffold520 | 143333 | [A/T] | - | Intergenic | - |
| CWSNP8206 | scaffold520 | 143324 | [G/A] | - | Intergenic | - |
| CWSNP8207 | scaffold520 | 143317 | [T/A] | - | Intergenic | - |
| CWSNP8208 | scaffold520 | 143300 | [G/A] | - | Intergenic | - |

| SNP IDs | Chromosomes | Physical positions (bp) | SNPs | Gene accession IDs | Sequence components of genes | Putative functions |
|---|---|---|---|---|---|---|
| CWSNP8209 | scaffold520 | 143294 | [G/A] | - | Intergenic | - |
| CWSNP8210 | scaffold520 | 143291 | [G/A] | - | Intergenic | - |
| CWSNP8211 | scaffold520 | 143372 | [G/A] | - | Intergenic | - |
| CWSNP8212 | scaffold520 | 143355 | [C/T] | - | Intergenic | - |
| CWSNP8213 | scaffold520 | 143391 | [A/C] | - | Intergenic | - |
| CWSNP8214 | scaffold53 | 49273 | [A/G] | - | URR | - |
| CWSNP8215 | scaffold531 | 2668 | [C/T] | - | Intergenic | - |
| CWSNP8216 | scaffold531 | 65242 | [T/G] | - | Intergenic | - |
| CWSNP8217 | scaffold531 | 101520 | [C/G] | - | DRR | - |
| CWSNP8218 | scaffold531 | 124019 | [T/G] | - | DRR | - |
| CWSNP8219 | scaffold5328 | 85 | [C/T] | - | Intergenic | - |
| CWSNP8220 | scaffold5328 | 117 | [C/T] | - | Intergenic | - |
| CWSNP8221 | scaffold535 | 156836 | [G/A] | - | Intergenic | - |
| CWSNP8222 | scaffold5358 | 2561 | [C/A] | - | Intergenic | - |
| CWSNP8223 | scaffold5358 | 3329 | [C/A] | - | Intergenic | - |
| CWSNP8224 | scaffold537 | 216656 | [A/G] | - | Intergenic | - |

| SNP IDs | Chromosomes | Physical positions (bp) | SNPs | Gene accession IDs | Sequence components of genes | Putative functions |
|---|---|---|---|---|---|---|
| CWSNP8225 | scaffold543 | 20780 | [T/G] | - | Intergenic | - |
| CWSNP8226 | scaffold543 | 20819 | [T/C] | - | Intergenic | - |
| CWSNP8227 | scaffold543 | 20827 | [G/T] | - | Intergenic | - |
| CWSNP8228 | scaffold543 | 143169 | [C/T] | - | DRR | - |
| CWSNP8229 | scaffold543 | 143191 | [G/A] | - | DRR | - |
| CWSNP8230 | scaffold543 | 266748 | [A/C] | - | Intergenic | - |
| CWSNP8231 | scaffold543 | 266729 | [A/C] | - | Intergenic | - |
| CWSNP8232 | scaffold543 | 266848 | [A/C] | - | Intergenic | - |
| CWSNP8233 | scaffold545 | 17699 | [A/C] | - | Intergenic | - |
| CWSNP8234 | scaffold545 | 139118 | [A/T] | Ca23923 | Synonymous-CDS | Zinc finger,C2H2-type |
| CWSNP8235 | scaffold545 | 147549 | [G/A] | Ca23924 | Non-Synonymous-CDS | Uncharacterised protein familyUPF0503 |
| CWSNP8236 | scaffold548 | 17462 | [G/A] | Ca21478 | Non-Synonymous-CDS | PeptidaseC48,SUMO/Sentrin/Ubl1 |
| CWSNP8237 | scaffold548 | 231396 | [G/A] | - | DRR | - |
| CWSNP8238 | scaffold5511 | 353 | [G/A] | - | Intergenic | - |
| CWSNP8239 | scaffold5511 | 366 | [G/A] | - | Intergenic | - |
| CWSNP8240 | scaffold5511 | 365 | [T/G] | - | Intergenic | - |

| SNP IDs | Chromosomes | Physical positions (bp) | SNPs | Gene accession IDs | Sequence components of genes | Putative functions |
|---|---|---|---|---|---|---|
| CWSNP8241 | scaffold5511 | 396 | [G/A] | - | Intergenic | - |
| CWSNP8242 | scaffold553 | 310759 | [T/A] | Ca19934 | Intron | - |
| CWSNP8243 | scaffold553 | 441898 | [G/A] | Ca19938 | Intron | Protein kinase, catalytic domain |
| CWSNP8244 | scaffold553 | 794439 | [G/T] | Ca19955 | Non-Synonymous-CDS | PeptidaseS8/S53,subtilisin/kexin/sedolisin |
| CWSNP8245 | scaffold562 | 221686 | [C/A] | Ca23135 | Non-Synonymous-CDS | PeptidaseC48,SUMO/Sentrin/Ubl1 |
| CWSNP8246 | scaffold562 | 221714 | [C/T] | Ca23135 | Non-Synonymous-CDS | PeptidaseC48,SUMO/Sentrin/Ubl1 |
| CWSNP8247 | scaffold562 | 221735 | [G/A] | Ca23135 | Non-Synonymous-CDS | PeptidaseC48,SUMO/Sentrin/Ubl1 |
| CWSNP8248 | scaffold562 | 221748 | [C/A] | Ca23135 | Synonymous-CDS | PeptidaseC48,SUMO/Sentrin/Ubl1 |
| CWSNP8249 | scaffold562 | 221758 | [G/A] | Ca23135 | Non-Synonymous-CDS | PeptidaseC48,SUMO/Sentrin/Ubl1 |
| CWSNP8250 | scaffold562 | 221769 | [T/A] | Ca23135 | Non-Synonymous-CDS | PeptidaseC48,SUMO/Sentrin/Ubl1 |
| CWSNP8251 | scaffold562 | 221778 | [G/A] | Ca23135 | Synonymous-CDS | PeptidaseC48,SUMO/Sentrin/Ubl1 |
| CWSNP8252 | scaffold562 | 221779 | [G/A] | Ca23135 | Non-Synonymous-CDS | PeptidaseC48,SUMO/Sentrin/Ubl1 |
| CWSNP8253 | scaffold562 | 221788 | [G/A] | Ca23135 | Non-Synonymous-CDS | PeptidaseC48,SUMO/Sentrin/Ubl1 |
| CWSNP8254 | scaffold562 | 221828 | [T/A] | Ca23135 | Non-Synonymous-CDS | PeptidaseC48,SUMO/Sentrin/Ubl1 |
| CWSNP8255 | scaffold562 | 221792 | [C/A] | Ca23135 | Non-Synonymous-CDS | PeptidaseC48,SUMO/Sentrin/Ubl1 |
| CWSNP8256 | scaffold562 | 255295 | [G/A] | Ca23136 | Intron | - |

| SNP IDs | Chromosomes | Physical positions (bp) | SNPs | Gene accession IDs | Sequence components of genes | Putative functions |
|---|---|---|---|---|---|---|
| CWSNP8257 | scaffold562 | 289080 | [C/A] | - | URR | - |
| CWSNP8258 | scaffold562 | 290071 | [C/T] | Ca23141 | Intron | Protein of unknown function DUF642 |
| CWSNP8259 | scaffold5714 | 24882 | [A/C] | - | Intergenic | - |
| CWSNP8260 | scaffold5714 | 36359 | [G/C] | - | DRR | - |
| CWSNP8261 | scaffold5730 | 192 | [A/G] | - | Intergenic | - |
| CWSNP8262 | scaffold575 | 36238 | [C/T] | Ca27000 | Intron | - |
| CWSNP8263 | scaffold575 | 45254 | [C/T] | - | URR | - |
| CWSNP8264 | scaffold575 | 45250 | [T/C] | - | Intergenic | - |
| CWSNP8265 | scaffold575 | 45236 | [G/A] | - | Intergenic | - |
| CWSNP8266 | scaffold575 | 45215 | [C/A] | - | Intergenic | - |
| CWSNP8267 | scaffold575 | 45192 | [C/T] | - | Intergenic | - |
| CWSNP8268 | scaffold575 | 45279 | [G/C] | - | Intergenic | - |
| CWSNP8269 | scaffold575 | 45245 | [G/A] | - | URR | - |
| CWSNP8270 | scaffold575 | 45237 | [C/T] | - | Intergenic | - |
| CWSNP8271 | scaffold575 | 45234 | [C/T] | - | Intergenic | - |
| CWSNP8272 | scaffold575 | 110772 | [G/T] | - | Intergenic | - |

| SNP IDs | Chromosomes | Physical positions (bp) | SNPs | Gene accession IDs | Sequence components of genes | Putative functions |
|---|---|---|---|---|---|---|
| CWSNP8273 | scaffold575 | 110785 | [C/T] | - | Intergenic | - |
| CWSNP8274 | scaffold575 | 110765 | [C/T] | - | Intergenic | - |
| CWSNP8275 | scaffold575 | 110759 | [C/T] | - | Intergenic | - |
| CWSNP8276 | scaffold575 | 110753 | [G/A] | - | Intergenic | - |
| CWSNP8277 | scaffold575 | 117287 | [T/G] | - | Intergenic | - |
| CWSNP8278 | scaffold5792 | 65166 | [A/C] | Ca26785 | Non-Synonymous-CDS | Paired amphipathic helix |
| CWSNP8279 | scaffold5792 | 86716 | [T/C] | - | Intergenic | - |
| CWSNP8280 | scaffold584 | 53203 | [G/T] | Ca25252 | Intron | SAGA-associated factor29 |
| CWSNP8281 | scaffold590 | 88429 | [A/T] | Ca25557 | Intron | WD40 repeat |
| CWSNP8282 | scaffold590 | 88420 | [T/C] | Ca25557 | Intron | WD40 repeat |
| CWSNP8283 | scaffold598 | 300825 | [A/C] | Ca21757 | Non-Synonymous-CDS | F-boxdomain,cyclin-like |
| CWSNP8284 | scaffold598 | 300940 | [C/T] | Ca21757 | Synonymous-CDS | F-boxdomain,cyclin-like |
| CWSNP8285 | scaffold598 | 404254 | [G/C] | - | Intergenic | - |
| CWSNP8286 | scaffold598 | 404275 | [G/T] | - | Intergenic | - |
| CWSNP8287 | scaffold599 | 349605 | [T/G] | - | Intergenic | - |
| CWSNP8288 | scaffold5997 | 742 | [G/C] | - | Intergenic | - |

| SNP IDs | Chromosomes | Physical positions (bp) | SNPs | Gene accession IDs | Sequence components of genes | Putative functions |
|---|---|---|---|---|---|---|
| CWSNP8289 | scaffold5997 | 679 | [C/T] | - | Intergenic | - |
| CWSNP8290 | scaffold601 | 71998 | [T/A] | - | Intergenic | - |
| CWSNP8291 | scaffold605 | 23769 | [A/C] | - | Intergenic | - |
| CWSNP8292 | scaffold605 | 23773 | [T/C] | - | Intergenic | - |
| CWSNP8293 | scaffold605 | 23774 | [A/G] | - | Intergenic | - |
| CWSNP8294 | scaffold605 | 23775 | [A/G] | - | Intergenic | - |
| CWSNP8295 | scaffold605 | 23793 | [A/G] | - | Intergenic | - |
| CWSNP8296 | scaffold605 | 23795 | [T/A] | - | Intergenic | - |
| CWSNP8297 | scaffold605 | 23803 | [A/G] | - | Intergenic | - |
| CWSNP8298 | scaffold605 | 23808 | [C/G] | - | Intergenic | - |
| CWSNP8299 | scaffold605 | 23811 | [G/A] | - | Intergenic | - |
| CWSNP8300 | scaffold605 | 23816 | [T/A] | - | Intergenic | - |
| CWSNP8301 | scaffold605 | 84761 | [A/C] | - | Intergenic | - |
| CWSNP8302 | scaffold605 | 84765 | [T/C] | - | Intergenic | - |
| CWSNP8303 | scaffold605 | 84766 | [A/G] | - | Intergenic | - |
| CWSNP8304 | scaffold605 | 84767 | [A/G] | - | Intergenic | - |

| SNP IDs | Chromosomes | Physical positions (bp) | SNPs | Gene accession IDs | Sequence components of genes | Putative functions |
|---|---|---|---|---|---|---|
| CWSNP8305 | scaffold605 | 84785 | [A/G] | - | Intergenic | - |
| CWSNP8306 | scaffold605 | 84787 | [T/A] | - | Intergenic | - |
| CWSNP8307 | scaffold605 | 84795 | [A/G] | - | Intergenic | - |
| CWSNP8308 | scaffold605 | 84800 | [C/G] | - | Intergenic | - |
| CWSNP8309 | scaffold605 | 84803 | [G/A] | - | Intergenic | - |
| CWSNP8310 | scaffold605 | 84807 | [A/G] | - | Intergenic | - |
| CWSNP8311 | scaffold62 | 46088 | [C/T] | Ca26577 | Non-Synonymous-CDS | PeptidaseC48,SUMO/Sentrin/Ubl1 |
| CWSNP8312 | scaffold624 | 254901 | [C/T] | - | Intergenic | - |
| CWSNP8313 | scaffold624 | 254950 | [G/A] | - | Intergenic | - |
| CWSNP8314 | scaffold624 | 254959 | [T/C] | - | Intergenic | - |
| CWSNP8315 | scaffold624 | 254971 | [G/A] | - | Intergenic | - |
| CWSNP8316 | scaffold624 | 254992 | [C/T] | - | Intergenic | - |
| CWSNP8317 | scaffold624 | 254942 | [A/T] | - | Intergenic | - |
| CWSNP8318 | scaffold6339 | 190 | [T/G] | - | Intergenic | - |
| CWSNP8319 | scaffold6367 | 6825 | [G/T] | Ca27012 | Synonymous-CDS | BP28,C-terminal |
| CWSNP8320 | scaffold6367 | 10856 | [T/G] | Ca27012 | Intron | BP28,C-terminal |

| SNP IDs | Chromosomes | Physical positions (bp) | SNPs | Gene accession IDs | Sequence components of genes | Putative functions |
|---|---|---|---|---|---|---|
| CWSNP8321 | scaffold6367 | 10842 | [T/A] | Ca27012 | Intron | BP28,C-terminal |
| CWSNP8322 | scaffold6367 | 13651 | [G/A] | Ca27012 | Intron | BP28,C-terminal |
| CWSNP8323 | scaffold6367 | 25674 | [T/C] | Ca27013 | Synonymous-CDS | Metallo-dependent phosphatase |
| CWSNP8324 | scaffold637 | 107661 | [A/C] | - | Intergenic | - |
| CWSNP8325 | scaffold637 | 107636 | [T/C] | - | Intergenic | - |
| CWSNP8326 | scaffold637 | 107613 | [C/G] | - | Intergenic | - |
| CWSNP8327 | scaffold6403 | 1553 | [T/A] | Ca27958 | Synonymous-CDS | - |
| CWSNP8328 | scaffold645 | 91908 | [C/T] | - | Intergenic | - |
| CWSNP8329 | scaffold653 | 145231 | [A/G] | - | DRR | - |
| CWSNP8330 | scaffold653 | 183054 | [T/C] | - | URR | - |
| CWSNP8331 | scaffold653 | 183063 | [G/A] | - | URR | - |
| CWSNP8332 | scaffold653 | 183069 | [C/A] | - | URR | - |
| CWSNP8333 | scaffold653 | 183074 | [C/G] | - | URR | - |
| CWSNP8334 | scaffold653 | 183078 | [T/G] | - | URR | - |
| CWSNP8335 | scaffold653 | 183091 | [T/C] | - | URR | - |
| CWSNP8336 | scaffold653 | 183143 | [T/A] | - | URR | - |

| SNP IDs | Chromosomes | Physical positions (bp) | SNPs | Gene accession IDs | Sequence components of genes | Putative functions |
|---|---|---|---|---|---|---|
| CWSNP8337 | scaffold653 | 183135 | [T/C] | - | URR | - |
| CWSNP8338 | scaffold653 | 183112 | [T/C] | - | URR | - |
| CWSNP8339 | scaffold653 | 189129 | [C/T] | Ca24313 | Synonymous-CDS | Aminotransferase,classI/classII |
| CWSNP8340 | scaffold653 | 197878 | [A/T] | Ca24313 | Non-Synonymous-CDS | Aminotransferase,classI/classII |
| CWSNP8341 | scaffold661 | 180364 | [G/T] | Ca23946 | Non-Synonymous-CDS | PeptidaseC48,SUMO/Sentrin/Ubl1 |
| CWSNP8342 | scaffold661 | 180362 | [A/C] | Ca23946 | Non-Synonymous-CDS | PeptidaseC48,SUMO/Sentrin/Ubl1 |
| CWSNP8343 | scaffold661 | 180348 | [C/T] | Ca23946 | Non-Synonymous-CDS | PeptidaseC48,SUMO/Sentrin/Ubl1 |
| CWSNP8344 | scaffold661 | 180302 | [G/T] | Ca23946 | Non-Synonymous-CDS | PeptidaseC48,SUMO/Sentrin/Ubl1 |
| CWSNP8345 | scaffold661 | 311222 | [G/T] | - | Intergenic | - |
| CWSNP8346 | scaffold661 | 311182 | [C/T] | - | Intergenic | - |
| CWSNP8347 | scaffold663 | 16068 | [G/A] | - | Intergenic | - |
| CWSNP8348 | scaffold663 | 16042 | [C/A] | - | Intergenic | - |
| CWSNP8349 | scaffold663 | 16029 | [G/A] | - | Intergenic | - |
| CWSNP8350 | scaffold663 | 16056 | [C/T] | - | Intergenic | - |
| CWSNP8351 | scaffold663 | 16061 | [A/G] | - | Intergenic | - |
| CWSNP8352 | scaffold663 | 156613 | [G/C] | - | DRR | - |

| SNP IDs | Chromosomes | Physical positions (bp) | SNPs | Gene accession IDs | Sequence components of genes | Putative functions |
|---|---|---|---|---|---|---|
| CWSNP8353 | scaffold674 | 91979 | [T/A] | - | Intergenic | - |
| CWSNP8354 | scaffold674 | 360388 | [C/T] | - | Intergenic | - |
| CWSNP8355 | scaffold674 | 360421 | [G/A] | - | Intergenic | - |
| CWSNP8356 | scaffold674 | 360406 | [C/T] | - | Intergenic | - |
| CWSNP8357 | scaffold674 | 360349 | [G/A] | - | Intergenic | - |
| CWSNP8358 | scaffold674 | 360411 | [G/A] | - | Intergenic | - |
| CWSNP8359 | scaffold674 | 360408 | [G/T] | - | Intergenic | - |
| CWSNP8360 | scaffold674 | 608623 | [T/G] | Ca20172 | Intron | Basic-leucine zipper (bZIP) Transcription factor |
| CWSNP8361 | scaffold674 | 652382 | [G/A] | - | Intergenic | - |
| CWSNP8362 | scaffold674 | 826136 | [T/C] | - | Intergenic | - |
| CWSNP8363 | scaffold682 | 157238 | [G/C] | Ca23185 | Non-Synonymous-CDS | Transcriptional factor B3 |
| CWSNP8364 | scaffold682 | 578200 | [T/G] | Ca23199 | Synonymous-CDS | AMP-dependent synthetase/ligase |
| CWSNP8365 | scaffold684 | 78894 | [G/A] | Ca26805 | Non-Synonymous-CDS | - |
| CWSNP8366 | scaffold684 | 78883 | [T/G] | Ca26805 | Non-Synonymous-CDS | - |
| CWSNP8367 | scaffold684 | 78871 | [C/G] | Ca26805 | Non-Synonymous-CDS | - |
| CWSNP8368 | scaffold684 | 78842 | [G/A] | Ca26805 | Synonymous-CDS | - |

| SNP IDs | Chromosomes | Physical positions (bp) | SNPs | Gene accession IDs | Sequence components of genes | Putative functions |
|---|---|---|---|---|---|---|
| CWSNP8369 | scaffold684 | 78844 | [A/G] | Ca26805 | Non-Synonymous-CDS | - |
| CWSNP8370 | scaffold684 | 78864 | [C/T] | Ca26805 | Non-Synonymous-CDS | - |
| CWSNP8371 | scaffold684 | 78893 | [C/T] | Ca26805 | Synonymous-CDS | - |
| CWSNP8372 | scaffold702 | 247644 | [G/C] | - | Intergenic | - |
| CWSNP8373 | scaffold711 | 153694 | [C/T] | Ca24295 | Non-Synonymous-CDS | - |
| CWSNP8374 | scaffold711 | 153680 | [C/A] | Ca24295 | Non-Synonymous-CDS | - |
| CWSNP8375 | scaffold711 | 153667 | [G/A] | Ca24295 | Non-Synonymous-CDS | - |
| CWSNP8376 | scaffold711 | 153697 | [G/A] | Ca24295 | Non-Synonymous-CDS | - |
| CWSNP8377 | scaffold711 | 153699 | [A/G] | Ca24295 | Synonymous-CDS | - |
| CWSNP8378 | scaffold711 | 153706 | [G/A] | Ca24295 | Non-Synonymous-CDS | - |
| CWSNP8379 | scaffold716 | 212326 | [G/A] | - | DRR | - |
| CWSNP8380 | scaffold716 | 269230 | [A/C] | - | Intergenic | - |
| CWSNP8381 | scaffold716 | 269242 | [A/G] | - | Intergenic | - |
| CWSNP8382 | scaffold716 | 269266 | [G/T] | - | Intergenic | - |
| CWSNP8383 | scaffold716 | 269281 | [T/G] | - | Intergenic | - |
| CWSNP8384 | scaffold716 | 269335 | [G/A] | - | Intergenic | - |

| SNP IDs | Chromosomes | Physical positions (bp) | SNPs | Gene accession IDs | Sequence components of genes | Putative functions |
|---|---|---|---|---|---|---|
| CWSNP8385 | scaffold716 | 269332 | [C/T] | - | Intergenic | - |
| CWSNP8386 | scaffold716 | 269314 | [G/A] | - | Intergenic | - |
| CWSNP8387 | scaffold716 | 269299 | [T/C] | - | Intergenic | - |
| CWSNP8388 | scaffold719 | 11850 | [C/T] | - | Intergenic | - |
| CWSNP8389 | scaffold720 | 60880 | [G/A] | - | URR | - |
| CWSNP8390 | scaffold720 | 370639 | [T/A] | - | Intergenic | - |
| CWSNP8391 | scaffold720 | 370725 | [G/A] | - | Intergenic | - |
| CWSNP8392 | scaffold724 | 25019 | [G/A] | - | Intergenic | - |
| CWSNP8393 | scaffold724 | 25722 | [G/C] | - | DRR | - |
| CWSNP8394 | scaffold731 | 315093 | [G/A] | - | Intergenic | - |
| CWSNP8395 | scaffold731 | 315114 | [C/T] | - | Intergenic | - |
| CWSNP8396 | scaffold731 | 315135 | [G/T] | - | Intergenic | - |
| CWSNP8397 | scaffold731 | 315138 | [C/T] | - | Intergenic | - |
| CWSNP8398 | scaffold731 | 315141 | [G/A] | - | Intergenic | - |
| CWSNP8399 | scaffold731 | 332497 | [G/A] | Ca23816 | Synonymous-CDS | Ribosomal proteinS7 |
| CWSNP8400 | scaffold731 | 332467 | [G/C] | Ca23816 | Synonymous-CDS | Ribosomal proteinS7 |

| SNP IDs | Chromosomes | Physical positions (bp) | SNPs | Gene accession IDs | Sequence components of genes | Putative functions |
|---|---|---|---|---|---|---|
| CWSNP8401 | scaffold731 | 332465 | [T/A] | Ca23816 | Non-Synonymous-CDS | Ribosomal proteinS7 |
| CWSNP8402 | scaffold731 | 332464 | [G/T] | Ca23816 | Synonymous-CDS | Ribosomal proteinS7 |
| CWSNP8403 | scaffold731 | 332458 | [A/T] | Ca23816 | Non-Synonymous-CDS | Ribosomal proteinS7 |
| CWSNP8404 | scaffold731 | 357674 | [G/A] | - | Intergenic | - |
| CWSNP8405 | scaffold731 | 357645 | [G/T] | - | Intergenic | - |
| CWSNP8406 | scaffold731 | 357644 | [G/C] | - | Intergenic | - |
| CWSNP8407 | scaffold731 | 357642 | [T/A] | - | Intergenic | - |
| CWSNP8408 | scaffold731 | 357641 | [G/T] | - | Intergenic | - |
| CWSNP8409 | scaffold731 | 357635 | [A/T] | - | Intergenic | - |
| CWSNP8410 | scaffold731 | 362123 | [G/A] | - | URR | - |
| CWSNP8411 | scaffold731 | 362118 | [C/A] | - | URR | - |
| CWSNP8412 | scaffold731 | 362114 | [A/C] | - | URR | - |
| CWSNP8413 | scaffold731 | 362553 | [C/T] | - | URR | - |
| CWSNP8414 | scaffold731 | 362554 | [T/A] | - | URR | - |
| CWSNP8415 | scaffold731 | 362555 | [T/G] | - | URR | - |
| CWSNP8416 | scaffold731 | 362556 | [C/A] | - | URR | - |

| SNP IDs | Chromosomes | Physical positions (bp) | SNPs | Gene accession IDs | Sequence components of genes | Putative functions |
|---|---|---|---|---|---|---|
| CWSNP8417 | scaffold731 | 362574 | [T/C] | - | URR | - |
| CWSNP8418 | scaffold731 | 362581 | [A/G] | - | URR | - |
| CWSNP8419 | scaffold731 | 362582 | [T/C] | - | URR | - |
| CWSNP8420 | scaffold731 | 362594 | [G/T] | - | URR | - |
| CWSNP8421 | scaffold731 | 362610 | [C/T] | - | URR | - |
| CWSNP8422 | scaffold731 | 372255 | [G/A] | - | URR | - |
| CWSNP8423 | scaffold731 | 372253 | [G/T] | - | URR | - |
| CWSNP8424 | scaffold731 | 372250 | [G/A] | - | URR | - |
| CWSNP8425 | scaffold731 | 372244 | [C/T] | - | URR | - |
| CWSNP8426 | scaffold731 | 372243 | [A/T] | - | URR | - |
| CWSNP8427 | scaffold731 | 372215 | [A/G] | - | URR | - |
| CWSNP8428 | scaffold731 | 372209 | [G/C] | - | URR | - |
| CWSNP8429 | scaffold731 | 372183 | [C/T] | - | URR | - |
| CWSNP8430 | scaffold731 | 372959 | [A/C] | - | URR | - |
| CWSNP8431 | scaffold7355 | 3608 | [A/G] | Ca28031 | Intron | CCT domain |
| CWSNP8432 | scaffold7355 | 3602 | [T/C] | Ca28031 | Intron | CCT domain |

| SNP IDs | Chromosomes | Physical positions (bp) | SNPs | Gene accession IDs | Sequence components of genes | Putative functions |
|---|---|---|---|---|---|---|
| CWSNP8433 | scaffold7355 | 3572 | [A/G] | Ca28031 | Intron | CCT domain |
| CWSNP8434 | scaffold752 | 382375 | [G/A] | Ca25235 | Intron | Dual specificity phosphatase,catalytic domain |
| CWSNP8435 | scaffold752 | 497452 | [T/A] | - | DRR | - |
| CWSNP8436 | scaffold752 | 497454 | [A/G] | - | DRR | - |
| CWSNP8437 | scaffold758 | 16480 | [G/T] | - | Intergenic | - |
| CWSNP8438 | scaffold7715 | 3434 | [A/T] | Ca27664 | Synonymous-CDS | - |
| CWSNP8439 | scaffold772 | 53192 | [C/T] | - | Intergenic | - |
| CWSNP8440 | scaffold772 | 53196 | [C/T] | - | Intergenic | - |
| CWSNP8441 | scaffold775 | 89238 | [T/C] | - | Intergenic | - |
| CWSNP8442 | scaffold775 | 89239 | [C/T] | - | Intergenic | - |
| CWSNP8443 | scaffold775 | 356107 | [C/A] | - | Intergenic | - |
| CWSNP8444 | scaffold775 | 448604 | [G/A] | - | DRR | - |
| CWSNP8445 | scaffold775 | 448743 | [T/C] | - | DRR | - |
| CWSNP8446 | scaffold776 | 188888 | [G/A] | - | Intergenic | - |
| CWSNP8447 | scaffold7765 | 503 | [C/T] | - | Intergenic | - |
| CWSNP8448 | scaffold7765 | 549 | [G/A] | - | Intergenic | - |

| SNP IDs | Chromosomes | Physical positions (bp) | SNPs | Gene accession IDs | Sequence components of genes | Putative functions |
|---|---|---|---|---|---|---|
| CWSNP8449 | scaffold783 | 201617 | [T/C] | - | Intergenic | - |
| CWSNP8450 | scaffold783 | 244209 | [T/C] | - | Intergenic | - |
| CWSNP8451 | scaffold7842 | 727 | [C/T] | - | Intergenic | - |
| CWSNP8452 | scaffold7842 | 1078 | [G/A] | - | Intergenic | - |
| CWSNP8453 | scaffold7842 | 1075 | [C/T] | - | Intergenic | - |
| CWSNP8454 | scaffold7842 | 1042 | [T/C] | - | Intergenic | - |
| CWSNP8455 | scaffold7842 | 1024 | [T/G] | - | Intergenic | - |
| CWSNP8456 | scaffold787 | 4363 | [A/G] | - | Intergenic | - |
| CWSNP8457 | scaffold794 | 50239 | [G/A] | - | Intergenic | - |
| CWSNP8458 | scaffold794 | 50240 | [C/A] | - | Intergenic | - |
| CWSNP8459 | scaffold799 | 3930 | [A/G] | - | Intergenic | - |
| CWSNP8460 | scaffold801 | 67115 | [C/T] | Ca26176 | Intron | PeptidaseS10,serine carboxypeptidase |
| CWSNP8461 | scaffold806 | 129263 | [A/G] | - | Intergenic | - |
| CWSNP8462 | scaffold806 | 129265 | [G/T] | - | Intergenic | - |
| CWSNP8463 | scaffold809 | 48678 | [T/C] | - | DRR | - |
| CWSNP8464 | scaffold809 | 48650 | [C/A] | - | DRR | - |

| SNP IDs | Chromosomes | Physical positions (bp) | SNPs | Gene accession IDs | Sequence components of genes | Putative functions |
|---|---|---|---|---|---|---|
| CWSNP8465 | scaffold809 | 53431 | [G/A] | - | Intergenic | - |
| CWSNP8466 | scaffold809 | 53444 | [G/A] | - | Intergenic | - |
| CWSNP8467 | scaffold809 | 53474 | [G/A] | - | Intergenic | - |
| CWSNP8468 | scaffold809 | 53464 | [G/A] | - | Intergenic | - |
| CWSNP8469 | scaffold809 | 53470 | [C/G] | - | Intergenic | - |
| CWSNP8470 | scaffold811 | 81373 | [C/T] | - | Intergenic | - |
| CWSNP8471 | scaffold812 | 34686 | [C/T] | - | Intergenic | - |
| CWSNP8472 | scaffold812 | 246208 | [A/C] | - | Intergenic | - |
| CWSNP8473 | scaffold812 | 528815 | [C/G] | - | Intergenic | - |
| CWSNP8474 | scaffold8226 | 21624 | [G/A] | - | Intergenic | - |
| CWSNP8475 | scaffold8226 | 21592 | [T/G] | - | Intergenic | - |
| CWSNP8476 | scaffold8226 | 21590 | [T/C] | - | Intergenic | - |
| CWSNP8477 | scaffold8226 | 21572 | [T/A] | - | Intergenic | - |
| CWSNP8478 | scaffold842 | 13898 | [T/C] | - | Intergenic | - |
| CWSNP8479 | scaffold842 | 13928 | [G/T] | - | Intergenic | - |
| CWSNP8480 | scaffold842 | 13946 | [T/C] | - | Intergenic | - |

| SNP IDs | Chromosomes | Physical positions (bp) | SNPs | Gene accession IDs | Sequence components of genes | Putative functions |
|---|---|---|---|---|---|---|
| CWSNP8481 | scaffold845 | 28172 | [G/T] | - | Intergenic | - |
| CWSNP8482 | scaffold845 | 45557 | [A/G] | Ca22411 | Synonymous-CDS | Cation efflux protein |
| CWSNP8483 | scaffold845 | 71041 | [A/G] | - | DRR | - |
| CWSNP8484 | scaffold845 | 129326 | [C/G] | Ca22416 | Synonymous-CDS | Dehydrogenase,E1component |
| CWSNP8485 | scaffold845 | 129370 | [A/G] | Ca22416 | Synonymous-CDS | Dehydrogenase,E1component |
| CWSNP8486 | scaffold845 | 130569 | [G/C] | Ca22416 | Synonymous-CDS | Dehydrogenase,E1component |
| CWSNP8487 | scaffold845 | 131911 | [A/G] | Ca22416 | Intron | Dehydrogenase,E1component |
| CWSNP8488 | scaffold845 | 136796 | [A/G] | Ca22417 | Intron | Zinc finger,RING-type |
| CWSNP8489 | scaffold845 | 139268 | [G/A] | Ca22417 | Synonymous-CDS | Zinc finger,RING-type |
| CWSNP8490 | scaffold845 | 154556 | [T/C] | Ca22418 | Intron | Glycosyltransferase,group1 |
| CWSNP8491 | scaffold845 | 205934 | [T/C] | Ca22422 | Synonymous-CDS | PeptidaseS8/S53,subtilisin/kexin/sedolisin |
| CWSNP8492 | scaffold848 | 39934 | [T/G] | - | Intergenic | - |
| CWSNP8493 | scaffold848 | 39984 | [G/T] | - | Intergenic | - |
| CWSNP8494 | scaffold848 | 39985 | [C/T] | - | Intergenic | - |
| CWSNP8495 | scaffold848 | 39990 | [A/G] | - | Intergenic | - |
| CWSNP8496 | scaffold848 | 39994 | [C/T] | - | Intergenic | - |

| SNP IDs | Chromosomes | Physical positions (bp) | SNPs | Gene accession IDs | Sequence components of genes | Putative functions |
|---|---|---|---|---|---|---|
| CWSNP8497 | scaffold848 | 158548 | [T/C] | - | Intergenic | - |
| CWSNP8498 | scaffold848 | 158557 | [G/A] | - | Intergenic | - |
| CWSNP8499 | scaffold848 | 158569 | [C/T] | - | Intergenic | - |
| CWSNP8500 | scaffold848 | 158570 | [A/G] | - | Intergenic | - |
| CWSNP8501 | scaffold848 | 158611 | [C/T] | - | Intergenic | - |
| CWSNP8502 | scaffold848 | 158638 | [G/A] | - | Intergenic | - |
| CWSNP8503 | scaffold848 | 158626 | [G/T] | - | Intergenic | - |
| CWSNP8504 | scaffold848 | 158594 | [A/G] | - | Intergenic | - |
| CWSNP8505 | scaffold8586 | 4415 | [A/G] | - | Intergenic | - |
| CWSNP8506 | scaffold8641 | 472 | [C/T] | - | Intergenic | - |
| CWSNP8507 | scaffold8641 | 460 | [T/C] | - | Intergenic | - |
| CWSNP8508 | scaffold8646 | 2056 | [A/T] | - | Intergenic | - |
| CWSNP8509 | scaffold8646 | 2073 | [G/A] | - | Intergenic | - |
| CWSNP8510 | scaffold8648 | 3977 | [A/G] | - | Intergenic | - |
| CWSNP8511 | scaffold866 | 27279 | [C/T] | - | Intergenic | - |
| CWSNP8512 | scaffold87 | 182788 | [A/C] | Ca23388 | Synonymous-CDS | PeptidaseS8/S53,subtilisin/kexin/sedolisin |

| SNP IDs | Chromosomes | Physical positions (bp) | SNPs | Gene accession IDs | Sequence components of genes | Putative functions |
|---|---|---|---|---|---|---|
| CWSNP8513 | scaffold873 | 265386 | [C/T] | - | URR | - |
| CWSNP8514 | scaffold873 | 265396 | [C/T] | - | URR | - |
| CWSNP8515 | scaffold873 | 265399 | [C/A] | - | URR | - |
| CWSNP8516 | scaffold873 | 265458 | [C/T] | - | URR | - |
| CWSNP8517 | scaffold875 | 49179 | [T/C] | - | Intergenic | - |
| CWSNP8518 | scaffold875 | 51703 | [C/G] | - | DRR | - |
| CWSNP8519 | scaffold875 | 51702 | [T/A] | - | DRR | - |
| CWSNP8520 | scaffold875 | 51701 | [T/C] | - | DRR | - |
| CWSNP8521 | scaffold875 | 51704 | [C/A] | - | DRR | - |
| CWSNP8522 | scaffold875 | 62738 | [A/G] | - | Intergenic | - |
| CWSNP8523 | scaffold877 | 9813 | [G/A] | Ca28083 | Non-Synonymous-CDS | Orotidine5'-phosphate decarboxylase domain |
| CWSNP8524 | scaffold877 | 9851 | [C/T] | Ca28083 | Synonymous-CDS | Orotidine5'-phosphate decarboxylase domain |
| CWSNP8525 | scaffold88 | 9982 | [C/A] | Ca22168 | Intron | Dakkinase |
| CWSNP8526 | scaffold88 | 16398 | [C/T] | Ca22169 | Non-Synonymous-CDS | ZF-HD homeobox protein,Cys/His-rich dimerisation domain |
| CWSNP8527 | scaffold88 | 274006 | [A/G] | Ca22187 | Intron | RNA polymerase II subunit A |
| CWSNP8528 | scaffold882 | 117376 | [G/A] | - | Intergenic | - |

| SNP IDs | Chromosomes | Physical positions (bp) | SNPs | Gene accession IDs | Sequence components of genes | Putative functions |
|---|---|---|---|---|---|---|
| CWSNP8529 | scaffold882 | 118266 | [G/T] | - | Intergenic | - |
| CWSNP8530 | scaffold882 | 232570 | [T/G] | - | Intergenic | - |
| CWSNP8531 | scaffold882 | 358449 | [T/A] | - | Intergenic | - |
| CWSNP8532 | scaffold887_1 | 26134 | [C/A] | Ca17229 | Intron | Forkhead-associated (FHA) domain |
| CWSNP8533 | scaffold887_1 | 26121 | [A/C] | Ca17229 | Intron | Forkhead-associated (FHA) domain |
| CWSNP8534 | scaffold887_1 | 26108 | [G/T] | Ca17229 | Intron | Forkhead-associated (FHA) domain |
| CWSNP8535 | scaffold887_1 | 144885 | [T/C] | Ca17233 | Intron | RNA recognition motif domain |
| CWSNP8536 | scaffold887_1 | 260488 | [A/C] | - | URR | - |
| CWSNP8537 | scaffold892 | 24349 | [T/C] | Ca27454 | Non-Synonymous-CDS | Sugar/inositol transporter |
| CWSNP8538 | scaffold892 | 24323 | [A/G] | Ca27454 | Intron | - |
| CWSNP8539 | scaffold892 | 24317 | [G/T] | Ca27454 | Intron | - |
| CWSNP8540 | scaffold892 | 24316 | [T/A] | Ca27454 | Intron | - |
| CWSNP8541 | scaffold892 | 35527 | [T/C] | Ca27455 | Non-Synonymous-CDS | Sugar/inositol transporter |
| CWSNP8542 | scaffold892 | 35501 | [A/G] | Ca27455 | Synonymous-CDS | - |
| CWSNP8543 | scaffold892 | 35495 | [G/T] | Ca27455 | Synonymous-CDS | - |
| CWSNP8544 | scaffold892 | 35494 | [T/A] | Ca27455 | Non-Synonymous-CDS | - |

| **SNP IDs** | **Chromosomes** | **Physical positions (bp)** | **SNPs** | **Gene accession IDs** | **Sequence components of genes** | **Putative functions** |
|---|---|---|---|---|---|---|
| CWSNP8545 | scaffold892 | 35489 | [A/G] | Ca27455 | Synonymous-CDS | - |
| CWSNP8546 | scaffold892 | 35514 | [G/T] | Ca27455 | Non-Synonymous-CDS | - |
| CWSNP8547 | scaffold895 | 18621 | [C/A] | - | Intergenic | - |
| CWSNP8548 | scaffold895 | 18693 | [G/C] | - | Intergenic | - |
| CWSNP8549 | scaffold895 | 20978 | [T/C] | Ca24246 | Non-Synonymous-CDS | Leucine-rich repeat |
| CWSNP8550 | scaffold895 | 158250 | [A/G] | - | Intergenic | - |
| CWSNP8551 | scaffold901 | 91817 | [C/T] | Ca26439 | Intron | K Homology |
| CWSNP8552 | scaffold901 | 91849 | [T/C] | Ca26439 | Synonymous-CDS | K Homology |
| CWSNP8553 | scaffold908 | 15845 | [C/A] | - | Intergenic | - |
| CWSNP8554 | scaffold908 | 15841 | [G/A] | - | Intergenic | - |
| CWSNP8555 | scaffold908 | 15816 | [C/T] | - | Intergenic | - |
| CWSNP8556 | scaffold908 | 15813 | [G/T] | - | Intergenic | - |
| CWSNP8557 | scaffold908 | 15810 | [A/G] | - | Intergenic | - |
| CWSNP8558 | scaffold908 | 15799 | [C/T] | - | Intergenic | - |
| CWSNP8559 | scaffold908 | 15786 | [G/A] | - | Intergenic | - |
| CWSNP8560 | scaffold908 | 15785 | [G/A] | - | Intergenic | - |

| SNP IDs | Chromosomes | Physical positions (bp) | SNPs | Gene accession IDs | Sequence components of genes | Putative functions |
|---|---|---|---|---|---|---|
| CWSNP8561 | scaffold908 | 15822 | [C/T] | - | Intergenic | - |
| CWSNP8562 | scaffold908 | 15834 | [C/A] | - | Intergenic | - |
| CWSNP8563 | scaffold913 | 332494 | [G/C] | Ca23215 | Non-Synonymous-CDS | - |
| CWSNP8564 | scaffold913 | 403513 | [G/A] | - | Intergenic | - |
| CWSNP8565 | scaffold913 | 403572 | [C/G] | - | Intergenic | - |
| CWSNP8566 | scaffold913 | 403553 | [T/C] | - | Intergenic | - |
| CWSNP8567 | scaffold913 | 403531 | [A/C] | - | Intergenic | - |
| CWSNP8568 | scaffold914 | 53125 | [T/A] | - | Intergenic | - |
| CWSNP8569 | scaffold914 | 53132 | [C/T] | - | Intergenic | - |
| CWSNP8570 | scaffold916 | 1036 | [A/G] | Ca21034 | Non-Synonymous-CDS | Major facilitator superfamily MFS-1 |
| CWSNP8571 | scaffold916 | 275497 | [T/C] | Ca21047 | Intron | C4-dicarboxylate transporter/malicacid transport protein |
| CWSNP8572 | scaffold916 | 275510 | [A/T] | Ca21047 | Intron | C4-dicarboxylate transporter/malicacid transport protein |
| CWSNP8573 | scaffold916 | 275512 | [G/T] | Ca21047 | Intron | C4-dicarboxylate transporter/malicacid transport protein |
| CWSNP8574 | scaffold916 | 275519 | [A/C] | Ca21047 | Intron | C4-dicarboxylate transporter/malicacid transport protein |
| CWSNP8575 | scaffold916 | 275493 | [A/T] | Ca21047 | Intron | C4-dicarboxylate transporter/malicacid transport protein |
| CWSNP8576 | scaffold919 | 119274 | [T/C] | Ca22829 | Intron | - |

| SNP IDs | Chromosomes | Physical positions (bp) | SNPs | Gene accession IDs | Sequence components of genes | Putative functions |
|---|---|---|---|---|---|---|
| CWSNP8577 | scaffold922 | 111921 | [C/A] | Ca27326 | Intron | Protein of unknown function DUF3414 |
| CWSNP8578 | scaffold922 | 121629 | [T/C] | - | Intergenic | - |
| CWSNP8579 | scaffold93 | 205677 | [G/A] | Ca23069 | Synonymous-CDS | Lipoxygenase |
| CWSNP8580 | scaffold93 | 506983 | [C/A] | - | URR | - |
| CWSNP8581 | scaffold93 | 640373 | [A/C] | - | Intergenic | - |
| CWSNP8582 | scaffold93 | 640389 | [G/T] | - | Intergenic | - |
| CWSNP8583 | scaffold93 | 640430 | [C/T] | - | Intergenic | - |
| CWSNP8584 | scaffold93 | 640487 | [A/C] | - | Intergenic | - |
| CWSNP8585 | scaffold93 | 640459 | [G/T] | - | Intergenic | - |
| CWSNP8586 | scaffold93 | 640448 | [T/C] | - | Intergenic | - |
| CWSNP8587 | scaffold93 | 640445 | [C/T] | - | Intergenic | - |
| CWSNP8588 | scaffold93 | 640444 | [G/T] | - | Intergenic | - |
| CWSNP8589 | scaffold93 | 645328 | [A/C] | - | Intergenic | - |
| CWSNP8590 | scaffold93 | 645344 | [G/T] | - | Intergenic | - |
| CWSNP8591 | scaffold93 | 645442 | [A/C] | - | Intergenic | - |
| CWSNP8592 | scaffold93 | 645414 | [G/T] | - | Intergenic | - |

| SNP IDs | Chromosomes | Physical positions (bp) | SNPs | Gene accession IDs | Sequence components of genes | Putative functions |
|---|---|---|---|---|---|---|
| CWSNP8593 | scaffold93 | 645403 | [T/C] | - | Intergenic | - |
| CWSNP8594 | scaffold93 | 645400 | [C/T] | - | Intergenic | - |
| CWSNP8595 | scaffold93 | 645399 | [G/T] | - | Intergenic | - |
| CWSNP8596 | scaffold93 | 645402 | [G/A] | - | Intergenic | - |
| CWSNP8597 | scaffold93 | 651020 | [C/T] | - | Intergenic | - |
| CWSNP8598 | scaffold93 | 651018 | [C/T] | - | Intergenic | - |
| CWSNP8599 | scaffold93 | 650981 | [T/C] | - | Intergenic | - |
| CWSNP8600 | scaffold93 | 650968 | [G/A] | - | Intergenic | - |
| CWSNP8601 | scaffold93 | 650958 | [G/A] | - | Intergenic | - |
| CWSNP8602 | scaffold93 | 650955 | [T/C] | - | Intergenic | - |
| CWSNP8603 | scaffold931 | 13212 | [T/C] | - | Intergenic | - |
| CWSNP8604 | scaffold9323 | 5263 | [A/G] | - | Intergenic | - |
| CWSNP8605 | scaffold937 | 132087 | [C/G] | - | DRR | - |
| CWSNP8606 | scaffold937 | 134580 | [C/G] | Ca25988 | Intron | Vacuolar ATPase assembly integral membrane protein VMA21-like domain |
| CWSNP8607 | scaffold937 | 134707 | [G/A] | Ca25988 | Intron | Vacuolar ATPase assembly integral membrane protein VMA21-like domain |
| CWSNP8608 | scaffold948 | 12188 | [C/T] | - | URR | - |

| SNP IDs | Chromosomes | Physical positions (bp) | SNPs | Gene accession IDs | Sequence components of genes | Putative functions |
|---|---|---|---|---|---|---|
| CWSNP8609 | scaffold948 | 21152 | [C/T] | Ca27369 | Non-Synonymous-CDS | AThook,DNA-binding motif |
| CWSNP8610 | scaffold953 | 42329 | [T/C] | - | Intergenic | - |
| CWSNP8611 | scaffold953 | 57413 | [A/T] | - | Intergenic | - |
| CWSNP8612 | scaffold956 | 21441 | [C/A] | - | Intergenic | - |
| CWSNP8613 | scaffold956 | 27710 | [T/G] | - | Intergenic | - |
| CWSNP8614 | scaffold956 | 27693 | [T/C] | - | Intergenic | - |
| CWSNP8615 | scaffold956 | 28015 | [A/C] | - | Intergenic | - |
| CWSNP8616 | scaffold956 | 28014 | [T/A] | - | Intergenic | - |
| CWSNP8617 | scaffold962 | 149524 | [C/T] | Ca26502 | Non-Synonymous-CDS | - |
| CWSNP8618 | scaffold962 | 149545 | [C/T] | Ca26502 | Non-Synonymous-CDS | - |
| CWSNP8619 | scaffold962 | 149580 | [G/A] | Ca26502 | Non-Synonymous-CDS | - |
| CWSNP8620 | scaffold962 | 149585 | [A/G] | Ca26502 | Synonymous-CDS | - |
| CWSNP8621 | scaffold962 | 149645 | [G/T] | Ca26502 | Synonymous-CDS | - |
| CWSNP8622 | scaffold962 | 149631 | [G/A] | Ca26502 | Non-Synonymous-CDS | - |
| CWSNP8623 | scaffold962 | 149589 | [G/A] | Ca26502 | Non-Synonymous-CDS | - |
| CWSNP8624 | scaffold962 | 149583 | [G/A] | Ca26502 | Non-Synonymous-CDS | - |

| SNP IDs | Chromosomes | Physical positions (bp) | SNPs | Gene accession IDs | Sequence components of genes | Putative functions |
|---|---|---|---|---|---|---|
| CWSNP8625 | scaffold962 | 149577 | [G/T] | Ca26502 | Non-Synonymous-CDS | - |
| CWSNP8626 | scaffold962 | 149689 | [G/A] | Ca26502 | Non-Synonymous-CDS | - |
| CWSNP8627 | scaffold962 | 149687 | [A/T] | Ca26502 | Synonymous-CDS | - |
| CWSNP8628 | scaffold962 | 149676 | [T/C] | Ca26502 | Non-Synonymous-CDS | - |
| CWSNP8629 | scaffold962 | 149661 | [G/A] | Ca26502 | Non-Synonymous-CDS | - |
| CWSNP8630 | scaffold962 | 149649 | [C/T] | Ca26502 | Non-Synonymous-CDS | - |
| CWSNP8631 | scaffold962 | 149628 | [G/A] | Ca26502 | Non-Synonymous-CDS | - |
| CWSNP8632 | scaffold962 | 149613 | [G/T] | Ca26502 | Non-Synonymous-CDS | - |
| CWSNP8633 | scaffold963 | 77099 | [C/T] | - | Intergenic | - |
| CWSNP8634 | scaffold98 | 287956 | [T/A] | - | Intergenic | - |
| CWSNP8635 | scaffold98 | 287933 | [T/C] | - | Intergenic | - |
| CWSNP8636 | scaffold98 | 440243 | [G/C] | - | Intergenic | - |
| CWSNP8637 | scaffold98 | 562979 | [G/A] | Ca21399 | Intron | Zinc finger,FYVE-type |
| CWSNP8638 | scaffold981 | 84088 | [C/G] | Ca27591 | Non-Synonymous-CDS | Endonuclease/exonuclease/phosphatase |
| CWSNP8639 | scaffold981 | 84072 | [G/A] | Ca27591 | Non-Synonymous-CDS | Endonuclease/exonuclease/phosphatase |
| CWSNP8640 | scaffold981 | 84057 | [A/G] | Ca27591 | Non-Synonymous-CDS | Endonuclease/exonuclease/phosphatase |

| SNP IDs | Chromosomes | Physical positions (bp) | SNPs | Gene accession IDs | Sequence components of genes | Putative functions |
|---|---|---|---|---|---|---|
| CWSNP8641 | scaffold981 | 84007 | [C/G] | Ca27591 | Intron | Endonuclease/exonuclease/phosphatase |
| CWSNP8642 | scaffold999 | 77761 | [A/T] | - | Intergenic | - |
| CWSNP8643 | scaffold999 | 77763 | [C/T] | - | Intergenic | - |
| CWSNP8644 | scaffold999 | 77767 | [A/T] | - | Intergenic | - |
| CWSNP8645 | C11058086 | 341 | [G/A] | - | Intergenic | - |
| CWSNP8646 | C11062436 | 381 | [T/A] | - | Intergenic | - |
| CWSNP8647 | C11079766 | 1418 | [A/C] | - | Intergenic | - |
| CWSNP8648 | C11079766 | 1422 | [G/C] | - | Intergenic | - |
| CWSNP8649 | C11079766 | 1412 | [A/T] | - | Intergenic | - |
| CWSNP8650 | C11080716 | 1568 | [A/C] | - | Intergenic | - |
| CWSNP8651 | C11084624 | 656 | [G/A] | - | Intergenic | - |
| CWSNP8652 | C11086066 | 641 | [A/C] | - | Intergenic | - |
| CWSNP8653 | C11086066 | 624 | [A/C] | - | Intergenic | - |
| CWSNP8654 | C11132768 | 2177 | [G/A] | - | Intergenic | - |
| CWSNP8655 | C11142252 | 3299 | [A/C] | Ca27871 | Intron | Ribosomal proteinS7 |
| CWSNP8656 | C11143608 | 1389 | [A/T] | Ca27926 | Intron | DNA-directed RNA polymerase, subunit 2,domain 6 |

| SNP IDs | Chromosomes | Physical positions (bp) | SNPs | Gene accession IDs | Sequence components of genes | Putative functions |
|---|---|---|---|---|---|---|
| CWSNP8657 | C11147634 | 3004 | [C/A] | Ca28253 | Intron | Domain of unknown function DUF231,plant |
| CWSNP8658 | C11147634 | 3028 | [C/T] | Ca28253 | Intron | Domain of unknown function DUF231,plant |
| CWSNP8659 | C11149092 | 2474 | [T/A] | - | Intergenic | - |
| CWSNP8660 | C11149092 | 3190 | [T/G] | - | Intergenic | - |
| CWSNP8661 | C11149092 | 3284 | [C/G] | - | Intergenic | - |
| CWSNP8662 | C11157320 | 766 | [C/G] | Ca28268 | Intron | GCN5-relatedN-acetyltransferase (GNAT) domain |
| CWSNP8663 | C11157320 | 2169 | [T/A] | Ca28268 | Intron | GCN5-relatedN-acetyltransferase (GNAT) domain |
| CWSNP8664 | C11157320 | 2152 | [G/C] | Ca28268 | Intron | GCN5-relatedN-acetyltransferase (GNAT) domain |
| CWSNP8665 | C11160518 | 4522 | [A/G] | Ca28216 | Non-Synonymous-CDS | CytochromeP450 |
| CWSNP8666 | C11164954 | 5784 | [G/A] | Ca28056 | Non-Synonymous-CDS | Protein of unknown function DUF2930 |
| CWSNP8667 | C11165890 | 154 | [C/G] | - | Intergenic | - |
| CWSNP8668 | C11175430 | 3929 | [T/G] | Ca27727 | Non-Synonymous-CDS | ABC transporter, transmembrane domain |
| CWSNP8669 | C11175430 | 3936 | [G/T] | Ca27727 | Non-Synonymous-CDS | ABC transporter, transmembrane domain |
| CWSNP8670 | C11175430 | 3952 | [T/C] | Ca27727 | Non-Synonymous-CDS | ABC transporter, transmembrane domain |
| CWSNP8671 | C11177172 | 2529 | [T/G] | Ca28257 | Intron | U3 small nucleolar RNA-associated protein 10 |
| CWSNP8672 | C11177172 | 2515 | [T/A] | Ca28257 | Intron | U3 small nucleolar RNA-associated protein 10 |

| SNP IDs | Chromosomes | Physical positions (bp) | SNPs | Gene accession IDs | Sequence components of genes | Putative functions |
|---|---|---|---|---|---|---|
| CWSNP8673 | C11177172 | 5324 | [G/A] | Ca28257 | Intron | U3 small nucleolar RNA-associated protein 10 |
| DRR: Downstream regulatory region | | | | | | |
| URR: Upstream regulatory region | | | | | | |
| CDS: Coding sequences | | | | | | |
| CWSNP: Cultivated Wild SNP | | | | | | |
