# Supplementary material for: A Genome-wide Combinatorial Strategy Dissects Complex Genetic Architecture of Seed Coat Color in Chickpea
Source: Front Plant Sci. 2015 Nov 17;6:979. doi: 10.3389/fpls.2015.00979 (PMC4647070; doi:10.3389/fpls.2015.00979)
Supplement: Supplementary file 3 [file Table3.PDF]

Table S3: Polymorphism and nucleotide diversity potential of GBS-based genome-wide and candidate gene-derived SNPs in 172 cultivated and wild chickpea accessions estimated by diverse polymorphism and diversity statistical measures

**Accessions/species used**	**Number (%) of SNPs showing polymorphism**	**Mean polymorphism information content (PIC)**	**Nucleotide diversity**		
			**θπ**	**θω**	**Tajima' D**
All 172 cultivated and wild accessions	9045	0.32	2.14	2.19	-1.59
Within cultivated species (among 93 *desi* and *kabuli* accessions)	8837 (97.7)	0.20	1.83	1.94	-2.37
Within wild species (among 79 wild accessions)	8920 (98.6)	0.26	2.51	2.38	-1.23
Among cultivated and wild species	9012 (99.6)	0.28	2.71	2.53	-0.87

θπ: Average pair-wise nucleotide diversity

θω: Watterson's estimator of segregating sites
