# Supplementary material for: A Genome-wide Combinatorial Strategy Dissects Complex Genetic Architecture of Seed Coat Color in Chickpea
Source: Front Plant Sci. 2015 Nov 17;6:979. doi: 10.3389/fpls.2015.00979 (PMC4647070; doi:10.3389/fpls.2015.00979)
Supplement: Supplementary file 4 [file Table4.PDF]

Table S4: Comparison of nucleotide diversity measures between two seed colour-associated MATE secondary transporter genes identified by candidate gene-based association mapping and GWAS

**Seed colour-associated MATE secondary transporter genes**	**Associated chickpea seed coat colour**	**Seed colour represented by maximum chickpea accessions**	**Theta w (θω) Kb^{-1}**	**Theta Pi (θπ) Kb^{-1}**	**Tajima's D**
tt12-Ca05557[a]	BE	*Kabuli*	1.93	1.95	1.82
	LB/YB	*Desi* and *C. reticulatum*	0.79	0.82	-2.63
tt12-Ca18123	BE	*Kabuli*	1.96	1.98	1.90
	LB/YB	*Desi* and *C. reticulatum*	0.73	0.77	-2.89

[a]Gene located at the selective sweep region of *kabuli* chromosome 4 reported by Varshney et al. (2013)
