# Supplementary material for: A Genome-wide Combinatorial Strategy Dissects Complex Genetic Architecture of Seed Coat Color in Chickpea
Source: Front Plant Sci. 2015 Nov 17;6:979. doi: 10.3389/fpls.2015.00979 (PMC4647070; doi:10.3389/fpls.2015.00979)
Supplement: Supplementary file 5 [file Table5.PDF]

Table S5: Markers mapped on eight LGs of an intra-specific chickpea genetic linkage map

**Linkage groups (LGs)/chromosomes (Chr)**	**SNP + SSR markers mapped**	**Map length covered (cM)**	**Mean inter-marker distance (cM)**
LG(Chr)01	62 + 8 = 70	134.4	1.92
LG(Chr)02	65 + 5 = 70	159.4	2.28
LG(Chr)03	46 + 4 = 70	169.8	3.40
LG(Chr)04	80 + 4 = 84	189.3	2.25
LG(Chr)05	29 + 3 = 32	127.9	4.0
LG(Chr)06	28 + 4 = 32	90.6	2.83
LG(Chr)07	47 + 3 = 50	105.8	2.12
LG(Chr)08	25+ 2 = 27	88.5	3.28
**Total**	**382 + 33 = 415**	**1065.7**	**2.57**
