# Supplementary material for: A Genome-wide Combinatorial Strategy Dissects Complex Genetic Architecture of Seed Coat Color in Chickpea
Source: Front Plant Sci. 2015 Nov 17;6:979. doi: 10.3389/fpls.2015.00979 (PMC4647070; doi:10.3389/fpls.2015.00979)
Supplement: Supplementary file 6 [file Table6.PDF]

Table S6: Significant QTLs governing seed coat colour identified and mapped on LGs of a high-density intra-specific chickpea genetic map (ICC 12299 x ICC 8261)

QTLs	LGs/chromosomes	Marker intervals with genetic positions (cM)	Markers associated with QTLs	2012			2013		
				LOD	PVE (R^2%)	A	LOD	PVE (R^2%)	A
CaqSC2.1[a]	*Ca_Kabuli*_Chr01	CWSNP1273 (25.2) to CWSNP1277 (30.2)	CWSNP1275	10.2	35.6	4.1	11.5	38.7	-3.9
CaqSC4.1	*Ca_Kabuli*_Chr02	CWSNP3060 (29.5) to CWSNP3063 (32.1)	gSNP4	5.6	20.1	2.5	6.3	22.3	-2.8
CaqSC4.2	*Ca_Kabuli*_Chr03	CWSNP4021 (127.6) to CWSNP4023 (130.9)	gSNP5	8.1	29.5	1.9	6.7	30.6	-2.5
CaqSC6.1	*Ca_Kabuli*_Chr04	CWSNP5266 (154.9) to CWSNP5270 (157.5)	gSNP6	9.0	31.4	2.9	8.8	32.5	-2.7
CaqSC7.1	*Ca_Kabuli*_Chr05	CWSNP6260 (24.7) to CWSNP6262 (27.7)	CWSNP6261	6.7	25.5	2.4	7.1	29.7	-3.1

**CaqSC2.1* (*Cicer arietinum* QTL for seed coat colour on chromosome 2 number 1), PVE: Percentage of phenotypic variation explained by QTLs, A: Additive effect of alleles from ICC 8261 with BE seed coat colour trait. Details regarding CWSNP and gSNP markers are provided in the Table S2. [a]known QTLs for seed coat color reported previously by Hossain et al. (2011).
