# Supplementary material for: A Genome-wide Combinatorial Strategy Dissects Complex Genetic Architecture of Seed Coat Color in Chickpea
Source: Front Plant Sci. 2015 Nov 17;6:979. doi: 10.3389/fpls.2015.00979 (PMC4647070; doi:10.3389/fpls.2015.00979)
Supplement: Supplementary file 7 [file Table7.PDF]

Table S7: Primers used for *MATE* gene expression analysis and *MATE* gene-derived SSR marker genotyping in chickpea

**SNP IDs**	**Gene accession IDs**	**Forward Primer sequences (5'-3')**	**Reverse Primer sequences (5'-3')**	**Actual annealing temperature (0C)**	**Size (bp) of alleles amplified**
Inositol monophosphate-QPCR	Ca00233	GGCAGATTTTGGAGTTCAGG	CCCCTCTATCAATTGCCTCA	59.7	203
Disease resistance protein-QPCR	Ca00260	GATTTCCCAAAGCAAAAGCA	GGGCAGTTAGAAATGGCAAA	60.2	200
MATE secondary transporter-QPCR	Ca18123	ATGTGGCTTGGTTAGTTTTGTG	GACCACAATTCTCTACAATGACAAG	61.0	226
MATE secondary transporter-QPCR	Ca10158	TATGTTGGGAATGGCAAGTG	GCCTTGCTCAGCTATGCTCT	59.4	199
Chalcone synthase-QPCR	Ca08294	TAGTTGGACAGGCGCTTTTT	TCGAAATAATCCCTGGAACG	59.9	204
Dihydroflavonol-4-reductase-QPCR	Ca10786	GAAGGCCCAAACTGTTCGTA	CTTTCGCAAATTTCCATGCT	60.1	194
Glutathione-S-reductase-QPCR	Ca03442	AGGGTTGTGGTCAGAAGTGG	TGAAACATTCACCTCCCAAA	60.0	200
MATE secondary transporter-QPCR	Ca05557	TGCAGTAATGGTTTGCCTTG	TTCAAATGGATTCCCAGCTC	59.7	202
Anthocyanin reductase-QPCR	Ca05336	TGCAGTTTCTGTCTGGTTCG	GGGGCAATCATCAAATTCAG	60.0	197
Ribonuclease T2-QPCR	Ca25968	ACCAACACCATTCCCACAAC	GCCATAGGCCATGAATTTTG	60.5	212
Domain of unknown function DUF231-QPCR	Ca23042	CTATGGAACCAACAGCAGCA	GACGATGTTGACCGATGATG	59.9	191
Leucoanthocyanidin dioxygenase-QPCR	Ca02395	CAAGCGAATATGCAAAGCAA	ATGGGCTTCAACTCCAAGTG	60.0	191
Integrase-QPCR	Ca23085	TCCTCCTAAAACCCCTCAGC	CCAAATCTTGTCTGGCACCT	60.6	193
MATE-SSR	Ca18123	TTGACTTATGCATTTTATTTGC	AAAACAGCATACCCAAAATAAC	55.0	350
