# Supplementary material for: A Genome-wide Combinatorial Strategy Dissects Complex Genetic Architecture of Seed Coat Color in Chickpea
Source: Front Plant Sci. 2015 Nov 17;6:979. doi: 10.3389/fpls.2015.00979 (PMC4647070; doi:10.3389/fpls.2015.00979)
Supplement: Supplementary file 8 [file Table8.PDF]

Table S8: SNP and SSR allelic variants mined from *MATE* gene for molecular haplotyping

**SNP IDs**	**Chromosomes**	**Physical positions (bp)**	**SNPs/SSR repeat-motifs**	**Gene accession IDs**	**Sequence components of genes**	**Putative functions**
MATE-SNP01	Ca-Kabuli-Chr2	7699252	[C/A]	Ca18123	URR	Multiantimicrobial extrusion protein
MATE-SSR	Ca-Kabuli-Chr2	7700046-7700057	[GTTG]3	Ca18123	CDS	Multiantimicrobial extrusion protein
MATE-SNP02/CWSNP1275	Ca-Kabuli-Chr2	7701199	[T/G]	Ca18123	Non-Synonymous-CDS	Multiantimicrobial extrusion protein
MATE-SNP03/CWSNP1276	Ca-Kabuli-Chr2	7701230	[T/G]	Ca18123	Synonymous-CDS	Multiantimicrobial extrusion protein
MATE-SNP04/CWSNP1277	Ca-Kabuli-Chr2	7702468	[A/T]	Ca18123	Intron	Multiantimicrobial extrusion protein
