# Supplementary material for: A Genome-wide Combinatorial Strategy Dissects Complex Genetic Architecture of Seed Coat Color in Chickpea
Source: Front Plant Sci. 2015 Nov 17;6:979. doi: 10.3389/fpls.2015.00979 (PMC4647070; doi:10.3389/fpls.2015.00979)
Supplement: Supplementary file 9 [file Image1.PDF]

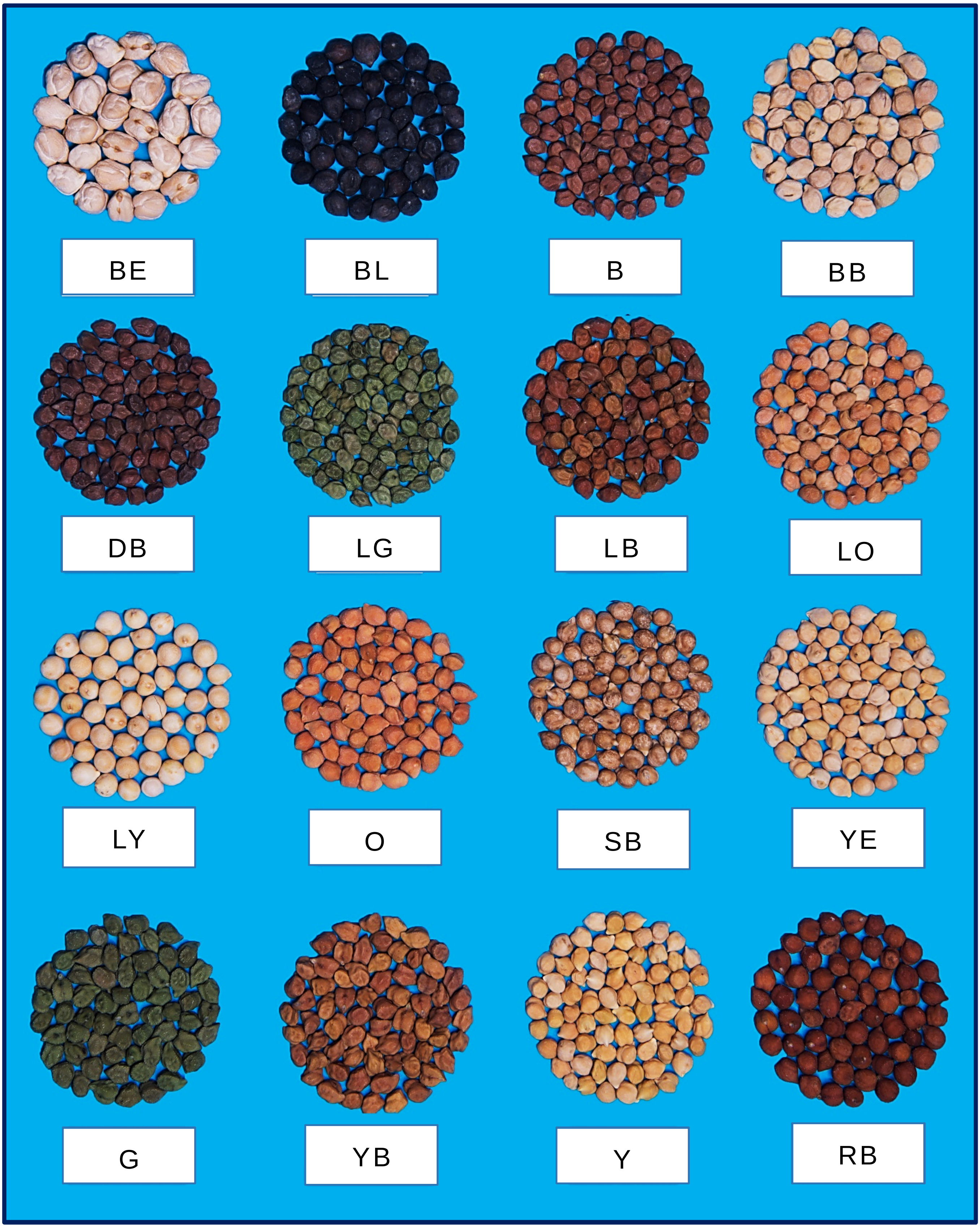

Figure S1: Diverse predominant 16 representative seed coat colour types visually estimated and characterized in mature seeds of 93 cultivated and 79 wild chickpea accessions by their multi-location replicated field phenotyping.
