# Supplementary material for: A Genome-wide Combinatorial Strategy Dissects Complex Genetic Architecture of Seed Coat Color in Chickpea
Source: Front Plant Sci. 2015 Nov 17;6:979. doi: 10.3389/fpls.2015.00979 (PMC4647070; doi:10.3389/fpls.2015.00979)
Supplement: Supplementary file 11 [file Image3.PDF]

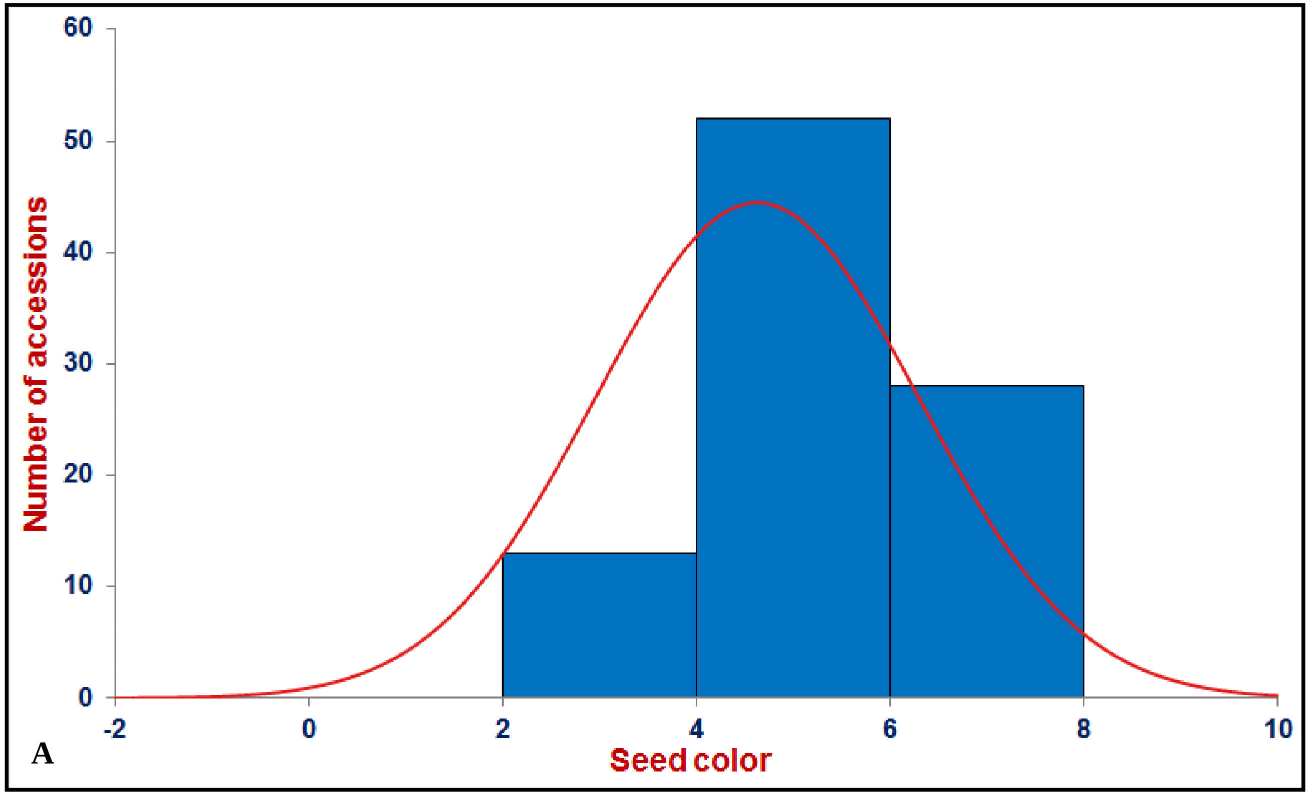

60
50
40
30
20
10
0
Number of accessions
-2
0
2
4
6
8
10
Seed color
A

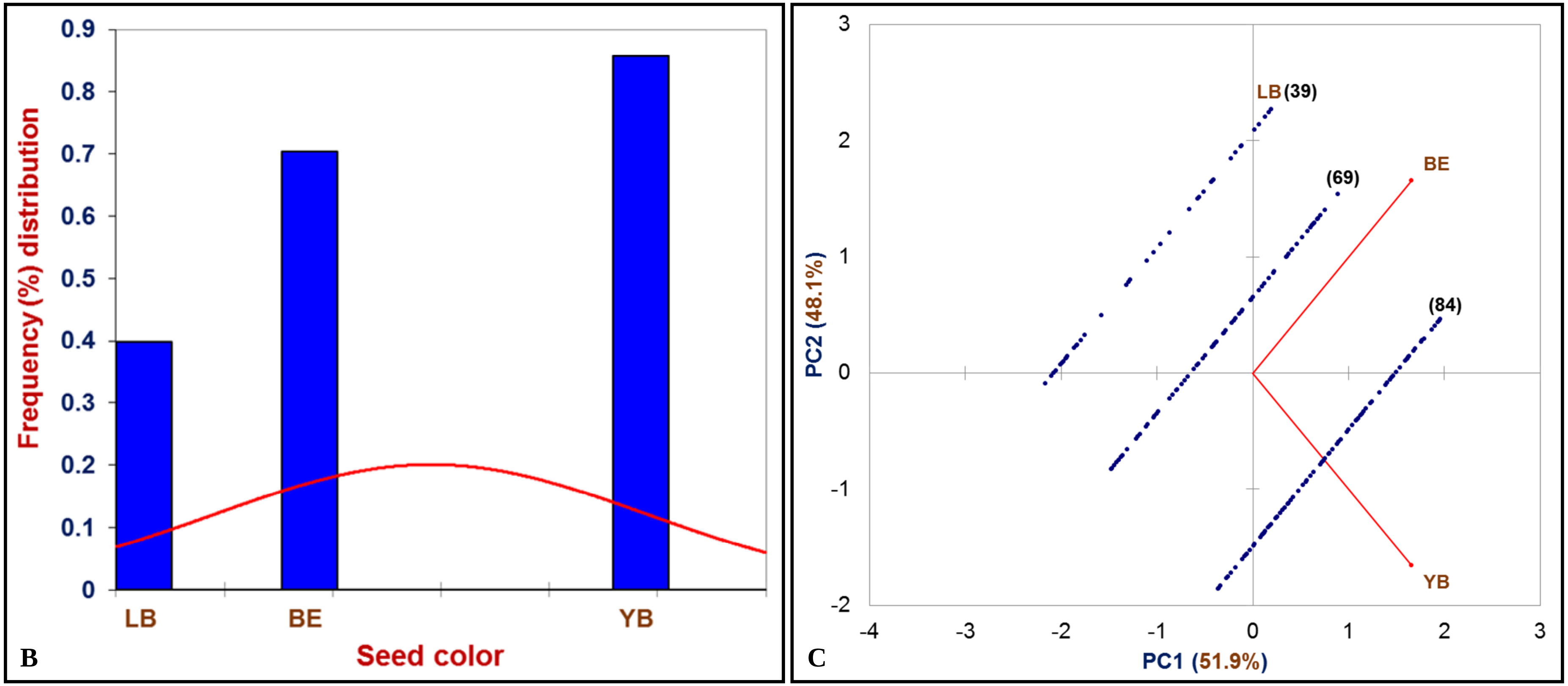

Figure S3: Frequency distribution of seed coat colour trait in 93 cultivated *desi* and *kabuli* chickpea accessions **(A)** and 190 F_7 RIL mapping individuals (ICC 12299 x ICC 8261) and parental accessions **(B)** illustrated a goodness of fit to the normal distribution. **(C)** PCA depicting the frequency distribution of seed coat colour trait among 190 RIL mapping individuals and parental accessions, and classified these accessions into two major (BE and YB) and one segregating (LB) seed coat colour groups.
