# Supplementary material for: A Genome-wide Combinatorial Strategy Dissects Complex Genetic Architecture of Seed Coat Color in Chickpea
Source: Front Plant Sci. 2015 Nov 17;6:979. doi: 10.3389/fpls.2015.00979 (PMC4647070; doi:10.3389/fpls.2015.00979)
Supplement: Supplementary file 12 [file Image4.PDF]

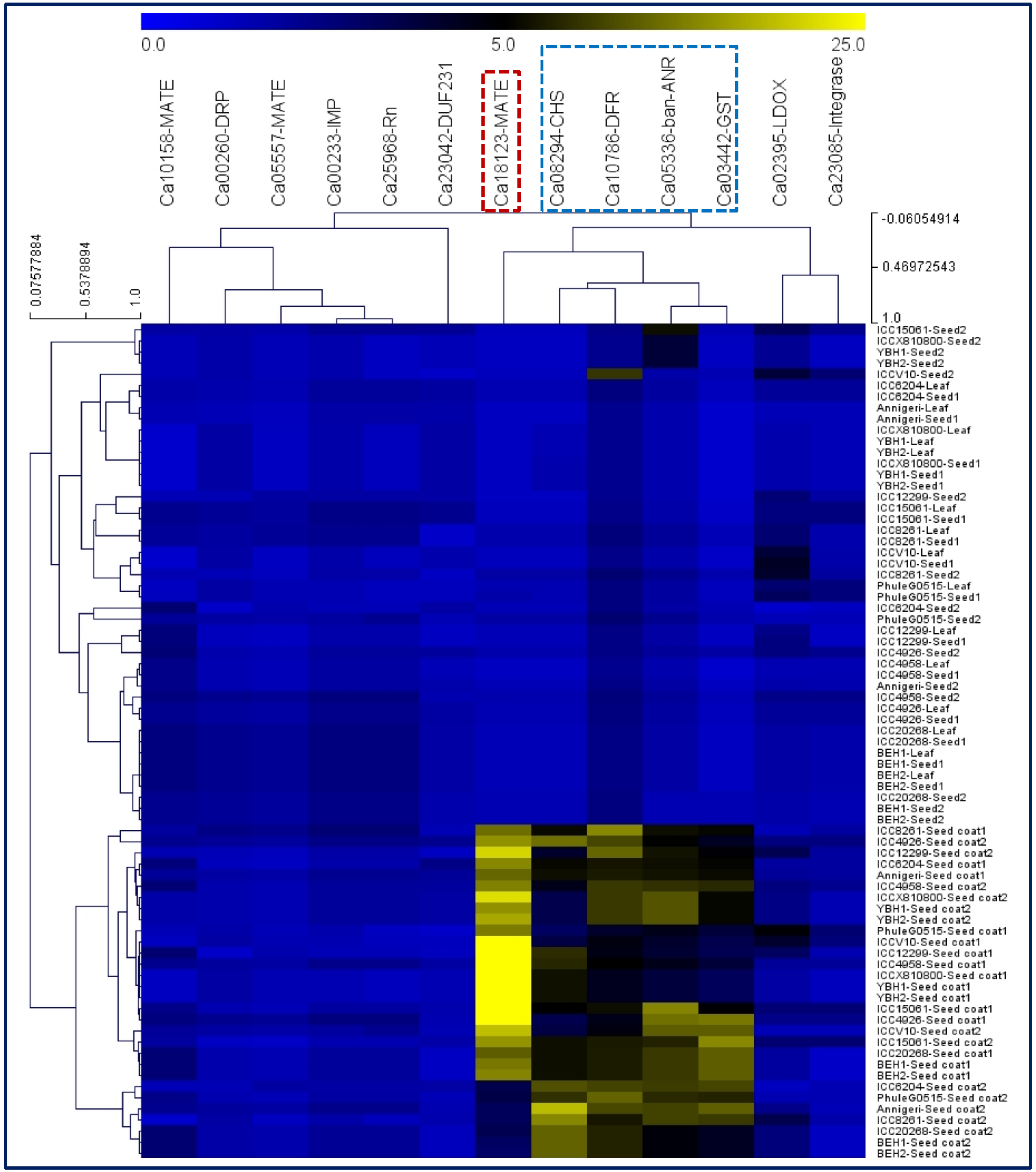
0.0
5.0
25.0
Ca10158-MATE
Ca00260-DRP
Ca05557-MATE
Ca00233-IMP
Ca25968-Rn
Ca23042-DUF231
Ca18123-MATE
Ca08294-CHS
Ca10786-DFR
Ca05336-ban-ANR
Ca03442-GST
Ca02395-LDOX
Ca23085-Integrase
-0.06054914
0.46972543
1.0
0.07577884
0.5378894
1.0
ICC15061-Seed2
ICCX810800-Seed2
YBH1-Seed2
YBH2-Seed2
ICCV10-Seed2
ICC6204-Leaf
ICC6204-Seed1
Annigeri-Leaf
Annigeri-Seed1
ICCX810800-Leaf
YBH1-Leaf
YBH2-Leaf
ICCX810800-Seed1
YBH1-Seed1
YBH2-Seed1
ICC12299-Seed2
ICC15061-Leaf
ICC15061-Seed1
ICC8261-Leaf
ICC8261-Seed1
ICCV10-Leaf
ICCV10-Seed1
ICC8261-Seed2
PhuleG0515-Leaf
PhuleG0515-Seed1
ICC6204-Seed2
PhuleG0515-Seed2
ICC12299-Leaf
ICC12299-Seed1
ICC4926-Seed2
ICC4958-Leaf
ICC4958-Seed1
Annigeri-Seed2
ICC4958-Seed2
ICC4926-Leaf
ICC4926-Seed1
ICC20268-Leaf
ICC20268-Seed1
BEH1-Leaf
BEH1-Seed1
BEH2-Leaf
BEH2-Seed1
ICC20268-Seed2
BEH1-Seed2
BEH2-Seed2
ICC8261-Seed coat1
ICC4926-Seed coat2
ICC12299-Seed coat2
ICC6204-Seed coat1
Annigeri-Seed coat1
ICC4958-Seed coat2
ICCX810800-Seed coat2
YBH1-Seed coat2
YBH2-Seed coat2
PhuleG0515-Seed coat1
ICCV10-Seed coat1
ICC12299-Seed coat1
ICC4958-Seed coat1
ICCX810800-Seed coat1
YBH1-Seed coat1
YBH2-Seed coat1
ICC15061-Seed coat1
ICC4926-Seed coat1
ICCV10-Seed coat2
ICC15061-Seed coat2
ICC20268-Seed coat1
BEH1-Seed coat1
BEH2-Seed coat1
ICC6204-Seed coat2
PhuleG0515-Seed coat2
Annigeri-Seed coat2
ICC8261-Seed coat2
ICC20268-Seed coat2
BEH1-Seed coat2
BEH2-Seed coat2

Figure S4: Hierarchical cluster display depicting the differential expression profiles of 13, including five seed colour-associated genes (validated by both association and QTL mapping) in vegetative leaf tissues, seeds without seed coats and seed coats scrapped from the immature and mature seeds of 11 contrasting chickpea accessions as well as mapping parental accessions and two homozygous RIL individuals representing two predominant seed coat colour types, LB/YB (*desi*: ICCX810800, *desi*: ICC 12299, *desi*: ICCV10, *desi*: ICC 4958, *desi*: ICC 15061, *kabuli*: ICC 6204 and *kabuli*: Annigeri) and BE (*kabuli*: ICC 20268, *kabuli*: ICC 8261, *kabuli*: Phule G0515 and *desi*: ICC 4926) using quantitative RT-PCR assay. The colour scale at the top represents average log signal expression values of genes in various tissues and developmental stages; in which blue, black and yellow color signify low, medium and high level of expression, respectively. One MATE secondary transporter *kabuli* gene (Ca18123) displaying pronounced differential down-regulation specifically in the mature/immature seed coats of BE seed coloured chickpea accessions during seed development is marked with red boxes. Four seed colour known cloned genes showing seed coat-specific differential (up/down-regulated) expression in mature and immature seed coats of LB/YB and BE seed coloured *desi* and *kabuli* accessions are indicated with blue box. The details regarding identities of genes are provided in the Tables 1 and 2. The tissues and genes used for expression profiling are depicted on the right and top side of expression map, respectively. The endogenous control *elongation factor-1 alpha* was used in quantitative RT-PCR assay to normalize the expression values across different tissues/developmental stages of accessions used. Seed1: Immature seed coat at seed development stage 1 (15-25 DAP) and Seed2: Mature seed coat at seed development stage 2 (26-36 DAP). YBH (yellow brown homozygous) and BEH (beige homozygous) RIL mapping individuals.
