# Supplementary material for: A Genome-wide Combinatorial Strategy Dissects Complex Genetic Architecture of Seed Coat Color in Chickpea
Source: Front Plant Sci. 2015 Nov 17;6:979. doi: 10.3389/fpls.2015.00979 (PMC4647070; doi:10.3389/fpls.2015.00979)
Supplement: Supplementary file 10 [file Image2.PDF]

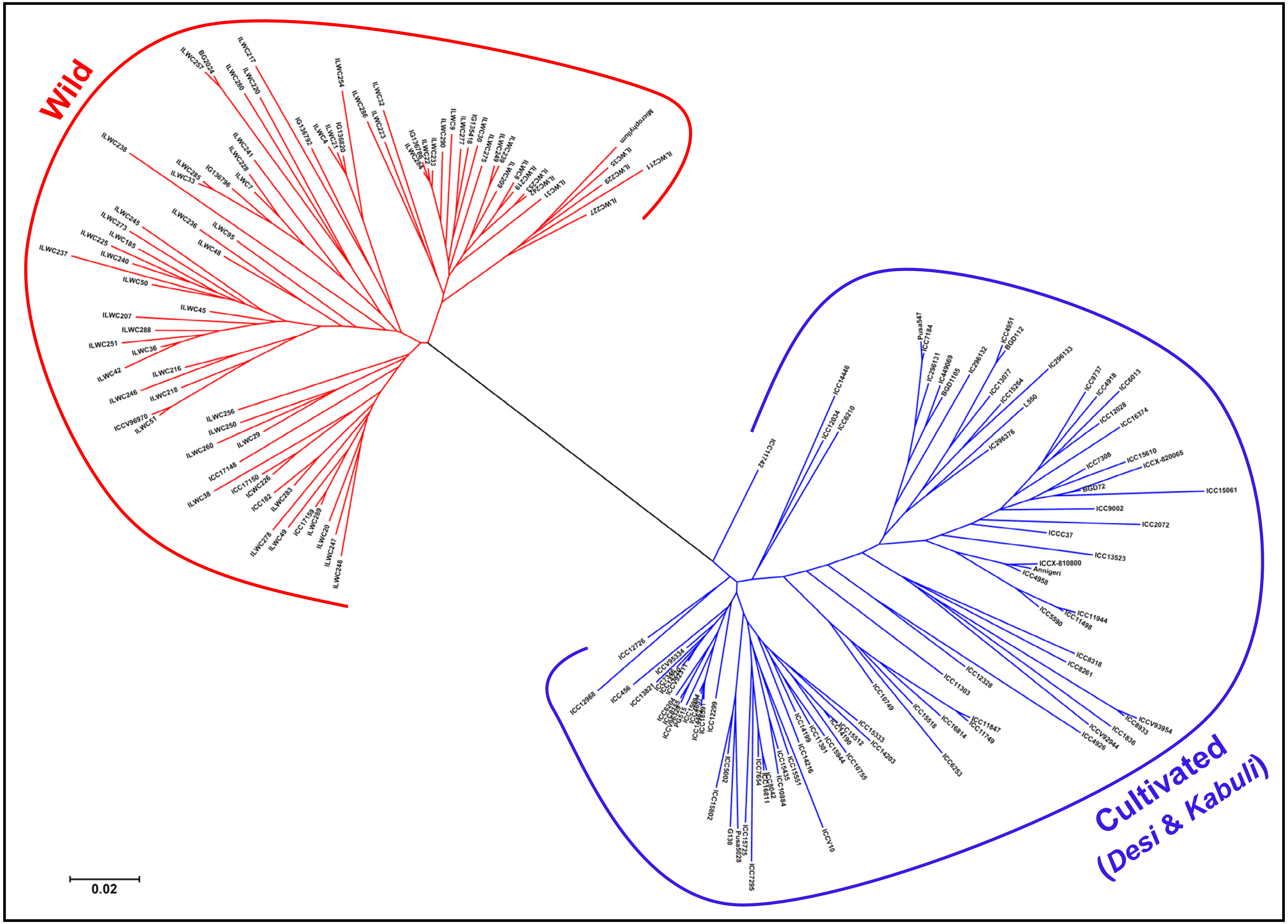

Figure S2: The Nei's genetic distance-based unrooted phylogram illustrating the genetic relationships among 172, including 93 cultivated (*desi* and *kabuli*) and 79 wild chickpea accessions using 9045 high-quality genome-wide GBS- and candidate gene-based SNPs.
